# The Development of Bacteriophage Resistance in *Vibrio alginolyticus* Depends on a Complex Metabolic Adaptation Strategy

**DOI:** 10.3390/v13040656

**Published:** 2021-04-10

**Authors:** Dimitrios Skliros, Panos G. Kalatzis, Chrysanthi Kalloniati, Fotios Komaitis, Sokratis Papathanasiou, Evangelia D. Kouri, Michael K. Udvardi, Constantina Kokkari, Pantelis Katharios, Emmanouil Flemetakis

**Affiliations:** 1 Laboratory of Molecular Biology, Department of Biotechnology, School of Applied Biology and Biotechnology, Agricultural University of Athens, 1855 Athens, Greece; dsklhros@gmail.com (D.S.); xkalloni@gmail.com (C.K.); fotiskomaitis@hotmail.com (F.K.); sokospapathanasiou@hotmail.com (S.P.); 2 Department of Biology, Marine Biological Section, University of Copenhagen, 3000 Helsingør, Denmark; panos.kalatzis@bio.ku.dk; 3 The Noble Research Institute, 2510 Sam Noble Pkwy, Ardmore, OK 73401, USA; edkouri@gmail.com (E.D.K.); mudvardi@noble.org (M.K.U.); 4 Institute of Marine Biology, Biotechnology and Aquaculture, Hellenic Centre for Marine Research, 71500 Heraklion, Greece; dkok@hcmr.gr (C.K.); katharios@hcmr.gr (P.K.)

**Keywords:** host–phage interaction, receptors, transporters, *Vibrio alginolyticus*, acquired phage resistance, metabolic reprogramming, bacteriophages, host metabolism

## Abstract

Lytic bacteriophages have been well documented to play a pivotal role in microbial ecology due to their complex interactions with bacterial species, especially in aquatic habitats. Although the use of phages as antimicrobial agents, known as phage therapy, in the aquatic environment has been increasing, recent research has revealed drawbacks due to the development of phage-resistant strains among Gram-negative species. Acquired phage resistance in marine *Vibrios* has been proven to be a very complicated process utilizing biochemical, metabolic, and molecular adaptation strategies. The results of our multi-omics approach, incorporating transcriptome and metabolome analyses of *Vibrio alginolyticus* phage-resistant strains, corroborate this prospect. Our results provide insights into phage-tolerant strains diminishing the expression of phage receptors *ompF*, *lamB*, and *btuB*. The same pattern was observed for genes encoding natural nutrient channels, such as *rbsA*, *ptsG*, *tryP*, *livH*, *lysE*, and *hisp*, meaning that the cell needs to readjust its biochemistry to achieve phage resistance. The results showed reprogramming of bacterial metabolism by transcript regulations in key-metabolic pathways, such as the tricarboxylic acid cycle (TCA) and lysine biosynthesis, as well as the content of intracellular metabolites belonging to processes that could also significantly affect the cell physiology. Finally, SNP analysis in resistant strains revealed no evidence of amino acid alterations in the studied putative bacterial phage receptors, but several SNPs were detected in genes involved in transcriptional regulation. This phenomenon appears to be a phage-specific, fine-tuned metabolic engineering, imposed by the different phage genera the bacteria have interacted with, updating the role of lytic phages in microbial marine ecology.

## 1. Introduction

Bacteriophage and bacteria interactions have been shown to drive coevolution in marine habitats and ecosystems [1]. These interactions also mutually contribute to both their coexistence and their genetic variability [2]. Interest in phage therapy has increased during the last decade as bacterial resistance to antibiotics has risen, although the application of this technology is hampered by the development of phage-resistant bacterial strains [3].

Successful bacteriophage infection is dependent on an efficient entry mechanism, involving many bacterial surface elements. Phages of Gram-positive bacteria are able to recognize and attach to the thick peptidoglycan layer that is composed of teichoic and lipoteichoic acids [4] before lysozyme action [5]. Phage infection of Gram-negative bacteria involves more complex mechanisms. Gram-negative species are thought to possess a very fluidic membrane, able to adapt to various conditions [6]. Surface proteins are constantly in contact with the surrounding environment, and some, such as porins, act as channels for nutrients into the periplasm. These proteins could represent the main conduits for phages [7]. After entering the periplasmic space, phages penetrate the thin layer of peptidoglycan, employing the activity of their tail-lysozyme [8]. It has been shown that tailed bacteriophages, such as T4, are able to generate enough force [9,10] to fuse inner and outer membranes, enabling phage DNA to reach outside the cytoplasm [11,12,13]. Hence, proteins with nutrient-related functions that are localized in the periplasmic space [14] can act as natural passages for DNA to enter the cytoplasm [15].

Aproximately 30% of bacterial genomes are thought to encode membrane proteins or proteins related to transmembrane transport [16]. Apart from porins, there are two major families of transporters that mediate and regulate the uptake of specific substrates in bacteria. The first of these, found exclusively in Gram-negative bacteria, employs a phosphotransferase system (PTS) that phosphorylates the substrate (mainly sugars) before transporting it into the cytoplasm [17]. This mechanism utilizes a phosphate group from phosphoenolpyruvic acid (PEP), producing and transporting to the cytoplasm a 6-phosphoryl sugar, in the case of sugar transport [18]. The second major family is the ABC transporters, which utilize ATP to energize nutrient transport. Members of the ABC transporter family are typically composed of three different subunits. The first is able to bind ATP, the second is located in the periplasmic space where it binds to the substrate, and the third component is a transmembrane protein that translocates the substrate into the cytoplasm [19].

Phage therapy applications require the use of strictly lytic phages that will necessarily infect and kill their bacterial hosts; on the other hand, phage-resistant strains will naturally emerge as a consequence of a bacterial strategy to counteract viral infection [20,21]. Multiple bacterial intracellular molecular defense mechanisms against virulent phages have been described over the past years [22] including the CRISPR/Cas9 system [23], BREX strategy [24], the prokaryotic Argonaute-based interference [25], or DISARM defense system [26]. Finally, prophages and prophage elements have also been shown to increase bacterial survivability and protect bacteria against virulent phages [27]. Although the aforementioned mechanisms can confer bacteriophage resistance, they cannot describe any resistance phenomena taking place in the bacterial membrane or in any bacterial species that do not harbor them.

In this study, we used *Vibrio alginolyticus*, in order to gain insight into the molecular and biochemical mechanisms of resistance development, during interaction with lytic phages, using both targeted transcriptomic and metabolomic approaches. *V. alginolyticus* is an opportunistic Gram-negative rod-shaped pathogen of various aquatic animals that has been occasionally reported in human infections [28], from which various bacteriophages have been isolated over the years. Our results indicate that the development of phage-resistant *V. alginolyticus* strains is accompanied by changes in the levels of nutrient transport-related transcripts, linked to an extensive reprogramming of several key metabolic processes within the host cell. We demonstrate that lytic bacteriophages interacting with *V. alginolyticus* could play a pivotal role in cell physiology. More specifically, resistant cells regulate the expression of proteins involved in nutrient uptake that are found in abundance on the bacterial membranes of Gram-negative species. This regulation seems to mainly affect nutrient uptake and availability, shifting the intracellular biochemical processes accordingly and, in a phage-dependent manner, highlighting a newly reported adaptation strategy. In addition, whole genome sequencing of the resistant strains revealed several SNPs in genes involved in bacterial transcriptional regulation. Interestingly, these complex transcriptional and metabolic interactions of lytic bacteriophages in Gram-negative species remain elusive, although various results on phage-induced phenotypes have been reported.

## 2. Materials and Methods

### 2.1. Vibrio alginolyticus Strain V1

*V. alginolyticus* strain V1, including its genome, has been thoroughly described in the past [29]. This strain was isolated from diseased gilthead seabream (*Sparus aurata*) in Crete, Greece. Culture and growth conditions included Luria–Bertani (LB) broth supplemented with MgSO_4_ 1 mM and CaCl_2_ 1 mM; the LB broth contained Tryptone (10 g/L), NaCl (10 gL), and Yeast Extract (5 g/L). All culture conditions (liquid and solid) included an incubation temperature of 25 °C and vigorous shaking. Long periods of preservation of the bacterial strain included cells in the exponential phase in glycerol stocks (20%) and −80 °C preservation temperature.

### 2.2. Bacteriophages Aphrodite1, phiSt2, and Ares1

Three lytic bacteriophages were used in the current study to generate phage-resistant strains of *V. alginolyticus*, (Table 1). Two of them belong to the *Myoviridae* family and one to *Siphoviridae*. phiSt2 was isolated in Crete, Greece [30], and belongs to the *Schizotequatrovirus* phage genus according to the International Committee on Taxonomy of Viruses (ICTV) as detailed described in [31]. Aphrodite1 belongs to the *Aphroditevirus* genus according to ICTV and was isolated from the aquatic area of North Euboea Greece. Ares1, a typical *Siphoviridae* family phage (genus still unclassified) was isolated from South Attika, Greece. All bacteriophages formed clear lytic plaques following the standard double-layer agar (0.5%) method [32]. For Transmission Electron Microscopy (TEM), virions were prepared on collodium copper grids, negatively stained with a 2% uranyl acetate, and examined using an electron microscope (JEOL JEM-1005) at 80 kV.

### 2.3. Vibrio alginolyticus Phage-Resistant Strains

Phage-resistant bacterial strains were generated after overnight exposure to each phage at a multiplicity of infection (MOI) of 100. In detail, three independent cultures of *V. alginolyticus* grown in LB medium were inoculated during their exponential phase (OD_600_: ~0.2) with each phage at MOI 100. The cultures were incubated overnight at 25 °C. Dilutions of the phage lysate were conducted after overnight culture and subsequently cultured on LB medium with 1.5% agar. After overnight incubation at 25 °C, single colonies were isolated. All selected colonies were re-cultured in LB liquid broth and infected by their corresponding phages by the spot test assay. High titer (>10^9^) phage stocks were used to confirm resistance. To test if bacteriophages could remain inside the cells, we used colony PCR with bacteriophage primers (Supplemental Table S2) for at least three independent resistant strains for each phage. We were not able to detect any amplified DNA region (data not shown). Phage-resistant colonies were re-plated, and three independent colonies that served as biological replicates from each bacterial host were stored with 20% glycerol at −80 °C for further analysis.

### 2.4. Growth Kinetics, Phage Biological Characteristics, and Phage Adsorption Dynamics

The growth curves of three phage-resistant strains and three control (wild type) strains were generated by culturing them at 25 °C in liquid LB medium. During the exponential phase, they were transferred to a 200 μL volume of a 96-well plate (TrueLine) using three technical replicates for each resistant strain. The OD_600_ was measured every 30 min by using a plate reader at 25 °C with shaking (Infinite M200 PRO, TECAN). 

Biological characteristics of the novel bacteriophages Aphrodite1 and Ares1 were determined by using the protocol described in [30]. Briefly, bacteriophages were added to the host bacterial culture in the early exponential phase in triplicate, using an MOI of 0.01 following incubation at 25 °C for 15 min. Cells were then centrifuged, and the supernatant containing unattached phages was discarded. Virocells were suspended in Luria–Bertani broth supplemented with MgSO_4_ and CaCl_2_ medium and transferred to 20 mL fresh liquid medium. Petri dishes containing a bacterial lawn of the host were spotted every 10 min for a total duration of 90 min. Phage plaques were formed after overnight incubation at 25 °C.

In order to monitor the adsorption dynamics of phage-resistant strains, we co-cultured each phage with the corresponding resistant strain in liquid LB with an initial phage titer of 10^4^ PFU mL^−1^. Bacteriophages were inoculated in the liquid culture during the exponential phase (OD_600_ = ~0.2) of the bacteria, and sample time points were 20 min, 40 min, 60 min, and 960 min post inoculation for every resistant and susceptible strain. Sample time points were established in order to monitor phage adsorption before the completion of a replication cycle for each bacteriophage with the addition of a long-term sample time point. For every time point, an aliquot of 100 μL was used for titration by spot assay.

### 2.5. Phage DNA Extraction and Sequencing

Bacteriophage DNA extraction and sequencing for Aphrodite1 and Ares1 were done as previously described [31]. Briefly, liquid bacterial cultures of *V. alginolyticus* strain V1 at the exponential phase of growth (grown in LB medium), were infected separately by bacteriophages Aphrodite1 and Ares1 with an MOI of 10. Both cultures were incubated overnight at 25 °C with reciprocal shaking. The following day, the cultures were centrifuged, and their supernatants were filtered (0.22 µm), titered, and stored at 4 °C. Starting with an optimal titer of 10^10^ PFU mL^−1^, phages were then concentrated using standard poly-ethylene glycol/NaCl precipitation. Concentrated phages were treated with Turbo DNAse (Ambion, TX, USA) and RNAse to remove the host’s and contaminant nucleic acids before phage DNA was extracted from the capsids. Genomic DNA was purified using a QIAamp DNA Blood Mini Kit (QIAGEN, Hilden, Germany) following the manufacturer’s instructions with the addition of absolute ethanol before the first column wash to ensure the efficient binding of the gDNA to the silica membrane. DNA integrity and purification were monitored by using NanoDrop (ThermοFisher Scientific, Waltham, MA, USA) measurements and by 0.7% agarose gel before library construction.

At least 5 μg of high-purity phage genomic DNA was used to ensure quality with a BioAnalyzer (BioRad, CA, USA) and for the construction of a paired-end library using a Nextera Library Construction Kit (Illumina, San Diego, CA, USA) with an insert size of 800 bp followed by Illumina sequencing using an Illumina Hi Seq 2000 (Illumina, San Diego, CA, USA) sequencer. Sequencing was conducted at the Beijing Genomic Institute (Shenzhen, Guangdong, China) according to the manufacturer’s protocol.

### 2.6. Bacterial DNA Extraction and Sequencing

Bacterial DNA from the obtained resistant strains for each bacteriophage was extracted using a sodium dodecyl sulfate (SDS)-based protocol including protease treatment. Briefly, cells were harvested during the exponential phase, washed, and incubated at 56 °C for 2 h with the DNA extraction buffer (2% SDS, 200 μg/mL proteinase k, Tris 7.2 pH 10 mM, EDTA 0.1 mM and beta-mercaptoethanol 0.1%). RNAse treatment followed at 37 °C, and DNA was then purified by using DNA purification silica columns with a commercially available kit (Macherey-Nagel, Duren, Germany) according to the manufacturer’s protocol. DNA quality and RNA contamination were assessed by using a 0.7% agarose gel and Nanodrop spectrophotometer (ThermoFisher Scientific, Waltham, MA, USA). 

At least 5 μg of high-purity bacterial DNA for each resistant strain was used to ensure quality with a BioAnalyzer (BioRad, CA, USA) and for the construction of a paired-end library using a Nextera Library Construction Kit (Illumina, San Diego, CA, USA) with an insert size of 350 bp, using a paired-end 300 genomic library followed by Illumina sequencing. In order to detect possible Single Nucleotide Polymorphisms (SNPs), we used a genome sequence depth of at least 50x.

### 2.7. Bacteria and Bacteriophage Genome Analyses

After the phage DNA sequencing, possible contaminated reads, primers, N-terminus, and 3’-, 5’- low quality reads were trimmed off with an error rate threshold of 0.05 from the raw data. *De novo* assembly was conducted using Velvet software (version 1.2.10) [33] and the Geneious platform (R10 version; Biomatters Ltd., Auckland, New Zealand; [34]). Assembly resulted in single contigs on both occasions.

Annotations were performed with the gene caller software Glimmer (version 3.0, [35]), the Rapid Annotation Subsystem Technology (R.A.S.T.; [36,37]), and the B2Go (BioBam, Valencia, Spain) platform against a non-reductant protein database and UniProt database with an E-value threshold of ≤10^−6^. The results from all annotators were filtered, evaluated, and combined. Putative protein products derived from the annotators were verified with the Kyoto Encyclopedia of Genes and Genomes database (K.E.G.G.; [38,39]). Bacteriophage genomic analysis presented in Supplemented Data 2 was conducted with the MAUVE (version 2.4.0) whole genome aligner [40]. Whole genome similarity was carried out using the LastZ algorithm (default parameters; version 1.02.00). 

Bacterial putative CRISPR/Cas protein products were analyzed with CRISPRs Finder software [41] and verified by using HMMCAS software with default parameters [42].

For the analysis of the resistant bacterial genomes, PE 300 reads for each library were filtered (removed reads containing adapters, Ns > 10%, and low quality (Qscore ≤ 5)) and merged accordingly. Duplicate reads were removed with Dedupe software (version 37.25) based on a k-mer value of 31 before assembly with the Geneious assembler (R10 version; Biomatters Ltd., Auckland, New Zealand; [34]), with a 5-time iteration parameter, and reads were compared to the reference genome of *Vibrio alginolyticus* strain V1 in the present study [29]. Quality coverage was assessed with a standard deviation from the mean equal to 2, before analyzing each contig for SNPs. Variant motifs (single nucleotides, insertions, and deletions) were searched with a minimum variant frequency of 0.25, a maximum variant *p*-value of 10^−6^, and a minimum strand-bias *p*-value of 10^−5^.

### 2.8. Gene Expression Analysis

Gene expression was studied in wild type (phage-sensitive strains) and phage-resistant bacterial colonies after retrieval from −80 °C stocks and overnight growth at 25 °C under vigorous shaking in LB medium. RNA extraction was performed using the Nucleozol^TM^ (Macherey-Nagel, Düren, Germany) reagent according to the manufacturer’s protocol. After harvesting, the cells were washed with 150 mM NaCl and then homogenized in 500 μL Nucleozol^TM^ followed by vortexing and multiple centrifugations at 6200× *g* for better homogenization of the cells. Then, ddH_2_O (200 μL) was added, and the samples were incubated at room temperature for 15 min before centrifugation at 12,000× *g* for 15 min. Isopropanol (700 μL) was added in the aqueous phase in order to precipitate RNA after incubation for 10 min at room temperature and centrifugation for 15 min at 13,000× *g*. The pellet was washed twice with 75% ethanol before dissolution in ddH_2_O. RNA integrity was checked by agarose gel electrophoresis.

RNA (6 μg per sample) was treated with TURBO DNAse (Ambion, TX, USA) according to the manufacturer’s protocol to remove DNA contaminants and then tested with PCR to verify absence of bacterial DNA. After the DNase treatment, a 70% yield was retrieved, and the integrity of the remaining RNA was checked again by using a 2% agarose gel. RNA quality was estimated using a Nanodrop (ThermoFisher Scientific, Waltham, MA, USA). Approximately 800 ng of RNA was used per cDNA synthesis step, employing the PrimeScript RT reagent (Takara, Dalian, China) synthesis kit, which utilizes the reverse transcription efficiency of the Primescript^TM^ enzyme. Specifically, reverse-strand cDNA synthesis was performed in a 10 μL total reaction volume containing 800 ng total RNA, 0.5 μL PrimeScript™ RT Enzyme mix, 2 μL random hexamers (100 μM), PrimeScript Buffer for Real-Time, and the addition of RNase-free ddH_2_O up to 10 μL. A master mix of the reagents was prepared before the addition of RNA for each sample. The reverse transcription took place by utilizing the following regime: 37 °C for 15 min, 85 °C for 7 s, and 4 °C for 5 min.

Primer pairs for cDNA amplification (Supplemental Table S2) were designed using the sequenced genome of the *V. alginolyticus* strain V1 [29] utilizing Geneious software (R10 version; Biomatters Ltd., Auckland, New Zealand; [34]) and were in silico tested against bacterial and bacteriophage genomic DNA to ensure that a single amplicon of 70 bps would be amplified. The sequences of studied genes were also compared to the reference strain 12G01 (NZ_CP042449, NZ_CP042450) to validate their function. Quantitative real-time PCR was performed on a StepOnePlus™ Real-Time PCR System (Applied Biosystems, Foster City, CA, USA) using SYBR Select Master Mix (Applied Biosystems, Austin, TX, USA) up to 10 μL per reaction. The gene-specific primers were used at a final concentration of 0.2 μΜ each. In total, 1 μL of cDNA template was used per reaction. PCR cycling started with initial polymerase activation at 95 °C for 10 min, followed by 40 cycles at 95 °C for 15 s and 60 °C for 1 min. The formation of primer dimers and each primer’s specificity were monitored by dissociation curve analysis and agarose gel electrophoresis. Melt curves for each reaction were also monitored to ensure single product amplification. Additionally, dense agarose gel (3%) was used after each reaction to monitor the correct product size (70 bps). The expression levels of *V. alginolyticus* gyrase A (*gyrA*) and the HSP70 protein (*dnaK*) were used as housekeeping genes (HK) to normalize cDNA templates and compute the relative transcript levels for each gene, which were calculated as (1 + E)^−ΔCt^. ΔCt was calculated as (Ct^X^–Ct^H^), whereas Ct^X^ corresponds to the Ct of the targeted gene, and Ct^H^ is the geometrical mean of the two HK genes’ Cts. PCR efficiency (E) for each amplicon (Supplemental Table S1) was calculated by applying the linear regression method to the log (fluorescence) per cycle number data, using LinRegPCR software [43].

### 2.9. Metabolite Extraction and Intracellular Metabolite Analysis

Five resistant *V. alginolyticus* biological replicates for each phage and four wild-type strains were grown in LB medium and harvested during their exponential phase, washed with 150 mM NaCl, and collected by centrifugation. Metabolites were extracted by using the cold methanol extraction method for Gram-negative bacteria according to [44]. At least 12 mg of bacterial dry biomass was extracted using 400 μL of methanol, and 5 μL of 1 mg/mL ribitol was added as an internal standard. The samples were then incubated at 70 °C for 15 min before the addition of 200 μL of chloroform to each sample in order to separate the polar from the non-polar metabolites. The samples were incubated at 37 °C with continuous agitation for 5 min. Finally, 400 μL of ddH_2_O was added, and the samples were centrifuged for 5 min at 16,200× *g*. Then, 100 μL from the aqueous phase was evaporated under N_2_ gas to prevent oxidation of metabolites. The derivatization protocol included re-suspension of every sample in 25 μL of methoxlyamine-HCl (20 mg mL^−1^ in pyridine) and incubation for 90 min at 30 °C with continuous gentle shaking followed by the addition of 50 μL of N-methyl-N-(trimethylsilyl)-trifluoroacetamide (MSTFA). Samples were then incubated for 30 min at 37 °C under continuous agitation. Finally, 10 μL of an n-alkane mix was added for the determination of retention indexes (RIs) using AMDIS software (version 2.72) with an RI window of 50*0.01.

GC/MS analysis was carried out on an Agilent 6890 GC coupled to a 5973i MSD (Agilent Technologies, Palo Alto, CA, USA). The separation was achieved on a DB-5MS fused capillary column (60 m long, 0.25 mm internal diameter, film thickness 0.25 μm, J&W Scientific). Helium was used as carrier gas at a flow rate of 1 mL min^−1^. The split mode of injection was used, and the injection volume was 1 μL. The GC inlet temperature was held at 280 °C. The oven temperature program was as follows: initial temperature 80 °C (held for 2 min), followed by an increase to 315 °C (held for 12 min) at a rate of 5 °C min^−1^. The total run time was 60 min. The MS source was held at 250 °C and the quadruple at 150 °C, scanned from 50–650 m/z. The GC/MS results were analyzed with AMDIS (version 2.72, minimum factor: 60, multiple identifications per component) software utilizing both Golm [45] and Fiehn [46] metabolite libraries. Final values are represented as the ratio between the area of the target metabolite divided by the area of ribitol (m/z 319), which was used as a reference metabolite and reported relative to the dry weight of each sample. 

### 2.10. Statistical Analysis

Statistical analysis, including one-way ANOVA with adjusted Student’s *t*-test as post-hoc analysis for multiple tests, and plot generation took place using SigmaPlot software (Systat Software Inc., San Jose, CA, USA; version 12.0). Partial least square discriminant analysis was conducted with the Multibase version of the 2015 add-on for Microsoft Excel and visualized with Sigmaplot. Heat maps were created by using Microsoft Excel.

All figure legends include details regarding statistical significance, number of biological replications used, the statistical test used, significance threshold, and dispersion and precision measures.

### 2.11. Accession Numbers

The draft genomes of the newly introduced bacteriophages Aphrodite1 and Ares1 are available in the GenBank public DNA repository under accession numbers MG720308 and MG720309, respectively. Additionally, the bacteriophage phiSt2 genome is available in the GenBank public DNA repository under the accession number MG720309. The *V. alginolyticus* strain V1 genome is also available in the GenBank public DNA repository under the accession number LCUM00000000. The genomes of the bacteriophage-resistant strains have been deposited n GenBank under the accession number JAGFOO000000000 for *Vibrio alginolyticus* strain VaAphrodite1_A, JAGFON000000000 for *Vibrio alginolyticus* strain VaAphrodite1_B, JAGFOM000000000 for *Vibrio alginolyticus* strain VaAphrodite1_C, JAGFOJ000000000 *Vibrio alginolyticus* strain VaphiSt2_A, JAGFOI000000000 for *Vibrio alginolyticus* strain VaphiSt2_B, JAGFOL000000000 for *Vibrio alginolyticus* strain VaAres1_A, JAGGED000000000 for VaAres1_B, and JAGFOK000000000 for *Vibrio alginolyticus* strain VaAres1_C.

## 3. Results

### 3.1. Description of Novel Bacteriophage Isolates

In this work, we report two newly isolated bacteriophages from coastal areas in Greece. Aphrodite1 was isolated from northern Euboea. Transmission electron microscopy showed a typical myovirus morphotype (Supplemental Data 1), different from phiSt2. Genome assembly resulted in a single contig of 237,722 bps, and according to ICTV, was assigned to the *Aphroditevirus* genus. This phage exhibits whole genome nucleotide similarity with the *Vibrio* phages pTD1 (AP017972) (76.8%) and VP4B (KC131130) (62%), which belong to the genus *Tidunavirus* according to ICTV. No integrase gene was detected in the genome, suggesting that it is a phage with a likely explicit lytic lifestyle. Aphrodite1 has an 80-min latent period.

Ares1 was isolated from South Attika. Transmission electron microscopy revealed a virion with a long non-contractile tail, a typical siphovirus morphotype (Supplemental Data 2). Its genome was assembled to a single contig of 80,500 bps. No ORF homologous to integrase was identified. Whole genome nucleotide similarity (82%) with bacteriophage VHS1 *Vibrio* phage (unclassified genus) indicates that the lifestyle of Ares1 remains an open question [47]. Ares1 has a 30-min latent period.

### 3.2. Cross Infection and Growth Curve of Phage-Resistant Strains

Phage-resistant colonies were obtained as described in the respective materials and methods section and annotated based upon the phage to which they developed resistance, i.e., VaAphrodite1, VaphiSt2, and VaAres1 (Table 2). Resistance was maintained throughout all the experiments for all strains. In addition, cross-infection with all the phages presented in Table 2 showed that bacteriophages remained ineffective against their corresponding phage-resistant strain and effective against all the remaining strains, apart from Ares1, which remained ineffective against VaAphrodite1.

**Figure 1:**
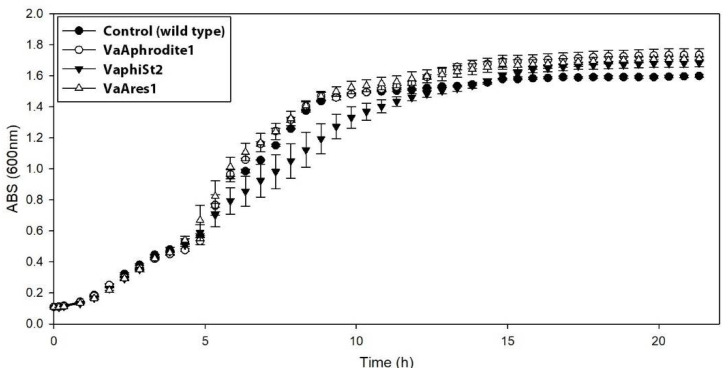
Growth kinetics of phage-resistant and phage-susceptible strains. Growth (average, ±SE) of the Control (phage-susceptible strains; black circle; *n* = 3), VaAphrodite1 strains (phage strains resistant to bacteriophage Aphrodite1; white circle; *n* = 9), VaphiSt2 strains (phage strains resistant to bacteriophage phiSt2; black triangle; *n* = 9), and VaAres1 strains (phage strains resistant to bacteriophage Ares1; white triangle; *n* = 9).

Furthermore, the growth kinetics of the phage-resistant strains were compared to those of the phage-susceptible strains (Figure 1). The resistant strains VaAphrodite1 and VaAres1 did not show any apparent changes in their growth rate. In contrast, resistant strains VaphiSt2 displayed a diverse growth pattern. Two out of the three resistant strains exhibited reduced growth compared to the wild type, while the growth rate of the third remained unaffected. Specifically, during their exponential phase and 5 h after incubation, the two independent colonies exhibited 36% and 12.5% reduced OD_600_ compared to the wild-type strains, resulting in a differentiation in their growth rate (Supplemental Figure S1). 

### 3.3. Study of Anti-Phage Defense Systems of Vibrio alginolyticius

**Figure 2:**
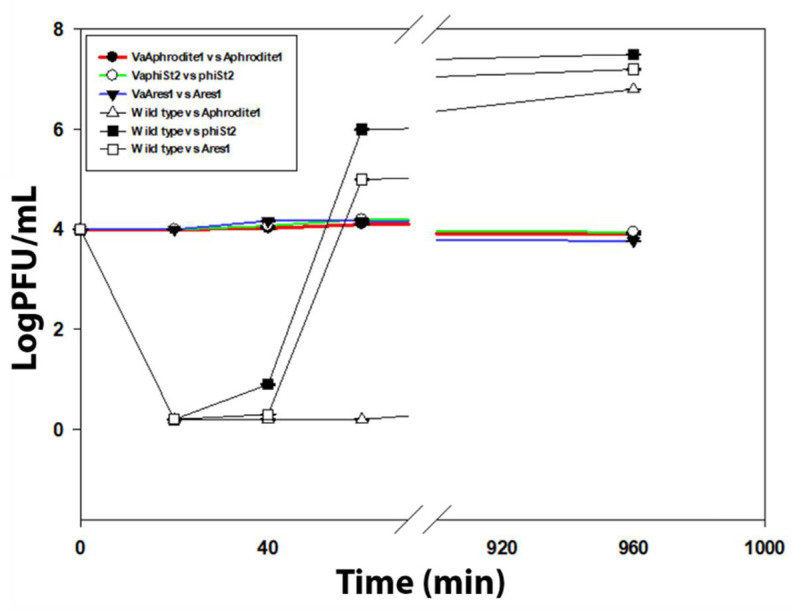
Adsorption assay for bacteriophages Aphrodite1, phiSt2, and Ares1 against their corresponding resistant strains. Initial titer was 10^4^, and cultures upon infection were in their exponential phase (±SE; *n* = 3).

To uncover mechanisms of acquired resistance, two different strategies were followed: (i) a genome annotation analysis of *V. alginolyticus* strain V1, to pinpoint known molecular mechanisms that could confer acquired resistance, and (ii) measurement of phage adsorption on resistant strains. The genome of *V. alginolyticus* strain V1 was in silico analyzed in order to identify potential CRISPR/Cas systems [23], which could confer an adaptive molecular mechanism. We identified three putative Cas homologs, with corresponding protein ids KLI72196, KLI72197, KLI72198 in the genome of *V. alginolyticus* strain V1, via comparative sequence analysis. However, our initial annotation could not be verified using the CRISPRsFinder software. Only a small genomic region in Contig 18 was identified as a putative Crispr/Cas associated product, using CRISPRsFinder, and this was not supported by HMMCAS software, leading us to conclude that this *Vibrio* stain does not possess a functional CRISPR/Cas system. Harbored prophages in the bacterial genomes have also been shown to contribute to phage resistance against lytic bacteriophages by a molecular mechanism called superinfection immunity [48]. Genome annotation did not reveal the presence of any whole prophage harbored in the genome of strain V1, and only isolated phage-related proteins were located, for example, genome ids KLI74070, KLI74028, KLI74283, KLI74284, KLI74285, and KLI74292. Finally, genome alignment of known and well-characterized BREX and DISARM genome cassettes in the genome of *V. alginolyticus* V1 did not reveal any positive results, which could also potentially confer resistance. On the other hand, phage adsorption assays revealed no adsorption process by resistant strains, even after 16 h of co-culture (Figure 2), when using phages of the original stock. These results indicate a radical modification of proteins that consist of the bacterial membrane, possibly responsible for the impaired adsorption and infection.

### 3.4. Transcript Profiling of Bacterial Genes Involved in the Phage Infection Process

**Figure 3:**
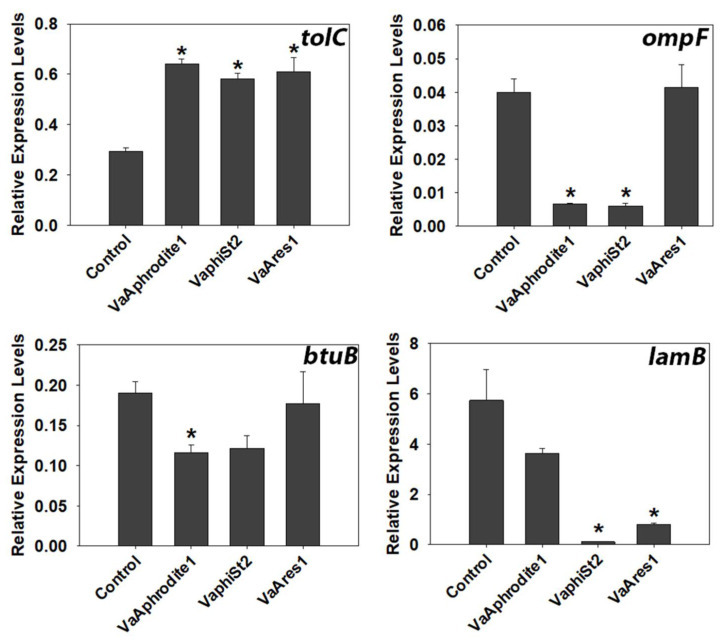
Relative expression levels of genes encoding for outer membrane and transmembrane proteins. Bars represent means ± SE of independent biological repeats (*n* = 3). Asterisks * indicate significantly different values based on Student’s *t*-test. Statistical significance was postulated for *p* ≤ 0.05.

Bacteriophage adsorption is the initial step for a successful infection process, as was demonstrated in detail for λ bacteriophage [49]. In the case of Aphrodite1, phiSt2, and Ares1, the exact bacterial receptors mediating infection remain to be established. However, in order to test whether the observed phage resistance is accompanied by changes in the expression of selected genes coding for bacterial outer and inner membrane proteins, we used an RT-qPCR-targeted transcriptomic approach. Targeted outer membrane proteins included proteins that have been well characterized as phage receptors, such as vitamin porin protein BtuB [50], maltose porin LamB [49], osmotic adjustment protein OmpF [51], and TolC ABC-like transmembrane protein [52] (Figure 3, Supplemental Table S1). Interestingly, *ompF* showed a 10-fold downregulation in the resistant strains for bacteriophages Aphrodite1 and phiSt2. Additionally, the *lamB* relative transcript levels were drastically 57- and 7-fold downregulated in VaphiSt2 and VaAres1 resistant strains, respectively. Finally, the *btuB* relative transcript levels were significantly downregulated in VaAphrodite1 strains. In contrast, we were able to detect a 2-fold statistically significant accumulation of *tolC* gene transcripts in all the resistant bacterial strains included in this work.

Additionally, we selected several membrane and transmembrane transport systems in order to study their gene expression patterns in wild-type and phage-resistant *Vibrio* strains for all phages used in the study (Supplemental Table S1). Interestingly, the results revealed mostly highly depleted relative transcript levels for all three bacteriophages. These included mainly ABC and PTS transporters, which consist of several subunits, among them permeases, which are responsible for delivering the nutrients from the periplasmic space into the cytoplasm (Table 3 and Table 4). Transcript levels for the permease domains *livH*, *livB,* and *azlC*, which are responsible for the transport of aliphatic amino acids with side chains, such as leucine, valine, and isoleucine, were studied. Our results revealed a statistically significant downregulation of the relative transcript levels for both *livH* and *livB* for the resistant strains. For the two *azlC* genes, in the case of VaphiSt2 and VaAphrodite1, we observed a 4-fold and 0.7-fold reduction in *Azlc*1 transcript levels, respectively, while *azlC* 2 transcripts were 2-fold downregulated in VaAres1. As for the cysteine transporter-encoding gene *tcyP*, we observed a significant downregulation for all the resistant strains. Transcripts for *metL*, *metN,* and *metQ* genes, encoding the three subunits of the methionine ABC transporter, showed significant downregulation in the resistant strain VaAphoridite1 in comparison to phage-susceptible strains. In addition, VaphiSt2 strains showed a significant downregulation for *metl* and *metN* subunit transcripts although transcript downregulation was also observed in *metQ* without being statistically significant. In the case of Arginine transporters encoded by the genes *artP* and *artL*, both showed a 5-fold downregulation in the case of VaAphrodite1. They also presented a 2-fold downregulation in the case of VaAres1, with *artL* being statistically significant. No alterations were observed in the case of VaphiSt2. The lysine export system in bacteria is represented by a protein encoded by the gene *lysE*, showing drastically reduced transcript levels (76-fold downregulation) only in the case of VaAphrodite1 resistant strains, but transcript levels remained unaffected for the other two phage-resistant strains. In addition, one of the *rhtB* genes coding for an LysE-like transport system, responsible for the export of Homoserine and Threonine, showed lower transcripts in the VaphiSt2-resistant strains. Finally, our results revealed that the *hisP* gene transcripts, encoding an ATP-binding protein dedicated for energy delivery in transport systems of Histidine, Lysine, Arginine, and Ornithine, were significantly downregulated in the resistant strains VaAphrodite1 and VaphiSt2.

In addition, we studied the transcription of selected genes coding for sugar and polyol transport systems. Genome wide annotation [29] of *V. alginolyticus* showed that the glucose PTS transport system is comprised of two permease isoforms encoded by *ptsG* 1 and *ptsG* 2 genes, and one subunit responsible for delivering ATP to the transport system encoded by *crr*. Interestingly, our results revealed statistically significant downregulation of *ptsG* 2 and *crr* in all resistant strains. By contrast, *ptsG* 1 expression was upregulated 4-fold in the resistant strain VaAphrodite1. The trehalose transport system is encoded partially by the gene *treB*, a subunit responsible for encapsulation and transport from the periplasmic space to the cytoplasm. Our results revealed a significant downregulation only for the resistant strains of bacteriophage Aphrodite1. A similar pattern was observed for *fruA* transcripts, encoding for the permease protein responsible for transporting fructose. In addition, *mtlA* transcripts, coding for a transmembrane complex responsible for transportation and phosphorylation of mannitol, showed a 10-fold downregulation only in VaphiSt2 strains. As part of the ribose transport system, *rbsA*, encoding for the ATP-binding subunit, was downregulated in all three phage-resistant strains. Furthermore, the *cellB* gene, encoding for permease responsible for translocating cellulose, was found to be significantly downregulated in VaAphrodite1 and VaphiSt2. Finally, *ptsN* gene transcripts, coding for a nitrogen transport protein, exhibited significant downregulation in both VaAphrodite1- and VaphiSt2-resistant strains, while *ptsH*, coding for the phosphorous carrier protein Hpr, showed lower mRNA levels only for VaphiSt2-resistant strains. The results showed that amino acids-related proteins were subjected to a more significant transcript reprogramming across all phage-resistant strains than the sugar-related proteins. 

### 3.5. Transcript Profiling of Genes Coding for Key Metabolic Enzymes

The observed changes in the accumulation patterns of many bacterial transcripts involved in nutrient transport prompted us to study the cellular biochemical processes involved in the metabolism of the respective compounds. For this purpose, an expression study of 21 selected genes involved in major bacterial metabolic pathways was conducted for our resistant *Vibrio* strains (Supplemental Table S1, Table 4 and Table 5). Three major metabolic pathways were studied, including alanine metabolism, lysine metabolism, and the TCA cycle and its anaplerotic reactions. Interestingly, transcript accumulation for *ald* encoding alanine dehydrogenase was significantly higher in resistant strains VaAphrodite1 and VaphiSt2 compared to wild-type bacteria. The amino acid ligase encoded by *murE* shown a 2-fold transcript accumulation only for VaphiSt2-resistant strains. The results for one of the lysine decarboxylase genes, *lysA* 2, showed a statistically significant upregulation for all the resistant strains, with VaAres1 exhibiting a 10-fold transcript accumulation. Additionally, alanine glycosylate (*agxT*) and aspartate 4-decarboxylase (*panD*) did not exhibit any statistically significant differences among the resistant strains. The study of genes involved in the TCA cycle and its anaplerotic reactions revealed substantial differences between the resistant and susceptible strains. Specifically, relative transcript levels of phosphoenolpyruvate carboxykinase (*pckA*) showed a 2-fold statistically significant accumulation for VaAphrodite1-resistant strains and a drastic 40-fold significant downregulation for VaphiSt2-resistant strains. Fumaric acid reductase (*frd*) also showed a similar pattern of a 7-fold statistically significant upregulation for VaAphrodite1 strains and a 17.5- fold statistically significant upregulation for VaphiSt2, while VaAres1 appeared to be significantly downregulated. Malic acid decarboxylase (*mdh*) showed significant downregulation for both resistant strains VaAphrodite1 and VaphiSt2. PEP carboxylase (*ppc*) transcripts appeared to be downregulated in all of the respective resistant strains without being statistically significant. Citrate synthase (*gltA*) was statistically significantly downregulated in resistant strains only for VaphiSt2. Two pyruvate kinases were tested in this study (*pykA* and *pykF*). Only *pykA* appeared to be downregulated significantly (12-fold) for resistant strain VaphiSt2, while it was upregulated for VaAres1. The results of the studied metabolic pathways revealed a strong metabolic adjustment for bacteriophage phiSt2, a medium adjustment for bacteriophage Aphrodite1, and a minor adjustment for bacteriophage Ares1. 

### 3.6. Metabolomic Profiling of the Resistant Strains

**Figure 4:**
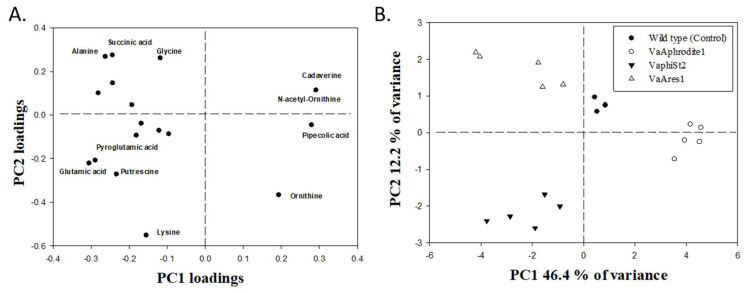
Partial Least Squares Discriminant Analysis (PLS-DA) of metabolomics results. and representation of component 1 (46.4%) and component 2 (12.2%) (**A**) of the identified metabolites discussed in the results and discussion section comparing the wild type (Control, phage-susceptible strains; *n* = 4), VaAphrodite1 strains (phage strains resistant to bacteriophage Aphrodite1; *n* = 5), VaphiSt2 strains (phage strains resistant to bacteriophage phiSt2; *n* = 5), and VaAres1 strains (phage strains resistant to bacteriophage Ares1; *n* = 5). (**B**) Loadings of individually identified components showing the highest variability under the studied experimental conditions.

In order to test whether the transcriptional reprogramming observed in several transport systems and related metabolic processes in our *Vibrio*-resistant strains could also lead to changes in the cell metabolome, we employed a GC-MS-based metabolomic approach. Partial Least Squares Discriminant Analysis (PLS-DA), which clusters the samples into groups, revealed strong differentiation in metabolite contents after interaction with different bacteriophages, hinting at phage-specific metabolome reprogramming. The first principal component (PC) accounted for 46.4% of the total variance in the means of the relative metabolite content and separated resistant strains VaArphrodite1 and VaAres1 from the wild type (control). The second PC component accounted for 12.2% of the total variance and discriminated resistant strains VaphiSt2 (Figure 4B, Table 6).

Several amino acids as well as some organic acids were positively identified. Among them, proline and aspartic acid were not detected in VaAphrodite1 strains. Ornithine showed statistically significant accumulation in VaAphrodite1 strains and was not detected in VaAres1-resistant strains. Glutamic acid showed a statistically significant reduction of its relative content in VaAphrodite1 strains. By contrast, significant accumulation in the resistant strain VaphiSt2 was observed. Similar accumulation in the resistant strain VaphiSt2 appeared for pyroglutamic acid. Cadaverine showed accumulation in each of the VaAphrodite1 strains, but a significant reduction in its relative content levels in VaphiSt2. Lysine showed 10-fold higher accumulation only in VaphiSt2-resistant strains, while the leucine content decreased in VaphiSt2 and VaAres1. Norleucine, alanine, and glycine also exhibited accumulation only in resistant strain VaAres1. Interestingly, the polyamine putrescine exhibited a 2-fold higher content only in VaphiSt2. These results are indicative of a strong amino acid metabolism reprogramming accompanying the development of resistance for the lytic bacteriophages. Correlations between alterations of amino acid contents and transcript regulation of genes due to acquired resistance were also observed. Finally, in the group of organic acids, succinic acid showed a 3-fold statistically significant increase in its respective relative content levels only in resistant strain VaAres1, and pipecolic acid was detected only in resistant strain VaAphrodite1.

### 3.7. Comparative Genomic Analysis of Resistant Vibrio Strains

In an attempt to gain a deeper insight into the molecular mechanisms of the observed impaired phage adsorption and transcriptional changes, we employed whole genome sequencing and comparative genome analysis of the target resistant *Vibrio* strains. Our main goals were (i) to investigate if the reported phage receptors studied here harbored amino acid substitutions that could result in defective phage adsorption and/or (ii) if the overall underlying genetic changes could provide clues for the observed transcriptional reprogramming. Thus, we sequenced three resistant strains for bacteriophages Aphrodite1 and Ares1 and two resistant strains for bacteriophage phiSt2. Illumina sequencing produced 7307072, 6,051,830, and 8,298,832 clean reads for VaAphrodite1 strains, 6,868,528 and 8,946,130 of clean reads for VaphiSt2 strains, and 6282184, 6,509,178, and 8,535,362 clean reads for VaAres1 strains. The map used for the reference assembly process mapped 6334151, 5,786,703, and 8,285,229 reads for VaAphrodite1 resistant strains, 4,031,927 and 8,930,089 for resistant strain VaphiSt2 and, 8285229, 4,341,793, and 6,485,229 for resistant strain VaAres1. These 150 bps reads corresponded to at least a 110X genome coverage for each assembly. 

**Figure 5:**
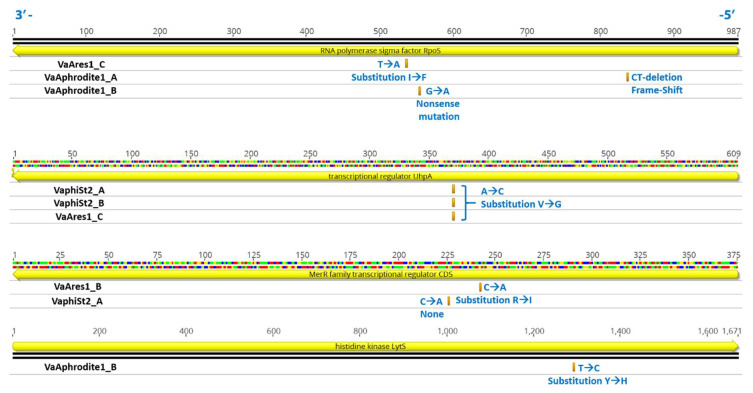
Schematic representation of nucleotide sequences of four CDSs and their orientation, involved in transcriptional regulation of *V. alginolyticus*, and their SNPs as presented in the text. Yellow represents CDS; orange, SNP positions; and blue, information of SNPs and protein effects in resistant bacteria VaAphoridte1 (A, B), VaphiSt2 (A, B), and VaAres1 (B, C). Orientation of the genome is also shown with blue numbers.

Interestingly, we could not detect any polymorphisms, deletions, or insertions in the resistant strains regarding the candidate phage receptors included in this study, or any of the reported genes that are related to membrane or transmembrane nutrient transport (Supplemental Table S3) or their neighboring genomic areas. Nevertheless, several polymorphisms were detected in numerous regions (Supplemental Table S3). Overall, resistant strain VaAphrodite1 harbored 471, 477, and 437 SNPs, VaphiSt2 harbored 525 and 451, and VaAres1 strains 493, 496, and 489 SNPs for each different resistant colony that was sequenced. In total, only two genomic deletions were observed, including the resistant strain for bacteriophage phiSt2, harboring a genomic deletion of 9171 bases in Contig 2 of the reference strain, between bases 223,684 and 232855. This region harbors 14 CDSs, including 10 hypothetical proteins (KLI74020, KLI74022, KLI74024, KLI74025, KLI74026, KLI74027, KLI74029, KLI74030, KLI74031, KLI74070), one putative regulatory protein containing a DUF3653 domain (KLI74019), one protein with phospholipase activity and a putative DNA repair role (KLI74021), one phage-related protein, and finally a peptide described as a toxin, harboring a Zot domain (KLI74023). All the resistant strains had a deletion in Contig 68 of the reference strain, within a hypothetical protein (KLI69882), known to have a tandem repeat motif, common among *Vibrio* species. In addition, several common SNPs among all the resistant strains were observed in the ribosomal 5 s, 16 s, and 23 s rRNA genes. Interestingly, genes coding for sigma factors, two-component systems, and transcription regulators were found to be usual targets of SNPs in the resistant strains studied here (Figure 5). These included the transcriptional regulator UhprA (KLI73980), known for regulating the phosphorylation of hexoses in Gram-negative bacteria, which exhibited a Val80 to Gly80 substitution in one of VaAphrodite1-resistant strain and in both the VaphiSt2-resistant strains. The MerR transcriptional regulator (KLI73845), which is known to respond to the presence of antibiotics and environmental stimuli, also showed a Tyr134 to His134 substitution in one VaAres1-resistant mutant. Additionally, the sigma factor RpoD of RNA polymerase (KLI73168) was a target of nine polymorphisms in one VaphiSt2 resistant strain and 20 in one VaAres1 resistant strain, although none of these polymorphisms resulted in amino acid substitutions or other protein effects (Supplemental Table S3). On the other hand, the gene coding for the RpoS sigma factor of RNA polymerase (KLI71089), harbored mutations in two out of three VaAphrodite1-resistant strains, including a 2-nucleotide deletion in position 150 driving a protein frameshift, and the introduction of a stop codon in position Ala151, resulting in a nonsense mutation and subsequently a merely or non-functional protein. Additionally, a substitution of Iso433 to Phe433 was observed in one VaAres1-resistant strain. Finally, one of the VaAphrodite1 mutants was also found to harbor a Tyr432 to His432 transition in a histidine kinase (KLI70462) sensor.

## 4. Discussion

Biological models of coevolving a gene for a gene between virulent bacteriophages and bacteria cannot provide explanations for the origin of various bacterial traits [53], which could be the result of a more complex molecular and metabolic strategy. In our study, we were able to pinpoint alterations in both the metabolic and phenotypic levels by generating independent phage-resistant strains for three phylogenetically distant vibriophages. We were also able to conclude that the most prominent would be to search for an adaptive mechanism with a membrane-related origin responsible for phage rejection by examining the molecular capacity of *V. alginolyticus* strain V1 and the reduced adsorption process of the resistant strains (Figure 2). Additionally, growth kinetics revealed a slight reduction in the growth of resistant strains for bacteriophage phiSt2 (Figure 1). This observation suggests a phenotypic impact, especially taking into account the very short duplication time of *Vibrio* species [54]. This indication corroborates previous results on *Vibrio* species [55], which demonstrated various novel traits (including reduced growth) of resistant bacteria to a lytic bacteriophage.

Survivability of bacteria against virulent phages depends on agile evolution [56] represented either as genomic mutations [57] and/or adjustment of an already present mechanism to counter infection [57,58]. Mutations have been demonstrated and linked with acquired resistance on motility proteins and putative transporters [59] and outer membrane proteins [54,60,61] in aquatic Gram-negative species. 

In the past, proteomic analysis of phage-bacterial interactions indicated that acquired resistance could be a result of a larger scale, fine-tuned protein regulation mechanism, including alterations of protein abundance of main phage receptors, inner membrane, and transmembrane proteins, as well as key metabolic enzymes capable of reprogramming cell metabolism [62]. 

### 4.1. Major Transcriptional Reprogramming Could Confer Phage Tolerance

**Figure 6:**
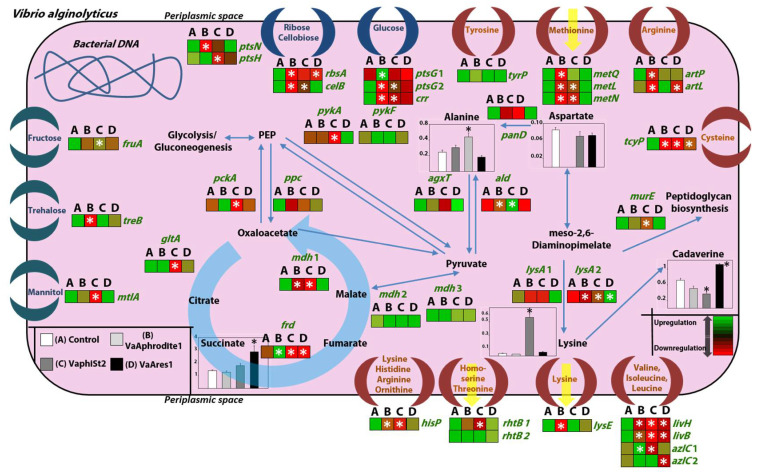
Schematic representation of metabolic reprogramming that takes place in phage-resistant strains. Relative transcript levels (heat maps) of sugar–polyol transporters (blue crescent moon) and amino acid transporters (brown crescent moon) of the inner bacterial membrane, accompanied by genes that are involved in important biochemical intracellular pathways of phage-susceptible (A) and phage-resistant (B, C, D) strains. Bars represent relative content levels of intracellular metabolites (±SE) in susceptible (white) and resistant strains in bacteriophages Aphrodite1 (light grey), phiSt2 (dark grey), and Ares1 (black). Blue arrows represent biochemical pathways in brief; the light blue round arrow represents part of the TCA cycle; yellow arrows represent putative extraction of metabolites through membrane proteins. White and black asterisks represent statistically significant differences between phage-susceptible strains and phage-resistant strains (ANOVA, post-hoc analysis Student’s *t*-test, *p* < 0.05).

Bacterial membrane protein modifications, either at the transcriptional or structural level, could represent a defensive strategy against bacteriophages [58]. Bacteriophage λ is able to utilize mainly a maltose porin for adsorption at the membrane of *E. coli* [49]. Considering infection strategies such as λ bacteriophage, putative transcriptional changes of membrane and transmembrane proteins were studied (Table 3 and Table 4, Figure 3 and Figure 6). Generally, bacteriophages recognize various receptors in the outer membrane of bacteria, which could be proteins or even polysaccharides [63]. It is also well established that the same phage genera could also utilize the same receptors for adsorption [64].

Our results showed statistically significant downregulation in the relative transcript levels of outer membrane proteins involved in phage adsorption, such as OmpF in the case of resistant strains VaAphrodite1 and VaphiSt2. OmpC, OmpF, and OmpK porin proteins have been strongly correlated with phage adsorption for *Tequatrovirus* phages and *Schizotequatroviruses* phages [65,66,67] and have also been characterized as major virulence factors [68]. Transcriptional adjustment of these proteins could correlate with phenotypic variations in bacterial virulence [69]. Additionally, *lamB* transcripts appeared to be almost depleted in the case of Ares1 and phiSt2 bacteriophages. LamB porin is the main receptor of the siphovirus λ, information that is in accordance with our results of the bacteria resistant to siphovirus Ares1 (Figure 3). This phage receptor has not been proposed to act as a binding site for *Myoviridae* phages, and the results for VaphiSt2-resistant strains show a transcriptional adjustment of non-specific phage receptors. The absence or decreased presence of the LamB porin in *E. coli* mutants was strongly correlated with decreased phage infection in the case of λ bacteriophages [15]. Decreased transcript levels were also observed for the BtuB protein in strains that interacted with the Aphrodite1 bacteriophage, a possible receptor for *Tequintavirus* bacteriophages [50]. In contrast, TolC protein, a possible receptor of podoviruses and siphoviruses, was upregulated in all three resistant strains. It is possible that by using the AcrAB-TolC efflux pump protein system, the resistant strains could enhance the extrusion of cytoplasmic nutrients and molecules [70] when other extraction channels are not functioning properly.

Loss or configuration of natural nutrient channels in both bacterial membranes of Gram-negative species interferes with the viral infection process and potentially could be involved in conferring a resistant phenotype [58,66,71,72]. Many membrane and transmembrane proteins have been linked to the viral invasion process [53], whose main role is nutrient assimilation and scavenging from the environment or the periplasmic bacterial space [19]. Our results revealed a strong transcript regulation of inner membrane proteins, including subunits localized in the cytoplasm, after phage-host interaction. We observed almost exclusively a significant reduction in mRNA levels for several transporter-coding genes, including transcripts for cysteine transporters (in all resistant strains) and the lysine exporter for VaAphrodite1 strains, where relative expression levels were almost totally depleted. Overall, results hint towards transcript reprogramming of the nutrient transport systems of the cells, which depends on the bacteriophage the bacteria have interacted with. Interestingly, the bacteriophages with the larger genome size (such as Aphrodite1 and phiSt2 with genome sizes greater than 200 kbs) resulted in more extensive transcriptional reprogramming in the cells. Significant gene expression changes in membrane and transmembrane proteins have also been reported previously [73] in Gram-negative *Pseudomonas aeruginosa* during infection with a lytic bacteriophage. Additionally, it is a well-known fact that the T4 bacteriophage causes extensive inner membrane protein redistribution in its host to achieve successful infection [11]. These results indicate that changes in the transcription of genes coding for both outer and inner membrane proteins in the host cell may play a pivotal role in conferring phage tolerance or in some cases, resistance.

### 4.2. Resistance to Lytic Bacteriophages Includes Metabolic Adaptation Mechanisms

The results of the transcript accumulation of membrane proteins hinted towards possible changes in the bacterial intracellular metabolism, a hypothesis tested by studying transcript levels of key metabolic enzymes and by monitoring the intracellular metabolome. Furthermore, possible metabolic reprogramming could account for the observed alterations in bacterial growth [55,72,74,75] and mobility [76,77,78].

In our case, the combination of transcript profiling with metabolome analysis revealed a clear shift of bacterial metabolism, linked to the development of phage resistance, and depended on the bacteriophage the bacteria interacted with (Figure 4). The observed changes in the transcription of the bacterial membrane proteins could result in extensive metabolic alterations in the tolerant bacterial strains [1,72,74,79,80] due to the failure of successfully assimilating the available nutrients in their habitat (Figure 6) [2,53,81].

Our study clearly showed an intense metabolic reprogramming of the bacteria as a result of bacteriophage resistance development, which includes accumulation or depletion of key gene transcripts and metabolites (Table 5 and Table 6, Figure 6), a fine-tuned bacteriophage-dependent process. Resistant strain VaAphrodite1 seemed to favor an alternative route for PEP production for fueling the TCA cycle, as seen by the highly induced *pckA* and *frd* genes and downregulation of *mdh*. This phenomenon agrees with the reduced content of several amino acids such as, proline, aspartic acid, glutamic acid, and pyroglutamic acid. The accumulation of ornithine also agrees with the downregulation of the *hisP* gene, an energy-providing subunit for ornithine translocation. Simultaneously, most PTS sugar transporters’ transcript levels were highly reduced, potentially resulting in a low intracellular content of sugars and polyols. These results hint towards a shift in the metabolism towards gluconeogenesis, which could require higher ATP consumption and correlate with the observed reduced transcript levels of ATP-subunits (such as *mtlA*, *crr*, *rbsA,* and *hisP*) in many transporters, which are known to be regulated allosterically [81]. Finally, these bacterial strains seem to not favor lysine conversion from the diaminopimelate (DAP) pathway, with *lysA* genes being significantly downregulated and the exported (*lysE*) having depleted transcript levels. Lysine biosynthesis is important for bacterial cell wall biosynthesis [82], and the DAP pathway being under expressed, could impose the low *lysE* transcripts regulation and subsequently reduced lysine excretion. Phage-resistant strains have a strong need for a robust lysine-rich membrane to maintain fitness and peptidoglycan integrity [83]. 

Our data suggest that bacteriophage phiSt2 causes the cell to undergo even greater metabolic reprogramming. Amino acids appear to play a key role in the metabolic state of these resistant strains. Most of the amino acids detected showed significant accumulation. Especially in the cases of lysine, glutamic acid, pyroglutamic acid, and leucine, the increased content was statistically significant. The leucine and isoleucine increased contents correlate with the decreased transcription levels of the corresponding membrane transporters, such as *livH*, *livB,* and *azlC* 1 (encoding for permeases responsible for transporting aliphatic amino acids with side chains) and their energy-providing subunit *hisP*. At the same time, depleted transcript levels of *gltA*, *mdh,* and *frd* genes were detected, hinting at highly reduced activity of the TCA cycle. It could be that the TCA cycle intermediates are being channeled towards amino acid biosynthesis such as glutamic acid, which is accumulated here, a precursor of the highly significantly accumulated lysine, a frequently targeted pathway in microbial engineering [84,85,86,87]. In fact, it is well documented that an adjusted carbon flux of the TCA cycle towards lysine production is adequate for sufficient cell growth [87]. In addition, the observed significantly reduced *murE* transcript levels, involved in peptidoglycan biosynthesis, correlate with the reduced growth observed for these resistant strains. Additionally, a significantly reduced cadaverine content could be indicative of high excretion of this particular metabolite. Gram-negative species are known to excrete cadaverine for decreasing outer membrane porins’ permeability, a mechanism that could significantly assist in achieving phage tolerance [88,89]. In addition, *pckA, pykA,* and lower level *ppc* transcripts were found to be significantly downregulated, indicating a decrease in the ATP-demanding process of gluconeogenesis and PEP abundance. Low ATP abundance could regulate the transcription of transporters allosterically as also discussed in the case of VaAphrodite1 strains. Low putative PEP abundance correlates with the statistically significantly reduced transcript levels of the HPr protein (*ptsH* gene), a protein that carries phosphorus from PEP to all PTS transport systems [18]. It is possible that an unknown mechanism controlling ATP and PEP as a bacterial community response to phage presence could orchestrate the regulation of ABC and PTS transporters, resulting in phage exclusion, but with utmost implications for cell physiology. PEP-dependent PTS systems are known to be strongly correlated with quorum sensing mechanisms in Gram-negative bacteria [90], assisting in the spreading of fine-tuned metabolic adaptation strategies to overcome various abiotic and biotic ecological niches.

Finally, in the case of resistant strain VaAres1, statistically significant accumulation of succinic acid correlated with the statistically significant downregulation of relative transcript levels of fumaric acid reductase (*frd* gene), which could indicate (i) that the cells favor the production of acetyl-CoA to fuel the TCA cycle through amino acids, such as leucine, alanine, glycine, and isoleucine, that appear to be accumulated and (ii) saves ATP molecules that could be incorporated to fuel ATP-subunits of the nutrient transport systems. At the same time, the relative transcript levels of *livH* and *livB*, which encode permeases of the ABC antiporter system of leucine and isoleucine, appeared strongly decreased, a common response of all presented phage-resistant strains. This could mean that excess of these amino acids cannot be sufficiently exported. Additionally, impaired transcription of *ptsG* genes could also be responsible for the accumulation of succinic acid [91] in Gram-negative bacteria. It is unknown whether the accumulation of succinic acid is also a result of low transcript levels in the succinic acid export protein or merely a collateral event of excess amino acids. Generally, this bacteriophage caused milder metabolic reprogramming in the cells as observed from the studied genes. 

Interestingly resistant strains for Aphrodite1 also appeared resistant to Ares1, without the two corresponding resistant strains showing a common transcript decrease in the studied outer membrane receptors. In contrast, the results revealed common decreases in the relative transcript levels of transporter proteins, which could account for common unknown metabolic shifts, concluding that it is possible for phage tolerance to be accomplished via interaction with phages that are phylogenetically distant.

**Figure 7:**
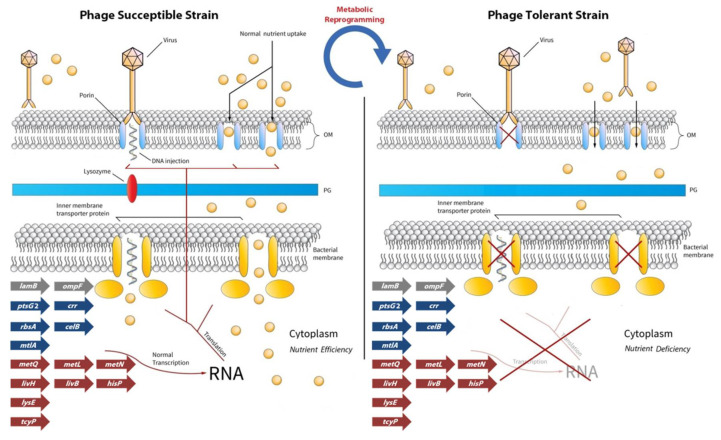
Schematic representation of how metabolic reprogramming of bacterial cells could introduce phage resistance against lytic bacteriophages after phage–host interaction. Arrows represent genes (as seen in Table 3) involved in nutrient assimilation or exportation for which their relative transcript levels were significantly downregulated compared to the control (wild-type) strains in the current study. Gray: outer membrane receptors (*lamb* and *ompF*); Blue: inner bacterial membrane sugars and polyols (*ptsG* 2, *crr, rbsA*, celB, *mtlA*); Red: inner bacterial membrane amino acids (*metQ*, *metL metN*, *livH*, *livB* and *hisP*). OM: outer membrane; PG: peptidoglycan layer.

In comparison, bacteriophage phiSt2 affected the metabolic state of the cells more radically and caused a more complicated adaptive metabolism in the cells (Figure 6 and Figure 7), a possible fact that may be a result of a combined fast and efficient lytic activity contrary to the other phages [30], rendered by its complicated genetic “armory” [31].

### 4.3. Transcription Reprogramming in Phage-Resistant Becterial Strains Could Be Triggered by Genomic Lesions

In order to better understand the observed transcriptional alterations in the *Vibrio*- resistant strains, we performed whole genome sequencing and a comparison of the independent resistant strains used in this study with the wild-type *Vibrio* strain. This comparative genomic analysis revealed that in all cases, the development of resistance to the selected phages was not accompanied by induced mutation in any of the putative phage receptors and/or nutrient transporters studied (Supplemental Table S3). In contrast, several genomic lesions were clustered in genomic regions involved in bacterial transcriptional processes [92], including RNA polymerase sigma factor RpoS, the histidine kinase Lyts family, and transcriptional regulators (Figure 5). Interestingly, sigma factor RpoS has been implicated as a main transcriptional regulator during stress responses. Impairment of this gene was previously shown to induce profound metabolic reprogramming in Gram-negative bacteria under specific growth conditions, including several genes involved in glucose transport [93]. In addition, loss of RpoS has been shown to enable the cell to utilize an alternative range of substrates for metabolic purposes [94]. Dysfunction of this sigma factor also correlates with improved nutrient scavenging and utilization of alternate metabolic pathways [95]. In addition, substitutions, such as that observed for the VaAres1 strain, can result in the radical effect of dysfunction in the highly conserved sigma70_r2 domain of the protein [94]. Histidine kinases are also known to contribute to the regulation of transcription. In our work, the LytS histidine kinase mutated in one resistant VaAphrodite1 strain, in which sigma factor RpoS was also affected. Being part of a two-component regulatory system, this kinase is known to regulate genes involved in biofilm formation and cell wall metabolism [96]. In our resistant strain, the substitution was observed in the LytS domain. The MerR HTH family of regulatory proteins was also affected in one VaAres1-resistant mutant. Most MerR family transcriptional regulators are known to respond in antibiotic or other abiotic stresses [97] and can activate suboptimal sigma70-dependent promoters [98]. DNA-binding capacity is also altered when mutations are introduced in Gram-negative species, subsequently affecting its transcriptional role [97]. Combined mutations in both MerR and its promoter have revealed several and various phenotypes in Gram-negative species [97]. Regarding the transcriptional regulator UhpA, substitutions in the REC-domain, which acts as a phosphoroacceptor, could positively impact the phosphorus-binding feature of the protein, as previously seen [99]. The UhpA regulatory protein is known for controlling the expression of the hexose phosphate transporter UhpT by binding to its promoter. Increased phosphorylation of UhpA could result in decreased binding to the promoter of UhpT transporter and subsequently, decreased expression of the regulated metabolic pathway [100]. Strong correlations between transcriptional regulation of *ptsH* and UhpA system were shown previously [101]. In our work, the *ptsH* gene was significantly downregulated in VaphiSt2 strains (Figure 6), in which the *uprA* regulator exhibited SNPs. Inorganic phosphorus could significantly affect how cells reprogram their PTS system transcription [101] and subsequently could play a pivotal role in how cells develop phage resistance against specific bacteriophages in certain ecological niches.

Taken together, the observed mutations point towards a molecular mechanism resulting in the drastic transcriptional alterations observed in the current work and subsequently, to metabolic reprogramming of bacteriophage-resistant strains. To this end, our work is in line with previous work on *Vibrio anguillarum* resistant mutants, which showed various mutations between resistant mutants for a lytic bacteriophage and that transcriptional regulators and sigma factors can be targets of SNPs, with diverse metabolical and phenotypical effects [55]. Our work corroborates that study in the *Vibrio* genus. In any case, complementation experiments have to be performed in order to elucidate the precise molecular role of these genes in the induced phage resistance mechanism. In our case, we could not detect mutations in the case of the main receptors or their possible promoters (Supplemental Table S3), as was reported previously [55]. It is possible that for the *Vibrio* genus, spontaneous mutations in phage receptors are not required for impaired adsorption or/and infection.

## 5. Conclusions

The results presented here complement previous reports on the alterations taking place at the genomic and metabolic level after lytic phage-host interactions and raises the hypothesis of a far more complicated adaptation strategy for Gram-negative bacteria against lytic bacteriophages related to phage resistance (Figure 7). By combining data from different approaches, we examined a new perspective, that interaction could impose a complicated transcriptional-dependent resistance adaptation strategy, with metabolic implications on cell physiology. The observed metabolic reprogramming appeared to be customized for every bacteriophage that interacted with *V. alginolyticus*. It is possible that the limited host range of many bacteriophages observed for Gram-negative species is due to the metabolic state of the studied cells, a result of their habitat, nutrient availability, and/or ancestry phage interactions. This hypothesis could impact and update primarily the role of bacteriophages in microbial ecology in various habitats; in our case, the marine realm [102,103]. New experimental strategies could also elucidate how quorum sensing contributes to the spreading of a fine-tuned metabolic adaptation state for successful tolerance [104,105]. Detailed genomic characterization of phage-resistant mutants and in-depth analysis of SNPs in the future will also assist in elucidating the metabolic phenotype reported here. Future results from other Gram-negative phage-resistant strains and multiphage-resistant strains will show how widespread and agile this metabolic response is. By examining the complicated metabolic and genomic adaptations that are taking place, phage biology in the future could reveal that phenomenon and render a more successful phage therapy.

## Figures and Tables

**Table 1:** Main characteristics of the phages used in this study.

Phage	Family	Genus	Lifestyle	Genome (bps)	GC %	Predicted # ORFs	Latent Period	Accession Number	Reference
Aphrodite1	*Myoviridae*	*Aphroditevirus*	Lytic	237.722	43.4	207	80 min	MG720308	This study
phiSt2	*Myoviridae*	*Schizotequatrovirus*	Lytic	250.485	42.6	412	30 min	KT919973	[30,31]
Ares1	*Siphoviridae*	unclassified	Unknown	80.500	45.1	119	30 min	MG720309	This study

Abbreviations: bps: base pairs; GC: genomic GC content; ORFs: open reading frames.

**Table 2:** Cross-infection test (- for the absence of lytic plaques; + for high lytic activity) of wild-type and resistant strains against highly lytic bacteriophages described in the present study.

Bacteriophages with High Lytic Activity			
*Vibrio alginolyticus* Strains	Aphrodite1	phiSt2	Ares1
Control (wild type)	+	+	+
VaAphrodite1	-	+	-
VaphiSt2	+	-	+
VaAres1	+	+	-

**Table 3:** Main characteristics of the inner membrane-related proteins that were studied in phage-susceptible and phage-resistant strains.

Gene Name	Nutrient Transport Participation	Type of Transporter	Subunit	Subunit Role
*mtlA*	Mannitol	PTS	IIA, IIB, IIC	Affinity, Transport, Energy
*treB*	Trehalose	PTS	IIB, IIC	Affinity, Transport
*fruA*	Fructose	PTS	IIB, IIC	Affinity, Transport
*celB*	Cellulose	PTS	IIC	Affinity
*ptsG 1*	Glucose	PTS	IIB, IIC	Affinity, Transport
*ptsG 2*	Glucose	PTS	IIB, IIC	Affinity, Transport
*crr*	Glucose	PTS	IIB, IIC	Energy
*ptsN*	Nitrogen	PTS	IIA	Transport
*ptsH*	Phosphorus	PTS	IIA	Transport
*tyrP*	Tyrosine	-	-	
*rbsA*	Ribose	ABC	ATP-subunit	Energy
*metQ*	Methionine	ABC	IIA	Affinity
*metl*	Methionine	ABC	Substrate subunit	Transport
*metN*	Methionine	ABC	Transmembrane subunit	Energy
*artP*	Arginine	ABC	Substrate subunit	Affinity
*artL*	Arginine	ABC	ATP-subunit	Energy
*tcyP*	Cysteine	-	-	Transport
*lysE*	Lysine	LysE-like	-	Lysine Export
*rhtB* 1	Homoserine and Threonine	LysE-like	-	Homoserine and Threonine Export
*rhtB* 2	Homoserine and Threonine	LysE-like	-	Homoserine and Threonine Export
*hisP*	Lysine, Histidine, Arginine, Ornithine	ABC	ATP-subunit	Energy
*azlC* 1	Valine, Isoleucine, Leucine	ABC	Transmembrane subunit	Transport
*azlC* 2	Valine, Isoleucine, Leucine	ABC	Transmembrane subunit	Transport
*livH*	Valine, Isoleucine, Leucine	ABC	Transmembrane subunit	Transport
*livM*	Valine, Isoleucine, Leucine	ABC	Transmembrane subunit	Transport

**Table 4:** Heat maps of relative transcript levels of some of the genes that were altered for every resistant strain expressed as a ratio vs. Control strains (r > 1 represents upregulation, 0 < r < 1 represents downregulation). Asterisks indicate statistically significant differences compared to control (wild-type) strains (Student’s *t*-test; *p* < 0.05).

Expression Ratio vs. Control										
Gene			VaAphrodite1			VaphiSt2		VaAres1		
*livH*			*			*		*		
*livB*			*			*		*		
*metl*			*			*				
*metN*			*			*				
*metQ*			*							
*tcyp*			*			*		*		
*lysE*			*							
*hisp*			*			*				
*ptsG* *1*			*							
*ptsG* *2*			*			*				
*crr*			*			*				
*rbsH*			*					*		
*cellB*			*			*				
*ptsH*						*				
*murE*						*				
*gltA*						*				
*frd*			*			*				
*mdh* *1*			*			*				
*pykA*						*				
*pykF*										
*pckA*			*			*				
r > 10	4 < r < 10	2 < r < 4	1.41 < r < 2	1.19 < r < 1.41	0.84 < r < 1.19	0.71 < r < 0.84	0.5 < r < 0.71	0.25 < r < 0.5	0.1 < r < 0.25	r < 0.1

**Table 5:** Main metabolic genes and their corresponding coding enzymes that were studied in phage-susceptible and phage-resistant strains.

Gene Name	Coding Enzyme	E.C. Number	Metabolic Pathway
*ald*	Alanine dehydrogenase	1.4.11	Alanine metabolism
*agxT*	Alanine-glycosylate and serine pyruvate aminotransferase	2.6.1.44	Alanine metabolism
*panD*	aspartate 4-decarboxylase; desulfinase	4.1.1.12	Alanine metabolism
*lysA 1*	Lysine decarboxylase	4.1.1.20	Lysine metabolism
*lysA 2*	Lysine decarboxylase	4.1.1.20	Lysine metabolism
*murE*	Amino acid ligase	6.3.2.13	Peptidoglycan biosynthesis
*gltA*	Citrate synthase	2.3.3.16	TCA cycle
*mdh 1*	Malic acid decarboxylase	1.1.1.37	TCA cycle
*frd*	Fumarate reductase	1.3.5.4	TCA cycle
*pykA*	Pyruvate kinase	2.7.1.40	Anaplerotic reactions of the TCA cycle
*pykF*	Pyruvate kinase	2.7.1.40	Anaplerotic reactions of the TCA cycle
*pckA*	PEP carboxykinase	4.1.1.49	Anaplerotic reactions of the TCA cycle
*mdh 2*	Malic acid dehydrogenase	1.1.1.38	Anaplerotic reactions of the TCA cycle
*mdh 3*	Malic acid dehydrogenase	1.1.1.39	Anaplerotic reactions of the TCA cycle
*ppc*	PEP carboxylase	4.1.1.31	Anaplerotic reactions of the TCA cycle

**Table 6:** Average relative response of intracellularly detected compounds and their respective retention time, mass charge ratio, and *p*-value (ANOVA, post-hoc Student’s *t*-test, <0.05; Control *n* = 4; Resistant strains *n* = 5). Bold values represent statistical significance.

Compound	RT	M/Z	Control	VaAphrodite1	VaphiSt2	VaAres1	*p*
Phenylalanine	25.90	218	0.046	0.068	0.079	0.087	0.093
Lysine	32.19	73	0.037	0.053	**0.558**	0.025	**0.000**
Isoleucine	17.34	158	0.074	0.091	0.113	0.105	0.129
Alanine	12.05	116	0.246	0.184	0.304	**0.440**	**0.000**
Ornithine	30.02	142	0.024	**0.127**	0.080	**0.000**	**0.000**
Proline	17.54	142	0.059	**0.000**	0.057	0.072	**0.000**
Valine	15.19	144	0.121	0.170	0.183	0.174	0.243
Glycine	17.73	174	0.084	0.157	0.121	**0.219**	**0.032**
Leucine	16.73	158	0.161	0.239	**0.376**	**0.532**	**0.002**
Aspartic acid	23.21	232	0.086	**0.000**	0.071	0.073	**0.000**
Glutamic acid	25.61	246	0.248	**0.116**	**0.486**	0.343	**0.000**
Pyroglutamic acid	23.37	156	0.121	0.092	**0.239**	0.156	**0.001**
Norleucine	16.73	158	0.178	0.238	0.324	**0.347**	**0.047**
Putrescine	28.39	174	0.166	0.121	**0.268**	0.201	**0.000**
Cadaverine	30.52	174	0.717	**1.120**	**0.361**	0.507	**0.000**
Succinic acid	17.92	148	1.394	1.271	1.784	**2.881**	**0.003**
Pipecolic acid	23.37	156	0.000	**0.074**	0.000	0.000	**0.000**

## Data Availability

The data is contained within the article or Supplementary Material.

## Supplementary Materials

The following are available online at https://www.mdpi.com/article/10.3390/v13040656/s1; Supplemental Data 1. Biological characterization of bacteriophage Aphrodite1; Supplemental Data 2. Biological characterization of bacteriophage Ares1; Figure S1. Growth kinetics of phage-resistant and phage-susceptible strains in detail. Table S1. Relative transcript abundance of genes studied. Table S2. Primer list for the real-time qPCR platform. Table S3. List of polymorphisms that were localized in the three respective resistant strains.
