# Supplementary material for: The Development of Bacteriophage Resistance in Vibrio alginolyticus Depends on a Complex Metabolic Adaptation Strategy
Source: Viruses. 2021 Apr 10;13(4):656. doi: 10.3390/v13040656 (PMC8069663; doi:10.3390/v13040656)

Skliros et al., 2021. Figure S1. Growth kinetics of phage-resistant and phage susceptible strains in detail. Growth (average, ±SE) of Control (phage susceptible strains; black circle; n=6), VaAphrodite1 strains (phage resistant strains to bacteriophage Aphrodite1; white circle; n=9), VaphiSt2 strains (phage resistant strains to bacteriophage *φ*St2; black triangle; n=9) and VaAres1 strains (phage resistant strains to bacteriophage Ares1; white triangle; n=9). Second diagram represents VaphiSt2 strains (phage resistant strains to bacteriophage *φ*St2; n=3).

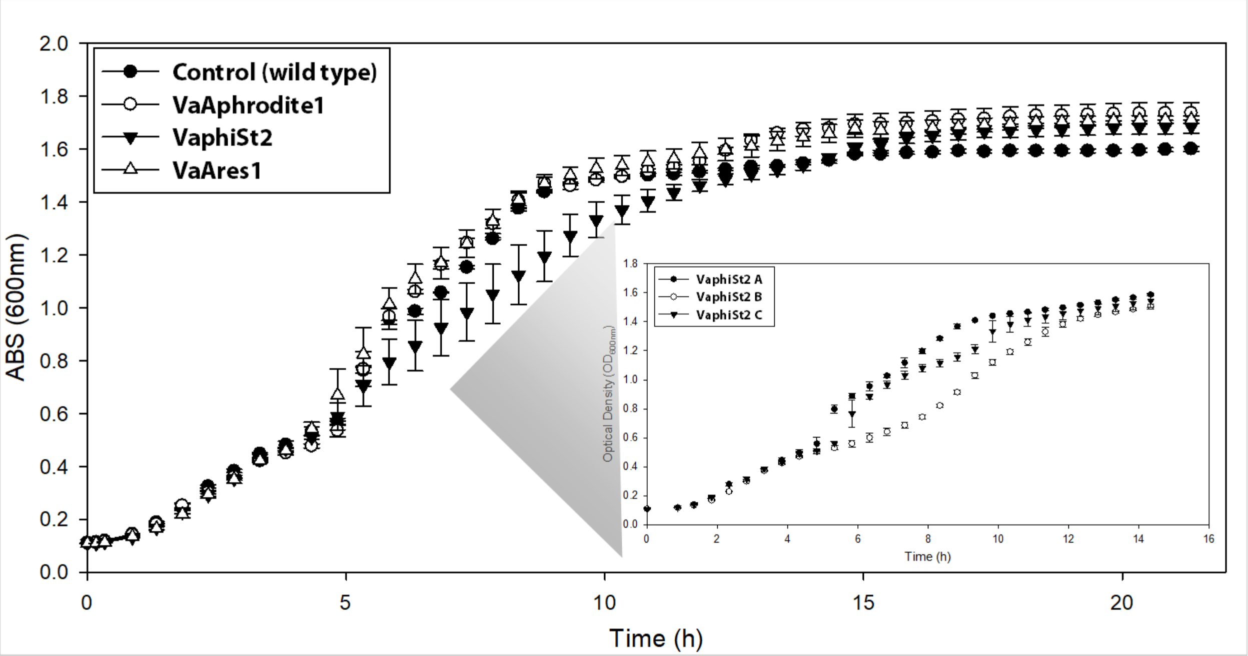

Supplement: Supplementary file 1 [file viruses-13-00656-s001.zip › supplementary 1050102/Figure S1.pdf]
