# Supplementary material for: The Development of Bacteriophage Resistance in Vibrio alginolyticus Depends on a Complex Metabolic Adaptation Strategy
Source: Viruses. 2021 Apr 10;13(4):656. doi: 10.3390/v13040656 (PMC8069663; doi:10.3390/v13040656)
Supplement: Supplementary file 1 [file viruses-13-00656-s001.zip › supplementary 1050102/Supplemental Data 1.pdf]

Skliros et al., 2020. Supplemental data 1.

Biological and molecular characterization of bacteriophage Aphrodite1

Bacteriophage Aphrodite1 (**Figure1**) owns the accession number in genbank MG720308 and has been isolated from the aquatic area of North Euboea Greece. Capsid diameter corresponds to approximately105 nm ±10.8 nm. Not contractile tail length is 144 ±10.6 nm (n=6). *In vitro* assay revealed that even after 10 hours (with MOI:100), is able to supress bacterial cultures **(Figure 2)**. 95 % of the phage particles are able to adsorb at 12 min and owns a long replication time of around 80 min **(Figure 3)**.

Genome analysis revealed a large genome size of 237.722 bps and GC content of 43.4 %. Its genome is able to translate at least 198 potential protein products **(Table1)**. Interestingly lacks the present of tRNAs. Taxonomy according to ICTV show that this bacteriophage belongs to genus *Aphroditevirus*. Genome orientation and transcriptional direction agrees with the most of the CDSs. Purine excess is strongly correlated with the leading transcriptional strand.

GenBank Record

Draft genome of Aphrodite1 MG720308 Skliros, D., Kalatzis, P.G., Katharios, P., Flemetakis, E., 2017

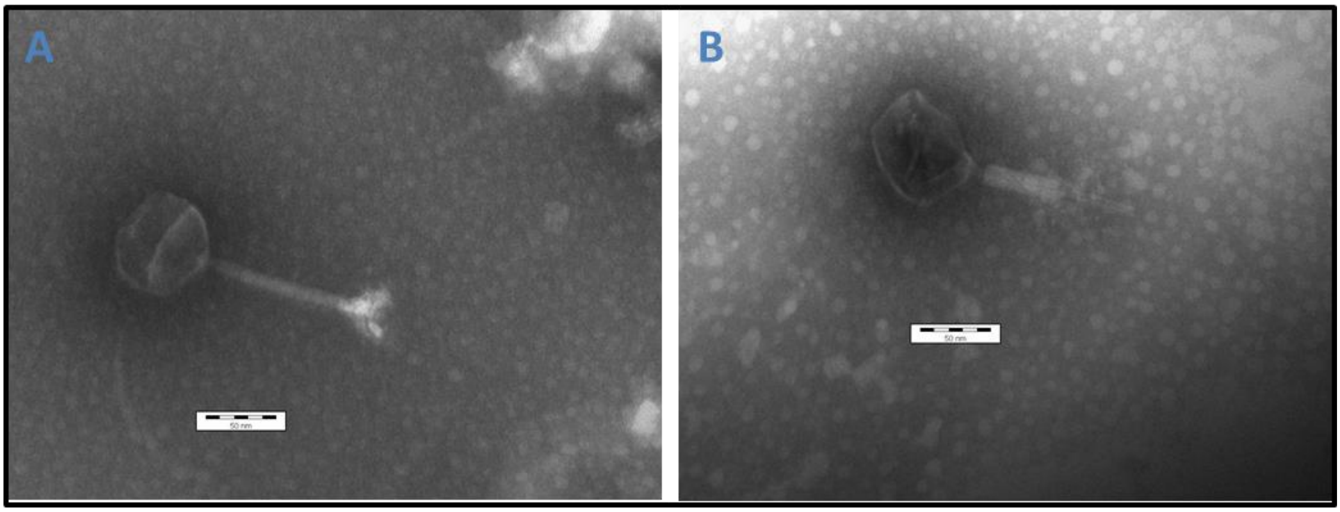

Figure 1: TEM image of bacteriophage Aphrodite1, a typical myovirus morphotype. Large capsid size is shown in A and the contracted tail in B.

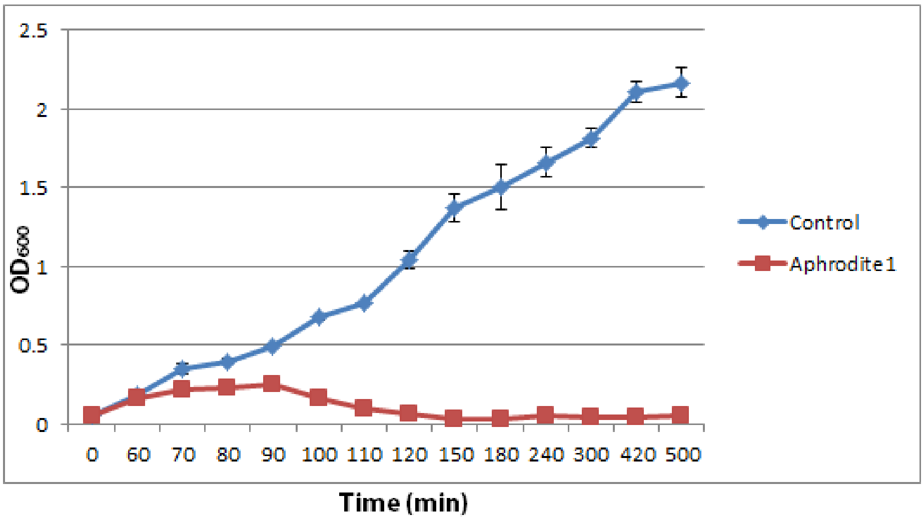

Figure 2: *In vitro* efficacy challenge with MOI:100 co-cultured with the main host *Vibrio alginolyticus* strain V1. Even after 10 hours the bacteria culture appears suppressed.

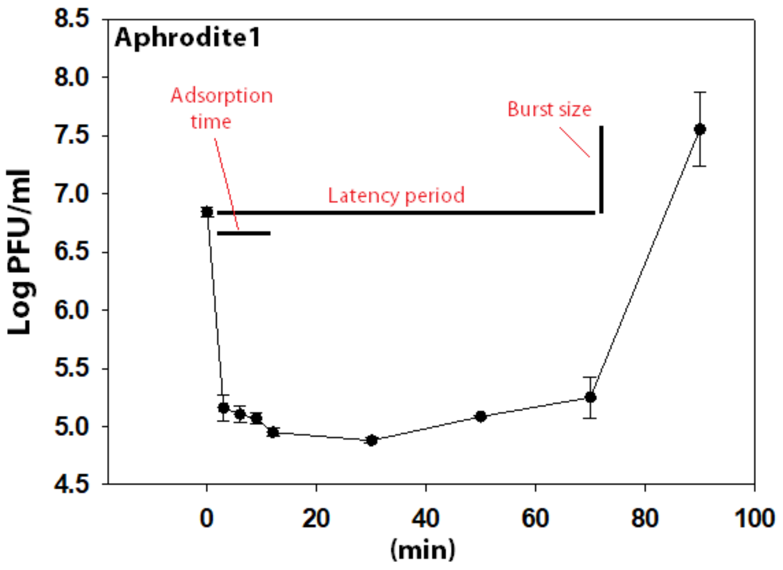

Figure 3: One-step growth curve revealed biological characteristics of the phage. Interestingly Aphrodite1 has a large latency period, which is approximately 80 min.

Table 1. Open reading frames of bacteriophage Aphrodite1.

Name	Length (nt)	Start	Finish	Orientation	Protein_id
putative virion structural protein CDS	3	635	633	forward	AUR81025.1
hypothetical protein CDS	637	1149	513	forward	AUR81040.1
hypothetical protein CDS	1151	1885	735	forward	AUR81010.1
hypothetical protein CDS	1946	2449	504	reverse	AUR81043.1
hypothetical protein CDS	2513	3115	603	forward	AUR81029.1
hypothetical protein CDS	3096	3497	402	forward	AUR81070.1
hypothetical protein CDS	3593	4723	1131	forward	AUR80974.1
hypothetical protein CDS	4776	6950	2175	reverse	AUR80924.1
endolysin CDS	7000	14055	7056	reverse	AUR80910.1
hypothetical protein CDS	14078	15685	1608	forward	AUR80938.1
putative RNA polymerase beta subunit CDS	15688	20037	4350	forward	AUR80913.1
hypothetical protein CDS	20040	20894	855	forward	AUR80998.1
hypothetical protein CDS	20894	21055	162	forward	AUR81099.1
hypothetical protein CDS	21058	22548	1491	forward	AUR80945.1
hypothetical protein CDS	22565	23299	735	forward	AUR81009.1
putative virion structural protein CDS	23292	24185	894	forward	AUR80994.1
hypothetical protein CDS	24297	24962	666	forward	AUR81019.1
hypothetical protein CDS	25076	25522	447	forward	AUR81057.1
hypothetical protein CDS	25671	26114	444	forward	AUR81060.1
hypothetical protein CDS	26185	26298	114	forward	AUR81105.1
hypothetical protein CDS	26295	26777	483	forward	AUR81050.1
hypothetical protein CDS	26780	27439	660	forward	AUR81020.1
hypothetical protein CDS	27436	27918	483	forward	AUR81049.1
hypothetical protein CDS	27915	28433	519	forward	AUR81038.1
hypothetical protein CDS	28472	28951	480	forward	AUR81051.1
putative metal-dependent phosphohydrolase CDS	29082	29870	789	forward	AUR81003.1
thymidylate synthase CDS	29872	30780	909	forward	AUR80992.1

hypothetical protein CDS	31362	31508	147	forward	AUR81102.1
hypothetical protein CDS	32153	32869	717	forward	AUR81015.1
hypothetical protein CDS	32873	33442	570	forward	AUR81032.1
hypothetical protein CDS	33445	33834	390	forward	AUR81075.1
hypothetical protein CDS	33831	34988	1158	forward	AUR80972.1
hypothetical protein CDS	35218	35589	372	forward	AUR81076.1
hypothetical protein CDS	35589	35888	300	forward	AUR81093.1
hypothetical protein CDS	35976	36239	264	forward	AUR81097.1
putative tail tube protein CDS	36397	37296	900	reverse	AUR80993.1
hypothetical protein CDS	37301	39436	2136	reverse	AUR80926.1
putative virion structural protein CDS	39582	40460	879	forward	AUR80997.1
hypothetical protein CDS	40485	43067	2583	forward	AUR80918.1
putative virion structural protein CDS	43060	47826	4767	forward	AUR80911.1
hypothetical protein CDS	47837	58426	10590	forward	AUR80908.1
lg-like domain protein CDS	58574	66220	7647	forward	AUR80909.1
hypothetical protein CDS	66309	69524	3216	forward	AUR80915.1
thymidylate kinase CDS	69708	70331	624	reverse	AUR81027.1
putative virion structural protein CDS	70390	71760	1371	reverse	AUR80954.1
putative virion structural protein CDS	71760	72605	846	reverse	AUR80999.1
hypothetical protein CDS	72895	73488	594	forward	AUR81031.1
hypothetical protein CDS	75147	75539	393	forward	AUR81073.1
hypothetical protein CDS	76031	77035	1005	forward	AUR80986.1
hypothetical protein CDS	77066	78085	1020	forward	AUR80984.1
hypothetical protein CDS	79507	80265	759	forward	AUR81006.1
hypothetical protein CDS	80593	81372	780	forward	AUR81005.1
hypothetical protein CDS	81444	82622	1179	forward	AUR80971.1
lactose operon transcriptional activator CDS	82610	83335	726	forward	AUR81012.1
Msm operon regulatory protein CDS	83328	84029	702	forward	AUR81017.1
chain A monomeric subunit of Tubz CDS	84528	85493	966	forward	AUR80988.1
hypothetical protein CDS	85889	86344	456	forward	AUR81055.1
hypothetical protein CDS	86860	87189	330	forward	AUR81086.1
hypothetical protein CDS	87179	87505	327	forward	AUR81088.1

hypothetical protein CDS	87483	87812	330	forward	AUR81085.1
hypothetical protein CDS	87842	88249	408	forward	AUR81068.1
hypothetical protein CDS	88315	88752	438	forward	AUR81062.1
hypothetical protein CDS	88811	90883	2073	forward	AUR80930.1
hypothetical protein CDS	90919	92025	1107	reverse	AUR80977.1
hypothetical protein CDS	92248	94314	2067	forward	AUR80931.1
putative DNA helicase CDS	94409	95896	1488	forward	AUR80946.1
hypothetical protein CDS	96221	96409	189	forward	AUR81098.1
hypothetical protein CDS	96409	97065	657	forward	AUR81021.1
hypothetical protein CDS	97091	97567	477	forward	AUR81052.1
hypothetical protein CDS	97641	97769	129	forward	AUR81104.1
putative virion structural protein CDS	97832	98254	423	forward	AUR81064.1
hypothetical protein CDS	98235	99122	888	forward	AUR80995.1
hypothetical protein CDS	99144	100268	1125	forward	AUR80975.1
hypothetical protein CDS	100265	101062	798	forward	AUR81002.1
hypothetical protein CDS	101136	101858	723	forward	AUR81014.1
hypothetical protein CDS	101972	103546	1575	forward	AUR80940.1
hypothetical protein CDS	103597	105078	1482	forward	AUR80947.1
hypothetical protein CDS	105165	106580	1416	forward	AUR80952.1
hypothetical protein CDS	106647	107273	627	forward	AUR81026.1
hypothetical protein CDS	107326	107646	321	reverse	AUR81090.1
hypothetical protein CDS	107742	109844	2103	forward	AUR80928.1
DNA-directer RNA polymerase beta subunit CDS	109822	111807	1986	forward	AUR80932.1
putative helicase CDS	111930	113348	1419	forward	AUR80951.1
hypothetical protein CDS	113415	113765	351	forward	AUR81082.1
hypothetical protein CDS	113770	114186	417	forward	AUR81065.1
hypothetical protein CDS	114159	114566	408	forward	AUR81067.1
hypothetical protein CDS	114579	115019	441	forward	AUR81061.1
hypothetical protein CDS	115192	116490	1299	reverse	AUR80959.1
putative virion structural protein CDS	116465	117391	927	reverse	AUR80990.1
hypothetical protein CDS	117421	118146	726	forward	AUR81011.1
hypothetical protein CDS	118139	119761	1623	forward	AUR80937.1

hypothetical protein CDS	119818	120216	399	forward	AUR81072.1
hypothetical protein CDS	120293	120850	558	forward	AUR81035.1
hypothetical protein CDS	121002	121361	360	forward	AUR81079.1
putative DNA polymerase CDS	121424	123190	1767	reverse	AUR80935.1
putative virion structural protein CDS	123274	124602	1329	forward	AUR80958.1
hypothetical protein CDS	124604	125014	411	forward	AUR81066.1
tail length tape-measure protein CDS	125025	126488	1464	forward	AUR80948.1
hypothetical protein CDS	126485	127207	723	forward	AUR81013.1
hypothetical protein CDS	127254	130193	2940	reverse	AUR80916.1
putative virion structural protein CDS	130175	131218	1044	reverse	AUR80982.1
capsid and scaffold protein CDS	131254	132375	1122	forward	AUR80976.1
putative virion structural protein CDS	132375	133256	882	forward	AUR80996.1
hypothetical protein CDS	133268	133777	510	forward	AUR81041.1
hypothetical protein CDS	133770	135035	1266	forward	AUR80963.1
hypothetical protein CDS	135038	135991	954	forward	AUR80989.1
hypothetical protein CDS	136049	137332	1284	forward	AUR80961.1
hypothetical protein CDS	137406	138596	1191	forward	AUR80969.1
hypothetical protein CDS	138614	139813	1200	forward	AUR80968.1
hypothetical protein CDS	139918	141168	1251	forward	AUR80964.1
hypothetical protein CDS	141215	142489	1275	forward	AUR80962.1
hypothetical protein CDS	142614	143852	1239	forward	AUR80966.1
hypothetical protein CDS	143943	145031	1089	forward	AUR80979.1
hypothetical protein CDS	145041	146453	1413	forward	AUR80953.1
putative virion structural protein CDS	146523	148127	1605	forward	AUR80939.1
putative virion structural protein CDS	148131	149420	1290	forward	AUR80960.1
hypothetical protein CDS	149422	150123	702	forward	AUR81016.1
putative virion structural protein CDS	150125	151456	1332	forward	AUR80956.1
hypothetical protein CDS	151576	153015	1440	forward	AUR80949.1
hypothetical protein CDS	153072	153470	399	forward	AUR81071.1
hypothetical protein CDS	153686	153997	312	forward	AUR81091.1
hypothetical protein CDS	154235	155074	840	forward	AUR81000.1
hypothetical protein CDS	155071	155568	498	forward	AUR81045.1

hypothetical protein CDS	155565	156071	507	forward	AUR81042.1
hypothetical protein CDS	156086	156490	405	forward	AUR81069.1
hypothetical protein CDS	156487	156945	459	forward	AUR81054.1
hypothetical protein CDS	157360	159183	1824	forward	AUR80934.1
hypothetical protein CDS	159188	159538	351	forward	AUR81081.1
hypothetical protein CDS	159600	159926	327	forward	AUR81087.1
DNA helicase CDS	159998	161536	1539	reverse	AUR80942.1
hypothetical protein CDS	161582	162148	567	forward	AUR81033.1
capsid and scaffold protein CDS	162248	164443	2196	forward	AUR80923.1
hypothetical protein CDS	164625	165695	1071	forward	AUR80980.1
hypothetical protein CDS	165739	167412	1674	forward	AUR80936.1
hypothetical protein CDS	167409	169892	2484	forward	AUR80920.1
hypothetical protein CDS	169952	170515	564	forward	AUR81034.1
hypothetical protein CDS	170519	171523	1005	forward	AUR80985.1
hypothetical protein CDS	171520	172269	750	forward	AUR81008.1
hypothetical protein CDS	172420	172863	444	forward	AUR81059.1
hypothetical protein CDS	172948	173313	366	forward	AUR81078.1
hypothetical protein CDS	173363	176269	2907	reverse	AUR80917.1
hypothetical protein CDS	176290	178401	2112	forward	AUR80927.1
putative virion structural protein CDS	178461	179375	915	forward	AUR80991.1
hypothetical protein CDS	179441	179977	537	forward	AUR81036.1
hypothetical protein CDS	179958	180311	354	forward	AUR81080.1
hypothetical protein CDS	180359	180505	147	reverse	AUR81101.1
hypothetical protein CDS	180801	181190	390	reverse	AUR81074.1
hypothetical protein CDS	181194	182429	1236	reverse	AUR80967.1
tail fiber protein CDS	182508	184649	2142	forward	AUR80925.1
minor tail protein CDS	184770	188039	3270	forward	AUR80914.1
minor tail protein CDS	188078	190288	2211	forward	AUR80922.1
hypothetical protein CDS	190290	192800	2511	forward	AUR80919.1
phage baseplate protein CDS	192849	193901	1053	reverse	AUR80981.1
DNA double-strand break repair Rad50 ATPase CDS	193952	196429	2478	forward	AUR80921.1
hypothetical protein CDS	196520	196792	273	forward	AUR81096.1

hypothetical protein CDS	196853	197296	444	forward	AUR81058.1
hypothetical protein CDS	197306	197941	636	forward	AUR81024.1
DNA ligase CDS	197928	199889	1962	forward	AUR80933.1
hypothetical protein CDS	199920	200222	303	forward	AUR81092.1
hypothetical protein CDS	200250	201494	1245	forward	AUR80965.1
hypothetical protein CDS	201552	202034	483	forward	AUR81048.1
hypothetical protein CDS	202207	203175	969	forward	AUR80987.1
ribonuclease HI CDS	203200	203724	525	forward	AUR81037.1
hypothetical protein CDS	203721	204041	321	forward	AUR81089.1
hypothetical protein CDS	204104	204595	492	forward	AUR81047.1
hypothetical protein CDS	204605	205249	645	forward	AUR81022.1
hypothetical protein CDS	205252	206397	1146	forward	AUR80973.1
heat shock protein 60 family chaperone CDS	206470	208029	1560	forward	AUR80941.1
hypothetical protein CDS	208130	208279	150	forward	AUR81100.1
hypothetical protein CDS	208937	209065	129	forward	AUR81103.1
thymidine kinase CDS	209195	209791	597	forward	AUR81030.1
hypothetical protein CDS	209850	211034	1185	forward	AUR80970.1
hypothetical protein CDS	211108	215631	4524	forward	AUR80912.1
hypothetical protein CDS	215759	216511	753	forward	AUR81007.1
hypothetical protein CDS	216559	217578	1020	forward	AUR80983.1
hypothetical protein CDS	217582	217914	333	forward	AUR81083.1
hypothetical protein CDS	218005	218457	453	forward	AUR81056.1
hypothetical protein CDS	218505	219002	498	forward	AUR81044.1
hypothetical protein CDS	219004	219474	471	forward	AUR81053.1
hypothetical protein CDS	219494	219985	492	forward	AUR81046.1
DNA helicase CDS	220048	222129	2082	reverse	AUR80929.1
hypothetical protein CDS	222177	223532	1356	forward	AUR80955.1
hypothetical protein CDS	223585	223875	291	forward	AUR81095.1
hypothetical protein CDS	224771	226294	1524	forward	AUR80944.1
hypothetical protein CDS	226342	227433	1092	forward	AUR80978.1
hypothetical protein CDS	227423	227719	297	forward	AUR81094.1
hypothetical protein CDS	227776	228288	513	forward	AUR81039.1

hypothetical protein CDS	228295	229074	780	forward	AUR81004.1
hypothetical protein CDS	229074	229748	675	forward	AUR81018.1
hypothetical protein CDS	229760	230377	618	forward	AUR81028.1
hypothetical protein CDS	230367	230696	330	forward	AUR81084.1
hypothetical protein CDS	230758	231393	636	reverse	AUR81023.1
putative virion structural protein CDS	231386	232714	1329	reverse	AUR80957.1
hypothetical protein CDS	232755	233186	432	forward	AUR81063.1
hypothetical protein CDS	233279	234811	1533	forward	AUR80943.1
hypothetical protein CDS	234850	235659	810	reverse	AUR81001.1
hypothetical protein CDS	235716	237152	1437	forward	AUR80950.1
hypothetical protein CDS	237212	237577	366	forward	AUR81077.1
