# Supplementary material for: The Development of Bacteriophage Resistance in Vibrio alginolyticus Depends on a Complex Metabolic Adaptation Strategy
Source: Viruses. 2021 Apr 10;13(4):656. doi: 10.3390/v13040656 (PMC8069663; doi:10.3390/v13040656)
Supplement: Supplementary file 1 [file viruses-13-00656-s001.zip › supplementary 1050102/Supplemental Data 2.pdf]

Skliros et al., 2020. Supplemental data 2.

Biological and molecular characterization of bacteriophage Ares1

Bacteriophage Ares1 (**Figure 1**) owns the accession number in genbank MG720309 and has been isolated from the aquatic area of South Attika, Greece. Capsid diameter corresponds to approximately ~49 ± 6.8 (n=5) nm. Not contractile tail length is 160 ± 14 nm. 95 % of the phage particles are able to adsorb at 6 min (**Figure 3**) and owns a latent period of 30 min (**Figure 3**). *In vitro* assay revealed that even after 7 hours (with MOI:100), is able to suppress bacterial culture (**Figure 2**).

Genome analysis revealed a genome size of 80500 bps and GC content of 45.1 %. Its genome is able to translate at least 119 ORFs (**Table 1**). TEM images revealed a typical siphophage morphotype with a strong lytic activity (**Figure 4**). ICTV has not yet classified this particular virus to a genus. In silico annotation process showed similarity with siphovirus VHS1, thus enabling us to proceed to a whole genome alignment representation, utilizing MAUVE aligner software. Genome orientation and transcriptional direction agrees with the most of the CDSs. Purine excess is strongly correlated with the leading transcriptional strand.

GenBank Record

Draft genome of Ares1 MG720309 Skliros, D., Kalatzis, P.G., Katharios, P., Flemetakis, E., 2017

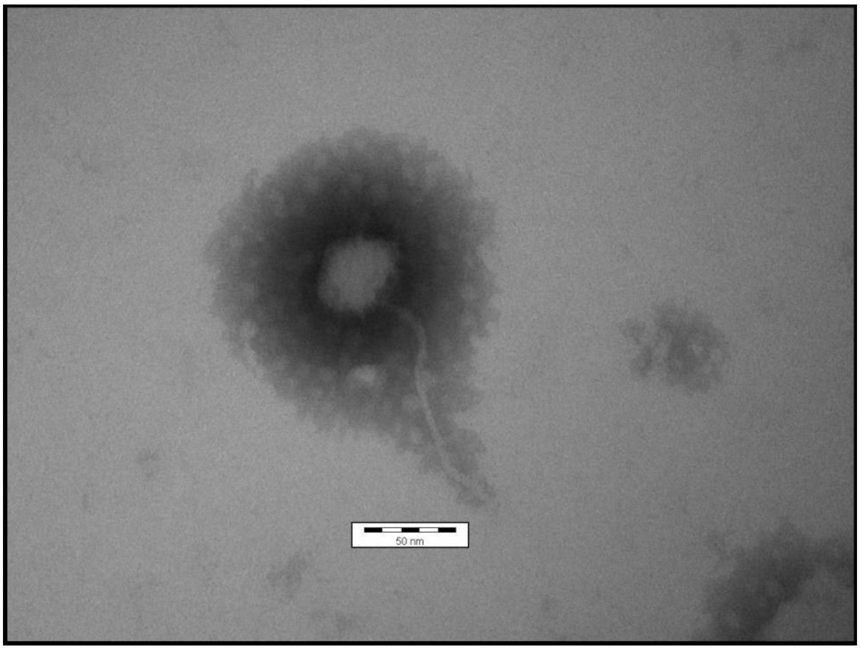

Figure 1: TEM image of bacteriophage Ares1 revealing a long tail structure, typical of siphovirus morphotype.

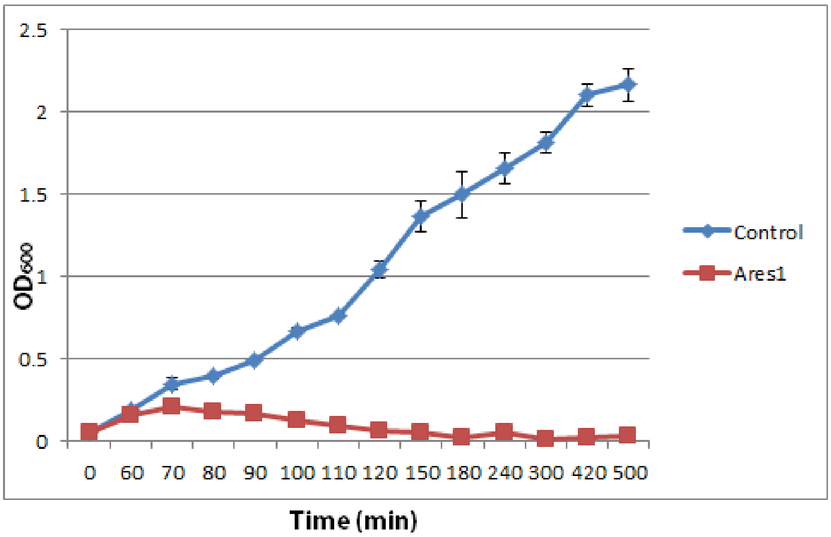

Figure 2: *In vitro* efficacy challenge with MOI:100 of bacteriophage Ares1 co-cultured with the main host *Vibrio alginolyticus* strain V1. Even after 7 hours the bacteria culture appears suppressed.

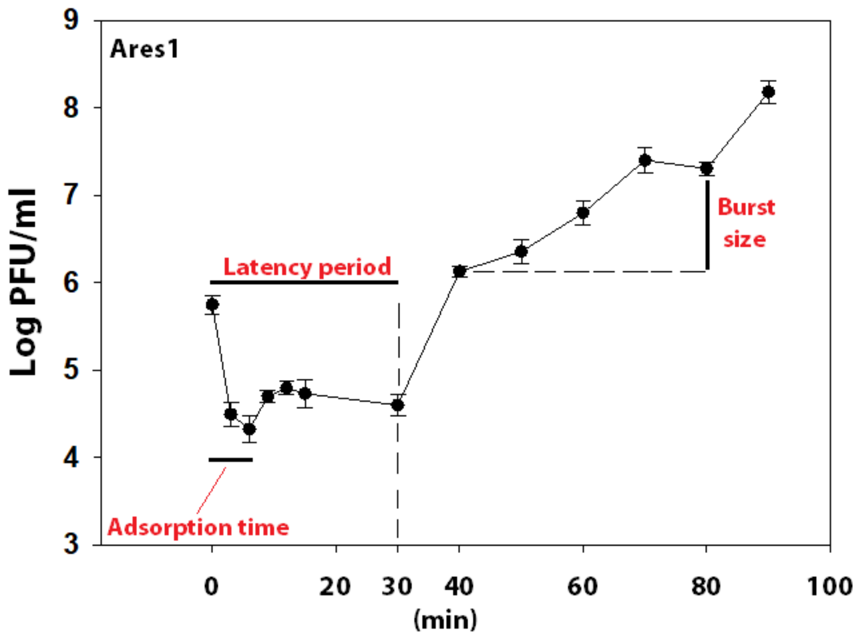

Figure 3: Two-step growth curve revealed biological characteristics of the bacteriophage Ares1.

Table 1. Open reading frames of bacteriophage Ares1.

Name	Length (nt)	Start	Finish	Orientation	Protein_id
hypothetical protein CDS	115	459	345	forward	AUR81185.1
hypothetical protein CDS	469	732	264	forward	AUR81208.1
hypothetical protein CDS	870	1160	291	forward	AUR81199.1
hypothetical protein CDS	1170	1472	303	forward	AUR81196.1
hypothetical protein CDS	1484	2008	525	forward	AUR81149.1
hypothetical protein CDS	1996	2280	285	forward	AUR81201.1
hypothetical protein CDS	2350	3138	789	forward	AUR81135.1
hypothetical protein CDS	3221	5170	1950	forward	AUR81110.1
hypothetical protein CDS	5246	5587	342	forward	AUR81186.1
hypothetical protein CDS	5719	6330	612	forward	AUR81141.1
hypothetical protein CDS	6333	7520	1188	forward	AUR81120.1
major capsid protein CDS	7606	8556	951	forward	AUR81128.1
hypothetical protein CDS	8632	8925	294	forward	AUR81198.1
hypothetical protein CDS	8937	9575	639	forward	AUR81140.1
hypothetical protein CDS	9572	10036	465	forward	AUR81158.1
hypothetical protein CDS	10033	10524	492	forward	AUR81155.1
hypothetical protein CDS	10578	11372	795	forward	AUR81134.1
hypothetical protein CDS	11462	11884	423	forward	AUR81169.1
hypothetical protein CDS	11962	12084	123	forward	AUR81224.1
tail length tape measure protein CDS	12092	16231	4140	forward	AUR81106.1
hypothetical protein CDS	16228	16614	387	forward	AUR81174.1
hypothetical protein CDS	16625	17614	990	forward	AUR81125.1
hypothetical protein CDS	17626	18504	879	forward	AUR81130.1
hypothetical protein CDS	18522	19820	1299	forward	AUR81118.1
hypothetical protein CDS	19823	20347	525	forward	AUR81150.1
hypothetical protein CDS	20348	20650	303	forward	AUR81197.1
hypothetical protein CDS	20647	20874	228	forward	AUR81214.1
hypothetical protein CDS	20864	21298	435	forward	AUR81167.1

hypothetical protein CDS	21301	21840	540	forward	AUR81148.1
AAA family ATPase CDS	21964	23184	1221	forward	AUR81119.1
hypothetical protein CDS	23576	25165	1590	forward	AUR81113.1
hypothetical protein CDS	25165	25758	594	forward	AUR81142.1
replicative DNA helicase CDS	25743	27191	1449	forward	AUR81114.1
hypothetical protein CDS	27263	27847	585	forward	AUR81143.1
hypothetical protein CDS	27840	28826	987	forward	AUR81126.1
hypothetical protein CDS	28837	29763	927	forward	AUR81129.1
putative DNA helicase CDS	29811	31238	1428	forward	AUR81116.1
hypothetical protein CDS	31334	31789	456	forward	AUR81160.1
hypothetical protein CDS	31794	32183	390	forward	AUR81173.1
RecA protein CDS	32196	33272	1077	forward	AUR81122.1
hypothetical protein CDS	33253	33666	414	forward	AUR81172.1
hypothetical protein CDS	33656	34651	996	forward	AUR81124.1
hypothetical protein CDS	34652	35212	561	forward	AUR81145.1
hypothetical protein CDS	35314	36288	975	forward	AUR81127.1
hypothetical protein CDS	36351	36641	291	forward	AUR81200.1
hypothetical protein CDS	36667	37410	744	forward	AUR81136.1
hypothetical protein CDS	37489	38220	732	forward	AUR81137.1
hypothetical protein CDS	38231	39040	810	forward	AUR81133.1
hypothetical protein CDS	39027	41003	1977	forward	AUR81109.1
hypothetical protein CDS	41015	41257	243	forward	AUR81210.1
hypothetical protein CDS	41358	41630	273	forward	AUR81204.1
hypothetical protein CDS	41669	41935	267	forward	AUR81207.1
hypothetical protein CDS	41925	42374	450	forward	AUR81164.1
pyruvate phosphate dikinase CDS	42409	44484	2076	forward	AUR81108.1
hypothetical protein CDS	44489	44971	483	forward	AUR81156.1
putative protein-tyrosine phosphatase CDS	44971	45519	549	forward	AUR81147.1
hypothetical protein CDS	45541	46554	1014	forward	AUR81123.1
hypothetical protein CDS	46565	47422	858	forward	AUR81131.1
DNA polymerase I CDS	47422	49779	2358	forward	AUR81107.1
portal protein CDS	49781	51646	1866	forward	AUR81111.1

hypothetical protein CDS	51643	52725	1083	forward	AUR81121.1
adenine DNA methyltransferase CDS	52731	53381	651	forward	AUR81139.1
hypothetical protein CDS	53470	53973	504	forward	AUR81154.1
hypothetical protein CDS	53982	54437	456	forward	AUR81161.1
putative DNA polymerase I CDS	54562	54930	369	forward	AUR81179.1
hypothetical protein CDS	54932	55126	195	forward	AUR81218.1
hypothetical protein CDS	55101	55556	456	forward	AUR81162.1
hypothetical protein CDS	55639	55782	144	forward	AUR81221.1
hypothetical protein CDS	55784	56233	450	forward	AUR81165.1
hypothetical protein CDS	56236	56520	285	forward	AUR81202.1
hypothetical protein CDS	56526	56831	306	forward	AUR81194.1
hypothetical protein CDS	56916	57224	309	forward	AUR81193.1
hypothetical protein CDS	57275	58627	1353	forward	AUR81117.1
hypothetical protein CDS	58593	58877	285	forward	AUR81203.1
hypothetical protein CDS	58886	59338	453	forward	AUR81163.1
hypothetical protein CDS	59331	59843	513	forward	AUR81151.1
hypothetical protein CDS	59853	60086	234	forward	AUR81213.1
hypothetical protein CDS	60153	60284	132	forward	AUR81223.1
hypothetical protein CDS	60755	61198	444	forward	AUR81166.1
hypothetical protein CDS	61195	61383	189	forward	AUR81220.1
hypothetical protein CDS	61370	61792	423	forward	AUR81170.1
hypothetical protein CDS	61789	62514	726	forward	AUR81138.1
hypothetical protein CDS	62501	63319	819	forward	AUR81132.1
hypothetical protein CDS	63322	63645	324	forward	AUR81191.1
hypothetical protein CDS	63780	64286	507	forward	AUR81153.1
hypothetical protein CDS	64349	64918	570	forward	AUR81144.1
hypothetical protein CDS	64955	65197	243	forward	AUR81211.1
hypothetical protein CDS	65317	65511	195	forward	AUR81219.1
hypothetical protein CDS	65526	65909	384	forward	AUR81176.1
hypothetical protein CDS	65960	66292	333	forward	AUR81190.1
hypothetical protein CDS	66305	66673	369	forward	AUR81180.1
hypothetical protein CDS	66673	67182	510	forward	AUR81152.1

hypothetical protein CDS	67179	67595	417	forward	AUR81171.1
hypothetical protein CDS	67651	68016	366	forward	AUR81182.1
hypothetical protein CDS	68037	68420	384	forward	AUR81177.1
hypothetical protein CDS	68420	68791	372	forward	AUR81178.1
hypothetical protein CDS	68846	69214	369	forward	AUR81181.1
hypothetical protein CDS	69269	69604	336	forward	AUR81189.1
hypothetical protein CDS	69660	70121	462	forward	AUR81159.1
hypothetical protein CDS	70108	70464	357	forward	AUR81184.1
hypothetical protein CDS	70600	70800	201	forward	AUR81215.1
hypothetical protein CDS	70864	71202	339	forward	AUR81187.1
hypothetical protein CDS	71205	71510	306	forward	AUR81195.1
hypothetical protein CDS	71514	71750	237	forward	AUR81212.1
hypothetical protein CDS	71879	72076	198	forward	AUR81217.1
hypothetical protein CDS	72079	72222	144	forward	AUR81222.1
hypothetical protein CDS	72340	72540	201	forward	AUR81216.1
hypothetical protein CDS	72590	72859	270	forward	AUR81205.1
hypothetical protein CDS	72859	73128	270	forward	AUR81206.1
hypothetical protein CDS	73125	74561	1437	forward	AUR81115.1
hypothetical protein CDS	74654	75214	561	forward	AUR81146.1
hypothetical protein CDS	75350	77209	1860	forward	AUR81112.1
hypothetical protein CDS	77374	77688	315	forward	AUR81192.1
hypothetical protein CDS	77776	78138	363	forward	AUR81183.1
hypothetical protein CDS	78188	78436	249	forward	AUR81209.1
hypothetical protein CDS	78438	78824	387	forward	AUR81175.1
hypothetical protein CDS	78821	79252	432	forward	AUR81168.1
hypothetical protein CDS	79323	79661	339	forward	AUR81188.1
hypothetical protein CDS	79674	80150	477	forward	AUR81157.1

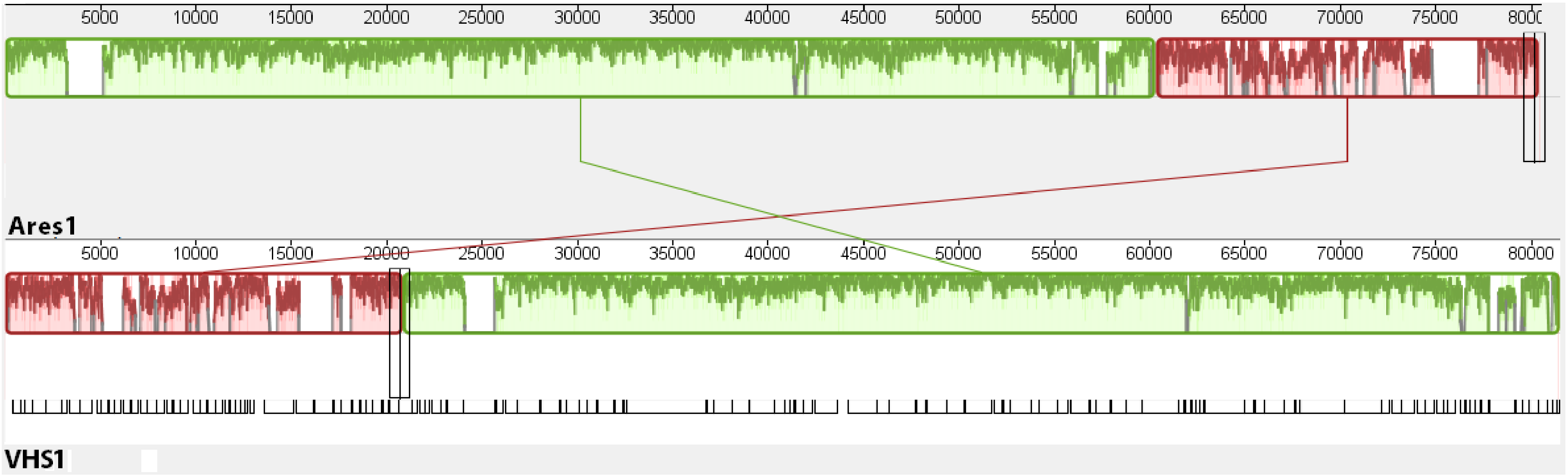

Figure 4: Whole genome multiple alignments with software MAUVE of Ares1 and phylogenetically close bacteriophage VHS1. Same color areas represent genomic synteny and diagrams inside areas represent percentage of synteny. White color represents unique genomic areas.
