# Supplementary material for: The Development of Bacteriophage Resistance in Vibrio alginolyticus Depends on a Complex Metabolic Adaptation Strategy
Source: Viruses. 2021 Apr 10;13(4):656. doi: 10.3390/v13040656 (PMC8069663; doi:10.3390/v13040656)
Supplement: Supplementary file 1 [file viruses-13-00656-s001.zip › supplementary 1050102/Table S1. Relative transcript abudances.pdf]

Skliros et al., 2020. Table S1. Average relative transcript abundances (±SE) of *Vibrio alginolyticus* strain V1 genes and its respective resistant strains to bacteriophages Aphrodite1 (VaAphrodite1), *φ*St2 (VaphiSt2) and Ares1 (VaAres1). **A** for receptors genes, **B** for genes involved in sugar transportation, **C** for genes involved in amino acid transporations, **D** for genes involved in major biochemical processes. Heat maps that appear in Figure 5 of main text correspond to the presented colored values here. *P* value represents t-tests between Control (wild type) and each resistant strain. Statistical significant is represented by bold *P* value. Mean primers' efficiency is also presented in table **E.**

A.

Relative expression levels of phage receptors												
	tolC	**±SE**	***P***	***ompF***	**±SE**	***P***	***btuB***	**±SE**	***P***	***lamB***	**±SE**	***P***
**Control**	0.2925	0.0139	na	0.0400	0.0039	na	0.1906	0.0142	na	5.7379	1.2186	na
**VaAphrodite1**	0.6417	0.0189	**0.0002**	0.0066	0.0002	**0.0023**	0.1164	0.0096	**0.0243**	3.6332	0.2073	0.237
**VaphiSt2**	0.5827	0.0215	**0.0007**	0.0061	0.0008	**0.0023**	0.1214	0.0156	0.0557	0.1063	0.0051	**0.0195**
**VaAres1**	0.6088	0.0581	**0.0124**	0.0414	0.0067	0.893	0.1775	0.0395	0.810	0.8058	0.0373	**0.0298**

B.

Relative transcript levels of genes involved in sugars transportation												
	***ptsG* 1**	**± SE**	***P***	***ptsG* 2**	**± SE**	***P***	***crr***	**± SE**	***P***	***celB***	**± SE**	***P***
**Control**	0.3775	0.0856	na	1.4519	0.1877	na	1.6949	0.4925	na	0.9120	0.0521	na
**VaAphrodite1**	1.3587	0.0258	**0.0036**	0.3239	0.0395	**0.0002**	0.2358	0.0428	**0.046**	0.1731	0.0516	**0.0004**
**Va*φ*St2**	0.2810	0.0994	0.428	0.6870	0.1674	**0.0040**	0.4834	0.1204	**0.047**	0.2966	0.0845	**0.0027**
**VaAres1**	0.2200	0.0184	0.216	0.6007	0.0645	0.889	0.5378	0.1469	0.0584	0.8508	0.1208	0.686
	rbsA	**± SE**	***P***	***treB***	**± SE**	***P***	***mtlA***	**± SE**	***P***	***fruA***	**± SE**	***P***
**Control**	1.5333	0.0949	na	1.0427	0.1180	na	0.7908	0.1704	na	0.3136	0.0331	na
**VaAphrodite1**	0.2333	0.0427	**0.002**	0.1652	0.1069	**0.0038**	0.6234	0.0468	0.482	0.1980	0.0143	**0.0230**
**Va*φ*St2**	0.5638	0.1894	0.055	0.8708	0.0177	0.253	0.0755	0.0490	**0.0301**	0.1924	0.0353	0.0764
**VaAres1**	0.5664	0.1793	**0.048**	0.6453	0.0358	0.0580	0.7221	0.0476	0.767	0.3474	0.0409	0.585

	ptsN	**± SE**	***P***	***ptsH***	**± SE**	***P***
**Control**	1.0454	0.0709	na	0.7503	0.0700	na
**VaAphrodite1**	0.1212	0.0257	**0.0002**	1.0928	0.1022	0.0604
**Va*φ*St2**	0.2781	0.1157	**0.0040**	0.2722	0.0915	**0.0160**
**VaAres1**	1.0167	0.1654	0.889	0.5863	0.0292	0.162

C.

Relative transcript levels of genes involved in amino acids transportation

	lysE	**± SE**	***P***	***tyrP***	**± SE**	***P***	***metl***	**± SE**	***P***	***metN***	**± SE**	***P***	***metQ***	**± SE**	***P***
**Control**	0.6267	0.1318	na	0.3810	0.1075	na	1.1441	0.1223	na	1.2661	0.0236	na	0.9026	0.1715	na
**VaAphrodite1**	0.0081	0.0031	**0.0186**	0.3319	0.0034	0.561	0.3791	0.0692	**0.0017**	0.1174	0.0375	**0.0000**	0.1265	0.0566	**0.00680**
**VaφSt2**	0.6481	0.0164	0.901	0.2690	0.0964	0.728	0.6151	0.0431	**0.0094**	0.4308	0.1407	**0.0085**	0.5843	0.1493	0.239
**VaAres1**	0.5162	0.0533	0.560	0.3636	0.0566	0.913	1.0528	0.1392	0.668	1.2330	0.0666	0.713	0.9403	0.0840	0.839
	***rhtB* 1**	**± SE**	***P***	***rhtB* 2**	**± SE**	***P***	***tcyP***	**± SE**	***P***	***hisP***	**± SE**	***P***	***azlC* 1**	**± SE**	***P***
**Control**	0.6020	0.0980	na	0.3086	0.0481	na	0.4799	0.0541	na	0.7633	0.0824	na	0.0363	0.0025	na
**VaAphrodite1**	0.2410	0.0582	0.0570	0.3459	0.0564	0.702	0.0723	0.0272	**0.0043**	0.3324	0.0329	**0.0145**	0.0512	0.0027	**0.0298**
**VaφSt2**	0.1928	0.0422	**0.0334**	0.3796	0.1071	0.647	0.0931	0.0022	**0.0053**	0.2552	0.1285	**0.0325**	0.0168	0.0006	**0.0035**
**VaAres1**	0.4428	0.0289	0.298	0.2417	0.0559	0.500	0.2172	0.0146	**0.0187**	0.5578	0.0522	0.160	0.0344	0.0039	0.747

	artP	**± SE**	***P***	***artL***	**± SE**	***P***	***livB***	**± SE**	***P***
**Control**	0.0152	0.0006	na	0.2992	0.0152	na	0.0021	0.0003	na
**VaAphrodite1**	0.0037	0.0016	**0.0022**	0.0695	0.0207	**0.0006**	0.0009	0.0001	**0.0346**
**VaφSt2**	0.0119	0.0019	0.236	0.4801	0.1178	0.279	0.0001	0.0000	**0.0044**
**VaAres1**	0.0117	0.0040	0.510	0.1239	0.0239	**0.0052**	0.0007	0.0001	**0.0155**
	***azlC* 2**	**± SE**	***P***	***livH***	**± SE**	***P***			
**Control**	0.0121	0.0010	na	0.0107	0.0015	na			
**VaAphrodite1**	0.0178	0.0024	0.152	0.0041	0.0010	**0.0382**			
**VaφSt2**	0.0164	0.0022	0.221	0.0017	0.0006	**0.0091**			
**VaAres1**	0.0064	0.0005	**0.0118**	0.0041	0.0002	**0.0211**			

D.

Relative transcript levels of genes involved in major biochemical processes of the cell															
	ald	**± SE**	***P***	***agxT***	**± SE**	***P***	***panD***	**± SE**	***P***	***lysA* 2**	**± SE**	***P***	***pykA***	**± SE**	***P***
**Control**	0.5209	0.2187	na	0.0365	0.0138	na	0.0005	0.0002	na	0.1652	0.0323	na	0.4902	0.1586	na
**VaAphrodite1**	1.3178	0.0204	**0.0415**	0.0275	0.0085	0.676	0.0001	0.0000	0.377	0.4523	0.0637	**0.0304**	0.6395	0.2170	0.674
**VaφSt2**	3.6661	0.5307	**0.0110**	0.0143	0.0026	0.266	0.0001	0.0000	0.330	0.5538	0.0256	**0.001**	0.0535	0.0051	0.127
**VaAres1**	0.2191	0.0509	0.334	0.0420	0.0054	0.776	0.0003	0.0001	0.286	1.1338	0.1234	**0.003**	1.4136	0.3587	0.087
	***lysA* 1**	**± SE**	***P***	***gltA***	**± SE**	***P***	***murE***	**± SE**	***P***	***ppc***	**± SE**	***P***	***mdh* 1**	**± SE**	***P***
**Control**	0.0367	0.0034	na	5.0889	0.6599	na	0.2192	0.0288	na	0.3602	0.0903	na	5.4497	0.0115	na
**VaAphrodite1**	0.0183	0.0015	0.502	6.1671	0.7864	0.439	0.1487	0.0122	0.140	0.1440	0.0282	0.1620	1.6533	0.1745	**0.000**
**VaφSt2**	0.0177	0.0030	0.986	0.7964	0.2812	**0.0081**	0.1143	0.0058	**0.0434**	0.1606	0.0317	0.1000	0.4048	0.0654	**0.000**
**VaAres1**	0.0488	0.0126	0.388	4.5747	0.6796	0.681	0.1890	0.0157	0.494	0.2558	0.0164	0.4060	5.5623	0.5020	0.864

	frd	**± SE**	***P***	***pykF***	**± SE**	***P***	***pckA***	**± SE**	***P***
**Control**	2.8835	0.1460	na	1.0785	0.1462	na	7.2277	0.7000	na
**VaAphrodite1**	14.1752	1.5394	**0.0039**	1.3193	0.1288	0.370	13.2575	0.6990	**0.0076**
**VaφSt2**	0.1669	0.0165	**0.0001**	1.4789	0.1546	0.199	0.4540	0.1260	**0.0014**
**VaAres1**	1.7651	0.0954	**0.0063**	0.9863	0.0656	0.663	5.5833	0.5228	0.199
	***mdh* 2**	**± SE**	***P***	***mdh* 3**	**± SE**	***P***			
**Control**	0.4060	0.1487	na	0.9289	0.2133	na			
**VaAphrodite1**	0.5390	0.1245	0.605	1.0160	0.1629	0.804			
**VaφSt2**	0.6721	0.0793	0.267	0.8036	0.0051	0.657			
**VaAres1**	0.6324	0.2170	0.521	0.6264	0.0339	0.317			

Upregulation **Downregulation**

E.

Gene studied	Mean of primer's efficiency	Gene studied	Mean of primer's efficiency	Gene studied	Mean of primer's efficiency
tolC	1.969	*tcyP*	1.981	*pykF*	1.988
ompF	1.988	*hisP*	1.960	*pckA*	1.993
btuB	1.988	*azlC* 1	1.984		
lamB	1.973	*azlC* 2	1.981		
ptsG 1	1.913	*artP*	1.994		
ptsG 2	1.989	*artL*	1.985		
crr	1.967	*liVB*	1.832		
celB	1.981	*livH*	1.990		
rbsA	1.977	*ald*	1.980		
treB	1.994	*agxT*	1.985		
mtlA	1.991	*panD*	1.989		
fruA	1.980	*lysA 1*	1.991		
ptsN	1.990	*lysA 2*	1.982		
ptsH	1.970	*pykA*	1.988		
lysE	1.952	*gltA*	1.994		
tyrP	1.993	*murE*	1.990		
metL	1.989	*ppc*	1.946		
metN	1.969	*mdh 1*	1.992		
metQ	1.981	*mdh 2*	1.976		
rhtB 1	1.956	*mdh 3*	1.992		
rhtB 2	1.971	*frd*	1.991		
