# Supplementary material for: The Development of Bacteriophage Resistance in Vibrio alginolyticus Depends on a Complex Metabolic Adaptation Strategy
Source: Viruses. 2021 Apr 10;13(4):656. doi: 10.3390/v13040656 (PMC8069663; doi:10.3390/v13040656)
Supplement: Supplementary file 1 [file viruses-13-00656-s001.zip › supplementary 1050102/Table S2. Primers list of qPCR platform.pdf]

Skliros et al., 2020. Table S2. Primers list of qPCR platform. Primers list, which were used to study relative transcript levels of *Vibrio alginolyticus* strain V1 genes involved in sugar transportation (A), amino acids transportation (B), primers for bacteriophage DNA detection (C) and major biochemical processes and bacterial phage receptors (D).

A.

**Primers amplifying genes relative to sugars transportation**	
Gene	Primers (R'/F')
cellB	Reverse-5'-GGATCGCAATACCGTTTCCG-3'
	Forward-5'-GAGCTGTTCGAATGCATGCT-3'
crr	Reverse-5'-CTCATTGCGCGAACATGGG-3'
	Forward-5'-GGCGCAGTCGAAAAGCATAT-3'
fruA	Reverse-5'-ACGAGAAGTGTCGACGCTAG-3'
	Forward-5'-GACGGAAGCTGACGTTGAAC-3'
ptsG 1	Reverse-5'-GGCAGCACCCACACCTAATA-3'
	Forward-5'-TGGTAAGGCTCTGATGCTTCC-3'
ptsG 2	Reverse-5'-ATCTGGCACGTCTTCGATGT-3'
	Forward-5'-TGACGCTGGTGCTATCGAAA-3'
ptsN	Reverse-5'-TGATGTCTGCGTCGAATGCT-3'
	Forward-5'-GAAAACGGCCTTCACACTCG-3'
rbsA	Reverse-5'-AGTGAGTTCATCGCGCTCTG-3'
	Forward-5'-CAGACTCTGAACTGACCGCT-3'
ptsH	Reverse-5'-CGTCGCTCTGAGGGTAAGAC-3'
	Forward-5'-TGGGGGCATAGAAAACCTGC-3'
treB	Reverse-5'-ACGCAAGCGAGTTAAACAGT-3'
	Forward-5'-GCGTCTTATTGAGCTGGTCG-3'
mtlA	Reverse-5'-TTGGTAGCCATCCAGTTGGG-3'
	Forward-5'-CCAAACATCGGCGCGTTTAT-3'

B.

**Primers amplifying genes relative to amino acids transportation**	
Gene	Primers (R'/F')
metQ	Reverse-5'-TGTTTTCAAGTAGGCCGCCT-3'
	Forward-5'-GGCTTTGCTGTGGGTATTCC-3'
metL	Reverse-5'-GTGCATGTGGTCCAACTGTC-3'
	Forward-5'-GGCTTAAGCGCTTGTGGAGA-3'
metN	Reverse-5'-GTCAACAAAGCGAGCAAGGT-3'
	Forward-5'-CACAAGGCGAGCATGTGTTT-3'
azlC 1	Reverse-5'-TCAATTGCGAATGAGCCTGC-3'
	Forward-5'-CACTTGCGATGATGCCTCTG-3'
azlC 2	Reverse-5'-TTGGGTGAATCCGTTCCAGA-3'
	Forward-5'-ACATCTTCAACGCTCCTCCA-3'
artP	Reverse-5'-TAGTGCATTCGCCAGATCCA-3'
	Forward-5'-CGCACCGTTCGAATACATGG-3'
artL	Reverse-5'-TCATATGCGGCCATAGGTTGT-3'
	Forward-5'-AAGCAAGGTTTAGCGCTTCG-3'
***rhtB* 1**	Reverse-5'-GCTGCGCCAATCATTTTTACG-3'
	Forward-5'-CGGTTGGTATCTCGGCGATT-3'
***rhtB* 2**	Reverse-5'-AAGGTTCGAAGTAGCCCCAG
	Forward-5'-ACGTGGCCACAAGTACAGTT-3'
tcyP	Reverse-5'-GCCACTGCCTACAATGCCTA-3'
	Forward-5'-ACTCATTCACGGTGAAGGCA-3'
hisP	Reverse-5'-CGGTAGGCGTTTCTAGCAAG-3'
	Forward-5'-ATCATTGGCTCTTCCGGCTC-3'

C.

**Gene Target and amplification size**	**Sequences**
Capsid Protein/ 70bp, Vibrio phage Aphrodite1	Forward-5’-GCGATCCGTGGTGATACAAC-3’<br>Reverse-5’-TAGTGCGCTTCAACCAACCA-3’
MCP, Major Capsid Protein/ 70bp, Vibrio phage phiSt2	Forward-5’-CGACCAATACGCAGTGAACG-3’<br>Reverse-5’-CACACCTGCGTCCATTTCAG-3’
MCP, Major Capsid Protein/ 70bp, Vibrio phage Ares1	Forward-5’-GCGATCCGTGGTGATACAAC-3’<br>Reverse-5’-TAGTGCGCTTCAACCAACCA-3’

D.

Primers amplifying genes responsible for major biochemical processes and bacterial phage receptors			
***mdh* 2**	Forward-5'-TTCAGGGTACTGCAGCTGTT-3'	***tolC***	Reverse-5'-AGTCGTAACGAGCGTTCGAT-3'
	Reverse-5'-GAAAGCTGAGTTCCTGCTGC-3'		Forward-5'-GTTGATGTACTGGACGCGAC-3'
***mdh* 1**	Forward-5'-GCATGTCGCTTTGGTCTGTC-3'	***ompF***	Forward-5'-AATAGCACCCGTTAGCAGCT-3'
	Reverse-5'-CGTAAGCGCACTCAACTACG-3'		Reverse-5'-TGCGCTTGGTTACAACGAAG-3'
***mdh* 3**	Forward-5'-TTGAAGTTAAGCACCGCACG-3'	***livH***	Reverse-5'-CCGAAATGGCCCAACTGATG-3'
	Reverse-5'-CCGCCAAAAGTGTCTGCAAT-3'		Forward-5'-AACCAGCAAGTAGCCCAGAG-3'
Ppc	Forward-5'-AGAACTCGAACTCGGCAAGT-3'	***livB***	Reverse-5'-CCTTTAATTCGAGCCGCTGG-3'
	Reverse-5'-TTCTACTCCGCCCTTAGGGT-3'		Forward-5'-TTCCGCTTGCGGGTTACTTA-3'
ald	Forward-5'-TATGGGTCGTTTGCCACTGT-3'	***lamB***	Reverse-5'-CAGAAGCTAGAGCCAGCAGA-3'
	Reverse-5'-CGCACCAGCTTGGATAGACA-3'		Forward-5'-ACGACGGCGAAGAAAACAAA-3'
agxT	Forward-5'-TTTGTGCATGCGGAAACCTC-3'	***btuB***	Reverse-5'-GCTAACCGACCAGTCCACTT-3'
	Reverse-5'-GCTGTTTCGCCAGCTTACTC-3'		Forward-5'-GCGTCGCGCTAAAGAAATGT-3'
panD	Forward-5'-AGTGCCACCACGTATGTTGA-3'	***murE***	Forward-5'-TTGACCATAAAGCGGCGGTA-3'
	Reverse-5'-GCCGTACATCTCTTCTGCGA-3'		Reverse-5'-TTCGCAGCTTGTTGCAAGTT-3'
***lysA* 1**	Forward-5'-TTCCCGCAAGTGTCACTCAA-3'	***pckA***	Forward-5'-ATGTTTATTCGCCCGACGGA-3'
	Reverse-5'-AGTGGCGTTCAACATCAAGC-3'		Reverse-5'-TTGCGCCATTCATTACCACG-3'
fdr	Forward-5'-ACAGAGCGTGATGACGTGAA-3'	***pykF***	Forward-5'-GGACGGTACTGACGCAGTAA-3'
	Reverse-5'-TACTTGGTGCTGACTCTGGC-3'		Reverse-5'-AGTTACCGCTTCAACAGGGT-3’
gltA	Forward-5'-GACCCAGGTTTTCTTGCCAC-3'		
	Reverse-5'-GAATACCTTTGCCGCCATCG-3'		
pykA	Forward-5'-GCATTACGCTCGACGCTTAG-3'		
	Reverse-5'-TGCTCGTTCGACTTTAGCCA-3'		
lysA2	Forward-5'-CTTGGGTAAAGATCGCGCAC-3'		
	Reverse-5'-ATAGGCACCTGCTGAACGAA-3'		
