# Supplementary material for: The Development of Bacteriophage Resistance in Vibrio alginolyticus Depends on a Complex Metabolic Adaptation Strategy
Source: Viruses. 2021 Apr 10;13(4):656. doi: 10.3390/v13040656 (PMC8069663; doi:10.3390/v13040656)
Supplement: Supplementary file 1 [file viruses-13-00656-s001.zip › supplementary 1050102/Table S3.pdf]

Skliros et al., 2021. Supplemental Table S3. List of polymorphisms that were localized in the 3 respective resistant strains. VaAphrodite1 (A, B, C) VaphiSt2 (A, B) and VaAres1 (A, B, C) for each Contig of the reference strain *Vibrio alginolyticus* strain V1 separately. Variant Frequency and Variant P-value are also provided.

Contig 1

Variant	Name	Minimum	Maximum	Length	Change	Coverage	Polymorphism Type	Variant Frequency	Variant P-Value (approximate)	Amino Acid Change	product	Protein Effect	protein_id	CDS
Variants: VaAphrodite_B_FDSW210055058-2r	A	141927	141927	1	G -> A	62	SNP (transition)	25.80%	2.70E-42	S -> F	hypothetical protein	Substitution	KLI74194.1	hypothetical protein CDS
Variants: VaAres1_A_FDSW210055060-2r	A	326430	326430	1	C -> A	83	SNP (transversion)	28.90%	4.30E-19		DNA repair protein	None	KLI74350.1	DNA repair protein CDS
Variants: VaphiVaphiSt2_B_A_FDSW210055054-2r	A	326430	326430	1	C -> A	81	SNP (transversion)	25.90%	2.10E-08		DNA repair protein	None	KLI74350.1	DNA repair protein CDS
Variants: VaAres1_A_FDSW210055060-2r	A	326447	326447	1	T -> A	79	SNP (transversion)	31.60%	1.10E-09	I -> N	DNA repair protein	Substitution	KLI74350.1	DNA repair protein CDS
Variants: VaAres1_B_FDSW210055061-2r	A	326447	326447	1	T -> A	112	SNP (transversion)	25.00%	3.90E-08	I -> N	DNA repair protein	Substitution	KLI74350.1	DNA repair protein CDS
Variants: VaphiVaphiSt2_B_A_FDSW210055054-2r	A	326447	326447	1	T -> A	80	SNP (transversion)	26.30%	2.80E-10	I -> N	DNA repair protein	Substitution	KLI74350.1	DNA repair protein CDS
Variants: VaAres1_B_FDSW210055061-2r	C	326452	326452	1	A -> C	112	SNP (transversion)	25.00%	2.50E-10		DNA repair protein	None	KLI74350.1	DNA repair protein CDS
Variants: VaAres1_A_FDSW210055060-2r	C	326453	326453	1	G -> C	85	SNP (transversion)	30.60%	8.00E-12	R -> T	DNA repair protein	Substitution	KLI74350.1	DNA repair protein CDS
Variants: VaphiVaphiSt2_B_A_FDSW210055054-2r	C	326455	326455	1	G -> C	84	SNP (transversion)	29.80%	2.70E-13	A -> P	DNA repair protein	Substitution	KLI74350.1	DNA repair protein CDS
Variants: VaAres1_A_FDSW210055060-2r	C	326458	326458	1	A -> C	89	SNP (transversion)	33.70%	2.80E-40	N -> H	DNA repair protein	Substitution	KLI74350.1	DNA repair protein CDS
Variants: VaphiVaphiSt2_B_A_FDSW210055054-2r	C	326458	326458	1	A -> C	83	SNP (transversion)	25.30%	1.40E-23	N -> H	DNA repair protein	Substitution	KLI74350.1	DNA repair protein CDS
Variants: VaphiVaphiSt2_B_A_FDSW210055054-2r	CT	485766	485767	2	AC -> CT	194 -> 195	Substitution	25.1% -> 25.3%	1.00E-128					
Variants: VaAphrodite_B_FDSW210055058-2r	A	485769	485769	1	T -> A	256	SNP (transversion)	28.10%	5.20E-152					
Variants: VaAres1_A_FDSW210055060-2r	A	485769	485769	1	T -> A	190	SNP (transversion)	27.40%	5.30E-94					
Variants: VaAres1_B_FDSW210055061-2r	A	485769	485769	1	T -> A	201	SNP (transversion)	33.30%	8.00E-161					
Variants: VaAres1_C_FDSW210055062-1r	A	485769	485769	1	T -> A	269	SNP (transversion)	29.70%	5.00E-155					
Variants: VaphiVaphiSt2_B_A_FDSW210055054-2r	A	485769	485769	1	T -> A	193	SNP (transversion)	34.20%	2.40E-159					
Variants: VaphiSt2_B_FDSW210055056-1r	A	485769	485769	1	T -> A	397	SNP (transversion)	33.50%	7.9E-318					
Variants: VaAphrodite_B_FDSW210055058-2r	T	485773	485773	1	A -> T	262	SNP (transversion)	32.40%	7.30E-194					
Variants: VaAphrodite_C_FDSW210055059-1r	T	485773	485773	1	A -> T	317	SNP (transversion)	26.50%	2.80E-191					
Variants: VaAres1_A_FDSW210055060-2r	T	485773	485773	1	A -> T	189	SNP (transversion)	25.90%	3.20E-92					
Variants: VaAres1_B_FDSW210055061-2r	T	485773	485773	1	A -> T	214	SNP (transversion)	34.60%	7.00E-179					
Variants: VaAres1_C_FDSW210055062-1r	T	485773	485773	1	A -> T	272	SNP (transversion)	32.70%	8.60E-168					
Variants: VaphiVaphiSt2_B_A_FDSW210055054-2r	T	485773	485773	1	A -> T	208	SNP (transversion)	34.10%	3.30E-171					
Variants: VaphiSt2_B_FDSW210055056-1r	T	485773	485773	1	A -> T	401	SNP (transversion)	35.70%	0					
Variants: VaAphrodite_C_FDSW210055059-1r	A	485788	485788	1	C -> A	421	SNP (transversion)	25.70%	1.90E-254					
Variants: VaAres1_B_FDSW210055061-2r	A	485788	485788	1	C -> A	252	SNP (transversion)	26.60%	2.00E-166					
Variants: VaAres1_C_FDSW210055062-1r	A	485788	485788	1	C -> A	364	SNP (transversion)	31.30%	2.70E-257					
Variants: VaphiVaphiSt2_B_A_FDSW210055054-2r	A	485788	485788	1	C -> A	263	SNP (transversion)	28.10%	3.40E-156					

Variants: VaphiSt2_B_FDSW210055056-1r	A	485788	485788	1	C -> A	511	SNP (transversion)	25.20%	1.20E-289
Variants: VaAphrodite_B_FDSW210055058-2r	C	485790	485790	1	T -> C	364	SNP (transition)	26.90%	1.20E-223
Variants: VaAphrodite_C_FDSW210055059-1r	C	485790	485790	1	T -> C	425	SNP (transition)	27.30%	9.20E-277
Variants: VaAres1_B_FDSW210055061-2r	C	485790	485790	1	T -> C	266	SNP (transition)	29.30%	2.50E-197
Variants: VaAres1_C_FDSW210055062-1r	C	485790	485790	1	T -> C	380	SNP (transition)	33.20%	1.60E-300
Variants: VaphiVaphiSt2_B_A_FDSW210055054-2r	C	485790	485790	1	T -> C	262	SNP (transition)	27.10%	7.60E-170
Variants: VaphiSt2_B_FDSW210055056-1r	C	485790	485790	1	T -> C	522	SNP (transition)	27.00%	0
Variants: VaAphrodite_B_FDSW210055058-2r	G	485906	485906	1	A -> G	409	SNP (transition)	39.90%	0
Variants: VaAphrodite_C_FDSW210055059-1r	G	485906	485906	1	A -> G	563	SNP (transition)	35.50%	0
Variants: VaAres1_A_FDSW210055060-2r	G	485906	485906	1	A -> G	309	SNP (transition)	30.10%	2.60E-134
Variants: VaAres1_B_FDSW210055061-2r	G	485906	485906	1	A -> G	348	SNP (transition)	37.90%	1.60E-271
Variants: VaAres1_C_FDSW210055062-1r	G	485906	485906	1	A -> G	464	SNP (transition)	42.90%	0
Variants: VaphiVaphiSt2_B_A_FDSW210055054-2r	G	485906	485906	1	A -> G	282	SNP (transition)	38.70%	8.90E-215
Variants: VaphiSt2_B_FDSW210055056-1r	G	485906	485906	1	A -> G	534	SNP (transition)	37.60%	0
Variants: VaAphrodite_B_FDSW210055058-2r	A	485920	485920	1	G -> A	346	SNP (transition)	38.40%	8.50E-288
Variants: VaAphrodite_C_FDSW210055059-1r	A	485920	485920	1	G -> A	490	SNP (transition)	38.60%	0
Variants: VaAres1_A_FDSW210055060-2r	A	485920	485920	1	G -> A	287	SNP (transition)	34.10%	1.90E-128
Variants: VaAres1_B_FDSW210055061-2r	A	485920	485920	1	G -> A	286	SNP (transition)	31.10%	2.40E-174
Variants: VaAres1_C_FDSW210055062-1r	A	485920	485920	1	G -> A	396	SNP (transition)	41.90%	0
Variants: VaphiVaphiSt2_B_A_FDSW210055054-2r	A	485920	485920	1	G -> A	211	SNP (transition)	46.40%	2.10E-203
Variants: VaphiSt2_B_FDSW210055056-1r	A	485920	485920	1	G -> A	423	SNP (transition)	37.10%	9.7E-321

Contig 2

Variant	Name	Minimum	Maximum	Length	Change	Coverage	Polymorphism Type	Variant Frequency	Variant P-Value (approximate)	Amino Acid Change	product	Protein Effect	protein_id	CDS
Variants: VaphiVaphiSt2_B_A_FDSW210055054-2r	A	17346	17346	1	C -> A	90	SNP (transversion)	26.70%	8.50E-14		MerR family transcriptional regulator	None	KLI73845.1	MerR family transcriptional regulator CDS
Variants: VaAres1_B_FDSW210055061-2r	A	17362	17362	1	C -> A	105	SNP (transversion)	25.70%	1.50E-12	R -> I	MerR family transcriptional regulator	Substitution	KLI73845.1	MerR family transcriptional regulator CDS
Variants: VaAphrodite_C_FDSW210055059-1r	C	180730	180730	1	A -> C	166	SNP (transversion)	100.00%	0	V -> G	transcriptional regulator	Substitution	KLI73980.1	transcriptional regulator CDS
Variants: VaphiVaphiSt2_B_A_FDSW210055054-2r	C	180730	180730	1	A -> C	88	SNP (transversion)	98.90%	2.80E-303	V -> G	transcriptional regulator	Substitution	KLI73980.1	transcriptional regulator CDS
Variants: VaphiSt2_B_FDSW210055056-1r	C	180730	180730	1	A -> C	183	SNP (transversion)	99.50%	0	V -> G	transcriptional regulator	Substitution	KLI73980.1	transcriptional regulator CDS
Variants: VaAphrodite_C_FDSW210055059-1r	TCCTAGAAAT	182647	182646	0	#NAME?	169 -> 170	Insertion	37.6% -> 37.9%	7.60E-161					
Variants: VaAres1_A_FDSW210055060-2r	T	188712	188712	1	G -> T	80	SNP (transversion)	30.00%	7.70E-09	S -> Y	membrane protein	Substitution	KLI73986.1	membrane protein CDS
Variants: VaphiVaphiSt2_B_A_FDSW210055054-2r	TATA	223385	223389	5	GGCGC -> TATA	50 -> 51	Deletion	100.00%	1.00E-80					
Variants: VaphiVaphiSt2_B_A_FDSW210055054-2r	A	223397	223397	1	G -> A	49	SNP (transition)	100.00%	2.50E-167					
Variants: VaphiVaphiSt2_B_A_FDSW210055054-2r	TGT	223401	223403	3	ACG -> TGT	49	Substitution	100.00%	2.50E-167					
Variants: VaphiVaphiSt2_B_A_FDSW210055054-2r		223406	223408	3	#NAME?	48	Deletion	100.00%	1.60E-77					
Variants: VaphiVaphiSt2_B_A_FDSW210055054-2r	CGC	223411	223413	3	ATG -> CGC	48	Substitution	100.00%	6.30E-164					
Variants: VaphiVaphiSt2_B_A_F	CG	223418	223419	2	TC -> CG	41 -> 42	Substitution	100.00%	4.00E-140					

DSW210055 054-2r															
Variants: VaphiVaphi St2_B_A_F DSW210055 054-2r	C	223421	223421	1	T -> C		41	SNP (transition)	100.00%	3.20E-144					
Variants: VaphiVaphi St2_B_A_F DSW210055 054-2r	A	223423	223423	1	T -> A		41	SNP (transversion)	100.00%	3.20E-144					
Variants: VaphiVaphi St2_B_A_F DSW210055 054-2r	TGT	223425	223427	3	AAA -> TGT		40	Substitution	100.00%	1.00E-128					
Variants: VaphiVaphi St2_B_A_F DSW210055 054-2r	GAG	223433	223435	3	CTC -> GAG	33 -> 36		Substitution	100.00%	3.20E-116					
Variants: VaphiVaphi St2_B_A_F DSW210055 054-2r	A	223437	223437	1	T -> A		31	SNP (transversion)	100.00%	4.00E-106					
Variants: VaphiVaphi St2_B_A_F DSW210055 054-2r	GCTCCT	223439	223444	6	TGAGGG -> GCTCCT	24 -> 27		Substitution	100.00%	2.50E-82					
Variants: VaphiVaphi St2_B_A_F DSW210055 054-2r	TTCCC	223446	223450	5	CAATT -> TTCCC	21 -> 23		Substitution	100.00%	6.30E-69					
Variants: VaphiVaphi St2_B_A_F DSW210055 054-2r	T	223452	223452	1	A -> T		21	SNP (transversion)	100.00%	4.00E-72					
Variants: VaphiVaphi St2_B_A_F DSW210055 054-2r	GCAGAT	223453	223458	5	ATGAAG -> GCAGAT	20 -> 21		Substitution	100.00%	1.00E-60	MK -> AD	regulator	Start Codon Loss	KLI74019.1	regulator CDS
Variants: VaphiVaphi St2_B_A_F DSW210055 054-2r	T	223460	223460	1	A -> T		19	SNP (transversion)	100.00%	7.90E-56	Y -> F	regulator	Substitution	KLI74019.1	regulator CDS
Variants: VaphiVaphi St2_B_A_F DSW210055 054-2r	AAATTT	223462	223467	5	CACGAA -> AAATTT	14 -> 18		Substitution	100.00%	1.00E-42	HE -> KF	regulator	Substitution	KLI74019.1	regulator CDS
Variants: VaphiVaphi St2_B_A_F DSW210055 054-2r	A	223469	223469	1	T -> A		14	SNP (transversion)	100.00%	1.00E-42	M -> K	regulator	Substitution	KLI74019.1	regulator CDS
Variants: VaphiVaphi St2_B_A_F	GGCCTT	223471	223476	6	ACTAAA -> GGCCTT	12 -> 14		Substitution	100.00%	4.00E-39	TK -> GL	regulator	Substitution	KLI74019.1	regulator CDS

DSW210055 054-2r														
Variants: VaphiVaphi St2_B_A_F DSW210055 054-2r	CTGTG	223478	223482	5	ACTAT -> CTGTG	8 -> 10	Substitution	100.00%	1.60E-25	NY -> TV	regulator	Substitution	KLI74019.1	regulator CDS
Variants: VaphiVaphi St2_B_A_F DSW210055 054-2r	GCA	223484	223486	3	TTT -> GCA	8	Substitution	100.00%	4.00E-23	IF -> SI	regulator	Substitution	KLI74019.1	regulator CDS
Variants: VaphiVaphi St2_B_A_F DSW210055 054-2r	AAACAT	223489	223494	5	CGTGAA -> AAACAT	3 -> 5	Substitution	100.00%	2.50E-07	RE -> KH	regulator	Substitution	KLI74019.1	regulator CDS
Variants: VaphiVaphi St2_B_A_F DSW210055 054-2r	CACCAAT G	232698	232705	8	TGATTGCA -> CACCAAT G	4 -> 8	Substitution	75.0% -> 87.5%	0.000001	VIA -> APM	antirepressor	Substitution	KLI74031.1	antirepressor CDS
Variants: VaphiVaphi St2_B_A_F DSW210055 054-2r	TAATCTTC T	232707	232715	9	GTCCACA GG -> TAATCTTC T	8 -> 11	Substitution	87.5% -> 90.9%	5.00E-18		antirepressor	Truncation	KLI74031.1	antirepressor CDS
Variants: VaphiVaphi St2_B_A_F DSW210055 054-2r	T	232721	232721	1	C -> T	14	SNP (transition)	92.90%	3.50E-41		antirepressor	None	KLI74031.1	antirepressor CDS
Variants: VaphiVaphi St2_B_A_F DSW210055 054-2r		232724	232727	4	#NAME?	14	Deletion	92.90%	2.20E-20		antirepressor	Frame Shift	KLI74031.1	antirepressor CDS
Variants: VaphiVaphi St2_B_A_F DSW210055 054-2r	T	232731	232731	1	G -> T	19	SNP (transversion)	94.70%	4.80E-57	C -> F	antirepressor	Substitution	KLI74031.1	antirepressor CDS
Variants: VaAres1_A_ FDSW21005 5060-2r	T	254034	254034	1	G -> T	85	SNP (transversion)	29.40%	2.20E-22		trans-2-enoyl-CoA reductase	Truncation	KLI74048.1	trans-2-enoyl-CoA reductase CDS
Variants: VaAres1_A_ FDSW21005 5060-2r	T	254042	254042	1	G -> T	89	SNP (transversion)	27.00%	4.40E-16	W -> C	trans-2-enoyl-CoA reductase	Substitution	KLI74048.1	trans-2-enoyl-CoA reductase CDS
Variants: VaphiVaphi St2_B_A_F DSW210055 054-2r	A	254057	254057	1	C -> A	83	SNP (transversion)	25.30%	3.30E-08		trans-2-enoyl-CoA reductase	None	KLI74048.1	trans-2-enoyl-CoA reductase CDS

Contig 3

Variant	Name	Minimum	Maximum	Length	Change	Coverage	Polymorphism Type	Variant Frequency	Variant P-Value (approximate)	Amino Acid Change	product	Protein Effect	protein_id	CDS
Variants: VaAphrodite_A_FDSW210055057-2r	TT	20571	20570	0	(TT)2 -> (TT)3	152	Insertion (tandem repeat)	27.60%	3.40E-93		glyceraldehyde-3-phosphate dehydrogenase	Frame Shift	KLI73546.1	glyceraldehyde-3-phosphate dehydrogenase CDS
Variants: VaAres1_B_FDSW210055061-2r	A	291743	291743	1	C -> A	104	SNP (transversion)	25.00%	4.30E-14		chemotaxis protein CheY	None	KLI73738.1	chemotaxis protein CheY CDS

Contig 4

Variant	Name	Minimum	Maximum	Length	Change	Coverage	Polymorphism Type	Variant Frequency	Variant P-Value (approximate)	Amino Acid Change	product	Protein Effect	protein_id	CDS
Variants: VaAphrodite_A_FDSW210055057-2r		94	94	1	#NAME?	85	Deletion	34.10%	6.50E-12					
Variants: VaAres1_A_FDSW210055060-2r		94	94	1	#NAME?	93	Deletion	40.90%	7.10E-36					
Variants: VaAres1_C_FDSW210055062-1r		94	94	1	#NAME?	123	Deletion	39.00%	1.40E-21					
Variants: VaAphrodite_A_FDSW210055057-2r	A	98	98	1	T -> A	84	SNP (transversion)	34.50%	7.90E-51					
Variants: VaAres1_A_FDSW210055060-2r	A	98	98	1	T -> A	85	SNP (transversion)	41.20%	2.80E-99					
Variants: VaAres1_C_FDSW210055062-1r	A	98	98	1	T -> A	119	SNP (transversion)	38.70%	2.50E-73					
Variants: VaphiVaphiSt2_B_A_FDSW210055054-2r	A	98	98	1	T -> A	78	SNP (transversion)	30.80%	9.00E-34					
Variants: VaAphrodite_A_FDSW210055057-2r	AGC	102	104	3	GAG -> AGC	83	Substitution	30.10%	2.90E-42					
Variants: VaAres1_A_FDSW210055060-2r	AGC	102	104	3	GAG -> AGC	80 -> 81	Substitution	33.8% -> 34.6%	1.10E-68					
Variants: VaAres1_C_FDSW210055062-1r	AGC	102	104	3	GAG -> AGC	123 -> 124	Substitution	29.8% -> 30.1%	2.60E-65					
Variants: VaphiVaphiSt2_B_A_FDSW210055054-2r	AGC	102	104	3	GAG -> AGC	78 -> 79	Substitution	26.9% -> 27.8%	5.50E-30					
Variants: VaAphrodite_A_FDSW210055057-2r	T	107	107	1	C -> T	79	SNP (transition)	27.80%	2.90E-45					
Variants: VaAres1_A_FDSW210055060-2r	T	107	107	1	C -> T	73	SNP (transition)	30.10%	6.30E-55					
Variants: VaAres1_A_FDSW210055060-2r	T	95321	95321	1	G -> T	82	SNP (transversion)	26.80%	8.10E-15	T -> N	membrane protein	Substitution	KLI73359.1	membrane protein CDS
Variants: VaAres1_A_FDSW210055060-2r	C	96286	96286	1	A -> C	80	SNP (transversion)	27.50%	4.60E-15	N -> H	membrane protein	Substitution	KLI73360.1	membrane protein CDS
Variants: VaphiVaphiSt2_B_A_FDSW210055054-2r	T	192079	192079	1	G -> T	94	SNP (transversion)	26.60%	1.50E-16	T -> N	hypothetical protein	Substitution	KLI73433.1	hypothetical protein CDS
Variants: VaphiVaphiSt2_B_A_FDSW210055054-2r	T	192096	192096	1	A -> T	95	SNP (transversion)	29.50%	3.50E-12	N -> K	hypothetical protein	Substitution	KLI73433.1	hypothetical protein CDS
Variants: VaphiVaphiSt2_B_A_FDSW210055054-2r	T	192104	192104	1	G -> T	92	SNP (transversion)	30.40%	1.50E-12		hypothetical protein	None	KLI73433.1	hypothetical protein CDS
Variants: VaAphrodite_B_FDSW210055058-2r	A	192111	192111	1	C -> A	108	SNP (transversion)	25.90%	4.60E-13	M -> I	hypothetical protein	Substitution	KLI73433.1	hypothetical protein CDS
Variants: VaAres1_B_FDSW210055061-2r	A	192111	192111	1	C -> A	92	SNP (transversion)	26.10%	1.50E-07	M -> I	hypothetical protein	Substitution	KLI73433.1	hypothetical protein CDS
Variants: VaAres1_C_FDSW210055062-1r	T	192120	192120	1	G -> T	123	SNP (transversion)	26.80%	3.50E-10		hypothetical protein	None	KLI73433.1	hypothetical protein CDS
Variants: VaAres1_B_FDSW210055061-2r	A	192122	192122	1	C -> A	95	SNP (transversion)	26.30%	6.90E-08	V -> F	hypothetical protein	Substitution	KLI73433.1	hypothetical protein CDS
Variants: VaphiVaphiSt2_B_A_FDSW210055054-2r	A	192122	192122	1	C -> A	95	SNP (transversion)	28.40%	2.20E-11	V -> F	hypothetical protein	Substitution	KLI73433.1	hypothetical protein CDS
Variants: VaAres1_A_FDSW210055060-2r	T	272667	272667	1	G -> T	86	SNP (transversion)	25.60%	2.30E-16					
Variants: VaAres1_B_FDSW210055061-2r	T	272667	272667	1	G -> T	101	SNP (transversion)	27.70%	2.50E-16					
Variants: VaphiVaphiSt2_B_A_FDSW210055054-2r	T	272667	272667	1	G -> T	88	SNP (transversion)	28.40%	2.70E-17					

Variants: VaAres1_A_FDSW210055060-2r	A	284583	284583	1	C -> A	77	SNP (transversion)	26.00%	4.40E-08		hypothetical protein	None	KLI73493.1	hypothetical protein CDS
Variants: VaAres1_A_FDSW210055060-2r	A	284587	284587	1	T -> A	76	SNP (transversion)	27.60%	1.70E-14	D -> V	hypothetical protein	Substitution	KLI73493.1	hypothetical protein CDS
Variants: VaAphrodite_B_FDSW210055058-2r	A	284590	284590	1	T -> A	133	SNP (transversion)	25.60%	2.80E-18	K -> M	hypothetical protein	Substitution	KLI73493.1	hypothetical protein CDS

Contig 5

Variant	Name	Minimum	Maximum	Length	Change	Coverage	Polymorphism Type	Variant Frequency	Variant P-Value (approximate)	Amino Acid Change	product	Protein Effect	protein_id	CDS
Variants: VaAres1_B_FDSW210055061-2r	C	127704	127704	1	T -> C	216	SNP (transition)	25.90%	7.20E-150		RNA polymerase sigma factor RpoD	None	KLI73168.1	RNA polymerase sigma factor RpoD CDS
Variants: VaAres1_B_FDSW210055061-2r	G	127710	127710	1	A -> G	220	SNP (transition)	28.20%	2.40E-168		RNA polymerase sigma factor RpoD	None	KLI73168.1	RNA polymerase sigma factor RpoD CDS
Variants: VaAres1_B_FDSW210055061-2r	C	127716	127716	1	T -> C	222	SNP (transition)	28.80%	1.80E-174		RNA polymerase sigma factor RpoD	None	KLI73168.1	RNA polymerase sigma factor RpoD CDS
Variants: VaAres1_B_FDSW210055061-2r	A	127725	127725	1	G -> A	221	SNP (transition)	33.00%	2.60E-189		RNA polymerase sigma factor RpoD	None	KLI73168.1	RNA polymerase sigma factor RpoD CDS
Variants: VaAres1_B_FDSW210055061-2r	G	127734	127734	1	C -> G	218	SNP (transversion)	34.40%	4.80E-211		RNA polymerase sigma factor RpoD	None	KLI73168.1	RNA polymerase sigma factor RpoD CDS
Variants: VaphiVaphiSt2_B_A_FDSW210055054-2r	G	127734	127734	1	C -> G	188	SNP (transversion)	25.00%	1.70E-120		RNA polymerase sigma factor RpoD	None	KLI73168.1	RNA polymerase sigma factor RpoD CDS
Variants: VaAres1_B_FDSW210055061-2r	C	127737	127737	1	T -> C	220	SNP (transition)	35.90%	5.20E-224		RNA polymerase sigma factor RpoD	None	KLI73168.1	RNA polymerase sigma factor RpoD CDS
Variants: VaphiVaphiSt2_B_A_FDSW210055054-2r	C	127737	127737	1	T -> C	185	SNP (transition)	25.90%	6.30E-124		RNA polymerase sigma factor RpoD	None	KLI73168.1	RNA polymerase sigma factor RpoD CDS
Variants: VaAres1_B_FDSW210055061-2r	G	127743	127743	1	A -> G	222	SNP (transition)	37.80%	1.90E-240		RNA polymerase sigma factor RpoD	None	KLI73168.1	RNA polymerase sigma factor RpoD CDS
Variants: VaphiVaphiSt2_B_A_FDSW210055054-2r	G	127743	127743	1	A -> G	187	SNP (transition)	27.80%	6.30E-136		RNA polymerase sigma factor RpoD	None	KLI73168.1	RNA polymerase sigma factor RpoD CDS
Variants: VaAres1_B_FDSW210055061-2r	G	127764	127764	1	A -> G	221	SNP (transition)	38.50%	4.70E-244		RNA polymerase sigma factor RpoD	None	KLI73168.1	RNA polymerase sigma factor RpoD CDS
Variants: VaphiVaphiSt2_B_A_FDSW210055054-2r	G	127764	127764	1	A -> G	192	SNP (transition)	28.60%	5.30E-139		RNA polymerase sigma factor RpoD	None	KLI73168.1	RNA polymerase sigma factor RpoD CDS
Variants: VaAres1_B_FDSW210055061-2r	G	127767	127767	1	A -> G	220	SNP (transition)	38.60%	2.90E-244		RNA polymerase sigma factor RpoD	None	KLI73168.1	RNA polymerase sigma factor RpoD CDS
Variants: VaphiVaphiSt2_B_A_FDSW210055054-2r	G	127767	127767	1	A -> G	193	SNP (transition)	28.50%	7.50E-150		RNA polymerase sigma factor RpoD	None	KLI73168.1	RNA polymerase sigma factor RpoD CDS
Variants: VaAres1_B_FDSW210055061-2r	C	127770	127770	1	T -> C	217	SNP (transition)	39.20%	6.70E-245		RNA polymerase sigma factor RpoD	None	KLI73168.1	RNA polymerase sigma factor RpoD CDS
Variants: VaphiVaphiSt2_B_A_FDSW210055054-2r	C	127770	127770	1	T -> C	197	SNP (transition)	27.90%	8.80E-144		RNA polymerase sigma factor RpoD	None	KLI73168.1	RNA polymerase sigma factor RpoD CDS
Variants: VaAres1_B_FDSW210055061-2r	G	127779	127779	1	A -> G	215	SNP (transition)	38.60%	8.10E-247		RNA polymerase sigma factor RpoD	None	KLI73168.1	RNA polymerase sigma factor RpoD CDS
Variants: VaphiVaphiSt2_B_A_FDSW210055054-2r	G	127779	127779	1	A -> G	195	SNP (transition)	27.70%	2.30E-146		RNA polymerase sigma factor RpoD	None	KLI73168.1	RNA polymerase sigma factor RpoD CDS
Variants: VaAres1_B_FDSW210055061-2r	G	127782	127782	1	T -> G	213	SNP (transversion)	38.00%	4.70E-224		RNA polymerase sigma factor RpoD	None	KLI73168.1	RNA polymerase sigma factor RpoD CDS
Variants: VaphiVaphiSt2_B_A_FDSW210055054-2r	G	127782	127782	1	T -> G	198	SNP (transversion)	26.80%	1.70E-137		RNA polymerase sigma factor RpoD	None	KLI73168.1	RNA polymerase sigma factor RpoD CDS
Variants: VaAres1_B_FDSW210055061-2r	C	127788	127788	1	T -> C	214	SNP (transition)	35.50%	1.60E-207		RNA polymerase sigma factor RpoD	None	KLI73168.1	RNA polymerase sigma factor RpoD CDS
Variants: VaphiVaphiSt2_B_A_FDSW210055054-2r	C	127788	127788	1	T -> C	197	SNP (transition)	25.40%	1.80E-123		RNA polymerase sigma factor RpoD	None	KLI73168.1	RNA polymerase sigma factor RpoD CDS
Variants: VaAres1_B_FDSW210055061-2r	G	127797	127797	1	A -> G	207	SNP (transition)	33.30%	3.00E-186		RNA polymerase sigma factor RpoD	None	KLI73168.1	RNA polymerase sigma factor RpoD CDS
Variants: VaAres1_B_FDSW210055061-2r	A	127803	127803	1	G -> A	206	SNP (transition)	32.50%	4.80E-180		RNA polymerase sigma factor RpoD	None	KLI73168.1	RNA polymerase sigma factor RpoD CDS
Variants: VaAres1_B_FDSW210055061-2r	G	127806	127806	1	A -> G	206	SNP (transition)	32.00%	1.90E-183		RNA polymerase sigma factor RpoD	None	KLI73168.1	RNA polymerase sigma factor RpoD CDS

Variants: VaAres1_B_FDSW210055061-2r	A	127815	127815	1	G -> A	194	SNP (transition)	31.40%	6.70E-157	RNA polymerase sigma factor RpoD	None	KLI731 68.1	RNA polymerase sigma factor RpoD CDS
Variants: VaAres1_B_FDSW210055061-2r	G	127818	127818	1	C -> G	193	SNP (transversion)	30.10%	1.70E-159	RNA polymerase sigma factor RpoD	None	KLI731 68.1	RNA polymerase sigma factor RpoD CDS
Variants: VaAres1_B_FDSW210055061-2r	GA G	127824	127826	2	CAA -> GAG	179	Substitution	26.30%	1.20E-121	RNA polymerase sigma factor RpoD	None	KLI731 68.1	RNA polymerase sigma factor RpoD CDS
Variants: VaAres1_B_FDSW210055061-2r	C	127833	127833	1	T -> C	175	SNP (transition)	26.30%	2.50E-128	RNA polymerase sigma factor RpoD	None	KLI731 68.1	RNA polymerase sigma factor RpoD CDS

Contig 6

Variant	Name	Minimum	Maximum	Length	Change	Coverage	Polymorphism Type	Variant Frequency	Variant P-Value (approximate)	Amino Acid Change	CDS	product	Protein Effect	protein_id
Variants: VaAphrodite_C_FDSW210055059-1r	A	21	21	1	T -> A	258	SNP (transversion)	26.40%	3.20E-148					
Variants: VaAres1_C_FDSW210055062-1r	CGC	276	278	3	GAT -> CGC	307 -> 314	Substitution	23.7% -> 25.8%	2.50E-150					
Variants: VaAphrodite_A_FDSW210055057-2r	CC	280	281	2	AG -> CC	284 -> 288	Substitution	27.4% -> 27.8%	2.20E-173					
Variants: VaAres1_A_FDSW210055060-2r	CC	280	281	2	AG -> CC	203 -> 205	Substitution	25.1% -> 25.9%	1.20E-115					
Variants: VaAres1_C_FDSW210055062-1r	CC	280	281	2	AG -> CC	329 -> 334	Substitution	28.6% -> 29.3%	2.80E-189					
Variants: VaAphrodite_A_FDSW210055057-2r	TTAT	283	286	4	GAGG -> TTAT	293 -> 303	Substitution	28.3% -> 29.6%	2.40E-188					
Variants: VaAres1_A_FDSW210055060-2r	TTAT	283	286	4	GAGG -> TTAT	211 -> 213	Substitution	27.0% -> 28.6%	6.70E-128					
Variants: VaAres1_C_FDSW210055062-1r	TTAT	283	286	4	GAGG -> TTAT	346 -> 354	Substitution	31.2% -> 33.1%	3.80E-240					
Variants: VaAphrodite_A_FDSW210055057-2r	A	288	288	1	T -> A	322	SNP (transversion)	32.00%	9.00E-234					
Variants: VaAres1_A_FDSW210055060-2r	A	288	288	1	T -> A	228	SNP (transversion)	29.80%	2.90E-159					
Variants: VaAres1_C_FDSW210055062-1r	A	288	288	1	T -> A	365	SNP (transversion)	35.90%	8.60E-305					
Variants: VaAphrodite_A_FDSW210055057-2r	C	293	294	2	AG -> C	331 -> 332	Deletion	31.0% -> 31.1%	2.00E-70					
Variants: VaAres1_A_FDSW210055060-2r	C	293	294	2	AG -> C	235 -> 236	Deletion	28.8% -> 28.9%	4.70E-51					
Variants: VaAres1_C_FDSW210055062-1r	C	293	294	2	AG -> C	373 -> 374	Deletion	35.6% -> 35.7%	1.90E-100					
Variants: VaAphrodite_A_FDSW210055057-2r	T	300	300	1	G -> T	344	SNP (transversion)	40.40%	0					
Variants: VaAres1_A_FDSW210055060-2r	T	300	300	1	G -> T	248	SNP (transversion)	41.10%	1.10E-265					
Variants: VaAres1_C_FDSW210055062-1r	T	300	300	1	G -> T	399	SNP (transversion)	44.10%	0					
Variants: VaAphrodite_A_FDSW210055057-2r	T	302	302	1	C -> T	358	SNP (transition)	35.80%	2.2E-310					
Variants: VaAres1_A_FDSW210055060-2r	T	302	302	1	C -> T	254	SNP (transition)	35.40%	2.50E-227					
Variants: VaAres1_C_FDSW210055062-1r	T	302	302	1	C -> T	409	SNP (transition)	37.40%	0					

Variants: VaAphrodite_A_FDSW210055057-2r	GAG	305	308	4	TTTA -> GAG	386 -> 394	Deletion	32.5% -> 33.2%	6.30E-102
Variants: VaAres1_A_FDSW210055060-2r	GAG	305	308	4	TTTA -> GAG	274 -> 276	Deletion	32.2% -> 32.5%	3.00E-71
Variants: VaAres1_C_FDSW210055062-1r	GAG	305	308	4	TTTA -> GAG	423 -> 444	Deletion	34.0% -> 35.7%	2.60E-123
Variants: VaAphrodite_A_FDSW210055057-2r	AA	310	311	2	CC -> AA	414 -> 421	Substitution	49.2% -> 49.3%	0
Variants: VaAphrodite_B_FDSW210055058-2r	AA	310	311	2	CC -> AA	226 -> 229	Substitution	27.9% -> 28.8%	9.50E-141
Variants: VaAphrodite_C_FDSW210055059-1r	AA	310	311	2	CC -> AA	373 -> 379	Substitution	34.8% -> 35.4%	0
Variants: VaAres1_A_FDSW210055060-2r	AA	310	311	2	CC -> AA	286 -> 289	Substitution	48.6% -> 49.1%	0
Variants: VaAres1_B_FDSW210055061-2r	AA	310	311	2	CC -> AA	259 -> 269	Substitution	29.3% -> 30.1%	1.30E-190
Variants: VaAres1_C_FDSW210055062-1r	AA	310	311	2	CC -> AA	455 -> 462	Substitution	48.1% -> 49.1%	0
Variants: VaAphrodite_A_FDSW210055057-2r	T	390	390	1	C -> T	816	SNP (transition)	40.60%	0
Variants: VaAphrodite_B_FDSW210055058-2r	T	390	390	1	C -> T	590	SNP (transition)	43.70%	0
Variants: VaAphrodite_C_FDSW210055059-1r	T	390	390	1	C -> T	956	SNP (transition)	39.10%	0
Variants: VaAres1_A_FDSW210055060-2r	T	390	390	1	C -> T	548	SNP (transition)	38.50%	0
Variants: VaAres1_B_FDSW210055061-2r	T	390	390	1	C -> T	685	SNP (transition)	46.10%	0
Variants: VaAres1_C_FDSW210055062-1r	T	390	390	1	C -> T	987	SNP (transition)	39.60%	0
Variants: VaphiVaphiSt2_B_A_FDSW210055054-2r	T	390	390	1	C -> T	377	SNP (transition)	45.90%	0
Variants: VaAphrodite_B_FDSW210055058-2r	C	397	397	1	T -> C	597	SNP (transition)	30.30%	0
Variants: VaAres1_A_FDSW210055060-2r	C	397	397	1	T -> C	558	SNP (transition)	26.30%	0
Variants: VaAres1_B_FDSW210055061-2r	C	397	397	1	T -> C	689	SNP (transition)	27.90%	0
Variants: VaphiVaphiSt2_B_A_FDSW210055054-2r	C	397	397	1	T -> C	387	SNP (transition)	34.10%	0
Variants: VaAphrodite_A_FDSW210055057-2r	T	432	432	1	C -> T	738	SNP (transition)	28.00%	0
Variants: VaAphrodite_B_FDSW210055058-2r	T	432	432	1	C -> T	597	SNP (transition)	29.10%	0

Variants: VaAres1_A_FDSW210055060-2r	T	432	432	1	C -> T	492	SNP (transition)	27.80%	0					
Variants: VaAres1_B_FDSW210055061-2r	T	432	432	1	C -> T	691	SNP (transition)	27.10%	0					
Variants: VaAres1_C_FDSW210055062-1r	T	432	432	1	C -> T	886	SNP (transition)	25.70%	0					
Variants: VaphiVaphiSt2_B_A_FDSW210055054-2r	T	432	432	1	C -> T	385	SNP (transition)	32.50%	9.7E-322					
Variants: VaAphrodite_A_FDSW210055057-2r	C	437	437	1	T -> C	723	SNP (transition)	52.10%	0					
Variants: VaAphrodite_B_FDSW210055058-2r	C	437	437	1	T -> C	601	SNP (transition)	53.20%	0					
Variants: VaAphrodite_C_FDSW210055059-1r	C	437	437	1	T -> C	953	SNP (transition)	55.70%	0					
Variants: VaAres1_A_FDSW210055060-2r	C	437	437	1	T -> C	478	SNP (transition)	54.40%	0					
Variants: VaAres1_B_FDSW210055061-2r	C	437	437	1	T -> C	685	SNP (transition)	49.20%	0					
Variants: VaAres1_C_FDSW210055062-1r	C	437	437	1	T -> C	859	SNP (transition)	54.90%	0					
Variants: VaphiVaphiSt2_B_A_FDSW210055054-2r	C	437	437	1	T -> C	389	SNP (transition)	49.40%	0					
Variants: VaAres1_A_FDSW210055060-2r	C	62190	62190	1	A -> C	99	SNP (transversion)	27.30%	6.20E-11	L -> V	gamma-glutamyl kinase CDS	gamma-glutamyl kinase	Substitution	KLI72930.1

Contig 7

Variant	Name	Minimum	Maximum	Length	Change	Coverage	Polymorphism Type	Variant Frequency	Variant P-Value (approximate)	Amino Acid Change	CDS	product	Protein Effect	protein_id
Variants: VaphiVaphiSt2_B_A_FDSW210055054-2r	A	111765	111765	1	C -> A	75	SNP (transversion)	25.30%	1.50E-07		cytoplasmic protein CDS	cytoplasmic protein	None	KLI72781.1
Variants: VaAres1_B_FDSW210055061-2r	A	188870	188870	1	C -> A	95	SNP (transversion)	100.00%	0	G -> V	prop expression regulator CDS	prop expression regulator	Substitution	KLI72845.1
Variants: VaphiVaphiSt2_B_A_FDSW210055054-2r	G	216951	216951	1	A -> G	119	SNP (transition)	28.60%	5.90E-36		calcium-binding protein CDS	calcium-binding protein	None	KLI72868.1
Variants: VaAphrodite_A_FDSW210055057-2r	A	216957	216957	1	G -> A	200	SNP (transition)	27.00%	1.20E-54		calcium-binding protein CDS	calcium-binding protein	None	KLI72868.1
Variants: VaAphrodite_A_FDSW210055057-2r	G	216975	216975	1	A -> G	200	SNP (transition)	28.50%	4.30E-59		calcium-binding protein CDS	calcium-binding protein	None	KLI72868.1
Variants: VaphiVaphiSt2_B_A_FDSW210055054-2r	A	216978	216978	1	G -> A	137	SNP (transition)	40.10%	########		calcium-binding protein CDS	calcium-binding protein	None	KLI72868.1
Variants: VaAphrodite_A_FDSW210055057-2r	C	216990	216990	1	T -> C	209	SNP (transition)	45.50%	########		calcium-binding protein CDS	calcium-binding protein	None	KLI72868.1
Variants: VaphiVaphiSt2_B_A_FDSW210055054-2r	C	216990	216990	1	T -> C	133	SNP (transition)	62.40%	########		calcium-binding protein CDS	calcium-binding protein	None	KLI72868.1
Variants: VaphiSt2_B_FDSW210055056-1r	C	216990	216990	1	T -> C	237	SNP (transition)	32.10%	4.20E-90		calcium-binding protein CDS	calcium-binding protein	None	KLI72868.1
Variants: VaAphrodite_A_FDSW210055057-2r	A	216993	216993	1	G -> A	213	SNP (transition)	42.70%	########		calcium-binding protein CDS	calcium-binding protein	None	KLI72868.1
Variants: VaphiVaphiSt2_B_A_FDSW210055054-2r	A	216993	216993	1	G -> A	141	SNP (transition)	56.70%	########		calcium-binding protein CDS	calcium-binding protein	None	KLI72868.1
Variants: VaphiSt2_B_FDSW210055056-1r	A	216993	216993	1	G -> A	229	SNP (transition)	25.80%	6.20E-64		calcium-binding protein CDS	calcium-binding protein	None	KLI72868.1
Variants: VaAphrodite_A_FDSW210055057-2r	C	217002	217002	1	A -> C	231	SNP (transversion)	34.60%	########		calcium-binding protein CDS	calcium-binding protein	None	KLI72868.1
Variants: VaAres1_A_FDSW210055060-2r	C	217002	217002	1	A -> C	135	SNP (transversion)	25.90%	6.50E-56		calcium-binding protein CDS	calcium-binding protein	None	KLI72868.1
Variants: VaphiVaphiSt2_B_A_FDSW210055054-2r	C	217002	217002	1	A -> C	167	SNP (transversion)	68.90%	########		calcium-binding protein CDS	calcium-binding protein	None	KLI72868.1
Variants: VaAphrodite_A_FDSW210055057-2r	T	217017	217017	1	C -> T	269	SNP (transition)	50.90%	########		calcium-binding protein CDS	calcium-binding protein	None	KLI72868.1
Variants: VaAres1_A_FDSW210055060-2r	T	217017	217017	1	C -> T	155	SNP (transition)	45.80%	########		calcium-binding protein CDS	calcium-binding protein	None	KLI72868.1
Variants: VaAres1_B_FDSW210055061-2r	T	217017	217017	1	C -> T	130	SNP (transition)	40.80%	2.60E-96		calcium-binding protein CDS	calcium-binding protein	None	KLI72868.1
Variants: VaphiVaphiSt2_B_A_FDSW210055054-2r	T	217017	217017	1	C -> T	197	SNP (transition)	82.70%	0		calcium-binding protein CDS	calcium-binding protein	None	KLI72868.1
Variants: VaphiSt2_B_FDSW210055056-1r	T	217017	217017	1	C -> T	251	SNP (transition)	31.10%	########		calcium-binding protein CDS	calcium-binding protein	None	KLI72868.1

Contig 13

Variant	Name	Minimum	Maximum	Length	Change	Coverage	Polymorphism Type	Variant Frequency	Variant P-Value (approximate)	Amino Acid Change	CDS	product	Protein Effect	protein_id
Variants: VaphiSt2_B_FDSW210055056-1r		34441	34446	6	#NAME?	271	Deletion	60.50%	1.20E-186	NIV -> N	outer membrane protein assembly protein YaeT CDS	outer membrane protein assembly protein YaeT	Deletion	KLI71940.1
Variants: VaAphrodite_B_FDSW210055058-2r	A	122350	122350	1	G -> A	174	SNP (transition)	27.60%	8.20E-116					

Contig 14

Variant	Name	Minimum	Maximum	Length	Change	Coverage	Polymorphism Type	Variant Frequency	Variant P-Value (approximate)	Amino Acid Change	CDS	product	Protein Effect	protein_id
Variants: VaAphrodite_A_FDSW210055057-2r	A	7	7	1	G -> A	388	SNP (transition)	37.10%	0		hypothetical protein CDS	hypothetical protein	None	KLI717 69.1
Variants: VaAphrodite_B_FDSW210055058-2r	A	7	7	1	G -> A	292	SNP (transition)	40.80%	5.8E-321		hypothetical protein CDS	hypothetical protein	None	KLI717 69.1
Variants: VaAphrodite_C_FDSW210055059-1r	A	7	7	1	G -> A	403	SNP (transition)	37.00%	0		hypothetical protein CDS	hypothetical protein	None	KLI717 69.1
Variants: VaAres1_A_FDSW210055060-2r	A	7	7	1	G -> A	267	SNP (transition)	37.50%	########		hypothetical protein CDS	hypothetical protein	None	KLI717 69.1
Variants: VaAres1_B_FDSW210055061-2r	A	7	7	1	G -> A	271	SNP (transition)	42.10%	5.8E-321		hypothetical protein CDS	hypothetical protein	None	KLI717 69.1
Variants: VaAres1_C_FDSW210055062-1r	A	7	7	1	G -> A	428	SNP (transition)	43.70%	0		hypothetical protein CDS	hypothetical protein	None	KLI717 69.1
Variants: VaphiVaphiSt2_B_A_FDSW2100550 54-2r	A	7	7	1	G -> A	228	SNP (transition)	49.10%	3.3E-314		hypothetical protein CDS	hypothetical protein	None	KLI717 69.1
Variants: VaphiSt2_B_FDSW210055056-1r	A	7	7	1	G -> A	509	SNP (transition)	38.70%	0		hypothetical protein CDS	hypothetical protein	None	KLI717 69.1
Variants: VaAphrodite_A_FDSW210055057-2r	GA T	10	12	2	CAC -> GAT	397 -> 405	Substitution	33.1% -> 33.8%	0	V -> I	hypothetical protein CDS	hypothetical protein	Substituti on	KLI717 69.1
Variants: VaAphrodite_B_FDSW210055058-2r	GA T	10	12	2	CAC -> GAT	298 -> 299	Substitution	38.8% -> 39.6%	9.2E-310	V -> I	hypothetical protein CDS	hypothetical protein	Substituti on	KLI717 69.1
Variants: VaAphrodite_C_FDSW210055059-1r	GA T	10	12	2	CAC -> GAT	415 -> 421	Substitution	35.4% -> 36.3%	0	V -> I	hypothetical protein CDS	hypothetical protein	Substituti on	KLI717 69.1
Variants: VaAres1_A_FDSW210055060-2r	GA T	10	12	2	CAC -> GAT	270 -> 271	Substitution	36.3% -> 36.9%	########	V -> I	hypothetical protein CDS	hypothetical protein	Substituti on	KLI717 69.1
Variants: VaAres1_B_FDSW210055061-2r	GA T	10	12	2	CAC -> GAT	266 -> 270	Substitution	41.1% -> 41.4%	########	V -> I	hypothetical protein CDS	hypothetical protein	Substituti on	KLI717 69.1
Variants: VaAres1_C_FDSW210055062-1r	GA T	10	12	2	CAC -> GAT	441 -> 444	Substitution	42.6% -> 43.1%	0	V -> I	hypothetical protein CDS	hypothetical protein	Substituti on	KLI717 69.1
Variants: VaphiVaphiSt2_B_A_FDSW2100550 54-2r	GA T	10	12	2	CAC -> GAT	235 -> 236	Substitution	45.8% -> 46.0%	########	V -> I	hypothetical protein CDS	hypothetical protein	Substituti on	KLI717 69.1
Variants: VaphiSt2_B_FDSW210055056-1r	GA T	10	12	2	CAC -> GAT	510 -> 513	Substitution	37.1% -> 37.2%	0	V -> I	hypothetical protein CDS	hypothetical protein	Substituti on	KLI717 69.1
Variants: VaAphrodite_A_FDSW210055057-2r	G	22	22	1	A -> G	418	SNP (transition)	41.60%	0		hypothetical protein CDS	hypothetical protein	None	KLI717 69.1
Variants: VaAphrodite_B_FDSW210055058-2r	G	22	22	1	A -> G	317	SNP (transition)	48.60%	0		hypothetical protein CDS	hypothetical protein	None	KLI717 69.1
Variants: VaAphrodite_C_FDSW210055059-1r	G	22	22	1	A -> G	429	SNP (transition)	45.00%	0		hypothetical protein CDS	hypothetical protein	None	KLI717 69.1
Variants: VaAres1_A_FDSW210055060-2r	G	22	22	1	A -> G	279	SNP (transition)	47.30%	0		hypothetical protein CDS	hypothetical protein	None	KLI717 69.1
Variants: VaAres1_B_FDSW210055061-2r	G	22	22	1	A -> G	279	SNP (transition)	50.90%	0		hypothetical protein CDS	hypothetical protein	None	KLI717 69.1
Variants: VaAres1_C_FDSW210055062-1r	G	22	22	1	A -> G	459	SNP (transition)	55.80%	0		hypothetical protein CDS	hypothetical protein	None	KLI717 69.1
Variants: VaphiVaphiSt2_B_A_FDSW2100550 54-2r	G	22	22	1	A -> G	246	SNP (transition)	52.00%	0		hypothetical protein CDS	hypothetical protein	None	KLI717 69.1
Variants: VaphiSt2_B_FDSW210055056-1r	G	22	22	1	A -> G	526	SNP (transition)	47.10%	0		hypothetical protein CDS	hypothetical protein	None	KLI717 69.1
Variants: VaAphrodite_A_FDSW210055057-2r	G	28	28	1	A -> G	435	SNP (transition)	29.70%	0		hypothetical protein CDS	hypothetical protein	None	KLI717 69.1
Variants: VaAphrodite_B_FDSW210055058-2r	G	28	28	1	A -> G	328	SNP (transition)	36.30%	0		hypothetical protein CDS	hypothetical protein	None	KLI717 69.1
Variants: VaAphrodite_C_FDSW210055059-1r	G	28	28	1	A -> G	444	SNP (transition)	32.90%	0		hypothetical protein CDS	hypothetical protein	None	KLI717 69.1
Variants: VaAres1_A_FDSW210055060-2r	G	28	28	1	A -> G	295	SNP (transition)	32.90%	########		hypothetical protein CDS	hypothetical protein	None	KLI717 69.1

Variants: VaAres1_B_FDSW210055061-2r	G	28	28	1	A -> G	292	SNP (transition)	38.00%	########		hypothetical protein CDS	hypothetical protein	None	KLI717 69.1
Variants: VaAres1_C_FDSW210055062-1r	G	28	28	1	A -> G	470	SNP (transition)	37.40%	0		hypothetical protein CDS	hypothetical protein	None	KLI717 69.1
Variants: VaphiVaphiSt2_B_A_FDSW2100550 54-2r	G	28	28	1	A -> G	263	SNP (transition)	41.40%	########		hypothetical protein CDS	hypothetical protein	None	KLI717 69.1
Variants: VaphiSt2_B_FDSW210055056-1r	G	28	28	1	A -> G	535	SNP (transition)	34.00%	0		hypothetical protein CDS	hypothetical protein	None	KLI717 69.1
Variants: VaAphrodite_A_FDSW210055057-2r	G	31	31	1	A -> G	442	SNP (transition)	83.90%	0		hypothetical protein CDS	hypothetical protein	None	KLI717 69.1
Variants: VaAphrodite_B_FDSW210055058-2r	G	31	31	1	A -> G	336	SNP (transition)	82.70%	0		hypothetical protein CDS	hypothetical protein	None	KLI717 69.1
Variants: VaAphrodite_C_FDSW210055059-1r	G	31	31	1	A -> G	453	SNP (transition)	83.90%	0		hypothetical protein CDS	hypothetical protein	None	KLI717 69.1
Variants: VaAres1_A_FDSW210055060-2r	G	31	31	1	A -> G	297	SNP (transition)	84.50%	0		hypothetical protein CDS	hypothetical protein	None	KLI717 69.1
Variants: VaAres1_B_FDSW210055061-2r	G	31	31	1	A -> G	302	SNP (transition)	85.10%	0		hypothetical protein CDS	hypothetical protein	None	KLI717 69.1
Variants: VaAres1_C_FDSW210055062-1r	G	31	31	1	A -> G	481	SNP (transition)	80.00%	0		hypothetical protein CDS	hypothetical protein	None	KLI717 69.1
Variants: VaphiVaphiSt2_B_A_FDSW2100550 54-2r	G	31	31	1	A -> G	262	SNP (transition)	85.50%	0		hypothetical protein CDS	hypothetical protein	None	KLI717 69.1
Variants: VaphiSt2_B_FDSW210055056-1r	G	31	31	1	A -> G	546	SNP (transition)	82.80%	0		hypothetical protein CDS	hypothetical protein	None	KLI717 69.1
Variants: VaAphrodite_A_FDSW210055057-2r	A	34	34	1	G -> A	450	SNP (transition)	83.80%	0		hypothetical protein CDS	hypothetical protein	None	KLI717 69.1
Variants: VaAphrodite_B_FDSW210055058-2r	A	34	34	1	G -> A	340	SNP (transition)	81.50%	0		hypothetical protein CDS	hypothetical protein	None	KLI717 69.1
Variants: VaAphrodite_C_FDSW210055059-1r	A	34	34	1	G -> A	461	SNP (transition)	83.50%	0		hypothetical protein CDS	hypothetical protein	None	KLI717 69.1
Variants: VaAres1_A_FDSW210055060-2r	A	34	34	1	G -> A	305	SNP (transition)	84.60%	0		hypothetical protein CDS	hypothetical protein	None	KLI717 69.1
Variants: VaAres1_B_FDSW210055061-2r	A	34	34	1	G -> A	304	SNP (transition)	84.20%	0		hypothetical protein CDS	hypothetical protein	None	KLI717 69.1
Variants: VaAres1_C_FDSW210055062-1r	A	34	34	1	G -> A	485	SNP (transition)	79.00%	0		hypothetical protein CDS	hypothetical protein	None	KLI717 69.1
Variants: VaphiVaphiSt2_B_A_FDSW2100550 54-2r	A	34	34	1	G -> A	268	SNP (transition)	84.00%	0		hypothetical protein CDS	hypothetical protein	None	KLI717 69.1
Variants: VaphiSt2_B_FDSW210055056-1r	A	34	34	1	G -> A	552	SNP (transition)	82.40%	0		hypothetical protein CDS	hypothetical protein	None	KLI717 69.1
Variants: VaAphrodite_A_FDSW210055057-2r	T	39	39	1	G -> T	454	SNP (transversion)	27.80%	########	H -> N	hypothetical protein CDS	hypothetical protein	Substituti on	KLI717 69.1
Variants: VaAphrodite_B_FDSW210055058-2r	T	39	39	1	G -> T	347	SNP (transversion)	33.40%	########	H -> N	hypothetical protein CDS	hypothetical protein	Substituti on	KLI717 69.1
Variants: VaAphrodite_C_FDSW210055059-1r	T	39	39	1	G -> T	462	SNP (transversion)	31.20%	0	H -> N	hypothetical protein CDS	hypothetical protein	Substituti on	KLI717 69.1
Variants: VaAres1_A_FDSW210055060-2r	T	39	39	1	G -> T	310	SNP (transversion)	30.30%	########	H -> N	hypothetical protein CDS	hypothetical protein	Substituti on	KLI717 69.1
Variants: VaAres1_B_FDSW210055061-2r	T	39	39	1	G -> T	308	SNP (transversion)	36.40%	########	H -> N	hypothetical protein CDS	hypothetical protein	Substituti on	KLI717 69.1
Variants: VaAres1_C_FDSW210055062-1r	T	39	39	1	G -> T	494	SNP (transversion)	33.60%	0	H -> N	hypothetical protein CDS	hypothetical protein	Substituti on	KLI717 69.1
Variants: VaphiVaphiSt2_B_A_FDSW2100550 54-2r	T	39	39	1	G -> T	280	SNP (transversion)	37.90%	########	H -> N	hypothetical protein CDS	hypothetical protein	Substituti on	KLI717 69.1
Variants: VaphiSt2_B_FDSW210055056-1r	T	39	39	1	G -> T	562	SNP (transversion)	32.70%	0	H -> N	hypothetical protein CDS	hypothetical protein	Substituti on	KLI717 69.1
Variants: VaAphrodite_A_FDSW210055057-2r	G	49	49	1	A -> G	467	SNP (transition)	26.10%	########		hypothetical protein CDS	hypothetical protein	None	KLI717 69.1
Variants: VaAphrodite_B_FDSW210055058-2r	G	49	49	1	A -> G	358	SNP (transition)	30.20%	########		hypothetical protein CDS	hypothetical protein	None	KLI717 69.1

Variants: VaAphrodite_C_FDSW210055059-1r	G	49	49	1	A -> G	490	SNP (transition)	28.80%		0	hypothetical protein CDS	hypothetical protein	None	KLI717 69.1
Variants: VaAres1_A_FDSW210055060-2r	G	49	49	1	A -> G	309	SNP (transition)	27.50%	########		hypothetical protein CDS	hypothetical protein	None	KLI717 69.1
Variants: VaAres1_B_FDSW210055061-2r	G	49	49	1	A -> G	323	SNP (transition)	31.60%	########		hypothetical protein CDS	hypothetical protein	None	KLI717 69.1
Variants: VaAres1_C_FDSW210055062-1r	G	49	49	1	A -> G	497	SNP (transition)	31.60%		0	hypothetical protein CDS	hypothetical protein	None	KLI717 69.1
Variants: VaphiVaphiSt2_B_A_FDSW2100550 54-2r	G	49	49	1	A -> G	285	SNP (transition)	35.10%	########		hypothetical protein CDS	hypothetical protein	None	KLI717 69.1
Variants: VaphiSt2_B_FDSW210055056-1r	G	49	49	1	A -> G	574	SNP (transition)	30.50%		0	hypothetical protein CDS	hypothetical protein	None	KLI717 69.1
Variants: VaAphrodite_B_FDSW210055058-2r	A	58	58	1	G -> A	364	SNP (transition)	27.50%	########		hypothetical protein CDS	hypothetical protein	None	KLI717 69.1
Variants: VaAphrodite_C_FDSW210055059-1r	A	58	58	1	G -> A	508	SNP (transition)	26.20%	2.9E-314		hypothetical protein CDS	hypothetical protein	None	KLI717 69.1
Variants: VaAres1_A_FDSW210055060-2r	A	58	58	1	G -> A	315	SNP (transition)	25.70%	########		hypothetical protein CDS	hypothetical protein	None	KLI717 69.1
Variants: VaAres1_B_FDSW210055061-2r	A	58	58	1	G -> A	339	SNP (transition)	29.50%	########		hypothetical protein CDS	hypothetical protein	None	KLI717 69.1
Variants: VaAres1_C_FDSW210055062-1r	A	58	58	1	G -> A	507	SNP (transition)	30.20%		0	hypothetical protein CDS	hypothetical protein	None	KLI717 69.1
Variants: VaphiVaphiSt2_B_A_FDSW2100550 54-2r	A	58	58	1	G -> A	292	SNP (transition)	34.60%	########		hypothetical protein CDS	hypothetical protein	None	KLI717 69.1
Variants: VaphiSt2_B_FDSW210055056-1r	A	58	58	1	G -> A	588	SNP (transition)	28.40%		0	hypothetical protein CDS	hypothetical protein	None	KLI717 69.1
Variants: VaAphrodite_B_FDSW210055058-2r	A	66	66	1	G -> A	372	SNP (transition)	25.50%	########		hypothetical protein CDS	hypothetical protein	None	KLI717 69.1
Variants: VaAres1_B_FDSW210055061-2r	A	66	66	1	G -> A	359	SNP (transition)	28.10%	########		hypothetical protein CDS	hypothetical protein	None	KLI717 69.1
Variants: VaAres1_C_FDSW210055062-1r	A	66	66	1	G -> A	514	SNP (transition)	29.60%		0	hypothetical protein CDS	hypothetical protein	None	KLI717 69.1
Variants: VaphiVaphiSt2_B_A_FDSW2100550 54-2r	A	66	66	1	G -> A	298	SNP (transition)	33.60%	########		hypothetical protein CDS	hypothetical protein	None	KLI717 69.1
Variants: VaphiSt2_B_FDSW210055056-1r	A	66	66	1	G -> A	596	SNP (transition)	27.70%		0	hypothetical protein CDS	hypothetical protein	None	KLI717 69.1
Variants: VaAphrodite_B_FDSW210055058-2r	T	70	70	1	G -> T	371	SNP (transversion)	25.30%	########		hypothetical protein CDS	hypothetical protein	None	KLI717 69.1
Variants: VaAres1_B_FDSW210055061-2r	T	70	70	1	G -> T	362	SNP (transversion)	28.20%	########		hypothetical protein CDS	hypothetical protein	None	KLI717 69.1
Variants: VaAres1_C_FDSW210055062-1r	T	70	70	1	G -> T	512	SNP (transversion)	29.30%		0	hypothetical protein CDS	hypothetical protein	None	KLI717 69.1
Variants: VaphiVaphiSt2_B_A_FDSW2100550 54-2r	T	70	70	1	G -> T	296	SNP (transversion)	33.10%	########		hypothetical protein CDS	hypothetical protein	None	KLI717 69.1
Variants: VaphiSt2_B_FDSW210055056-1r	T	70	70	1	G -> T	590	SNP (transversion)	26.80%		0	hypothetical protein CDS	hypothetical protein	None	KLI717 69.1
Variants: VaAphrodite_A_FDSW210055057-2r	G	79	79	1	A -> G	464	SNP (transition)	33.80%		0	hypothetical protein CDS	hypothetical protein	None	KLI717 69.1
Variants: VaAphrodite_B_FDSW210055058-2r	G	79	79	1	A -> G	360	SNP (transition)	29.40%	########		hypothetical protein CDS	hypothetical protein	None	KLI717 69.1
Variants: VaAphrodite_C_FDSW210055059-1r	G	79	79	1	A -> G	494	SNP (transition)	31.60%		0	hypothetical protein CDS	hypothetical protein	None	KLI717 69.1
Variants: VaAres1_A_FDSW210055060-2r	G	79	79	1	A -> G	307	SNP (transition)	30.60%	########		hypothetical protein CDS	hypothetical protein	None	KLI717 69.1
Variants: VaAres1_B_FDSW210055061-2r	G	79	79	1	A -> G	343	SNP (transition)	26.20%	########		hypothetical protein CDS	hypothetical protein	None	KLI717 69.1
Variants: VaphiVaphiSt2_B_A_FDSW2100550 54-2r	G	79	79	1	A -> G	290	SNP (transition)	27.20%	########		hypothetical protein CDS	hypothetical protein	None	KLI717 69.1
Variants: VaphiSt2_B_FDSW210055056-1r	G	79	79	1	A -> G	574	SNP (transition)	29.10%		0	hypothetical protein CDS	hypothetical protein	None	KLI717 69.1

Variants: VaAphrodite_A_FDSW210055057-2r	A	85	85	1	G -> A	458	SNP (transition)	52.60%		0		hypothetical protein CDS	hypothetical protein	None	KLI717 69.1
Variants: VaAphrodite_B_FDSW210055058-2r	A	85	85	1	G -> A	356	SNP (transition)	51.10%		0		hypothetical protein CDS	hypothetical protein	None	KLI717 69.1
Variants: VaAphrodite_C_FDSW210055059-1r	A	85	85	1	G -> A	488	SNP (transition)	50.00%		0		hypothetical protein CDS	hypothetical protein	None	KLI717 69.1
Variants: VaAres1_A_FDSW210055060-2r	A	85	85	1	G -> A	303	SNP (transition)	51.50%		0		hypothetical protein CDS	hypothetical protein	None	KLI717 69.1
Variants: VaAres1_B_FDSW210055061-2r	A	85	85	1	G -> A	340	SNP (transition)	51.50%		0		hypothetical protein CDS	hypothetical protein	None	KLI717 69.1
Variants: VaAres1_C_FDSW210055062-1r	A	85	85	1	G -> A	494	SNP (transition)	46.00%		0		hypothetical protein CDS	hypothetical protein	None	KLI717 69.1
Variants: VaphiVaphiSt2_B_A_FDSW2100550 54-2r	A	85	85	1	G -> A	282	SNP (transition)	56.70%		0		hypothetical protein CDS	hypothetical protein	None	KLI717 69.1
Variants: VaphiSt2_B_FDSW210055056-1r	A	85	85	1	G -> A	564	SNP (transition)	50.00%		0		hypothetical protein CDS	hypothetical protein	None	KLI717 69.1
Variants: VaAphrodite_A_FDSW210055057-2r	TG T	97	99	2	CGC -> TGT	441 -> 453	Substitution	30.4% -> 31.1%		0	A -> T	hypothetical protein CDS	hypothetical protein	Substituti on	KLI717 69.1
Variants: VaAphrodite_B_FDSW210055058-2r	T	97	97	1	C -> T	348	SNP (transition)	25.90%	########			hypothetical protein CDS	hypothetical protein	None	KLI717 69.1
Variants: VaAphrodite_C_FDSW210055059-1r	TG T	97	99	2	CGC -> TGT	459 -> 460	Substitution	28.1% -> 28.3%	4.6E-310		A -> T	hypothetical protein CDS	hypothetical protein	Substituti on	KLI717 69.1
Variants: VaAres1_A_FDSW210055060-2r	TG T	97	99	2	CGC -> TGT	292 -> 297	Substitution	27.1% -> 27.9%	########		A -> T	hypothetical protein CDS	hypothetical protein	Substituti on	KLI717 69.1
Variants: VaphiSt2_B_FDSW210055056-1r	TG T	97	99	2	CGC -> TGT	547 -> 552	Substitution	25.2% -> 25.9%	4.9E-324		A -> T	hypothetical protein CDS	hypothetical protein	Substituti on	KLI717 69.1
Variants: VaphiVaphiSt2_B_A_FDSW2100550 54-2r	A	109	109	1	G -> A	262	SNP (transition)	26.70%	########			hypothetical protein CDS	hypothetical protein	None	KLI717 69.1
Variants: VaAphrodite_A_FDSW210055057-2r	A	112	112	1	C -> A	411	SNP (transversion)	28.00%	########			hypothetical protein CDS	hypothetical protein	None	KLI717 69.1
Variants: VaAphrodite_A_FDSW210055057-2r	C	120	120	1	T -> C	400	SNP (transition)	49.30%		0	K -> E	hypothetical protein CDS	hypothetical protein	Substituti on	KLI717 69.1
Variants: VaAphrodite_B_FDSW210055058-2r	C	120	120	1	T -> C	314	SNP (transition)	44.60%		0	K -> E	hypothetical protein CDS	hypothetical protein	Substituti on	KLI717 69.1
Variants: VaAphrodite_C_FDSW210055059-1r	C	120	120	1	T -> C	429	SNP (transition)	48.30%		0	K -> E	hypothetical protein CDS	hypothetical protein	Substituti on	KLI717 69.1
Variants: VaAres1_A_FDSW210055060-2r	C	120	120	1	T -> C	261	SNP (transition)	46.00%		0	K -> E	hypothetical protein CDS	hypothetical protein	Substituti on	KLI717 69.1
Variants: VaAres1_B_FDSW210055061-2r	C	120	120	1	T -> C	309	SNP (transition)	46.00%		0	K -> E	hypothetical protein CDS	hypothetical protein	Substituti on	KLI717 69.1
Variants: VaAres1_C_FDSW210055062-1r	C	120	120	1	T -> C	431	SNP (transition)	43.20%		0	K -> E	hypothetical protein CDS	hypothetical protein	Substituti on	KLI717 69.1
Variants: VaphiVaphiSt2_B_A_FDSW2100550 54-2r	C	120	120	1	T -> C	255	SNP (transition)	46.30%		0	K -> E	hypothetical protein CDS	hypothetical protein	Substituti on	KLI717 69.1
Variants: VaphiSt2_B_FDSW210055056-1r	C	120	120	1	T -> C	506	SNP (transition)	44.70%		0	K -> E	hypothetical protein CDS	hypothetical protein	Substituti on	KLI717 69.1
Variants: VaAphrodite_A_FDSW210055057-2r	T	131	131	1	G -> T	373	SNP (transversion)	39.10%		0	T -> K	hypothetical protein CDS	hypothetical protein	Substituti on	KLI717 69.1
Variants: VaAphrodite_B_FDSW210055058-2r	T	131	131	1	G -> T	295	SNP (transversion)	35.30%	########		T -> K	hypothetical protein CDS	hypothetical protein	Substituti on	KLI717 69.1
Variants: VaAphrodite_C_FDSW210055059-1r	T	131	131	1	G -> T	390	SNP (transversion)	34.60%	########		T -> K	hypothetical protein CDS	hypothetical protein	Substituti on	KLI717 69.1
Variants: VaAres1_A_FDSW210055060-2r	T	131	131	1	G -> T	249	SNP (transversion)	34.90%	########		T -> K	hypothetical protein CDS	hypothetical protein	Substituti on	KLI717 69.1
Variants: VaAres1_B_FDSW210055061-2r	T	131	131	1	G -> T	291	SNP (transversion)	38.10%	########		T -> K	hypothetical protein CDS	hypothetical protein	Substituti on	KLI717 69.1
Variants: VaAres1_C_FDSW210055062-1r	T	131	131	1	G -> T	397	SNP (transversion)	28.20%	########		T -> K	hypothetical protein CDS	hypothetical protein	Substituti on	KLI717 69.1
Variants: VaphiVaphiSt2_B_A_FDSW2100550 54-2r	T	131	131	1	G -> T	239	SNP (transversion)	45.20%	########		T -> K	hypothetical protein CDS	hypothetical protein	Substituti on	KLI717 69.1

Variants: VaphiSt2_B_FDSW210055056-1r	T	131	131	1	G -> T	485	SNP (transversion)	34.60%	0	T -> K	hypothetical protein CDS	hypothetical protein	Substitution	KLI71769.1
Variants: VaAphrodite_A_FDSW210055057-2r	A	136	136	1	G -> A	356	SNP (transition)	25.60%	########		hypothetical protein CDS	hypothetical protein	None	KLI71769.1
Variants: VaAphrodite_A_FDSW210055057-2r	C	175	175	1	T -> C	235	SNP (transition)	45.10%	########		hypothetical protein CDS	hypothetical protein	None	KLI71769.1
Variants: VaAphrodite_B_FDSW210055058-2r	C	175	175	1	T -> C	199	SNP (transition)	43.70%	########		hypothetical protein CDS	hypothetical protein	None	KLI71769.1
Variants: VaAphrodite_C_FDSW210055059-1r	C	175	175	1	T -> C	253	SNP (transition)	45.50%	########		hypothetical protein CDS	hypothetical protein	None	KLI71769.1
Variants: VaAres1_A_FDSW210055060-2r	C	175	175	1	T -> C	158	SNP (transition)	39.90%	########		hypothetical protein CDS	hypothetical protein	None	KLI71769.1
Variants: VaAres1_B_FDSW210055061-2r	C	175	175	1	T -> C	205	SNP (transition)	46.80%	########		hypothetical protein CDS	hypothetical protein	None	KLI71769.1
Variants: VaAres1_C_FDSW210055062-1r	C	175	175	1	T -> C	243	SNP (transition)	40.70%	########		hypothetical protein CDS	hypothetical protein	None	KLI71769.1
Variants: VaphiVaphiSt2_B_A_FDSW210055054-2r	C	175	175	1	T -> C	144	SNP (transition)	49.30%	########		hypothetical protein CDS	hypothetical protein	None	KLI71769.1
Variants: VaphiSt2_B_FDSW210055056-1r	C	175	175	1	T -> C	307	SNP (transition)	40.40%	########		hypothetical protein CDS	hypothetical protein	None	KLI71769.1
Variants: VaAphrodite_A_FDSW210055057-2r	GCG	180	182	3	AGC -> GCG	210 -> 211	Substitution	26.1% -> 26.2%	########	SS -> TP	hypothetical protein CDS	hypothetical protein	Substitution	KLI71769.1
Variants: VaAphrodite_B_FDSW210055058-2r	GCG	180	182	3	AGC -> GCG	175 -> 185	Substitution	24.9% -> 26.3%	1.10E-96	SS -> TP	hypothetical protein CDS	hypothetical protein	Substitution	KLI71769.1
Variants: VaAphrodite_C_FDSW210055059-1r	GCG	180	182	3	AGC -> GCG	228 -> 233	Substitution	28.3% -> 28.9%	########	SS -> TP	hypothetical protein CDS	hypothetical protein	Substitution	KLI71769.1
Variants: VaAres1_A_FDSW210055060-2r	GC	180	181	2	AG -> GC	143 -> 147	Substitution	35.7% -> 36.1%	########	SS -> RP	hypothetical protein CDS	hypothetical protein	Substitution	KLI71769.1
Variants: VaAres1_B_FDSW210055061-2r	GCG	180	182	3	AGC -> GCG	178 -> 183	Substitution	30.6% -> 31.5%	########	SS -> TP	hypothetical protein CDS	hypothetical protein	Substitution	KLI71769.1
Variants: VaAres1_C_FDSW210055062-1r	GCG	180	182	3	AGC -> GCG	215 -> 222	Substitution	27.9% -> 28.8%	########	SS -> TP	hypothetical protein CDS	hypothetical protein	Substitution	KLI71769.1
Variants: VaphiVaphiSt2_B_A_FDSW210055054-2r	GCG	180	182	3	AGC -> GCG	127 -> 129	Substitution	34.9% -> 35.4%	########	SS -> TP	hypothetical protein CDS	hypothetical protein	Substitution	KLI71769.1
Variants: VaphiSt2_B_FDSW210055056-1r	GCG	180	182	3	AGC -> GCG	286 -> 293	Substitution	25.9% -> 26.6%	########	SS -> TP	hypothetical protein CDS	hypothetical protein	Substitution	KLI71769.1
Variants: VaAphrodite_A_FDSW210055057-2r	G	190	190	1	C -> G	195	SNP (transversion)	32.30%	########	E -> D	hypothetical protein CDS	hypothetical protein	Substitution	KLI71769.1
Variants: VaAphrodite_B_FDSW210055058-2r	G	190	190	1	C -> G	158	SNP (transversion)	35.40%	########	E -> D	hypothetical protein CDS	hypothetical protein	Substitution	KLI71769.1
Variants: VaAphrodite_C_FDSW210055059-1r	G	190	190	1	C -> G	199	SNP (transversion)	33.70%	########	E -> D	hypothetical protein CDS	hypothetical protein	Substitution	KLI71769.1
Variants: VaAres1_A_FDSW210055060-2r	G	190	190	1	C -> G	127	SNP (transversion)	26.00%	1.10E-62	E -> D	hypothetical protein CDS	hypothetical protein	Substitution	KLI71769.1
Variants: VaAres1_B_FDSW210055061-2r	G	190	190	1	C -> G	161	SNP (transversion)	37.90%	########	E -> D	hypothetical protein CDS	hypothetical protein	Substitution	KLI71769.1
Variants: VaAres1_C_FDSW210055062-1r	G	190	190	1	C -> G	184	SNP (transversion)	28.80%	########	E -> D	hypothetical protein CDS	hypothetical protein	Substitution	KLI71769.1
Variants: VaphiVaphiSt2_B_A_FDSW210055054-2r	G	190	190	1	C -> G	101	SNP (transversion)	42.60%	1.20E-96	E -> D	hypothetical protein CDS	hypothetical protein	Substitution	KLI71769.1
Variants: VaphiSt2_B_FDSW210055056-1r	G	190	190	1	C -> G	256	SNP (transversion)	32.00%	########	E -> D	hypothetical protein CDS	hypothetical protein	Substitution	KLI71769.1
Variants: VaAphrodite_A_FDSW210055057-2r	A	193	193	1	T -> A	181	SNP (transversion)	28.70%	2.80E-95		hypothetical protein CDS	hypothetical protein	None	KLI71769.1
Variants: VaAphrodite_B_FDSW210055058-2r	A	193	193	1	T -> A	148	SNP (transversion)	33.80%	########		hypothetical protein CDS	hypothetical protein	None	KLI71769.1
Variants: VaAphrodite_C_FDSW210055059-1r	A	193	193	1	T -> A	188	SNP (transversion)	30.90%	########		hypothetical protein CDS	hypothetical protein	None	KLI71769.1
Variants: VaAres1_A_FDSW210055060-2r	A	193	193	1	T -> A	124	SNP (transversion)	25.00%	2.20E-58		hypothetical protein CDS	hypothetical protein	None	KLI71769.1

Variants: VaAres1_B_FDSW210055061-2r	A	193	193	1	T -> A	151	SNP (transversion)	34.40%	########		hypothetical protein CDS	hypothetical protein	None	KLI71769.1
Variants: VaAres1_C_FDSW210055062-1r	A	193	193	1	T -> A	179	SNP (transversion)	27.40%	1.50E-93		hypothetical protein CDS	hypothetical protein	None	KLI71769.1
Variants: VaphiVaphiSt2_B_A_FDSW210055054-2r	A	193	193	1	T -> A	102	SNP (transversion)	40.20%	6.60E-91		hypothetical protein CDS	hypothetical protein	None	KLI71769.1
Variants: VaphiSt2_B_FDSW210055056-1r	A	193	193	1	T -> A	244	SNP (transversion)	29.10%	########		hypothetical protein CDS	hypothetical protein	None	KLI71769.1
Variants: VaAphrodite_B_FDSW210055058-2r	C	198	198	1	T -> C	135	SNP (transition)	29.60%	2.80E-82	I -> V	hypothetical protein CDS	hypothetical protein	Substitution	KLI71769.1
Variants: VaAphrodite_C_FDSW210055059-1r	C	198	198	1	T -> C	175	SNP (transition)	26.90%	########	I -> V	hypothetical protein CDS	hypothetical protein	Substitution	KLI71769.1
Variants: VaAres1_B_FDSW210055061-2r	C	198	198	1	T -> C	137	SNP (transition)	31.40%	2.60E-85	I -> V	hypothetical protein CDS	hypothetical protein	Substitution	KLI71769.1
Variants: VaphiVaphiSt2_B_A_FDSW210055054-2r	C	198	198	1	T -> C	95	SNP (transition)	33.70%	4.50E-65	I -> V	hypothetical protein CDS	hypothetical protein	Substitution	KLI71769.1
Variants: VaphiSt2_B_FDSW210055056-1r	C	198	198	1	T -> C	227	SNP (transition)	27.30%	########	I -> V	hypothetical protein CDS	hypothetical protein	Substitution	KLI71769.1
Variants: VaAphrodite_B_FDSW210055058-2r	C	201	201	1	T -> C	126	SNP (transition)	25.40%	1.80E-60	T -> A	hypothetical protein CDS	hypothetical protein	Substitution	KLI71769.1
Variants: VaAres1_B_FDSW210055061-2r	C	201	201	1	T -> C	126	SNP (transition)	28.60%	1.50E-73	T -> A	hypothetical protein CDS	hypothetical protein	Substitution	KLI71769.1
Variants: VaphiVaphiSt2_B_A_FDSW210055054-2r	C	201	201	1	T -> C	92	SNP (transition)	28.30%	2.10E-53	T -> A	hypothetical protein CDS	hypothetical protein	Substitution	KLI71769.1
Variants: VaAres1_B_FDSW210055061-2r	A	34442	34442	1	C -> A	51	SNP (transversion)	25.50%	9.90E-07					
Variants: VaphiVaphiSt2_B_A_FDSW210055054-2r	A	45182	45182	1	C -> A	104	SNP (transversion)	26.00%	5.10E-15	M -> I	prpE CDS	propionyl-CoA synthetase	Substitution	KLI71805.1

Contig 15

Variant	Name	Minimum	Maximum	Length	Change	Coverage	Polymorphism Type	Variant Frequency	Variant P-Value (approximate)	Amino Acid Change	CDS	product	Protein Effect	protein_id
Variants: VaAres1_A_FDSW210055060-2r	T	64122	64122	1	G -> T	120	SNP (transversion)	96.70%	0	Q -> H	transcriptional regulator CDS	transcriptional regulator	Substitution	KLI71554.1
Variants: VaAres1_C_FDSW210055062-1r	T	64122	64122	1	G -> T	190	SNP (transversion)	96.80%	0	Q -> H	transcriptional regulator CDS	transcriptional regulator	Substitution	KLI71554.1
Variants: VaAres1_A_FDSW210055060-2r	A	76493	76493	1	C -> A	103	SNP (transversion)	27.20%	5.40E-09					
Variants: VaphiVaphiSt2_B_A_FDSW210055054-2r	A	131308	131308	1	T -> A	104	SNP (transversion)	26.90%	5.80E-16	K -> N	membrane protein CDS	membrane protein	Substitution	KLI71610.1

Contig 16

Variant	Name	Minimum	Maximum	Length	Change	Coverage	Polymorphism Type	Variant Frequency	Variant P-Value (approximate)	CDS	product	Protein Effect	protein_id
Variants: VaphiVaphiSt2_B_A_FDSW210055054-2r	CTGACGTCATC	48427	48426	0	#NAME?	154 -> 158	Insertion	100.00%	0	chemotaxis protein CheY CDS	chemotaxis protein CheY	Frame Shift	KLI71478.1
Variants: VaphiSt2_B_FDSW210055056-1r	CTGACGTCATC	48427	48426	0	#NAME?	289	Insertion	29.80%	########	chemotaxis protein CheY CDS	chemotaxis protein CheY	Frame Shift	KLI71478.1

Contig 17

Variant	Name	Minimum	Maximum	Length	Change	Coverage	Polymorphism Type	Variant Frequency	Variant P-Value (approximate)	CDS	product	Protein Effect	protein_id	Amino Acid Change
Variants: VaAphrodite_A_FDSW210055057-2r	G	22	22	1	A -> G	485	SNP (transition)	38.10%	0					
Variants: VaAphrodite_B_FDSW210055058-2r	G	22	22	1	A -> G	422	SNP (transition)	37.90%	0					
Variants: VaAphrodite_C_FDSW210055059-1r	G	22	22	1	A -> G	523	SNP (transition)	39.00%	0					
Variants: VaAres1_A_FDSW210055060-2r	G	22	22	1	A -> G	261	SNP (transition)	51.00%	2.00E-269					
Variants: VaAres1_B_FDSW210055061-2r	G	22	22	1	A -> G	289	SNP (transition)	39.40%	8.20E-226					
Variants: VaAres1_C_FDSW210055062-1r	G	22	22	1	A -> G	382	SNP (transition)	41.10%	0					
Variants: VaphiVaphiSt2_B_A_FDSW210055054-2r	G	22	22	1	A -> G	318	SNP (transition)	41.50%	3.00E-291					
Variants: VaphiSt2_B_FDSW210055056-1r	G	22	22	1	A -> G	591	SNP (transition)	35.90%	0					
Variants: VaAphrodite_A_FDSW210055057-2r	A	36	36	1	G -> A	547	SNP (transition)	31.10%	2.9E-314					
Variants: VaAphrodite_B_FDSW210055058-2r	A	36	36	1	G -> A	488	SNP (transition)	34.40%	0					
Variants: VaAphrodite_C_FDSW210055059-1r	A	36	36	1	G -> A	575	SNP (transition)	35.80%	0					
Variants: VaAres1_A_FDSW210055060-2r	A	36	36	1	G -> A	275	SNP (transition)	40.40%	3.90E-177					
Variants: VaAres1_B_FDSW210055061-2r	A	36	36	1	G -> A	324	SNP (transition)	39.50%	2.00E-253					
Variants: VaAres1_C_FDSW210055062-1r	A	36	36	1	G -> A	447	SNP (transition)	37.60%	0					
Variants: VaphiVaphiSt2_B_A_FDSW210055054-2r	A	36	36	1	G -> A	341	SNP (transition)	38.10%	5.20E-216					
Variants: VaphiSt2_B_FDSW210055056-1r	A	36	36	1	G -> A	674	SNP (transition)	35.50%	0					
Variants: VaAphrodite_A_FDSW210055057-2r	C	72	73	2	AT -> C	587 -> 588	Deletion	32.3% -> 32.4%	3.40E-101					
Variants: VaAphrodite_B_FDSW210055058-2r	C	72	73	2	AT -> C	539 -> 543	Deletion	28.4% -> 28.6%	2.20E-78					
Variants: VaAphrodite_C_FDSW210055059-1r	C	72	73	2	AT -> C	639 -> 640	Deletion	28.1% -> 28.2%	9.10E-85					
Variants: VaAres1_B_FDSW210055061-2r	C	72	73	2	AT -> C	361 -> 364	Deletion	31.3% -> 31.6%	2.50E-61					
Variants: VaAres1_C_FDSW210055062-1r	C	72	73	2	AT -> C	502 -> 504	Deletion	28.2% -> 28.3%	8.10E-71					
Variants: VaphiVaphiSt2_B_A_FDSW210055054-2r	C	72	73	2	AT -> C	376	Deletion	29.80%	1.10E-47					
Variants: VaphiSt2_B_FDSW210055056-1r	C	72	73	2	AT -> C	745 -> 749	Deletion	32.0% -> 32.2%	2.90E-128					
Variants: VaAres1_A_FDSW210055060-2r	T	77	77	1	C -> T	221	SNP (transition)	39.40%	2.40E-181					
Variants: VaAres1_B_FDSW210055061-2r	T	77	77	1	C -> T	368	SNP (transition)	25.30%	1.50E-181					
Variants: VaAres1_C_FDSW210055062-1r	T	77	77	1	C -> T	514	SNP (transition)	25.90%	3.70E-247					
Variants: VaphiVaphiSt2_B_A_FDSW210055054-2r	T	77	77	1	C -> T	375	SNP (transition)	25.10%	2.00E-136					
Variants: VaphiSt2_B_FDSW210055056-1r		17255	17266	12	#NAME?	254 -> 258	Deletion	64.3% -> 65.4%	5.10E-212	membrane protein CDS	membrane protein	Deletion	KLI71254.1	LTHTN -> H

Variants:<br>VaAphrodite_C_FDSW210055059-1r	T	21847	21847	1	G -> T	240	SNP<br>(transversion)	56.30%	0	DNA-binding<br>protein CDS	DNA-binding<br>protein	Substituti<br>on	KLI712<br>57.1	Q -> K
Variants:<br>VaAres1_B_FDSW210055061-2r	A	10495<br>4	10495<br>4	1	C -> A	94	SNP<br>(transversion)	26.60%	1.50E-16	type II citrate<br>synthase CDS	type II citrate<br>synthase	Substituti<br>on	KLI713<br>27.1	Q -> K
Variants:<br>VaAphrodite_A_FDSW210055057-2r	A	21684<br>9	21684<br>8	0	#NAME?	402	Insertion	46.00%	0					
Variants:<br>VaAphrodite_B_FDSW210055058-2r	A	21684<br>9	21684<br>8	0	#NAME?	172	Insertion	99.40%	0					
Variants:<br>VaAphrodite_C_FDSW210055059-1r	A	21684<br>9	21684<br>8	0	#NAME?	228	Insertion	99.60%	0					
Variants:<br>VaAres1_A_FDSW210055060-2r	A	21684<br>9	21684<br>8	0	#NAME?	244	Insertion	60.20%	0					
Variants:<br>VaAres1_B_FDSW210055061-2r	A	21684<br>9	21684<br>8	0	#NAME?	152	Insertion	99.30%	0					
Variants:<br>VaAres1_C_FDSW210055062-1r	A	21684<br>9	21684<br>8	0	#NAME?	324	Insertion	53.40%	0					
Variants:<br>VaphiVaphiSt2_B_A_FDSW2100550<br>54-2r	A	21684<br>9	21684<br>8	0	#NAME?	120	Insertion	99.20%	0					
Variants:<br>VaphiSt2_B_FDSW210055056-1r	A	21684<br>9	21684<br>8	0	#NAME?	269	Insertion	97.00%	0					
Variants:<br>VaAphrodite_A_FDSW210055057-2r	T	21685<br>0	21685<br>0	1	A -> T	403	SNP<br>(transversion)	50.60%	0					
Variants:<br>VaAphrodite_B_FDSW210055058-2r	T	21685<br>0	21685<br>0	1	A -> T	172	SNP<br>(transversion)	98.30%	0					
Variants:<br>VaAphrodite_C_FDSW210055059-1r	T	21685<br>0	21685<br>0	1	A -> T	228	SNP<br>(transversion)	100.00%	0					
Variants:<br>VaAres1_A_FDSW210055060-2r	T	21685<br>0	21685<br>0	1	A -> T	244	SNP<br>(transversion)	61.10%	0					
Variants:<br>VaAres1_B_FDSW210055061-2r	T	21685<br>0	21685<br>0	1	A -> T	157	SNP<br>(transversion)	100.00%	0					
Variants:<br>VaAres1_C_FDSW210055062-1r	T	21685<br>0	21685<br>0	1	A -> T	316	SNP<br>(transversion)	55.40%	0					
Variants:<br>VaphiVaphiSt2_B_A_FDSW2100550<br>54-2r	T	21685<br>0	21685<br>0	1	A -> T	121	SNP<br>(transversion)	100.00%	0					
Variants:<br>VaphiSt2_B_FDSW210055056-1r	T	21685<br>0	21685<br>0	1	A -> T	269	SNP<br>(transversion)	97.40%	0					
Variants:<br>VaAphrodite_A_FDSW210055057-2r	GT<br>C	21685<br>6	21685<br>8	3	TAG -><br>GTC	403 -><br>406	Substitution	39.7% -><br>40.0%	0					
Variants:<br>VaAres1_A_FDSW210055060-2r	GT<br>C	21685<br>6	21685<br>8	3	TAG -><br>GTC	242 -><br>245	Substitution	32.7% -><br>33.1%	2.50E-205					
Variants:<br>VaAres1_C_FDSW210055062-1r	GT<br>C	21685<br>6	21685<br>8	3	TAG -><br>GTC	322 -><br>323	Substitution	34.1% -><br>34.2%	2.50E-278					
Variants:<br>VaAphrodite_A_FDSW210055057-2r	A	21686<br>1	21686<br>1	1	T -> A	404	SNP<br>(transversion)	47.00%	0					
Variants:<br>VaAphrodite_B_FDSW210055058-2r	A	21686<br>1	21686<br>1	1	T -> A	170	SNP<br>(transversion)	99.40%	0					
Variants:<br>VaAphrodite_C_FDSW210055059-1r	A	21686<br>1	21686<br>1	1	T -> A	224	SNP<br>(transversion)	100.00%	0					
Variants:<br>VaAres1_A_FDSW210055060-2r	A	21686<br>1	21686<br>1	1	T -> A	239	SNP<br>(transversion)	59.00%	0					
Variants:<br>VaAres1_B_FDSW210055061-2r	A	21686<br>1	21686<br>1	1	T -> A	164	SNP<br>(transversion)	100.00%	0					
Variants:<br>VaAres1_C_FDSW210055062-1r	A	21686<br>1	21686<br>1	1	T -> A	321	SNP<br>(transversion)	53.60%	0					
Variants:<br>VaphiVaphiSt2_B_A_FDSW2100550<br>54-2r	A	21686<br>1	21686<br>1	1	T -> A	121	SNP<br>(transversion)	100.00%	0					
Variants:<br>VaphiSt2_B_FDSW210055056-1r	A	21686<br>1	21686<br>1	1	T -> A	292	SNP<br>(transversion)	98.30%	0					
Variants:<br>VaAphrodite_A_FDSW210055057-2r	T	21686<br>4	21686<br>4	1	G -> T	404	SNP<br>(transversion)	49.80%	0					

Variants: VaAres1_A_FDSW210055060-2r	T	21686 4	21686 4	1	G -> T	240	SNP (transversion)	39.60%	4.60E-255
Variants: VaAres1_C_FDSW210055062-1r	T	21686 4	21686 4	1	G -> T	324	SNP (transversion)	44.40%	0
Variants: VaAphrodite_A_FDSW210055057-2r	A	21686 5	21686 5	1	T -> A	403	SNP (transversion)	38.70%	0
Variants: VaAres1_A_FDSW210055060-2r	A	21686 5	21686 5	1	T -> A	239	SNP (transversion)	33.50%	7.70E-224
Variants: VaAres1_C_FDSW210055062-1r	A	21686 5	21686 5	1	T -> A	324	SNP (transversion)	34.60%	8.50E-270
Variants: VaAphrodite_A_FDSW210055057-2r	T	21686 6	21686 6	1	G -> T	405	SNP (transversion)	50.40%	0
Variants: VaAres1_A_FDSW210055060-2r	T	21686 6	21686 6	1	G -> T	237	SNP (transversion)	39.20%	5.40E-240
Variants: VaAres1_C_FDSW210055062-1r	T	21686 6	21686 6	1	G -> T	324	SNP (transversion)	44.80%	0
Variants: VaAphrodite_A_FDSW210055057-2r	TA G	21686 7	21686 9	3	ACT -> TAG	404 -> 405	Substitution	38.5% -> 38.6%	0
Variants: VaAres1_A_FDSW210055060-2r	TA G	21686 7	21686 9	3	ACT -> TAG	231 -> 235	Substitution	33.2% -> 33.8%	3.60E-206
Variants: VaAres1_C_FDSW210055062-1r	TA G	21686 7	21686 9	3	ACT -> TAG	323 -> 325	Substitution	32.6% -> 33.1%	3.10E-261
Variants: VaAphrodite_A_FDSW210055057-2r	G	21687 1	21687 1	1	T -> G	409	SNP (transversion)	49.10%	0
Variants: VaAres1_A_FDSW210055060-2r	G	21687 1	21687 1	1	T -> G	230	SNP (transversion)	40.40%	8.00E-251
Variants: VaAres1_C_FDSW210055062-1r	G	21687 1	21687 1	1	T -> G	328	SNP (transversion)	43.60%	0
Variants: VaAphrodite_A_FDSW210055057-2r	CA A	21687 2	21687 5	4	TGCC -> CAA	406 -> 409	Deletion	38.4% -> 38.7%	8.50E-113
Variants: VaAres1_A_FDSW210055060-2r	CA A	21687 2	21687 5	4	TGCC -> CAA	231 -> 236	Deletion	32.6% -> 33.3%	2.60E-57
Variants: VaAres1_C_FDSW210055062-1r	CA A	21687 2	21687 5	4	TGCC -> CAA	330 -> 334	Deletion	31.7% -> 32.1%	2.20E-58
Variants: VaAphrodite_A_FDSW210055057-2r	T	21687 8	21687 8	1	C -> T	406	SNP (transition)	52.00%	0
Variants: VaAres1_A_FDSW210055060-2r	T	21687 8	21687 8	1	C -> T	238	SNP (transition)	40.30%	1.00E-258
Variants: VaAres1_C_FDSW210055062-1r	T	21687 8	21687 8	1	C -> T	329	SNP (transition)	45.00%	0
Variants: VaAphrodite_A_FDSW210055057-2r	G	21688 2	21688 2	1	T -> G	403	SNP (transversion)	52.40%	0
Variants: VaAres1_A_FDSW210055060-2r	G	21688 2	21688 2	1	T -> G	243	SNP (transversion)	39.50%	5.00E-248
Variants: VaAres1_C_FDSW210055062-1r	G	21688 2	21688 2	1	T -> G	325	SNP (transversion)	45.80%	0
Variants: VaAphrodite_A_FDSW210055057-2r	C	21694 6	21694 6	1	A -> C	299	SNP (transversion)	39.50%	2.20E-304
Variants: VaAres1_A_FDSW210055060-2r	C	21694 6	21694 6	1	A -> C	233	SNP (transversion)	39.50%	6.20E-247
Variants: VaAres1_C_FDSW210055062-1r	C	21694 6	21694 6	1	A -> C	315	SNP (transversion)	42.90%	0
Variants: VaAres1_C_FDSW210055062-1r		21695 5	21695 5	1	#NAME?	263	Deletion	26.20%	2.10E-42
Variants: VaAres1_C_FDSW210055062-1r	A	21695 8	21695 8	1	T -> A	249	SNP (transversion)	28.90%	1.40E-174
Variants: VaAphrodite_A_FDSW210055057-2r	G	21710 9	21710 9	1	A -> G	306	SNP (transition)	59.80%	0
Variants: VaAphrodite_B_FDSW210055058-2r	G	21710 9	21710 9	1	A -> G	282	SNP (transition)	55.70%	0
Variants: VaAphrodite_C_FDSW210055059-1r	G	21710 9	21710 9	1	A -> G	479	SNP (transition)	54.90%	0
Variants: VaAres1_A_FDSW210055060-2r	G	21710 9	21710 9	1	A -> G	191	SNP (transition)	57.60%	0

Variants:<br>VaAres1_B_FDSW210055061-2r	G	21710<br>9	21710<br>9	1	A -> G	257	SNP<br>(transition)	56.80%		0
Variants:<br>VaAres1_C_FDSW210055062-1r	G	21710<br>9	21710<br>9	1	A -> G	343	SNP<br>(transition)	55.70%		0
Variants:<br>VaphiVaphiSt2_B_A_FDSW2100550<br>54-2r	G	21710<br>9	21710<br>9	1	A -> G	184	SNP<br>(transition)	57.10%	7.1E-315	
Variants:<br>VaphiSt2_B_FDSW210055056-1r	G	21710<br>9	21710<br>9	1	A -> G	484	SNP<br>(transition)	60.70%		0

Contig 18

Variant	Name	Minimum	Maximum	Length	Change	Coverage	Polymorphism Type	Variant Frequency	Variant P-Value (approximate)
Variants: VaphiVaphiSt2_B_A_FDSW210055054-2r	A	70559	70559	1	C -> A	61	SNP (transversion)	31.10%	7.70E-11
Variants: VaphiVaphiSt2_B_A_FDSW210055054-2r	T	70568	70568	1	G -> T	63	SNP (transversion)	30.20%	2.50E-07
Variants: VaphiSt2_B_FDSW210055056-1r		96521	96521	1	(T)7 -> (T)6	177	Deletion (tandem repeat)	26.00%	7.30E-33

Contig 19

Variant	Name	Minimum	Maximum	Length	Change	Coverage	Polymorphism Type	Variant Frequency	Variant P-Value (approximate)	Amino Acid Change	CDS	product	Protein Effect	protein_id
Variants: VaAphrodite_A_FDSW210055057-2r	A	10530	10530	1	G -> A	235	SNP (transition)	29.80%	7.80E-185		RNA polymerase sigma factor RpoS CDS	RNA polymerase sigma factor RpoS	Truncation	KLI71089.1
Variants: VaAphrodite_B_FDSW210055058-2r		10830	10831	2	#NAME?	187	Deletion	73.80%	1.60E-176		RNA polymerase sigma factor RpoS CDS	RNA polymerase sigma factor RpoS	Frame Shift	KLI71089.1
Variants: VaAres1_C_FDSW210055062-1r	A	10548	10548	1	T -> A	230	SNP (transversion)	98.70%	0	I -> F	RNA polymerase sigma factor RpoS CDS	RNA polymerase sigma factor RpoS	Substitution	KLI71089.1

Contig 21

Variant	Name	Minimum	Maximum	Length	Change	Coverage	Polymorphism Type	Variant Frequency	Variant P-Value (approximate)
Variants: VaAphrodite_A_FDSW210055057-2r	T	107595	107595	1	A -> T	115	SNP (transversion)	25.20%	1.80E-08
Variants: VaAphrodite_B_FDSW210055058-2r	T	107591	107591	1	G -> T	104	SNP (transversion)	25.00%	4.30E-14
Variants: VaAphrodite_B_FDSW210055058-2r	A	107604	107604	1	C -> A	107	SNP (transversion)	26.20%	1.00E-20
Variants: VaAres1_A_FDSW210055060-2r	T	107601	107601	1	G -> T	99	SNP (transversion)	26.30%	3.40E-10
Variants: VaAres1_B_FDSW210055061-2r	T	107585	107585	1	G -> T	99	SNP (transversion)	25.30%	5.90E-16
Variants: VaphiVaphiSt2_B_A_FDSW210055054-2r	T	107591	107591	1	G -> T	79	SNP (transversion)	27.80%	2.60E-19
Variants: VaphiVaphiSt2_B_A_FDSW210055054-2r	T	107601	107601	1	G -> T	79	SNP (transversion)	30.40%	4.80E-13
Variants: VaphiVaphiSt2_B_A_FDSW210055054-2r	A	107604	107604	1	C -> A	81	SNP (transversion)	25.90%	9.80E-07

Contig 22

Variant	Name	Minimum	Maximum	Length	Change	Coverage	Polymorphism Type	Variant Frequency	Variant P-Value (approximate)	Amino Acid Change	CDS	product	Protein Effect	protein_id
Variants: VaAres1_A_FDSW210055060-2r	A	13788	13788	1	C -> A	94	SNP (transversion)	27.70%	3.00E-15	R -> I	hypothetical protein CDS	hypothetical protein	Substitution	KLI70757.1
Variants: VaAres1_A_FDSW210055060-2r	A	13803	13803	1	C -> A	101	SNP (transversion)	25.70%	3.70E-12	G -> V	hypothetical protein CDS	hypothetical protein	Substitution	KLI70757.1
Variants: VaphiVaphiSt2_B_A_FDSW210055054-2r	T	2133	2133	1	G -> T	73	SNP (transversion)	27.40%	3.20E-10	C -> F	hypothetical protein CDS	hypothetical protein	Substitution	KLI70750.1
Variants: VaphiVaphiSt2_B_A_FDSW210055054-2r	A	13788	13788	1	C -> A	82	SNP (transversion)	32.90%	4.40E-13	R -> I	hypothetical protein CDS	hypothetical protein	Substitution	KLI70757.1
Variants: VaphiVaphiSt2_B_A_FDSW210055054-2r	A	13803	13803	1	C -> A	80	SNP (transversion)	28.70%	4.70E-14	G -> V	hypothetical protein CDS	hypothetical protein	Substitution	KLI70757.1
Variants: VaphiVaphiSt2_B_A_FDSW210055054-2r	A	13810	13810	1	T -> A	82	SNP (transversion)	25.60%	2.70E-08	M -> L	hypothetical protein CDS	hypothetical protein	Substitution	KLI70757.1
Variants: VaphiVaphiSt2_B_A_FDSW210055054-2r	T	13822	13822	1	G -> T	84	SNP (transversion)	28.60%	1.00E-16	Q -> K	hypothetical protein CDS	hypothetical protein	Substitution	KLI70757.1

Contig 23

Variant	Name	Minimum	Maximum	Length	Change	Coverage	Polymorphism Type	Variant Frequency	Variant P-Value (approximate)
Variants: VaAres1_A_FDSW210055060-2r	CT	2	3	2	TA -> CT	127	Substitution	34.60%	1.70E-111
Variants: VaAres1_A_FDSW210055060-2r	T	6	6	1	G -> T	132	SNP (transversion)	34.80%	5.00E-112

Contig 24

Variant	Name	Minimum	Maximum	Length	Change	Coverage	Polymorphism Type	Variant Frequency	Variant P-Value (approximate)	CDS	product	Protein Effect	protein_id	Amino Acid Change
Variants: VaAphrodite_A_FDSW210055057-2r	T	74	74	1	C -> T	586	SNP (transition)	47.80%	0					
Variants: VaAphrodite_A_FDSW210055057-2r	C	199875	199875	1	A -> C	214	SNP (transversion)	99.50%	0	UDP-phosphate N-acetylglucosaminyl 1-phosphate transferase CDS	UDP-phosphate N-acetylglucosaminyl 1-phosphate transferase	Substitution	KLI70639.1	M -> R
Variants: VaAphrodite_B_FDSW210055058-2r	T	74	74	1	C -> T	530	SNP (transition)	50.60%	0					
Variants: VaAphrodite_B_FDSW210055058-2r	CGCATGAG	200585	200584	0	#NAME?	236 -> 240	Insertion	98.7% -> 99.2%	0	UDP-phosphate N-acetylglucosaminyl 1-phosphate transferase CDS	UDP-phosphate N-acetylglucosaminyl 1-phosphate transferase	Frame Shift	KLI70639.1	
Variants: VaAphrodite_C_FDSW210055059-1r	T	74	74	1	C -> T	529	SNP (transition)	59.00%	0					
Variants: VaAphrodite_C_FDSW210055059-1r		189238	189241	4	(AAAG)3 -> (AAAG)2	222	Deletion (tandem repeat)	100.00%	0	hypothetical protein CDS	hypothetical protein	Frame Shift	KLI70632.1	
Variants: VaAres1_A_FDSW210055060-2r	T	74	74	1	C -> T	360	SNP (transition)	49.70%	2.90E-270					
Variants: VaAres1_A_FDSW210055060-2r	A	75750	75750	1	C -> A	131	SNP (transversion)	26.70%	1.90E-19					
Variants: VaAres1_A_FDSW210055060-2r		199111	199112	2	#NAME?	122	Deletion	100.00%	6.30E-196	hypothetical protein CDS	hypothetical protein	Frame Shift	KLI70638.1	
Variants: VaAres1_B_FDSW210055061-2r	T	74	74	1	C -> T	246	SNP (transition)	57.30%	9.40E-282					
Variants: VaAres1_B_FDSW210055061-2r	C	199878	199878	1	T -> C	177	SNP (transition)	100.00%	0	UDP-phosphate N-acetylglucosaminyl 1-phosphate transferase CDS	UDP-phosphate N-acetylglucosaminyl 1-phosphate transferase	Substitution	KLI70639.1	D -> G
Variants: VaAres1_C_FDSW210055062-1r	T	74	74	1	C -> T	502	SNP (transition)	50.20%	0					
Variants: VaAres1_C_FDSW210055062-1r		199111	199112	2	#NAME?	155	Deletion	100.00%	1.00E-248	hypothetical protein CDS	hypothetical protein	Frame Shift	KLI70638.1	
Variants: VaphiVaphiSt2_B_A_FDSW210055054-2r	T	74	74	1	C -> T	330	SNP (transition)	48.20%	7.10E-206					
Variants: VaphiSt2_B_FDSW210055056-1r	T	74	74	1	C -> T	824	SNP (transition)	47.00%	0					

Contig 25

Variant	Name	Minimum	Maximum	Length	Change	Coverage	Polymorphism Type	Variant Frequency	Variant P-Value (approximate)	CDS	product	Protein Effect	protein_id	Amino Acid Change
Variants: VaAphrodite_B_FDSW210055058-2r	A	8289	8288	0	(A)3 -> (A)4	185	Insertion (tandem repeat)	27.60%	5.00E-128	pheA CDS	chorismate mutase	Frame Shift	KLI70447.1	
Variants: VaAphrodite_B_FDSW210055058-2r	C	25651	25651	1	T -> C	210	SNP (transition)	29.50%	5.10E-176	histidine kinase CDS	histidine kinase	Substitution	KLI70462.1	Y -> H

Contig 27

Variant	Name	Minimum	Maximum	Length	Change	Coverage	Polymorphism Type	Variant Frequency	Variant P-Value (approximate)	CDS	product	Protein Effect	protein_id	Amino Acid Change
Variants: VaAphrodite_A_FDSW210055057-2r	AG	27267	27268	2	TA -> AG	170 -> 186	Substitution	24.7% -> 27.1%	1.40E-90	hypothetical protein CDS	hypothetical protein	Substitution	KLI70436.1	L -> Q
Variants: VaAphrodite_A_FDSW210055057-2r	C	27269	27269	1	A -> C	196	SNP (transversion)	37.80%	1.40E-167	hypothetical protein CDS	hypothetical protein	Substitution	KLI70436.1	K -> Q
Variants: VaAphrodite_B_FDSW210055058-2r	AGC	27267	27269	3	TAA -> AGC	145 -> 163	Substitution	23.4% -> 33.1%	1.40E-79	hypothetical protein CDS	hypothetical protein	Substitution	KLI70436.1	LK -> QQ
Variants: VaAphrodite_C_FDSW210055059-1r	C	27269	27269	1	A -> C	263	SNP (transversion)	29.30%	5.10E-164	hypothetical protein CDS	hypothetical protein	Substitution	KLI70436.1	K -> Q
Variants: VaAres1_A_FDSW210055060-2r	AGC	27267	27269	3	TAA -> AGC	109 -> 127	Substitution	21.1% -> 30.7%	4.90E-60	hypothetical protein CDS	hypothetical protein	Substitution	KLI70436.1	LK -> QQ
Variants: VaAres1_B_FDSW210055061-2r	C	27269	27269	1	A -> C	135	SNP (transversion)	33.30%	1.50E-126	hypothetical protein CDS	hypothetical protein	Substitution	KLI70436.1	K -> Q
Variants: VaAres1_C_FDSW210055062-1r	C	27269	27269	1	A -> C	170	SNP (transversion)	25.30%	4.70E-102	hypothetical protein CDS	hypothetical protein	Substitution	KLI70436.1	K -> Q
Variants: VaphiVaphiSt2_B_A_FDSW210055054-2r	C	27269	27269	1	A -> C	142	SNP (transversion)	26.80%	1.90E-91	hypothetical protein CDS	hypothetical protein	Substitution	KLI70436.1	K -> Q
Variants: VaphiSt2_B_FDSW210055056-1r	A	27267	27267	1	T -> A	288	SNP (transversion)	25.70%	1.40E-167	hypothetical protein CDS	hypothetical protein	Substitution	KLI70436.1	L -> Q
Variants: VaphiSt2_B_FDSW210055056-1r	C	27269	27269	1	A -> C	333	SNP (transversion)	34.50%	6.50E-277	hypothetical protein CDS	hypothetical protein	Substitution	KLI70436.1	K -> Q

Contig 28

Variant	Name	Minimum	Maximum	Length	Change	Coverage	Polymorphism Type	Variant Frequency	Variant P-Value (approximate)	CDS	product	Protein Effect	protein_id	Amino Acid Change
Variants: VaAphrodite_A_FDSW210055057-2r	C	10111	10111	1	T -> C	244	SNP (transition)	27.00%	1.20E-47	calcium-binding protein CDS	calcium-binding protein	None	KLI70416.1	
Variants: VaAphrodite_A_FDSW210055057-2r	C	10129	10129	1	G -> C	311	SNP (transversion)	39.50%	3.90E-146	calcium-binding protein CDS	calcium-binding protein	None	KLI70416.1	
Variants: VaAphrodite_A_FDSW210055057-2r	T	10145	10145	1	C -> T	361	SNP (transition)	35.70%	7.00E-210	calcium-binding protein CDS	calcium-binding protein	None	KLI70416.1	
Variants: VaAphrodite_A_FDSW210055057-2r	C	10153	10153	1	T -> C	375	SNP (transition)	33.60%	1.50E-238	calcium-binding protein CDS	calcium-binding protein	None	KLI70416.1	
Variants: VaAphrodite_A_FDSW210055057-2r	T	10159	10159	1	C -> T	390	SNP (transition)	37.20%	1.70E-296	calcium-binding protein CDS	calcium-binding protein	None	KLI70416.1	
Variants: VaAphrodite_A_FDSW210055057-2r	A	10204	10204	1	C -> A	556	SNP (transversion)	39.00%	0	calcium-binding protein CDS	calcium-binding protein	None	KLI70416.1	
Variants: VaAphrodite_A_FDSW210055057-2r	TG	10255	10256	2	GA -> TG	721	Substitution	35.90%	2.10E-292	calcium-binding protein CDS	calcium-binding protein	Substitution	KLI70416.1	VT -> VA
Variants: VaAphrodite_A_FDSW210055057-2r	C	10273	10273	1	T -> C	722	SNP (transition)	41.60%	0	calcium-binding protein CDS	calcium-binding protein	None	KLI70416.1	
Variants: VaAphrodite_A_FDSW210055057-2r	G	10282	10282	1	C -> G	733	SNP (transversion)	41.60%	0	calcium-binding protein CDS	calcium-binding protein	None	KLI70416.1	
Variants: VaAphrodite_A_FDSW210055057-2r	A	10288	10288	1	G -> A	750	SNP (transition)	37.50%	0	calcium-binding protein CDS	calcium-binding protein	None	KLI70416.1	
Variants: VaAphrodite_A_FDSW210055057-2r	T	10315	10315	1	C -> T	905	SNP (transition)	26.10%	0	calcium-binding protein CDS	calcium-binding protein	None	KLI70416.1	
Variants: VaAphrodite_A_FDSW210055057-2r	T	10322	10322	1	G -> T	927	SNP (transversion)	51.00%	0	calcium-binding protein CDS	calcium-binding protein	Substitution	KLI70416.1	A -> S
Variants: VaAphrodite_A_FDSW210055057-2r	C	10333	10333	1	T -> C	984	SNP (transition)	33.60%	0	calcium-binding protein CDS	calcium-binding protein	None	KLI70416.1	
Variants: VaAphrodite_A_FDSW210055057-2r	T	10351	10351	1	A -> T	1055	SNP (transversion)	31.70%	0	calcium-binding protein CDS	calcium-binding protein	None	KLI70416.1	
Variants: VaAphrodite_A_FDSW210055057-2r	A	10369	10369	1	G -> A	1123	SNP (transition)	34.80%	0	calcium-binding protein CDS	calcium-binding protein	None	KLI70416.1	
Variants: VaAphrodite_A_FDSW210055057-2r	A	10387	10387	1	G -> A	1175	SNP (transition)	46.40%	0	calcium-binding protein CDS	calcium-binding protein	None	KLI70416.1	
Variants: VaAphrodite_A_FDSW210055057-2r	C	10396	10396	1	T -> C	1189	SNP (transition)	33.70%	0	calcium-binding protein CDS	calcium-binding protein	None	KLI70416.1	
Variants: VaAphrodite_A_FDSW210055057-2r	G	10405	10405	1	C -> G	1209	SNP (transversion)	50.20%	0	calcium-binding protein CDS	calcium-binding protein	None	KLI70416.1	
Variants: VaAphrodite_A_FDSW210055057-2r	G	10414	10414	1	A -> G	1215	SNP (transition)	26.30%	0	calcium-binding protein CDS	calcium-binding protein	None	KLI70416.1	
Variants: VaAphrodite_A_FDSW210055057-2r	T	10429	10429	1	C -> T	1229	SNP (transition)	29.70%	0	calcium-binding protein CDS	calcium-binding protein	None	KLI70416.1	

Variants: VaAphrodite_A_FDSW2100550 57-2r	T	10435	10435	1	G -> T	1207	SNP (transversion)	25.70%	0	calcium-binding protein CDS	calcium-binding protein	None	KLI70416.1	
Variants: VaAphrodite_A_FDSW2100550 57-2r	G	10441	10441	1	A -> G	1180	SNP (transition)	47.80%	0	calcium-binding protein CDS	calcium-binding protein	None	KLI70416.1	
Variants: VaAphrodite_A_FDSW2100550 57-2r	C	10444	10444	1	A -> C	1164	SNP (transversion)	42.40%	0	calcium-binding protein CDS	calcium-binding protein	None	KLI70416.1	
Variants: VaAphrodite_A_FDSW2100550 57-2r	ACC	10481	10483	2	TCA -> ACC	1064 -> 1075	Substitution	29.6% -> 29.9%	0	calcium-binding protein CDS	calcium-binding protein	Substitution	KLI70 416.1	S -> T
Variants: VaAphrodite_A_FDSW2100550 57-2r	A	10534	10534	1	G -> A	951	SNP (transition)	91.60%	0	calcium-binding protein CDS	calcium-binding protein	None	KLI70416.1	
Variants: VaAphrodite_A_FDSW2100550 57-2r	C	10540	10540	1	T -> C	944	SNP (transition)	95.40%	0	calcium-binding protein CDS	calcium-binding protein	None	KLI70416.1	
Variants: VaAphrodite_A_FDSW2100550 57-2r	G	10543	10543	1	C -> G	923	SNP (transversion)	30.10%	0	calcium-binding protein CDS	calcium-binding protein	None	KLI70416.1	
Variants: VaAphrodite_A_FDSW2100550 57-2r	T	10546	10546	1	C -> T	922	SNP (transition)	96.40%	0	calcium-binding protein CDS	calcium-binding protein	None	KLI70416.1	
Variants: VaAphrodite_A_FDSW2100550 57-2r	AGCGGA AACG	10549	10558	9	GTCCACTGTT -> AGCGGAAACG	899 -> 916	Substitution	90.7% -> 95.1%	0	calcium-binding protein CDS	calcium-binding protein	Substitution	KLI70 416.1	ESTV -> EAET
Variants: VaAphrodite_A_FDSW2100550 57-2r	A	10564	10564	1	C -> A	889	SNP (transversion)	75.00%	0	calcium-binding protein CDS	calcium-binding protein	None	KLI70416.1	
Variants: VaAphrodite_A_FDSW2100550 57-2r	TTG	10568	10570	2	CTA -> TTG	872 -> 878	Substitution	97.8% -> 97.9%	0	calcium-binding protein CDS	calcium-binding protein	None	KLI70416.1	
Variants: VaAphrodite_A_FDSW2100550 57-2r	T	10573	10573	1	C -> T	857	SNP (transition)	98.70%	0	calcium-binding protein CDS	calcium-binding protein	None	KLI70416.1	
Variants: VaAphrodite_A_FDSW2100550 57-2r	T	10576	10576	1	G -> T	848	SNP (transversion)	98.50%	0	calcium-binding protein CDS	calcium-binding protein	None	KLI70416.1	
Variants: VaAphrodite_A_FDSW2100550 57-2r	TCTC	10579	10582	3	CTTG -> TCTC	817 -> 829	Substitution	98.4% -> 98.5%	0	calcium-binding protein CDS	calcium-binding protein	None	KLI70416.1	
Variants: VaAphrodite_A_FDSW2100550 57-2r	T	10596	10596	1	C -> T	753	SNP (transition)	99.10%	0	calcium-binding protein CDS	calcium-binding protein	Substitution	KLI70 416.1	A -> V
Variants: VaAphrodite_A_FDSW2100550 57-2r	C	10609	10609	1	T -> C	670	SNP (transition)	98.80%	0	calcium-binding protein CDS	calcium-binding protein	None	KLI70416.1	
Variants: VaAphrodite_A_FDSW2100550 57-2r	A	10615	10615	1	G -> A	639	SNP (transition)	98.70%	0	calcium-binding protein CDS	calcium-binding protein	None	KLI70416.1	
Variants: VaAphrodite_B_FDSW2100550 58-2r	C	10129	10129	1	G -> C	237	SNP (transversion)	37.10%	9.60E-111	calcium-binding protein CDS	calcium-binding protein	None	KLI70416.1	
Variants: VaAphrodite_B_FDSW2100550 58-2r	T	10145	10145	1	C -> T	268	SNP (transition)	28.70%	5.90E-125	calcium-binding protein CDS	calcium-binding protein	None	KLI70416.1	
Variants: VaAphrodite_B_FDSW2100550 58-2r	C	10153	10153	1	T -> C	285	SNP (transition)	32.30%	9.70E-173	calcium-binding protein CDS	calcium-binding protein	None	KLI70416.1	
Variants: VaAphrodite_B_FDSW2100550 58-2r	T	10159	10159	1	C -> T	296	SNP (transition)	34.50%	5.60E-205	calcium-binding protein CDS	calcium-binding protein	None	KLI70416.1	
Variants: VaAphrodite_B_FDSW2100550 58-2r	A	10204	10204	1	C -> A	343	SNP (transversion)	38.50%	0	calcium-binding protein CDS	calcium-binding protein	None	KLI70416.1	

Variants: VaAphrodite_B_FDSW210055058-2r	TG	10255	10256	2	GA -> TG	431 -> 432	Substitution	30.2% -> 30.3%	1.90E-112	calcium-binding protein CDS	calcium-binding protein	Substitution	KLI70416.1	VT -> VA
Variants: VaAphrodite_B_FDSW210055058-2r	C	10273	10273	1	T -> C	445	SNP (transition)	41.30%	7.40E-295	calcium-binding protein CDS	calcium-binding protein	None	KLI70416.1	
Variants: VaAphrodite_B_FDSW210055058-2r	G	10282	10282	1	C -> G	446	SNP (transversion)	40.40%	4.40E-286	calcium-binding protein CDS	calcium-binding protein	None	KLI70416.1	
Variants: VaAphrodite_B_FDSW210055058-2r	A	10288	10288	1	G -> A	469	SNP (transition)	36.90%	1.70E-181	calcium-binding protein CDS	calcium-binding protein	None	KLI70416.1	
Variants: VaAphrodite_B_FDSW210055058-2r	T	10315	10315	1	C -> T	592	SNP (transition)	28.90%	5.70E-225	calcium-binding protein CDS	calcium-binding protein	None	KLI70416.1	
Variants: VaAphrodite_B_FDSW210055058-2r	T	10318	10318	1	C -> T	613	SNP (transition)	25.10%	4.40E-162	calcium-binding protein CDS	calcium-binding protein	None	KLI70416.1	
Variants: VaAphrodite_B_FDSW210055058-2r	T	10322	10322	1	G -> T	639	SNP (transversion)	53.40%	0	calcium-binding protein CDS	calcium-binding protein	Substitution	KLI70416.1	A -> S
Variants: VaAphrodite_B_FDSW210055058-2r	C	10333	10333	1	T -> C	702	SNP (transition)	39.50%	0	calcium-binding protein CDS	calcium-binding protein	None	KLI70416.1	
Variants: VaAphrodite_B_FDSW210055058-2r	T	10351	10351	1	A -> T	769	SNP (transversion)	29.10%	0	calcium-binding protein CDS	calcium-binding protein	None	KLI70416.1	
Variants: VaAphrodite_B_FDSW210055058-2r	A	10369	10369	1	G -> A	821	SNP (transition)	33.40%	0	calcium-binding protein CDS	calcium-binding protein	None	KLI70416.1	
Variants: VaAphrodite_B_FDSW210055058-2r	A	10387	10387	1	G -> A	857	SNP (transition)	45.70%	0	calcium-binding protein CDS	calcium-binding protein	None	KLI70416.1	
Variants: VaAphrodite_B_FDSW210055058-2r	C	10396	10396	1	T -> C	869	SNP (transition)	28.90%	0	calcium-binding protein CDS	calcium-binding protein	None	KLI70416.1	
Variants: VaAphrodite_B_FDSW210055058-2r	G	10405	10405	1	C -> G	884	SNP (transversion)	45.10%	0	calcium-binding protein CDS	calcium-binding protein	None	KLI70416.1	
Variants: VaAphrodite_B_FDSW210055058-2r	T	10429	10429	1	C -> T	901	SNP (transition)	25.30%	0	calcium-binding protein CDS	calcium-binding protein	None	KLI70416.1	
Variants: VaAphrodite_B_FDSW210055058-2r	GT	10434	10435	2	CG -> GT	910 -> 913	Substitution	25.6% -> 25.7%	5.70E-295	calcium-binding protein CDS	calcium-binding protein	Substitution	KLI70416.1	T -> S
Variants: VaAphrodite_B_FDSW210055058-2r	G	10441	10441	1	A -> G	913	SNP (transition)	40.40%	0	calcium-binding protein CDS	calcium-binding protein	None	KLI70416.1	
Variants: VaAphrodite_B_FDSW210055058-2r	C	10444	10444	1	A -> C	910	SNP (transversion)	45.90%	0	calcium-binding protein CDS	calcium-binding protein	None	KLI70416.1	
Variants: VaAphrodite_B_FDSW210055058-2r	G	10468	10468	1	A -> G	920	SNP (transition)	28.30%	0	calcium-binding protein CDS	calcium-binding protein	None	KLI70416.1	
Variants: VaAphrodite_B_FDSW210055058-2r	AT	10470	10471	2	GC -> AT	915 -> 920	Substitution	27.7% -> 27.8%	0	calcium-binding protein CDS	calcium-binding protein	Substitution	KLI70416.1	S -> N
Variants: VaAphrodite_B_FDSW210055058-2r	A	10481	10481	1	T -> A	922	SNP (transversion)	47.30%	0	calcium-binding protein CDS	calcium-binding protein	Substitution	KLI70416.1	S -> T
Variants: VaAphrodite_B_FDSW210055058-2r	C	10486	10486	1	T -> C	916	SNP (transition)	34.50%	0	calcium-binding protein CDS	calcium-binding protein	None	KLI70416.1	
Variants: VaAphrodite_B_FDSW210055058-2r	C	10498	10498	1	T -> C	932	SNP (transition)	41.70%	0	calcium-binding protein CDS	calcium-binding protein	None	KLI70416.1	

Variants: VaAphrodite_B_FDSW210055058-2r	A	10534	10534	1	G -> A	975	SNP (transition)	93.70%	0	calcium-binding protein CDS	calcium-binding protein	None	KLI70416.1	
Variants: VaAphrodite_B_FDSW210055058-2r	C	10540	10540	1	T -> C	972	SNP (transition)	97.10%	0	calcium-binding protein CDS	calcium-binding protein	None	KLI70416.1	
Variants: VaAphrodite_B_FDSW210055058-2r	G	10543	10543	1	C -> G	961	SNP (transversion)	48.80%	0	calcium-binding protein CDS	calcium-binding protein	None	KLI70416.1	
Variants: VaAphrodite_B_FDSW210055058-2r	T	10546	10546	1	C -> T	963	SNP (transition)	97.20%	0	calcium-binding protein CDS	calcium-binding protein	None	KLI70416.1	
Variants: VaAphrodite_B_FDSW210055058-2r	AGCGGAAACG	10549	10558	9	GTCCACTGTT -> AGCGGAAACG	952 -> 963	Substitution	94.2% -> 96.3%	0	calcium-binding protein CDS	calcium-binding protein	Substitution	KLI70416.1	ESTV -> EAET
Variants: VaAphrodite_B_FDSW210055058-2r	A	10564	10564	1	C -> A	944	SNP (transversion)	57.70%	0	calcium-binding protein CDS	calcium-binding protein	None	KLI70416.1	
Variants: VaAphrodite_B_FDSW210055058-2r	G	10564	10564	1	C -> G	944	SNP (transversion)	39.90%	0	calcium-binding protein CDS	calcium-binding protein	None	KLI70416.1	
Variants: VaAphrodite_B_FDSW210055058-2r	A	10567	10567	1	G -> A	952	SNP (transition)	39.20%	0	calcium-binding protein CDS	calcium-binding protein	None	KLI70416.1	
Variants: VaAphrodite_B_FDSW210055058-2r	TTG	10568	10570	2	CTA -> TTG	936 -> 950	Substitution	96.80%	0	calcium-binding protein CDS	calcium-binding protein	None	KLI70416.1	
Variants: VaAphrodite_B_FDSW210055058-2r	T	10573	10573	1	C -> T	933	SNP (transition)	98.40%	0	calcium-binding protein CDS	calcium-binding protein	None	KLI70416.1	
Variants: VaAphrodite_B_FDSW210055058-2r	T	10576	10576	1	G -> T	925	SNP (transversion)	98.40%	0	calcium-binding protein CDS	calcium-binding protein	None	KLI70416.1	
Variants: VaAphrodite_B_FDSW210055058-2r	TCTC	10579	10582	3	CTTG -> TCTC	922 -> 925	Substitution	69.8% -> 70.6%	0	calcium-binding protein CDS	calcium-binding protein	None	KLI70416.1	
Variants: VaAphrodite_B_FDSW210055058-2r	TCTT	10579	10582	3	CTTG -> TCTT	922 -> 925	Substitution	25.8% -> 25.9%	0	calcium-binding protein CDS	calcium-binding protein	None	KLI70416.1	
Variants: VaAphrodite_B_FDSW210055058-2r	T	10596	10596	1	C -> T	890	SNP (transition)	97.30%	0	calcium-binding protein CDS	calcium-binding protein	Substitution	KLI70416.1	A -> V
Variants: VaAphrodite_B_FDSW210055058-2r	C	10609	10609	1	T -> C	812	SNP (transition)	95.90%	0	calcium-binding protein CDS	calcium-binding protein	None	KLI70416.1	
Variants: VaAphrodite_B_FDSW210055058-2r	A	10615	10615	1	G -> A	774	SNP (transition)	96.10%	0	calcium-binding protein CDS	calcium-binding protein	None	KLI70416.1	
Variants: VaAphrodite_C_FDSW210055059-1r	C	10129	10129	1	G -> C	307	SNP (transversion)	43.30%	9.70E-191	calcium-binding protein CDS	calcium-binding protein	None	KLI70416.1	
Variants: VaAphrodite_C_FDSW210055059-1r	T	10145	10145	1	C -> T	365	SNP (transition)	37.50%	5.20E-240	calcium-binding protein CDS	calcium-binding protein	None	KLI70416.1	
Variants: VaAphrodite_C_FDSW210055059-1r	C	10153	10153	1	T -> C	394	SNP (transition)	38.30%	3.5E-311	calcium-binding protein CDS	calcium-binding protein	None	KLI70416.1	
Variants: VaAphrodite_C_FDSW210055059-1r	T	10159	10159	1	C -> T	411	SNP (transition)	41.10%	0	calcium-binding protein CDS	calcium-binding protein	None	KLI70416.1	
Variants: VaAphrodite_C_FDSW210055059-1r	A	10204	10204	1	C -> A	505	SNP (transversion)	44.00%	0	calcium-binding protein CDS	calcium-binding protein	None	KLI70416.1	
Variants: VaAphrodite_C_FDSW210055059-1r	TG	10255	10256	2	GA -> TG	633 -> 639	Substitution	26.2% -> 26.8%	1.70E-131	calcium-binding protein CDS	calcium-binding protein	Substitution	KLI70416.1	VT -> VA

Variants: VaAphrodite_C_FDSW210055059-1r	C	10273	10273	1	T -> C	654	SNP (transition)	36.90%	0	calcium-binding protein CDS	calcium-binding protein	None	KLI70416.1	
Variants: VaAphrodite_C_FDSW210055059-1r	G	10282	10282	1	C -> G	671	SNP (transversion)	36.80%	0	calcium-binding protein CDS	calcium-binding protein	None	KLI70416.1	
Variants: VaAphrodite_C_FDSW210055059-1r	A	10288	10288	1	G -> A	694	SNP (transition)	32.60%	4.70E-266	calcium-binding protein CDS	calcium-binding protein	None	KLI70416.1	
Variants: VaAphrodite_C_FDSW210055059-1r	T	10315	10315	1	C -> T	900	SNP (transition)	27.80%	0	calcium-binding protein CDS	calcium-binding protein	None	KLI70416.1	
Variants: VaAphrodite_C_FDSW210055059-1r	T	10322	10322	1	G -> T	957	SNP (transversion)	48.20%	0	calcium-binding protein CDS	calcium-binding protein	Substitution	KLI70416.1	A -> S
Variants: VaAphrodite_C_FDSW210055059-1r	C	10333	10333	1	T -> C	1025	SNP (transition)	32.90%	0	calcium-binding protein CDS	calcium-binding protein	None	KLI70416.1	
Variants: VaAphrodite_C_FDSW210055059-1r	T	10351	10351	1	A -> T	1134	SNP (transversion)	35.40%	0	calcium-binding protein CDS	calcium-binding protein	None	KLI70416.1	
Variants: VaAphrodite_C_FDSW210055059-1r	A	10369	10369	1	G -> A	1218	SNP (transition)	34.70%	0	calcium-binding protein CDS	calcium-binding protein	None	KLI70416.1	
Variants: VaAphrodite_C_FDSW210055059-1r	A	10387	10387	1	G -> A	1258	SNP (transition)	45.90%	0	calcium-binding protein CDS	calcium-binding protein	None	KLI70416.1	
Variants: VaAphrodite_C_FDSW210055059-1r	C	10396	10396	1	T -> C	1291	SNP (transition)	35.20%	0	calcium-binding protein CDS	calcium-binding protein	None	KLI70416.1	
Variants: VaAphrodite_C_FDSW210055059-1r	G	10405	10405	1	C -> G	1322	SNP (transversion)	52.60%	0	calcium-binding protein CDS	calcium-binding protein	None	KLI70416.1	
Variants: VaAphrodite_C_FDSW210055059-1r	G	10414	10414	1	A -> G	1338	SNP (transition)	26.00%	0	calcium-binding protein CDS	calcium-binding protein	None	KLI70416.1	
Variants: VaAphrodite_C_FDSW210055059-1r	T	10429	10429	1	C -> T	1364	SNP (transition)	28.40%	0	calcium-binding protein CDS	calcium-binding protein	None	KLI70416.1	
Variants: VaAphrodite_C_FDSW210055059-1r	T	10435	10435	1	G -> T	1358	SNP (transversion)	26.10%	0	calcium-binding protein CDS	calcium-binding protein	None	KLI70416.1	
Variants: VaAphrodite_C_FDSW210055059-1r	G	10441	10441	1	A -> G	1320	SNP (transition)	43.90%	0	calcium-binding protein CDS	calcium-binding protein	None	KLI70416.1	
Variants: VaAphrodite_C_FDSW210055059-1r	C	10444	10444	1	A -> C	1326	SNP (transversion)	46.50%	0	calcium-binding protein CDS	calcium-binding protein	None	KLI70416.1	
Variants: VaAphrodite_C_FDSW210055059-1r	G	10468	10468	1	A -> G	1297	SNP (transition)	26.90%	0	calcium-binding protein CDS	calcium-binding protein	None	KLI70416.1	
Variants: VaAphrodite_C_FDSW210055059-1r	AT	10470	10471	2	GC -> AT	1302 -> 1304	Substitution	26.5% -> 26.6%	0	calcium-binding protein CDS	calcium-binding protein	Substitution	KLI70416.1	S -> N
Variants: VaAphrodite_C_FDSW210055059-1r	A	10481	10481	1	T -> A	1290	SNP (transversion)	44.30%	0	calcium-binding protein CDS	calcium-binding protein	Substitution	KLI70416.1	S -> T
Variants: VaAphrodite_C_FDSW210055059-1r	C	10486	10486	1	T -> C	1306	SNP (transition)	32.70%	0	calcium-binding protein CDS	calcium-binding protein	None	KLI70416.1	
Variants: VaAphrodite_C_FDSW210055059-1r	C	10498	10498	1	T -> C	1322	SNP (transition)	42.20%	0	calcium-binding protein CDS	calcium-binding protein	None	KLI70416.1	
Variants: VaAphrodite_C_FDSW210055059-1r	A	10534	10534	1	G -> A	1362	SNP (transition)	93.10%	0	calcium-binding protein CDS	calcium-binding protein	None	KLI70416.1	

Variants: VaAphrodite_C_FDSW210055059-1r	C	10540	10540	1	T -> C	1347	SNP (transition)	95.80%	0	calcium-binding protein CDS	calcium-binding protein	None	KLI70416.1	
Variants: VaAphrodite_C_FDSW210055059-1r	G	10543	10543	1	C -> G	1343	SNP (transversion)	50.10%	0	calcium-binding protein CDS	calcium-binding protein	None	KLI70416.1	
Variants: VaAphrodite_C_FDSW210055059-1r	T	10546	10546	1	C -> T	1330	SNP (transition)	96.20%	0	calcium-binding protein CDS	calcium-binding protein	None	KLI70416.1	
Variants: VaAphrodite_C_FDSW210055059-1r	AGCGGAAACG	10549	10558	9	GTCCACTGTT -> AGCGGAAACG	1307 -> 1325	Substitution	93.8% -> 96.0%	0	calcium-binding protein CDS	calcium-binding protein	Substitution	KLI70416.1	ESTV -> EAET
Variants: VaAphrodite_C_FDSW210055059-1r	A	10564	10564	1	C -> A	1302	SNP (transversion)	55.90%	0	calcium-binding protein CDS	calcium-binding protein	None	KLI70416.1	
Variants: VaAphrodite_C_FDSW210055059-1r	G	10564	10564	1	C -> G	1302	SNP (transversion)	41.90%	0	calcium-binding protein CDS	calcium-binding protein	None	KLI70416.1	
Variants: VaAphrodite_C_FDSW210055059-1r	A	10567	10567	1	G -> A	1294	SNP (transition)	40.80%	0	calcium-binding protein CDS	calcium-binding protein	None	KLI70416.1	
Variants: VaAphrodite_C_FDSW210055059-1r	TTG	10568	10570	2	CTA -> TTG	1280 -> 1289	Substitution	97.7% -> 97.8%	0	calcium-binding protein CDS	calcium-binding protein	None	KLI70416.1	
Variants: VaAphrodite_C_FDSW210055059-1r	T	10573	10573	1	C -> T	1284	SNP (transition)	97.50%	0	calcium-binding protein CDS	calcium-binding protein	None	KLI70416.1	
Variants: VaAphrodite_C_FDSW210055059-1r	T	10576	10576	1	G -> T	1288	SNP (transversion)	98.10%	0	calcium-binding protein CDS	calcium-binding protein	None	KLI70416.1	
Variants: VaAphrodite_C_FDSW210055059-1r	TC	10579	10580	2	CT -> TC	1276 -> 1281	Substitution	25.4% -> 26.1%	0	calcium-binding protein CDS	calcium-binding protein	None	KLI70416.1	
Variants: VaAphrodite_C_FDSW210055059-1r	TCTC	10579	10582	3	CTTG -> TCTC	1276 -> 1285	Substitution	71.6% -> 72.3%	0	calcium-binding protein CDS	calcium-binding protein	None	KLI70416.1	
Variants: VaAphrodite_C_FDSW210055059-1r	T	10596	10596	1	C -> T	1222	SNP (transition)	97.50%	0	calcium-binding protein CDS	calcium-binding protein	Substitution	KLI70416.1	A -> V
Variants: VaAphrodite_C_FDSW210055059-1r	C	10609	10609	1	T -> C	1077	SNP (transition)	98.10%	0	calcium-binding protein CDS	calcium-binding protein	None	KLI70416.1	
Variants: VaAphrodite_C_FDSW210055059-1r	A	10615	10615	1	G -> A	1022	SNP (transition)	97.90%	0	calcium-binding protein CDS	calcium-binding protein	None	KLI70416.1	
Variants: VaAphrodite_C_FDSW210055059-1r	AAG	10630	10632	3	CTC -> AAG	869 -> 888	Substitution	27.3% -> 27.7%	0	calcium-binding protein CDS	calcium-binding protein	None	KLI70416.1	
Variants: VaAres1_A_FDSW210055060-2r	C	10129	10129	1	G -> C	169	SNP (transversion)	42.00%	2.00E-94	calcium-binding protein CDS	calcium-binding protein	None	KLI70416.1	
Variants: VaAres1_A_FDSW210055060-2r	T	10145	10145	1	C -> T	193	SNP (transition)	34.70%	1.70E-115	calcium-binding protein CDS	calcium-binding protein	None	KLI70416.1	
Variants: VaAres1_A_FDSW210055060-2r	C	10153	10153	1	T -> C	212	SNP (transition)	40.10%	1.50E-186	calcium-binding protein CDS	calcium-binding protein	None	KLI70416.1	
Variants: VaAres1_A_FDSW210055060-2r	T	10159	10159	1	C -> T	223	SNP (transition)	43.50%	4.70E-217	calcium-binding protein CDS	calcium-binding protein	None	KLI70416.1	
Variants: VaAres1_A_FDSW210055060-2r	A	10204	10204	1	C -> A	298	SNP (transversion)	46.00%	0	calcium-binding protein CDS	calcium-binding protein	None	KLI70416.1	
Variants: VaAres1_A_FDSW210055060-2r	TG	10255	10256	2	GA -> TG	418 -> 420	Substitution	29.9% -> 30.0%	2.10E-106	calcium-binding protein CDS	calcium-binding protein	Substitution	KLI70416.1	VT -> VA

Variants: VaAres1_A_FDSW210055060-2r	C	10273	10273	1	T -> C	425	SNP (transition)	36.70%	3.60E-240	calcium-binding protein CDS	calcium-binding protein	None	KLI70416.1	
Variants: VaAres1_A_FDSW210055060-2r	G	10282	10282	1	C -> G	429	SNP (transversion)	35.90%	2.70E-235	calcium-binding protein CDS	calcium-binding protein	None	KLI70416.1	
Variants: VaAres1_A_FDSW210055060-2r	A	10288	10288	1	G -> A	450	SNP (transition)	33.60%	1.00E-180	calcium-binding protein CDS	calcium-binding protein	None	KLI70416.1	
Variants: VaAres1_A_FDSW210055060-2r	T	10315	10315	1	C -> T	555	SNP (transition)	28.30%	1.30E-251	calcium-binding protein CDS	calcium-binding protein	None	KLI70416.1	
Variants: VaAres1_A_FDSW210055060-2r	T	10322	10322	1	G -> T	583	SNP (transversion)	47.00%	0	calcium-binding protein CDS	calcium-binding protein	Substitution	KLI70416.1	A -> S
Variants: VaAres1_A_FDSW210055060-2r	C	10333	10333	1	T -> C	613	SNP (transition)	30.80%	4.40E-236	calcium-binding protein CDS	calcium-binding protein	None	KLI70416.1	
Variants: VaAres1_A_FDSW210055060-2r	T	10351	10351	1	A -> T	681	SNP (transversion)	31.60%	0	calcium-binding protein CDS	calcium-binding protein	None	KLI70416.1	
Variants: VaAres1_A_FDSW210055060-2r	A	10369	10369	1	G -> A	693	SNP (transition)	36.20%	0	calcium-binding protein CDS	calcium-binding protein	None	KLI70416.1	
Variants: VaAres1_A_FDSW210055060-2r	A	10387	10387	1	G -> A	722	SNP (transition)	41.60%	0	calcium-binding protein CDS	calcium-binding protein	None	KLI70416.1	
Variants: VaAres1_A_FDSW210055060-2r	C	10396	10396	1	T -> C	741	SNP (transition)	33.20%	0	calcium-binding protein CDS	calcium-binding protein	None	KLI70416.1	
Variants: VaAres1_A_FDSW210055060-2r	G	10405	10405	1	C -> G	745	SNP (transversion)	50.60%	0	calcium-binding protein CDS	calcium-binding protein	None	KLI70416.1	
Variants: VaAres1_A_FDSW210055060-2r	G	10414	10414	1	A -> G	751	SNP (transition)	26.00%	1.10E-303	calcium-binding protein CDS	calcium-binding protein	None	KLI70416.1	
Variants: VaAres1_A_FDSW210055060-2r	T	10429	10429	1	C -> T	758	SNP (transition)	29.60%	0	calcium-binding protein CDS	calcium-binding protein	None	KLI70416.1	
Variants: VaAres1_A_FDSW210055060-2r	G	10441	10441	1	A -> G	738	SNP (transition)	46.30%	0	calcium-binding protein CDS	calcium-binding protein	None	KLI70416.1	
Variants: VaAres1_A_FDSW210055060-2r	C	10444	10444	1	A -> C	730	SNP (transversion)	39.90%	0	calcium-binding protein CDS	calcium-binding protein	None	KLI70416.1	
Variants: VaAres1_A_FDSW210055060-2r	ACC	10481	10483	2	TCA -> ACC	673 -> 675	Substitution	27.6% -> 27.9%	0	calcium-binding protein CDS	calcium-binding protein	Substitution	KLI70416.1	S -> T
Variants: VaAres1_A_FDSW210055060-2r	A	10534	10534	1	G -> A	603	SNP (transition)	90.50%	0	calcium-binding protein CDS	calcium-binding protein	None	KLI70416.1	
Variants: VaAres1_A_FDSW210055060-2r	C	10540	10540	1	T -> C	601	SNP (transition)	93.70%	0	calcium-binding protein CDS	calcium-binding protein	None	KLI70416.1	
Variants: VaAres1_A_FDSW210055060-2r	G	10543	10543	1	C -> G	596	SNP (transversion)	29.90%	0	calcium-binding protein CDS	calcium-binding protein	None	KLI70416.1	
Variants: VaAres1_A_FDSW210055060-2r	T	10546	10546	1	C -> T	598	SNP (transition)	94.10%	0	calcium-binding protein CDS	calcium-binding protein	None	KLI70416.1	
Variants: VaAres1_A_FDSW210055060-2r	AGCGGAAACG	10549	10558	9	GTCCACTGTT -> AGCGGAAACG	588 -> 599	Substitution	94.0% -> 94.8%	0	calcium-binding protein CDS	calcium-binding protein	Substitution	KLI70416.1	ESTV -> EAET
Variants: VaAres1_A_FDSW210055060-2r	A	10564	10564	1	C -> A	589	SNP (transversion)	73.70%	0	calcium-binding protein CDS	calcium-binding protein	None	KLI70416.1	

Variants: VaAres1_A_FDSW210055060-2r	TTG	10568	10570	2	CTA -> TTG	581 -> 585	Substitution	95.5% -> 95.6%	0	calcium-binding protein CDS	calcium-binding protein	None	KLI70416.1	
Variants: VaAres1_A_FDSW210055060-2r	T	10573	10573	1	C -> T	580	SNP (transition)	96.20%	0	calcium-binding protein CDS	calcium-binding protein	None	KLI70416.1	
Variants: VaAres1_A_FDSW210055060-2r	T	10576	10576	1	G -> T	578	SNP (transversion)	95.70%	0	calcium-binding protein CDS	calcium-binding protein	None	KLI70416.1	
Variants: VaAres1_A_FDSW210055060-2r	TCTC	10579	10582	3	CTTG -> TCTC	574 -> 579	Substitution	95.0% -> 95.5%	0	calcium-binding protein CDS	calcium-binding protein	None	KLI70416.1	
Variants: VaAres1_A_FDSW210055060-2r	T	10596	10596	1	C -> T	521	SNP (transition)	97.30%	0	calcium-binding protein CDS	calcium-binding protein	Substitution	KLI70416.1	A -> V
Variants: VaAres1_A_FDSW210055060-2r	C	10609	10609	1	T -> C	476	SNP (transition)	97.30%	0	calcium-binding protein CDS	calcium-binding protein	None	KLI70416.1	
Variants: VaAres1_A_FDSW210055060-2r	A	10615	10615	1	G -> A	452	SNP (transition)	97.60%	0	calcium-binding protein CDS	calcium-binding protein	None	KLI70416.1	
Variants: VaAres1_B_FDSW210055061-2r	C	10129	10129	1	G -> C	207	SNP (transversion)	40.60%	1.60E-101	calcium-binding protein CDS	calcium-binding protein	None	KLI70416.1	
Variants: VaAres1_B_FDSW210055061-2r	T	10145	10145	1	C -> T	244	SNP (transition)	29.90%	4.70E-120	calcium-binding protein CDS	calcium-binding protein	None	KLI70416.1	
Variants: VaAres1_B_FDSW210055061-2r	C	10153	10153	1	T -> C	258	SNP (transition)	31.40%	3.90E-151	calcium-binding protein CDS	calcium-binding protein	None	KLI70416.1	
Variants: VaAres1_B_FDSW210055061-2r	T	10159	10159	1	C -> T	269	SNP (transition)	34.20%	7.20E-194	calcium-binding protein CDS	calcium-binding protein	None	KLI70416.1	
Variants: VaAres1_B_FDSW210055061-2r	A	10204	10204	1	C -> A	363	SNP (transversion)	40.80%	0	calcium-binding protein CDS	calcium-binding protein	None	KLI70416.1	
Variants: VaAres1_B_FDSW210055061-2r	C	10273	10273	1	T -> C	493	SNP (transition)	33.70%	2.50E-297	calcium-binding protein CDS	calcium-binding protein	None	KLI70416.1	
Variants: VaAres1_B_FDSW210055061-2r	G	10282	10282	1	C -> G	507	SNP (transversion)	32.90%	1.40E-280	calcium-binding protein CDS	calcium-binding protein	None	KLI70416.1	
Variants: VaAres1_B_FDSW210055061-2r	A	10288	10288	1	G -> A	528	SNP (transition)	26.10%	1.00E-134	calcium-binding protein CDS	calcium-binding protein	None	KLI70416.1	
Variants: VaAres1_B_FDSW210055061-2r	T	10322	10322	1	G -> T	698	SNP (transversion)	41.50%	0	calcium-binding protein CDS	calcium-binding protein	Substitution	KLI70416.1	A -> S
Variants: VaAres1_B_FDSW210055061-2r	C	10333	10333	1	T -> C	731	SNP (transition)	25.70%	8.60E-255	calcium-binding protein CDS	calcium-binding protein	None	KLI70416.1	
Variants: VaAres1_B_FDSW210055061-2r	T	10351	10351	1	A -> T	808	SNP (transversion)	38.00%	0	calcium-binding protein CDS	calcium-binding protein	None	KLI70416.1	
Variants: VaAres1_B_FDSW210055061-2r	T	10360	10360	1	C -> T	841	SNP (transition)	27.20%	0	calcium-binding protein CDS	calcium-binding protein	None	KLI70416.1	
Variants: VaAres1_B_FDSW210055061-2r	A	10369	10369	1	G -> A	865	SNP (transition)	38.00%	0	calcium-binding protein CDS	calcium-binding protein	None	KLI70416.1	
Variants: VaAres1_B_FDSW210055061-2r	A	10387	10387	1	G -> A	910	SNP (transition)	44.70%	0	calcium-binding protein CDS	calcium-binding protein	None	KLI70416.1	
Variants: VaAres1_B_FDSW210055061-2r	C	10396	10396	1	T -> C	927	SNP (transition)	33.80%	0	calcium-binding protein CDS	calcium-binding protein	None	KLI70416.1	

Variants: VaAres1_B_FDSW210055061-2r	G	10405	10405	1	C -> G	989	SNP (transversion)	51.40%	0	calcium-binding protein CDS	calcium-binding protein	None	KLI70416.1	
Variants: VaAres1_B_FDSW210055061-2r	T	10429	10429	1	C -> T	1007	SNP (transition)	31.30%	0	calcium-binding protein CDS	calcium-binding protein	None	KLI70416.1	
Variants: VaAres1_B_FDSW210055061-2r	G	10441	10441	1	A -> G	975	SNP (transition)	47.10%	0	calcium-binding protein CDS	calcium-binding protein	None	KLI70416.1	
Variants: VaAres1_B_FDSW210055061-2r	C	10444	10444	1	A -> C	956	SNP (transversion)	43.10%	0	calcium-binding protein CDS	calcium-binding protein	None	KLI70416.1	
Variants: VaAres1_B_FDSW210055061-2r	ACC	10481	10483	2	TCA -> ACC	916 -> 919	Substitution	29.3% -> 29.4%	0	calcium-binding protein CDS	calcium-binding protein	Substitution	KLI70 416.1	S -> T
Variants: VaAres1_B_FDSW210055061-2r	C	10498	10498	1	T -> C	884	SNP (transition)	29.10%	0	calcium-binding protein CDS	calcium-binding protein	None	KLI70416.1	
Variants: VaAres1_B_FDSW210055061-2r	A	10534	10534	1	G -> A	823	SNP (transition)	89.80%	0	calcium-binding protein CDS	calcium-binding protein	None	KLI70416.1	
Variants: VaAres1_B_FDSW210055061-2r	C	10540	10540	1	T -> C	827	SNP (transition)	94.90%	0	calcium-binding protein CDS	calcium-binding protein	None	KLI70416.1	
Variants: VaAres1_B_FDSW210055061-2r	G	10543	10543	1	C -> G	817	SNP (transversion)	35.10%	0	calcium-binding protein CDS	calcium-binding protein	None	KLI70416.1	
Variants: VaAres1_B_FDSW210055061-2r	T	10546	10546	1	C -> T	799	SNP (transition)	94.90%	0	calcium-binding protein CDS	calcium-binding protein	None	KLI70416.1	
Variants: VaAres1_B_FDSW210055061-2r	AGCGGAAACG	10549	10558	9	GTCCACTGTT -> AGCGGAAACG	740 -> 756	Substitution	90.7% -> 93.5%	0	calcium-binding protein CDS	calcium-binding protein	Substitution	KLI70 416.1	ESTV -> EAET
Variants: VaAres1_B_FDSW210055061-2r	A	10564	10564	1	C -> A	744	SNP (transversion)	72.60%	0	calcium-binding protein CDS	calcium-binding protein	None	KLI70416.1	
Variants: VaAres1_B_FDSW210055061-2r	TTG	10568	10570	2	CTA -> TTG	741 -> 742	Substitution	96.4% -> 96.5%	0	calcium-binding protein CDS	calcium-binding protein	None	KLI70416.1	
Variants: VaAres1_B_FDSW210055061-2r	T	10573	10573	1	C -> T	743	SNP (transition)	97.30%	0	calcium-binding protein CDS	calcium-binding protein	None	KLI70416.1	
Variants: VaAres1_B_FDSW210055061-2r	T	10576	10576	1	G -> T	733	SNP (transversion)	97.50%	0	calcium-binding protein CDS	calcium-binding protein	None	KLI70416.1	
Variants: VaAres1_B_FDSW210055061-2r	TCTC	10579	10582	3	CTTG -> TCTC	734 -> 740	Substitution	96.2% -> 96.6%	0	calcium-binding protein CDS	calcium-binding protein	None	KLI70416.1	
Variants: VaAres1_B_FDSW210055061-2r	T	10596	10596	1	C -> T	737	SNP (transition)	97.80%	0	calcium-binding protein CDS	calcium-binding protein	Substitution	KLI70 416.1	A -> V
Variants: VaAres1_B_FDSW210055061-2r	C	10609	10609	1	T -> C	710	SNP (transition)	98.30%	0	calcium-binding protein CDS	calcium-binding protein	None	KLI70416.1	
Variants: VaAres1_B_FDSW210055061-2r	A	10615	10615	1	G -> A	676	SNP (transition)	98.50%	0	calcium-binding protein CDS	calcium-binding protein	None	KLI70416.1	
Variants: VaAres1_C_FDSW210055062-1r	CATC	10108	10111	3	GGTT -> CATC	235 -> 245	Substitution	25.5% -> 26.7%	1.00E-48	calcium-binding protein CDS	calcium-binding protein	Substitution	KLI70 416.1	TV -> TI
Variants: VaAres1_C_FDSW210055062-1r	C	10129	10129	1	G -> C	322	SNP (transversion)	43.80%	6.40E-217	calcium-binding protein CDS	calcium-binding protein	None	KLI70416.1	
Variants: VaAres1_C_FDSW210055062-1r	T	10145	10145	1	C -> T	372	SNP (transition)	37.10%	2.40E-268	calcium-binding protein CDS	calcium-binding protein	None	KLI70416.1	

Variants: VaAres1_C_FDSW210055062-1r	C	10153	10153	1	T -> C	387	SNP (transition)	38.80%	0	calcium-binding protein CDS	calcium-binding protein	None	KLI70416.1	
Variants: VaAres1_C_FDSW210055062-1r	T	10159	10159	1	C -> T	397	SNP (transition)	40.30%	0	calcium-binding protein CDS	calcium-binding protein	None	KLI70416.1	
Variants: VaAres1_C_FDSW210055062-1r	A	10204	10204	1	C -> A	543	SNP (transversion)	38.50%	0	calcium-binding protein CDS	calcium-binding protein	None	KLI70416.1	
Variants: VaAres1_C_FDSW210055062-1r	TG	10255	10256	2	GA -> TG	737 -> 742	Substitution	31.10%	1.40E-155	calcium-binding protein CDS	calcium-binding protein	Substitution	KLI70416.1	VT -> VA
Variants: VaAres1_C_FDSW210055062-1r	C	10273	10273	1	T -> C	757	SNP (transition)	40.30%	0	calcium-binding protein CDS	calcium-binding protein	None	KLI70416.1	
Variants: VaAres1_C_FDSW210055062-1r	G	10282	10282	1	C -> G	788	SNP (transversion)	41.80%	0	calcium-binding protein CDS	calcium-binding protein	None	KLI70416.1	
Variants: VaAres1_C_FDSW210055062-1r	A	10288	10288	1	G -> A	814	SNP (transition)	35.50%	4.9E-324	calcium-binding protein CDS	calcium-binding protein	None	KLI70416.1	
Variants: VaAres1_C_FDSW210055062-1r	T	10322	10322	1	G -> T	1036	SNP (transversion)	47.60%	0	calcium-binding protein CDS	calcium-binding protein	Substitution	KLI70416.1	A -> S
Variants: VaAres1_C_FDSW210055062-1r	C	10333	10333	1	T -> C	1108	SNP (transition)	34.80%	0	calcium-binding protein CDS	calcium-binding protein	None	KLI70416.1	
Variants: VaAres1_C_FDSW210055062-1r	T	10351	10351	1	A -> T	1206	SNP (transversion)	32.30%	0	calcium-binding protein CDS	calcium-binding protein	None	KLI70416.1	
Variants: VaAres1_C_FDSW210055062-1r	A	10369	10369	1	G -> A	1233	SNP (transition)	32.80%	0	calcium-binding protein CDS	calcium-binding protein	None	KLI70416.1	
Variants: VaAres1_C_FDSW210055062-1r	A	10387	10387	1	G -> A	1227	SNP (transition)	46.40%	0	calcium-binding protein CDS	calcium-binding protein	None	KLI70416.1	
Variants: VaAres1_C_FDSW210055062-1r	C	10396	10396	1	T -> C	1256	SNP (transition)	33.90%	0	calcium-binding protein CDS	calcium-binding protein	None	KLI70416.1	
Variants: VaAres1_C_FDSW210055062-1r	G	10405	10405	1	C -> G	1269	SNP (transversion)	50.00%	0	calcium-binding protein CDS	calcium-binding protein	None	KLI70416.1	
Variants: VaAres1_C_FDSW210055062-1r	G	10414	10414	1	A -> G	1275	SNP (transition)	25.60%	0	calcium-binding protein CDS	calcium-binding protein	None	KLI70416.1	
Variants: VaAres1_C_FDSW210055062-1r	T	10429	10429	1	C -> T	1277	SNP (transition)	28.80%	0	calcium-binding protein CDS	calcium-binding protein	None	KLI70416.1	
Variants: VaAres1_C_FDSW210055062-1r	GT	10434	10435	2	CG -> GT	1292 -> 1293	Substitution	25.1% -> 25.6%	0	calcium-binding protein CDS	calcium-binding protein	Substitution	KLI70416.1	T -> S
Variants: VaAres1_C_FDSW210055062-1r	G	10441	10441	1	A -> G	1268	SNP (transition)	45.00%	0	calcium-binding protein CDS	calcium-binding protein	None	KLI70416.1	
Variants: VaAres1_C_FDSW210055062-1r	C	10444	10444	1	A -> C	1287	SNP (transversion)	47.10%	0	calcium-binding protein CDS	calcium-binding protein	None	KLI70416.1	
Variants: VaAres1_C_FDSW210055062-1r	G	10468	10468	1	A -> G	1314	SNP (transition)	27.20%	0	calcium-binding protein CDS	calcium-binding protein	None	KLI70416.1	
Variants: VaAres1_C_FDSW210055062-1r	AT	10470	10471	2	GC -> AT	1318 -> 1328	Substitution	27.3% -> 27.4%	0	calcium-binding protein CDS	calcium-binding protein	Substitution	KLI70416.1	S -> N
Variants: VaAres1_C_FDSW210055062-1r	A	10481	10481	1	T -> A	1328	SNP (transversion)	48.10%	0	calcium-binding protein CDS	calcium-binding protein	Substitution	KLI70416.1	S -> T

Variants: VaAres1_C_FDSW210055062-1r	C	10486	10486	1	T -> C	1335	SNP (transition)	34.30%	0	calcium-binding protein CDS	calcium-binding protein	None	KLI70416.1	
Variants: VaAres1_C_FDSW210055062-1r	C	10498	10498	1	T -> C	1317	SNP (transition)	42.90%	0	calcium-binding protein CDS	calcium-binding protein	None	KLI70416.1	
Variants: VaAres1_C_FDSW210055062-1r	A	10534	10534	1	G -> A	1358	SNP (transition)	93.30%	0	calcium-binding protein CDS	calcium-binding protein	None	KLI70416.1	
Variants: VaAres1_C_FDSW210055062-1r	C	10540	10540	1	T -> C	1355	SNP (transition)	96.20%	0	calcium-binding protein CDS	calcium-binding protein	None	KLI70416.1	
Variants: VaAres1_C_FDSW210055062-1r	G	10543	10543	1	C -> G	1343	SNP (transversion)	52.50%	0	calcium-binding protein CDS	calcium-binding protein	None	KLI70416.1	
Variants: VaAres1_C_FDSW210055062-1r	T	10546	10546	1	C -> T	1330	SNP (transition)	96.90%	0	calcium-binding protein CDS	calcium-binding protein	None	KLI70416.1	
Variants: VaAres1_C_FDSW210055062-1r	AGCGGAAACG	10549	10558	9	GTCCACTGTT -> AGCGGAAACG	1315 -> 1328	Substitution	93.2% -> 94.7%	0	calcium-binding protein CDS	calcium-binding protein	Substitution	KLI70416.1	ESTV -> EAET
Variants: VaAres1_C_FDSW210055062-1r	A	10564	10564	1	C -> A	1313	SNP (transversion)	57.70%	0	calcium-binding protein CDS	calcium-binding protein	None	KLI70416.1	
Variants: VaAres1_C_FDSW210055062-1r	G	10564	10564	1	C -> G	1313	SNP (transversion)	40.50%	0	calcium-binding protein CDS	calcium-binding protein	None	KLI70416.1	
Variants: VaAres1_C_FDSW210055062-1r	A	10567	10567	1	G -> A	1317	SNP (transition)	38.70%	0	calcium-binding protein CDS	calcium-binding protein	None	KLI70416.1	
Variants: VaAres1_C_FDSW210055062-1r	TTG	10568	10570	2	CTA -> TTG	1305 -> 1310	Substitution	97.7% -> 98.0%	0	calcium-binding protein CDS	calcium-binding protein	None	KLI70416.1	
Variants: VaAres1_C_FDSW210055062-1r	T	10573	10573	1	C -> T	1287	SNP (transition)	98.40%	0	calcium-binding protein CDS	calcium-binding protein	None	KLI70416.1	
Variants: VaAres1_C_FDSW210055062-1r	T	10576	10576	1	G -> T	1286	SNP (transversion)	98.80%	0	calcium-binding protein CDS	calcium-binding protein	None	KLI70416.1	
Variants: VaAres1_C_FDSW210055062-1r	TCTC	10579	10582	3	CTTG -> TCTC	1274 -> 1287	Substitution	70.6% -> 71.7%	0	calcium-binding protein CDS	calcium-binding protein	None	KLI70416.1	
Variants: VaAres1_C_FDSW210055062-1r	TCTT	10579	10582	3	CTTG -> TCTT	1274 -> 1287	Substitution	25.5% -> 25.7%	0	calcium-binding protein CDS	calcium-binding protein	None	KLI70416.1	
Variants: VaAres1_C_FDSW210055062-1r	T	10596	10596	1	C -> T	1134	SNP (transition)	74.30%	0	calcium-binding protein CDS	calcium-binding protein	Substitution	KLI70416.1	A -> V
Variants: VaAres1_C_FDSW210055062-1r	A	10600	10600	1	T -> A	1099	SNP (transversion)	25.80%	0	calcium-binding protein CDS	calcium-binding protein	None	KLI70416.1	
Variants: VaAres1_C_FDSW210055062-1r	C	10609	10609	1	T -> C	1006	SNP (transition)	99.60%	0	calcium-binding protein CDS	calcium-binding protein	None	KLI70416.1	
Variants: VaAres1_C_FDSW210055062-1r	A	10615	10615	1	G -> A	912	SNP (transition)	99.20%	0	calcium-binding protein CDS	calcium-binding protein	None	KLI70416.1	
Variants: VaAres1_C_FDSW210055062-1r	AAG	10630	10632	3	CTC -> AAG	740 -> 765	Substitution	29.6% -> 30.5%	0	calcium-binding protein CDS	calcium-binding protein	None	KLI70416.1	
Variants: VaphiVaphiSt2_B_A_FDSW210055054-2r	CATC	10108	10111	3	GGTT -> CATC	113 -> 121	Substitution	25.7% -> 30.6%	3.40E-32	calcium-binding protein CDS	calcium-binding protein	Substitution	KLI70416.1	TV -> TI
Variants: VaphiVaphiSt2_B_A_FDSW210055054-2r	C	10129	10129	1	G -> C	154	SNP (transversion)	46.80%	1.20E-128	calcium-binding protein CDS	calcium-binding protein	None	KLI70416.1	

Variants: VaphiVaphiSt2_B_A_FDSW210055054-2r	T	10145	10145	1	C -> T	196	SNP (transition)	40.30%	1.30E-181	calcium-binding protein CDS	calcium-binding protein	None	KLI70416.1	
Variants: VaphiVaphiSt2_B_A_FDSW210055054-2r	C	10153	10153	1	T -> C	216	SNP (transition)	44.00%	1.10E-241	calcium-binding protein CDS	calcium-binding protein	None	KLI70416.1	
Variants: VaphiVaphiSt2_B_A_FDSW210055054-2r	T	10159	10159	1	C -> T	236	SNP (transition)	49.60%	4.3E-317	calcium-binding protein CDS	calcium-binding protein	None	KLI70416.1	
Variants: VaphiVaphiSt2_B_A_FDSW210055054-2r	A	10204	10204	1	C -> A	309	SNP (transversion)	44.30%	0	calcium-binding protein CDS	calcium-binding protein	None	KLI70416.1	
Variants: VaphiVaphiSt2_B_A_FDSW210055054-2r	C	10273	10273	1	T -> C	381	SNP (transition)	34.10%	5.00E-273	calcium-binding protein CDS	calcium-binding protein	None	KLI70416.1	
Variants: VaphiVaphiSt2_B_A_FDSW210055054-2r	G	10282	10282	1	C -> G	396	SNP (transversion)	31.60%	1.70E-257	calcium-binding protein CDS	calcium-binding protein	None	KLI70416.1	
Variants: VaphiVaphiSt2_B_A_FDSW210055054-2r	A	10288	10288	1	G -> A	411	SNP (transition)	25.10%	6.60E-109	calcium-binding protein CDS	calcium-binding protein	None	KLI70416.1	
Variants: VaphiVaphiSt2_B_A_FDSW210055054-2r	T	10315	10315	1	C -> T	492	SNP (transition)	25.60%	3.90E-171	calcium-binding protein CDS	calcium-binding protein	None	KLI70416.1	
Variants: VaphiVaphiSt2_B_A_FDSW210055054-2r	T	10322	10322	1	G -> T	520	SNP (transversion)	43.50%	3.5E-323	calcium-binding protein CDS	calcium-binding protein	Substitution	KLI70416.1	A -> S
Variants: VaphiVaphiSt2_B_A_FDSW210055054-2r	C	10333	10333	1	T -> C	537	SNP (transition)	26.60%	9.90E-197	calcium-binding protein CDS	calcium-binding protein	None	KLI70416.1	
Variants: VaphiVaphiSt2_B_A_FDSW210055054-2r	T	10351	10351	1	A -> T	591	SNP (transversion)	33.70%	0	calcium-binding protein CDS	calcium-binding protein	None	KLI70416.1	
Variants: VaphiVaphiSt2_B_A_FDSW210055054-2r	A	10369	10369	1	G -> A	625	SNP (transition)	35.20%	0	calcium-binding protein CDS	calcium-binding protein	None	KLI70416.1	
Variants: VaphiVaphiSt2_B_A_FDSW210055054-2r	A	10387	10387	1	G -> A	675	SNP (transition)	43.30%	0	calcium-binding protein CDS	calcium-binding protein	None	KLI70416.1	
Variants: VaphiVaphiSt2_B_A_FDSW210055054-2r	C	10396	10396	1	T -> C	692	SNP (transition)	37.00%	0	calcium-binding protein CDS	calcium-binding protein	None	KLI70416.1	
Variants: VaphiVaphiSt2_B_A_FDSW210055054-2r	G	10405	10405	1	C -> G	709	SNP (transversion)	54.20%	0	calcium-binding protein CDS	calcium-binding protein	None	KLI70416.1	
Variants: VaphiVaphiSt2_B_A_FDSW210055054-2r	G	10414	10414	1	A -> G	707	SNP (transition)	26.60%	0	calcium-binding protein CDS	calcium-binding protein	None	KLI70416.1	
Variants: VaphiVaphiSt2_B_A_FDSW210055054-2r	T	10429	10429	1	C -> T	697	SNP (transition)	33.40%	0	calcium-binding protein CDS	calcium-binding protein	None	KLI70416.1	
Variants: VaphiVaphiSt2_B_A_FDSW210055054-2r	G	10441	10441	1	A -> G	656	SNP (transition)	50.60%	0	calcium-binding protein CDS	calcium-binding protein	None	KLI70416.1	
Variants: VaphiVaphiSt2_B_A_FDSW210055054-2r	C	10444	10444	1	A -> C	656	SNP (transversion)	43.80%	0	calcium-binding protein CDS	calcium-binding protein	None	KLI70416.1	
Variants: VaphiVaphiSt2_B_A_FDSW210055054-2r	ACC	10481	10483	2	TCA -> ACC	570 -> 571	Substitution	32.4% -> 32.8%	0	calcium-binding protein CDS	calcium-binding protein	Substitution	KLI70416.1	S -> T
Variants: VaphiVaphiSt2_B_A_FDSW210055054-2r	A	10534	10534	1	G -> A	510	SNP (transition)	89.40%	0	calcium-binding protein CDS	calcium-binding protein	None	KLI70416.1	
Variants: VaphiVaphiSt2_B_A_FDSW210055054-2r	C	10540	10540	1	T -> C	488	SNP (transition)	94.30%	0	calcium-binding protein CDS	calcium-binding protein	None	KLI70416.1	

Variants: VaphiVaphiSt2_B_A_FDSW210055054-2r	G	10543	10543	1	C -> G	473	SNP (transversion)	31.10%	0	calcium-binding protein CDS	calcium-binding protein	None	KLI70416.1	
Variants: VaphiVaphiSt2_B_A_FDSW210055054-2r	T	10546	10546	1	C -> T	466	SNP (transition)	95.10%	0	calcium-binding protein CDS	calcium-binding protein	None	KLI70416.1	
Variants: VaphiVaphiSt2_B_A_FDSW210055054-2r	AGCGGAAACG	10549	10558	9	GTCCACTGTT -> AGCGGAAACG	421 -> 460	Substitution	92.2% -> 93.6%	0	calcium-binding protein CDS	calcium-binding protein	Substitution	KLI70416.1	ESTV -> EAET
Variants: VaphiVaphiSt2_B_A_FDSW210055054-2r	A	10564	10564	1	C -> A	403	SNP (transversion)	73.90%	0	calcium-binding protein CDS	calcium-binding protein	None	KLI70416.1	
Variants: VaphiVaphiSt2_B_A_FDSW210055054-2r	TTG	10568	10570	2	CTA -> TTG	389 -> 394	Substitution	95.7% -> 96.1%	0	calcium-binding protein CDS	calcium-binding protein	None	KLI70416.1	
Variants: VaphiVaphiSt2_B_A_FDSW210055054-2r	T	10573	10573	1	C -> T	382	SNP (transition)	97.60%	0	calcium-binding protein CDS	calcium-binding protein	None	KLI70416.1	
Variants: VaphiVaphiSt2_B_A_FDSW210055054-2r	T	10576	10576	1	G -> T	376	SNP (transversion)	97.90%	0	calcium-binding protein CDS	calcium-binding protein	None	KLI70416.1	
Variants: VaphiVaphiSt2_B_A_FDSW210055054-2r	TCTC	10579	10582	3	CTTG -> TCTC	358 -> 371	Substitution	96.5% -> 97.2%	0	calcium-binding protein CDS	calcium-binding protein	None	KLI70416.1	
Variants: VaphiVaphiSt2_B_A_FDSW210055054-2r	T	10596	10596	1	C -> T	325	SNP (transition)	97.80%	0	calcium-binding protein CDS	calcium-binding protein	Substitution	KLI70416.1	A -> V
Variants: VaphiVaphiSt2_B_A_FDSW210055054-2r	C	10609	10609	1	T -> C	290	SNP (transition)	99.00%	0	calcium-binding protein CDS	calcium-binding protein	None	KLI70416.1	
Variants: VaphiVaphiSt2_B_A_FDSW210055054-2r	A	10615	10615	1	G -> A	267	SNP (transition)	99.30%	0	calcium-binding protein CDS	calcium-binding protein	None	KLI70416.1	
Variants: VaphiSt2_B_FDSW210055056-1r	C	10129	10129	1	G -> C	299	SNP (transversion)	33.80%	5.70E-112	calcium-binding protein CDS	calcium-binding protein	None	KLI70416.1	
Variants: VaphiSt2_B_FDSW210055056-1r	T	10145	10145	1	C -> T	343	SNP (transition)	30.60%	4.00E-173	calcium-binding protein CDS	calcium-binding protein	None	KLI70416.1	
Variants: VaphiSt2_B_FDSW210055056-1r	C	10153	10153	1	T -> C	375	SNP (transition)	29.10%	2.70E-209	calcium-binding protein CDS	calcium-binding protein	None	KLI70416.1	
Variants: VaphiSt2_B_FDSW210055056-1r	T	10159	10159	1	C -> T	396	SNP (transition)	33.10%	5.60E-273	calcium-binding protein CDS	calcium-binding protein	None	KLI70416.1	
Variants: VaphiSt2_B_FDSW210055056-1r	A	10204	10204	1	C -> A	516	SNP (transversion)	36.40%	0	calcium-binding protein CDS	calcium-binding protein	None	KLI70416.1	
Variants: VaphiSt2_B_FDSW210055056-1r	TG	10255	10256	2	GA -> TG	738 -> 739	Substitution	37.3% -> 37.5%	4.50E-265	calcium-binding protein CDS	calcium-binding protein	Substitution	KLI70416.1	VT -> VA
Variants: VaphiSt2_B_FDSW210055056-1r	C	10273	10273	1	T -> C	730	SNP (transition)	40.30%	0	calcium-binding protein CDS	calcium-binding protein	None	KLI70416.1	
Variants: VaphiSt2_B_FDSW210055056-1r	G	10282	10282	1	C -> G	756	SNP (transversion)	39.60%	0	calcium-binding protein CDS	calcium-binding protein	None	KLI70416.1	
Variants: VaphiSt2_B_FDSW210055056-1r	A	10288	10288	1	G -> A	769	SNP (transition)	38.80%	0	calcium-binding protein CDS	calcium-binding protein	None	KLI70416.1	
Variants: VaphiSt2_B_FDSW210055056-1r	T	10315	10315	1	C -> T	940	SNP (transition)	26.80%	0	calcium-binding protein CDS	calcium-binding protein	None	KLI70416.1	
Variants: VaphiSt2_B_FDSW210055056-1r	T	10322	10322	1	G -> T	997	SNP (transversion)	51.10%	0	calcium-binding protein CDS	calcium-binding protein	Substitution	KLI70416.1	A -> S

Variants: VaphiSt2_B_FDSW210055056-1r	C	10333	10333	1	T -> C	1049	SNP (transition)	35.40%	0	calcium-binding protein CDS	calcium-binding protein	None	KLI70416.1	
Variants: VaphiSt2_B_FDSW210055056-1r	T	10351	10351	1	A -> T	1163	SNP (transversion)	30.90%	0	calcium-binding protein CDS	calcium-binding protein	None	KLI70416.1	
Variants: VaphiSt2_B_FDSW210055056-1r	A	10369	10369	1	G -> A	1187	SNP (transition)	35.60%	0	calcium-binding protein CDS	calcium-binding protein	None	KLI70416.1	
Variants: VaphiSt2_B_FDSW210055056-1r	A	10387	10387	1	G -> A	1217	SNP (transition)	42.10%	0	calcium-binding protein CDS	calcium-binding protein	None	KLI70416.1	
Variants: VaphiSt2_B_FDSW210055056-1r	C	10396	10396	1	T -> C	1263	SNP (transition)	32.30%	0	calcium-binding protein CDS	calcium-binding protein	None	KLI70416.1	
Variants: VaphiSt2_B_FDSW210055056-1r	G	10405	10405	1	C -> G	1278	SNP (transversion)	50.30%	0	calcium-binding protein CDS	calcium-binding protein	None	KLI70416.1	
Variants: VaphiSt2_B_FDSW210055056-1r	G	10414	10414	1	A -> G	1299	SNP (transition)	26.70%	0	calcium-binding protein CDS	calcium-binding protein	None	KLI70416.1	
Variants: VaphiSt2_B_FDSW210055056-1r	T	10429	10429	1	C -> T	1313	SNP (transition)	29.20%	0	calcium-binding protein CDS	calcium-binding protein	None	KLI70416.1	
Variants: VaphiSt2_B_FDSW210055056-1r	T	10435	10435	1	G -> T	1316	SNP (transversion)	25.20%	0	calcium-binding protein CDS	calcium-binding protein	None	KLI70416.1	
Variants: VaphiSt2_B_FDSW210055056-1r	G	10441	10441	1	A -> G	1276	SNP (transition)	46.90%	0	calcium-binding protein CDS	calcium-binding protein	None	KLI70416.1	
Variants: VaphiSt2_B_FDSW210055056-1r	C	10444	10444	1	A -> C	1255	SNP (transversion)	44.50%	0	calcium-binding protein CDS	calcium-binding protein	None	KLI70416.1	
Variants: VaphiSt2_B_FDSW210055056-1r	ACC	10481	10483	2	TCA -> ACC	1126 -> 1140	Substitution	30.4% -> 30.8%	0	calcium-binding protein CDS	calcium-binding protein	Substitution	KLI70416.1	S -> T
Variants: VaphiSt2_B_FDSW210055056-1r	A	10534	10534	1	G -> A	1058	SNP (transition)	90.00%	0	calcium-binding protein CDS	calcium-binding protein	None	KLI70416.1	
Variants: VaphiSt2_B_FDSW210055056-1r	C	10540	10540	1	T -> C	1056	SNP (transition)	93.30%	0	calcium-binding protein CDS	calcium-binding protein	None	KLI70416.1	
Variants: VaphiSt2_B_FDSW210055056-1r	G	10543	10543	1	C -> G	1034	SNP (transversion)	31.80%	0	calcium-binding protein CDS	calcium-binding protein	None	KLI70416.1	
Variants: VaphiSt2_B_FDSW210055056-1r	T	10546	10546	1	C -> T	1029	SNP (transition)	94.60%	0	calcium-binding protein CDS	calcium-binding protein	None	KLI70416.1	
Variants: VaphiSt2_B_FDSW210055056-1r	AGCGGAAACG	10549	10558	9	GTCCACTGTT -> AGCGGAAACG	1000 -> 1017	Substitution	90.6% -> 92.9%	0	calcium-binding protein CDS	calcium-binding protein	Substitution	KLI70416.1	ESTV -> EAET
Variants: VaphiSt2_B_FDSW210055056-1r	A	10564	10564	1	C -> A	1005	SNP (transversion)	74.70%	0	calcium-binding protein CDS	calcium-binding protein	None	KLI70416.1	
Variants: VaphiSt2_B_FDSW210055056-1r	TTG	10568	10570	2	CTA -> TTG	996 -> 1003	Substitution	96.5% -> 96.6%	0	calcium-binding protein CDS	calcium-binding protein	None	KLI70416.1	
Variants: VaphiSt2_B_FDSW210055056-1r	T	10573	10573	1	C -> T	987	SNP (transition)	96.40%	0	calcium-binding protein CDS	calcium-binding protein	None	KLI70416.1	
Variants: VaphiSt2_B_FDSW210055056-1r	T	10576	10576	1	G -> T	977	SNP (transversion)	96.50%	0	calcium-binding protein CDS	calcium-binding protein	None	KLI70416.1	
Variants: VaphiSt2_B_FDSW210055056-1r	TCTC	10579	10582	3	CTTG -> TCTC	938 -> 956	Substitution	95.4% -> 95.5%	0	calcium-binding protein CDS	calcium-binding protein	None	KLI70416.1	

Variants: VaphiSt2_B_FDSW210055056-1r	T	10596	10596	1	C -> T	867	SNP (transition)	97.80%	0	calcium-binding protein CDS	calcium-binding protein	Substitution	KLI70416.1	A -> V
Variants: VaphiSt2_B_FDSW210055056-1r	C	10609	10609	1	T -> C	782	SNP (transition)	98.50%	0	calcium-binding protein CDS	calcium-binding protein	None	KLI70416.1	
Variants: VaphiSt2_B_FDSW210055056-1r	A	10615	10615	1	G -> A	734	SNP (transition)	98.80%	0	calcium-binding protein CDS	calcium-binding protein	None	KLI70416.1	

Contig 29

Variant	Name	Minimum	Maximum	Length	Change	Coverage	Polymorphism Type	Variant Frequency	Variant P-Value (approximate)
Variants: VaphiVaphiSt2_B_A_FDSW210055054-2r	G	21	21	1	A -> G	207	SNP (transition)	32.40%	2.30E-126
Variants: VaphiVaphiSt2_B_A_FDSW210055054-2r	T	23	23	1	A -> T	205	SNP (transversion)	27.30%	4.70E-101

Contig 34

Variant	Name	Minimum	Maximum	Length	Change	Coverage	Polymorphism Type	Variant Frequency	Variant P-Value (approximate)	Amino Acid Change	CDS	product	Protein Effect	protein_id
Variants: VaAphrodite_A_FDSW210055057-2r	C	3880	3880	1	T -> C	120	SNP (transition)	27.50%	1.60E-50		nucleoside permease CDS	nucleoside permease	None	KLI70272.1
Variants: VaAphrodite_A_FDSW210055057-2r	G	3957	3957	1	A -> G	175	SNP (transition)	45.10%	1.90E-202		nucleoside permease CDS	nucleoside permease	None	KLI70272.1
Variants: VaAphrodite_A_FDSW210055057-2r	AT	3996	3997	2	GC -> AT	184 -> 185	Substitution	45.9% -> 46.2%	8.20E-236	RH -> RY	nucleoside permease CDS	nucleoside permease	Substitution	KLI70272.1
Variants: VaAphrodite_A_FDSW210055057-2r	G	4024	4024	1	T -> G	178	SNP (transversion)	45.50%	4.30E-224	E -> D	nucleoside permease CDS	nucleoside permease	Substitution	KLI70272.1
Variants: VaAphrodite_B_FDSW210055058-2r	GC	2	3	2	TG -> GC	64 -> 65	Substitution	25.0% -> 27.7%	3.10E-45					
Variants: VaAphrodite_B_FDSW210055058-2r	G	3957	3957	1	A -> G	188	SNP (transition)	29.80%	1.30E-142		nucleoside permease CDS	nucleoside permease	None	KLI70272.1
Variants: VaAphrodite_B_FDSW210055058-2r	AT	3996	3997	2	GC -> AT	207 -> 208	Substitution	31.9% -> 32.2%	2.60E-166	RH -> RY	nucleoside permease CDS	nucleoside permease	Substitution	KLI70272.1
Variants: VaAphrodite_B_FDSW210055058-2r	G	4024	4024	1	T -> G	205	SNP (transversion)	33.20%	5.00E-163	E -> D	nucleoside permease CDS	nucleoside permease	Substitution	KLI70272.1
Variants: VaAphrodite_C_FDSW210055059-1r	GC	2	3	2	TG -> GC	107 -> 108	Substitution	27.1% -> 27.8%	3.80E-76					
Variants: VaAphrodite_C_FDSW210055059-1r	G	3957	3957	1	A -> G	284	SNP (transition)	35.90%	2.50E-268		nucleoside permease CDS	nucleoside permease	None	KLI70272.1
Variants: VaAphrodite_C_FDSW210055059-1r	AT	3996	3997	2	GC -> AT	280 -> 283	Substitution	35.3% -> 35.7%	3.10E-262	RH -> RY	nucleoside permease CDS	nucleoside permease	Substitution	KLI70272.1
Variants: VaAphrodite_C_FDSW210055059-1r	G	4024	4024	1	T -> G	287	SNP (transversion)	38.00%	5.10E-279	E -> D	nucleoside permease CDS	nucleoside permease	Substitution	KLI70272.1
Variants: VaAres1_A_FDSW210055060-2r	G	3957	3957	1	A -> G	128	SNP (transition)	48.40%	8.60E-162		nucleoside permease CDS	nucleoside permease	None	KLI70272.1
Variants: VaAres1_A_FDSW210055060-2r	AT	3996	3997	2	GC -> AT	131 -> 132	Substitution	51.5% -> 51.9%	4.20E-180	RH -> RY	nucleoside permease CDS	nucleoside permease	Substitution	KLI70272.1
Variants: VaAres1_A_FDSW210055060-2r	G	4024	4024	1	T -> G	132	SNP (transversion)	47.00%	4.60E-173	E -> D	nucleoside permease CDS	nucleoside permease	Substitution	KLI70272.1
Variants: VaAres1_B_FDSW210055061-2r	G	3957	3957	1	A -> G	142	SNP (transition)	34.50%	9.30E-129		nucleoside permease CDS	nucleoside permease	None	KLI70272.1
Variants: VaAres1_B_FDSW210055061-2r	AT	3996	3997	2	GC -> AT	154 -> 155	Substitution	29.0% -> 29.2%	2.40E-114	RH -> RY	nucleoside permease CDS	nucleoside permease	Substitution	KLI70272.1
Variants: VaAres1_B_FDSW210055061-2r	G	4024	4024	1	T -> G	161	SNP (transversion)	28.60%	1.80E-116	E -> D	nucleoside permease CDS	nucleoside permease	Substitution	KLI70272.1
Variants: VaAres1_C_FDSW210055062-1r	GC	2	3	2	TG -> GC	64 -> 65	Substitution	26.2% -> 28.1%	2.30E-48					
Variants: VaAres1_C_FDSW210055062-1r	G	3957	3957	1	A -> G	187	SNP (transition)	44.40%	2.10E-228		nucleoside permease CDS	nucleoside permease	None	KLI70272.1
Variants: VaAres1_C_FDSW210055062-1r	AT	3996	3997	2	GC -> AT	190	Substitution	41.60%	1.50E-214	RH -> RY	nucleoside permease CDS	nucleoside permease	Substitution	KLI70272.1
Variants: VaAres1_C_FDSW210055062-1r	G	4024	4024	1	T -> G	199	SNP (transversion)	38.70%	2.10E-198	E -> D	nucleoside permease CDS	nucleoside permease	Substitution	KLI70272.1
Variants: VaphiVaphiSt2_B_A_FDSW210055054-2r	GC	2	3	2	TG -> GC	71	Substitution	25.40%	1.20E-43					
Variants: VaphiVaphiSt2_B_A_FDSW210055054-2r	G	3957	3957	1	A -> G	159	SNP (transition)	40.90%	3.20E-176		nucleoside permease CDS	nucleoside permease	None	KLI70272.1
Variants: VaphiVaphiSt2_B_A_FDSW210055054-2r	AT	3996	3997	2	GC -> AT	170 -> 171	Substitution	37.4% -> 37.6%	1.20E-170	RH -> RY	nucleoside permease CDS	nucleoside permease	Substitution	KLI70272.1
Variants: VaphiVaphiSt2_B_A_FDSW210055054-2r	G	4024	4024	1	T -> G	164	SNP (transversion)	39.60%	4.10E-175	E -> D	nucleoside permease CDS	nucleoside permease	Substitution	KLI70272.1
Variants: VaphiSt2_B_FDSW210055056-1r	GC	2	3	2	TG -> GC	135 -> 136	Substitution	27.4% -> 28.7%	4.50E-99					
Variants: VaphiSt2_B_FDSW210055056-1r	G	3957	3957	1	A -> G	291	SNP (transition)	34.40%	9.30E-251		nucleoside permease CDS	nucleoside permease	None	KLI70272.1

Variants: VaphiSt2_B_FDSW210055056-1r	AT	3996	3997	2	GC -> AT	307 -> 308	Substitution	33.4% -> 33.6%		6.40E-257	RH -> RY	nucleoside permease CDS	nucleoside permease	Substitution	KLI70272.1
Variants: VaphiSt2_B_FDSW210055056-1r	G	4024	4024	1	T -> G	308	SNP (transversion)	34.10%		3.00E-273	E -> D	nucleoside permease CDS	nucleoside permease	Substitution	KLI70272.1

Contig 40

Variant	Name	Minimum	Maximum	Length	Change	Coverage	Polymorphism Type	Variant Frequency	Variant P-Value (approximate)
Variants: VaAphrodite_A_FDSW210055057-2r	C	66081	66081	1	G -> C	448	SNP (transversion)	39.70%	0
Variants: VaAphrodite_A_FDSW210055057-2r	G	66087	66087	1	A -> G	460	SNP (transition)	38.90%	0
Variants: VaAphrodite_A_FDSW210055057-2r	A	66092	66092	1	G -> A	463	SNP (transition)	39.30%	0
Variants: VaAphrodite_A_FDSW210055057-2r	T	66114	66114	1	C -> T	498	SNP (transition)	44.20%	0
Variants: VaAphrodite_B_FDSW210055058-2r	C	66081	66081	1	G -> C	350	SNP (transversion)	42.00%	0
Variants: VaAphrodite_B_FDSW210055058-2r	G	66087	66087	1	A -> G	373	SNP (transition)	42.60%	0
Variants: VaAphrodite_B_FDSW210055058-2r	A	66092	66092	1	G -> A	375	SNP (transition)	43.20%	0
Variants: VaAphrodite_B_FDSW210055058-2r	T	66114	66114	1	C -> T	432	SNP (transition)	45.60%	0
Variants: VaAphrodite_C_FDSW210055059-1r	C	66081	66081	1	G -> C	497	SNP (transversion)	44.30%	0
Variants: VaAphrodite_C_FDSW210055059-1r	G	66087	66087	1	A -> G	513	SNP (transition)	44.60%	0
Variants: VaAphrodite_C_FDSW210055059-1r	A	66092	66092	1	G -> A	519	SNP (transition)	45.90%	0
Variants: VaAphrodite_C_FDSW210055059-1r	T	66114	66114	1	C -> T	562	SNP (transition)	47.50%	0
Variants: VaAres1_A_FDSW210055060-2r	C	66081	66081	1	G -> C	303	SNP (transversion)	43.90%	4.9E-324
Variants: VaAres1_A_FDSW210055060-2r	G	66087	66087	1	A -> G	304	SNP (transition)	45.10%	0
Variants: VaAres1_A_FDSW210055060-2r	A	66092	66092	1	G -> A	307	SNP (transition)	46.60%	0
Variants: VaAres1_A_FDSW210055060-2r	T	66114	66114	1	C -> T	320	SNP (transition)	49.10%	0
Variants: VaAres1_B_FDSW210055061-2r	C	66081	66081	1	G -> C	393	SNP (transversion)	39.70%	0
Variants: VaAres1_B_FDSW210055061-2r	G	66087	66087	1	A -> G	400	SNP (transition)	40.00%	0
Variants: VaAres1_B_FDSW210055061-2r	A	66092	66092	1	G -> A	405	SNP (transition)	40.70%	0
Variants: VaAres1_B_FDSW210055061-2r	T	66114	66114	1	C -> T	423	SNP (transition)	42.80%	0
Variants: VaAres1_C_FDSW210055062-1r	C	66081	66081	1	G -> C	464	SNP (transversion)	38.10%	0
Variants: VaAres1_C_FDSW210055062-1r	G	66087	66087	1	A -> G	480	SNP (transition)	39.20%	0
Variants: VaAres1_C_FDSW210055062-1r	A	66092	66092	1	G -> A	486	SNP (transition)	39.70%	0
Variants: VaAres1_C_FDSW210055062-1r	T	66114	66114	1	C -> T	520	SNP (transition)	40.80%	0
Variants: VaphiVaphiSt2_B_A_FDSW210055054-2r	C	66081	66081	1	G -> C	252	SNP (transversion)	41.70%	3.20E-274
Variants: VaphiVaphiSt2_B_A_FDSW210055054-2r	G	66087	66087	1	A -> G	269	SNP (transition)	42.80%	8.00E-302
Variants: VaphiVaphiSt2_B_A_FDSW210055054-2r	A	66092	66092	1	G -> A	274	SNP (transition)	42.30%	3.30E-292
Variants: VaphiVaphiSt2_B_A_FDSW210055054-2r	T	66114	66114	1	C -> T	298	SNP (transition)	48.70%	0
Variants: VaphiSt2_B_FDSW210055056-1r	C	66081	66081	1	G -> C	537	SNP (transversion)	38.90%	0

Variants: VaphiSt2_B_FDSW210055056-1r	G	66087	66087	1	A -> G	555	SNP (transition)	40.40%	0
Variants: VaphiSt2_B_FDSW210055056-1r	A	66092	66092	1	G -> A	568	SNP (transition)	40.70%	0
Variants: VaphiSt2_B_FDSW210055056-1r	T	66114	66114	1	C -> T	598	SNP (transition)	44.10%	0

Contig 46

Variant	Name	Minimum	Maximum	Length	Change	Coverage	Polymorphism Type	Variant Frequency	Variant P-Value (approximate)
Variants: VaAphrodite_A_FDSW210055057-2r	G	1920	1920	1	A -> G	162	SNP (transition)	30.90%	1.70E-98
Variants: VaAphrodite_A_FDSW210055057-2r	A	1999	1999	1	C -> A	181	SNP (transversion)	34.80%	8.60E-109
Variants: VaAphrodite_A_FDSW210055057-2r	G	2012	2012	1	A -> G	177	SNP (transition)	31.10%	6.00E-92
Variants: VaAphrodite_B_FDSW210055058-2r	G	1920	1920	1	A -> G	157	SNP (transition)	31.80%	2.90E-114
Variants: VaAphrodite_B_FDSW210055058-2r	A	1999	1999	1	C -> A	177	SNP (transversion)	28.80%	2.00E-83
Variants: VaAres1_A_FDSW210055060-2r	G	1920	1920	1	A -> G	95	SNP (transition)	31.60%	4.30E-60
Variants: VaAres1_A_FDSW210055060-2r	A	1999	1999	1	C -> A	97	SNP (transversion)	32.00%	2.50E-65
Variants: VaAres1_B_FDSW210055061-2r	A	1890	1890	1	G -> A	106	SNP (transition)	27.40%	1.40E-67
Variants: VaAres1_B_FDSW210055061-2r	G	1920	1920	1	A -> G	122	SNP (transition)	36.90%	1.80E-124
Variants: VaAres1_B_FDSW210055061-2r	A	1999	1999	1	C -> A	138	SNP (transversion)	34.80%	5.50E-112
Variants: VaAres1_B_FDSW210055061-2r	G	2012	2012	1	A -> G	127	SNP (transition)	29.10%	6.60E-76
Variants: VaAres1_C_FDSW210055062-1r	G	1920	1920	1	A -> G	167	SNP (transition)	31.70%	8.30E-137
Variants: VaAres1_C_FDSW210055062-1r	A	1999	1999	1	C -> A	182	SNP (transversion)	39.60%	3.30E-136
Variants: VaAres1_C_FDSW210055062-1r	G	2012	2012	1	A -> G	175	SNP (transition)	33.10%	2.70E-87
Variants: VaphiVaphiSt2_B_A_FDSW210055054-2r	A	1999	1999	1	C -> A	122	SNP (transversion)	34.40%	3.30E-81
Variants: VaphiSt2_B_FDSW210055056-1r	G	1920	1920	1	A -> G	249	SNP (transition)	25.30%	7.10E-130
Variants: VaphiSt2_B_FDSW210055056-1r	A	1999	1999	1	C -> A	275	SNP (transversion)	37.80%	1.60E-224
Variants: VaphiSt2_B_FDSW210055056-1r	G	2012	2012	1	A -> G	269	SNP (transition)	30.50%	1.00E-151

Contig 49

Variant	Name	Minimum	Maximum	Length	Change	Coverage	Polymorphism Type	Variant Frequency	Variant P-Value (approximate)	Amino Acid Change	CDS	product	Protein Effect	protein_id
Variants: VaAphrodite_A_FDSW210055057-2r	C	2	2	1	A -> C	781	SNP (transversion)	61.50%	0		hypothetical protein CDS	hypothetical protein	None	KLI70043.1
Variants: VaAphrodite_A_FDSW210055057-2r	C	8	8	1	G -> C	767	SNP (transversion)	60.20%	0	D -> E	hypothetical protein CDS	hypothetical protein	Substitution	KLI70043.1
Variants: VaAphrodite_A_FDSW210055057-2r	A	47	47	1	G -> A	604	SNP (transition)	58.80%	0		hypothetical protein CDS	hypothetical protein	None	KLI70043.1
Variants: VaAphrodite_A_FDSW210055057-2r	A	53	53	1	G -> A	589	SNP (transition)	60.60%	0		hypothetical protein CDS	hypothetical protein	None	KLI70043.1
Variants: VaAphrodite_A_FDSW210055057-2r	A	56	56	1	G -> A	579	SNP (transition)	67.90%	0		hypothetical protein CDS	hypothetical protein	None	KLI70043.1
Variants: VaAphrodite_A_FDSW210055057-2r	G	59	59	1	A -> G	559	SNP (transition)	70.30%	0		hypothetical protein CDS	hypothetical protein	None	KLI70043.1
Variants: VaAphrodite_A_FDSW210055057-2r	G	62	62	1	A -> G	541	SNP (transition)	69.50%	0		hypothetical protein CDS	hypothetical protein	None	KLI70043.1
Variants: VaAphrodite_A_FDSW210055057-2r	G	73	73	1	A -> G	491	SNP (transition)	67.20%	0		hypothetical protein CDS	hypothetical protein	None	KLI70043.1
Variants: VaAphrodite_A_FDSW210055057-2r	G	101	101	1	A -> G	369	SNP (transition)	55.80%	0		hypothetical protein CDS	hypothetical protein	None	KLI70043.1
Variants: VaAphrodite_A_FDSW210055057-2r	CG	109	110	2	TC -> CG	322 -> 328	Substitution	46.9% -> 47.3%	4.30E-195	AI -> AV	hypothetical protein CDS	hypothetical protein	Substitution	KLI70043.1
Variants: VaAphrodite_A_FDSW210055057-2r	AT	113	114	2	CG -> AT	301 -> 307	Substitution	42.5% -> 44.3%	4.50E-121	T -> N	hypothetical protein CDS	hypothetical protein	Substitution	KLI70043.1
Variants: VaAphrodite_A_FDSW210055057-2r	G	341	341	1	A -> G	200	SNP (transition)	32.50%	3.40E-168		hypothetical protein CDS	hypothetical protein	None	KLI70043.1
Variants: VaAphrodite_A_FDSW210055057-2r	T	348	348	1	C -> T	246	SNP (transition)	34.60%	1.20E-230	G -> D	hypothetical protein CDS	hypothetical protein	Substitution	KLI70043.1
Variants: VaAphrodite_A_FDSW210055057-2r	G	359	359	1	A -> G	327	SNP (transition)	30.60%	1.20E-264		hypothetical protein CDS	hypothetical protein	None	KLI70043.1
Variants: VaAphrodite_A_FDSW210055057-2r	T	371	371	1	C -> T	430	SNP (transition)	26.00%	3.30E-298		hypothetical protein CDS	hypothetical protein	None	KLI70043.1
Variants: VaAphrodite_A_FDSW210055057-2r	A	374	374	1	G -> A	461	SNP (transition)	42.70%	0		hypothetical protein CDS	hypothetical protein	None	KLI70043.1
Variants: VaAphrodite_A_FDSW210055057-2r	C	386	386	1	T -> C	590	SNP (transition)	76.60%	0		hypothetical protein CDS	hypothetical protein	None	KLI70043.1
Variants: VaAphrodite_A_FDSW210055057-2r	T	392	392	1	G -> T	641	SNP (transversion)	74.60%	0	N -> K	hypothetical protein CDS	hypothetical protein	Substitution	KLI70043.1
Variants: VaAphrodite_A_FDSW210055057-2r	A	395	395	1	G -> A	670	SNP (transition)	63.30%	0		hypothetical protein CDS	hypothetical protein	None	KLI70043.1
Variants: VaAphrodite_A_FDSW210055057-2r	A	407	407	1	G -> A	758	SNP (transition)	37.50%	0		hypothetical protein CDS	hypothetical protein	None	KLI70043.1
Variants: VaAphrodite_A_FDSW210055057-2r	C	413	413	1	T -> C	815	SNP (transition)	82.10%	0		hypothetical protein CDS	hypothetical protein	None	KLI70043.1
Variants: VaAphrodite_A_FDSW210055057-2r	T	416	416	1	A -> T	840	SNP (transversion)	65.50%	0		hypothetical protein CDS	hypothetical protein	None	KLI70043.1
Variants: VaAphrodite_A_FDSW210055057-2r	CGC	419	421	2	GGT -> CGC	858 -> 875	Substitution	39.5% -> 40.3%	0	T -> A	hypothetical protein CDS	hypothetical protein	Substitution	KLI70043.1
Variants: VaAphrodite_A_FDSW210055057-2r	C	421	421	1	T -> C	875	SNP (transition)	32.30%	0	T -> A	hypothetical protein CDS	hypothetical protein	Substitution	KLI70043.1
Variants: VaAphrodite_A_FDSW210055057-2r	T	422	422	1	A -> T	885	SNP (transversion)	74.70%	0		hypothetical protein CDS	hypothetical protein	None	KLI70043.1
Variants: VaAphrodite_A_FDSW210055057-2r	T	425	425	1	C -> T	919	SNP (transition)	56.60%	0		hypothetical protein CDS	hypothetical protein	None	KLI70043.1
Variants: VaAphrodite_A_FDSW210055057-2r	G	428	428	1	A -> G	936	SNP (transition)	80.00%	0		hypothetical protein CDS	hypothetical protein	None	KLI70043.1
Variants: VaAphrodite_A_FDSW210055057-2r	C	452	452	1	T -> C	1184	SNP (transition)	46.50%	0		hypothetical protein CDS	hypothetical protein	None	KLI70043.1
Variants: VaAphrodite_A_FDSW210055057-2r	A	455	455	1	G -> A	1220	SNP (transition)	40.60%	0		hypothetical protein CDS	hypothetical protein	None	KLI70043.1

Variants: VaAphrodite_A_FDSW210055057-2r	A	470	470	1	G -> A	1359	SNP (transition)	38.30%	0		hypothetical protein CDS	hypothetical protein	None	KLI700 43.1
Variants: VaAphrodite_A_FDSW210055057-2r	G	473	473	1	C -> G	1389	SNP (transversion)	51.10%	0		hypothetical protein CDS	hypothetical protein	None	KLI700 43.1
Variants: VaAphrodite_A_FDSW210055057-2r	G	479	479	1	C -> G	1451	SNP (transversion)	60.90%	0		hypothetical protein CDS	hypothetical protein	None	KLI700 43.1
Variants: VaAphrodite_A_FDSW210055057-2r	TA G	482	484	2	CAA -> TAG	1467 -> 1490	Substitution	26.2% -> 26.7%	0		hypothetical protein CDS	hypothetical protein	None	KLI700 43.1
Variants: VaAphrodite_A_FDSW210055057-2r	G	484	484	1	A -> G	1490	SNP (transition)	28.40%	0		hypothetical protein CDS	hypothetical protein	None	KLI700 43.1
Variants: VaAphrodite_A_FDSW210055057-2r	GG T	485	487	2	CGC -> GGT	1494 -> 1508	Substitution	27.4% -> 27.5%	0	A -> T	hypothetical protein CDS	hypothetical protein	Substituti on	KLI700 43.1
Variants: VaAphrodite_A_FDSW210055057-2r	A	497	497	1	G -> A	1549	SNP (transition)	28.00%	0		hypothetical protein CDS	hypothetical protein	None	KLI700 43.1
Variants: VaAphrodite_A_FDSW210055057-2r	C	509	509	1	G -> C	1586	SNP (transversion)	56.00%	0		hypothetical protein CDS	hypothetical protein	None	KLI700 43.1
Variants: VaAphrodite_A_FDSW210055057-2r	G	530	530	1	C -> G	1669	SNP (transversion)	36.40%	0		hypothetical protein CDS	hypothetical protein	None	KLI700 43.1
Variants: VaAphrodite_A_FDSW210055057-2r	A	563	563	1	G -> A	1735	SNP (transition)	32.60%	0		hypothetical protein CDS	hypothetical protein	None	KLI700 43.1
Variants: VaAphrodite_A_FDSW210055057-2r	A	608	608	1	G -> A	1837	SNP (transition)	43.20%	0		hypothetical protein CDS	hypothetical protein	None	KLI700 43.1
Variants: VaAphrodite_A_FDSW210055057-2r	G	614	614	1	A -> G	1845	SNP (transition)	35.00%	0		hypothetical protein CDS	hypothetical protein	None	KLI700 43.1
Variants: VaAphrodite_A_FDSW210055057-2r	T	623	623	1	C -> T	1828	SNP (transition)	28.70%	0		hypothetical protein CDS	hypothetical protein	None	KLI700 43.1
Variants: VaAphrodite_A_FDSW210055057-2r	C	628	628	1	T -> C	1811	SNP (transition)	44.10%	0	N -> D	hypothetical protein CDS	hypothetical protein	Substituti on	KLI700 43.1
Variants: VaAphrodite_A_FDSW210055057-2r	A	644	644	1	G -> A	1784	SNP (transition)	25.40%	0		hypothetical protein CDS	hypothetical protein	None	KLI700 43.1
Variants: VaAphrodite_A_FDSW210055057-2r	G	671	671	1	A -> G	1698	SNP (transition)	30.90%	0		hypothetical protein CDS	hypothetical protein	None	KLI700 43.1
Variants: VaAphrodite_A_FDSW210055057-2r	T	675	675	1	C -> T	1679	SNP (transition)	28.50%	0	S -> N	hypothetical protein CDS	hypothetical protein	Substituti on	KLI700 43.1
Variants: VaAphrodite_A_FDSW210055057-2r	A	677	677	1	G -> A	1642	SNP (transition)	40.30%	0		hypothetical protein CDS	hypothetical protein	None	KLI700 43.1
Variants: VaAphrodite_A_FDSW210055057-2r	A	722	722	1	G -> A	1405	SNP (transition)	30.00%	0		hypothetical protein CDS	hypothetical protein	None	KLI700 43.1
Variants: VaAphrodite_A_FDSW210055057-2r	T	728	728	1	C -> T	1357	SNP (transition)	26.20%	0		hypothetical protein CDS	hypothetical protein	None	KLI700 43.1
Variants: VaAphrodite_A_FDSW210055057-2r	A	737	737	1	G -> A	1273	SNP (transition)	70.20%	0		hypothetical protein CDS	hypothetical protein	None	KLI700 43.1
Variants: VaAphrodite_A_FDSW210055057-2r	A	745	745	1	C -> A	1206	SNP (transversion)	26.70%	0	A -> S	hypothetical protein CDS	hypothetical protein	Substituti on	KLI700 43.1
Variants: VaAphrodite_A_FDSW210055057-2r	C	752	752	1	G -> C	1183	SNP (transversion)	26.60%	0		hypothetical protein CDS	hypothetical protein	None	KLI700 43.1
Variants: VaAphrodite_A_FDSW210055057-2r	A	755	755	1	C -> A	1156	SNP (transversion)	32.30%	0		hypothetical protein CDS	hypothetical protein	None	KLI700 43.1
Variants: VaAphrodite_A_FDSW210055057-2r	A	767	767	1	G -> A	1030	SNP (transition)	27.40%	0		hypothetical protein CDS	hypothetical protein	None	KLI700 43.1
Variants: VaAphrodite_A_FDSW210055057-2r	G	806	806	1	C -> G	881	SNP (transversion)	73.10%	0		hypothetical protein CDS	hypothetical protein	None	KLI700 43.1
Variants: VaAphrodite_A_FDSW210055057-2r	C	823	823	1	T -> C	854	SNP (transition)	58.40%	0	I -> V	hypothetical protein CDS	hypothetical protein	Substituti on	KLI700 43.1
Variants: VaAphrodite_A_FDSW210055057-2r	T	834	834	1	G -> T	826	SNP (transversion)	57.30%	0	T -> K	hypothetical protein CDS	hypothetical protein	Substituti on	KLI700 43.1
Variants: VaAphrodite_A_FDSW210055057-2r	C	844	844	1	T -> C	802	SNP (transition)	71.90%	0	T -> A	hypothetical protein CDS	hypothetical protein	Substituti on	KLI700 43.1
Variants: VaAphrodite_A_FDSW210055057-2r	A	854	854	1	G -> A	812	SNP (transition)	64.50%	0		hypothetical protein CDS	hypothetical protein	None	KLI700 43.1
Variants: VaAphrodite_A_FDSW210055057-2r	CA	865	866	2	GG -> CA	813 -> 815	Substitution	29.8% -> 30.1%	0	IQ -> IE	hypothetical protein CDS	hypothetical protein	Substituti on	KLI700 43.1
Variants: VaAphrodite_A_FDSW210055057-2r	A	874	874	1	G -> A	812	SNP (transition)	26.00%	1.30E-307		hypothetical protein CDS	hypothetical protein	None	KLI700 43.1

Variants: VaAphrodite_A_FDSW210055057-2r	A	881	881	1	G -> A	817	SNP (transition)	44.80%	0		hypothetical protein CDS	hypothetical protein	None	KLI700 43.1
Variants: VaAphrodite_A_FDSW210055057-2r	A	887	887	1	G -> A	829	SNP (transition)	79.00%	0		hypothetical protein CDS	hypothetical protein	None	KLI700 43.1
Variants: VaAphrodite_A_FDSW210055057-2r	A	890	890	1	G -> A	829	SNP (transition)	27.30%	2.0E-312		hypothetical protein CDS	hypothetical protein	None	KLI700 43.1
Variants: VaAphrodite_A_FDSW210055057-2r	G	896	896	1	A -> G	851	SNP (transition)	79.90%	0		hypothetical protein CDS	hypothetical protein	None	KLI700 43.1
Variants: VaAphrodite_A_FDSW210055057-2r	G	908	908	1	A -> G	880	SNP (transition)	30.50%	0		hypothetical protein CDS	hypothetical protein	None	KLI700 43.1
Variants: VaAphrodite_A_FDSW210055057-2r	GA C	941	943	2	AAT -> GAC	973 -> 974	Substitution	27.00%	0	I -> V	hypothetical protein CDS	hypothetical protein	Substituti on	KLI700 43.1
Variants: VaAphrodite_A_FDSW210055057-2r	C	943	943	1	T -> C	974	SNP (transition)	55.50%	0	I -> V	hypothetical protein CDS	hypothetical protein	Substituti on	KLI700 43.1
Variants: VaAphrodite_A_FDSW210055057-2r	A	944	944	1	C -> A	979	SNP (transversion)	52.90%	0		hypothetical protein CDS	hypothetical protein	None	KLI700 43.1
Variants: VaAphrodite_A_FDSW210055057-2r	G	944	944	1	C -> G	979	SNP (transversion)	29.90%	0		hypothetical protein CDS	hypothetical protein	None	KLI700 43.1
Variants: VaAphrodite_A_FDSW210055057-2r	G	947	947	1	C -> G	984	SNP (transversion)	52.60%	0		hypothetical protein CDS	hypothetical protein	None	KLI700 43.1
Variants: VaAphrodite_A_FDSW210055057-2r	AT	947	948	2	CG -> AT	984 -> 993	Substitution	29.4% -> 29.7%	4.30E-303	T -> N	hypothetical protein CDS	hypothetical protein	Substituti on	KLI700 43.1
Variants: VaAphrodite_A_FDSW210055057-2r	A	950	950	1	G -> A	1000	SNP (transition)	52.80%	0		hypothetical protein CDS	hypothetical protein	None	KLI700 43.1
Variants: VaAphrodite_A_FDSW210055057-2r	CT T	955	957	3	TGG -> CTT	997 -> 999	Substitution	52.10%	0	TN -> KD	hypothetical protein CDS	hypothetical protein	Substituti on	KLI700 43.1
Variants: VaAphrodite_A_FDSW210055057-2r	T	959	959	1	C -> T	997	SNP (transition)	51.80%	0		hypothetical protein CDS	hypothetical protein	None	KLI700 43.1
Variants: VaAphrodite_A_FDSW210055057-2r	G	971	971	1	A -> G	1011	SNP (transition)	37.70%	0		hypothetical protein CDS	hypothetical protein	None	KLI700 43.1
Variants: VaAphrodite_A_FDSW210055057-2r	G	974	974	1	A -> G	1013	SNP (transition)	25.30%	7.7E-319		hypothetical protein CDS	hypothetical protein	None	KLI700 43.1
Variants: VaAphrodite_A_FDSW210055057-2r	C	977	977	1	G -> C	1027	SNP (transversion)	48.60%	0		hypothetical protein CDS	hypothetical protein	None	KLI700 43.1
Variants: VaAphrodite_A_FDSW210055057-2r	C	983	983	1	T -> C	1055	SNP (transition)	68.40%	0		hypothetical protein CDS	hypothetical protein	None	KLI700 43.1
Variants: VaAphrodite_A_FDSW210055057-2r	C	986	986	1	G -> C	1064	SNP (transversion)	68.80%	0		hypothetical protein CDS	hypothetical protein	None	KLI700 43.1
Variants: VaAphrodite_A_FDSW210055057-2r	T	1013	1013	1	C -> T	1137	SNP (transition)	31.90%	0		hypothetical protein CDS	hypothetical protein	None	KLI700 43.1
Variants: VaAphrodite_A_FDSW210055057-2r	G	1025	1025	1	C -> G	1191	SNP (transversion)	43.00%	0		hypothetical protein CDS	hypothetical protein	None	KLI700 43.1
Variants: VaAphrodite_A_FDSW210055057-2r	T	1025	1025	1	C -> T	1191	SNP (transition)	34.20%	0		hypothetical protein CDS	hypothetical protein	None	KLI700 43.1
Variants: VaAphrodite_A_FDSW210055057-2r	G	1028	1028	1	C -> G	1192	SNP (transversion)	29.40%	0		hypothetical protein CDS	hypothetical protein	None	KLI700 43.1
Variants: VaAphrodite_A_FDSW210055057-2r	T	1054	1054	1	C -> T	1250	SNP (transition)	57.30%	0	A -> T	hypothetical protein CDS	hypothetical protein	Substituti on	KLI700 43.1
Variants: VaAphrodite_A_FDSW210055057-2r	A	1055	1055	1	C -> A	1251	SNP (transversion)	32.10%	0		hypothetical protein CDS	hypothetical protein	None	KLI700 43.1
Variants: VaAphrodite_A_FDSW210055057-2r	G	1055	1055	1	C -> G	1251	SNP (transversion)	39.20%	0		hypothetical protein CDS	hypothetical protein	None	KLI700 43.1
Variants: VaAphrodite_A_FDSW210055057-2r	A	1062	1062	1	T -> A	1256	SNP (transversion)	54.00%	0	Y -> F	hypothetical protein CDS	hypothetical protein	Substituti on	KLI700 43.1
Variants: VaAphrodite_B_FDSW210055058-2r	C	2	2	1	A -> C	638	SNP (transversion)	56.40%	0		hypothetical protein CDS	hypothetical protein	None	KLI700 43.1
Variants: VaAphrodite_B_FDSW210055058-2r	C	8	8	1	G -> C	621	SNP (transversion)	55.20%	0	D -> E	hypothetical protein CDS	hypothetical protein	Substituti on	KLI700 43.1
Variants: VaAphrodite_B_FDSW210055058-2r	A	47	47	1	G -> A	478	SNP (transition)	52.50%	0		hypothetical protein CDS	hypothetical protein	None	KLI700 43.1
Variants: VaAphrodite_B_FDSW210055058-2r	A	53	53	1	G -> A	459	SNP (transition)	56.40%	0		hypothetical protein CDS	hypothetical protein	None	KLI700 43.1
Variants: VaAphrodite_B_FDSW210055058-2r	A	56	56	1	G -> A	452	SNP (transition)	66.60%	0		hypothetical protein CDS	hypothetical protein	None	KLI700 43.1

Variants: VaAphrodite_B_FDSW210055058-2r	G	59	59	1	A -> G	441	SNP (transition)	70.10%	0		hypothetical protein CDS	hypothetical protein	None	KLI700 43.1
Variants: VaAphrodite_B_FDSW210055058-2r	G	62	62	1	A -> G	423	SNP (transition)	69.50%	0		hypothetical protein CDS	hypothetical protein	None	KLI700 43.1
Variants: VaAphrodite_B_FDSW210055058-2r	G	73	73	1	A -> G	380	SNP (transition)	67.10%	0		hypothetical protein CDS	hypothetical protein	None	KLI700 43.1
Variants: VaAphrodite_B_FDSW210055058-2r	G	101	101	1	A -> G	288	SNP (transition)	54.20%	7.10E-275		hypothetical protein CDS	hypothetical protein	None	KLI700 43.1
Variants: VaAphrodite_B_FDSW210055058-2r	C	109	109	1	T -> C	259	SNP (transition)	49.80%	2.10E-195	I -> V	hypothetical protein CDS	hypothetical protein	Substituti on	KLI700 43.1
Variants: VaAphrodite_B_FDSW210055058-2r	G	110	110	1	C -> G	255	SNP (transversion)	42.00%	1.70E-162		hypothetical protein CDS	hypothetical protein	None	KLI700 43.1
Variants: VaAphrodite_B_FDSW210055058-2r	AT	113	114	2	CG -> AT	244 -> 245	Substitution	38.4% -> 38.9%	2.80E-112	T -> N	hypothetical protein CDS	hypothetical protein	Substituti on	KLI700 43.1
Variants: VaAphrodite_B_FDSW210055058-2r	C	413	413	1	T -> C	229	SNP (transition)	44.10%	2.00E-186		hypothetical protein CDS	hypothetical protein	None	KLI700 43.1
Variants: VaAphrodite_B_FDSW210055058-2r	T	416	416	1	A -> T	248	SNP (transversion)	26.60%	1.20E-137		hypothetical protein CDS	hypothetical protein	None	KLI700 43.1
Variants: VaAphrodite_B_FDSW210055058-2r	C	421	421	1	T -> C	272	SNP (transition)	49.60%	9.7E-312	T -> A	hypothetical protein CDS	hypothetical protein	Substituti on	KLI700 43.1
Variants: VaAphrodite_B_FDSW210055058-2r	T	422	422	1	A -> T	282	SNP (transversion)	41.80%	1.60E-237		hypothetical protein CDS	hypothetical protein	None	KLI700 43.1
Variants: VaAphrodite_B_FDSW210055058-2r	T	425	425	1	C -> T	298	SNP (transition)	27.20%	4.00E-137		hypothetical protein CDS	hypothetical protein	None	KLI700 43.1
Variants: VaAphrodite_B_FDSW210055058-2r	G	428	428	1	A -> G	326	SNP (transition)	52.50%	0		hypothetical protein CDS	hypothetical protein	None	KLI700 43.1
Variants: VaAphrodite_B_FDSW210055058-2r	C	452	452	1	T -> C	475	SNP (transition)	32.00%	0		hypothetical protein CDS	hypothetical protein	None	KLI700 43.1
Variants: VaAphrodite_B_FDSW210055058-2r	A	455	455	1	G -> A	485	SNP (transition)	38.80%	0		hypothetical protein CDS	hypothetical protein	None	KLI700 43.1
Variants: VaAphrodite_B_FDSW210055058-2r	A	470	470	1	G -> A	583	SNP (transition)	27.40%	0		hypothetical protein CDS	hypothetical protein	None	KLI700 43.1
Variants: VaAphrodite_B_FDSW210055058-2r	G	473	473	1	C -> G	607	SNP (transversion)	52.40%	0		hypothetical protein CDS	hypothetical protein	None	KLI700 43.1
Variants: VaAphrodite_B_FDSW210055058-2r	G	479	479	1	C -> G	661	SNP (transversion)	64.10%	0		hypothetical protein CDS	hypothetical protein	None	KLI700 43.1
Variants: VaAphrodite_B_FDSW210055058-2r	TA G	482	484	2	CAA -> TAG	678 -> 699	Substitution	27.3% -> 28.2%	0		hypothetical protein CDS	hypothetical protein	None	KLI700 43.1
Variants: VaAphrodite_B_FDSW210055058-2r	GG T	485	487	2	CGC -> GGT	702 -> 718	Substitution	27.9% -> 28.5%	0	A -> T	hypothetical protein CDS	hypothetical protein	Substituti on	KLI700 43.1
Variants: VaAphrodite_B_FDSW210055058-2r	A	497	497	1	G -> A	804	SNP (transition)	29.00%	0		hypothetical protein CDS	hypothetical protein	None	KLI700 43.1
Variants: VaAphrodite_B_FDSW210055058-2r	C	509	509	1	G -> C	895	SNP (transversion)	52.00%	0		hypothetical protein CDS	hypothetical protein	None	KLI700 43.1
Variants: VaAphrodite_B_FDSW210055058-2r	G	530	530	1	C -> G	1083	SNP (transversion)	39.00%	0		hypothetical protein CDS	hypothetical protein	None	KLI700 43.1
Variants: VaAphrodite_B_FDSW210055058-2r	A	563	563	1	G -> A	1266	SNP (transition)	32.90%	0		hypothetical protein CDS	hypothetical protein	None	KLI700 43.1
Variants: VaAphrodite_B_FDSW210055058-2r	GG	593	594	2	CA -> GG	1288	Substitution	25.30%	0	V -> A	hypothetical protein CDS	hypothetical protein	Substituti on	KLI700 43.1
Variants: VaAphrodite_B_FDSW210055058-2r	A	608	608	1	G -> A	1276	SNP (transition)	39.70%	0		hypothetical protein CDS	hypothetical protein	None	KLI700 43.1
Variants: VaAphrodite_B_FDSW210055058-2r	G	614	614	1	A -> G	1271	SNP (transition)	33.00%	0		hypothetical protein CDS	hypothetical protein	None	KLI700 43.1
Variants: VaAphrodite_B_FDSW210055058-2r	C	628	628	1	T -> C	1232	SNP (transition)	38.40%	0	N -> D	hypothetical protein CDS	hypothetical protein	Substituti on	KLI700 43.1
Variants: VaAphrodite_B_FDSW210055058-2r	A	644	644	1	G -> A	1175	SNP (transition)	25.70%	0		hypothetical protein CDS	hypothetical protein	None	KLI700 43.1
Variants: VaAphrodite_B_FDSW210055058-2r	T	675	675	1	C -> T	1126	SNP (transition)	28.60%	0	S -> N	hypothetical protein CDS	hypothetical protein	Substituti on	KLI700 43.1
Variants: VaAphrodite_B_FDSW210055058-2r	A	677	677	1	G -> A	1101	SNP (transition)	46.10%	0		hypothetical protein CDS	hypothetical protein	None	KLI700 43.1
Variants: VaAphrodite_B_FDSW210055058-2r	A	722	722	1	G -> A	968	SNP (transition)	30.10%	0		hypothetical protein CDS	hypothetical protein	None	KLI700 43.1

Variants: VaAphrodite_B_FDSW210055058-2r	T	728	728	1	C -> T	935	SNP (transition)	25.30%	0		hypothetical protein CDS	hypothetical protein	None	KLI700 43.1
Variants: VaAphrodite_B_FDSW210055058-2r	A	737	737	1	G -> A	888	SNP (transition)	68.80%	0		hypothetical protein CDS	hypothetical protein	None	KLI700 43.1
Variants: VaAphrodite_B_FDSW210055058-2r	A	745	745	1	C -> A	883	SNP (transversion)	32.60%	0	A -> S	hypothetical protein CDS	hypothetical protein	Substituti on	KLI700 43.1
Variants: VaAphrodite_B_FDSW210055058-2r	C	752	752	1	G -> C	871	SNP (transversion)	32.70%	0		hypothetical protein CDS	hypothetical protein	None	KLI700 43.1
Variants: VaAphrodite_B_FDSW210055058-2r	A	755	755	1	C -> A	856	SNP (transversion)	33.40%	0		hypothetical protein CDS	hypothetical protein	None	KLI700 43.1
Variants: VaAphrodite_B_FDSW210055058-2r	A	767	767	1	G -> A	821	SNP (transition)	26.90%	0		hypothetical protein CDS	hypothetical protein	None	KLI700 43.1
Variants: VaAphrodite_B_FDSW210055058-2r	G	806	806	1	C -> G	689	SNP (transversion)	75.00%	0		hypothetical protein CDS	hypothetical protein	None	KLI700 43.1
Variants: VaAphrodite_B_FDSW210055058-2r	C	823	823	1	T -> C	653	SNP (transition)	63.70%	0	I -> V	hypothetical protein CDS	hypothetical protein	Substituti on	KLI700 43.1
Variants: VaAphrodite_B_FDSW210055058-2r	T	834	834	1	G -> T	634	SNP (transversion)	59.30%	0	T -> K	hypothetical protein CDS	hypothetical protein	Substituti on	KLI700 43.1
Variants: VaAphrodite_B_FDSW210055058-2r	C	844	844	1	T -> C	611	SNP (transition)	75.30%	0	T -> A	hypothetical protein CDS	hypothetical protein	Substituti on	KLI700 43.1
Variants: VaAphrodite_B_FDSW210055058-2r	A	854	854	1	G -> A	599	SNP (transition)	67.30%	0		hypothetical protein CDS	hypothetical protein	None	KLI700 43.1
Variants: VaAphrodite_B_FDSW210055058-2r	CA	865	866	2	GG -> CA	592 -> 593	Substitution	28.3% -> 28.7%	1.50E-275	IQ -> IE	hypothetical protein CDS	hypothetical protein	Substituti on	KLI700 43.1
Variants: VaAphrodite_B_FDSW210055058-2r	A	881	881	1	G -> A	621	SNP (transition)	50.60%	0		hypothetical protein CDS	hypothetical protein	None	KLI700 43.1
Variants: VaAphrodite_B_FDSW210055058-2r	A	887	887	1	G -> A	633	SNP (transition)	82.00%	0		hypothetical protein CDS	hypothetical protein	None	KLI700 43.1
Variants: VaAphrodite_B_FDSW210055058-2r	A	890	890	1	G -> A	637	SNP (transition)	26.80%	2.90E-185		hypothetical protein CDS	hypothetical protein	None	KLI700 43.1
Variants: VaAphrodite_B_FDSW210055058-2r	G	896	896	1	A -> G	625	SNP (transition)	82.20%	0		hypothetical protein CDS	hypothetical protein	None	KLI700 43.1
Variants: VaAphrodite_B_FDSW210055058-2r	G	908	908	1	A -> G	655	SNP (transition)	30.70%	1.80E-250		hypothetical protein CDS	hypothetical protein	None	KLI700 43.1
Variants: VaAphrodite_B_FDSW210055058-2r	GA C	941	943	2	AAT -> GAC	731 -> 738	Substitution	28.5% -> 29.0%	0	I -> V	hypothetical protein CDS	hypothetical protein	Substituti on	KLI700 43.1
Variants: VaAphrodite_B_FDSW210055058-2r	C	943	943	1	T -> C	731	SNP (transition)	55.30%	0	I -> V	hypothetical protein CDS	hypothetical protein	Substituti on	KLI700 43.1
Variants: VaAphrodite_B_FDSW210055058-2r	A	944	944	1	C -> A	734	SNP (transversion)	52.60%	0		hypothetical protein CDS	hypothetical protein	None	KLI700 43.1
Variants: VaAphrodite_B_FDSW210055058-2r	G	944	944	1	C -> G	734	SNP (transversion)	31.50%	5.80E-223		hypothetical protein CDS	hypothetical protein	None	KLI700 43.1
Variants: VaAphrodite_B_FDSW210055058-2r	G	947	947	1	C -> G	735	SNP (transversion)	52.20%	0		hypothetical protein CDS	hypothetical protein	None	KLI700 43.1
Variants: VaAphrodite_B_FDSW210055058-2r	AT	947	948	2	CG -> AT	735 -> 740	Substitution	31.6% -> 31.8%	2.90E-205	T -> N	hypothetical protein CDS	hypothetical protein	Substituti on	KLI700 43.1
Variants: VaAphrodite_B_FDSW210055058-2r	A	950	950	1	G -> A	754	SNP (transition)	52.40%	0		hypothetical protein CDS	hypothetical protein	None	KLI700 43.1
Variants: VaAphrodite_B_FDSW210055058-2r	CT T	955	957	3	TGG -> CTT	754 -> 756	Substitution	50.9% -> 51.7%	0	TN -> KD	hypothetical protein CDS	hypothetical protein	Substituti on	KLI700 43.1
Variants: VaAphrodite_B_FDSW210055058-2r	T	959	959	1	C -> T	762	SNP (transition)	51.00%	0		hypothetical protein CDS	hypothetical protein	None	KLI700 43.1
Variants: VaAphrodite_B_FDSW210055058-2r	G	971	971	1	A -> G	775	SNP (transition)	41.20%	0		hypothetical protein CDS	hypothetical protein	None	KLI700 43.1
Variants: VaAphrodite_B_FDSW210055058-2r	G	974	974	1	A -> G	765	SNP (transition)	25.10%	5.90E-183		hypothetical protein CDS	hypothetical protein	None	KLI700 43.1
Variants: VaAphrodite_B_FDSW210055058-2r	C	977	977	1	G -> C	764	SNP (transversion)	49.20%	0		hypothetical protein CDS	hypothetical protein	None	KLI700 43.1
Variants: VaAphrodite_B_FDSW210055058-2r	C	983	983	1	T -> C	772	SNP (transition)	69.00%	0		hypothetical protein CDS	hypothetical protein	None	KLI700 43.1
Variants: VaAphrodite_B_FDSW210055058-2r	C	986	986	1	G -> C	770	SNP (transversion)	69.00%	0		hypothetical protein CDS	hypothetical protein	None	KLI700 43.1
Variants: VaAphrodite_B_FDSW210055058-2r	T	1013	1013	1	C -> T	793	SNP (transition)	27.50%	4.60E-196		hypothetical protein CDS	hypothetical protein	None	KLI700 43.1

Variants: VaAphrodite_B_FDSW210055058-2r	G	1025	1025	1	C -> G	797	SNP (transversion)	48.20%	0		hypothetical protein CDS	hypothetical protein	None	KLI70043.1
Variants: VaAphrodite_B_FDSW210055058-2r	T	1025	1025	1	C -> T	797	SNP (transition)	29.40%	8.60E-218		hypothetical protein CDS	hypothetical protein	None	KLI70043.1
Variants: VaAphrodite_B_FDSW210055058-2r	G	1028	1028	1	C -> G	804	SNP (transversion)	31.30%	0		hypothetical protein CDS	hypothetical protein	None	KLI70043.1
Variants: VaAphrodite_B_FDSW210055058-2r	T	1054	1054	1	C -> T	862	SNP (transition)	58.70%	0	A -> T	hypothetical protein CDS	hypothetical protein	Substitution	KLI70043.1
Variants: VaAphrodite_B_FDSW210055058-2r	A	1055	1055	1	C -> A	868	SNP (transversion)	37.10%	0		hypothetical protein CDS	hypothetical protein	None	KLI70043.1
Variants: VaAphrodite_B_FDSW210055058-2r	G	1055	1055	1	C -> G	868	SNP (transversion)	37.30%	0		hypothetical protein CDS	hypothetical protein	None	KLI70043.1
Variants: VaAphrodite_B_FDSW210055058-2r	A	1062	1062	1	T -> A	871	SNP (transversion)	53.20%	0	Y -> F	hypothetical protein CDS	hypothetical protein	Substitution	KLI70043.1
Variants: VaAphrodite_C_FDSW210055059-1r	C	2	2	1	A -> C	892	SNP (transversion)	53.50%	0		hypothetical protein CDS	hypothetical protein	None	KLI70043.1
Variants: VaAphrodite_C_FDSW210055059-1r	C	8	8	1	G -> C	860	SNP (transversion)	54.10%	0	D -> E	hypothetical protein CDS	hypothetical protein	Substitution	KLI70043.1
Variants: VaAphrodite_C_FDSW210055059-1r	A	47	47	1	G -> A	701	SNP (transition)	52.20%	0		hypothetical protein CDS	hypothetical protein	None	KLI70043.1
Variants: VaAphrodite_C_FDSW210055059-1r	A	53	53	1	G -> A	671	SNP (transition)	58.00%	0		hypothetical protein CDS	hypothetical protein	None	KLI70043.1
Variants: VaAphrodite_C_FDSW210055059-1r	A	56	56	1	G -> A	657	SNP (transition)	65.40%	0		hypothetical protein CDS	hypothetical protein	None	KLI70043.1
Variants: VaAphrodite_C_FDSW210055059-1r	G	59	59	1	A -> G	643	SNP (transition)	71.50%	0		hypothetical protein CDS	hypothetical protein	None	KLI70043.1
Variants: VaAphrodite_C_FDSW210055059-1r	G	62	62	1	A -> G	629	SNP (transition)	70.60%	0		hypothetical protein CDS	hypothetical protein	None	KLI70043.1
Variants: VaAphrodite_C_FDSW210055059-1r	G	73	73	1	A -> G	573	SNP (transition)	67.50%	0		hypothetical protein CDS	hypothetical protein	None	KLI70043.1
Variants: VaAphrodite_C_FDSW210055059-1r	G	101	101	1	A -> G	444	SNP (transition)	58.80%	0		hypothetical protein CDS	hypothetical protein	None	KLI70043.1
Variants: VaAphrodite_C_FDSW210055059-1r	C	109	109	1	T -> C	421	SNP (transition)	57.20%	0	I -> V	hypothetical protein CDS	hypothetical protein	Substitution	KLI70043.1
Variants: VaAphrodite_C_FDSW210055059-1r	G	110	110	1	C -> G	415	SNP (transversion)	46.50%	5.0E-322		hypothetical protein CDS	hypothetical protein	None	KLI70043.1
Variants: VaAphrodite_C_FDSW210055059-1r	AT	113	114	2	CG -> AT	388 -> 396	Substitution	43.0% -> 43.9%	2.30E-244	T -> N	hypothetical protein CDS	hypothetical protein	Substitution	KLI70043.1
Variants: VaAphrodite_C_FDSW210055059-1r	T	392	392	1	G -> T	238	SNP (transversion)	41.20%	2.00E-167	N -> K	hypothetical protein CDS	hypothetical protein	Substitution	KLI70043.1
Variants: VaAphrodite_C_FDSW210055059-1r	A	395	395	1	G -> A	264	SNP (transition)	30.70%	2.60E-166		hypothetical protein CDS	hypothetical protein	None	KLI70043.1
Variants: VaAphrodite_C_FDSW210055059-1r	C	413	413	1	T -> C	419	SNP (transition)	66.30%	0		hypothetical protein CDS	hypothetical protein	None	KLI70043.1
Variants: VaAphrodite_C_FDSW210055059-1r	T	416	416	1	A -> T	439	SNP (transversion)	47.20%	0		hypothetical protein CDS	hypothetical protein	None	KLI70043.1
Variants: VaAphrodite_C_FDSW210055059-1r	CGC	419	421	2	GGT -> CGC	460 -> 475	Substitution	30.3% -> 31.3%	0	T -> A	hypothetical protein CDS	hypothetical protein	Substitution	KLI70043.1
Variants: VaAphrodite_C_FDSW210055059-1r	C	421	421	1	T -> C	475	SNP (transition)	33.10%	0	T -> A	hypothetical protein CDS	hypothetical protein	Substitution	KLI70043.1
Variants: VaAphrodite_C_FDSW210055059-1r	T	422	422	1	A -> T	487	SNP (transversion)	57.10%	0		hypothetical protein CDS	hypothetical protein	None	KLI70043.1
Variants: VaAphrodite_C_FDSW210055059-1r	T	425	425	1	C -> T	508	SNP (transition)	45.50%	0		hypothetical protein CDS	hypothetical protein	None	KLI70043.1
Variants: VaAphrodite_C_FDSW210055059-1r	G	428	428	1	A -> G	532	SNP (transition)	67.10%	0		hypothetical protein CDS	hypothetical protein	None	KLI70043.1
Variants: VaAphrodite_C_FDSW210055059-1r	C	452	452	1	T -> C	739	SNP (transition)	42.50%	0		hypothetical protein CDS	hypothetical protein	None	KLI70043.1
Variants: VaAphrodite_C_FDSW210055059-1r	A	455	455	1	G -> A	757	SNP (transition)	37.60%	0		hypothetical protein CDS	hypothetical protein	None	KLI70043.1
Variants: VaAphrodite_C_FDSW210055059-1r	A	470	470	1	G -> A	912	SNP (transition)	32.90%	0		hypothetical protein CDS	hypothetical protein	None	KLI70043.1
Variants: VaAphrodite_C_FDSW210055059-1r	G	473	473	1	C -> G	960	SNP (transversion)	51.50%	0		hypothetical protein CDS	hypothetical protein	None	KLI70043.1

Variants: VaAphrodite_C_FDSW210055059-1r	G	479	479	1	C -> G	1030	SNP (transversion)	62.20%		0		hypothetical protein CDS	hypothetical protein	None	KLI70043.1
Variants: VaAphrodite_C_FDSW210055059-1r	TAG	482	484	2	CAA -> TAG	1065 -> 1092	Substitution	29.7% -> 30.4%		0		hypothetical protein CDS	hypothetical protein	None	KLI70043.1
Variants: VaAphrodite_C_FDSW210055059-1r	GGT	485	487	2	CGC -> GGT	1106 -> 1129	Substitution	30.7% -> 30.8%		0	A -> T	hypothetical protein CDS	hypothetical protein	Substitution	KLI70043.1
Variants: VaAphrodite_C_FDSW210055059-1r	A	497	497	1	G -> A	1238	SNP (transition)	31.50%		0		hypothetical protein CDS	hypothetical protein	None	KLI70043.1
Variants: VaAphrodite_C_FDSW210055059-1r	C	509	509	1	G -> C	1372	SNP (transversion)	57.70%		0		hypothetical protein CDS	hypothetical protein	None	KLI70043.1
Variants: VaAphrodite_C_FDSW210055059-1r	G	530	530	1	C -> G	1564	SNP (transversion)	36.50%		0		hypothetical protein CDS	hypothetical protein	None	KLI70043.1
Variants: VaAphrodite_C_FDSW210055059-1r	A	563	563	1	G -> A	1705	SNP (transition)	36.20%		0		hypothetical protein CDS	hypothetical protein	None	KLI70043.1
Variants: VaAphrodite_C_FDSW210055059-1r	A	608	608	1	G -> A	1827	SNP (transition)	39.50%		0		hypothetical protein CDS	hypothetical protein	None	KLI70043.1
Variants: VaAphrodite_C_FDSW210055059-1r	G	614	614	1	A -> G	1824	SNP (transition)	30.00%		0		hypothetical protein CDS	hypothetical protein	None	KLI70043.1
Variants: VaAphrodite_C_FDSW210055059-1r	C	628	628	1	T -> C	1780	SNP (transition)	38.50%		0	N -> D	hypothetical protein CDS	hypothetical protein	Substitution	KLI70043.1
Variants: VaAphrodite_C_FDSW210055059-1r	A	644	644	1	G -> A	1730	SNP (transition)	29.00%		0		hypothetical protein CDS	hypothetical protein	None	KLI70043.1
Variants: VaAphrodite_C_FDSW210055059-1r	G	671	671	1	A -> G	1626	SNP (transition)	25.50%		0		hypothetical protein CDS	hypothetical protein	None	KLI70043.1
Variants: VaAphrodite_C_FDSW210055059-1r	T	675	675	1	C -> T	1610	SNP (transition)	32.40%		0	S -> N	hypothetical protein CDS	hypothetical protein	Substitution	KLI70043.1
Variants: VaAphrodite_C_FDSW210055059-1r	A	677	677	1	G -> A	1582	SNP (transition)	42.50%		0		hypothetical protein CDS	hypothetical protein	None	KLI70043.1
Variants: VaAphrodite_C_FDSW210055059-1r	A	722	722	1	G -> A	1381	SNP (transition)	29.70%		0		hypothetical protein CDS	hypothetical protein	None	KLI70043.1
Variants: VaAphrodite_C_FDSW210055059-1r	T	728	728	1	C -> T	1326	SNP (transition)	28.10%		0		hypothetical protein CDS	hypothetical protein	None	KLI70043.1
Variants: VaAphrodite_C_FDSW210055059-1r	A	737	737	1	G -> A	1232	SNP (transition)	70.40%		0		hypothetical protein CDS	hypothetical protein	None	KLI70043.1
Variants: VaAphrodite_C_FDSW210055059-1r	A	745	745	1	C -> A	1203	SNP (transversion)	28.80%		0	A -> S	hypothetical protein CDS	hypothetical protein	Substitution	KLI70043.1
Variants: VaAphrodite_C_FDSW210055059-1r	C	752	752	1	G -> C	1177	SNP (transversion)	30.20%		0		hypothetical protein CDS	hypothetical protein	None	KLI70043.1
Variants: VaAphrodite_C_FDSW210055059-1r	A	755	755	1	C -> A	1165	SNP (transversion)	33.40%		0		hypothetical protein CDS	hypothetical protein	None	KLI70043.1
Variants: VaAphrodite_C_FDSW210055059-1r	A	767	767	1	G -> A	1082	SNP (transition)	26.90%		0		hypothetical protein CDS	hypothetical protein	None	KLI70043.1
Variants: VaAphrodite_C_FDSW210055059-1r	G	806	806	1	C -> G	931	SNP (transversion)	75.30%		0		hypothetical protein CDS	hypothetical protein	None	KLI70043.1
Variants: VaAphrodite_C_FDSW210055059-1r	C	823	823	1	T -> C	941	SNP (transition)	58.40%		0	I -> V	hypothetical protein CDS	hypothetical protein	Substitution	KLI70043.1
Variants: VaAphrodite_C_FDSW210055059-1r	T	834	834	1	G -> T	950	SNP (transversion)	58.00%		0	T -> K	hypothetical protein CDS	hypothetical protein	Substitution	KLI70043.1
Variants: VaAphrodite_C_FDSW210055059-1r	C	844	844	1	T -> C	935	SNP (transition)	73.70%		0	T -> A	hypothetical protein CDS	hypothetical protein	Substitution	KLI70043.1
Variants: VaAphrodite_C_FDSW210055059-1r	A	854	854	1	G -> A	924	SNP (transition)	64.00%		0		hypothetical protein CDS	hypothetical protein	None	KLI70043.1
Variants: VaAphrodite_C_FDSW210055059-1r	CA	865	866	2	GG -> CA	922 -> 924	Substitution	29.3% -> 29.5%		0	IQ -> IE	hypothetical protein CDS	hypothetical protein	Substitution	KLI70043.1
Variants: VaAphrodite_C_FDSW210055059-1r	A	874	874	1	G -> A	918	SNP (transition)	25.30%		3.80E-289		hypothetical protein CDS	hypothetical protein	None	KLI70043.1
Variants: VaAphrodite_C_FDSW210055059-1r	A	881	881	1	G -> A	941	SNP (transition)	46.30%		0		hypothetical protein CDS	hypothetical protein	None	KLI70043.1
Variants: VaAphrodite_C_FDSW210055059-1r	A	887	887	1	G -> A	946	SNP (transition)	79.00%		0		hypothetical protein CDS	hypothetical protein	None	KLI70043.1
Variants: VaAphrodite_C_FDSW210055059-1r	G	896	896	1	A -> G	931	SNP (transition)	79.40%		0		hypothetical protein CDS	hypothetical protein	None	KLI70043.1
Variants: VaAphrodite_C_FDSW210055059-1r	G	908	908	1	A -> G	946	SNP (transition)	27.90%	9.0E-316			hypothetical protein CDS	hypothetical protein	None	KLI70043.1

Variants: VaAphrodite_C_FDSW210055059-1r	GA C	941	943	2	AAT -> GAC	984 -> 996	Substitution	29.9% -> 30.2%		0	I -> V	hypothetical protein CDS	hypothetical protein	Substituti on	KLI700 43.1
Variants: VaAphrodite_C_FDSW210055059-1r	C	943	943	1	T -> C	984	SNP (transition)	53.20%		0	I -> V	hypothetical protein CDS	hypothetical protein	Substituti on	KLI700 43.1
Variants: VaAphrodite_C_FDSW210055059-1r	A	944	944	1	C -> A	986	SNP (transversion)	54.90%		0		hypothetical protein CDS	hypothetical protein	None	KLI700 43.1
Variants: VaAphrodite_C_FDSW210055059-1r	G	944	944	1	C -> G	986	SNP (transversion)	28.30%		2.90E-254		hypothetical protein CDS	hypothetical protein	None	KLI700 43.1
Variants: VaAphrodite_C_FDSW210055059-1r	G	947	947	1	C -> G	998	SNP (transversion)	54.20%		0		hypothetical protein CDS	hypothetical protein	None	KLI700 43.1
Variants: VaAphrodite_C_FDSW210055059-1r	AT	947	948	2	CG -> AT	998 -> 999	Substitution	28.5% -> 28.6%		1.50E-235	T -> N	hypothetical protein CDS	hypothetical protein	Substituti on	KLI700 43.1
Variants: VaAphrodite_C_FDSW210055059-1r	A	950	950	1	G -> A	1011	SNP (transition)	54.60%		0		hypothetical protein CDS	hypothetical protein	None	KLI700 43.1
Variants: VaAphrodite_C_FDSW210055059-1r	CT T	955	957	3	TGG -> CTT	1025 -> 1028	Substitution	53.4% -> 53.7%		0	TN -> KD	hypothetical protein CDS	hypothetical protein	Substituti on	KLI700 43.1
Variants: VaAphrodite_C_FDSW210055059-1r	T	959	959	1	C -> T	1035	SNP (transition)	53.10%		0		hypothetical protein CDS	hypothetical protein	None	KLI700 43.1
Variants: VaAphrodite_C_FDSW210055059-1r	G	971	971	1	A -> G	1034	SNP (transition)	40.80%		0		hypothetical protein CDS	hypothetical protein	None	KLI700 43.1
Variants: VaAphrodite_C_FDSW210055059-1r	C	977	977	1	G -> C	1049	SNP (transversion)	48.00%		0		hypothetical protein CDS	hypothetical protein	None	KLI700 43.1
Variants: VaAphrodite_C_FDSW210055059-1r	C	983	983	1	T -> C	1018	SNP (transition)	67.80%		0		hypothetical protein CDS	hypothetical protein	None	KLI700 43.1
Variants: VaAphrodite_C_FDSW210055059-1r	C	986	986	1	G -> C	1022	SNP (transversion)	66.90%		0		hypothetical protein CDS	hypothetical protein	None	KLI700 43.1
Variants: VaAphrodite_C_FDSW210055059-1r	G	1025	1025	1	C -> G	1078	SNP (transversion)	51.50%		0		hypothetical protein CDS	hypothetical protein	None	KLI700 43.1
Variants: VaAphrodite_C_FDSW210055059-1r	T	1025	1025	1	C -> T	1078	SNP (transition)	25.10%		8.30E-284		hypothetical protein CDS	hypothetical protein	None	KLI700 43.1
Variants: VaAphrodite_C_FDSW210055059-1r	G	1028	1028	1	C -> G	1078	SNP (transversion)	34.00%		0		hypothetical protein CDS	hypothetical protein	None	KLI700 43.1
Variants: VaAphrodite_C_FDSW210055059-1r	T	1054	1054	1	C -> T	1165	SNP (transition)	58.70%		0	A -> T	hypothetical protein CDS	hypothetical protein	Substituti on	KLI700 43.1
Variants: VaAphrodite_C_FDSW210055059-1r	A	1055	1055	1	C -> A	1164	SNP (transversion)	34.40%		0		hypothetical protein CDS	hypothetical protein	None	KLI700 43.1
Variants: VaAphrodite_C_FDSW210055059-1r	G	1055	1055	1	C -> G	1164	SNP (transversion)	36.30%		0		hypothetical protein CDS	hypothetical protein	None	KLI700 43.1
Variants: VaAphrodite_C_FDSW210055059-1r	A	1062	1062	1	T -> A	1173	SNP (transversion)	53.70%		0	Y -> F	hypothetical protein CDS	hypothetical protein	Substituti on	KLI700 43.1
Variants: VaAres1_A_FDSW210055060-2r	C	2	2	1	A -> C	559	SNP (transversion)	63.70%		0		hypothetical protein CDS	hypothetical protein	None	KLI700 43.1
Variants: VaAres1_A_FDSW210055060-2r	C	8	8	1	G -> C	545	SNP (transversion)	62.40%		0	D -> E	hypothetical protein CDS	hypothetical protein	Substituti on	KLI700 43.1
Variants: VaAres1_A_FDSW210055060-2r	A	47	47	1	G -> A	447	SNP (transition)	60.90%		0		hypothetical protein CDS	hypothetical protein	None	KLI700 43.1
Variants: VaAres1_A_FDSW210055060-2r	A	53	53	1	G -> A	426	SNP (transition)	63.40%		0		hypothetical protein CDS	hypothetical protein	None	KLI700 43.1
Variants: VaAres1_A_FDSW210055060-2r	A	56	56	1	G -> A	416	SNP (transition)	74.00%		0		hypothetical protein CDS	hypothetical protein	None	KLI700 43.1
Variants: VaAres1_A_FDSW210055060-2r	G	59	59	1	A -> G	403	SNP (transition)	78.20%		0		hypothetical protein CDS	hypothetical protein	None	KLI700 43.1
Variants: VaAres1_A_FDSW210055060-2r	G	62	62	1	A -> G	397	SNP (transition)	77.80%		0		hypothetical protein CDS	hypothetical protein	None	KLI700 43.1
Variants: VaAres1_A_FDSW210055060-2r	G	73	73	1	A -> G	361	SNP (transition)	75.90%		0		hypothetical protein CDS	hypothetical protein	None	KLI700 43.1
Variants: VaAres1_A_FDSW210055060-2r	G	101	101	1	A -> G	269	SNP (transition)	68.40%	8.0E-316			hypothetical protein CDS	hypothetical protein	None	KLI700 43.1
Variants: VaAres1_A_FDSW210055060-2r	CG	109	110	2	TC -> CG	231 -> 233	Substitution	55.0% -> 56.7%		2.80E-162	AI -> AV	hypothetical protein CDS	hypothetical protein	Substituti on	KLI700 43.1
Variants: VaAres1_A_FDSW210055060-2r	AT	113	114	2	CG -> AT	213 -> 218	Substitution	50.2% -> 51.4%		7.90E-112	T -> N	hypothetical protein CDS	hypothetical protein	Substituti on	KLI700 43.1
Variants: VaAres1_A_FDSW210055060-2r	C	386	386	1	T -> C	238	SNP (transition)	48.70%		5.20E-209		hypothetical protein CDS	hypothetical protein	None	KLI700 43.1

Variants: VaAres1_A_FDSW210055060-2r	T	392	392	1	G -> T	257	SNP (transversion)	51.00%		2.30E-252	N -> K	hypothetical protein CDS	hypothetical protein	Substitution	KLI70043.1
Variants: VaAres1_A_FDSW210055060-2r	A	395	395	1	G -> A	268	SNP (transition)	40.70%		4.10E-196		hypothetical protein CDS	hypothetical protein	None	KLI70043.1
Variants: VaAres1_A_FDSW210055060-2r	C	413	413	1	T -> C	339	SNP (transition)	66.70%		0		hypothetical protein CDS	hypothetical protein	None	KLI70043.1
Variants: VaAres1_A_FDSW210055060-2r	T	416	416	1	A -> T	361	SNP (transversion)	48.50%		0		hypothetical protein CDS	hypothetical protein	None	KLI70043.1
Variants: VaAres1_A_FDSW210055060-2r	CG C	419	421	2	GGT -> CGC	371 -> 384	Substitution	30.7% -> 31.8%		1.10E-267	T -> A	hypothetical protein CDS	hypothetical protein	Substitution	KLI70043.1
Variants: VaAres1_A_FDSW210055060-2r	C	421	421	1	T -> C	384	SNP (transition)	37.80%		0	T -> A	hypothetical protein CDS	hypothetical protein	Substitution	KLI70043.1
Variants: VaAres1_A_FDSW210055060-2r	T	422	422	1	A -> T	395	SNP (transversion)	58.00%		0		hypothetical protein CDS	hypothetical protein	None	KLI70043.1
Variants: VaAres1_A_FDSW210055060-2r	T	425	425	1	C -> T	410	SNP (transition)	35.60%	4.9E-324			hypothetical protein CDS	hypothetical protein	None	KLI70043.1
Variants: VaAres1_A_FDSW210055060-2r	G	428	428	1	A -> G	426	SNP (transition)	65.70%		0		hypothetical protein CDS	hypothetical protein	None	KLI70043.1
Variants: VaAres1_A_FDSW210055060-2r	C	452	452	1	T -> C	533	SNP (transition)	34.50%		0		hypothetical protein CDS	hypothetical protein	None	KLI70043.1
Variants: VaAres1_A_FDSW210055060-2r	A	455	455	1	G -> A	555	SNP (transition)	43.10%		0		hypothetical protein CDS	hypothetical protein	None	KLI70043.1
Variants: VaAres1_A_FDSW210055060-2r	G	464	464	1	A -> G	589	SNP (transition)	30.90%		0		hypothetical protein CDS	hypothetical protein	None	KLI70043.1
Variants: VaAres1_A_FDSW210055060-2r	A	470	470	1	G -> A	602	SNP (transition)	29.10%		0		hypothetical protein CDS	hypothetical protein	None	KLI70043.1
Variants: VaAres1_A_FDSW210055060-2r	G	473	473	1	C -> G	616	SNP (transversion)	53.10%		0		hypothetical protein CDS	hypothetical protein	None	KLI70043.1
Variants: VaAres1_A_FDSW210055060-2r	G	479	479	1	C -> G	656	SNP (transversion)	58.80%		0		hypothetical protein CDS	hypothetical protein	None	KLI70043.1
Variants: VaAres1_A_FDSW210055060-2r	G	484	484	1	A -> G	688	SNP (transition)	25.30%		0		hypothetical protein CDS	hypothetical protein	None	KLI70043.1
Variants: VaAres1_A_FDSW210055060-2r	A	497	497	1	G -> A	754	SNP (transition)	25.70%		0		hypothetical protein CDS	hypothetical protein	None	KLI70043.1
Variants: VaAres1_A_FDSW210055060-2r	C	509	509	1	G -> C	805	SNP (transversion)	48.00%		0		hypothetical protein CDS	hypothetical protein	None	KLI70043.1
Variants: VaAres1_A_FDSW210055060-2r	G	530	530	1	C -> G	962	SNP (transversion)	36.60%		0		hypothetical protein CDS	hypothetical protein	None	KLI70043.1
Variants: VaAres1_A_FDSW210055060-2r	A	563	563	1	G -> A	1081	SNP (transition)	32.80%		0		hypothetical protein CDS	hypothetical protein	None	KLI70043.1
Variants: VaAres1_A_FDSW210055060-2r	A	608	608	1	G -> A	1207	SNP (transition)	44.40%		0		hypothetical protein CDS	hypothetical protein	None	KLI70043.1
Variants: VaAres1_A_FDSW210055060-2r	G	614	614	1	A -> G	1240	SNP (transition)	35.60%		0		hypothetical protein CDS	hypothetical protein	None	KLI70043.1
Variants: VaAres1_A_FDSW210055060-2r	T	623	623	1	C -> T	1267	SNP (transition)	28.40%		0		hypothetical protein CDS	hypothetical protein	None	KLI70043.1
Variants: VaAres1_A_FDSW210055060-2r	C	628	628	1	T -> C	1276	SNP (transition)	44.70%		0	N -> D	hypothetical protein CDS	hypothetical protein	Substitution	KLI70043.1
Variants: VaAres1_A_FDSW210055060-2r	A	644	644	1	G -> A	1245	SNP (transition)	27.40%		0		hypothetical protein CDS	hypothetical protein	None	KLI70043.1
Variants: VaAres1_A_FDSW210055060-2r	G	671	671	1	A -> G	1174	SNP (transition)	32.10%		0		hypothetical protein CDS	hypothetical protein	None	KLI70043.1
Variants: VaAres1_A_FDSW210055060-2r	T	675	675	1	C -> T	1149	SNP (transition)	30.90%		0	S -> N	hypothetical protein CDS	hypothetical protein	Substitution	KLI70043.1
Variants: VaAres1_A_FDSW210055060-2r	A	677	677	1	G -> A	1110	SNP (transition)	37.30%		0		hypothetical protein CDS	hypothetical protein	None	KLI70043.1
Variants: VaAres1_A_FDSW210055060-2r	A	722	722	1	G -> A	963	SNP (transition)	27.50%		0		hypothetical protein CDS	hypothetical protein	None	KLI70043.1
Variants: VaAres1_A_FDSW210055060-2r	T	728	728	1	C -> T	935	SNP (transition)	29.70%		0		hypothetical protein CDS	hypothetical protein	None	KLI70043.1
Variants: VaAres1_A_FDSW210055060-2r	A	737	737	1	G -> A	874	SNP (transition)	72.40%		0		hypothetical protein CDS	hypothetical protein	None	KLI70043.1
Variants: VaAres1_A_FDSW210055060-2r	A	745	745	1	C -> A	843	SNP (transversion)	27.30%		0	A -> S	hypothetical protein CDS	hypothetical protein	Substitution	KLI70043.1

Variants: VaAres1_A_FDSW210055060-2r	C	752	752	1	G -> C	808	SNP (transversion)	27.80%	0		hypothetical protein CDS	hypothetical protein	None	KLI700 43.1
Variants: VaAres1_A_FDSW210055060-2r	A	755	755	1	C -> A	804	SNP (transversion)	33.60%	0		hypothetical protein CDS	hypothetical protein	None	KLI700 43.1
Variants: VaAres1_A_FDSW210055060-2r	A	767	767	1	G -> A	727	SNP (transition)	28.20%	0		hypothetical protein CDS	hypothetical protein	None	KLI700 43.1
Variants: VaAres1_A_FDSW210055060-2r	G	806	806	1	C -> G	646	SNP (transversion)	74.10%	0		hypothetical protein CDS	hypothetical protein	None	KLI700 43.1
Variants: VaAres1_A_FDSW210055060-2r	C	823	823	1	T -> C	639	SNP (transition)	60.30%	0	I -> V	hypothetical protein CDS	hypothetical protein	Substituti on	KLI700 43.1
Variants: VaAres1_A_FDSW210055060-2r	T	834	834	1	G -> T	604	SNP (transversion)	55.80%	0	T -> K	hypothetical protein CDS	hypothetical protein	Substituti on	KLI700 43.1
Variants: VaAres1_A_FDSW210055060-2r	C	844	844	1	T -> C	576	SNP (transition)	74.50%	0	T -> A	hypothetical protein CDS	hypothetical protein	Substituti on	KLI700 43.1
Variants: VaAres1_A_FDSW210055060-2r	A	854	854	1	G -> A	590	SNP (transition)	65.90%	0		hypothetical protein CDS	hypothetical protein	None	KLI700 43.1
Variants: VaAres1_A_FDSW210055060-2r	CA	865	866	2	GG -> CA	599	Substitution	30.90%	6.8E-311	IQ -> IE	hypothetical protein CDS	hypothetical protein	Substituti on	KLI700 43.1
Variants: VaAres1_A_FDSW210055060-2r	A	874	874	1	G -> A	610	SNP (transition)	27.40%	3.10E-248		hypothetical protein CDS	hypothetical protein	None	KLI700 43.1
Variants: VaAres1_A_FDSW210055060-2r	A	881	881	1	G -> A	605	SNP (transition)	43.10%	0		hypothetical protein CDS	hypothetical protein	None	KLI700 43.1
Variants: VaAres1_A_FDSW210055060-2r	A	887	887	1	G -> A	625	SNP (transition)	80.20%	0		hypothetical protein CDS	hypothetical protein	None	KLI700 43.1
Variants: VaAres1_A_FDSW210055060-2r	A	890	890	1	G -> A	616	SNP (transition)	27.80%	1.40E-221		hypothetical protein CDS	hypothetical protein	None	KLI700 43.1
Variants: VaAres1_A_FDSW210055060-2r	G	896	896	1	A -> G	623	SNP (transition)	80.10%	0		hypothetical protein CDS	hypothetical protein	None	KLI700 43.1
Variants: VaAres1_A_FDSW210055060-2r	G	908	908	1	A -> G	665	SNP (transition)	32.50%	1.50E-296		hypothetical protein CDS	hypothetical protein	None	KLI700 43.1
Variants: VaAres1_A_FDSW210055060-2r	GA C	941	943	2	AAT -> GAC	733 -> 736	Substitution	28.5% -> 28.8%	0	I -> V	hypothetical protein CDS	hypothetical protein	Substituti on	KLI700 43.1
Variants: VaAres1_A_FDSW210055060-2r	C	943	943	1	T -> C	736	SNP (transition)	56.40%	0	I -> V	hypothetical protein CDS	hypothetical protein	Substituti on	KLI700 43.1
Variants: VaAres1_A_FDSW210055060-2r	A	944	944	1	C -> A	735	SNP (transversion)	57.60%	0		hypothetical protein CDS	hypothetical protein	None	KLI700 43.1
Variants: VaAres1_A_FDSW210055060-2r	G	944	944	1	C -> G	735	SNP (transversion)	27.80%	5.50E-224		hypothetical protein CDS	hypothetical protein	None	KLI700 43.1
Variants: VaAres1_A_FDSW210055060-2r	G	947	947	1	C -> G	725	SNP (transversion)	57.10%	0		hypothetical protein CDS	hypothetical protein	None	KLI700 43.1
Variants: VaAres1_A_FDSW210055060-2r	AT	947	948	2	CG -> AT	725 -> 726	Substitution	28.0% -> 28.5%	6.90E-210	T -> N	hypothetical protein CDS	hypothetical protein	Substituti on	KLI700 43.1
Variants: VaAres1_A_FDSW210055060-2r	A	950	950	1	G -> A	730	SNP (transition)	56.80%	0		hypothetical protein CDS	hypothetical protein	None	KLI700 43.1
Variants: VaAres1_A_FDSW210055060-2r	CT T	955	957	3	TGG -> CTT	724 -> 728	Substitution	54.9% -> 55.5%	0	TN -> KD	hypothetical protein CDS	hypothetical protein	Substituti on	KLI700 43.1
Variants: VaAres1_A_FDSW210055060-2r	T	959	959	1	C -> T	725	SNP (transition)	54.80%	0		hypothetical protein CDS	hypothetical protein	None	KLI700 43.1
Variants: VaAres1_A_FDSW210055060-2r	G	971	971	1	A -> G	730	SNP (transition)	40.50%	0		hypothetical protein CDS	hypothetical protein	None	KLI700 43.1
Variants: VaAres1_A_FDSW210055060-2r	C	977	977	1	G -> C	731	SNP (transversion)	49.00%	0		hypothetical protein CDS	hypothetical protein	None	KLI700 43.1
Variants: VaAres1_A_FDSW210055060-2r	C	983	983	1	T -> C	722	SNP (transition)	68.80%	0		hypothetical protein CDS	hypothetical protein	None	KLI700 43.1
Variants: VaAres1_A_FDSW210055060-2r	C	986	986	1	G -> C	722	SNP (transversion)	68.80%	0		hypothetical protein CDS	hypothetical protein	None	KLI700 43.1
Variants: VaAres1_A_FDSW210055060-2r	T	1013	1013	1	C -> T	735	SNP (transition)	28.30%	1.20E-250		hypothetical protein CDS	hypothetical protein	None	KLI700 43.1
Variants: VaAres1_A_FDSW210055060-2r	G	1025	1025	1	C -> G	745	SNP (transversion)	44.80%	0		hypothetical protein CDS	hypothetical protein	None	KLI700 43.1
Variants: VaAres1_A_FDSW210055060-2r	T	1025	1025	1	C -> T	745	SNP (transition)	30.60%	1.60E-283		hypothetical protein CDS	hypothetical protein	None	KLI700 43.1
Variants: VaAres1_A_FDSW210055060-2r	G	1028	1028	1	C -> G	753	SNP (transversion)	27.60%	3.70E-248		hypothetical protein CDS	hypothetical protein	None	KLI700 43.1

Variants: VaAres1_A_FDSW210055060-2r	G	1031	1031	1	A -> G	763	SNP (transition)	26.30%		0		hypothetical protein CDS	hypothetical protein	None	KLI70043.1
Variants: VaAres1_A_FDSW210055060-2r	T	1054	1054	1	C -> T	798	SNP (transition)	64.20%		0	A -> T	hypothetical protein CDS	hypothetical protein	Substitution	KLI70043.1
Variants: VaAres1_A_FDSW210055060-2r	A	1055	1055	1	C -> A	797	SNP (transversion)	36.00%		0		hypothetical protein CDS	hypothetical protein	None	KLI70043.1
Variants: VaAres1_A_FDSW210055060-2r	G	1055	1055	1	C -> G	797	SNP (transversion)	40.90%		0		hypothetical protein CDS	hypothetical protein	None	KLI70043.1
Variants: VaAres1_A_FDSW210055060-2r	A	1062	1062	1	T -> A	814	SNP (transversion)	57.10%		0	Y -> F	hypothetical protein CDS	hypothetical protein	Substitution	KLI70043.1
Variants: VaAres1_B_FDSW210055061-2r	C	2	2	1	A -> C	681	SNP (transversion)	60.50%		0		hypothetical protein CDS	hypothetical protein	None	KLI70043.1
Variants: VaAres1_B_FDSW210055061-2r	C	8	8	1	G -> C	670	SNP (transversion)	58.80%		0	D -> E	hypothetical protein CDS	hypothetical protein	Substitution	KLI70043.1
Variants: VaAres1_B_FDSW210055061-2r	A	47	47	1	G -> A	542	SNP (transition)	58.10%		0		hypothetical protein CDS	hypothetical protein	None	KLI70043.1
Variants: VaAres1_B_FDSW210055061-2r	A	53	53	1	G -> A	521	SNP (transition)	61.60%		0		hypothetical protein CDS	hypothetical protein	None	KLI70043.1
Variants: VaAres1_B_FDSW210055061-2r	A	56	56	1	G -> A	505	SNP (transition)	71.10%		0		hypothetical protein CDS	hypothetical protein	None	KLI70043.1
Variants: VaAres1_B_FDSW210055061-2r	G	59	59	1	A -> G	497	SNP (transition)	74.20%		0		hypothetical protein CDS	hypothetical protein	None	KLI70043.1
Variants: VaAres1_B_FDSW210055061-2r	G	62	62	1	A -> G	488	SNP (transition)	73.60%		0		hypothetical protein CDS	hypothetical protein	None	KLI70043.1
Variants: VaAres1_B_FDSW210055061-2r	G	73	73	1	A -> G	452	SNP (transition)	70.60%		0		hypothetical protein CDS	hypothetical protein	None	KLI70043.1
Variants: VaAres1_B_FDSW210055061-2r	G	101	101	1	A -> G	317	SNP (transition)	59.00%		0		hypothetical protein CDS	hypothetical protein	None	KLI70043.1
Variants: VaAres1_B_FDSW210055061-2r	CG	109	110	2	TC -> CG	289 -> 294	Substitution	47.8% -> 48.6%		1.70E-205	AI -> AV	hypothetical protein CDS	hypothetical protein	Substitution	KLI70043.1
Variants: VaAres1_B_FDSW210055061-2r	AT	113	114	2	CG -> AT	259 -> 265	Substitution	42.5% -> 43.8%		2.30E-124	T -> N	hypothetical protein CDS	hypothetical protein	Substitution	KLI70043.1
Variants: VaAres1_B_FDSW210055061-2r	A	374	374	1	G -> A	306	SNP (transition)	43.10%	1.8E-320			hypothetical protein CDS	hypothetical protein	None	KLI70043.1
Variants: VaAres1_B_FDSW210055061-2r	C	386	386	1	T -> C	408	SNP (transition)	67.20%		0		hypothetical protein CDS	hypothetical protein	None	KLI70043.1
Variants: VaAres1_B_FDSW210055061-2r	T	392	392	1	G -> T	445	SNP (transversion)	67.20%		0	N -> K	hypothetical protein CDS	hypothetical protein	Substitution	KLI70043.1
Variants: VaAres1_B_FDSW210055061-2r	A	395	395	1	G -> A	463	SNP (transition)	54.20%		0		hypothetical protein CDS	hypothetical protein	None	KLI70043.1
Variants: VaAres1_B_FDSW210055061-2r	A	407	407	1	G -> A	521	SNP (transition)	29.60%		0		hypothetical protein CDS	hypothetical protein	None	KLI70043.1
Variants: VaAres1_B_FDSW210055061-2r	C	413	413	1	T -> C	562	SNP (transition)	77.20%		0		hypothetical protein CDS	hypothetical protein	None	KLI70043.1
Variants: VaAres1_B_FDSW210055061-2r	T	416	416	1	A -> T	582	SNP (transversion)	58.60%		0		hypothetical protein CDS	hypothetical protein	None	KLI70043.1
Variants: VaAres1_B_FDSW210055061-2r	CGC	419	421	2	GGT -> CGC	608 -> 619	Substitution	38.9% -> 39.6%		0	T -> A	hypothetical protein CDS	hypothetical protein	Substitution	KLI70043.1
Variants: VaAres1_B_FDSW210055061-2r	C	421	421	1	T -> C	619	SNP (transition)	35.50%		0	T -> A	hypothetical protein CDS	hypothetical protein	Substitution	KLI70043.1
Variants: VaAres1_B_FDSW210055061-2r	T	422	422	1	A -> T	624	SNP (transversion)	70.40%		0		hypothetical protein CDS	hypothetical protein	None	KLI70043.1
Variants: VaAres1_B_FDSW210055061-2r	T	425	425	1	C -> T	667	SNP (transition)	51.00%		0		hypothetical protein CDS	hypothetical protein	None	KLI70043.1
Variants: VaAres1_B_FDSW210055061-2r	G	428	428	1	A -> G	676	SNP (transition)	76.30%		0		hypothetical protein CDS	hypothetical protein	None	KLI70043.1
Variants: VaAres1_B_FDSW210055061-2r	C	452	452	1	T -> C	814	SNP (transition)	43.00%		0		hypothetical protein CDS	hypothetical protein	None	KLI70043.1
Variants: VaAres1_B_FDSW210055061-2r	A	455	455	1	G -> A	826	SNP (transition)	41.50%		0		hypothetical protein CDS	hypothetical protein	None	KLI70043.1
Variants: VaAres1_B_FDSW210055061-2r	A	470	470	1	G -> A	931	SNP (transition)	40.40%		0		hypothetical protein CDS	hypothetical protein	None	KLI70043.1
Variants: VaAres1_B_FDSW210055061-2r	G	473	473	1	C -> G	943	SNP (transversion)	47.00%		0		hypothetical protein CDS	hypothetical protein	None	KLI70043.1

Variants: VaAres1_B_FDSW210055061-2r	G	479	479	1	C -> G	965	SNP (transversion)	58.00%	0		hypothetical protein CDS	hypothetical protein	None	KLI70043.1
Variants: VaAres1_B_FDSW210055061-2r	G	484	484	1	A -> G	1027	SNP (transition)	29.30%	0		hypothetical protein CDS	hypothetical protein	None	KLI70043.1
Variants: VaAres1_B_FDSW210055061-2r	A	497	497	1	G -> A	1111	SNP (transition)	25.00%	0		hypothetical protein CDS	hypothetical protein	None	KLI70043.1
Variants: VaAres1_B_FDSW210055061-2r	C	509	509	1	G -> C	1172	SNP (transversion)	54.40%	0		hypothetical protein CDS	hypothetical protein	None	KLI70043.1
Variants: VaAres1_B_FDSW210055061-2r	G	530	530	1	C -> G	1324	SNP (transversion)	37.80%	0		hypothetical protein CDS	hypothetical protein	None	KLI70043.1
Variants: VaAres1_B_FDSW210055061-2r	A	563	563	1	G -> A	1327	SNP (transition)	34.10%	0		hypothetical protein CDS	hypothetical protein	None	KLI70043.1
Variants: VaAres1_B_FDSW210055061-2r	A	608	608	1	G -> A	1394	SNP (transition)	43.70%	0		hypothetical protein CDS	hypothetical protein	None	KLI70043.1
Variants: VaAres1_B_FDSW210055061-2r	G	614	614	1	A -> G	1394	SNP (transition)	34.10%	0		hypothetical protein CDS	hypothetical protein	None	KLI70043.1
Variants: VaAres1_B_FDSW210055061-2r	T	623	623	1	C -> T	1429	SNP (transition)	28.30%	0		hypothetical protein CDS	hypothetical protein	None	KLI70043.1
Variants: VaAres1_B_FDSW210055061-2r	C	628	628	1	T -> C	1432	SNP (transition)	44.30%	0	N -> D	hypothetical protein CDS	hypothetical protein	Substitution	KLI70043.1
Variants: VaAres1_B_FDSW210055061-2r	A	644	644	1	G -> A	1428	SNP (transition)	26.50%	0		hypothetical protein CDS	hypothetical protein	None	KLI70043.1
Variants: VaAres1_B_FDSW210055061-2r	G	671	671	1	A -> G	1440	SNP (transition)	35.70%	0		hypothetical protein CDS	hypothetical protein	None	KLI70043.1
Variants: VaAres1_B_FDSW210055061-2r	T	675	675	1	C -> T	1407	SNP (transition)	28.90%	0	S -> N	hypothetical protein CDS	hypothetical protein	Substitution	KLI70043.1
Variants: VaAres1_B_FDSW210055061-2r	A	677	677	1	G -> A	1270	SNP (transition)	37.20%	0		hypothetical protein CDS	hypothetical protein	None	KLI70043.1
Variants: VaAres1_B_FDSW210055061-2r	A	722	722	1	G -> A	1147	SNP (transition)	27.50%	0		hypothetical protein CDS	hypothetical protein	None	KLI70043.1
Variants: VaAres1_B_FDSW210055061-2r	T	728	728	1	C -> T	1107	SNP (transition)	29.10%	0		hypothetical protein CDS	hypothetical protein	None	KLI70043.1
Variants: VaAres1_B_FDSW210055061-2r	A	737	737	1	G -> A	1041	SNP (transition)	72.20%	0		hypothetical protein CDS	hypothetical protein	None	KLI70043.1
Variants: VaAres1_B_FDSW210055061-2r	A	755	755	1	C -> A	972	SNP (transversion)	40.30%	0		hypothetical protein CDS	hypothetical protein	None	KLI70043.1
Variants: VaAres1_B_FDSW210055061-2r	A	767	767	1	G -> A	883	SNP (transition)	35.00%	0		hypothetical protein CDS	hypothetical protein	None	KLI70043.1
Variants: VaAres1_B_FDSW210055061-2r	G	776	776	1	A -> G	871	SNP (transition)	30.00%	0		hypothetical protein CDS	hypothetical protein	None	KLI70043.1
Variants: VaAres1_B_FDSW210055061-2r	A	779	779	1	G -> A	867	SNP (transition)	29.60%	0		hypothetical protein CDS	hypothetical protein	None	KLI70043.1
Variants: VaAres1_B_FDSW210055061-2r	G	806	806	1	C -> G	713	SNP (transversion)	72.40%	0		hypothetical protein CDS	hypothetical protein	None	KLI70043.1
Variants: VaAres1_B_FDSW210055061-2r	C	823	823	1	T -> C	643	SNP (transition)	59.60%	0	I -> V	hypothetical protein CDS	hypothetical protein	Substitution	KLI70043.1
Variants: VaAres1_B_FDSW210055061-2r	T	834	834	1	G -> T	629	SNP (transversion)	58.00%	0	T -> K	hypothetical protein CDS	hypothetical protein	Substitution	KLI70043.1
Variants: VaAres1_B_FDSW210055061-2r	C	844	844	1	T -> C	603	SNP (transition)	74.00%	0	T -> A	hypothetical protein CDS	hypothetical protein	Substitution	KLI70043.1
Variants: VaAres1_B_FDSW210055061-2r	A	854	854	1	G -> A	615	SNP (transition)	65.70%	0		hypothetical protein CDS	hypothetical protein	None	KLI70043.1
Variants: VaAres1_B_FDSW210055061-2r	CA	865	866	2	GG -> CA	600 -> 603	Substitution	26.3% -> 26.4%	3.40E-225	IQ -> IE	hypothetical protein CDS	hypothetical protein	Substitution	KLI70043.1
Variants: VaAres1_B_FDSW210055061-2r	A	881	881	1	G -> A	642	SNP (transition)	48.40%	0		hypothetical protein CDS	hypothetical protein	None	KLI70043.1
Variants: VaAres1_B_FDSW210055061-2r	A	887	887	1	G -> A	644	SNP (transition)	78.40%	0		hypothetical protein CDS	hypothetical protein	None	KLI70043.1
Variants: VaAres1_B_FDSW210055061-2r	A	890	890	1	G -> A	644	SNP (transition)	25.60%	3.70E-191		hypothetical protein CDS	hypothetical protein	None	KLI70043.1
Variants: VaAres1_B_FDSW210055061-2r	G	896	896	1	A -> G	642	SNP (transition)	78.50%	0		hypothetical protein CDS	hypothetical protein	None	KLI70043.1
Variants: VaAres1_B_FDSW210055061-2r	G	908	908	1	A -> G	685	SNP (transition)	31.80%	1.60E-275		hypothetical protein CDS	hypothetical protein	None	KLI70043.1

Variants: VaAres1_B_FDSW210055061-2r	GA C	941	943	2	AAT -> GAC	738 -> 749	Substitution	27.1% -> 27.5%	0	I -> V	hypothetical protein CDS	hypothetical protein	Substituti on	KLI700 43.1
Variants: VaAres1_B_FDSW210055061-2r	C	943	943	1	T -> C	749	SNP (transition)	55.80%	0	I -> V	hypothetical protein CDS	hypothetical protein	Substituti on	KLI700 43.1
Variants: VaAres1_B_FDSW210055061-2r	A	944	944	1	C -> A	757	SNP (transversion)	50.90%	0		hypothetical protein CDS	hypothetical protein	None	KLI700 43.1
Variants: VaAres1_B_FDSW210055061-2r	G	944	944	1	C -> G	757	SNP (transversion)	32.00%	2.20E-235		hypothetical protein CDS	hypothetical protein	None	KLI700 43.1
Variants: VaAres1_B_FDSW210055061-2r	G	947	947	1	C -> G	757	SNP (transversion)	50.60%	0		hypothetical protein CDS	hypothetical protein	None	KLI700 43.1
Variants: VaAres1_B_FDSW210055061-2r	AT	947	948	2	CG -> AT	756 -> 757	Substitution	32.10%	4.40E-215	T -> N	hypothetical protein CDS	hypothetical protein	Substituti on	KLI700 43.1
Variants: VaAres1_B_FDSW210055061-2r	A	950	950	1	G -> A	767	SNP (transition)	50.10%	0		hypothetical protein CDS	hypothetical protein	None	KLI700 43.1
Variants: VaAres1_B_FDSW210055061-2r	CT T	955	957	3	TGG -> CTT	773 -> 785	Substitution	49.7% -> 50.5%	0	TN -> KD	hypothetical protein CDS	hypothetical protein	Substituti on	KLI700 43.1
Variants: VaAres1_B_FDSW210055061-2r	T	959	959	1	C -> T	780	SNP (transition)	49.20%	0		hypothetical protein CDS	hypothetical protein	None	KLI700 43.1
Variants: VaAres1_B_FDSW210055061-2r	G	971	971	1	A -> G	802	SNP (transition)	36.80%	0		hypothetical protein CDS	hypothetical protein	None	KLI700 43.1
Variants: VaAres1_B_FDSW210055061-2r	C	977	977	1	G -> C	804	SNP (transversion)	46.90%	0		hypothetical protein CDS	hypothetical protein	None	KLI700 43.1
Variants: VaAres1_B_FDSW210055061-2r	C	983	983	1	T -> C	794	SNP (transition)	70.00%	0		hypothetical protein CDS	hypothetical protein	None	KLI700 43.1
Variants: VaAres1_B_FDSW210055061-2r	C	986	986	1	G -> C	790	SNP (transversion)	69.90%	0		hypothetical protein CDS	hypothetical protein	None	KLI700 43.1
Variants: VaAres1_B_FDSW210055061-2r	G	1025	1025	1	C -> G	794	SNP (transversion)	46.60%	0		hypothetical protein CDS	hypothetical protein	None	KLI700 43.1
Variants: VaAres1_B_FDSW210055061-2r	T	1025	1025	1	C -> T	794	SNP (transition)	26.10%	7.20E-201		hypothetical protein CDS	hypothetical protein	None	KLI700 43.1
Variants: VaAres1_B_FDSW210055061-2r	G	1028	1028	1	C -> G	794	SNP (transversion)	32.00%	0		hypothetical protein CDS	hypothetical protein	None	KLI700 43.1
Variants: VaAres1_B_FDSW210055061-2r	G	1031	1031	1	A -> G	795	SNP (transition)	25.70%	0		hypothetical protein CDS	hypothetical protein	None	KLI700 43.1
Variants: VaAres1_B_FDSW210055061-2r	T	1054	1054	1	C -> T	850	SNP (transition)	58.10%	0	A -> T	hypothetical protein CDS	hypothetical protein	Substituti on	KLI700 43.1
Variants: VaAres1_B_FDSW210055061-2r	A	1055	1055	1	C -> A	844	SNP (transversion)	34.50%	0		hypothetical protein CDS	hypothetical protein	None	KLI700 43.1
Variants: VaAres1_B_FDSW210055061-2r	G	1055	1055	1	C -> G	844	SNP (transversion)	36.50%	0		hypothetical protein CDS	hypothetical protein	None	KLI700 43.1
Variants: VaAres1_B_FDSW210055061-2r	A	1062	1062	1	T -> A	862	SNP (transversion)	49.90%	0	Y -> F	hypothetical protein CDS	hypothetical protein	Substituti on	KLI700 43.1
Variants: VaAres1_C_FDSW210055062-1r	C	2	2	1	A -> C	892	SNP (transversion)	63.30%	0		hypothetical protein CDS	hypothetical protein	None	KLI700 43.1
Variants: VaAres1_C_FDSW210055062-1r	C	8	8	1	G -> C	886	SNP (transversion)	62.80%	0	D -> E	hypothetical protein CDS	hypothetical protein	Substituti on	KLI700 43.1
Variants: VaAres1_C_FDSW210055062-1r	A	47	47	1	G -> A	691	SNP (transition)	61.10%	0		hypothetical protein CDS	hypothetical protein	None	KLI700 43.1
Variants: VaAres1_C_FDSW210055062-1r	A	53	53	1	G -> A	661	SNP (transition)	64.40%	0		hypothetical protein CDS	hypothetical protein	None	KLI700 43.1
Variants: VaAres1_C_FDSW210055062-1r	A	56	56	1	G -> A	651	SNP (transition)	73.30%	0		hypothetical protein CDS	hypothetical protein	None	KLI700 43.1
Variants: VaAres1_C_FDSW210055062-1r	G	59	59	1	A -> G	631	SNP (transition)	76.90%	0		hypothetical protein CDS	hypothetical protein	None	KLI700 43.1
Variants: VaAres1_C_FDSW210055062-1r	G	62	62	1	A -> G	623	SNP (transition)	75.60%	0		hypothetical protein CDS	hypothetical protein	None	KLI700 43.1
Variants: VaAres1_C_FDSW210055062-1r	G	73	73	1	A -> G	556	SNP (transition)	73.00%	0		hypothetical protein CDS	hypothetical protein	None	KLI700 43.1
Variants: VaAres1_C_FDSW210055062-1r	G	101	101	1	A -> G	389	SNP (transition)	65.00%	0		hypothetical protein CDS	hypothetical protein	None	KLI700 43.1
Variants: VaAres1_C_FDSW210055062-1r	CG	109	110	2	TC -> CG	338 -> 339	Substitution	54.7% -> 54.9%	0	AI -> AV	hypothetical protein CDS	hypothetical protein	Substituti on	KLI700 43.1
Variants: VaAres1_C_FDSW210055062-1r	AT	113	114	2	CG -> AT	315 -> 320	Substitution	51.4% -> 51.9%	5.10E-236	T -> N	hypothetical protein CDS	hypothetical protein	Substituti on	KLI700 43.1

Variants: VaAres1_C_FDSW210055062-1r	C	413	413	1	T -> C	367	SNP (transition)	49.00%	0		hypothetical protein CDS	hypothetical protein	None	KLI700 43.1
Variants: VaAres1_C_FDSW210055062-1r	T	416	416	1	A -> T	398	SNP (transversion)	31.70%	1.80E-297		hypothetical protein CDS	hypothetical protein	None	KLI700 43.1
Variants: VaAres1_C_FDSW210055062-1r	CG C	419	421	2	GGT -> CGC	413 -> 422	Substitution	26.8% -> 27.4%	1.10E-256	T -> A	hypothetical protein CDS	hypothetical protein	Substituti on	KLI700 43.1
Variants: VaAres1_C_FDSW210055062-1r	T	422	422	1	A -> T	441	SNP (transversion)	42.20%	0		hypothetical protein CDS	hypothetical protein	None	KLI700 43.1
Variants: VaAres1_C_FDSW210055062-1r	T	425	425	1	C -> T	483	SNP (transition)	34.00%	0		hypothetical protein CDS	hypothetical protein	None	KLI700 43.1
Variants: VaAres1_C_FDSW210055062-1r	G	428	428	1	A -> G	504	SNP (transition)	54.60%	0		hypothetical protein CDS	hypothetical protein	None	KLI700 43.1
Variants: VaAres1_C_FDSW210055062-1r	C	452	452	1	T -> C	704	SNP (transition)	35.20%	0		hypothetical protein CDS	hypothetical protein	None	KLI700 43.1
Variants: VaAres1_C_FDSW210055062-1r	A	455	455	1	G -> A	728	SNP (transition)	37.50%	0		hypothetical protein CDS	hypothetical protein	None	KLI700 43.1
Variants: VaAres1_C_FDSW210055062-1r	G	473	473	1	C -> G	891	SNP (transversion)	55.40%	0		hypothetical protein CDS	hypothetical protein	None	KLI700 43.1
Variants: VaAres1_C_FDSW210055062-1r	G	479	479	1	C -> G	944	SNP (transversion)	57.90%	0		hypothetical protein CDS	hypothetical protein	None	KLI700 43.1
Variants: VaAres1_C_FDSW210055062-1r	TA G	482	484	2	CAA -> TAG	973 -> 999	Substitution	31.4% -> 32.3%	0		hypothetical protein CDS	hypothetical protein	None	KLI700 43.1
Variants: VaAres1_C_FDSW210055062-1r	GG T	485	487	2	CGC -> GGT	1009 -> 1028	Substitution	32.3% -> 32.9%	0	A -> T	hypothetical protein CDS	hypothetical protein	Substituti on	KLI700 43.1
Variants: VaAres1_C_FDSW210055062-1r	A	497	497	1	G -> A	1141	SNP (transition)	33.60%	0		hypothetical protein CDS	hypothetical protein	None	KLI700 43.1
Variants: VaAres1_C_FDSW210055062-1r	C	509	509	1	G -> C	1256	SNP (transversion)	49.50%	0		hypothetical protein CDS	hypothetical protein	None	KLI700 43.1
Variants: VaAres1_C_FDSW210055062-1r	G	530	530	1	C -> G	1534	SNP (transversion)	31.90%	0		hypothetical protein CDS	hypothetical protein	None	KLI700 43.1
Variants: VaAres1_C_FDSW210055062-1r	A	563	563	1	G -> A	1747	SNP (transition)	31.80%	0		hypothetical protein CDS	hypothetical protein	None	KLI700 43.1
Variants: VaAres1_C_FDSW210055062-1r	A	608	608	1	G -> A	1908	SNP (transition)	45.20%	0		hypothetical protein CDS	hypothetical protein	None	KLI700 43.1
Variants: VaAres1_C_FDSW210055062-1r	G	614	614	1	A -> G	1907	SNP (transition)	36.10%	0		hypothetical protein CDS	hypothetical protein	None	KLI700 43.1
Variants: VaAres1_C_FDSW210055062-1r	T	623	623	1	C -> T	1907	SNP (transition)	27.90%	0		hypothetical protein CDS	hypothetical protein	None	KLI700 43.1
Variants: VaAres1_C_FDSW210055062-1r	C	628	628	1	T -> C	1912	SNP (transition)	43.40%	0	N -> D	hypothetical protein CDS	hypothetical protein	Substituti on	KLI700 43.1
Variants: VaAres1_C_FDSW210055062-1r	A	644	644	1	G -> A	1883	SNP (transition)	27.00%	0		hypothetical protein CDS	hypothetical protein	None	KLI700 43.1
Variants: VaAres1_C_FDSW210055062-1r	G	671	671	1	A -> G	1781	SNP (transition)	28.50%	0		hypothetical protein CDS	hypothetical protein	None	KLI700 43.1
Variants: VaAres1_C_FDSW210055062-1r	T	675	675	1	C -> T	1741	SNP (transition)	30.30%	0	S -> N	hypothetical protein CDS	hypothetical protein	Substituti on	KLI700 43.1
Variants: VaAres1_C_FDSW210055062-1r	A	677	677	1	G -> A	1692	SNP (transition)	40.70%	0		hypothetical protein CDS	hypothetical protein	None	KLI700 43.1
Variants: VaAres1_C_FDSW210055062-1r	A	722	722	1	G -> A	1454	SNP (transition)	28.80%	0		hypothetical protein CDS	hypothetical protein	None	KLI700 43.1
Variants: VaAres1_C_FDSW210055062-1r	T	728	728	1	C -> T	1397	SNP (transition)	27.10%	0		hypothetical protein CDS	hypothetical protein	None	KLI700 43.1
Variants: VaAres1_C_FDSW210055062-1r	A	737	737	1	G -> A	1305	SNP (transition)	71.00%	0		hypothetical protein CDS	hypothetical protein	None	KLI700 43.1
Variants: VaAres1_C_FDSW210055062-1r	A	745	745	1	C -> A	1238	SNP (transversion)	28.80%	0	A -> S	hypothetical protein CDS	hypothetical protein	Substituti on	KLI700 43.1
Variants: VaAres1_C_FDSW210055062-1r	C	752	752	1	G -> C	1180	SNP (transversion)	29.50%	0		hypothetical protein CDS	hypothetical protein	None	KLI700 43.1
Variants: VaAres1_C_FDSW210055062-1r	A	755	755	1	C -> A	1156	SNP (transversion)	32.70%	0		hypothetical protein CDS	hypothetical protein	None	KLI700 43.1
Variants: VaAres1_C_FDSW210055062-1r	A	767	767	1	G -> A	1083	SNP (transition)	25.60%	0		hypothetical protein CDS	hypothetical protein	None	KLI700 43.1
Variants: VaAres1_C_FDSW210055062-1r	G	806	806	1	C -> G	911	SNP (transversion)	75.50%	0		hypothetical protein CDS	hypothetical protein	None	KLI700 43.1

Variants: VaAres1_C_FDSW210055062-1r	C	823	823	1	T -> C	871	SNP (transition)	62.50%	0	I -> V	hypothetical protein CDS	hypothetical protein	Substitution	KLI70043.1
Variants: VaAres1_C_FDSW210055062-1r	T	834	834	1	G -> T	847	SNP (transversion)	58.00%	0	T -> K	hypothetical protein CDS	hypothetical protein	Substitution	KLI70043.1
Variants: VaAres1_C_FDSW210055062-1r	C	844	844	1	T -> C	825	SNP (transition)	74.10%	0	T -> A	hypothetical protein CDS	hypothetical protein	Substitution	KLI70043.1
Variants: VaAres1_C_FDSW210055062-1r	A	854	854	1	G -> A	832	SNP (transition)	66.60%	0		hypothetical protein CDS	hypothetical protein	None	KLI70043.1
Variants: VaAres1_C_FDSW210055062-1r	CA	865	866	2	GG -> CA	831 -> 833	Substitution	31.0% -> 31.2%	0	IQ -> IE	hypothetical protein CDS	hypothetical protein	Substitution	KLI70043.1
Variants: VaAres1_C_FDSW210055062-1r	A	874	874	1	G -> A	852	SNP (transition)	26.40%	1.70E-307		hypothetical protein CDS	hypothetical protein	None	KLI70043.1
Variants: VaAres1_C_FDSW210055062-1r	A	881	881	1	G -> A	869	SNP (transition)	47.40%	0		hypothetical protein CDS	hypothetical protein	None	KLI70043.1
Variants: VaAres1_C_FDSW210055062-1r	A	887	887	1	G -> A	874	SNP (transition)	81.20%	0		hypothetical protein CDS	hypothetical protein	None	KLI70043.1
Variants: VaAres1_C_FDSW210055062-1r	A	890	890	1	G -> A	886	SNP (transition)	28.30%	0		hypothetical protein CDS	hypothetical protein	None	KLI70043.1
Variants: VaAres1_C_FDSW210055062-1r	G	896	896	1	A -> G	883	SNP (transition)	81.50%	0		hypothetical protein CDS	hypothetical protein	None	KLI70043.1
Variants: VaAres1_C_FDSW210055062-1r	G	908	908	1	A -> G	939	SNP (transition)	32.80%	0		hypothetical protein CDS	hypothetical protein	None	KLI70043.1
Variants: VaAres1_C_FDSW210055062-1r	GAC	941	943	2	AAT -> GAC	1031 -> 1033	Substitution	26.6% -> 27.2%	0	I -> V	hypothetical protein CDS	hypothetical protein	Substitution	KLI70043.1
Variants: VaAres1_C_FDSW210055062-1r	C	943	943	1	T -> C	1033	SNP (transition)	55.40%	0	I -> V	hypothetical protein CDS	hypothetical protein	Substitution	KLI70043.1
Variants: VaAres1_C_FDSW210055062-1r	A	944	944	1	C -> A	1036	SNP (transversion)	50.10%	0		hypothetical protein CDS	hypothetical protein	None	KLI70043.1
Variants: VaAres1_C_FDSW210055062-1r	G	944	944	1	C -> G	1036	SNP (transversion)	31.90%	0		hypothetical protein CDS	hypothetical protein	None	KLI70043.1
Variants: VaAres1_C_FDSW210055062-1r	G	947	947	1	C -> G	1046	SNP (transversion)	49.90%	0		hypothetical protein CDS	hypothetical protein	None	KLI70043.1
Variants: VaAres1_C_FDSW210055062-1r	AT	947	948	2	CG -> AT	1046 -> 1054	Substitution	32.1% -> 32.3%	0	T -> N	hypothetical protein CDS	hypothetical protein	Substitution	KLI70043.1
Variants: VaAres1_C_FDSW210055062-1r	A	950	950	1	G -> A	1058	SNP (transition)	49.30%	0		hypothetical protein CDS	hypothetical protein	None	KLI70043.1
Variants: VaAres1_C_FDSW210055062-1r	CTT	955	957	3	TGG -> CTT	1083 -> 1094	Substitution	48.4% -> 48.5%	0	TN -> KD	hypothetical protein CDS	hypothetical protein	Substitution	KLI70043.1
Variants: VaAres1_C_FDSW210055062-1r	T	959	959	1	C -> T	1092	SNP (transition)	47.70%	0		hypothetical protein CDS	hypothetical protein	None	KLI70043.1
Variants: VaAres1_C_FDSW210055062-1r	G	971	971	1	A -> G	1093	SNP (transition)	38.00%	0		hypothetical protein CDS	hypothetical protein	None	KLI70043.1
Variants: VaAres1_C_FDSW210055062-1r	C	977	977	1	G -> C	1102	SNP (transversion)	48.30%	0		hypothetical protein CDS	hypothetical protein	None	KLI70043.1
Variants: VaAres1_C_FDSW210055062-1r	C	983	983	1	T -> C	1104	SNP (transition)	66.00%	0		hypothetical protein CDS	hypothetical protein	None	KLI70043.1
Variants: VaAres1_C_FDSW210055062-1r	C	986	986	1	G -> C	1114	SNP (transversion)	66.20%	0		hypothetical protein CDS	hypothetical protein	None	KLI70043.1
Variants: VaAres1_C_FDSW210055062-1r	T	1013	1013	1	C -> T	1144	SNP (transition)	25.20%	7.50E-274		hypothetical protein CDS	hypothetical protein	None	KLI70043.1
Variants: VaAres1_C_FDSW210055062-1r	G	1025	1025	1	C -> G	1141	SNP (transversion)	47.00%	0		hypothetical protein CDS	hypothetical protein	None	KLI70043.1
Variants: VaAres1_C_FDSW210055062-1r	T	1025	1025	1	C -> T	1141	SNP (transition)	27.60%	2.3E-313		hypothetical protein CDS	hypothetical protein	None	KLI70043.1
Variants: VaAres1_C_FDSW210055062-1r	G	1028	1028	1	C -> G	1144	SNP (transversion)	31.60%	0		hypothetical protein CDS	hypothetical protein	None	KLI70043.1
Variants: VaAres1_C_FDSW210055062-1r	T	1054	1054	1	C -> T	1248	SNP (transition)	54.50%	0	A -> T	hypothetical protein CDS	hypothetical protein	Substitution	KLI70043.1
Variants: VaAres1_C_FDSW210055062-1r	A	1055	1055	1	C -> A	1243	SNP (transversion)	33.50%	0		hypothetical protein CDS	hypothetical protein	None	KLI70043.1
Variants: VaAres1_C_FDSW210055062-1r	G	1055	1055	1	C -> G	1243	SNP (transversion)	36.40%	0		hypothetical protein CDS	hypothetical protein	None	KLI70043.1
Variants: VaAres1_C_FDSW210055062-1r	A	1062	1062	1	T -> A	1250	SNP (transversion)	50.30%	0	Y -> F	hypothetical protein CDS	hypothetical protein	Substitution	KLI70043.1

Variants: VaphiVaphiSt2_B_A_FDSW210055054-2r	C	2	2	1	A -> C	451	SNP (transversion)	63.40%	0		hypothetical protein CDS	hypothetical protein	None	KLI70043.1
Variants: VaphiVaphiSt2_B_A_FDSW210055054-2r	C	8	8	1	G -> C	447	SNP (transversion)	62.90%	0	D -> E	hypothetical protein CDS	hypothetical protein	Substitution	KLI70043.1
Variants: VaphiVaphiSt2_B_A_FDSW210055054-2r	A	47	47	1	G -> A	360	SNP (transition)	59.70%	0		hypothetical protein CDS	hypothetical protein	None	KLI70043.1
Variants: VaphiVaphiSt2_B_A_FDSW210055054-2r	A	53	53	1	G -> A	331	SNP (transition)	62.50%	0		hypothetical protein CDS	hypothetical protein	None	KLI70043.1
Variants: VaphiVaphiSt2_B_A_FDSW210055054-2r	A	56	56	1	G -> A	322	SNP (transition)	71.70%	0		hypothetical protein CDS	hypothetical protein	None	KLI70043.1
Variants: VaphiVaphiSt2_B_A_FDSW210055054-2r	G	59	59	1	A -> G	317	SNP (transition)	74.10%	0		hypothetical protein CDS	hypothetical protein	None	KLI70043.1
Variants: VaphiVaphiSt2_B_A_FDSW210055054-2r	G	62	62	1	A -> G	309	SNP (transition)	73.50%	0		hypothetical protein CDS	hypothetical protein	None	KLI70043.1
Variants: VaphiVaphiSt2_B_A_FDSW210055054-2r	G	73	73	1	A -> G	292	SNP (transition)	72.60%	0		hypothetical protein CDS	hypothetical protein	None	KLI70043.1
Variants: VaphiVaphiSt2_B_A_FDSW210055054-2r	G	101	101	1	A -> G	217	SNP (transition)	58.10%	2.40E-215		hypothetical protein CDS	hypothetical protein	None	KLI70043.1
Variants: VaphiVaphiSt2_B_A_FDSW210055054-2r	CG	109	110	2	TC -> CG	186 -> 188	Substitution	45.7% -> 46.3%	2.60E-108	AI -> AV	hypothetical protein CDS	hypothetical protein	Substitution	KLI70043.1
Variants: VaphiVaphiSt2_B_A_FDSW210055054-2r	AT	113	114	2	CG -> AT	178 -> 181	Substitution	41.6% -> 42.0%	3.70E-76	T -> N	hypothetical protein CDS	hypothetical protein	Substitution	KLI70043.1
Variants: VaphiVaphiSt2_B_A_FDSW210055054-2r	T	392	392	1	G -> T	109	SNP (transversion)	25.70%	9.30E-26	N -> K	hypothetical protein CDS	hypothetical protein	Substitution	KLI70043.1
Variants: VaphiVaphiSt2_B_A_FDSW210055054-2r	C	413	413	1	T -> C	184	SNP (transition)	59.80%	3.90E-278		hypothetical protein CDS	hypothetical protein	None	KLI70043.1
Variants: VaphiVaphiSt2_B_A_FDSW210055054-2r	T	416	416	1	A -> T	198	SNP (transversion)	43.40%	9.50E-210		hypothetical protein CDS	hypothetical protein	None	KLI70043.1
Variants: VaphiVaphiSt2_B_A_FDSW210055054-2r	CGC	419	421	2	GGT -> CGC	207 -> 214	Substitution	30.8% -> 31.9%	5.40E-168	T -> A	hypothetical protein CDS	hypothetical protein	Substitution	KLI70043.1
Variants: VaphiVaphiSt2_B_A_FDSW210055054-2r	C	421	421	1	T -> C	214	SNP (transition)	31.80%	2.70E-181	T -> A	hypothetical protein CDS	hypothetical protein	Substitution	KLI70043.1
Variants: VaphiVaphiSt2_B_A_FDSW210055054-2r	T	422	422	1	A -> T	220	SNP (transversion)	51.80%	7.10E-278		hypothetical protein CDS	hypothetical protein	None	KLI70043.1
Variants: VaphiVaphiSt2_B_A_FDSW210055054-2r	T	425	425	1	C -> T	232	SNP (transition)	37.50%	8.90E-214		hypothetical protein CDS	hypothetical protein	None	KLI70043.1
Variants: VaphiVaphiSt2_B_A_FDSW210055054-2r	G	428	428	1	A -> G	250	SNP (transition)	62.00%	0		hypothetical protein CDS	hypothetical protein	None	KLI70043.1
Variants: VaphiVaphiSt2_B_A_FDSW210055054-2r	C	452	452	1	T -> C	345	SNP (transition)	40.60%	0		hypothetical protein CDS	hypothetical protein	None	KLI70043.1
Variants: VaphiVaphiSt2_B_A_FDSW210055054-2r	A	455	455	1	G -> A	356	SNP (transition)	37.90%	0		hypothetical protein CDS	hypothetical protein	None	KLI70043.1
Variants: VaphiVaphiSt2_B_A_FDSW210055054-2r	G	473	473	1	C -> G	445	SNP (transversion)	59.10%	0		hypothetical protein CDS	hypothetical protein	None	KLI70043.1

Variants: VaphiVaphiSt2_B_A_FDSW2100550 54-2r	G	479	479	1	C -> G	480	SNP (transversion)	60.60%	0		hypothetical protein CDS	hypothetical protein	None	KLI700 43.1
Variants: VaphiVaphiSt2_B_A_FDSW2100550 54-2r	TA G	482	484	2	CAA -> TAG	500 -> 510	Substitution	34.3% -> 35.0%	0		hypothetical protein CDS	hypothetical protein	None	KLI700 43.1
Variants: VaphiVaphiSt2_B_A_FDSW2100550 54-2r	GG T	485	487	2	CGC -> GGT	521 -> 529	Substitution	34.4% -> 34.9%	0	A -> T	hypothetical protein CDS	hypothetical protein	Substituti on	KLI700 43.1
Variants: VaphiVaphiSt2_B_A_FDSW2100550 54-2r	A	497	497	1	G -> A	590	SNP (transition)	35.60%	0		hypothetical protein CDS	hypothetical protein	None	KLI700 43.1
Variants: VaphiVaphiSt2_B_A_FDSW2100550 54-2r	C	509	509	1	G -> C	660	SNP (transversion)	51.20%	0		hypothetical protein CDS	hypothetical protein	None	KLI700 43.1
Variants: VaphiVaphiSt2_B_A_FDSW2100550 54-2r	G	530	530	1	C -> G	773	SNP (transversion)	31.00%	0		hypothetical protein CDS	hypothetical protein	None	KLI700 43.1
Variants: VaphiVaphiSt2_B_A_FDSW2100550 54-2r	A	563	563	1	G -> A	891	SNP (transition)	30.30%	0		hypothetical protein CDS	hypothetical protein	None	KLI700 43.1
Variants: VaphiVaphiSt2_B_A_FDSW2100550 54-2r	A	608	608	1	G -> A	1044	SNP (transition)	46.30%	0		hypothetical protein CDS	hypothetical protein	None	KLI700 43.1
Variants: VaphiVaphiSt2_B_A_FDSW2100550 54-2r	G	614	614	1	A -> G	1063	SNP (transition)	36.10%	0		hypothetical protein CDS	hypothetical protein	None	KLI700 43.1
Variants: VaphiVaphiSt2_B_A_FDSW2100550 54-2r	T	623	623	1	C -> T	1076	SNP (transition)	28.10%	0		hypothetical protein CDS	hypothetical protein	None	KLI700 43.1
Variants: VaphiVaphiSt2_B_A_FDSW2100550 54-2r	C	628	628	1	T -> C	1076	SNP (transition)	44.20%	0	N -> D	hypothetical protein CDS	hypothetical protein	Substituti on	KLI700 43.1
Variants: VaphiVaphiSt2_B_A_FDSW2100550 54-2r	A	644	644	1	G -> A	1051	SNP (transition)	27.60%	0		hypothetical protein CDS	hypothetical protein	None	KLI700 43.1
Variants: VaphiVaphiSt2_B_A_FDSW2100550 54-2r	G	671	671	1	A -> G	1012	SNP (transition)	30.60%	0		hypothetical protein CDS	hypothetical protein	None	KLI700 43.1
Variants: VaphiVaphiSt2_B_A_FDSW2100550 54-2r	T	675	675	1	C -> T	1000	SNP (transition)	30.10%	0	S -> N	hypothetical protein CDS	hypothetical protein	Substituti on	KLI700 43.1
Variants: VaphiVaphiSt2_B_A_FDSW2100550 54-2r	A	677	677	1	G -> A	990	SNP (transition)	38.90%	0		hypothetical protein CDS	hypothetical protein	None	KLI700 43.1
Variants: VaphiVaphiSt2_B_A_FDSW2100550 54-2r	A	722	722	1	G -> A	858	SNP (transition)	28.70%	0		hypothetical protein CDS	hypothetical protein	None	KLI700 43.1
Variants: VaphiVaphiSt2_B_A_FDSW2100550 54-2r	T	728	728	1	C -> T	816	SNP (transition)	26.00%	0		hypothetical protein CDS	hypothetical protein	None	KLI700 43.1
Variants: VaphiVaphiSt2_B_A_FDSW2100550 54-2r	A	737	737	1	G -> A	757	SNP (transition)	71.50%	0		hypothetical protein CDS	hypothetical protein	None	KLI700 43.1
Variants: VaphiVaphiSt2_B_A_FDSW2100550 54-2r	A	745	745	1	C -> A	724	SNP (transversion)	27.30%	0	A -> S	hypothetical protein CDS	hypothetical protein	Substituti on	KLI700 43.1
Variants: VaphiVaphiSt2_B_A_FDSW2100550 54-2r	C	752	752	1	G -> C	706	SNP (transversion)	28.00%	0		hypothetical protein CDS	hypothetical protein	None	KLI700 43.1
Variants: VaphiVaphiSt2_B_A_FDSW2100550 54-2r	A	755	755	1	C -> A	688	SNP (transversion)	35.00%	0		hypothetical protein CDS	hypothetical protein	None	KLI700 43.1
Variants: VaphiVaphiSt2_B_A_FDSW2100550 54-2r	A	767	767	1	G -> A	633	SNP (transition)	25.30%	0		hypothetical protein CDS	hypothetical protein	None	KLI700 43.1

Variants: VaphiVaphiSt2_B_A_FDSW2100550 54-2r	G	776	776	1	A -> G	588	SNP (transition)	27.40%	0		hypothetical protein CDS	hypothetical protein	None	KLI700 43.1
Variants: VaphiVaphiSt2_B_A_FDSW2100550 54-2r	A	779	779	1	G -> A	582	SNP (transition)	26.80%	2.0E-323		hypothetical protein CDS	hypothetical protein	None	KLI700 43.1
Variants: VaphiVaphiSt2_B_A_FDSW2100550 54-2r	G	806	806	1	C -> G	537	SNP (transversion)	77.10%	0		hypothetical protein CDS	hypothetical protein	None	KLI700 43.1
Variants: VaphiVaphiSt2_B_A_FDSW2100550 54-2r	C	823	823	1	T -> C	518	SNP (transition)	62.50%	0	I -> V	hypothetical protein CDS	hypothetical protein	Substituti on	KLI700 43.1
Variants: VaphiVaphiSt2_B_A_FDSW2100550 54-2r	T	834	834	1	G -> T	496	SNP (transversion)	59.30%	0	T -> K	hypothetical protein CDS	hypothetical protein	Substituti on	KLI700 43.1
Variants: VaphiVaphiSt2_B_A_FDSW2100550 54-2r	C	844	844	1	T -> C	478	SNP (transition)	78.20%	0	T -> A	hypothetical protein CDS	hypothetical protein	Substituti on	KLI700 43.1
Variants: VaphiVaphiSt2_B_A_FDSW2100550 54-2r	A	854	854	1	G -> A	475	SNP (transition)	68.60%	0		hypothetical protein CDS	hypothetical protein	None	KLI700 43.1
Variants: VaphiVaphiSt2_B_A_FDSW2100550 54-2r	CA	865	866	2	GG -> CA	470 -> 473	Substitution	32.3% -> 32.6%	2.90E-245	IQ -> IE	hypothetical protein CDS	hypothetical protein	Substituti on	KLI700 43.1
Variants: VaphiVaphiSt2_B_A_FDSW2100550 54-2r	A	874	874	1	G -> A	472	SNP (transition)	29.00%	5.10E-208		hypothetical protein CDS	hypothetical protein	None	KLI700 43.1
Variants: VaphiVaphiSt2_B_A_FDSW2100550 54-2r	A	881	881	1	G -> A	486	SNP (transition)	45.70%	0		hypothetical protein CDS	hypothetical protein	None	KLI700 43.1
Variants: VaphiVaphiSt2_B_A_FDSW2100550 54-2r	A	887	887	1	G -> A	493	SNP (transition)	81.10%	0		hypothetical protein CDS	hypothetical protein	None	KLI700 43.1
Variants: VaphiVaphiSt2_B_A_FDSW2100550 54-2r	A	890	890	1	G -> A	492	SNP (transition)	27.00%	5.90E-158		hypothetical protein CDS	hypothetical protein	None	KLI700 43.1
Variants: VaphiVaphiSt2_B_A_FDSW2100550 54-2r	G	896	896	1	A -> G	489	SNP (transition)	82.60%	0		hypothetical protein CDS	hypothetical protein	None	KLI700 43.1
Variants: VaphiVaphiSt2_B_A_FDSW2100550 54-2r	G	908	908	1	A -> G	504	SNP (transition)	34.50%	8.50E-245		hypothetical protein CDS	hypothetical protein	None	KLI700 43.1
Variants: VaphiVaphiSt2_B_A_FDSW2100550 54-2r	GA C	941	943	2	AAT -> GAC	542	Substitution	28.00%	2.10E-291	I -> V	hypothetical protein CDS	hypothetical protein	Substituti on	KLI700 43.1
Variants: VaphiVaphiSt2_B_A_FDSW2100550 54-2r	C	943	943	1	T -> C	542	SNP (transition)	55.20%	0	I -> V	hypothetical protein CDS	hypothetical protein	Substituti on	KLI700 43.1
Variants: VaphiVaphiSt2_B_A_FDSW2100550 54-2r	A	944	944	1	C -> A	546	SNP (transversion)	51.80%	0		hypothetical protein CDS	hypothetical protein	None	KLI700 43.1
Variants: VaphiVaphiSt2_B_A_FDSW2100550 54-2r	G	944	944	1	C -> G	546	SNP (transversion)	31.90%	2.20E-186		hypothetical protein CDS	hypothetical protein	None	KLI700 43.1
Variants: VaphiVaphiSt2_B_A_FDSW2100550 54-2r	G	947	947	1	C -> G	551	SNP (transversion)	51.70%	0		hypothetical protein CDS	hypothetical protein	None	KLI700 43.1
Variants: VaphiVaphiSt2_B_A_FDSW2100550 54-2r	AT	947	948	2	CG -> AT	546 -> 551	Substitution	31.8% -> 32.1%	1.20E-170	T -> N	hypothetical protein CDS	hypothetical protein	Substituti on	KLI700 43.1
Variants: VaphiVaphiSt2_B_A_FDSW2100550 54-2r	A	950	950	1	G -> A	546	SNP (transition)	50.40%	0		hypothetical protein CDS	hypothetical protein	None	KLI700 43.1
Variants: VaphiVaphiSt2_B_A_FDSW2100550 54-2r	CT T	955	957	3	TGG -> CTT	558 -> 562	Substitution	49.5% -> 50.2%	0	TN -> KD	hypothetical protein CDS	hypothetical protein	Substituti on	KLI700 43.1

Variants: VaphiVaphiSt2_B_A_FDSW210055054-2r	T	959	959	1	C -> T	559	SNP (transition)	49.60%	0		hypothetical protein CDS	hypothetical protein	None	KLI70043.1
Variants: VaphiVaphiSt2_B_A_FDSW210055054-2r	G	971	971	1	A -> G	557	SNP (transition)	38.60%	0		hypothetical protein CDS	hypothetical protein	None	KLI70043.1
Variants: VaphiVaphiSt2_B_A_FDSW210055054-2r	G	974	974	1	A -> G	551	SNP (transition)	25.20%	2.50E-133		hypothetical protein CDS	hypothetical protein	None	KLI70043.1
Variants: VaphiVaphiSt2_B_A_FDSW210055054-2r	C	977	977	1	G -> C	553	SNP (transversion)	50.30%	0		hypothetical protein CDS	hypothetical protein	None	KLI70043.1
Variants: VaphiVaphiSt2_B_A_FDSW210055054-2r	C	983	983	1	T -> C	545	SNP (transition)	69.90%	0		hypothetical protein CDS	hypothetical protein	None	KLI70043.1
Variants: VaphiVaphiSt2_B_A_FDSW210055054-2r	C	986	986	1	G -> C	544	SNP (transversion)	69.90%	0		hypothetical protein CDS	hypothetical protein	None	KLI70043.1
Variants: VaphiVaphiSt2_B_A_FDSW210055054-2r	T	1013	1013	1	C -> T	587	SNP (transition)	25.60%	3.70E-159		hypothetical protein CDS	hypothetical protein	None	KLI70043.1
Variants: VaphiVaphiSt2_B_A_FDSW210055054-2r	G	1025	1025	1	C -> G	610	SNP (transversion)	48.20%	0		hypothetical protein CDS	hypothetical protein	None	KLI70043.1
Variants: VaphiVaphiSt2_B_A_FDSW210055054-2r	T	1025	1025	1	C -> T	610	SNP (transition)	29.00%	8.30E-216		hypothetical protein CDS	hypothetical protein	None	KLI70043.1
Variants: VaphiVaphiSt2_B_A_FDSW210055054-2r	G	1028	1028	1	C -> G	612	SNP (transversion)	34.30%	2.30E-294		hypothetical protein CDS	hypothetical protein	None	KLI70043.1
Variants: VaphiVaphiSt2_B_A_FDSW210055054-2r	T	1054	1054	1	C -> T	645	SNP (transition)	62.30%	0	A -> T	hypothetical protein CDS	hypothetical protein	Substitution	KLI70043.1
Variants: VaphiVaphiSt2_B_A_FDSW210055054-2r	A	1055	1055	1	C -> A	644	SNP (transversion)	29.80%	0		hypothetical protein CDS	hypothetical protein	None	KLI70043.1
Variants: VaphiVaphiSt2_B_A_FDSW210055054-2r	G	1055	1055	1	C -> G	644	SNP (transversion)	43.20%	0		hypothetical protein CDS	hypothetical protein	None	KLI70043.1
Variants: VaphiVaphiSt2_B_A_FDSW210055054-2r	A	1062	1062	1	T -> A	643	SNP (transversion)	57.20%	0	Y -> F	hypothetical protein CDS	hypothetical protein	Substitution	KLI70043.1
Variants: VaphiSt2_B_FDSW210055056-1r	C	2	2	1	A -> C	1058	SNP (transversion)	57.20%	0		hypothetical protein CDS	hypothetical protein	None	KLI70043.1
Variants: VaphiSt2_B_FDSW210055056-1r	C	8	8	1	G -> C	1046	SNP (transversion)	56.40%	0	D -> E	hypothetical protein CDS	hypothetical protein	Substitution	KLI70043.1
Variants: VaphiSt2_B_FDSW210055056-1r	A	47	47	1	G -> A	850	SNP (transition)	52.60%	0		hypothetical protein CDS	hypothetical protein	None	KLI70043.1
Variants: VaphiSt2_B_FDSW210055056-1r	A	53	53	1	G -> A	822	SNP (transition)	58.30%	0		hypothetical protein CDS	hypothetical protein	None	KLI70043.1
Variants: VaphiSt2_B_FDSW210055056-1r	A	56	56	1	G -> A	811	SNP (transition)	65.60%	0		hypothetical protein CDS	hypothetical protein	None	KLI70043.1
Variants: VaphiSt2_B_FDSW210055056-1r	G	59	59	1	A -> G	790	SNP (transition)	71.40%	0		hypothetical protein CDS	hypothetical protein	None	KLI70043.1
Variants: VaphiSt2_B_FDSW210055056-1r	G	62	62	1	A -> G	773	SNP (transition)	71.40%	0		hypothetical protein CDS	hypothetical protein	None	KLI70043.1
Variants: VaphiSt2_B_FDSW210055056-1r	G	73	73	1	A -> G	713	SNP (transition)	68.20%	0		hypothetical protein CDS	hypothetical protein	None	KLI70043.1
Variants: VaphiSt2_B_FDSW210055056-1r	G	101	101	1	A -> G	521	SNP (transition)	59.70%	0		hypothetical protein CDS	hypothetical protein	None	KLI70043.1
Variants: VaphiSt2_B_FDSW210055056-1r	C	109	109	1	T -> C	477	SNP (transition)	56.80%	0	I -> V	hypothetical protein CDS	hypothetical protein	Substitution	KLI70043.1
Variants: VaphiSt2_B_FDSW210055056-1r	G	110	110	1	C -> G	464	SNP (transversion)	44.60%	9.4E-320		hypothetical protein CDS	hypothetical protein	None	KLI70043.1
Variants: VaphiSt2_B_FDSW210055056-1r	AT	113	114	2	CG -> AT	446 -> 450	Substitution	42.6% -> 42.9%	7.20E-255	T -> N	hypothetical protein CDS	hypothetical protein	Substitution	KLI70043.1

Variants: VaphiSt2_B_FDSW210055056-1r	C	386	386	1	T -> C	308	SNP (transition)	41.60%	1.50E-282		hypothetical protein CDS	hypothetical protein	None	KLI70043.1
Variants: VaphiSt2_B_FDSW210055056-1r	T	392	392	1	G -> T	359	SNP (transversion)	44.00%	0	N -> K	hypothetical protein CDS	hypothetical protein	Substitution	KLI70043.1
Variants: VaphiSt2_B_FDSW210055056-1r	A	395	395	1	G -> A	396	SNP (transition)	46.00%	0		hypothetical protein CDS	hypothetical protein	None	KLI70043.1
Variants: VaphiSt2_B_FDSW210055056-1r	A	407	407	1	G -> A	512	SNP (transition)	27.50%	0.00E+00		hypothetical protein CDS	hypothetical protein	None	KLI70043.1
Variants: VaphiSt2_B_FDSW210055056-1r	C	413	413	1	T -> C	575	SNP (transition)	69.00%	0		hypothetical protein CDS	hypothetical protein	None	KLI70043.1
Variants: VaphiSt2_B_FDSW210055056-1r	T	416	416	1	A -> T	595	SNP (transversion)	55.30%	0		hypothetical protein CDS	hypothetical protein	None	KLI70043.1
Variants: VaphiSt2_B_FDSW210055056-1r	CGC	419	421	2	GGT -> CGC	631 -> 649	Substitution	31.1% -> 32.0%	0	T -> A	hypothetical protein CDS	hypothetical protein	Substitution	KLI70043.1
Variants: VaphiSt2_B_FDSW210055056-1r	C	421	421	1	T -> C	649	SNP (transition)	31.00%	0	T -> A	hypothetical protein CDS	hypothetical protein	Substitution	KLI70043.1
Variants: VaphiSt2_B_FDSW210055056-1r	T	422	422	1	A -> T	664	SNP (transversion)	58.30%	0		hypothetical protein CDS	hypothetical protein	None	KLI70043.1
Variants: VaphiSt2_B_FDSW210055056-1r	T	425	425	1	C -> T	697	SNP (transition)	38.00%	0		hypothetical protein CDS	hypothetical protein	None	KLI70043.1
Variants: VaphiSt2_B_FDSW210055056-1r	G	428	428	1	A -> G	722	SNP (transition)	67.20%	0		hypothetical protein CDS	hypothetical protein	None	KLI70043.1
Variants: VaphiSt2_B_FDSW210055056-1r	C	452	452	1	T -> C	973	SNP (transition)	42.20%	0		hypothetical protein CDS	hypothetical protein	None	KLI70043.1
Variants: VaphiSt2_B_FDSW210055056-1r	A	455	455	1	G -> A	1018	SNP (transition)	38.60%	0		hypothetical protein CDS	hypothetical protein	None	KLI70043.1
Variants: VaphiSt2_B_FDSW210055056-1r	G	464	464	1	A -> G	1092	SNP (transition)	25.40%	0		hypothetical protein CDS	hypothetical protein	None	KLI70043.1
Variants: VaphiSt2_B_FDSW210055056-1r	A	470	470	1	G -> A	1160	SNP (transition)	26.00%	0		hypothetical protein CDS	hypothetical protein	None	KLI70043.1
Variants: VaphiSt2_B_FDSW210055056-1r	G	473	473	1	C -> G	1193	SNP (transversion)	58.30%	0		hypothetical protein CDS	hypothetical protein	None	KLI70043.1
Variants: VaphiSt2_B_FDSW210055056-1r	G	479	479	1	C -> G	1271	SNP (transversion)	64.80%	0		hypothetical protein CDS	hypothetical protein	None	KLI70043.1
Variants: VaphiSt2_B_FDSW210055056-1r	TAG	482	484	2	CAA -> TAG	1302 -> 1314	Substitution	33.4% -> 33.7%	0		hypothetical protein CDS	hypothetical protein	None	KLI70043.1
Variants: VaphiSt2_B_FDSW210055056-1r	GGT	485	487	2	CGC -> GGT	1323 -> 1354	Substitution	33.2% -> 34.0%	0	A -> T	hypothetical protein CDS	hypothetical protein	Substitution	KLI70043.1
Variants: VaphiSt2_B_FDSW210055056-1r	A	497	497	1	G -> A	1496	SNP (transition)	33.00%	0		hypothetical protein CDS	hypothetical protein	None	KLI70043.1
Variants: VaphiSt2_B_FDSW210055056-1r	C	509	509	1	G -> C	1645	SNP (transversion)	53.20%	0		hypothetical protein CDS	hypothetical protein	None	KLI70043.1
Variants: VaphiSt2_B_FDSW210055056-1r	G	530	530	1	C -> G	1921	SNP (transversion)	35.60%	0		hypothetical protein CDS	hypothetical protein	None	KLI70043.1
Variants: VaphiSt2_B_FDSW210055056-1r	A	563	563	1	G -> A	2067	SNP (transition)	36.20%	0		hypothetical protein CDS	hypothetical protein	None	KLI70043.1
Variants: VaphiSt2_B_FDSW210055056-1r	GG	593	594	2	CA -> GG	2202 -> 2203	Substitution	25.00%	0	V -> A	hypothetical protein CDS	hypothetical protein	Substitution	KLI70043.1
Variants: VaphiSt2_B_FDSW210055056-1r	A	608	608	1	G -> A	2249	SNP (transition)	43.80%	0		hypothetical protein CDS	hypothetical protein	None	KLI70043.1
Variants: VaphiSt2_B_FDSW210055056-1r	G	614	614	1	A -> G	2316	SNP (transition)	35.60%	0		hypothetical protein CDS	hypothetical protein	None	KLI70043.1
Variants: VaphiSt2_B_FDSW210055056-1r	T	623	623	1	C -> T	2358	SNP (transition)	28.00%	0		hypothetical protein CDS	hypothetical protein	None	KLI70043.1
Variants: VaphiSt2_B_FDSW210055056-1r	C	628	628	1	T -> C	2351	SNP (transition)	44.00%	0	N -> D	hypothetical protein CDS	hypothetical protein	Substitution	KLI70043.1
Variants: VaphiSt2_B_FDSW210055056-1r	A	644	644	1	G -> A	2297	SNP (transition)	26.20%	0		hypothetical protein CDS	hypothetical protein	None	KLI70043.1
Variants: VaphiSt2_B_FDSW210055056-1r	G	671	671	1	A -> G	2161	SNP (transition)	30.30%	0		hypothetical protein CDS	hypothetical protein	None	KLI70043.1
Variants: VaphiSt2_B_FDSW210055056-1r	T	675	675	1	C -> T	2139	SNP (transition)	30.20%	0	S -> N	hypothetical protein CDS	hypothetical protein	Substitution	KLI70043.1
Variants: VaphiSt2_B_FDSW210055056-1r	A	677	677	1	G -> A	2107	SNP (transition)	39.60%	0		hypothetical protein CDS	hypothetical protein	None	KLI70043.1

Variants: VaphiSt2_B_FDSW210055056-1r	A	722	722	1	G -> A	1844	SNP (transition)	29.00%	0		hypothetical protein CDS	hypothetical protein	None	KLI700 43.1
Variants: VaphiSt2_B_FDSW210055056-1r	T	728	728	1	C -> T	1788	SNP (transition)	25.40%	0		hypothetical protein CDS	hypothetical protein	None	KLI700 43.1
Variants: VaphiSt2_B_FDSW210055056-1r	A	737	737	1	G -> A	1695	SNP (transition)	71.50%	0		hypothetical protein CDS	hypothetical protein	None	KLI700 43.1
Variants: VaphiSt2_B_FDSW210055056-1r	A	745	745	1	C -> A	1608	SNP (transversion)	27.90%	0	A -> S	hypothetical protein CDS	hypothetical protein	Substituti on	KLI700 43.1
Variants: VaphiSt2_B_FDSW210055056-1r	C	752	752	1	G -> C	1555	SNP (transversion)	27.70%	0		hypothetical protein CDS	hypothetical protein	None	KLI700 43.1
Variants: VaphiSt2_B_FDSW210055056-1r	A	755	755	1	C -> A	1518	SNP (transversion)	34.80%	0		hypothetical protein CDS	hypothetical protein	None	KLI700 43.1
Variants: VaphiSt2_B_FDSW210055056-1r	A	767	767	1	G -> A	1343	SNP (transition)	27.30%	0		hypothetical protein CDS	hypothetical protein	None	KLI700 43.1
Variants: VaphiSt2_B_FDSW210055056-1r	G	806	806	1	C -> G	1121	SNP (transversion)	77.40%	0		hypothetical protein CDS	hypothetical protein	None	KLI700 43.1
Variants: VaphiSt2_B_FDSW210055056-1r	C	823	823	1	T -> C	1089	SNP (transition)	62.50%	0	I -> V	hypothetical protein CDS	hypothetical protein	Substituti on	KLI700 43.1
Variants: VaphiSt2_B_FDSW210055056-1r	T	834	834	1	G -> T	1097	SNP (transversion)	58.80%	0	T -> K	hypothetical protein CDS	hypothetical protein	Substituti on	KLI700 43.1
Variants: VaphiSt2_B_FDSW210055056-1r	C	844	844	1	T -> C	1041	SNP (transition)	77.20%	0	T -> A	hypothetical protein CDS	hypothetical protein	Substituti on	KLI700 43.1
Variants: VaphiSt2_B_FDSW210055056-1r	A	854	854	1	G -> A	1051	SNP (transition)	67.60%	0		hypothetical protein CDS	hypothetical protein	None	KLI700 43.1
Variants: VaphiSt2_B_FDSW210055056-1r	CA	865	866	2	GG -> CA	1076 -> 1082	Substitution	32.7% -> 33.0%	0	IQ -> IE	hypothetical protein CDS	hypothetical protein	Substituti on	KLI700 43.1
Variants: VaphiSt2_B_FDSW210055056-1r	A	874	874	1	G -> A	1098	SNP (transition)	27.00%	0		hypothetical protein CDS	hypothetical protein	None	KLI700 43.1
Variants: VaphiSt2_B_FDSW210055056-1r	A	881	881	1	G -> A	1099	SNP (transition)	50.40%	0		hypothetical protein CDS	hypothetical protein	None	KLI700 43.1
Variants: VaphiSt2_B_FDSW210055056-1r	A	887	887	1	G -> A	1109	SNP (transition)	83.90%	0		hypothetical protein CDS	hypothetical protein	None	KLI700 43.1
Variants: VaphiSt2_B_FDSW210055056-1r	A	890	890	1	G -> A	1118	SNP (transition)	27.40%	0		hypothetical protein CDS	hypothetical protein	None	KLI700 43.1
Variants: VaphiSt2_B_FDSW210055056-1r	G	896	896	1	A -> G	1116	SNP (transition)	83.60%	0		hypothetical protein CDS	hypothetical protein	None	KLI700 43.1
Variants: VaphiSt2_B_FDSW210055056-1r	G	908	908	1	A -> G	1152	SNP (transition)	32.60%	0		hypothetical protein CDS	hypothetical protein	None	KLI700 43.1
Variants: VaphiSt2_B_FDSW210055056-1r	GA C	941	943	2	AAT -> GAC	1257 -> 1266	Substitution	27.5% -> 27.9%	0	I -> V	hypothetical protein CDS	hypothetical protein	Substituti on	KLI700 43.1
Variants: VaphiSt2_B_FDSW210055056-1r	C	943	943	1	T -> C	1266	SNP (transition)	58.10%	0	I -> V	hypothetical protein CDS	hypothetical protein	Substituti on	KLI700 43.1
Variants: VaphiSt2_B_FDSW210055056-1r	A	944	944	1	C -> A	1271	SNP (transversion)	53.60%	0		hypothetical protein CDS	hypothetical protein	None	KLI700 43.1
Variants: VaphiSt2_B_FDSW210055056-1r	G	944	944	1	C -> G	1271	SNP (transversion)	32.30%	0		hypothetical protein CDS	hypothetical protein	None	KLI700 43.1
Variants: VaphiSt2_B_FDSW210055056-1r	G	947	947	1	C -> G	1282	SNP (transversion)	53.10%	0		hypothetical protein CDS	hypothetical protein	None	KLI700 43.1
Variants: VaphiSt2_B_FDSW210055056-1r	AT	947	948	2	CG -> AT	1282 -> 1289	Substitution	32.0% -> 32.2%	0	T -> N	hypothetical protein CDS	hypothetical protein	Substituti on	KLI700 43.1
Variants: VaphiSt2_B_FDSW210055056-1r	A	950	950	1	G -> A	1292	SNP (transition)	51.90%	0		hypothetical protein CDS	hypothetical protein	None	KLI700 43.1
Variants: VaphiSt2_B_FDSW210055056-1r	CT T	955	957	3	TGG -> CTT	1298 -> 1299	Substitution	50.9% -> 51.2%	0	TN -> KD	hypothetical protein CDS	hypothetical protein	Substituti on	KLI700 43.1
Variants: VaphiSt2_B_FDSW210055056-1r	T	959	959	1	C -> T	1302	SNP (transition)	50.80%	0		hypothetical protein CDS	hypothetical protein	None	KLI700 43.1
Variants: VaphiSt2_B_FDSW210055056-1r	G	971	971	1	A -> G	1332	SNP (transition)	38.30%	0		hypothetical protein CDS	hypothetical protein	None	KLI700 43.1
Variants: VaphiSt2_B_FDSW210055056-1r	G	974	974	1	A -> G	1345	SNP (transition)	26.20%	0		hypothetical protein CDS	hypothetical protein	None	KLI700 43.1
Variants: VaphiSt2_B_FDSW210055056-1r	C	977	977	1	G -> C	1350	SNP (transversion)	49.00%	0		hypothetical protein CDS	hypothetical protein	None	KLI700 43.1
Variants: VaphiSt2_B_FDSW210055056-1r	C	983	983	1	T -> C	1344	SNP (transition)	70.90%	0		hypothetical protein CDS	hypothetical protein	None	KLI700 43.1

Variants: VaphiSt2_B_FDSW210055056-1r	C	986	986	1	G -> C	1365	SNP (transversion)	70.80%	0		hypothetical protein CDS	hypothetical protein	None	KLI70043.1
Variants: VaphiSt2_B_FDSW210055056-1r	T	1013	1013	1	C -> T	1449	SNP (transition)	34.80%	0		hypothetical protein CDS	hypothetical protein	None	KLI70043.1
Variants: VaphiSt2_B_FDSW210055056-1r	G	1025	1025	1	C -> G	1455	SNP (transversion)	41.00%	0		hypothetical protein CDS	hypothetical protein	None	KLI70043.1
Variants: VaphiSt2_B_FDSW210055056-1r	T	1025	1025	1	C -> T	1455	SNP (transition)	38.00%	0		hypothetical protein CDS	hypothetical protein	None	KLI70043.1
Variants: VaphiSt2_B_FDSW210055056-1r	G	1028	1028	1	C -> G	1488	SNP (transversion)	27.10%	0		hypothetical protein CDS	hypothetical protein	None	KLI70043.1
Variants: VaphiSt2_B_FDSW210055056-1r	T	1054	1054	1	C -> T	1578	SNP (transition)	62.70%	0	A -> T	hypothetical protein CDS	hypothetical protein	Substitution	KLI70043.1
Variants: VaphiSt2_B_FDSW210055056-1r	A	1055	1055	1	C -> A	1573	SNP (transversion)	31.40%	0		hypothetical protein CDS	hypothetical protein	None	KLI70043.1
Variants: VaphiSt2_B_FDSW210055056-1r	G	1055	1055	1	C -> G	1573	SNP (transversion)	44.50%	0		hypothetical protein CDS	hypothetical protein	None	KLI70043.1
Variants: VaphiSt2_B_FDSW210055056-1r	A	1062	1062	1	T -> A	1577	SNP (transversion)	57.30%	0	Y -> F	hypothetical protein CDS	hypothetical protein	Substitution	KLI70043.1

Contig 52

Variant	Name	Minimum	Maximum	Length	Change	Coverage	Polymorphism Type	Variant Frequency	Variant P-Value (approximate)
Variants: VaAphrodite_B_FDSW210055058-2r	T	18	18	1	C -> T	180	SNP (transition)	32.80%	2.60E-141
Variants: VaAphrodite_B_FDSW210055058-2r	G	25	25	1	A -> G	183	SNP (transition)	31.70%	6.40E-138
Variants: VaAphrodite_B_FDSW210055058-2r	AG	35	36	2	GA -> AG	168 -> 169	Substitution	29.6% -> 29.8%	2.30E-117
Variants: VaAphrodite_C_FDSW210055059-1r	T	18	18	1	C -> T	235	SNP (transition)	31.10%	5.70E-187
Variants: VaAphrodite_C_FDSW210055059-1r	G	25	25	1	A -> G	233	SNP (transition)	28.80%	2.20E-162
Variants: VaAres1_A_FDSW210055060-2r	T	18	18	1	C -> T	109	SNP (transition)	26.60%	2.70E-64
Variants: VaAres1_A_FDSW210055060-2r	G	25	25	1	A -> G	109	SNP (transition)	26.60%	2.70E-64
Variants: VaAres1_B_FDSW210055061-2r	A	1519	1519	1	C -> A	56	SNP (transversion)	28.60%	2.60E-46
Variants: VaAres1_B_FDSW210055061-2r	G	1520	1520	1	A -> G	74	SNP (transition)	27.00%	5.80E-53
Variants: VaAres1_B_FDSW210055061-2r	G	1521	1521	1	C -> G	94	SNP (transversion)	25.50%	5.70E-65
Variants: VaAres1_C_FDSW210055062-1r	T	18	18	1	C -> T	173	SNP (transition)	27.20%	4.50E-113
Variants: VaAres1_C_FDSW210055062-1r	G	25	25	1	A -> G	178	SNP (transition)	25.80%	1.50E-109
Variants: VaAres1_C_FDSW210055062-1r	A	35	35	1	G -> A	170	SNP (transition)	25.30%	4.70E-102
Variants: VaphiVaphiSt2_B_A_FDSW210055054-2r	T	18	18	1	C -> T	143	SNP (transition)	25.20%	5.00E-82
Variants: VaphiSt2_B_FDSW210055056-1r	T	18	18	1	C -> T	262	SNP (transition)	27.50%	6.50E-180
Variants: VaphiSt2_B_FDSW210055056-1r	G	25	25	1	A -> G	262	SNP (transition)	26.70%	5.60E-167

Contig 53

Variant	Name	Minimum	Maximum	Length	Change	Coverage	Polymorphism Type	Variant Frequency	Variant P-Value (approximate)
Variants: VaAphrodite_A_FDSW210055057-2r	A	36	36	1	G -> A	2079	SNP (transition)	26.20%	0
Variants: VaAphrodite_A_FDSW210055057-2r	T	856	856	1	A -> T	3958	SNP (transversion)	25.80%	0
Variants: VaAphrodite_A_FDSW210055057-2r	C	1196	1196	1	A -> C	3835	SNP (transversion)	49.80%	0
Variants: VaAphrodite_A_FDSW210055057-2r		2519	2519	1	(A)3 -> (A)2	3773	Deletion (tandem repeat)	25.00%	0
Variants: VaAphrodite_A_FDSW210055057-2r	A	2748	2748	1	C -> A	3679	SNP (transversion)	30.90%	0
Variants: VaAphrodite_A_FDSW210055057-2r	A	2757	2757	1	G -> A	3649	SNP (transition)	30.70%	0
Variants: VaAphrodite_A_FDSW210055057-2r	C	2759	2759	1	T -> C	3650	SNP (transition)	30.80%	0
Variants: VaAphrodite_B_FDSW210055058-2r	A	36	36	1	G -> A	1555	SNP (transition)	31.70%	0
Variants: VaAphrodite_B_FDSW210055058-2r	T	856	856	1	A -> T	2920	SNP (transversion)	25.50%	0
Variants: VaAphrodite_B_FDSW210055058-2r	C	1196	1196	1	A -> C	2826	SNP (transversion)	49.90%	0
Variants: VaAphrodite_B_FDSW210055058-2r	A	2748	2748	1	C -> A	2843	SNP (transversion)	32.10%	0
Variants: VaAphrodite_B_FDSW210055058-2r	A	2757	2757	1	G -> A	2844	SNP (transition)	32.10%	0
Variants: VaAphrodite_B_FDSW210055058-2r	C	2759	2759	1	T -> C	2849	SNP (transition)	32.10%	0
Variants: VaAphrodite_C_FDSW210055059-1r	T	856	856	1	A -> T	3639	SNP (transversion)	25.40%	0
Variants: VaAphrodite_C_FDSW210055059-1r	C	1196	1196	1	A -> C	3480	SNP (transversion)	50.30%	0
Variants: VaAphrodite_C_FDSW210055059-1r	A	2748	2748	1	C -> A	3688	SNP (transversion)	31.10%	0
Variants: VaAphrodite_C_FDSW210055059-1r	A	2757	2757	1	G -> A	3661	SNP (transition)	31.30%	0
Variants: VaAphrodite_C_FDSW210055059-1r	C	2759	2759	1	T -> C	3647	SNP (transition)	31.10%	0
Variants: VaAres1_A_FDSW210055060-2r	A	36	36	1	G -> A	1223	SNP (transition)	30.10%	0
Variants: VaAres1_A_FDSW210055060-2r	C	1196	1196	1	A -> C	2336	SNP (transversion)	47.80%	0
Variants: VaAres1_A_FDSW210055060-2r	A	2748	2748	1	C -> A	2434	SNP (transversion)	31.70%	0
Variants: VaAres1_A_FDSW210055060-2r	A	2757	2757	1	G -> A	2442	SNP (transition)	30.80%	0
Variants: VaAres1_A_FDSW210055060-2r	C	2759	2759	1	T -> C	2440	SNP (transition)	30.80%	0
Variants: VaAres1_B_FDSW210055061-2r	A	36	36	1	G -> A	1771	SNP (transition)	25.60%	0
Variants: VaAres1_B_FDSW210055061-2r	T	856	856	1	A -> T	3122	SNP (transversion)	25.70%	0
Variants: VaAres1_B_FDSW210055061-2r	C	1196	1196	1	A -> C	3354	SNP (transversion)	47.80%	0
Variants: VaAres1_B_FDSW210055061-2r	A	2748	2748	1	C -> A	3046	SNP (transversion)	31.30%	0
Variants: VaAres1_B_FDSW210055061-2r	A	2757	2757	1	G -> A	2968	SNP (transition)	30.20%	0
Variants: VaAres1_B_FDSW210055061-2r	C	2759	2759	1	T -> C	2948	SNP (transition)	30.40%	0

Variants: VaAres1_C_FDSW210055062-1r	A	36	36	1	G -> A	2038	SNP (transition)	28.20%	0
Variants: VaAres1_C_FDSW210055062-1r	T	856	856	1	A -> T	4120	SNP (transversion)	27.10%	0
Variants: VaAres1_C_FDSW210055062-1r	C	1196	1196	1	A -> C	3833	SNP (transversion)	52.10%	0
Variants: VaAres1_C_FDSW210055062-1r	A	2748	2748	1	C -> A	3752	SNP (transversion)	33.20%	0
Variants: VaAres1_C_FDSW210055062-1r	A	2757	2757	1	G -> A	3723	SNP (transition)	33.00%	0
Variants: VaAres1_C_FDSW210055062-1r	C	2759	2759	1	T -> C	3734	SNP (transition)	32.90%	0
Variants: VaphiVaphiSt2_B_A_FDSW210055054-2r	A	36	36	1	G -> A	1134	SNP (transition)	27.60%	0
Variants: VaphiVaphiSt2_B_A_FDSW210055054-2r	T	856	856	1	A -> T	2187	SNP (transversion)	25.50%	0
Variants: VaphiVaphiSt2_B_A_FDSW210055054-2r	C	1196	1196	1	A -> C	2260	SNP (transversion)	46.90%	0
Variants: VaphiVaphiSt2_B_A_FDSW210055054-2r	A	2748	2748	1	C -> A	2039	SNP (transversion)	28.80%	0
Variants: VaphiVaphiSt2_B_A_FDSW210055054-2r	A	2757	2757	1	G -> A	2009	SNP (transition)	28.70%	0
Variants: VaphiVaphiSt2_B_A_FDSW210055054-2r	C	2759	2759	1	T -> C	2004	SNP (transition)	28.60%	0
Variants: VaphiSt2_B_FDSW210055056-1r	A	36	36	1	G -> A	2116	SNP (transition)	31.80%	0
Variants: VaphiSt2_B_FDSW210055056-1r	C	1196	1196	1	A -> C	3860	SNP (transversion)	48.20%	0
Variants: VaphiSt2_B_FDSW210055056-1r	A	2748	2748	1	C -> A	4007	SNP (transversion)	30.70%	0
Variants: VaphiSt2_B_FDSW210055056-1r	A	2757	2757	1	G -> A	3993	SNP (transition)	30.90%	0
Variants: VaphiSt2_B_FDSW210055056-1r	C	2759	2759	1	T -> C	4008	SNP (transition)	30.90%	0

Contig 54

Variant	Name	Minimum	Maximum	Length	Change	Coverage	Polymorphism Type	Variant Frequency	Variant P-Value (approximate)
Variants: VaAphrodite_A_FDSW210055057-2r	C	38	38	1	T -> C	1399	SNP (transition)	27.10%	0
Variants: VaAphrodite_A_FDSW210055057-2r	G	61	61	1	A -> G	1641	SNP (transition)	28.30%	0
Variants: VaAphrodite_A_FDSW210055057-2r	T	72	72	1	C -> T	1803	SNP (transition)	27.60%	0
Variants: VaAphrodite_A_FDSW210055057-2r	G	1153	1153	1	A -> G	3861	SNP (transition)	32.00%	0
Variants: VaAphrodite_A_FDSW210055057-2r	T	1172	1172	1	C -> T	3877	SNP (transition)	36.50%	0
Variants: VaAphrodite_A_FDSW210055057-2r	C	1173	1173	1	T -> C	3886	SNP (transition)	39.70%	0
Variants: VaAphrodite_A_FDSW210055057-2r	G	1550	1550	1	A -> G	3360	SNP (transition)	28.40%	0
Variants: VaAphrodite_A_FDSW210055057-2r	A	1679	1678	0	(A)2 -> (A)3	2389	Insertion (tandem repeat)	46.20%	0
Variants: VaAphrodite_A_FDSW210055057-2r	CGAA	1707	1710	4	TTTC -> CGAA	2038 -> 2081	Substitution	33.9% -> 34.7%	0
Variants: VaAphrodite_B_FDSW210055058-2r	C	38	38	1	T -> C	1137	SNP (transition)	27.40%	0
Variants: VaAphrodite_B_FDSW210055058-2r	A	46	46	1	G -> A	1241	SNP (transition)	26.10%	0
Variants: VaAphrodite_B_FDSW210055058-2r	G	61	61	1	A -> G	1477	SNP (transition)	32.80%	0
Variants: VaAphrodite_B_FDSW210055058-2r	T	72	72	1	C -> T	1573	SNP (transition)	31.20%	0
Variants: VaAphrodite_B_FDSW210055058-2r	G	1153	1153	1	A -> G	2982	SNP (transition)	30.00%	0
Variants: VaAphrodite_B_FDSW210055058-2r	T	1172	1172	1	C -> T	2962	SNP (transition)	36.50%	0
Variants: VaAphrodite_B_FDSW210055058-2r	C	1173	1173	1	T -> C	2977	SNP (transition)	38.80%	0
Variants: VaAphrodite_B_FDSW210055058-2r	G	1550	1550	1	A -> G	2753	SNP (transition)	25.50%	0
Variants: VaAphrodite_B_FDSW210055058-2r	A	1679	1678	0	(A)2 -> (A)3	2058	Insertion (tandem repeat)	48.10%	0
Variants: VaAphrodite_B_FDSW210055058-2r	CGAA	1707	1710	4	TTTC -> CGAA	1737 -> 1783	Substitution	36.6% -> 37.2%	0
Variants: VaAphrodite_C_FDSW210055059-1r	C	38	38	1	T -> C	1411	SNP (transition)	25.70%	0
Variants: VaAphrodite_C_FDSW210055059-1r	A	46	46	1	G -> A	1480	SNP (transition)	28.90%	0
Variants: VaAphrodite_C_FDSW210055059-1r	G	61	61	1	A -> G	1755	SNP (transition)	34.60%	0
Variants: VaAphrodite_C_FDSW210055059-1r	T	72	72	1	C -> T	1903	SNP (transition)	33.70%	0
Variants: VaAphrodite_C_FDSW210055059-1r	G	1153	1153	1	A -> G	3637	SNP (transition)	31.50%	0
Variants: VaAphrodite_C_FDSW210055059-1r	T	1172	1172	1	C -> T	3679	SNP (transition)	35.50%	0
Variants: VaAphrodite_C_FDSW210055059-1r	C	1173	1173	1	T -> C	3671	SNP (transition)	39.00%	0
Variants: VaAphrodite_C_FDSW210055059-1r	G	1550	1550	1	A -> G	3445	SNP (transition)	27.60%	0
Variants: VaAphrodite_C_FDSW210055059-1r	A	1679	1678	0	(A)2 -> (A)3	2599	Insertion (tandem repeat)	46.70%	0
Variants: VaAphrodite_C_FDSW210055059-1r	CGAA	1707	1710	4	TTTC -> CGAA	2086 -> 2127	Substitution	34.7% -> 35.2%	0

Variants: VaAres1_A_FDSW210055060-2r	C	38	38	1	T -> C	880	SNP (transition)	25.70%	0
Variants: VaAres1_A_FDSW210055060-2r	G	61	61	1	A -> G	1064	SNP (transition)	30.30%	0
Variants: VaAres1_A_FDSW210055060-2r	T	72	72	1	C -> T	1151	SNP (transition)	29.50%	0
Variants: VaAres1_A_FDSW210055060-2r	G	1153	1153	1	A -> G	2445	SNP (transition)	31.80%	0
Variants: VaAres1_A_FDSW210055060-2r	T	1172	1172	1	C -> T	2428	SNP (transition)	34.40%	0
Variants: VaAres1_A_FDSW210055060-2r	C	1173	1173	1	T -> C	2435	SNP (transition)	40.70%	0
Variants: VaAres1_A_FDSW210055060-2r	G	1550	1550	1	A -> G	2065	SNP (transition)	26.00%	0
Variants: VaAres1_A_FDSW210055060-2r	A	1679	1678	0	(A)2 -> (A)3	1489	Insertion (tandem repeat)	42.80%	0
Variants: VaAres1_A_FDSW210055060-2r	CGAA	1707	1710	4	TTTC -> CGAA	1312 -> 1333	Substitution	34.4% -> 35.0%	0
Variants: VaAres1_B_FDSW210055061-2r	G	61	61	1	A -> G	1310	SNP (transition)	29.40%	0
Variants: VaAres1_B_FDSW210055061-2r	T	72	72	1	C -> T	1385	SNP (transition)	29.20%	0
Variants: VaAres1_B_FDSW210055061-2r	G	1153	1153	1	A -> G	3109	SNP (transition)	30.60%	0
Variants: VaAres1_B_FDSW210055061-2r	T	1172	1172	1	C -> T	3098	SNP (transition)	36.10%	0
Variants: VaAres1_B_FDSW210055061-2r	C	1173	1173	1	T -> C	3106	SNP (transition)	41.40%	0
Variants: VaAres1_B_FDSW210055061-2r	G	1550	1550	1	A -> G	2425	SNP (transition)	25.20%	0
Variants: VaAres1_B_FDSW210055061-2r	A	1679	1678	0	(A)2 -> (A)3	1828	Insertion (tandem repeat)	44.70%	0
Variants: VaAres1_B_FDSW210055061-2r	CGAA	1707	1710	4	TTTC -> CGAA	1651 -> 1677	Substitution	31.6% -> 32.8%	0
Variants: VaAres1_C_FDSW210055062-1r	G	61	61	1	A -> G	1793	SNP (transition)	30.50%	0
Variants: VaAres1_C_FDSW210055062-1r	T	72	72	1	C -> T	1934	SNP (transition)	29.60%	0
Variants: VaAres1_C_FDSW210055062-1r	G	1153	1153	1	A -> G	4064	SNP (transition)	31.40%	0
Variants: VaAres1_C_FDSW210055062-1r	T	1172	1172	1	C -> T	4067	SNP (transition)	33.30%	0
Variants: VaAres1_C_FDSW210055062-1r	C	1173	1173	1	T -> C	4077	SNP (transition)	40.10%	0
Variants: VaAres1_C_FDSW210055062-1r	G	1550	1550	1	A -> G	3380	SNP (transition)	26.90%	0
Variants: VaAres1_C_FDSW210055062-1r	A	1679	1678	0	(A)2 -> (A)3	2261	Insertion (tandem repeat)	45.90%	0
Variants: VaAres1_C_FDSW210055062-1r	CGAA	1707	1710	4	TTTC -> CGAA	1938 -> 1990	Substitution	33.7% -> 34.5%	0
Variants: VaphiVaphiSt2_B_A_FDSW210055054-2r	C	38	38	1	T -> C	754	SNP (transition)	25.20%	0
Variants: VaphiVaphiSt2_B_A_FDSW210055054-2r	A	46	46	1	G -> A	815	SNP (transition)	25.60%	0
Variants: VaphiVaphiSt2_B_A_FDSW210055054-2r	G	61	61	1	A -> G	916	SNP (transition)	33.10%	0
Variants: VaphiVaphiSt2_B_A_FDSW210055054-2r	T	72	72	1	C -> T	1002	SNP (transition)	32.30%	0
Variants: VaphiVaphiSt2_B_A_FDSW210055054-2r	G	1153	1153	1	A -> G	2272	SNP (transition)	32.20%	0
Variants: VaphiVaphiSt2_B_A_FDSW210055054-2r	T	1172	1172	1	C -> T	2260	SNP (transition)	35.00%	0
Variants: VaphiVaphiSt2_B_A_FDSW210055054-2r	C	1173	1173	1	T -> C	2268	SNP (transition)	41.30%	0

Variants: VaphiVaphiSt2_B_A_FDSW210055054-2r	G	1550	1550	1	A -> G	1916	SNP (transition)	25.50%	0
Variants: VaphiVaphiSt2_B_A_FDSW210055054-2r	A	1679	1678	0	(A)2 -> (A)3	1372	Insertion (tandem repeat)	45.30%	0
Variants: VaphiVaphiSt2_B_A_FDSW210055054-2r	CGAA	1707	1710	4	TTTC -> CGAA	1189 -> 1203	Substitution	33.1% -> 33.5%	0
Variants: VaphiSt2_B_FDSW210055056-1r	C	38	38	1	T -> C	1557	SNP (transition)	27.10%	0
Variants: VaphiSt2_B_FDSW210055056-1r	A	46	46	1	G -> A	1706	SNP (transition)	26.70%	0
Variants: VaphiSt2_B_FDSW210055056-1r	G	61	61	1	A -> G	1982	SNP (transition)	33.10%	0
Variants: VaphiSt2_B_FDSW210055056-1r	T	72	72	1	C -> T	2178	SNP (transition)	31.30%	0
Variants: VaphiSt2_B_FDSW210055056-1r	G	1153	1153	1	A -> G	4230	SNP (transition)	30.60%	0
Variants: VaphiSt2_B_FDSW210055056-1r	T	1172	1172	1	C -> T	4223	SNP (transition)	37.10%	0
Variants: VaphiSt2_B_FDSW210055056-1r	C	1173	1173	1	T -> C	4226	SNP (transition)	39.70%	0
Variants: VaphiSt2_B_FDSW210055056-1r	G	1550	1550	1	A -> G	3845	SNP (transition)	26.60%	0
Variants: VaphiSt2_B_FDSW210055056-1r	A	1679	1678	0	(A)2 -> (A)3	2638	Insertion (tandem repeat)	46.10%	0
Variants: VaphiSt2_B_FDSW210055056-1r	CGAA	1707	1710	4	TTTC -> CGAA	2226 -> 2270	Substitution	32.6% -> 33.3%	0

Contig 57

Variant	Name	Minimum	Maximum	Length	Change	Coverage	Polymorphism Type	Variant Frequency	Variant P-Value (approximate)	Amino Acid Change	CDS	product	Protein Effect	protein_id
Variants: VaAphrodite_A_FDSW210055057-2r	G	159	159	1	T -> G	283	SNP (transversion)	44.90%	0		RTX toxin CDS	RTX toxin	None	KLI69968.1
Variants: VaAphrodite_A_FDSW210055057-2r	G	173	173	1	A -> G	295	SNP (transition)	45.40%	0	D -> G	RTX toxin CDS	RTX toxin	Substitution	KLI69968.1
Variants: VaAphrodite_A_FDSW210055057-2r	A	201	201	1	G -> A	304	SNP (transition)	53.60%	0		RTX toxin CDS	RTX toxin	None	KLI69968.1
Variants: VaAphrodite_A_FDSW210055057-2r	G	363	363	1	A -> G	292	SNP (transition)	56.20%	0		RTX toxin CDS	RTX toxin	None	KLI69968.1
Variants: VaAphrodite_A_FDSW210055057-2r	G	427	427	1	A -> G	288	SNP (transition)	53.50%	0	I -> V	RTX toxin CDS	RTX toxin	Substitution	KLI69968.1
Variants: VaAphrodite_A_FDSW210055057-2r	C	437	437	1	T -> C	294	SNP (transition)	53.70%	0	L -> S	RTX toxin CDS	RTX toxin	Substitution	KLI69968.1
Variants: VaAphrodite_A_FDSW210055057-2r	G	723	723	1	A -> G	296	SNP (transition)	51.00%	0		RTX toxin CDS	RTX toxin	None	KLI69968.1
Variants: VaAphrodite_A_FDSW210055057-2r	G	728	728	1	T -> G	295	SNP (transversion)	51.50%	0	V -> G	RTX toxin CDS	RTX toxin	Substitution	KLI69968.1
Variants: VaAphrodite_A_FDSW210055057-2r	A	745	745	1	G -> A	285	SNP (transition)	50.90%	0	A -> T	RTX toxin CDS	RTX toxin	Substitution	KLI69968.1
Variants: VaAphrodite_A_FDSW210055057-2r	A	840	840	1	G -> A	282	SNP (transition)	52.10%	0		RTX toxin CDS	RTX toxin	None	KLI69968.1
Variants: VaAphrodite_A_FDSW210055057-2r	C	986	986	1	T -> C	278	SNP (transition)	46.80%	0	I -> T	RTX toxin CDS	RTX toxin	Substitution	KLI69968.1
Variants: VaAphrodite_A_FDSW210055057-2r	T	1019	1019	1	C -> T	259	SNP (transition)	46.70%	0	T -> M	RTX toxin CDS	RTX toxin	Substitution	KLI69968.1
Variants: VaAphrodite_A_FDSW210055057-2r	C	1122	1122	1	A -> C	258	SNP (transversion)	56.60%	0		RTX toxin CDS	RTX toxin	None	KLI69968.1
Variants: VaAphrodite_A_FDSW210055057-2r	A	1159	1159	1	G -> A	261	SNP (transition)	54.40%	0	A -> T	RTX toxin CDS	RTX toxin	Substitution	KLI69968.1
Variants: VaAphrodite_A_FDSW210055057-2r	G	1191	1191	1	A -> G	258	SNP (transition)	51.20%	0		RTX toxin CDS	RTX toxin	None	KLI69968.1
Variants: VaAphrodite_A_FDSW210055057-2r	T	1323	1323	1	C -> T	250	SNP (transition)	54.00%	0		RTX toxin CDS	RTX toxin	None	KLI69968.1
Variants: VaAphrodite_A_FDSW210055057-2r	C	1353	1353	1	T -> C	232	SNP (transition)	55.20%	0		RTX toxin CDS	RTX toxin	None	KLI69968.1
Variants: VaAphrodite_A_FDSW210055057-2r	T	1418	1418	1	C -> T	223	SNP (transition)	39.50%	########	S -> L	RTX toxin CDS	RTX toxin	Substitution	KLI69968.1
Variants: VaAphrodite_A_FDSW210055057-2r	G	1425	1425	1	A -> G	216	SNP (transition)	37.00%	########		RTX toxin CDS	RTX toxin	None	KLI69968.1
Variants: VaAphrodite_B_FDSW210055058-2r	G	159	159	1	T -> G	227	SNP (transversion)	44.90%	########		RTX toxin CDS	RTX toxin	None	KLI69968.1
Variants: VaAphrodite_B_FDSW210055058-2r	G	173	173	1	A -> G	238	SNP (transition)	45.40%	0.00E+00	D -> G	RTX toxin CDS	RTX toxin	Substitution	KLI69968.1
Variants: VaAphrodite_B_FDSW210055058-2r	A	201	201	1	G -> A	247	SNP (transition)	53.40%	0		RTX toxin CDS	RTX toxin	None	KLI69968.1
Variants: VaAphrodite_B_FDSW210055058-2r	G	363	363	1	A -> G	279	SNP (transition)	45.50%	0		RTX toxin CDS	RTX toxin	None	KLI69968.1
Variants: VaAphrodite_B_FDSW210055058-2r	G	427	427	1	A -> G	278	SNP (transition)	50.70%	0	I -> V	RTX toxin CDS	RTX toxin	Substitution	KLI69968.1
Variants: VaAphrodite_B_FDSW210055058-2r	C	437	437	1	T -> C	282	SNP (transition)	50.40%	0	L -> S	RTX toxin CDS	RTX toxin	Substitution	KLI69968.1
Variants: VaAphrodite_B_FDSW210055058-2r	G	723	723	1	A -> G	237	SNP (transition)	53.20%	0		RTX toxin CDS	RTX toxin	None	KLI69968.1
Variants: VaAphrodite_B_FDSW210055058-2r	G	728	728	1	T -> G	233	SNP (transversion)	52.40%	0	V -> G	RTX toxin CDS	RTX toxin	Substitution	KLI69968.1
Variants: VaAphrodite_B_FDSW210055058-2r	A	745	745	1	G -> A	240	SNP (transition)	54.20%	0	A -> T	RTX toxin CDS	RTX toxin	Substitution	KLI69968.1
Variants: VaAphrodite_B_FDSW210055058-2r	A	840	840	1	G -> A	200	SNP (transition)	55.00%	0		RTX toxin CDS	RTX toxin	None	KLI69968.1

Variants: VaAphrodite_B_FDSW210055058-2r	C	986	986	1	T -> C	221	SNP (transition)	42.10%	########	I -> T	RTX toxin CDS	RTX toxin	Substitution	KLI69968.1
Variants: VaAphrodite_B_FDSW210055058-2r	T	1019	1019	1	C -> T	228	SNP (transition)	42.10%	########	T -> M	RTX toxin CDS	RTX toxin	Substitution	KLI69968.1
Variants: VaAphrodite_B_FDSW210055058-2r	C	1122	1122	1	A -> C	249	SNP (transversion)	55.00%	0		RTX toxin CDS	RTX toxin	None	KLI69968.1
Variants: VaAphrodite_B_FDSW210055058-2r	A	1159	1159	1	G -> A	258	SNP (transition)	57.40%	0	A -> T	RTX toxin CDS	RTX toxin	Substitution	KLI69968.1
Variants: VaAphrodite_B_FDSW210055058-2r	G	1191	1191	1	A -> G	245	SNP (transition)	52.70%	0		RTX toxin CDS	RTX toxin	None	KLI69968.1
Variants: VaAphrodite_B_FDSW210055058-2r	T	1323	1323	1	C -> T	274	SNP (transition)	53.30%	0		RTX toxin CDS	RTX toxin	None	KLI69968.1
Variants: VaAphrodite_B_FDSW210055058-2r	C	1353	1353	1	T -> C	260	SNP (transition)	51.90%	0		RTX toxin CDS	RTX toxin	None	KLI69968.1
Variants: VaAphrodite_B_FDSW210055058-2r	T	1418	1418	1	C -> T	227	SNP (transition)	40.10%	########	S -> L	RTX toxin CDS	RTX toxin	Substitution	KLI69968.1
Variants: VaAphrodite_B_FDSW210055058-2r	G	1425	1425	1	A -> G	218	SNP (transition)	37.60%	########		RTX toxin CDS	RTX toxin	None	KLI69968.1
Variants: VaAphrodite_C_FDSW210055059-1r	G	159	159	1	T -> G	338	SNP (transversion)	51.20%	0		RTX toxin CDS	RTX toxin	None	KLI69968.1
Variants: VaAphrodite_C_FDSW210055059-1r	G	173	173	1	A -> G	333	SNP (transition)	51.40%	0	D -> G	RTX toxin CDS	RTX toxin	Substitution	KLI69968.1
Variants: VaAphrodite_C_FDSW210055059-1r	A	201	201	1	G -> A	334	SNP (transition)	47.60%	0		RTX toxin CDS	RTX toxin	None	KLI69968.1
Variants: VaAphrodite_C_FDSW210055059-1r	G	363	363	1	A -> G	392	SNP (transition)	49.70%	0		RTX toxin CDS	RTX toxin	None	KLI69968.1
Variants: VaAphrodite_C_FDSW210055059-1r	G	427	427	1	A -> G	384	SNP (transition)	48.20%	0	I -> V	RTX toxin CDS	RTX toxin	Substitution	KLI69968.1
Variants: VaAphrodite_C_FDSW210055059-1r	C	437	437	1	T -> C	390	SNP (transition)	47.70%	0	L -> S	RTX toxin CDS	RTX toxin	Substitution	KLI69968.1
Variants: VaAphrodite_C_FDSW210055059-1r	G	723	723	1	A -> G	397	SNP (transition)	51.40%	0		RTX toxin CDS	RTX toxin	None	KLI69968.1
Variants: VaAphrodite_C_FDSW210055059-1r	G	728	728	1	T -> G	391	SNP (transversion)	51.70%	0	V -> G	RTX toxin CDS	RTX toxin	Substitution	KLI69968.1
Variants: VaAphrodite_C_FDSW210055059-1r	A	745	745	1	G -> A	391	SNP (transition)	52.20%	0	A -> T	RTX toxin CDS	RTX toxin	Substitution	KLI69968.1
Variants: VaAphrodite_C_FDSW210055059-1r	A	840	840	1	G -> A	383	SNP (transition)	54.30%	0		RTX toxin CDS	RTX toxin	None	KLI69968.1
Variants: VaAphrodite_C_FDSW210055059-1r	C	986	986	1	T -> C	349	SNP (transition)	50.10%	0	I -> T	RTX toxin CDS	RTX toxin	Substitution	KLI69968.1
Variants: VaAphrodite_C_FDSW210055059-1r	T	1019	1019	1	C -> T	333	SNP (transition)	51.70%	0	T -> M	RTX toxin CDS	RTX toxin	Substitution	KLI69968.1
Variants: VaAphrodite_C_FDSW210055059-1r	C	1122	1122	1	A -> C	328	SNP (transversion)	47.00%	0		RTX toxin CDS	RTX toxin	None	KLI69968.1
Variants: VaAphrodite_C_FDSW210055059-1r	A	1159	1159	1	G -> A	348	SNP (transition)	47.70%	0	A -> T	RTX toxin CDS	RTX toxin	Substitution	KLI69968.1
Variants: VaAphrodite_C_FDSW210055059-1r	G	1191	1191	1	A -> G	368	SNP (transition)	45.70%	0		RTX toxin CDS	RTX toxin	None	KLI69968.1
Variants: VaAphrodite_C_FDSW210055059-1r	T	1323	1323	1	C -> T	360	SNP (transition)	53.90%	0		RTX toxin CDS	RTX toxin	None	KLI69968.1
Variants: VaAphrodite_C_FDSW210055059-1r	C	1353	1353	1	T -> C	365	SNP (transition)	52.10%	0		RTX toxin CDS	RTX toxin	None	KLI69968.1
Variants: VaAphrodite_C_FDSW210055059-1r	T	1418	1418	1	C -> T	322	SNP (transition)	42.90%	1.2E-320	S -> L	RTX toxin CDS	RTX toxin	Substitution	KLI69968.1
Variants: VaAphrodite_C_FDSW210055059-1r	G	1425	1425	1	A -> G	313	SNP (transition)	41.20%	########		RTX toxin CDS	RTX toxin	None	KLI69968.1
Variants: VaAres1_A_FDSW210055060-2r	G	159	159	1	T -> G	204	SNP (transversion)	47.10%	########		RTX toxin CDS	RTX toxin	None	KLI69968.1
Variants: VaAres1_A_FDSW210055060-2r	G	173	173	1	A -> G	201	SNP (transition)	45.80%	########	D -> G	RTX toxin CDS	RTX toxin	Substitution	KLI69968.1
Variants: VaAres1_A_FDSW210055060-2r	A	201	201	1	G -> A	202	SNP (transition)	55.00%	0		RTX toxin CDS	RTX toxin	None	KLI69968.1
Variants: VaAres1_A_FDSW210055060-2r	G	363	363	1	A -> G	211	SNP (transition)	52.10%	1.5E-323		RTX toxin CDS	RTX toxin	None	KLI69968.1

Variants: VaAres1_A_FDSW210055060-2r	G	427	427	1	A -> G	204	SNP (transition)	53.40%	2.7E-322		I -> V	RTX toxin CDS	RTX toxin	Substitution	KLI69968.1
Variants: VaAres1_A_FDSW210055060-2r	C	437	437	1	T -> C	206	SNP (transition)	55.30%		0	L -> S	RTX toxin CDS	RTX toxin	Substitution	KLI69968.1
Variants: VaAres1_A_FDSW210055060-2r	G	723	723	1	A -> G	203	SNP (transition)	55.70%		0		RTX toxin CDS	RTX toxin	None	KLI69968.1
Variants: VaAres1_A_FDSW210055060-2r	G	728	728	1	T -> G	203	SNP (transversion)	54.20%		0	V -> G	RTX toxin CDS	RTX toxin	Substitution	KLI69968.1
Variants: VaAres1_A_FDSW210055060-2r	A	745	745	1	G -> A	206	SNP (transition)	50.00%	########		A -> T	RTX toxin CDS	RTX toxin	Substitution	KLI69968.1
Variants: VaAres1_A_FDSW210055060-2r	A	840	840	1	G -> A	192	SNP (transition)	50.00%	########			RTX toxin CDS	RTX toxin	None	KLI69968.1
Variants: VaAres1_A_FDSW210055060-2r	C	986	986	1	T -> C	190	SNP (transition)	49.50%	########		I -> T	RTX toxin CDS	RTX toxin	Substitution	KLI69968.1
Variants: VaAres1_A_FDSW210055060-2r	T	1019	1019	1	C -> T	195	SNP (transition)	48.20%	########		T -> M	RTX toxin CDS	RTX toxin	Substitution	KLI69968.1
Variants: VaAres1_A_FDSW210055060-2r	C	1122	1122	1	A -> C	208	SNP (transversion)	51.40%		0		RTX toxin CDS	RTX toxin	None	KLI69968.1
Variants: VaAres1_A_FDSW210055060-2r	A	1159	1159	1	G -> A	205	SNP (transition)	50.70%	1.1E-314		A -> T	RTX toxin CDS	RTX toxin	Substitution	KLI69968.1
Variants: VaAres1_A_FDSW210055060-2r	G	1191	1191	1	A -> G	200	SNP (transition)	47.50%	########			RTX toxin CDS	RTX toxin	None	KLI69968.1
Variants: VaAres1_A_FDSW210055060-2r	T	1323	1323	1	C -> T	193	SNP (transition)	51.80%	########			RTX toxin CDS	RTX toxin	None	KLI69968.1
Variants: VaAres1_A_FDSW210055060-2r	C	1353	1353	1	T -> C	188	SNP (transition)	49.50%	########			RTX toxin CDS	RTX toxin	None	KLI69968.1
Variants: VaAres1_A_FDSW210055060-2r	T	1418	1418	1	C -> T	164	SNP (transition)	43.90%	########		S -> L	RTX toxin CDS	RTX toxin	Substitution	KLI69968.1
Variants: VaAres1_A_FDSW210055060-2r	G	1425	1425	1	A -> G	163	SNP (transition)	41.70%	########			RTX toxin CDS	RTX toxin	None	KLI69968.1
Variants: VaAres1_B_FDSW210055061-2r	G	159	159	1	T -> G	230	SNP (transversion)	47.80%		0		RTX toxin CDS	RTX toxin	None	KLI69968.1
Variants: VaAres1_B_FDSW210055061-2r	G	173	173	1	A -> G	213	SNP (transition)	46.50%	########		D -> G	RTX toxin CDS	RTX toxin	Substitution	KLI69968.1
Variants: VaAres1_B_FDSW210055061-2r	A	201	201	1	G -> A	214	SNP (transition)	51.90%		0		RTX toxin CDS	RTX toxin	None	KLI69968.1
Variants: VaAres1_B_FDSW210055061-2r	G	363	363	1	A -> G	205	SNP (transition)	48.30%	########			RTX toxin CDS	RTX toxin	None	KLI69968.1
Variants: VaAres1_B_FDSW210055061-2r	G	427	427	1	A -> G	223	SNP (transition)	49.30%	6.8E-320		I -> V	RTX toxin CDS	RTX toxin	Substitution	KLI69968.1
Variants: VaAres1_B_FDSW210055061-2r	C	437	437	1	T -> C	211	SNP (transition)	51.20%	1.6E-316		L -> S	RTX toxin CDS	RTX toxin	Substitution	KLI69968.1
Variants: VaAres1_B_FDSW210055061-2r	G	723	723	1	A -> G	238	SNP (transition)	51.70%		0		RTX toxin CDS	RTX toxin	None	KLI69968.1
Variants: VaAres1_B_FDSW210055061-2r	G	728	728	1	T -> G	234	SNP (transversion)	53.80%		0	V -> G	RTX toxin CDS	RTX toxin	Substitution	KLI69968.1
Variants: VaAres1_B_FDSW210055061-2r	A	745	745	1	G -> A	225	SNP (transition)	50.70%	6.8E-322		A -> T	RTX toxin CDS	RTX toxin	Substitution	KLI69968.1
Variants: VaAres1_B_FDSW210055061-2r	A	840	840	1	G -> A	208	SNP (transition)	46.60%	########			RTX toxin CDS	RTX toxin	None	KLI69968.1
Variants: VaAres1_B_FDSW210055061-2r	C	986	986	1	T -> C	211	SNP (transition)	54.50%		0	I -> T	RTX toxin CDS	RTX toxin	Substitution	KLI69968.1
Variants: VaAres1_B_FDSW210055061-2r	T	1019	1019	1	C -> T	218	SNP (transition)	54.10%		0	T -> M	RTX toxin CDS	RTX toxin	Substitution	KLI69968.1
Variants: VaAres1_B_FDSW210055061-2r	C	1122	1122	1	A -> C	231	SNP (transversion)	52.40%		0		RTX toxin CDS	RTX toxin	None	KLI69968.1
Variants: VaAres1_B_FDSW210055061-2r	A	1159	1159	1	G -> A	223	SNP (transition)	51.10%		0	A -> T	RTX toxin CDS	RTX toxin	Substitution	KLI69968.1
Variants: VaAres1_B_FDSW210055061-2r	G	1191	1191	1	A -> G	214	SNP (transition)	49.50%	3.5E-319			RTX toxin CDS	RTX toxin	None	KLI69968.1
Variants: VaAres1_B_FDSW210055061-2r	T	1323	1323	1	C -> T	236	SNP (transition)	49.20%		0		RTX toxin CDS	RTX toxin	None	KLI69968.1
Variants: VaAres1_B_FDSW210055061-2r	C	1353	1353	1	T -> C	212	SNP (transition)	47.20%	########			RTX toxin CDS	RTX toxin	None	KLI69968.1

Variants: VaAres1_B_FDSW210055061-2r	T	1418	1418	1	C -> T	188	SNP (transition)	41.00%	########		S -> L	RTX toxin CDS	RTX toxin	Substitution	KLI69968.1
Variants: VaAres1_B_FDSW210055061-2r	G	1425	1425	1	A -> G	185	SNP (transition)	41.10%	########			RTX toxin CDS	RTX toxin	None	KLI69968.1
Variants: VaAres1_C_FDSW210055062-1r	G	159	159	1	T -> G	266	SNP (transversion)	50.80%		0		RTX toxin CDS	RTX toxin	None	KLI69968.1
Variants: VaAres1_C_FDSW210055062-1r	G	173	173	1	A -> G	278	SNP (transition)	52.20%		0	D -> G	RTX toxin CDS	RTX toxin	Substitution	KLI69968.1
Variants: VaAres1_C_FDSW210055062-1r	A	201	201	1	G -> A	275	SNP (transition)	47.30%		0		RTX toxin CDS	RTX toxin	None	KLI69968.1
Variants: VaAres1_C_FDSW210055062-1r	G	363	363	1	A -> G	315	SNP (transition)	52.40%		0		RTX toxin CDS	RTX toxin	None	KLI69968.1
Variants: VaAres1_C_FDSW210055062-1r	G	427	427	1	A -> G	312	SNP (transition)	53.80%		0	I -> V	RTX toxin CDS	RTX toxin	Substitution	KLI69968.1
Variants: VaAres1_C_FDSW210055062-1r	C	437	437	1	T -> C	304	SNP (transition)	54.60%		0	L -> S	RTX toxin CDS	RTX toxin	Substitution	KLI69968.1
Variants: VaAres1_C_FDSW210055062-1r	G	723	723	1	A -> G	319	SNP (transition)	51.10%		0		RTX toxin CDS	RTX toxin	None	KLI69968.1
Variants: VaAres1_C_FDSW210055062-1r	G	728	728	1	T -> G	316	SNP (transversion)	50.30%		0	V -> G	RTX toxin CDS	RTX toxin	Substitution	KLI69968.1
Variants: VaAres1_C_FDSW210055062-1r	A	745	745	1	G -> A	309	SNP (transition)	50.50%		0	A -> T	RTX toxin CDS	RTX toxin	Substitution	KLI69968.1
Variants: VaAres1_C_FDSW210055062-1r	A	840	840	1	G -> A	303	SNP (transition)	50.20%		0		RTX toxin CDS	RTX toxin	None	KLI69968.1
Variants: VaAres1_C_FDSW210055062-1r	C	986	986	1	T -> C	273	SNP (transition)	51.30%		0	I -> T	RTX toxin CDS	RTX toxin	Substitution	KLI69968.1
Variants: VaAres1_C_FDSW210055062-1r	T	1019	1019	1	C -> T	270	SNP (transition)	52.20%		0	T -> M	RTX toxin CDS	RTX toxin	Substitution	KLI69968.1
Variants: VaAres1_C_FDSW210055062-1r	C	1122	1122	1	A -> C	268	SNP (transversion)	51.50%		0		RTX toxin CDS	RTX toxin	None	KLI69968.1
Variants: VaAres1_C_FDSW210055062-1r	A	1159	1159	1	G -> A	279	SNP (transition)	50.90%		0	A -> T	RTX toxin CDS	RTX toxin	Substitution	KLI69968.1
Variants: VaAres1_C_FDSW210055062-1r	G	1191	1191	1	A -> G	278	SNP (transition)	48.20%		0		RTX toxin CDS	RTX toxin	None	KLI69968.1
Variants: VaAres1_C_FDSW210055062-1r	T	1323	1323	1	C -> T	278	SNP (transition)	48.90%		0		RTX toxin CDS	RTX toxin	None	KLI69968.1
Variants: VaAres1_C_FDSW210055062-1r	C	1353	1353	1	T -> C	280	SNP (transition)	46.40%		0		RTX toxin CDS	RTX toxin	None	KLI69968.1
Variants: VaAres1_C_FDSW210055062-1r	T	1418	1418	1	C -> T	250	SNP (transition)	45.60%	########		S -> L	RTX toxin CDS	RTX toxin	Substitution	KLI69968.1
Variants: VaAres1_C_FDSW210055062-1r	G	1425	1425	1	A -> G	244	SNP (transition)	44.70%	########			RTX toxin CDS	RTX toxin	None	KLI69968.1
Variants: VaphiVaphiSt2_B_A_FDSW210055054-2r	G	159	159	1	T -> G	201	SNP (transversion)	53.20%		0		RTX toxin CDS	RTX toxin	None	KLI69968.1
Variants: VaphiVaphiSt2_B_A_FDSW210055054-2r	G	173	173	1	A -> G	204	SNP (transition)	52.50%	3.4E-315		D -> G	RTX toxin CDS	RTX toxin	Substitution	KLI69968.1
Variants: VaphiVaphiSt2_B_A_FDSW210055054-2r	A	201	201	1	G -> A	197	SNP (transition)	49.70%	########			RTX toxin CDS	RTX toxin	None	KLI69968.1
Variants: VaphiVaphiSt2_B_A_FDSW210055054-2r	G	363	363	1	A -> G	214	SNP (transition)	49.10%	########			RTX toxin CDS	RTX toxin	None	KLI69968.1
Variants: VaphiVaphiSt2_B_A_FDSW210055054-2r	G	427	427	1	A -> G	226	SNP (transition)	53.50%		0	I -> V	RTX toxin CDS	RTX toxin	Substitution	KLI69968.1
Variants: VaphiVaphiSt2_B_A_FDSW210055054-2r	C	437	437	1	T -> C	220	SNP (transition)	54.50%		0	L -> S	RTX toxin CDS	RTX toxin	Substitution	KLI69968.1
Variants: VaphiVaphiSt2_B_A_FDSW210055054-2r	G	723	723	1	A -> G	184	SNP (transition)	50.50%	########			RTX toxin CDS	RTX toxin	None	KLI69968.1

Variants: VaphiVaphiSt2_B_A_FDSW210055054-2r	G	728	728	1	T -> G	186	SNP (transversion)	51.10%	########	V -> G	RTX toxin CDS	RTX toxin	Substitution	KLI69968.1
Variants: VaphiVaphiSt2_B_A_FDSW210055054-2r	A	745	745	1	G -> A	174	SNP (transition)	50.00%	########	A -> T	RTX toxin CDS	RTX toxin	Substitution	KLI69968.1
Variants: VaphiVaphiSt2_B_A_FDSW210055054-2r	A	840	840	1	G -> A	172	SNP (transition)	51.20%	########		RTX toxin CDS	RTX toxin	None	KLI69968.1
Variants: VaphiVaphiSt2_B_A_FDSW210055054-2r	C	986	986	1	T -> C	180	SNP (transition)	51.70%	########	I -> T	RTX toxin CDS	RTX toxin	Substitution	KLI69968.1
Variants: VaphiVaphiSt2_B_A_FDSW210055054-2r	T	1019	1019	1	C -> T	183	SNP (transition)	48.60%	########	T -> M	RTX toxin CDS	RTX toxin	Substitution	KLI69968.1
Variants: VaphiVaphiSt2_B_A_FDSW210055054-2r	C	1122	1122	1	A -> C	211	SNP (transversion)	45.50%	########		RTX toxin CDS	RTX toxin	None	KLI69968.1
Variants: VaphiVaphiSt2_B_A_FDSW210055054-2r	A	1159	1159	1	G -> A	225	SNP (transition)	46.70%	1.7E-312	A -> T	RTX toxin CDS	RTX toxin	Substitution	KLI69968.1
Variants: VaphiVaphiSt2_B_A_FDSW210055054-2r	G	1191	1191	1	A -> G	227	SNP (transition)	45.80%	0.00E+00		RTX toxin CDS	RTX toxin	None	KLI69968.1
Variants: VaphiVaphiSt2_B_A_FDSW210055054-2r	T	1323	1323	1	C -> T	168	SNP (transition)	47.00%	########		RTX toxin CDS	RTX toxin	None	KLI69968.1
Variants: VaphiVaphiSt2_B_A_FDSW210055054-2r	C	1353	1353	1	T -> C	170	SNP (transition)	47.60%	########		RTX toxin CDS	RTX toxin	None	KLI69968.1
Variants: VaphiVaphiSt2_B_A_FDSW210055054-2r	T	1418	1418	1	C -> T	157	SNP (transition)	49.70%	########	S -> L	RTX toxin CDS	RTX toxin	Substitution	KLI69968.1
Variants: VaphiVaphiSt2_B_A_FDSW210055054-2r	G	1425	1425	1	A -> G	148	SNP (transition)	46.60%	########		RTX toxin CDS	RTX toxin	None	KLI69968.1
Variants: VaphiSt2_B_FDSW210055056-1r	G	159	159	1	T -> G	382	SNP (transversion)	48.70%	0		RTX toxin CDS	RTX toxin	None	KLI69968.1
Variants: VaphiSt2_B_FDSW210055056-1r	G	173	173	1	A -> G	374	SNP (transition)	47.60%	0	D -> G	RTX toxin CDS	RTX toxin	Substitution	KLI69968.1
Variants: VaphiSt2_B_FDSW210055056-1r	A	201	201	1	G -> A	386	SNP (transition)	51.00%	0		RTX toxin CDS	RTX toxin	None	KLI69968.1
Variants: VaphiSt2_B_FDSW210055056-1r	G	363	363	1	A -> G	431	SNP (transition)	47.30%	0		RTX toxin CDS	RTX toxin	None	KLI69968.1
Variants: VaphiSt2_B_FDSW210055056-1r	G	427	427	1	A -> G	420	SNP (transition)	48.30%	0	I -> V	RTX toxin CDS	RTX toxin	Substitution	KLI69968.1
Variants: VaphiSt2_B_FDSW210055056-1r	C	437	437	1	T -> C	411	SNP (transition)	48.40%	0	L -> S	RTX toxin CDS	RTX toxin	Substitution	KLI69968.1
Variants: VaphiSt2_B_FDSW210055056-1r	G	723	723	1	A -> G	398	SNP (transition)	54.50%	0		RTX toxin CDS	RTX toxin	None	KLI69968.1
Variants: VaphiSt2_B_FDSW210055056-1r	G	728	728	1	T -> G	392	SNP (transversion)	53.30%	0	V -> G	RTX toxin CDS	RTX toxin	Substitution	KLI69968.1
Variants: VaphiSt2_B_FDSW210055056-1r	A	745	745	1	G -> A	389	SNP (transition)	50.60%	0	A -> T	RTX toxin CDS	RTX toxin	Substitution	KLI69968.1
Variants: VaphiSt2_B_FDSW210055056-1r	A	840	840	1	G -> A	416	SNP (transition)	55.50%	0		RTX toxin CDS	RTX toxin	None	KLI69968.1
Variants: VaphiSt2_B_FDSW210055056-1r	C	986	986	1	T -> C	370	SNP (transition)	46.80%	0	I -> T	RTX toxin CDS	RTX toxin	Substitution	KLI69968.1
Variants: VaphiSt2_B_FDSW210055056-1r	T	1019	1019	1	C -> T	391	SNP (transition)	48.80%	0	T -> M	RTX toxin CDS	RTX toxin	Substitution	KLI69968.1
Variants: VaphiSt2_B_FDSW210055056-1r	C	1122	1122	1	A -> C	400	SNP (transversion)	53.80%	0		RTX toxin CDS	RTX toxin	None	KLI69968.1
Variants: VaphiSt2_B_FDSW210055056-1r	A	1159	1159	1	G -> A	380	SNP (transition)	56.60%	0	A -> T	RTX toxin CDS	RTX toxin	Substitution	KLI69968.1
Variants: VaphiSt2_B_FDSW210055056-1r	G	1191	1191	1	A -> G	387	SNP (transition)	55.30%	0		RTX toxin CDS	RTX toxin	None	KLI69968.1

Variants: VaphiSt2_B_FDSW210055056-1r	T	1323	1323	1	C -> T	434	SNP (transition)	46.50%	0		RTX toxin CDS	RTX toxin	None	KLI69968.1
Variants: VaphiSt2_B_FDSW210055056-1r	C	1353	1353	1	T -> C	418	SNP (transition)	44.70%	0		RTX toxin CDS	RTX toxin	None	KLI69968.1
Variants: VaphiSt2_B_FDSW210055056-1r	T	1418	1418	1	C -> T	351	SNP (transition)	47.60%	0	S -> L	RTX toxin CDS	RTX toxin	Substitution	KLI69968.1
Variants: VaphiSt2_B_FDSW210055056-1r	G	1425	1425	1	A -> G	341	SNP (transition)	46.30%	0		RTX toxin CDS	RTX toxin	None	KLI69968.1

Contig 60

Variant	Name	Minimum	Maximum	Length	Change	Coverage	Polymorphism Type	Variant Frequency	Variant P-Value (approximate)
Variants: VaAphrodite_A_FDSW210055057-2r	C	98	98	1	T -> C	194	SNP (transition)	42.30%	8.20E-199
Variants: VaAphrodite_B_FDSW210055058-2r	C	98	98	1	T -> C	201	SNP (transition)	37.30%	7.70E-177
Variants: VaAphrodite_C_FDSW210055059-1r	C	98	98	1	T -> C	271	SNP (transition)	40.20%	1.50E-271
Variants: VaAres1_A_FDSW210055060-2r	C	98	98	1	T -> C	147	SNP (transition)	33.30%	5.70E-123
Variants: VaAres1_B_FDSW210055061-2r	C	98	98	1	T -> C	145	SNP (transition)	38.60%	4.10E-139
Variants: VaAres1_C_FDSW210055062-1r	C	98	98	1	T -> C	175	SNP (transition)	34.30%	4.30E-145
Variants: VaphiVaphiSt2_B_A_FDSW210055054-2r	C	98	98	1	T -> C	145	SNP (transition)	33.80%	1.60E-113
Variants: VaphiSt2_B_FDSW210055056-1r	C	98	98	1	T -> C	332	SNP (transition)	46.10%	0

Contig 62

Variant	Name	Minimum	Maximum	Length	Change	Coverage	Polymorphism Type	Variant Frequency	Variant P-Value (approximate)
Variants: VaAphrodite_A_FDSW210055057-2r	CG	2723	2724	2	TT -> CG	37	Substitution	97.30%	3.70E-89
Variants: VaAphrodite_A_FDSW210055057-2r	T	2728	2728	1	C -> T	36	SNP (transition)	100.00%	4.00E-87
Variants: VaAphrodite_A_FDSW210055057-2r	T	2744	2744	1	A -> T	28	SNP (transversion)	100.00%	2.50E-62
Variants: VaAphrodite_A_FDSW210055057-2r	A	2747	2747	1	G -> A	25	SNP (transition)	100.00%	1.00E-50
Variants: VaAphrodite_B_FDSW210055058-2r	CG	2723	2724	2	TT -> CG	55	Substitution	100.00%	1.00E-143
Variants: VaAphrodite_B_FDSW210055058-2r	T	2728	2728	1	C -> T	50	SNP (transition)	100.00%	1.00E-120
Variants: VaAphrodite_B_FDSW210055058-2r	T	2744	2744	1	A -> T	42	SNP (transversion)	100.00%	4.00E-93
Variants: VaAphrodite_B_FDSW210055058-2r	A	2747	2747	1	G -> A	39	SNP (transition)	97.40%	6.10E-79
Variants: VaAphrodite_C_FDSW210055059-1r	CG	2723	2724	2	TT -> CG	70 -> 72	Substitution	100.00%	1.00E-168
Variants: VaAphrodite_C_FDSW210055059-1r	T	2728	2728	1	C -> T	67	SNP (transition)	100.00%	7.90E-155
Variants: VaAphrodite_C_FDSW210055059-1r	T	2744	2744	1	A -> T	61	SNP (transversion)	100.00%	6.30E-135
Variants: VaAphrodite_C_FDSW210055059-1r	A	2747	2747	1	G -> A	60	SNP (transition)	100.00%	1.00E-132
Variants: VaAres1_A_FDSW210055060-2r	CG	2723	2724	2	TT -> CG	22	Substitution	95.50%	6.90E-52
Variants: VaAres1_A_FDSW210055060-2r	T	2728	2728	1	C -> T	19	SNP (transition)	100.00%	2.50E-46
Variants: VaAres1_A_FDSW210055060-2r	T	2744	2744	1	A -> T	10	SNP (transversion)	100.00%	1.00E-22
Variants: VaAres1_A_FDSW210055060-2r	A	2747	2747	1	G -> A	10	SNP (transition)	100.00%	1.00E-21
Variants: VaAres1_B_FDSW210055061-2r	CG	2723	2724	2	TT -> CG	55 -> 56	Substitution	98.20%	5.60E-175
Variants: VaAres1_B_FDSW210055061-2r	T	2728	2728	1	C -> T	52	SNP (transition)	100.00%	1.60E-151
Variants: VaAres1_B_FDSW210055061-2r	T	2744	2744	1	A -> T	39	SNP (transversion)	100.00%	6.30E-110
Variants: VaAres1_B_FDSW210055061-2r	A	2747	2747	1	G -> A	39	SNP (transition)	100.00%	5.00E-106
Variants: VaAres1_C_FDSW210055062-1r	CG	2723	2724	2	TT -> CG	54	Substitution	98.10%	8.50E-137
Variants: VaAres1_C_FDSW210055062-1r	T	2728	2728	1	C -> T	46	SNP (transition)	97.80%	4.60E-98
Variants: VaAres1_C_FDSW210055062-1r	T	2744	2744	1	A -> T	37	SNP (transversion)	100.00%	2.00E-78
Variants: VaAres1_C_FDSW210055062-1r	A	2747	2747	1	G -> A	37	SNP (transition)	100.00%	1.00E-74
Variants: VaphiVaphiSt2_B_A_FDSW210055054-2r	CG	2723	2724	2	TT -> CG	57 -> 60	Substitution	100.00%	2.50E-160
Variants: VaphiVaphiSt2_B_A_FDSW210055054-2r	T	2728	2728	1	C -> T	55	SNP (transition)	100.00%	1.00E-154
Variants: VaphiVaphiSt2_B_A_FDSW210055054-2r	T	2744	2744	1	A -> T	39	SNP (transversion)	100.00%	2.50E-94
Variants: VaphiVaphiSt2_B_A_FDSW210055054-2r	A	2747	2747	1	G -> A	36	SNP (transition)	100.00%	4.00E-87
Variants: VaphiSt2_B_FDSW210055056-1r	CG	2723	2724	2	TT -> CG	86 -> 90	Substitution	100.00%	2.50E-224

Variants: VaphiSt2_B_FDSW210055056-1r	T	2728	2728	1	C -> T	76	SNP (transition)	100.00%	4.00E-183
Variants: VaphiSt2_B_FDSW210055056-1r	T	2744	2744	1	A -> T	61	SNP (transversion)	100.00%	7.90E-129
Variants: VaphiSt2_B_FDSW210055056-1r	A	2747	2747	1	G -> A	58	SNP (transition)	100.00%	2.50E-128

Contig 63

Variant	Name	Minimum	Maximum	Length	Change	Coverage	Polymorphism Type	Variant Frequency	Variant P-Value (approximate)	Amino Acid Change	CDS	product	Protein Effect	protein_id
Variants: VaAphrodite_A_FDSW210055057-2r	C	3	3	1	A -> C	80	SNP (transversion)	98.80%	2.00E-267	K -> Q	hypothetical protein CDS	hypothetical protein	Substitution	KLI69899.1
Variants: VaAphrodite_B_FDSW210055058-2r	C	3	3	1	A -> C	52	SNP (transversion)	96.20%	1.30E-167	K -> Q	hypothetical protein CDS	hypothetical protein	Substitution	KLI69899.1
Variants: VaAphrodite_C_FDSW210055059-1r	C	3	3	1	A -> C	108	SNP (transversion)	98.10%	0	K -> Q	hypothetical protein CDS	hypothetical protein	Substitution	KLI69899.1
Variants: VaAres1_A_FDSW210055060-2r	C	3	3	1	A -> C	58	SNP (transversion)	96.60%	1.70E-193	K -> Q	hypothetical protein CDS	hypothetical protein	Substitution	KLI69899.1
Variants: VaAres1_B_FDSW210055061-2r	C	3	3	1	A -> C	58	SNP (transversion)	94.80%	3.10E-172	K -> Q	hypothetical protein CDS	hypothetical protein	Substitution	KLI69899.1
Variants: VaAres1_C_FDSW210055062-1r	C	3	3	1	A -> C	83	SNP (transversion)	97.60%	1.40E-272	K -> Q	hypothetical protein CDS	hypothetical protein	Substitution	KLI69899.1
Variants: VaphiVaphiSt2_B_A_FDSW210055054-2r	C	3	3	1	A -> C	63	SNP (transversion)	98.40%	1.60E-203	K -> Q	hypothetical protein CDS	hypothetical protein	Substitution	KLI69899.1
Variants: VaphiSt2_B_FDSW210055056-1r	C	3	3	1	A -> C	119	SNP (transversion)	97.50%	0	K -> Q	hypothetical protein CDS	hypothetical protein	Substitution	KLI69899.1

Contig 64

Variant	Name	Minimum	Maximum	Length	Change	Coverage	Polymorphism Type	Variant Frequency	Variant P-Value (approximate)
Variants: VaAres1_C_FDSW210055062-1r	C	4910	4910	1	T -> C	213	SNP (transition)	25.80%	3.70E-114
Variants: VaphiVaphiSt2_B_A_FDSW210055054-2r	A	4892	4892	1	G -> A	178	SNP (transition)	25.30%	2.90E-84
Variants: VaphiVaphiSt2_B_A_FDSW210055054-2r	C	4910	4910	1	T -> C	197	SNP (transition)	26.90%	3.70E-111

Contig 65

Variant	Name	Minimum	Maximum	Length	Change	Coverage	Polymorphism Type	Variant Frequency	Variant P-Value (approximate)
Variants: VaAphrodite_B_FDSW210055058-2r	C	6681	6681	1	T -> C	94	SNP (transition)	26.60%	4.00E-63
Variants: VaAphrodite_B_FDSW210055058-2r	T	6686	6686	1	A -> T	100	SNP (transversion)	29.00%	1.50E-65
Variants: VaAphrodite_B_FDSW210055058-2r	A	6690	6689	0	#NAME?	101	Insertion	29.70%	4.00E-68
Variants: VaAres1_A_FDSW210055060-2r	C	6775	6775	1	T -> C	17	SNP (transition)	29.40%	2.00E-15
Variants: VaAres1_B_FDSW210055061-2r	C	6775	6775	1	T -> C	38	SNP (transition)	28.90%	2.40E-32
Variants: VaphiVaphiSt2_B_A_FDSW210055054-2r	C	6775	6775	1	T -> C	48	SNP (transition)	35.40%	2.70E-49

Contig 66

Variant	Name	Minimum	Maximum	Length	Change	Coverage	Polymorphism Type	Variant Frequency	Variant P-Value (approximate)
Variants: VaAphrodite_A_FDSW210055057-2r	AC	1609	1610	2	GA -> AC	135	Substitution	100.00%	0
Variants: VaAphrodite_A_FDSW210055057-2r	A	1612	1612	1	C -> A	134	SNP (transversion)	100.00%	0
Variants: VaAphrodite_A_FDSW210055057-2r	T	1614	1614	1	A -> T	133	SNP (transversion)	100.00%	0
Variants: VaAphrodite_A_FDSW210055057-2r	T	1616	1616	1	C -> T	130	SNP (transition)	100.00%	0
Variants: VaAphrodite_A_FDSW210055057-2r	A	1618	1618	1	G -> A	131	SNP (transition)	100.00%	0
Variants: VaAphrodite_A_FDSW210055057-2r	C	1620	1620	1	T -> C	133	SNP (transition)	100.00%	0
Variants: VaAphrodite_B_FDSW210055058-2r	AC	1609	1610	2	GA -> AC	116 -> 117	Substitution	100.00%	0
Variants: VaAphrodite_B_FDSW210055058-2r	A	1612	1612	1	C -> A	118	SNP (transversion)	100.00%	0
Variants: VaAphrodite_B_FDSW210055058-2r	T	1614	1614	1	A -> T	121	SNP (transversion)	100.00%	0
Variants: VaAphrodite_B_FDSW210055058-2r	T	1616	1616	1	C -> T	120	SNP (transition)	100.00%	0
Variants: VaAphrodite_B_FDSW210055058-2r	A	1618	1618	1	G -> A	119	SNP (transition)	100.00%	0
Variants: VaAphrodite_B_FDSW210055058-2r	C	1620	1620	1	T -> C	119	SNP (transition)	100.00%	0
Variants: VaAphrodite_C_FDSW210055059-1r	AC	1609	1610	2	GA -> AC	167	Substitution	100.00%	0
Variants: VaAphrodite_C_FDSW210055059-1r	A	1612	1612	1	C -> A	168	SNP (transversion)	100.00%	0
Variants: VaAphrodite_C_FDSW210055059-1r	T	1614	1614	1	A -> T	163	SNP (transversion)	100.00%	0
Variants: VaAphrodite_C_FDSW210055059-1r	T	1616	1616	1	C -> T	163	SNP (transition)	100.00%	0
Variants: VaAphrodite_C_FDSW210055059-1r	A	1618	1618	1	G -> A	166	SNP (transition)	100.00%	0
Variants: VaAphrodite_C_FDSW210055059-1r	C	1620	1620	1	T -> C	163	SNP (transition)	100.00%	0
Variants: VaAres1_A_FDSW210055060-2r	AC	1609	1610	2	GA -> AC	83 -> 86	Substitution	98.80%	8.30E-286
Variants: VaAres1_A_FDSW210055060-2r	A	1612	1612	1	C -> A	84	SNP (transversion)	100.00%	4.00E-261
Variants: VaAres1_A_FDSW210055060-2r	T	1614	1614	1	A -> T	82	SNP (transversion)	100.00%	1.00E-287
Variants: VaAres1_A_FDSW210055060-2r	T	1616	1616	1	C -> T	81	SNP (transition)	98.80%	8.10E-271
Variants: VaAres1_A_FDSW210055060-2r	A	1618	1618	1	G -> A	81	SNP (transition)	100.00%	3.20E-284
Variants: VaAres1_A_FDSW210055060-2r	C	1620	1620	1	T -> C	80	SNP (transition)	100.00%	1.00E-280
Variants: VaAres1_B_FDSW210055061-2r	AC	1609	1610	2	GA -> AC	100 -> 102	Substitution	100.00%	0
Variants: VaAres1_B_FDSW210055061-2r	A	1612	1612	1	C -> A	100	SNP (transversion)	100.00%	0
Variants: VaAres1_B_FDSW210055061-2r	T	1614	1614	1	A -> T	100	SNP (transversion)	99.00%	0
Variants: VaAres1_B_FDSW210055061-2r	T	1616	1616	1	C -> T	101	SNP (transition)	100.00%	0
Variants: VaAres1_B_FDSW210055061-2r	A	1618	1618	1	G -> A	100	SNP (transition)	100.00%	0

Variants: VaAres1_B_FDSW210055061-2r	C	1620	1620	1	T -> C	99	SNP (transition)	100.00%	0
Variants: VaAres1_C_FDSW210055062-1r	AC	1609	1610	2	GA -> AC	117 -> 118	Substitution	99.1% -> 99.2%	0
Variants: VaAres1_C_FDSW210055062-1r	A	1612	1612	1	C -> A	117	SNP (transversion)	100.00%	0
Variants: VaAres1_C_FDSW210055062-1r	T	1614	1614	1	A -> T	117	SNP (transversion)	99.10%	0
Variants: VaAres1_C_FDSW210055062-1r	T	1616	1616	1	C -> T	119	SNP (transition)	100.00%	0
Variants: VaAres1_C_FDSW210055062-1r	A	1618	1618	1	G -> A	121	SNP (transition)	99.20%	0
Variants: VaAres1_C_FDSW210055062-1r	C	1620	1620	1	T -> C	123	SNP (transition)	99.20%	0
Variants: VaphiVaphiSt2_B_A_FDSW210055054-2r	AC	1609	1610	2	GA -> AC	116 -> 118	Substitution	100.00%	0
Variants: VaphiVaphiSt2_B_A_FDSW210055054-2r	A	1612	1612	1	C -> A	118	SNP (transversion)	99.20%	0
Variants: VaphiVaphiSt2_B_A_FDSW210055054-2r	T	1614	1614	1	A -> T	118	SNP (transversion)	98.30%	0
Variants: VaphiVaphiSt2_B_A_FDSW210055054-2r	T	1616	1616	1	C -> T	119	SNP (transition)	100.00%	0
Variants: VaphiVaphiSt2_B_A_FDSW210055054-2r	A	1618	1618	1	G -> A	118	SNP (transition)	99.20%	0
Variants: VaphiVaphiSt2_B_A_FDSW210055054-2r	C	1620	1620	1	T -> C	119	SNP (transition)	100.00%	0
Variants: VaphiSt2_B_FDSW210055056-1r	AC	1609	1610	2	GA -> AC	202 -> 203	Substitution	99.50%	0
Variants: VaphiSt2_B_FDSW210055056-1r	A	1612	1612	1	C -> A	202	SNP (transversion)	100.00%	0
Variants: VaphiSt2_B_FDSW210055056-1r	T	1614	1614	1	A -> T	203	SNP (transversion)	100.00%	0
Variants: VaphiSt2_B_FDSW210055056-1r	T	1616	1616	1	C -> T	200	SNP (transition)	100.00%	0
Variants: VaphiSt2_B_FDSW210055056-1r	A	1618	1618	1	G -> A	200	SNP (transition)	100.00%	0
Variants: VaphiSt2_B_FDSW210055056-1r	C	1620	1620	1	T -> C	201	SNP (transition)	100.00%	0

Contig 68

Variant	Name	Minimum	Maximum	Length	Change	Coverage	Polymorphism Type	Variant Frequency	Variant P-Value (approximate)	Amino Acid Change	CDS	product	Protein Effect	protein_id
Variants: VaAphrodite_A_FDSW210055057-2r	T	79	79	1	C -> T	68	SNP (transition)	82.40%	4.50E-139					
Variants: VaAphrodite_A_FDSW210055057-2r	GG	80	81	2	CT -> GG	60 -> 65	Substitution	67.7% -> 68.3%	4.90E-92					
Variants: VaAphrodite_A_FDSW210055057-2r	G	84	84	1	A -> G	46	SNP (transition)	80.40%	8.40E-77					
Variants: VaAphrodite_A_FDSW210055057-2r	G	86	86	1	C -> G	39	SNP (transversion)	59.00%	1.10E-47					
Variants: VaAphrodite_A_FDSW210055057-2r	G	87	87	1	A -> G	39	SNP (transition)	59.00%	2.20E-45					
Variants: VaAphrodite_A_FDSW210055057-2r	GG	88	89	2	TC -> GG	37 -> 39	Substitution	35.1% -> 46.2%	2.70E-26					
Variants: VaAphrodite_A_FDSW210055057-2r	G	96	96	1	T -> G	55	SNP (transversion)	27.30%	2.30E-16					
Variants: VaAphrodite_A_FDSW210055057-2r	C	102	102	1	T -> C	76	SNP (transition)	34.20%	3.70E-25					
Variants: VaAphrodite_A_FDSW210055057-2r	T	108	108	1	A -> T	105	SNP (transversion)	28.60%	1.50E-64					
Variants: VaAphrodite_A_FDSW210055057-2r	G	120	120	1	A -> G	146	SNP (transition)	30.80%	4.50E-40					
Variants: VaAphrodite_A_FDSW210055057-2r	T	126	126	1	C -> T	171	SNP (transition)	25.70%	2.00E-35					
Variants: VaAphrodite_A_FDSW210055057-2r	T	129	129	1	C -> T	182	SNP (transition)	59.30%	1.20E-218					
Variants: VaAphrodite_A_FDSW210055057-2r	G	132	132	1	C -> G	197	SNP (transversion)	48.20%	8.60E-209					
Variants: VaAphrodite_A_FDSW210055057-2r	T	144	144	1	C -> T	239	SNP (transition)	27.20%	5.70E-72					
Variants: VaAphrodite_A_FDSW210055057-2r	G	153	153	1	A -> G	280	SNP (transition)	27.90%	2.70E-195					
Variants: VaAphrodite_A_FDSW210055057-2r	A	159	159	1	G -> A	305	SNP (transition)	65.20%	0					
Variants: VaAphrodite_A_FDSW210055057-2r	A	165	165	1	T -> A	329	SNP (transversion)	41.30%	1.50E-272					
Variants: VaAphrodite_A_FDSW210055057-2r	A	183	183	1	G -> A	434	SNP (transition)	29.50%	3.70E-259					
Variants: VaAphrodite_A_FDSW210055057-2r	A	192	192	1	G -> A	479	SNP (transition)	48.00%	0					
Variants: VaAphrodite_A_FDSW210055057-2r	A	201	201	1	G -> A	524	SNP (transition)	28.60%	4.9E-316					

Variants: VaAphrodite_A_FDSW210055 057-2r	G	216	216	1	A -> G	592	SNP (transition)	34.50%	0
Variants: VaAphrodite_A_FDSW210055 057-2r	C	225	225	1	A -> C	623	SNP (transversion)	25.80%	0
Variants: VaAphrodite_A_FDSW210055 057-2r	T	225	225	1	A -> T	623	SNP (transversion)	33.50%	0
Variants: VaAphrodite_A_FDSW210055 057-2r	T	227	227	1	C -> T	625	SNP (transition)	29.30%	0
Variants: VaAphrodite_A_FDSW210055 057-2r	T	239	239	1	C -> T	653	SNP (transition)	28.20%	0
Variants: VaAphrodite_A_FDSW210055 057-2r	C	240	240	1	T -> C	652	SNP (transition)	31.90%	0
Variants: VaAphrodite_A_FDSW210055 057-2r	A	294	294	1	G -> A	703	SNP (transition)	44.40%	0
Variants: VaAphrodite_A_FDSW210055 057-2r	A	303	303	1	T -> A	729	SNP (transversion)	27.20%	0
Variants: VaAphrodite_A_FDSW210055 057-2r	G	306	306	1	A -> G	743	SNP (transition)	26.90%	0
Variants: VaAphrodite_A_FDSW210055 057-2r	A	315	315	1	G -> A	774	SNP (transition)	25.30%	0
Variants: VaAphrodite_A_FDSW210055 057-2r	A	357	357	1	G -> A	901	SNP (transition)	49.40%	0
Variants: VaAphrodite_A_FDSW210055 057-2r	G	363	363	1	A -> G	929	SNP (transition)	53.10%	0
Variants: VaAphrodite_A_FDSW210055 057-2r	C	369	369	1	T -> C	953	SNP (transition)	83.70%	0
Variants: VaAphrodite_A_FDSW210055 057-2r	A	371	371	1	G -> A	956	SNP (transition)	56.10%	0
Variants: VaAphrodite_A_FDSW210055 057-2r	G	387	387	1	C -> G	1062	SNP (transversion)	58.60%	0
Variants: VaAphrodite_A_FDSW210055 057-2r	C	393	393	1	G -> C	1095	SNP (transversion)	49.40%	0
Variants: VaAphrodite_A_FDSW210055 057-2r	C	396	396	1	A -> C	1116	SNP (transversion)	34.80%	0
Variants: VaAphrodite_A_FDSW210055 057-2r	G	420	420	1	A -> G	1222	SNP (transition)	31.50%	0
Variants: VaAphrodite_A_FDSW210055 057-2r	T	420	420	1	A -> T	1222	SNP (transversion)	35.40%	0
Variants: VaAphrodite_A_FDSW210055 057-2r	A	422	422	1	T -> A	1219	SNP (transversion)	35.30%	0
Variants: VaAphrodite_A_FDSW210055 057-2r	A	423	423	1	G -> A	1222	SNP (transition)	65.60%	0
Variants: VaAphrodite_A_FDSW210055 057-2r	A	426	426	1	G -> A	1226	SNP (transition)	64.80%	0

Variants: VaAphrodite_A_FDSW210055 057-2r	G	438	438	1	A -> G	1261	SNP (transition)	49.70%	0
Variants: VaAphrodite_A_FDSW210055 057-2r	A	456	456	1	G -> A	1255	SNP (transition)	47.80%	0
Variants: VaAphrodite_A_FDSW210055 057-2r	C	489	489	1	T -> C	1246	SNP (transition)	42.10%	0
Variants: VaAphrodite_A_FDSW210055 057-2r	C	498	498	1	G -> C	1209	SNP (transversion)	27.50%	0
Variants: VaAphrodite_A_FDSW210055 057-2r	C	513	513	1	T -> C	1182	SNP (transition)	50.20%	0
Variants: VaAphrodite_A_FDSW210055 057-2r	C	516	516	1	T -> C	1178	SNP (transition)	32.90%	0
Variants: VaAphrodite_A_FDSW210055 057-2r	T	531	531	1	C -> T	1200	SNP (transition)	29.90%	0
Variants: VaAphrodite_A_FDSW210055 057-2r	C	537	537	1	G -> C	1177	SNP (transversion)	56.40%	0
Variants: VaAphrodite_A_FDSW210055 057-2r	G	540	540	1	A -> G	1167	SNP (transition)	36.50%	0
Variants: VaAphrodite_A_FDSW210055 057-2r	A	552	552	1	T -> A	1124	SNP (transversion)	34.50%	0
Variants: VaAphrodite_A_FDSW210055 057-2r	A	564	564	1	C -> A	1108	SNP (transversion)	30.10%	0
Variants: VaAphrodite_A_FDSW210055 057-2r	G	588	588	1	A -> G	1037	SNP (transition)	39.50%	0
Variants: VaAphrodite_A_FDSW210055 057-2r	C	663	663	1	T -> C	568	SNP (transition)	63.70%	0
Variants: VaAphrodite_A_FDSW210055 057-2r	G	677	677	1	A -> G	424	SNP (transition)	63.00%	0
Variants: VaAphrodite_A_FDSW210055 057-2r	C	684	684	1	T -> C	372	SNP (transition)	56.50%	0
Variants: VaAphrodite_A_FDSW210055 057-2r	A	687	687	1	G -> A	354	SNP (transition)	56.50%	0
Variants: VaAphrodite_A_FDSW210055 057-2r	T	690	690	1	A -> T	340	SNP (transversion)	57.90%	0
Variants: VaAphrodite_A_FDSW210055 057-2r	A	693	693	1	G -> A	321	SNP (transition)	57.60%	0
Variants: VaAphrodite_A_FDSW210055 057-2r	A	708	708	1	G -> A	227	SNP (transition)	55.10%	0
Variants: VaAphrodite_A_FDSW210055 057-2r	T	723	723	1	C -> T	128	SNP (transition)	62.50%	4.10E-197
Variants: VaAphrodite_A_FDSW210055 057-2r	C	726	726	1	T -> C	113	SNP (transition)	61.90%	3.00E-186
Variants: VaAphrodite_A_FDSW210055 057-2r	AATC	738	741	4	CGCT -> AATC	97 -> 101	Substitution	32.7% -> 40.4%	5.90E-60

Variants: VaAphrodite_A_FDSW210055 057-2r	A	744	743	0	#NAME?	90	Insertion	31.10%	8.50E-34					
Variants: VaAphrodite_A_FDSW210055 057-2r	GAC	744	746	3	AGA -> GAC	93 -> 95	Substitution	84.2% -> 92.5%	1.10E-191					
Variants: VaAphrodite_A_FDSW210055 057-2r	C	750	750	1	A -> C	94	SNP (transversion)	36.20%	6.80E-84					
Variants: VaAphrodite_A_FDSW210055 057-2r	C	756	756	1	G -> C	101	SNP (transversion)	48.50%	2.30E-123					
Variants: VaAphrodite_A_FDSW210055 057-2r	A	768	768	1	T -> A	162	SNP (transversion)	52.50%	8.90E-217					
Variants: VaAphrodite_A_FDSW210055 057-2r	A	795	795	1	G -> A	329	SNP (transition)	53.50%	0	A -> T	hypothetical protein CDS	hypothetica l protein	Substituti on	KLI69 879.1
Variants: VaAphrodite_A_FDSW210055 057-2r	AAT	801	803	2	CAC -> AAT	374 -> 393	Substitution	72.2% -> 72.3%	0	H -> N	hypothetical protein CDS	hypothetica l protein	Substituti on	KLI69 879.1
Variants: VaAphrodite_A_FDSW210055 057-2r	C	834	834	1	T -> C	567	SNP (transition)	73.40%	0	Y -> H	hypothetical protein CDS	hypothetica l protein	Substituti on	KLI69 879.1
Variants: VaAphrodite_A_FDSW210055 057-2r	G	888	888	1	A -> G	781	SNP (transition)	28.30%	0	I -> V	hypothetical protein CDS	hypothetica l protein	Substituti on	KLI69 879.1
Variants: VaAphrodite_A_FDSW210055 057-2r	C	897	897	1	T -> C	796	SNP (transition)	59.70%	0	C -> R	hypothetical protein CDS	hypothetica l protein	Substituti on	KLI69 879.1
Variants: VaAphrodite_A_FDSW210055 057-2r	C	900	900	1	G -> C	800	SNP (transversion)	60.10%	0	D -> H	hypothetical protein CDS	hypothetica l protein	Substituti on	KLI69 879.1
Variants: VaAphrodite_A_FDSW210055 057-2r	C	942	942	1	G -> C	797	SNP (transversion)	31.50%	0	G -> R	hypothetical protein CDS	hypothetica l protein	Substituti on	KLI69 879.1
Variants: VaAphrodite_A_FDSW210055 057-2r	TGT	966	968	2	AGC -> TGT	775 -> 776	Substitution	49.0% -> 50.1%	0	S -> C	hypothetical protein CDS	hypothetica l protein	Substituti on	KLI69 879.1
Variants: VaAphrodite_A_FDSW210055 057-2r	G	969	969	1	C -> G	774	SNP (transversion)	35.40%	0	P -> A	hypothetical protein CDS	hypothetica l protein	Substituti on	KLI69 879.1
Variants: VaAphrodite_A_FDSW210055 057-2r	A	976	976	1	T -> A	773	SNP (transversion)	37.00%	0	V -> E	hypothetical protein CDS	hypothetica l protein	Substituti on	KLI69 879.1
Variants: VaAphrodite_A_FDSW210055 057-2r	A	1008	1008	1	G -> A	746	SNP (transition)	41.20%	0	V -> I	hypothetical protein CDS	hypothetica l protein	Substituti on	KLI69 879.1
Variants: VaAphrodite_A_FDSW210055 057-2r	C	1035	1035	1	G -> C	768	SNP (transversion)	32.00%	0	G -> R	hypothetical protein CDS	hypothetica l protein	Substituti on	KLI69 879.1
Variants: VaAphrodite_A_FDSW210055 057-2r	A	1041	1041	1	G -> A	785	SNP (transition)	39.50%	0	A -> T	hypothetical protein CDS	hypothetica l protein	Substituti on	KLI69 879.1
Variants: VaAphrodite_A_FDSW210055 057-2r	C	1049	1049	1	T -> C	772	SNP (transition)	27.20%	0		hypothetical protein CDS	hypothetica l protein	None	KLI69 879.1
Variants: VaAphrodite_A_FDSW210055 057-2r	G	1083	1083	1	A -> G	784	SNP (transition)	37.90%	0	K -> E	hypothetical protein CDS	hypothetica l protein	Substituti on	KLI69 879.1
Variants: VaAphrodite_A_FDSW210055 057-2r	A	1100	1100	1	G -> A	790	SNP (transition)	36.80%	0		hypothetical protein CDS	hypothetica l protein	None	KLI69 879.1
Variants: VaAphrodite_A_FDSW210055 057-2r	A	1101	1101	1	G -> A	799	SNP (transition)	33.00%	0					

Variants: VaAphrodite_A_FDSW210055057-2r	C	1104	1104	1	G -> C	795	SNP (transversion)	37.20%	0					
Variants: VaAphrodite_A_FDSW210055057-2r	T	1116	1116	1	C -> T	800	SNP (transition)	37.80%	0		hypothetical protein CDS	hypothetical protein	None	KLI69880.1
Variants: VaAphrodite_A_FDSW210055057-2r	T	1119	1119	1	C -> T	802	SNP (transition)	35.30%	0		hypothetical protein CDS	hypothetical protein	None	KLI69880.1
Variants: VaAphrodite_A_FDSW210055057-2r	G	1135	1135	1	T -> G	810	SNP (transversion)	55.90%	0	F -> V	hypothetical protein CDS	hypothetical protein	Substitution	KLI69880.1
Variants: VaAphrodite_A_FDSW210055057-2r	TGTG	1173	1176	3	CATA -> TGTG	864 -> 871	Substitution	42.5% -> 43.2%	0	TI -> TV	hypothetical protein CDS	hypothetical protein	Substitution	KLI69880.1
Variants: VaAphrodite_A_FDSW210055057-2r	C	1182	1182	1	T -> C	862	SNP (transition)	45.00%	0		hypothetical protein CDS	hypothetical protein	None	KLI69880.1
Variants: VaAphrodite_A_FDSW210055057-2r	G	1215	1215	1	A -> G	794	SNP (transition)	50.40%	0		hypothetical protein CDS	hypothetical protein	None	KLI69880.1
Variants: VaAphrodite_A_FDSW210055057-2r	G	1218	1218	1	A -> G	781	SNP (transition)	39.20%	0		hypothetical protein CDS	hypothetical protein	None	KLI69880.1
Variants: VaAphrodite_A_FDSW210055057-2r	G	1221	1221	1	C -> G	767	SNP (transversion)	38.30%	0		hypothetical protein CDS	hypothetical protein	None	KLI69880.1
Variants: VaAphrodite_A_FDSW210055057-2r	GG	1232	1233	2	AC -> GG	689 -> 694	Substitution	34.6% -> 34.8%	0	N -> R	hypothetical protein CDS	hypothetical protein	Substitution	KLI69880.1
Variants: VaAphrodite_A_FDSW210055057-2r	T	1235	1235	1	C -> T	676	SNP (transition)	34.80%	0	A -> V	hypothetical protein CDS	hypothetical protein	Substitution	KLI69880.1
Variants: VaAphrodite_A_FDSW210055057-2r	G	1269	1269	1	A -> G	484	SNP (transition)	61.40%	0	I -> M	hypothetical protein CDS	hypothetical protein	Substitution	KLI69880.1
Variants: VaAphrodite_A_FDSW210055057-2r	G	1275	1275	1	A -> G	450	SNP (transition)	63.60%	0		hypothetical protein CDS	hypothetical protein	None	KLI69880.1
Variants: VaAphrodite_A_FDSW210055057-2r	T	1287	1287	1	A -> T	396	SNP (transversion)	45.70%	0		hypothetical protein CDS	hypothetical protein	None	KLI69880.1
Variants: VaAphrodite_A_FDSW210055057-2r	T	1290	1290	1	C -> T	387	SNP (transition)	45.70%	0		hypothetical protein CDS	hypothetical protein	None	KLI69880.1
Variants: VaAphrodite_A_FDSW210055057-2r	G	1293	1293	1	C -> G	367	SNP (transversion)	45.00%	0	D -> E	hypothetical protein CDS	hypothetical protein	Substitution	KLI69880.1
Variants: VaAphrodite_A_FDSW210055057-2r	ATTG	1296	1299	4	CAAC -> ATTG	321 -> 342	Substitution	41.7% -> 42.4%	0	TN -> TL	hypothetical protein CDS	hypothetical protein	Substitution	KLI69880.1
Variants: VaAphrodite_A_FDSW210055057-2r	A	1305	1305	1	G -> A	289	SNP (transition)	39.10%	5.40E-291		hypothetical protein CDS	hypothetical protein	None	KLI69880.1
Variants: VaAphrodite_A_FDSW210055057-2r	GCT	1320	1322	3	TGA -> GCT	189 -> 200	Substitution	26.5% -> 29.0%	1.60E-114	TE -> TL	hypothetical protein CDS	hypothetical protein	Substitution	KLI69880.1
Variants: VaAphrodite_A_FDSW210055057-2r	AAGTT	1324	1328	4	CACAC -> AAGTT	164 -> 181	Substitution	72.4% -> 77.6%	0	HT -> KF	hypothetical protein CDS	hypothetical protein	Substitution	KLI69880.1
Variants: VaAphrodite_A_FDSW210055057-2r	AGTGCC	1330	1335	5	TTAACA -> AGTGCC	127 -> 153	Substitution	73.2% -> 80.3%	0	LT -> SA	hypothetical protein CDS	hypothetical protein	Substitution	KLI69880.1
Variants: VaAphrodite_A_FDSW210055057-2r	TCG	1339	1341	3	CGT -> TCG	100 -> 106	Substitution	54.0% -> 56.6%	2.90E-166	R -> S	hypothetical protein CDS	hypothetical protein	Substitution	KLI69880.1

Variants: VaAphrodite_A_FDSW210055 057-2r	CT	1342	1343	2	TA -> CT	95 -> 101	Substitution	51.5% -> 54.7%	2.10E-155		hypothetical protein CDS	hypothetica l protein	Extension	KLI69 880.1
Variants: VaAphrodite_A_FDSW210055 057-2r	G	1343	1343	1	A -> G	95	SNP (transition)	31.60%	4.60E-84		hypothetical protein CDS	hypothetica l protein	Extension	KLI69 880.1
Variants: VaAphrodite_A_FDSW210055 057-2r	T	1345	1345	1	G -> T	83	SNP (transversion)	62.70%	5.90E-160					
Variants: VaAphrodite_A_FDSW210055 057-2r	TT	1347	1348	2	AA -> TT	78 -> 79	Substitution	73.1% -> 77.2%	2.00E-185					
Variants: VaAphrodite_A_FDSW210055 057-2r	AAT	1351	1353	3	GGA -> AAT	64 -> 67	Substitution	67.2% -> 70.1%	2.60E-130					
Variants: VaAphrodite_A_FDSW210055 057-2r	CGTC	1355	>135 7	>3	TAA -> CGTC	55 -> 59	Insertion	54.5% -> 57.6%	3.10E-90					
Variants: VaAphrodite_A_FDSW210055 057-2r	TTA	1360	1362	2	ATG -> TTA	42 -> 47	Substitution	38.1% -> 46.8%	6.60E-44	M -> L	hypothetical protein CDS	hypothetica l protein	Start Codon Loss	KLI69 881.1
Variants: VaAphrodite_A_FDSW210055 057-2r	AA	2111	2112	2	TC -> AA	27 -> 28	Substitution	25.9% -> 28.6%	2.80E-19	I -> K	hypothetical protein CDS	hypothetica l protein	Substituti on	KLI69 881.1
Variants: VaAphrodite_A_FDSW210055 057-2r	CCGAAAC GGATGA	2114	2126	12	ATTTTGAAAAAAT -> CCGAAACGGATGA	27 -> 39	Substitution	35.7% -> 61.5%	1.30E-27	DFEKI -> AETDD	hypothetical protein CDS	hypothetica l protein	Substituti on	KLI69 881.1
Variants: VaAphrodite_A_FDSW210055 057-2r	AA	2128	2129	2	GC -> AA	40 -> 42	Substitution	64.3% -> 70.0%	1.60E-81	A -> N	hypothetical protein CDS	hypothetica l protein	Substituti on	KLI69 881.1
Variants: VaAphrodite_A_FDSW210055 057-2r	CAC	2131	2133	2	TAA -> CAC	39	Substitution	74.40%	1.60E-90		hypothetical protein CDS	hypothetica l protein	Extension	KLI69 881.1
Variants: VaAphrodite_A_FDSW210055 057-2r	T	2135	2135	1	A -> T	45	SNP (transversion)	77.80%	1.00E-113					
Variants: VaAphrodite_A_FDSW210055 057-2r	CTG	2137	2139	3	AGC -> CTG	43 -> 46	Substitution	83.7% -> 89.1%	1.90E-123					
Variants: VaAphrodite_A_FDSW210055 057-2r	AG	2141	2142	2	TT -> AG	46 -> 50	Substitution	89.1% -> 90.0%	5.40E-134					
Variants: VaAphrodite_A_FDSW210055 057-2r	G	2145	2145	1	A -> G	53	SNP (transition)	90.60%	1.80E-157					
Variants: VaAphrodite_A_FDSW210055 057-2r	T	2147	2147	1	A -> T	55	SNP (transversion)	90.90%	3.50E-169					
Variants: VaAphrodite_A_FDSW210055 057-2r	G	2149	2148	0	#NAME?	55	Insertion	92.70%	1.10E-173					
Variants: VaAphrodite_A_FDSW210055 057-2r	GAG	2902	2901	0	#NAME?	20	Insertion	100.00%	1.00E-60					
Variants: VaAphrodite_A_FDSW210055 057-2r	GACG	2904	2907	4	ATTC -> GACG	12 -> 14	Substitution	42.9% -> 64.3%	1.80E-10					
Variants: VaAphrodite_A_FDSW210055 057-2r	G	2909	2909	1	C -> G	15	SNP (transversion)	33.30%	9.10E-09					
Variants: VaAphrodite_A_FDSW210055057-2r		3097	3097	1	#NAME?	185	Deletion	41.10%	2.50E-70					
Variants: VaAphrodite_A_FDSW210055 057-2r	TAG	3450	3452	3	CGT -> TAG	15 -> 17	Substitution	26.7% -> 41.2%	8.20E-07					

Variants: VaAphrodite_A_FDSW210055057-2r	T	3659	3659	1	G -> T	200	SNP (transversion)	44.00%	2.10E-250					
Variants: VaAphrodite_A_FDSW210055057-2r	C	3758	3758	1	A -> C	183	SNP (transversion)	51.90%	6.20E-289					
Variants: VaAphrodite_A_FDSW210055057-2r	G	3778	3778	1	A -> G	190	SNP (transition)	45.80%	7.10E-241					
Variants: VaAphrodite_A_FDSW210055057-2r	T	3780	3780	1	C -> T	189	SNP (transition)	46.00%	1.50E-258					
Variants: VaAphrodite_A_FDSW210055057-2r	C	3852	3852	1	T -> C	184	SNP (transition)	52.70%	3.40E-286					
Variants: VaAphrodite_A_FDSW210055057-2r	G	3864	3864	1	A -> G	180	SNP (transition)	48.30%	2.50E-252					
Variants: VaAphrodite_A_FDSW210055057-2r	T	3922	3922	1	C -> T	139	SNP (transition)	42.40%	1.10E-143					
Variants: VaAphrodite_A_FDSW210055057-2r	T	4022	4022	1	G -> T	31	SNP (transversion)	100.00%	4.00E-75					
Variants: VaAphrodite_A_FDSW210055057-2r	A	4023	4023	1	T -> A	28	SNP (transversion)	82.10%	7.70E-58					
Variants: VaAphrodite_A_FDSW210055057-2r	T	4025	4025	1	C -> T	27	SNP (transition)	100.00%	3.20E-68					
Variants: VaAphrodite_A_FDSW210055057-2r	T	4027	4027	1	A -> T	24	SNP (transversion)	100.00%	2.50E-58					
Variants: VaAphrodite_A_FDSW210055057-2r	TTG	4031	4033	3	CCA -> TTG	20 -> 21	Substitution	100.00%	1.00E-44					
Variants: VaAphrodite_A_FDSW210055057-2r	TTT	4035	4038	4	CGGC -> TTT	18 -> 19	Deletion	84.2% -> 88.9%	1.00E-18					
Variants: VaAphrodite_A_FDSW210055057-2r	A	4040	4040	1	T -> A	18	SNP (transversion)	100.00%	1.60E-38					
Variants: VaAphrodite_A_FDSW210055057-2r	GTTTAA	4043	4048	6	ACCGTT -> GTTTAA	11 -> 16	Substitution	100.00%	5.00E-26					
Variants: VaAphrodite_A_FDSW210055057-2r	GTGGTA	4050	4055	6	CGCTGC -> GTGGTA	10 -> 11	Substitution	100.00%	1.00E-22					
Variants: VaAphrodite_A_FDSW210055057-2r	GG	4059	4060	2	TT -> GG	8 -> 9	Substitution	100.00%	4.00E-19					
Variants: VaAphrodite_A_FDSW210055057-2r	GGC	4062	4064	3	TCA -> GGC	8	Substitution	100.00%	4.00E-19					
Variants: VaAphrodite_A_FDSW210055057-2r		4067	4067	1	#NAME?	6	Deletion	100.00%	2.50E-07					
Variants: VaAphrodite_A_FDSW210055057-2r	G	4069	4069	1	C -> G	6	SNP (transversion)	100.00%	1.60E-14					
Variants: VaAphrodite_A_FDSW210055057-2r	ATT	4072	4074	3	CGC -> ATT	4	Substitution	100.00%	6.30E-10					
Variants: VaAphrodite_A_FDSW210055057-2r	GGTAG	4342	4346	4	TCGAA -> GGTAG	12 -> 25	Substitution	100.00%	4.00E-09	FD -> LP	hypothetical protein CDS	hypothetical protein	Substitution	KLI69882.1

Variants: VaAphrodite_A_FDSW210055 057-2r	TTG	<437 4	4375	>2	GA -> TTG	273 -> 294	Insertion	99.6% -> 99.7%	0		hypothetical protein CDS	hypothetica l protein	Frame Shift	KLI69 882.1
Variants: VaAphrodite_A_FDSW210055 057-2r	T	4379	4379	1	C -> T	334	SNP (transition)	100.00%	0	A -> T	hypothetical protein CDS	hypothetica l protein	Substituti on	KLI69 882.1
Variants: VaAphrodite_A_FDSW210055 057-2r	CATGA	4381	4385	5	TGGTG -> CATGA	353 -> 391	Substitution	99.70%	0		hypothetical protein CDS	hypothetica l protein	Truncatio n	KLI69 882.1
Variants: VaAphrodite_A_FDSW210055 057-2r	GG	4388	4389	2	CA -> GG	410 -> 413	Substitution	99.80%	0	PE -> PQ	hypothetical protein CDS	hypothetica l protein	Substituti on	KLI69 882.1
Variants: VaAphrodite_A_FDSW210055 057-2r	TGA	4392	4394	2	CGT -> TGA	439 -> 457	Substitution	99.80%	0	T -> S	hypothetical protein CDS	hypothetica l protein	Substituti on	KLI69 882.1
Variants: VaAphrodite_A_FDSW210055 057-2r	GT	4396	4397	2	AA -> GT	464 -> 472	Substitution	99.80%	0	F -> T	hypothetical protein CDS	hypothetica l protein	Substituti on	KLI69 882.1
Variants: VaAphrodite_A_FDSW210055 057-2r	A	4401	4401	1	C -> A	511	SNP (transversion)	100.00%	0	M -> V	hypothetical protein CDS	hypothetica l protein	Start Codon Loss	KLI69 882.1
Variants: VaAphrodite_A_FDSW210055 057-2r	A	4404	4406	3	GTT -> A	537	Deletion	99.80%	0					
Variants: VaAphrodite_A_FDSW210055 057-2r	GT	4409	4410	2	TC -> GT	552 -> 558	Substitution	99.80%	0					
Variants: VaAphrodite_A_FDSW210055 057-2r	GC	4414	4415	2	CT -> GC	581 -> 603	Substitution	99.80%	0					
Variants: VaAphrodite_A_FDSW210055 057-2r	A	4417	4417	1	G -> A	625	SNP (transition)	99.70%	0					
Variants: VaAphrodite_A_FDSW210055 057-2r	GG	4419	4420	2	CA -> GG	645 -> 663	Substitution	99.50%	0					
Variants: VaAphrodite_A_FDSW210055 057-2r	AC	4423	4424	2	GA -> AC	704 -> 711	Substitution	99.1% -> 99.2%	0					
Variants: VaAphrodite_A_FDSW210055 057-2r	G	4427	4426	0	#NAME?	740	Insertion	99.30%	0					
Variants: VaAphrodite_A_FDSW210055 057-2r	TG	4431	4432	2	GA -> TG	786 -> 794	Substitution	99.6% -> 99.9%	0					
Variants: VaAphrodite_A_FDSW210055 057-2r	A	4434	4433	0	#NAME?	812	Insertion	99.90%	0					
Variants: VaAphrodite_A_FDSW210055 057-2r	CA	4435	4436	2	GT -> CA	843 -> 856	Substitution	99.60%	0					
Variants: VaAphrodite_A_FDSW210055 057-2r	A	4973	4973	1	G -> A	195	SNP (transition)	74.40%	0					
Variants: VaAphrodite_A_FDSW210055 057-2r	C	4975	4974	0	+C	183	Insertion	74.30%	0					
Variants: VaAphrodite_A_FDSW210055 057-2r	G	4975	4974	0	#NAME?	179	Insertion	49.20%	1.70E-238					
Variants: VaAphrodite_A_FDSW210055 057-2r	T	4975	4974	0	(T)3 -> (T)4	179	Insertion (tandem repeat)	25.70%	1.20E-95					
Variants: VaAphrodite_A_FDSW210055 057-2r	A	4975	4975	1	T -> A	180	SNP (transversion)	48.30%	2.50E-252					

Variants: VaAphrodite_A_FDSW210055057-2r	C	4975	4975	1	T -> C	180	SNP (transition)	25.00%	5.90E-111
Variants: VaAphrodite_A_FDSW210055057-2r	A	4976	4976	1	T -> A	176	SNP (transversion)	46.60%	2.40E-244
Variants: VaAphrodite_A_FDSW210055057-2r	G	4978	4978	1	A -> G	163	SNP (transition)	46.00%	4.20E-208
Variants: VaAphrodite_A_FDSW210055057-2r	A	4979	4979	1	G -> A	159	SNP (transition)	70.40%	0
Variants: VaAphrodite_A_FDSW210055057-2r	T	4980	4980	1	A -> T	158	SNP (transversion)	46.20%	4.50E-210
Variants: VaAphrodite_A_FDSW210055057-2r	A	4981	4981	1	G -> A	149	SNP (transition)	71.10%	8.5E-313
Variants: VaAphrodite_A_FDSW210055057-2r	A	4982	4982	1	T -> A	148	SNP (transversion)	70.90%	3.8E-320
Variants: VaAphrodite_A_FDSW210055057-2r	A	4983	4983	1	G -> A	144	SNP (transition)	91.70%	0
Variants: VaAphrodite_A_FDSW210055057-2r	C	4984	4984	1	A -> C	146	SNP (transversion)	45.90%	5.50E-186
Variants: VaAphrodite_A_FDSW210055057-2r	AC	4985	4986	2	TT -> AC	141 -> 145	Substitution	66.7% -> 66.9%	1.00E-276
Variants: VaAphrodite_A_FDSW210055057-2r	A	4988	4988	1	G -> A	135	SNP (transition)	44.40%	1.30E-159
Variants: VaAphrodite_A_FDSW210055057-2r	C	4988	4988	1	G -> C	135	SNP (transversion)	45.20%	7.70E-163
Variants: VaAphrodite_A_FDSW210055057-2r	A	4989	4989	1	C -> A	131	SNP (transversion)	64.90%	1.70E-245
Variants: VaAphrodite_A_FDSW210055057-2r	AA	4992	4993	2	CG -> AA	116 -> 121	Substitution	86.2% -> 87.6%	1.7E-311
Variants: VaAphrodite_A_FDSW210055057-2r	A	4994	4994	1	C -> A	115	SNP (transversion)	40.90%	8.60E-114
Variants: VaAphrodite_A_FDSW210055057-2r	ACA	4995	4997	3	GTG -> ACA	100 -> 109	Substitution	55.0% -> 60.6%	1.20E-173
Variants: VaAphrodite_A_FDSW210055057-2r	T	4998	4998	1	G -> T	99	SNP (transversion)	35.40%	6.90E-93
Variants: VaAphrodite_A_FDSW210055057-2r	T	4999	4999	1	C -> T	94	SNP (transition)	54.30%	4.50E-147
Variants: VaAphrodite_A_FDSW210055057-2r	C	5000	5000	1	A -> C	90	SNP (transversion)	32.20%	1.00E-78
Variants: VaAphrodite_A_FDSW210055057-2r	T	5000	5000	1	A -> T	90	SNP (transversion)	38.90%	1.10E-94
Variants: VaAphrodite_A_FDSW210055057-2r	A	5001	5001	1	T -> A	84	SNP (transversion)	28.60%	2.40E-54
Variants: VaAphrodite_A_FDSW210055057-2r	A	5002	5002	1	T -> A	85	SNP (transversion)	47.10%	2.80E-104

Variants: VaAphrodite_A_FDSW210055 057-2r	A	5003	5003	1	T -> A	80	SNP (transversion)	38.80%	7.00E-81
Variants: VaAphrodite_A_FDSW210055 057-2r	G	5005	5005	1	T -> G	71	SNP (transversion)	36.60%	1.70E-72
Variants: VaAphrodite_A_FDSW210055 057-2r	G	5006	5006	1	A -> G	64	SNP (transition)	26.60%	1.10E-41
Variants: VaAphrodite_A_FDSW210055 057-2r	T	5006	5006	1	A -> T	64	SNP (transversion)	31.30%	1.90E-54
Variants: VaAphrodite_A_FDSW210055 057-2r	T	5007	5007	1	C -> T	58	SNP (transition)	31.00%	2.80E-47
Variants: VaAphrodite_A_FDSW210055 057-2r	T	5011	5011	1	G -> T	52	SNP (transversion)	38.50%	1.20E-50
Variants: VaAphrodite_A_FDSW210055 057-2r	C	5579	5579	1	G -> C	45	SNP (transversion)	100.00%	3.20E-149
Variants: VaAphrodite_A_FDSW210055 057-2r	T	5581	5581	1	A -> T	44	SNP (transversion)	100.00%	1.60E-141
Variants: VaAphrodite_A_FDSW210055 057-2r	CG	5583	5584	2	TA -> CG	41 -> 42	Substitution	100.00%	4.00E-140
Variants: VaAphrodite_A_FDSW210055 057-2r	TC	5606	5607	2	AA -> TC	29 -> 32	Substitution	27.6% -> 34.4%	2.10E-09
Variants: VaAphrodite_A_FDSW210055 057-2r	TT	5609	5610	2	AA -> TT	39 -> 41	Substitution	51.3% -> 53.7%	6.00E-32
Variants: VaAphrodite_A_FDSW210055 057-2r	AA	5612	5613	2	GC -> AA	43 -> 44	Substitution	55.8% -> 59.1%	6.70E-37
Variants: VaAphrodite_A_FDSW210055 057-2r	GGG	5615	5617	3	TTA -> GGG	42 -> 47	Substitution	63.6% -> 66.0%	1.80E-46
Variants: VaAphrodite_A_FDSW210055 057-2r	A	5618	5618	1	G -> A	49	SNP (transition)	67.30%	1.50E-57
Variants: VaAphrodite_A_FDSW210055 057-2r	T	5618	5618	1	G -> T	49	SNP (transversion)	28.60%	6.50E-31
Variants: VaAphrodite_A_FDSW210055 057-2r	GT	5619	5620	2	TC -> GT	51	Substitution	96.10%	3.20E-115
Variants: VaAphrodite_A_FDSW210055 057-2r	C	5621	5621	1	G -> C	49	SNP (transversion)	75.50%	3.40E-71
Variants: VaAphrodite_A_FDSW210055 057-2r	T	5622	5622	1	C -> T	50	SNP (transition)	96.00%	7.70E-113
Variants: VaAphrodite_A_FDSW210055 057-2r	G	5623	5623	1	C -> G	51	SNP (transversion)	72.50%	4.70E-70
Variants: VaAphrodite_A_FDSW210055 057-2r	AA	5624	5625	2	TT -> AA	49 -> 52	Substitution	88.5% -> 93.9%	2.90E-102
Variants: VaAphrodite_A_FDSW210055 057-2r	A	5626	5626	1	G -> A	52	SNP (transition)	73.10%	2.50E-68
Variants: VaAphrodite_A_FDSW210055 057-2r	T	5632	5632	1	A -> T	56	SNP (transversion)	83.90%	1.40E-89

Variants: VaAphrodite_A_FDSW210055 057-2r	TATG	5633	5636	4	ATCA -> TATG	57 -> 58	Substitution	65.5% -> 66.7%	4.90E-69
Variants: VaAphrodite_A_FDSW210055 057-2r	AA	5637	5638	2	TT -> AA	61	Substitution	77.00%	1.50E-93
Variants: VaAphrodite_A_FDSW210055 057-2r	AG	5645	5646	2	TC -> AG	68 -> 70	Substitution	74.3% -> 76.5%	5.20E-108
Variants: VaAphrodite_A_FDSW210055057-2r		5648	5648	1	(C)3 -> (C)2	80	Deletion (tandem repeat)	63.70%	3.90E-36
Variants: VaAphrodite_A_FDSW210055 057-2r	T	5653	5653	1	G -> T	110	SNP (transversion)	58.20%	1.10E-116
Variants: VaAphrodite_A_FDSW210055 057-2r	GC	5658	5657	0	#NAME?	132	Insertion	46.20%	9.10E-103
Variants: VaAphrodite_B_FDSW210055 058-2r	TGGT	79	82	4	CCTG -> TGGT	58 -> 61	Substitution	91.4% -> 94.9%	8.60E-95
Variants: VaAphrodite_B_FDSW210055 058-2r	G	84	84	1	A -> G	50	SNP (transition)	96.00%	1.20E-93
Variants: VaAphrodite_B_FDSW210055 058-2r	GGGG	86	89	4	CATC -> GGGG	47 -> 49	Substitution	70.2% -> 83.0%	1.10E-48
Variants: VaAphrodite_B_FDSW210055 058-2r	C	91	91	1	T -> C	46	SNP (transition)	65.20%	8.10E-46
Variants: VaAphrodite_B_FDSW210055 058-2r	GGA	93	95	3	CAC -> GGA	41 -> 45	Substitution	36.6% -> 44.4%	1.20E-15
Variants: VaAphrodite_B_FDSW210055 058-2r	T	97	97	1	A -> T	43	SNP (transversion)	27.90%	1.20E-14
Variants: VaAphrodite_B_FDSW210055 058-2r	C	102	102	1	T -> C	52	SNP (transition)	57.70%	1.60E-34
Variants: VaAphrodite_B_FDSW210055 058-2r	G	120	120	1	A -> G	131	SNP (transition)	40.50%	4.70E-44
Variants: VaAphrodite_B_FDSW210055 058-2r	T	126	126	1	C -> T	170	SNP (transition)	41.20%	2.70E-58
Variants: VaAphrodite_B_FDSW210055 058-2r	T	129	129	1	C -> T	185	SNP (transition)	78.90%	5.00E-209
Variants: VaAphrodite_B_FDSW210055 058-2r	G	132	132	1	C -> G	200	SNP (transversion)	40.50%	7.60E-114
Variants: VaAphrodite_B_FDSW210055 058-2r	T	144	144	1	C -> T	246	SNP (transition)	40.70%	4.20E-101
Variants: VaAphrodite_B_FDSW210055 058-2r	T	147	147	1	C -> T	257	SNP (transition)	34.20%	2.00E-56
Variants: VaAphrodite_B_FDSW210055 058-2r	G	150	150	1	C -> G	264	SNP (transversion)	25.40%	7.10E-72
Variants: VaAphrodite_B_FDSW210055 058-2r	A	159	159	1	G -> A	288	SNP (transition)	79.50%	0
Variants: VaAphrodite_B_FDSW210055 058-2r	A	165	165	1	T -> A	308	SNP (transversion)	47.70%	1.10E-189

Variants: VaAphrodite_B_FDSW210055 058-2r	A	183	183	1	G -> A	361	SNP (transition)	38.00%	5.50E-173
Variants: VaAphrodite_B_FDSW210055 058-2r	A	192	192	1	G -> A	393	SNP (transition)	35.10%	4.70E-278
Variants: VaAphrodite_B_FDSW210055 058-2r	A	201	201	1	G -> A	421	SNP (transition)	26.80%	2.70E-156
Variants: VaAphrodite_B_FDSW210055 058-2r	G	216	216	1	A -> G	469	SNP (transition)	27.70%	3.00E-220
Variants: VaAphrodite_B_FDSW210055 058-2r	T	225	225	1	A -> T	522	SNP (transversion)	48.30%	1.20E-300
Variants: VaAphrodite_B_FDSW210055 058-2r	C	237	237	1	T -> C	547	SNP (transition)	33.50%	5.00E-165
Variants: VaAphrodite_B_FDSW210055 058-2r	C	240	240	1	T -> C	545	SNP (transition)	42.20%	9.60E-280
Variants: VaAphrodite_B_FDSW210055 058-2r	A	294	294	1	G -> A	539	SNP (transition)	39.70%	0
Variants: VaAphrodite_B_FDSW210055 058-2r	A	357	357	1	G -> A	745	SNP (transition)	54.60%	0
Variants: VaAphrodite_B_FDSW210055 058-2r	G	363	363	1	A -> G	764	SNP (transition)	56.20%	0
Variants: VaAphrodite_B_FDSW210055 058-2r	C	369	369	1	T -> C	779	SNP (transition)	79.50%	0
Variants: VaAphrodite_B_FDSW210055 058-2r	A	371	371	1	G -> A	786	SNP (transition)	49.70%	0
Variants: VaAphrodite_B_FDSW210055 058-2r	G	387	387	1	C -> G	846	SNP (transversion)	54.60%	0
Variants: VaAphrodite_B_FDSW210055 058-2r	C	393	393	1	G -> C	857	SNP (transversion)	49.50%	0
Variants: VaAphrodite_B_FDSW210055 058-2r	C	396	396	1	A -> C	869	SNP (transversion)	32.60%	0
Variants: VaAphrodite_B_FDSW210055 058-2r	G	420	420	1	A -> G	942	SNP (transition)	30.30%	0
Variants: VaAphrodite_B_FDSW210055 058-2r	T	420	420	1	A -> T	942	SNP (transversion)	33.30%	0
Variants: VaAphrodite_B_FDSW210055 058-2r	A	422	422	1	T -> A	933	SNP (transversion)	33.20%	0
Variants: VaAphrodite_B_FDSW210055 058-2r	A	423	423	1	G -> A	936	SNP (transition)	61.90%	0
Variants: VaAphrodite_B_FDSW210055 058-2r	A	426	426	1	G -> A	939	SNP (transition)	61.10%	0
Variants: VaAphrodite_B_FDSW210055 058-2r	G	438	438	1	A -> G	965	SNP (transition)	45.80%	0
Variants: VaAphrodite_B_FDSW210055 058-2r	A	456	456	1	G -> A	975	SNP (transition)	48.10%	0

Variants: VaAphrodite_B_FDSW210055 058-2r	C	489	489	1	T -> C	918	SNP (transition)	46.30%	0
Variants: VaAphrodite_B_FDSW210055 058-2r	C	498	498	1	G -> C	887	SNP (transversion)	30.30%	0
Variants: VaAphrodite_B_FDSW210055 058-2r	C	513	513	1	T -> C	824	SNP (transition)	47.90%	0
Variants: VaAphrodite_B_FDSW210055 058-2r	C	516	516	1	T -> C	825	SNP (transition)	32.10%	0
Variants: VaAphrodite_B_FDSW210055 058-2r	T	531	531	1	C -> T	798	SNP (transition)	29.20%	0
Variants: VaAphrodite_B_FDSW210055 058-2r	C	537	537	1	G -> C	776	SNP (transversion)	55.40%	0
Variants: VaAphrodite_B_FDSW210055 058-2r	G	540	540	1	A -> G	777	SNP (transition)	34.60%	0
Variants: VaAphrodite_B_FDSW210055 058-2r	A	552	552	1	T -> A	752	SNP (transversion)	37.90%	0
Variants: VaAphrodite_B_FDSW210055 058-2r	A	564	564	1	C -> A	715	SNP (transversion)	28.50%	0
Variants: VaAphrodite_B_FDSW210055 058-2r	G	588	588	1	A -> G	660	SNP (transition)	35.80%	0
Variants: VaAphrodite_B_FDSW210055 058-2r	C	663	663	1	T -> C	421	SNP (transition)	75.30%	0
Variants: VaAphrodite_B_FDSW210055 058-2r	G	677	677	1	A -> G	341	SNP (transition)	77.10%	0
Variants: VaAphrodite_B_FDSW210055 058-2r	C	684	684	1	T -> C	314	SNP (transition)	74.50%	0
Variants: VaAphrodite_B_FDSW210055 058-2r	A	687	687	1	G -> A	300	SNP (transition)	75.00%	0
Variants: VaAphrodite_B_FDSW210055 058-2r	T	690	690	1	A -> T	287	SNP (transversion)	77.70%	0
Variants: VaAphrodite_B_FDSW210055 058-2r	A	693	693	1	G -> A	281	SNP (transition)	76.90%	0
Variants: VaAphrodite_B_FDSW210055 058-2r	A	708	708	1	G -> A	213	SNP (transition)	74.20%	0
Variants: VaAphrodite_B_FDSW210055 058-2r	T	723	723	1	C -> T	160	SNP (transition)	78.80%	0
Variants: VaAphrodite_B_FDSW210055 058-2r	C	726	726	1	T -> C	146	SNP (transition)	79.50%	0
Variants: VaAphrodite_B_FDSW210055 058-2r	C	747	747	1	T -> C	105	SNP (transition)	88.60%	1.90E-264
Variants: VaAphrodite_B_FDSW210055 058-2r	CGTTCGA	749	755	7	TACCTTG -> CGTTCGA	92 -> 105	Substitution	34.8% -> 59.4%	7.30E-65
Variants: VaAphrodite_B_FDSW210055 058-2r	C	756	756	1	G -> C	91	SNP (transversion)	40.70%	2.40E-71

Variants: VaAphrodite_B_FDSW210055058-2r	T	756	756	1	G -> T	91	SNP (transversion)	34.10%	3.50E-60					
Variants: VaAphrodite_B_FDSW210055058-2r	AG	757	758	2	TC -> AG	91 -> 95	Substitution	25.3% -> 28.6%	3.70E-36					
Variants: VaAphrodite_B_FDSW210055058-2r	T	765	765	1	C -> T	114	SNP (transition)	36.80%	1.90E-49					
Variants: VaAphrodite_B_FDSW210055058-2r	A	768	768	1	T -> A	118	SNP (transversion)	50.00%	1.80E-137					
Variants: VaAphrodite_B_FDSW210055058-2r	T	790	790	1	G -> T	217	SNP (transversion)	27.20%	4.20E-112	R -> L	hypothetical protein CDS	hypothetical protein	Substitution	KLI69879.1
Variants: VaAphrodite_B_FDSW210055058-2r	A	795	795	1	G -> A	236	SNP (transition)	53.40%	6.40E-259	A -> T	hypothetical protein CDS	hypothetical protein	Substitution	KLI69879.1
Variants: VaAphrodite_B_FDSW210055058-2r	AAT	801	803	2	CAC -> AAT	256 -> 265	Substitution	75.8% -> 76.2%	0	H -> N	hypothetical protein CDS	hypothetical protein	Substitution	KLI69879.1
Variants: VaAphrodite_B_FDSW210055058-2r	C	834	834	1	T -> C	386	SNP (transition)	76.20%	0	Y -> H	hypothetical protein CDS	hypothetical protein	Substitution	KLI69879.1
Variants: VaAphrodite_B_FDSW210055058-2r	G	888	888	1	A -> G	549	SNP (transition)	29.70%	8.4E-314	I -> V	hypothetical protein CDS	hypothetical protein	Substitution	KLI69879.1
Variants: VaAphrodite_B_FDSW210055058-2r	C	897	897	1	T -> C	558	SNP (transition)	58.60%	0	C -> R	hypothetical protein CDS	hypothetical protein	Substitution	KLI69879.1
Variants: VaAphrodite_B_FDSW210055058-2r	C	900	900	1	G -> C	561	SNP (transversion)	58.30%	0	D -> H	hypothetical protein CDS	hypothetical protein	Substitution	KLI69879.1
Variants: VaAphrodite_B_FDSW210055058-2r	C	942	942	1	G -> C	554	SNP (transversion)	30.50%	3.40E-277	G -> R	hypothetical protein CDS	hypothetical protein	Substitution	KLI69879.1
Variants: VaAphrodite_B_FDSW210055058-2r	TGT	966	968	2	AGC -> TGT	531 -> 533	Substitution	45.2% -> 45.4%	0	S -> C	hypothetical protein CDS	hypothetical protein	Substitution	KLI69879.1
Variants: VaAphrodite_B_FDSW210055058-2r	G	969	969	1	C -> G	537	SNP (transversion)	28.30%	1.20E-198	P -> A	hypothetical protein CDS	hypothetical protein	Substitution	KLI69879.1
Variants: VaAphrodite_B_FDSW210055058-2r	A	976	976	1	T -> A	536	SNP (transversion)	29.70%	3.00E-180	V -> E	hypothetical protein CDS	hypothetical protein	Substitution	KLI69879.1
Variants: VaAphrodite_B_FDSW210055058-2r	A	1008	1008	1	G -> A	529	SNP (transition)	33.80%	1.20E-214	V -> I	hypothetical protein CDS	hypothetical protein	Substitution	KLI69879.1
Variants: VaAphrodite_B_FDSW210055058-2r	A	1041	1041	1	G -> A	540	SNP (transition)	33.50%	1.30E-287	A -> T	hypothetical protein CDS	hypothetical protein	Substitution	KLI69879.1
Variants: VaAphrodite_B_FDSW210055058-2r	G	1083	1083	1	A -> G	560	SNP (transition)	36.10%	0	K -> E	hypothetical protein CDS	hypothetical protein	Substitution	KLI69879.1
Variants: VaAphrodite_B_FDSW210055058-2r	A	1100	1100	1	G -> A	588	SNP (transition)	38.90%	0		hypothetical protein CDS	hypothetical protein	None	KLI69879.1
Variants: VaAphrodite_B_FDSW210055058-2r	A	1101	1101	1	G -> A	594	SNP (transition)	34.70%	0					
Variants: VaAphrodite_B_FDSW210055058-2r	C	1104	1104	1	G -> C	604	SNP (transversion)	39.70%	0					
Variants: VaAphrodite_B_FDSW210055058-2r	T	1116	1116	1	C -> T	616	SNP (transition)	39.80%	0		hypothetical protein CDS	hypothetical protein	None	KLI69880.1

Variants: VaAphrodite_B_FDSW210055 058-2r	T	1119	1119	1	C -> T	616	SNP (transition)	37.20%	0		hypothetical protein CDS	hypothetica l protein	None	KLI69 880.1
Variants: VaAphrodite_B_FDSW210055 058-2r	G	1135	1135	1	T -> G	609	SNP (transversion)	57.10%	0	F -> V	hypothetical protein CDS	hypothetica l protein	Substituti on	KLI69 880.1
Variants: VaAphrodite_B_FDSW210055 058-2r	TGTG	1173	1176	3	CATA -> TGTG	654 -> 662	Substitution	44.6% -> 45.2%	0	TI -> TV	hypothetical protein CDS	hypothetica l protein	Substituti on	KLI69 880.1
Variants: VaAphrodite_B_FDSW210055 058-2r	C	1182	1182	1	T -> C	664	SNP (transition)	46.20%	0		hypothetical protein CDS	hypothetica l protein	None	KLI69 880.1
Variants: VaAphrodite_B_FDSW210055 058-2r	G	1215	1215	1	A -> G	576	SNP (transition)	51.90%	0		hypothetical protein CDS	hypothetica l protein	None	KLI69 880.1
Variants: VaAphrodite_B_FDSW210055 058-2r	G	1218	1218	1	A -> G	559	SNP (transition)	41.10%	0		hypothetical protein CDS	hypothetica l protein	None	KLI69 880.1
Variants: VaAphrodite_B_FDSW210055 058-2r	G	1221	1221	1	C -> G	548	SNP (transversion)	40.50%	0		hypothetical protein CDS	hypothetica l protein	None	KLI69 880.1
Variants: VaAphrodite_B_FDSW210055 058-2r	GG	1232	1233	2	AC -> GG	500 -> 507	Substitution	37.7% -> 38.2%	0	N -> R	hypothetical protein CDS	hypothetica l protein	Substituti on	KLI69 880.1
Variants: VaAphrodite_B_FDSW210055 058-2r	T	1235	1235	1	C -> T	488	SNP (transition)	37.10%	0	A -> V	hypothetical protein CDS	hypothetica l protein	Substituti on	KLI69 880.1
Variants: VaAphrodite_B_FDSW210055 058-2r	G	1269	1269	1	A -> G	324	SNP (transition)	66.70%	0	I -> M	hypothetical protein CDS	hypothetica l protein	Substituti on	KLI69 880.1
Variants: VaAphrodite_B_FDSW210055 058-2r	G	1275	1275	1	A -> G	311	SNP (transition)	68.20%	0		hypothetical protein CDS	hypothetica l protein	None	KLI69 880.1
Variants: VaAphrodite_B_FDSW210055 058-2r	T	1287	1287	1	A -> T	277	SNP (transversion)	49.80%	0		hypothetical protein CDS	hypothetica l protein	None	KLI69 880.1
Variants: VaAphrodite_B_FDSW210055 058-2r	T	1290	1290	1	C -> T	272	SNP (transition)	49.30%	0		hypothetical protein CDS	hypothetica l protein	None	KLI69 880.1
Variants: VaAphrodite_B_FDSW210055 058-2r	G	1293	1293	1	C -> G	256	SNP (transversion)	49.20%	0	D -> E	hypothetical protein CDS	hypothetica l protein	Substituti on	KLI69 880.1
Variants: VaAphrodite_B_FDSW210055 058-2r	ATTG	1296	1299	4	CAAC -> ATTG	230 -> 243	Substitution	48.7% -> 49.8%	1.00E-307	TN -> TL	hypothetical protein CDS	hypothetica l protein	Substituti on	KLI69 880.1
Variants: VaAphrodite_B_FDSW210055 058-2r	G	1302	1302	1	A -> G	219	SNP (transition)	25.60%	1.80E-149		hypothetical protein CDS	hypothetica l protein	None	KLI69 880.1
Variants: VaAphrodite_B_FDSW210055 058-2r	A	1305	1305	1	G -> A	204	SNP (transition)	46.60%	8.30E-245		hypothetical protein CDS	hypothetica l protein	None	KLI69 880.1
Variants: VaAphrodite_B_FDSW210055 058-2r	A	1311	1311	1	G -> A	182	SNP (transition)	30.80%	2.30E-132		hypothetical protein CDS	hypothetica l protein	None	KLI69 880.1
Variants: VaAphrodite_B_FDSW210055 058-2r	T	1314	1314	1	G -> T	160	SNP (transversion)	28.70%	7.70E-103		hypothetical protein CDS	hypothetica l protein	None	KLI69 880.1
Variants: VaAphrodite_B_FDSW210055 058-2r	G	1317	1317	1	C -> G	144	SNP (transversion)	28.50%	9.90E-96	D -> E	hypothetical protein CDS	hypothetica l protein	Substituti on	KLI69 880.1
Variants: VaAphrodite_B_FDSW210055 058-2r	GCTA	1320	1323	4	TGAG -> GCTA	120 -> 134	Substitution	30.0% -> 33.6%	7.60E-71	TE -> TL	hypothetical protein CDS	hypothetica l protein	Substituti on	KLI69 880.1
Variants: VaAphrodite_B_FDSW210055 058-2r	A	1324	1324	1	C -> A	117	SNP (transversion)	34.20%	4.10E-127	H -> N	hypothetical protein CDS	hypothetica l protein	Substituti on	KLI69 880.1

Variants: VaAphrodite_B_FDSW210055 058-2r	AAGTT	1324	1328	4	CACAC -> AAGTT	107 -> 117	Substitution	64.1% -> 70.1%	1.90E-228	HT -> KF	hypothetical protein CDS	hypothetica l protein	Substituti on	KLI69 880.1
Variants: VaAphrodite_B_FDSW210055 058-2r	AGTGCC	1330	1335	5	TTAACA -> AGTGCC	79 -> 102	Substitution	65.8% -> 72.5%	6.40E-167	LT -> SA	hypothetical protein CDS	hypothetica l protein	Substituti on	KLI69 880.1
Variants: VaAphrodite_B_FDSW210055 058-2r	TCG	1339	1341	3	CGT -> TCG	71 -> 77	Substitution	36.6% -> 42.9%	3.70E-81	R -> S	hypothetical protein CDS	hypothetica l protein	Substituti on	KLI69 880.1
Variants: VaAphrodite_B_FDSW210055 058-2r	CT	1342	1343	2	TA -> CT	61 -> 67	Substitution	35.8% -> 39.3%	5.90E-68		hypothetical protein CDS	hypothetica l protein	Extension	KLI69 880.1
Variants: VaAphrodite_B_FDSW210055 058-2r	T	1345	1345	1	G -> T	58	SNP (transversion)	41.40%	1.30E-68					
Variants: VaAphrodite_B_FDSW210055 058-2r	TT	1347	1348	2	AA -> TT	50 -> 52	Substitution	48.0% -> 53.8%	4.80E-73					
Variants: VaAphrodite_B_FDSW210055 058-2r	AAT	1351	1353	3	GGA -> AAT	44 -> 46	Substitution	33.3% -> 37.0%	1.00E-41					
Variants: VaAphrodite_B_FDSW210055 058-2r	CGT	1355	1357	3	TAA -> CGT	44 -> 45	Substitution	24.4% -> 28.9%	1.80E-27					
Variants: VaAphrodite_B_FDSW210055 058-2r	A	2111	2111	1	T -> A	30	SNP (transversion)	33.30%	3.00E-26	I -> N	hypothetical protein CDS	hypothetica l protein	Substituti on	KLI69 881.1
Variants: VaAphrodite_B_FDSW210055 058-2r	C	2114	2114	1	A -> C	32	SNP (transversion)	37.50%	8.90E-31	D -> A	hypothetical protein CDS	hypothetica l protein	Substituti on	KLI69 881.1
Variants: VaAphrodite_B_FDSW210055 058-2r	AA	2117	2118	2	TT -> AA	33 -> 34	Substitution	45.5% -> 47.1%	3.30E-44		hypothetical protein CDS	hypothetica l protein	Truncatio n	KLI69 881.1
Variants: VaAphrodite_B_FDSW210055 058-2r	ACG	2119	2121	3	GAA -> ACG	35 -> 36	Substitution	27.8% -> 28.6%	1.80E-26	E -> T	hypothetical protein CDS	hypothetica l protein	Substituti on	KLI69 881.1
Variants: VaAphrodite_B_FDSW210055 058-2r	GATGA	2122	2126	4	AAAAT -> GATGA	37 -> 38	Substitution	36.8% -> 40.5%	4.80E-37	KI -> DD	hypothetical protein CDS	hypothetica l protein	Substituti on	KLI69 881.1
Variants: VaAphrodite_B_FDSW210055 058-2r	AA	2128	2129	2	GC -> AA	42	Substitution	45.2% -> 47.6%	5.10E-55	A -> N	hypothetical protein CDS	hypothetica l protein	Substituti on	KLI69 881.1
Variants: VaAphrodite_B_FDSW210055 058-2r	CAC	2131	2133	2	TAA -> CAC	39 -> 41	Substitution	51.2% -> 53.8%	8.50E-63		hypothetical protein CDS	hypothetica l protein	Extension	KLI69 881.1
Variants: VaAphrodite_B_FDSW210055 058-2r	T	2135	2135	1	A -> T	41	SNP (transversion)	58.50%	2.40E-66					
Variants: VaAphrodite_B_FDSW210055 058-2r	CT	2137	2138	2	AG -> CT	45	Substitution	57.80%	1.50E-71					
Variants: VaAphrodite_B_FDSW210055 058-2r	A	2139	2139	1	C -> A	47	SNP (transversion)	29.80%	1.30E-39					
Variants: VaAphrodite_B_FDSW210055 058-2r	G	2139	2139	1	C -> G	47	SNP (transversion)	55.30%	1.20E-78					
Variants: VaAphrodite_B_FDSW210055 058-2r	A	2141	2141	1	T -> A	51	SNP (transversion)	51.00%	9.60E-62					
Variants: VaAphrodite_B_FDSW210055 058-2r	G	2141	2141	1	T -> G	51	SNP (transversion)	37.30%	1.50E-53					
Variants: VaAphrodite_B_FDSW210055 058-2r	G	2142	2142	1	T -> G	51	SNP (transversion)	52.90%	1.80E-75					

Variants: VaAphrodite_B_FDSW210055058-2r	AG	2143	2144	2	TA -> AG	52 -> 53	Substitution	39.6% -> 40.4%	6.00E-60
Variants: VaAphrodite_B_FDSW210055058-2r	G	2145	2145	1	A -> G	52	SNP (transition)	90.40%	4.10E-154
Variants: VaAphrodite_B_FDSW210055058-2r	T	2146	2146	1	G -> T	53	SNP (transversion)	43.40%	9.80E-69
Variants: VaAphrodite_B_FDSW210055058-2r	G	2147	2147	1	A -> G	54	SNP (transition)	44.40%	1.40E-69
Variants: VaAphrodite_B_FDSW210055058-2r	T	2147	2147	1	A -> T	54	SNP (transversion)	48.10%	7.40E-74
Variants: VaAphrodite_B_FDSW210055058-2r	G	2149	2148	0	#NAME?	53	Insertion	49.10%	1.50E-71
Variants: VaAphrodite_B_FDSW210055058-2r	A	2897	2897	1	T -> A	24	SNP (transversion)	25.00%	7.70E-09
Variants: VaAphrodite_B_FDSW210055058-2r	GAG	2902	2904	3	TGA -> GAG	19	Substitution	52.60%	8.50E-16
Variants: VaAphrodite_B_FDSW210055058-2r	GG	2906	2907	2	TC -> GG	17 -> 18	Substitution	41.2% -> 44.4%	2.10E-08
Variants: VaAphrodite_B_FDSW210055058-2r		3097	3097	1	#NAME?	204	Deletion	42.60%	5.30E-82
Variants: VaAphrodite_B_FDSW210055058-2r	G	3452	3452	1	T -> G	13	SNP (transversion)	46.20%	2.50E-08
Variants: VaAphrodite_B_FDSW210055058-2r	G	3456	3456	1	T -> G	14	SNP (transversion)	35.70%	1.90E-08
Variants: VaAphrodite_B_FDSW210055058-2r	T	3659	3659	1	G -> T	200	SNP (transversion)	50.50%	2.70E-295
Variants: VaAphrodite_B_FDSW210055058-2r	C	3758	3758	1	A -> C	189	SNP (transversion)	52.40%	1.10E-291
Variants: VaAphrodite_B_FDSW210055058-2r	G	3778	3778	1	A -> G	201	SNP (transition)	43.30%	4.50E-238
Variants: VaAphrodite_B_FDSW210055058-2r	T	3780	3780	1	C -> T	200	SNP (transition)	44.00%	2.10E-250
Variants: VaAphrodite_B_FDSW210055058-2r	C	3852	3852	1	T -> C	189	SNP (transition)	53.40%	9.00E-299
Variants: VaAphrodite_B_FDSW210055058-2r	G	3864	3864	1	A -> G	187	SNP (transition)	46.00%	2.40E-238
Variants: VaAphrodite_B_FDSW210055058-2r	T	3922	3922	1	C -> T	141	SNP (transition)	44.00%	1.50E-133
Variants: VaAphrodite_B_FDSW210055058-2r	A	4023	4023	1	T -> A	33	SNP (transversion)	57.60%	2.00E-37
Variants: VaAphrodite_B_FDSW210055058-2r	G	4023	4023	1	T -> G	33	SNP (transversion)	42.40%	4.60E-13
Variants: VaAphrodite_B_FDSW210055058-2r	C	4029	4029	1	T -> C	28	SNP (transition)	39.30%	4.80E-08

Variants: VaAphrodite_B_FDSW210055 058-2r	TTG	4031	4033	3	CCA -> TTG	27 -> 28	Substitution	100.00%	5.00E-52					
Variants: VaAphrodite_B_FDSW210055 058-2r	T	4035	4035	1	C -> T	26	SNP (transition)	65.40%	2.40E-33					
Variants: VaAphrodite_B_FDSW210055 058-2r	T	4036	4036	1	G -> T	25	SNP (transversion)	100.00%	1.00E-45					
Variants: VaAphrodite_B_FDSW210055 058-2r	T	4037	4037	1	G -> T	24	SNP (transversion)	62.50%	1.20E-27					
Variants: VaAphrodite_B_FDSW210055 058-2r	TA	4038	4039	2	CT -> TA	23 -> 24	Substitution	100.00%	7.90E-40					
Variants: VaAphrodite_B_FDSW210055 058-2r	G	4040	4040	1	T -> G	22	SNP (transversion)	63.60%	4.80E-26					
Variants: VaAphrodite_B_FDSW210055 058-2r	TGTTTAAG	4041	4048	8	GTACCGTT -> TGTTTAAG	17 -> 22	Substitution	94.1% -> 95.5%	5.00E-33					
Variants: VaAphrodite_B_FDSW210055 058-2r	T	4050	4050	1	C -> T	15	SNP (transition)	100.00%	1.00E-30					
Variants: VaAphrodite_B_FDSW210055 058-2r	G	4052	4052	1	C -> G	14	SNP (transversion)	92.90%	1.40E-25					
Variants: VaAphrodite_B_FDSW210055 058-2r	ATGCGG	4054	4059	6	GCTGCT -> ATGCGG	11 -> 12	Substitution	90.9% -> 91.7%	1.10E-20					
Variants: VaAphrodite_B_FDSW210055 058-2r	GG	4061	4062	2	TT -> GG	11	Substitution	90.90%	1.10E-20					
Variants: VaAphrodite_B_FDSW210055 058-2r	TCGGTA	4065	4070	6	ATACCG -> TCGGTA	6 -> 10	Substitution	100.00%	1.00E-12					
Variants: VaAphrodite_B_FDSW210055 058-2r	T	4072	4072	1	C -> T	5	SNP (transition)	100.00%	1.00E-10					
Variants: VaAphrodite_B_FDSW210055 058-2r	AA	4343	4344	2	CG -> AA	16 -> 22	Substitution	93.8% -> 95.5%	5.00E-25	FD -> FY	hypothetical protein CDS	hypothetica l protein	Substituti on	KLI69 882.1
Variants: VaAphrodite_B_FDSW210055 058-2r	A	4365	4365	1	G -> A	178	SNP (transition)	99.40%	0		hypothetical protein CDS	hypothetica l protein	None	KLI69 882.1
Variants: VaAphrodite_B_FDSW210055 058-2r	TG	4371	4376	6	GCCGAT -> TG	215 -> 229	Deletion	93.4% -> 99.5%	1.00E-301		hypothetical protein CDS	hypothetica l protein	Frame Shift	KLI69 882.1
Variants: VaAphrodite_B_FDSW210055 058-2r	T	4378	4379	2	GC -> T	250 -> 261	Deletion	85.6% -> 86.2%	2.40E-257		hypothetical protein CDS	hypothetica l protein	Frame Shift	KLI69 882.1
Variants: VaAphrodite_B_FDSW210055 058-2r	CATGA	4381	4385	5	TGGTG -> CATGA	274 -> 296	Substitution	99.3% -> 99.7%	0		hypothetical protein CDS	hypothetica l protein	Truncatio n	KLI69 882.1
Variants: VaAphrodite_B_FDSW210055 058-2r	GG	4388	4389	2	CA -> GG	321 -> 328	Substitution	99.70%	0	PE -> PQ	hypothetical protein CDS	hypothetica l protein	Substituti on	KLI69 882.1
Variants: VaAphrodite_B_FDSW210055 058-2r	TGA	4392	4394	2	CGT -> TGA	345 -> 353	Substitution	100.00%	0	T -> S	hypothetical protein CDS	hypothetica l protein	Substituti on	KLI69 882.1
Variants: VaAphrodite_B_FDSW210055 058-2r	GT	4396	4397	2	AA -> GT	367 -> 375	Substitution	99.50%	0	F -> T	hypothetical protein CDS	hypothetica l protein	Substituti on	KLI69 882.1
Variants: VaAphrodite_B_FDSW210055 058-2r	A	4401	4401	1	C -> A	407	SNP (transversion)	99.80%	0	M -> V	hypothetical protein CDS	hypothetica l protein	Start Codon Loss	KLI69 882.1

Variants: VaAphrodite_B_FDSW210055 058-2r	A	4404	4406	3	GTT -> A	429	Deletion	99.80%	0
Variants: VaAphrodite_B_FDSW210055 058-2r	GT	4409	4410	2	TC -> GT	449 -> 460	Substitution	99.10%	0
Variants: VaAphrodite_B_FDSW210055 058-2r	GC	4414	4415	2	CT -> GC	485 -> 499	Substitution	99.40%	0
Variants: VaAphrodite_B_FDSW210055 058-2r	A	4417	4417	1	G -> A	517	SNP (transition)	99.80%	0
Variants: VaAphrodite_B_FDSW210055 058-2r	GG	4419	4420	2	CA -> GG	527 -> 535	Substitution	99.40%	0
Variants: VaAphrodite_B_FDSW210055 058-2r	AC	4423	4424	2	GA -> AC	555 -> 559	Substitution	99.60%	0
Variants: VaAphrodite_B_FDSW210055 058-2r	G	4427	4426	0	#NAME?	576	Insertion	99.80%	0
Variants: VaAphrodite_B_FDSW210055 058-2r	TG	4431	4432	2	GA -> TG	613 -> 618	Substitution	99.70%	0
Variants: VaAphrodite_B_FDSW210055 058-2r	A	4434	4433	0	#NAME?	635	Insertion	100.00%	0
Variants: VaAphrodite_B_FDSW210055 058-2r	CA	4435	4436	2	GT -> CA	647 -> 652	Substitution	100.00%	0
Variants: VaAphrodite_B_FDSW210055 058-2r	A	4668	4668	1	G -> A	756	SNP (transition)	25.50%	0
Variants: VaAphrodite_B_FDSW210055 058-2r	A	4973	4973	1	G -> A	166	SNP (transition)	66.90%	0
Variants: VaAphrodite_B_FDSW210055 058-2r	C	4975	4974	0	+C	158	Insertion	85.40%	0
Variants: VaAphrodite_B_FDSW210055 058-2r	G	4975	4974	0	#NAME?	154	Insertion	52.60%	4.60E-231
Variants: VaAphrodite_B_FDSW210055 058-2r	T	4975	4974	0	(T)3 -> (T)4	154	Insertion (tandem repeat)	34.40%	4.70E-139
Variants: VaAphrodite_B_FDSW210055 058-2r	AA	4975	4976	2	TT -> AA	144 -> 145	Substitution	48.6% -> 49.7%	1.40E-196
Variants: VaAphrodite_B_FDSW210055 058-2r	G	4978	4978	1	A -> G	139	SNP (transition)	48.20%	1.30E-194
Variants: VaAphrodite_B_FDSW210055 058-2r	A	4979	4979	1	G -> A	130	SNP (transition)	60.80%	1.10E-232
Variants: VaAphrodite_B_FDSW210055 058-2r	T	4980	4980	1	A -> T	128	SNP (transversion)	49.20%	1.50E-177
Variants: VaAphrodite_B_FDSW210055 058-2r	A	4981	4981	1	G -> A	124	SNP (transition)	62.10%	3.10E-220
Variants: VaAphrodite_B_FDSW210055 058-2r	C	4981	4981	1	G -> C	124	SNP (transversion)	29.00%	8.40E-92
Variants: VaAphrodite_B_FDSW210055 058-2r	A	4982	4982	1	T -> A	124	SNP (transversion)	76.60%	1.60E-295

Variants: VaAphrodite_B_FDSW210055 058-2r	A	4983	4983	1	G -> A	124	SNP (transition)	88.70%	0
Variants: VaAphrodite_B_FDSW210055 058-2r	C	4984	4984	1	A -> C	122	SNP (transversion)	47.50%	5.20E-174
Variants: VaAphrodite_B_FDSW210055 058-2r	A	4985	4985	1	T -> A	118	SNP (transversion)	59.30%	3.10E-198
Variants: VaAphrodite_B_FDSW210055 058-2r	C	4985	4985	1	T -> C	118	SNP (transition)	29.70%	1.10E-89
Variants: VaAphrodite_B_FDSW210055 058-2r	A	4986	4986	1	T -> A	114	SNP (transversion)	30.70%	8.50E-94
Variants: VaAphrodite_B_FDSW210055 058-2r	C	4986	4986	1	T -> C	114	SNP (transition)	57.90%	5.80E-186
Variants: VaAphrodite_B_FDSW210055 058-2r	A	4988	4988	1	G -> A	104	SNP (transition)	49.00%	9.50E-134
Variants: VaAphrodite_B_FDSW210055 058-2r	C	4988	4988	1	G -> C	104	SNP (transversion)	37.50%	1.20E-100
Variants: VaAphrodite_B_FDSW210055 058-2r	A	4989	4989	1	C -> A	100	SNP (transversion)	75.00%	2.40E-217
Variants: VaAphrodite_B_FDSW210055 058-2r	AA	4992	4993	2	CG -> AA	85 -> 87	Substitution	83.5% -> 83.9%	1.90E-219
Variants: VaAphrodite_B_FDSW210055 058-2r	A	4994	4994	1	C -> A	84	SNP (transversion)	42.90%	4.40E-92
Variants: VaAphrodite_B_FDSW210055 058-2r	A	4995	4995	1	G -> A	85	SNP (transition)	48.20%	9.90E-120
Variants: VaAphrodite_B_FDSW210055 058-2r	C	4995	4995	1	G -> C	85	SNP (transversion)	30.60%	7.80E-65
Variants: VaAphrodite_B_FDSW210055 058-2r	A	4996	4996	1	T -> A	83	SNP (transversion)	30.10%	3.40E-67
Variants: VaAphrodite_B_FDSW210055 058-2r	C	4996	4996	1	T -> C	83	SNP (transition)	47.00%	2.90E-117
Variants: VaAphrodite_B_FDSW210055 058-2r	A	4997	4997	1	G -> A	82	SNP (transition)	46.30%	3.40E-110
Variants: VaAphrodite_B_FDSW210055 058-2r	C	4997	4997	1	G -> C	82	SNP (transversion)	30.50%	2.30E-62
Variants: VaAphrodite_B_FDSW210055 058-2r	A	4998	4998	1	G -> A	81	SNP (transition)	29.60%	5.70E-62
Variants: VaAphrodite_B_FDSW210055 058-2r	T	4998	4998	1	G -> T	81	SNP (transversion)	35.80%	1.60E-74
Variants: VaAphrodite_B_FDSW210055 058-2r	T	4999	4999	1	C -> T	80	SNP (transition)	62.50%	8.70E-144
Variants: VaAphrodite_B_FDSW210055 058-2r	C	5000	5000	1	A -> C	77	SNP (transversion)	32.50%	3.60E-68
Variants: VaAphrodite_B_FDSW210055 058-2r	T	5000	5000	1	A -> T	77	SNP (transversion)	39.00%	2.10E-75

Variants: VaAphrodite_B_FDSW210055058-2r	A	5001	5001	1	T -> A	72	SNP (transversion)	30.60%	1.70E-48
Variants: VaAphrodite_B_FDSW210055058-2r	C	5001	5001	1	T -> C	72	SNP (transition)	27.80%	3.00E-47
Variants: VaAphrodite_B_FDSW210055058-2r	A	5002	5002	1	T -> A	70	SNP (transversion)	55.70%	6.90E-98
Variants: VaAphrodite_B_FDSW210055058-2r	A	5003	5003	1	T -> A	71	SNP (transversion)	38.00%	5.70E-65
Variants: VaAphrodite_B_FDSW210055058-2r	G	5003	5003	1	T -> G	71	SNP (transversion)	28.20%	2.20E-51
Variants: VaAphrodite_B_FDSW210055058-2r	GT	5005	5006	2	TA -> GT	57 -> 58	Substitution	33.3% -> 36.2%	9.90E-47
Variants: VaAphrodite_B_FDSW210055058-2r	T	5007	5007	1	C -> T	53	SNP (transition)	34.00%	6.30E-41
Variants: VaAphrodite_B_FDSW210055058-2r	T	5011	5011	1	G -> T	45	SNP (transversion)	31.10%	2.60E-34
Variants: VaAphrodite_B_FDSW210055058-2r	C	5579	5579	1	G -> C	48	SNP (transversion)	100.00%	1.00E-168
Variants: VaAphrodite_B_FDSW210055058-2r	T	5581	5581	1	A -> T	48	SNP (transversion)	100.00%	2.50E-154
Variants: VaAphrodite_B_FDSW210055058-2r	CG	5583	5584	2	TA -> CG	47	Substitution	100.00%	1.60E-160
Variants: VaAphrodite_B_FDSW210055058-2r	GTC	5605	5607	3	TAA -> GTC	39 -> 40	Substitution	25.0% -> 28.2%	8.20E-22
Variants: VaAphrodite_B_FDSW210055058-2r	TT	5609	5610	2	AA -> TT	42 -> 45	Substitution	35.7% -> 40.0%	3.00E-33
Variants: VaAphrodite_B_FDSW210055058-2r	AA	5612	5613	2	GC -> AA	51 -> 55	Substitution	47.1% -> 52.7%	2.20E-58
Variants: VaAphrodite_B_FDSW210055058-2r	GGG	5615	5617	3	TTA -> GGG	51 -> 52	Substitution	60.8% -> 63.5%	4.80E-86
Variants: VaAphrodite_B_FDSW210055058-2r	A	5618	5618	1	G -> A	52	SNP (transition)	65.40%	6.70E-96
Variants: VaAphrodite_B_FDSW210055058-2r	T	5618	5618	1	G -> T	52	SNP (transversion)	34.60%	1.10E-44
Variants: VaAphrodite_B_FDSW210055058-2r	GT	5619	5620	2	TC -> GT	51 -> 52	Substitution	100.00%	4.00E-167
Variants: VaAphrodite_B_FDSW210055058-2r	C	5621	5621	1	G -> C	53	SNP (transversion)	67.90%	5.10E-106
Variants: VaAphrodite_B_FDSW210055058-2r	T	5622	5622	1	C -> T	54	SNP (transition)	100.00%	4.00E-168
Variants: VaAphrodite_B_FDSW210055058-2r	G	5623	5623	1	C -> G	55	SNP (transversion)	67.30%	1.10E-108
Variants: VaAphrodite_B_FDSW210055058-2r	AA	5624	5625	2	TT -> AA	57 -> 58	Substitution	96.5% -> 96.6%	1.60E-165

Variants: VaAphrodite_B_FDSW210055 058-2r	A	5626	5626	1	G -> A	57	SNP (transition)	75.40%	7.60E-117
Variants: VaAphrodite_B_FDSW210055 058-2r	T	5632	5632	1	A -> T	61	SNP (transversion)	93.40%	6.50E-149
Variants: VaAphrodite_B_FDSW210055 058-2r	TATG	5633	5636	4	ATCA -> TATG	61 -> 62	Substitution	79.0% -> 80.6%	5.10E-125
Variants: VaAphrodite_B_FDSW210055 058-2r	AA	5637	5638	2	TT -> AA	63 -> 64	Substitution	84.1% -> 84.4%	9.40E-141
Variants: VaAphrodite_B_FDSW210055 058-2r	AG	5645	5646	2	TC -> AG	75 -> 77	Substitution	89.3% -> 89.6%	8.40E-185
Variants: VaAphrodite_B_FDSW210055058-2r		5648	5648	1	(C)3 -> (C)2	83	Deletion (tandem repeat)	78.30%	9.50E-68
Variants: VaAphrodite_B_FDSW210055 058-2r	T	5653	5653	1	G -> T	118	SNP (transversion)	71.20%	2.90E-206
Variants: VaAphrodite_B_FDSW210055 058-2r	GC	5658	5657	0	#NAME?	144	Insertion	56.30%	6.90E-186
Variants: VaAphrodite_C_FDSW210055 059-1r	TGGT	79	82	4	CCTG -> TGGT	151 -> 181	Substitution	89.4% -> 91.2%	0
Variants: VaAphrodite_C_FDSW210055 059-1r	G	84	84	1	A -> G	136	SNP (transition)	95.60%	0
Variants: VaAphrodite_C_FDSW210055 059-1r	GGGG	86	89	4	CATC -> GGGG	112 -> 122	Substitution	90.2% -> 91.8%	2.50E-160
Variants: VaAphrodite_C_FDSW210055 059-1r	C	91	91	1	T -> C	107	SNP (transition)	94.40%	1.80E-294
Variants: VaAphrodite_C_FDSW210055 059-1r	GGA	93	95	3	CAC -> GGA	91 -> 93	Substitution	92.3% -> 92.5%	8.00E-243
Variants: VaAphrodite_C_FDSW210055 059-1r	T	97	97	1	A -> T	80	SNP (transversion)	85.00%	1.50E-204
Variants: VaAphrodite_C_FDSW210055 059-1r	GAACC	99	103	5	CGTTG -> GAACC	63 -> 74	Substitution	75.4% -> 86.5%	1.30E-82
Variants: VaAphrodite_C_FDSW210055 059-1r	G	104	104	1	C -> G	54	SNP (transversion)	92.60%	3.10E-135
Variants: VaAphrodite_C_FDSW210055 059-1r	A	105	105	1	C -> A	48	SNP (transversion)	62.50%	7.00E-66
Variants: VaAphrodite_C_FDSW210055 059-1r	CG	106	107	2	GT -> CG	47 -> 50	Substitution	70.2% -> 76.0%	6.70E-85
Variants: VaAphrodite_C_FDSW210055 059-1r	G	120	120	1	A -> G	94	SNP (transition)	29.80%	4.60E-25
Variants: VaAphrodite_C_FDSW210055 059-1r	T	126	126	1	C -> T	125	SNP (transition)	43.20%	3.70E-57
Variants: VaAphrodite_C_FDSW210055 059-1r	T	129	129	1	C -> T	144	SNP (transition)	75.70%	2.60E-185
Variants: VaAphrodite_C_FDSW210055 059-1r	G	132	132	1	C -> G	159	SNP (transversion)	33.30%	4.50E-80

Variants: VaAphrodite_C_FDSW210055 059-1r	T	144	144	1	C -> T	227	SNP (transition)	45.40%	1.30E-140
Variants: VaAphrodite_C_FDSW210055 059-1r	G	150	150	1	C -> G	268	SNP (transversion)	37.30%	1.40E-145
Variants: VaAphrodite_C_FDSW210055 059-1r	A	159	159	1	G -> A	316	SNP (transition)	78.50%	0
Variants: VaAphrodite_C_FDSW210055 059-1r	A	165	165	1	T -> A	347	SNP (transversion)	34.30%	1.30E-167
Variants: VaAphrodite_C_FDSW210055 059-1r	A	183	183	1	G -> A	401	SNP (transition)	27.90%	3.20E-168
Variants: VaAphrodite_C_FDSW210055 059-1r	A	192	192	1	G -> A	436	SNP (transition)	35.80%	0
Variants: VaAphrodite_C_FDSW210055 059-1r	G	216	216	1	A -> G	547	SNP (transition)	25.60%	3.40E-287
Variants: VaAphrodite_C_FDSW210055 059-1r	T	225	225	1	A -> T	582	SNP (transversion)	47.40%	0
Variants: VaAphrodite_C_FDSW210055 059-1r	C	237	237	1	T -> C	641	SNP (transition)	33.20%	3.00E-232
Variants: VaAphrodite_C_FDSW210055 059-1r	C	240	240	1	T -> C	649	SNP (transition)	41.30%	0
Variants: VaAphrodite_C_FDSW210055 059-1r	A	294	294	1	G -> A	700	SNP (transition)	39.40%	0
Variants: VaAphrodite_C_FDSW210055 059-1r	A	357	357	1	G -> A	985	SNP (transition)	56.30%	0
Variants: VaAphrodite_C_FDSW210055 059-1r	G	363	363	1	A -> G	1009	SNP (transition)	58.90%	0
Variants: VaAphrodite_C_FDSW210055 059-1r	C	369	369	1	T -> C	1049	SNP (transition)	78.50%	0
Variants: VaAphrodite_C_FDSW210055 059-1r	A	371	371	1	G -> A	1057	SNP (transition)	53.20%	0
Variants: VaAphrodite_C_FDSW210055 059-1r	G	387	387	1	C -> G	1115	SNP (transversion)	56.80%	0
Variants: VaAphrodite_C_FDSW210055 059-1r	A	393	393	1	G -> A	1132	SNP (transition)	25.70%	0
Variants: VaAphrodite_C_FDSW210055 059-1r	C	393	393	1	G -> C	1132	SNP (transversion)	44.80%	0
Variants: VaAphrodite_C_FDSW210055 059-1r	C	396	396	1	A -> C	1144	SNP (transversion)	36.50%	0
Variants: VaAphrodite_C_FDSW210055 059-1r	G	420	420	1	A -> G	1247	SNP (transition)	33.00%	0
Variants: VaAphrodite_C_FDSW210055 059-1r	T	420	420	1	A -> T	1247	SNP (transversion)	29.60%	0
Variants: VaAphrodite_C_FDSW210055 059-1r	A	422	422	1	T -> A	1258	SNP (transversion)	30.20%	0

Variants: VaAphrodite_C_FDSW210055 059-1r	A	423	423	1	G -> A	1255	SNP (transition)	61.90%	0
Variants: VaAphrodite_C_FDSW210055 059-1r	A	426	426	1	G -> A	1271	SNP (transition)	61.70%	0
Variants: VaAphrodite_C_FDSW210055 059-1r	G	438	438	1	A -> G	1278	SNP (transition)	47.60%	0
Variants: VaAphrodite_C_FDSW210055 059-1r	A	456	456	1	G -> A	1246	SNP (transition)	46.10%	0
Variants: VaAphrodite_C_FDSW210055 059-1r	C	462	462	1	T -> C	1231	SNP (transition)	25.80%	0
Variants: VaAphrodite_C_FDSW210055 059-1r	G	469	469	1	C -> G	1242	SNP (transversion)	25.70%	0
Variants: VaAphrodite_C_FDSW210055 059-1r	C	489	489	1	T -> C	1215	SNP (transition)	44.80%	0
Variants: VaAphrodite_C_FDSW210055 059-1r	C	498	498	1	G -> C	1165	SNP (transversion)	31.80%	0
Variants: VaAphrodite_C_FDSW210055 059-1r	C	513	513	1	T -> C	1095	SNP (transition)	51.70%	0
Variants: VaAphrodite_C_FDSW210055 059-1r	C	516	516	1	T -> C	1075	SNP (transition)	33.20%	0
Variants: VaAphrodite_C_FDSW210055 059-1r	T	531	531	1	C -> T	1048	SNP (transition)	30.80%	0
Variants: VaAphrodite_C_FDSW210055 059-1r	C	537	537	1	G -> C	1035	SNP (transversion)	57.80%	0
Variants: VaAphrodite_C_FDSW210055 059-1r	G	540	540	1	A -> G	1029	SNP (transition)	34.20%	0
Variants: VaAphrodite_C_FDSW210055 059-1r	A	552	552	1	T -> A	1015	SNP (transversion)	38.40%	0
Variants: VaAphrodite_C_FDSW210055 059-1r	A	564	564	1	C -> A	972	SNP (transversion)	31.30%	0
Variants: VaAphrodite_C_FDSW210055 059-1r	G	588	588	1	A -> G	886	SNP (transition)	36.20%	0
Variants: VaAphrodite_C_FDSW210055 059-1r	C	663	663	1	T -> C	561	SNP (transition)	78.60%	0
Variants: VaAphrodite_C_FDSW210055 059-1r	G	677	677	1	A -> G	469	SNP (transition)	80.40%	0
Variants: VaAphrodite_C_FDSW210055 059-1r	C	684	684	1	T -> C	429	SNP (transition)	77.40%	0
Variants: VaAphrodite_C_FDSW210055 059-1r	A	687	687	1	G -> A	408	SNP (transition)	78.70%	0
Variants: VaAphrodite_C_FDSW210055 059-1r	T	690	690	1	A -> T	390	SNP (transversion)	82.60%	0
Variants: VaAphrodite_C_FDSW210055 059-1r	A	693	693	1	G -> A	370	SNP (transition)	81.60%	0

Variants: VaAphrodite_C_FDSW210055 059-1r	A	708	708	1	G -> A	284	SNP (transition)	82.40%	0					
Variants: VaAphrodite_C_FDSW210055 059-1r	T	723	723	1	C -> T	225	SNP (transition)	84.00%	0					
Variants: VaAphrodite_C_FDSW210055 059-1r	C	726	726	1	T -> C	214	SNP (transition)	84.10%	0					
Variants: VaAphrodite_C_FDSW210055 059-1r	AC	745	746	2	GA -> AC	156 -> 160	Substitution	25.6% -> 28.8%	5.50E-65					
Variants: VaAphrodite_C_FDSW210055 059-1r	C	747	747	1	T -> C	159	SNP (transition)	67.90%	2.20E-293					
Variants: VaAphrodite_C_FDSW210055 059-1r	CGTTCGA	749	755	7	TACCTTG -> CGTTCGA	163 -> 174	Substitution	40.2% -> 58.9%	1.40E-169					
Variants: VaAphrodite_C_FDSW210055 059-1r	C	756	756	1	G -> C	178	SNP (transversion)	37.60%	8.70E-152					
Variants: VaAphrodite_C_FDSW210055 059-1r	T	756	756	1	G -> T	178	SNP (transversion)	41.60%	4.00E-164					
Variants: VaAphrodite_C_FDSW210055 059-1r	AGCG	757	760	4	TCAC -> AGCG	171 -> 176	Substitution	25.7% -> 37.5%	3.20E-76					
Variants: VaAphrodite_C_FDSW210055 059-1r	T	765	765	1	C -> T	172	SNP (transition)	33.10%	1.50E-74					
Variants: VaAphrodite_C_FDSW210055 059-1r	A	768	768	1	T -> A	176	SNP (transversion)	56.30%	1.40E-246					
Variants: VaAphrodite_C_FDSW210055 059-1r	T	790	790	1	G -> T	296	SNP (transversion)	27.00%	2.70E-127	R -> L	hypothetical protein CDS	hypothetica l protein	Substituti on	KLI69 879.1
Variants: VaAphrodite_C_FDSW210055 059-1r	A	795	795	1	G -> A	337	SNP (transition)	59.60%	0	A -> T	hypothetical protein CDS	hypothetica l protein	Substituti on	KLI69 879.1
Variants: VaAphrodite_C_FDSW210055 059-1r	AAT	801	803	2	CAC -> AAT	379 -> 395	Substitution	73.4% -> 76.5%	0	H -> N	hypothetical protein CDS	hypothetica l protein	Substituti on	KLI69 879.1
Variants: VaAphrodite_C_FDSW210055 059-1r	C	834	834	1	T -> C	543	SNP (transition)	79.00%	0	Y -> H	hypothetical protein CDS	hypothetica l protein	Substituti on	KLI69 879.1
Variants: VaAphrodite_C_FDSW210055 059-1r	G	888	888	1	A -> G	814	SNP (transition)	32.60%	0	I -> V	hypothetical protein CDS	hypothetica l protein	Substituti on	KLI69 879.1
Variants: VaAphrodite_C_FDSW210055 059-1r	C	897	897	1	T -> C	823	SNP (transition)	61.50%	0	C -> R	hypothetical protein CDS	hypothetica l protein	Substituti on	KLI69 879.1
Variants: VaAphrodite_C_FDSW210055 059-1r	C	900	900	1	G -> C	818	SNP (transversion)	61.60%	0	D -> H	hypothetical protein CDS	hypothetica l protein	Substituti on	KLI69 879.1
Variants: VaAphrodite_C_FDSW210055 059-1r	C	942	942	1	G -> C	790	SNP (transversion)	32.40%	0	G -> R	hypothetical protein CDS	hypothetica l protein	Substituti on	KLI69 879.1
Variants: VaAphrodite_C_FDSW210055 059-1r	TGT	966	968	2	AGC -> TGT	711 -> 714	Substitution	45.3% -> 46.5%	0	S -> C	hypothetical protein CDS	hypothetica l protein	Substituti on	KLI69 879.1
Variants: VaAphrodite_C_FDSW210055 059-1r	G	969	969	1	C -> G	716	SNP (transversion)	29.10%	2.50E-294	P -> A	hypothetical protein CDS	hypothetica l protein	Substituti on	KLI69 879.1
Variants: VaAphrodite_C_FDSW210055 059-1r	A	976	976	1	T -> A	711	SNP (transversion)	29.50%	1.50E-257	V -> E	hypothetical protein CDS	hypothetica l protein	Substituti on	KLI69 879.1

Variants: VaAphrodite_C_FDSW210055 059-1r	A	1008	1008	1	G -> A	669	SNP (transition)	32.70%	4.60E-237	V -> I	hypothetical protein CDS	hypothetica l protein	Substituti on	KLI69 879.1
Variants: VaAphrodite_C_FDSW210055 059-1r	C	1035	1035	1	G -> C	689	SNP (transversion)	27.60%	7.70E-283	G -> R	hypothetical protein CDS	hypothetica l protein	Substituti on	KLI69 879.1
Variants: VaAphrodite_C_FDSW210055 059-1r	A	1041	1041	1	G -> A	685	SNP (transition)	32.70%	0	A -> T	hypothetical protein CDS	hypothetica l protein	Substituti on	KLI69 879.1
Variants: VaAphrodite_C_FDSW210055 059-1r	C	1049	1049	1	T -> C	700	SNP (transition)	25.40%	0		hypothetical protein CDS	hypothetica l protein	None	KLI69 879.1
Variants: VaAphrodite_C_FDSW210055 059-1r	G	1083	1083	1	A -> G	726	SNP (transition)	32.90%	0	K -> E	hypothetical protein CDS	hypothetica l protein	Substituti on	KLI69 879.1
Variants: VaAphrodite_C_FDSW210055 059-1r	A	1100	1100	1	G -> A	738	SNP (transition)	33.60%	0		hypothetical protein CDS	hypothetica l protein	None	KLI69 879.1
Variants: VaAphrodite_C_FDSW210055 059-1r	A	1101	1101	1	G -> A	741	SNP (transition)	30.80%	0					
Variants: VaAphrodite_C_FDSW210055 059-1r	C	1104	1104	1	G -> C	757	SNP (transversion)	33.90%	0					
Variants: VaAphrodite_C_FDSW210055 059-1r	T	1116	1116	1	C -> T	790	SNP (transition)	35.40%	0		hypothetical protein CDS	hypothetica l protein	None	KLI69 880.1
Variants: VaAphrodite_C_FDSW210055 059-1r	T	1119	1119	1	C -> T	787	SNP (transition)	33.30%	0		hypothetical protein CDS	hypothetica l protein	None	KLI69 880.1
Variants: VaAphrodite_C_FDSW210055 059-1r	G	1135	1135	1	T -> G	805	SNP (transversion)	61.00%	0	F -> V	hypothetical protein CDS	hypothetica l protein	Substituti on	KLI69 880.1
Variants: VaAphrodite_C_FDSW210055 059-1r	TGTG	1173	1176	3	CATA -> TGTG	722 -> 742	Substitution	34.6% -> 35.9%	0	TI -> TV	hypothetical protein CDS	hypothetica l protein	Substituti on	KLI69 880.1
Variants: VaAphrodite_C_FDSW210055 059-1r	C	1182	1182	1	T -> C	708	SNP (transition)	35.20%	0		hypothetical protein CDS	hypothetica l protein	None	KLI69 880.1
Variants: VaAphrodite_C_FDSW210055 059-1r	G	1194	1194	1	A -> G	699	SNP (transition)	31.30%	0		hypothetical protein CDS	hypothetica l protein	None	KLI69 880.1
Variants: VaAphrodite_C_FDSW210055 059-1r	G	1215	1215	1	A -> G	560	SNP (transition)	43.80%	0		hypothetical protein CDS	hypothetica l protein	None	KLI69 880.1
Variants: VaAphrodite_C_FDSW210055 059-1r	G	1218	1218	1	A -> G	548	SNP (transition)	43.10%	0		hypothetical protein CDS	hypothetica l protein	None	KLI69 880.1
Variants: VaAphrodite_C_FDSW210055 059-1r	G	1221	1221	1	C -> G	536	SNP (transversion)	43.10%	0		hypothetical protein CDS	hypothetica l protein	None	KLI69 880.1
Variants: VaAphrodite_C_FDSW210055 059-1r	GG	1232	1233	2	AC -> GG	484 -> 490	Substitution	42.1% -> 42.2%	0	N -> R	hypothetical protein CDS	hypothetica l protein	Substituti on	KLI69 880.1
Variants: VaAphrodite_C_FDSW210055 059-1r	T	1235	1235	1	C -> T	477	SNP (transition)	41.90%	0	A -> V	hypothetical protein CDS	hypothetica l protein	Substituti on	KLI69 880.1
Variants: VaAphrodite_C_FDSW210055 059-1r	A	1262	1262	1	G -> A	305	SNP (transition)	30.20%	9.10E-234	G -> E	hypothetical protein CDS	hypothetica l protein	Substituti on	KLI69 880.1
Variants: VaAphrodite_C_FDSW210055 059-1r	G	1269	1269	1	A -> G	272	SNP (transition)	61.00%	0	I -> M	hypothetical protein CDS	hypothetica l protein	Substituti on	KLI69 880.1
Variants: VaAphrodite_C_FDSW210055 059-1r	G	1275	1275	1	A -> G	241	SNP (transition)	64.30%	0		hypothetical protein CDS	hypothetica l protein	None	KLI69 880.1

Variants: VaAphrodite_C_FDSW210055059-1r	T	1287	1287	1	A -> T	187	SNP (transversion)	64.70%	0		hypothetical protein CDS	hypothetical protein	None	KLI69880.1
Variants: VaAphrodite_C_FDSW210055059-1r	T	1290	1290	1	C -> T	178	SNP (transition)	63.50%	1.60E-301		hypothetical protein CDS	hypothetical protein	None	KLI69880.1
Variants: VaAphrodite_C_FDSW210055059-1r	G	1293	1293	1	C -> G	162	SNP (transversion)	65.40%	9.20E-296	D -> E	hypothetical protein CDS	hypothetical protein	Substitution	KLI69880.1
Variants: VaAphrodite_C_FDSW210055059-1r	ATTG	1296	1299	4	CAAC -> ATTG	144 -> 151	Substitution	64.9% -> 66.7%	1.10E-261	TN -> TL	hypothetical protein CDS	hypothetical protein	Substitution	KLI69880.1
Variants: VaAphrodite_C_FDSW210055059-1r	A	1305	1305	1	G -> A	135	SNP (transition)	66.70%	1.50E-252		hypothetical protein CDS	hypothetical protein	None	KLI69880.1
Variants: VaAphrodite_C_FDSW210055059-1r	A	1311	1311	1	G -> A	119	SNP (transition)	51.30%	2.80E-161		hypothetical protein CDS	hypothetical protein	None	KLI69880.1
Variants: VaAphrodite_C_FDSW210055059-1r	T	1314	1314	1	G -> T	115	SNP (transversion)	52.20%	2.70E-159		hypothetical protein CDS	hypothetical protein	None	KLI69880.1
Variants: VaAphrodite_C_FDSW210055059-1r	G	1317	1317	1	C -> G	112	SNP (transversion)	50.90%	1.50E-150	D -> E	hypothetical protein CDS	hypothetical protein	Substitution	KLI69880.1
Variants: VaAphrodite_C_FDSW210055059-1r	GCTA	1320	1323	4	TGAG -> GCTA	91 -> 99	Substitution	61.2% -> 62.6%	2.70E-135	TE -> TL	hypothetical protein CDS	hypothetical protein	Substitution	KLI69880.1
Variants: VaAphrodite_C_FDSW210055059-1r	A	1324	1324	1	C -> A	91	SNP (transversion)	62.60%	3.40E-167	H -> N	hypothetical protein CDS	hypothetical protein	Substitution	KLI69880.1
Variants: VaAphrodite_C_FDSW210055059-1r	AAG	1324	1326	2	CAC -> AAG	91 -> 92	Substitution	31.5% -> 31.9%	8.50E-67	H -> K	hypothetical protein CDS	hypothetical protein	Substitution	KLI69880.1
Variants: VaAphrodite_C_FDSW210055059-1r	TT	1327	1328	2	AC -> TT	81 -> 86	Substitution	29.6% -> 30.2%	4.70E-49	T -> F	hypothetical protein CDS	hypothetical protein	Substitution	KLI69880.1
Variants: VaAphrodite_C_FDSW210055059-1r	G	1327	1329	3	ACC -> G	77 -> 86	Deletion	50.0% -> 55.8%	2.60E-38		hypothetical protein CDS	hypothetical protein	Frame Shift	KLI69880.1
Variants: VaAphrodite_C_FDSW210055059-1r	A	1330	1330	1	T -> A	76	SNP (transversion)	26.30%	1.00E-40	L -> I	hypothetical protein CDS	hypothetical protein	Substitution	KLI69880.1
Variants: VaAphrodite_C_FDSW210055059-1r	TCAC	1335	1338	3	AGAG -> TCAC	61 -> 74	Substitution	45.9% -> 47.3%	1.20E-64	TE -> TH	hypothetical protein CDS	hypothetical protein	Substitution	KLI69880.1
Variants: VaAphrodite_C_FDSW210055059-1r	A	1340	1340	1	G -> A	57	SNP (transition)	43.90%	9.60E-60	R -> H	hypothetical protein CDS	hypothetical protein	Substitution	KLI69880.1
Variants: VaAphrodite_C_FDSW210055059-1r	CG	1342	1343	2	TA -> CG	51 -> 54	Substitution	24.1% -> 25.5%	1.00E-33		hypothetical protein CDS	hypothetical protein	Extension	KLI69880.1
Variants: VaAphrodite_C_FDSW210055059-1r	C	1345	1345	1	G -> C	42	SNP (transversion)	28.60%	2.70E-24					
Variants: VaAphrodite_C_FDSW210055059-1r	A	2111	2111	1	T -> A	44	SNP (transversion)	27.30%	1.30E-33	I -> N	hypothetical protein CDS	hypothetical protein	Substitution	KLI69881.1
Variants: VaAphrodite_C_FDSW210055059-1r	C	2114	2114	1	A -> C	43	SNP (transversion)	32.60%	3.10E-40	D -> A	hypothetical protein CDS	hypothetical protein	Substitution	KLI69881.1
Variants: VaAphrodite_C_FDSW210055059-1r	GAA	2116	2118	3	TTT -> GAA	48 -> 49	Substitution	26.5% -> 27.1%	5.80E-38	F -> E	hypothetical protein CDS	hypothetical protein	Substitution	KLI69881.1
Variants: VaAphrodite_C_FDSW210055059-1r	ACG	2119	2121	3	GAA -> ACG	46 -> 50	Substitution	34.0% -> 37.0%	8.10E-51	E -> T	hypothetical protein CDS	hypothetical protein	Substitution	KLI69881.1

Variants: VaAphrodite_C_FDSW210055 059-1r	GATGA	2122	2126	4	AAAAT -> GATGA	50 -> 51	Substitution	44.0% -> 54.9%	1.40E-61	KI -> DD	hypothetical protein CDS	hypothetica l protein	Substituti on	KLI69 881.1
Variants: VaAphrodite_C_FDSW210055 059-1r	AA	2128	2129	2	GC -> AA	51 -> 53	Substitution	60.4% -> 62.7%	1.20E-92	A -> N	hypothetical protein CDS	hypothetica l protein	Substituti on	KLI69 881.1
Variants: VaAphrodite_C_FDSW210055 059-1r	CAC	2131	2133	2	TAA -> CAC	49 -> 51	Substitution	66.7% -> 69.4%	1.60E-107		hypothetical protein CDS	hypothetica l protein	Extension	KLI69 881.1
Variants: VaAphrodite_C_FDSW210055 059-1r	T	2135	2135	1	A -> T	50	SNP (transversion)	80.00%	1.00E-122					
Variants: VaAphrodite_C_FDSW210055 059-1r	CTG	2137	2139	3	AGC -> CTG	54 -> 55	Substitution	76.4% -> 77.8%	5.40E-132					
Variants: VaAphrodite_C_FDSW210055 059-1r	A	2141	2141	1	T -> A	60	SNP (transversion)	68.30%	1.30E-116					
Variants: VaAphrodite_C_FDSW210055 059-1r	G	2141	2141	1	T -> G	60	SNP (transversion)	28.30%	1.20E-45					
Variants: VaAphrodite_C_FDSW210055 059-1r	G	2142	2142	1	T -> G	61	SNP (transversion)	68.90%	4.70E-128					
Variants: VaAphrodite_C_FDSW210055 059-1r	AG	2143	2144	2	TA -> AG	62	Substitution	30.60%	1.70E-53					
Variants: VaAphrodite_C_FDSW210055 059-1r	G	2145	2145	1	A -> G	62	SNP (transition)	98.40%	2.00E-212					
Variants: VaAphrodite_C_FDSW210055 059-1r	T	2146	2146	1	G -> T	62	SNP (transversion)	30.60%	1.30E-51					
Variants: VaAphrodite_C_FDSW210055 059-1r	G	2147	2147	1	A -> G	64	SNP (transition)	32.80%	1.60E-55					
Variants: VaAphrodite_C_FDSW210055 059-1r	T	2147	2147	1	A -> T	64	SNP (transversion)	65.60%	1.30E-126					
Variants: VaAphrodite_C_FDSW210055 059-1r	G	2149	2148	0	#NAME?	67	Insertion	61.20%	1.10E-121					
Variants: VaAphrodite_C_FDSW210055 059-1r	GAG	2902	2904	3	TGA -> GAG	22 -> 26	Substitution	86.4% -> 88.5%	7.70E-49					
Variants: VaAphrodite_C_FDSW210055 059-1r	GGA	2906	2908	3	TCG -> GGA	20	Substitution	70.0% -> 85.0%	1.50E-32					
Variants: VaAphrodite_C_FDSW210055 059-1r	GGGA	2910	2913	4	AACG -> GGGA	20 -> 22	Substitution	55.0% -> 59.1%	1.20E-18					
Variants: VaAphrodite_C_FDSW210055059-1r		3097	3097	1	#NAME?	326	Deletion	46.60%	2.20E-149					
Variants: VaAphrodite_C_FDSW210055 059-1r	AG	3451	3452	2	GT -> AG	15 -> 16	Substitution	31.3% -> 33.3%	4.00E-07					
Variants: VaAphrodite_C_FDSW210055 059-1r	T	3659	3659	1	G -> T	332	SNP (transversion)	53.00%	0					
Variants: VaAphrodite_C_FDSW210055 059-1r	C	3758	3758	1	A -> C	352	SNP (transversion)	51.70%	0					
Variants: VaAphrodite_C_FDSW210055 059-1r	G	3778	3778	1	A -> G	367	SNP (transition)	47.10%	0					

Variants: VaAphrodite_C_FDSW210055059-1r	T	3780	3780	1	C -> T	368	SNP (transition)	46.70%	0					
Variants: VaAphrodite_C_FDSW210055059-1r	C	3852	3852	1	T -> C	392	SNP (transition)	50.80%	0					
Variants: VaAphrodite_C_FDSW210055059-1r	G	3864	3864	1	A -> G	381	SNP (transition)	50.90%	0					
Variants: VaAphrodite_C_FDSW210055059-1r	T	3922	3922	1	C -> T	283	SNP (transition)	43.80%	0					
Variants: VaAphrodite_C_FDSW210055059-1r	T	4022	4022	1	G -> T	49	SNP (transversion)	100.00%	6.30E-138					
Variants: VaAphrodite_C_FDSW210055059-1r	A	4023	4023	1	T -> A	47	SNP (transversion)	78.70%	2.00E-109					
Variants: VaAphrodite_C_FDSW210055059-1r	T	4025	4025	1	C -> T	42	SNP (transition)	100.00%	2.50E-118					
Variants: VaAphrodite_C_FDSW210055059-1r	T	4027	4027	1	A -> T	38	SNP (transversion)	100.00%	4.00E-107					
Variants: VaAphrodite_C_FDSW210055059-1r	TTG	4031	4033	3	CCA -> TTG	27 -> 30	Substitution	100.00%	6.30E-71					
Variants: VaAphrodite_C_FDSW210055059-1r	T	4035	4035	1	C -> T	24	SNP (transition)	75.00%	2.10E-42					
Variants: VaAphrodite_C_FDSW210055059-1r	T	4036	4036	1	G -> T	22	SNP (transversion)	100.00%	2.50E-51					
Variants: VaAphrodite_C_FDSW210055059-1r	T	4037	4037	1	G -> T	22	SNP (transversion)	77.30%	1.60E-40					
Variants: VaAphrodite_C_FDSW210055059-1r		4038	4038	1	-C	20	Deletion	100.00%	1.00E-22					
Variants: VaAphrodite_C_FDSW210055059-1r	A	4040	4040	1	T -> A	19	SNP (transversion)	100.00%	2.00E-44					
Variants: VaAphrodite_C_FDSW210055059-1r	GTTTAA	4043	4048	6	ACCGTT -> GTTTAA	13 -> 16	Substitution	100.00%	2.00E-25					
Variants: VaAphrodite_C_FDSW210055059-1r	GTGGTA	4050	4055	6	CGCTGC -> GTGGTA	6 -> 11	Substitution	100.00%	1.60E-14					
Variants: VaAphrodite_C_FDSW210055059-1r	AG	4339	4340	2	TT -> AG	25 -> 26	Substitution	100.00%	1.00E-45	K -> L	hypothetical protein CDS	hypothetical protein	Substitution	KLI69882.1
Variants: VaAphrodite_C_FDSW210055059-1r	TGGTTCATGA	4378	4387	9	GCTTGGTGCT -> TGGTTCATGA	335 -> 431	Substitution	98.6% -> 99.1%	0	EHQA -> VMNH	hypothetical protein CDS	hypothetical protein	Substitution	KLI69882.1
Variants: VaAphrodite_C_FDSW210055059-1r	T	4389	4389	1	A -> T	441	SNP (transversion)	99.80%	0		hypothetical protein CDS	hypothetical protein	None	KLI69882.1
Variants: VaAphrodite_C_FDSW210055059-1r	G	4392	4392	1	C -> G	468	SNP (transversion)	99.60%	0		hypothetical protein CDS	hypothetical protein	None	KLI69882.1
Variants: VaAphrodite_C_FDSW210055059-1r	G	4395	4395	1	A -> G	485	SNP (transition)	100.00%	0		hypothetical protein CDS	hypothetical protein	None	KLI69882.1
Variants: VaAphrodite_C_FDSW210055059-1r	GTCGTA	4398	4403	6	CGTCAC -> GTCGTA	512 -> 560	Substitution	98.7% -> 98.8%	0	MT -> YD	hypothetical protein CDS	hypothetical protein	Start Codon Loss	KLI69882.1

Variants: VaAphrodite_C_FDSW210055 059-1r	ACA	4404	4406	3	GTT -> ACA	565 -> 583	Substitution	99.10%		0
Variants: VaAphrodite_C_FDSW210055 059-1r	GT	4409	4410	2	TC -> GT	609 -> 618	Substitution	99.3% -> 99.4%		0
Variants: VaAphrodite_C_FDSW210055 059-1r	GC	4414	4415	2	CT -> GC	639 -> 655	Substitution	99.80%		0
Variants: VaAphrodite_C_FDSW210055 059-1r	A	4417	4417	1	G -> A	670	SNP (transition)	99.70%		0
Variants: VaAphrodite_C_FDSW210055 059-1r	GG	4419	4420	2	CA -> GG	688 -> 695	Substitution	100.00%		0
Variants: VaAphrodite_C_FDSW210055 059-1r	AC	4423	4424	2	GA -> AC	722 -> 725	Substitution	98.80%		0
Variants: VaAphrodite_C_FDSW210055 059-1r	G	4427	4426	0	#NAME?	744	Insertion	100.00%		0
Variants: VaAphrodite_C_FDSW210055 059-1r	TG	4431	4432	2	GA -> TG	788 -> 802	Substitution	99.50%		0
Variants: VaAphrodite_C_FDSW210055 059-1r	A	4434	4433	0	#NAME?	828	Insertion	99.90%		0
Variants: VaAphrodite_C_FDSW210055 059-1r	CA	4435	4436	2	GT -> CA	856 -> 873	Substitution	99.5% -> 99.6%		0
Variants: VaAphrodite_C_FDSW210055 059-1r	A	4973	4973	1	G -> A	200	SNP (transition)	71.50%		0
Variants: VaAphrodite_C_FDSW210055 059-1r	C	4975	4975	1	T -> C	192	SNP (transition)	80.20%		0
Variants: VaAphrodite_C_FDSW210055 059-1r	G	4976	4976	1	T -> G	188	SNP (transversion)	70.20%		0
Variants: VaAphrodite_C_FDSW210055 059-1r	AGA	4979	4981	3	GAG -> AGA	162 -> 173	Substitution	65.4% -> 67.6%	1.2E-320	
Variants: VaAphrodite_C_FDSW210055 059-1r	A	4982	4982	1	T -> A	156	SNP (transversion)	28.80%		1.10E-109
Variants: VaAphrodite_C_FDSW210055 059-1r	A	4983	4983	1	G -> A	148	SNP (transition)	85.80%		0
Variants: VaAphrodite_C_FDSW210055 059-1r	A	4985	4985	1	T -> A	144	SNP (transversion)	73.60%		8.90E-284
Variants: VaAphrodite_C_FDSW210055 059-1r	A	4986	4986	1	T -> A	140	SNP (transversion)	26.40%		3.70E-85
Variants: VaAphrodite_C_FDSW210055 059-1r	C	4986	4986	1	T -> C	140	SNP (transition)	64.30%		2.90E-259
Variants: VaAphrodite_C_FDSW210055 059-1r	A	4987	4987	1	C -> A	140	SNP (transversion)	64.30%		2.90E-259
Variants: VaAphrodite_C_FDSW210055 059-1r	C	4988	4988	1	G -> C	139	SNP (transversion)	79.10%	6.9E-323	
Variants: VaAphrodite_C_FDSW210055 059-1r	AAAA	4992	4995	4	CGCG -> AAAA	107 -> 124	Substitution	72.9% -> 80.6%		1.90E-216

Variants: VaAphrodite_C_FDSW210055059-1r	CACAT	4996	5000	5	TGGCA -> CACAT	88 -> 104	Substitution	51.1% -> 59.6%	1.50E-122
Variants: VaAphrodite_C_FDSW210055059-1r	CA	5002	5003	2	TT -> CA	78 -> 83	Substitution	46.2% -> 49.4%	1.30E-75
Variants: VaAphrodite_C_FDSW210055059-1r	A	5004	5004	1	T -> A	74	SNP (transversion)	60.80%	2.90E-97
Variants: VaAphrodite_C_FDSW210055059-1r	GTG	5005	5007	3	TAC -> GTG	62 -> 71	Substitution	33.9% -> 40.8%	2.70E-43
Variants: VaAphrodite_C_FDSW210055059-1r	TC	5009	5010	2	AT -> TC	60	Substitution	25.0% -> 26.7%	1.50E-27
Variants: VaAphrodite_C_FDSW210055059-1r	T	5011	5011	1	G -> T	56	SNP (transversion)	26.80%	4.60E-25
Variants: VaAphrodite_C_FDSW210055059-1r	C	5579	5579	1	G -> C	66	SNP (transversion)	100.00%	6.30E-212
Variants: VaAphrodite_C_FDSW210055059-1r	T	5581	5581	1	A -> T	65	SNP (transversion)	100.00%	1.00E-208
Variants: VaAphrodite_C_FDSW210055059-1r	CG	5583	5584	2	TA -> CG	62 -> 64	Substitution	98.40%	4.00E-199
Variants: VaAphrodite_C_FDSW210055059-1r	C	5607	5607	1	A -> C	49	SNP (transversion)	30.60%	4.80E-32
Variants: VaAphrodite_C_FDSW210055059-1r	TT	5609	5610	2	AA -> TT	56 -> 59	Substitution	41.1% -> 45.8%	1.50E-56
Variants: VaAphrodite_C_FDSW210055059-1r	AA	5612	5613	2	GC -> AA	57 -> 60	Substitution	50.9% -> 53.3%	7.10E-63
Variants: VaAphrodite_C_FDSW210055059-1r	GGG	5615	5617	3	TTA -> GGG	61 -> 65	Substitution	50.8% -> 56.9%	8.20E-70
Variants: VaAphrodite_C_FDSW210055059-1r	A	5618	5618	1	G -> A	64	SNP (transition)	65.60%	1.20E-105
Variants: VaAphrodite_C_FDSW210055059-1r	T	5618	5618	1	G -> T	64	SNP (transversion)	34.40%	7.70E-50
Variants: VaAphrodite_C_FDSW210055059-1r	GT	5619	5620	2	TC -> GT	64	Substitution	98.40%	1.60E-173
Variants: VaAphrodite_C_FDSW210055059-1r	C	5621	5621	1	G -> C	64	SNP (transversion)	64.10%	1.80E-102
Variants: VaAphrodite_C_FDSW210055059-1r	T	5622	5622	1	C -> T	64	SNP (transition)	100.00%	4.00E-167
Variants: VaAphrodite_C_FDSW210055059-1r	G	5623	5623	1	C -> G	68	SNP (transversion)	70.60%	3.20E-118
Variants: VaAphrodite_C_FDSW210055059-1r	AA	5624	5625	2	TT -> AA	65 -> 68	Substitution	95.6% -> 96.9%	5.00E-165
Variants: VaAphrodite_C_FDSW210055059-1r	A	5626	5626	1	G -> A	73	SNP (transition)	74.00%	6.00E-124
Variants: VaAphrodite_C_FDSW210055059-1r	T	5632	5632	1	A -> T	84	SNP (transversion)	94.00%	7.60E-183

Variants: VaAphrodite_C_FDSW210055 059-1r	TATG	5633	5636	4	ATCA -> TATG	85 -> 86	Substitution	73.3% -> 77.6%	3.90E-134
Variants: VaAphrodite_C_FDSW210055 059-1r	A	5637	5637	1	T -> A	84	SNP (transversion)	94.00%	9.60E-191
Variants: VaAphrodite_C_FDSW210055 059-1r	A	5638	5638	1	T -> A	85	SNP (transversion)	78.80%	3.60E-150
Variants: VaAphrodite_C_FDSW210055 059-1r	AG	5645	5646	2	TC -> AG	106 -> 112	Substitution	72.3% -> 76.4%	9.60E-184
Variants: VaAphrodite_C_FDSW210055059-1r		5648	5648	1	(C)3 -> (C)2	130	Deletion (tandem repeat)	61.50%	1.20E-61
Variants: VaAphrodite_C_FDSW210055 059-1r	T	5653	5653	1	G -> T	172	SNP (transversion)	59.30%	9.80E-217
Variants: VaAphrodite_C_FDSW210055 059-1r	GC	5658	5657	0	#NAME?	201	Insertion	49.30%	7.20E-209
Variants: VaAres1_A_FDSW210055060-2r	TGGT	79	82	4	CCTG -> TGGT	55 -> 69	Substitution	73.9% -> 81.8%	1.70E-64
Variants: VaAres1_A_FDSW210055060-2r	G	84	84	1	A -> G	45	SNP (transition)	80.00%	1.80E-49
Variants: VaAres1_A_FDSW210055060-2r	G	86	86	1	C -> G	44	SNP (transversion)	100.00%	1.60E-75
Variants: VaAres1_A_FDSW210055060-2r	GGG	87	89	3	ATC -> GGG	40 -> 44	Substitution	79.5% -> 81.0%	4.00E-44
Variants: VaAres1_A_FDSW210055060-2r	C	91	91	1	T -> C	39	SNP (transition)	82.10%	5.30E-48
Variants: VaAres1_A_FDSW210055060-2r	GGA	93	95	3	CAC -> GGA	29 -> 31	Substitution	51.7% -> 58.1%	4.60E-14
Variants: VaAres1_A_FDSW210055060-2r	T	97	97	1	A -> T	27	SNP (transversion)	63.00%	4.20E-21
Variants: VaAres1_A_FDSW210055060-2r	C	102	102	1	T -> C	25	SNP (transition)	44.00%	4.80E-15
Variants: VaAres1_A_FDSW210055060-2r	T	108	108	1	A -> T	47	SNP (transversion)	25.50%	1.20E-23
Variants: VaAres1_A_FDSW210055060-2r	G	120	120	1	A -> G	88	SNP (transition)	26.10%	2.80E-17
Variants: VaAres1_A_FDSW210055060-2r	T	126	126	1	C -> T	109	SNP (transition)	34.90%	1.60E-47
Variants: VaAres1_A_FDSW210055060-2r	T	129	129	1	C -> T	117	SNP (transition)	65.80%	1.00E-138
Variants: VaAres1_A_FDSW210055060-2r	G	132	132	1	C -> G	130	SNP (transversion)	37.70%	6.00E-92
Variants: VaAres1_A_FDSW210055060-2r	T	144	144	1	C -> T	177	SNP (transition)	33.30%	8.50E-95
Variants: VaAres1_A_FDSW210055060-2r	G	150	150	1	C -> G	201	SNP (transversion)	26.40%	4.60E-100

Variants: VaAres1_A_FDSW210055060-2r	G	153	153	1	A -> G	217	SNP (transition)	27.60%	1.60E-108
Variants: VaAres1_A_FDSW210055060-2r	A	159	159	1	G -> A	246	SNP (transition)	69.90%	0
Variants: VaAres1_A_FDSW210055060-2r	A	165	165	1	T -> A	260	SNP (transversion)	35.40%	1.00E-149
Variants: VaAres1_A_FDSW210055060-2r	A	183	183	1	G -> A	324	SNP (transition)	29.90%	5.70E-159
Variants: VaAres1_A_FDSW210055060-2r	A	192	192	1	G -> A	353	SNP (transition)	38.20%	1.1E-318
Variants: VaAres1_A_FDSW210055060-2r	A	201	201	1	G -> A	383	SNP (transition)	26.10%	7.40E-167
Variants: VaAres1_A_FDSW210055060-2r	G	216	216	1	A -> G	428	SNP (transition)	30.10%	2.40E-288
Variants: VaAres1_A_FDSW210055060-2r	C	225	225	1	A -> C	450	SNP (transversion)	25.30%	6.50E-211
Variants: VaAres1_A_FDSW210055060-2r	T	225	225	1	A -> T	450	SNP (transversion)	36.70%	1.40E-286
Variants: VaAres1_A_FDSW210055060-2r	T	227	227	1	C -> T	455	SNP (transition)	29.20%	1.1E-321
Variants: VaAres1_A_FDSW210055060-2r	C	237	237	1	T -> C	484	SNP (transition)	25.80%	2.60E-170
Variants: VaAres1_A_FDSW210055060-2r	T	239	239	1	C -> T	490	SNP (transition)	28.40%	0
Variants: VaAres1_A_FDSW210055060-2r	C	240	240	1	T -> C	488	SNP (transition)	34.60%	2.30E-288
Variants: VaAres1_A_FDSW210055060-2r	A	294	294	1	G -> A	490	SNP (transition)	46.90%	0
Variants: VaAres1_A_FDSW210055060-2r	A	303	303	1	T -> A	493	SNP (transversion)	26.40%	7.50E-243
Variants: VaAres1_A_FDSW210055060-2r	G	306	306	1	A -> G	491	SNP (transition)	26.10%	1.10E-225
Variants: VaAres1_A_FDSW210055060-2r	A	357	357	1	G -> A	586	SNP (transition)	48.00%	0
Variants: VaAres1_A_FDSW210055060-2r	G	363	363	1	A -> G	603	SNP (transition)	50.70%	0
Variants: VaAres1_A_FDSW210055060-2r	C	369	369	1	T -> C	613	SNP (transition)	82.70%	0
Variants: VaAres1_A_FDSW210055060-2r	A	371	371	1	G -> A	625	SNP (transition)	52.80%	0
Variants: VaAres1_A_FDSW210055060-2r	G	387	387	1	C -> G	690	SNP (transversion)	56.20%	0
Variants: VaAres1_A_FDSW210055060-2r	A	393	393	1	G -> A	700	SNP (transition)	25.10%	4.9E-324

Variants: VaAres1_A_FDSW210055060-2r	C	393	393	1	G -> C	700	SNP (transversion)	48.40%	0
Variants: VaAres1_A_FDSW210055060-2r	C	396	396	1	A -> C	712	SNP (transversion)	32.90%	0
Variants: VaAres1_A_FDSW210055060-2r	G	420	420	1	A -> G	778	SNP (transition)	29.90%	0
Variants: VaAres1_A_FDSW210055060-2r	T	420	420	1	A -> T	778	SNP (transversion)	35.60%	0
Variants: VaAres1_A_FDSW210055060-2r	A	422	422	1	T -> A	775	SNP (transversion)	35.20%	0
Variants: VaAres1_A_FDSW210055060-2r	A	423	423	1	G -> A	779	SNP (transition)	64.60%	0
Variants: VaAres1_A_FDSW210055060-2r	A	426	426	1	G -> A	783	SNP (transition)	63.60%	0
Variants: VaAres1_A_FDSW210055060-2r	G	438	438	1	A -> G	827	SNP (transition)	50.80%	0
Variants: VaAres1_A_FDSW210055060-2r	A	456	456	1	G -> A	851	SNP (transition)	48.60%	0
Variants: VaAres1_A_FDSW210055060-2r	G	469	469	1	C -> G	851	SNP (transversion)	26.30%	0
Variants: VaAres1_A_FDSW210055060-2r	C	489	489	1	T -> C	865	SNP (transition)	42.70%	0
Variants: VaAres1_A_FDSW210055060-2r	C	498	498	1	G -> C	848	SNP (transversion)	26.80%	0
Variants: VaAres1_A_FDSW210055060-2r	C	513	513	1	T -> C	830	SNP (transition)	51.80%	0
Variants: VaAres1_A_FDSW210055060-2r	C	516	516	1	T -> C	831	SNP (transition)	36.50%	0
Variants: VaAres1_A_FDSW210055060-2r	T	531	531	1	C -> T	834	SNP (transition)	28.50%	0
Variants: VaAres1_A_FDSW210055060-2r	C	537	537	1	G -> C	816	SNP (transversion)	59.20%	0
Variants: VaAres1_A_FDSW210055060-2r	G	540	540	1	A -> G	813	SNP (transition)	39.40%	0
Variants: VaAres1_A_FDSW210055060-2r	A	552	552	1	T -> A	786	SNP (transversion)	33.30%	0
Variants: VaAres1_A_FDSW210055060-2r	A	564	564	1	C -> A	779	SNP (transversion)	29.10%	0
Variants: VaAres1_A_FDSW210055060-2r	G	588	588	1	A -> G	707	SNP (transition)	34.50%	0
Variants: VaAres1_A_FDSW210055060-2r	C	663	663	1	T -> C	379	SNP (transition)	68.10%	0
Variants: VaAres1_A_FDSW210055060-2r	G	677	677	1	A -> G	286	SNP (transition)	69.20%	0

Variants: VaAres1_A_FDSW210055060-2r	C	684	684	1	T -> C	243	SNP (transition)	62.60%	0					
Variants: VaAres1_A_FDSW210055060-2r	A	687	687	1	G -> A	239	SNP (transition)	61.90%	0					
Variants: VaAres1_A_FDSW210055060-2r	T	690	690	1	A -> T	226	SNP (transversion)	69.90%	0					
Variants: VaAres1_A_FDSW210055060-2r	A	693	693	1	G -> A	213	SNP (transition)	69.00%	0					
Variants: VaAres1_A_FDSW210055060-2r	A	708	708	1	G -> A	150	SNP (transition)	69.30%	1.60E-294					
Variants: VaAres1_A_FDSW210055060-2r	T	723	723	1	C -> T	107	SNP (transition)	68.20%	2.30E-206					
Variants: VaAres1_A_FDSW210055060-2r	C	726	726	1	T -> C	93	SNP (transition)	67.70%	2.70E-184					
Variants: VaAres1_A_FDSW210055060-2r	AATC	738	741	4	CGCT -> AATC	62 -> 67	Substitution	32.8% -> 43.5%	8.20E-32					
Variants: VaAres1_A_FDSW210055060-2r	G	744	744	1	A -> G	61	SNP (transition)	50.80%	5.40E-64					
Variants: VaAres1_A_FDSW210055060-2r	AC	745	746	2	GA -> AC	62 -> 63	Substitution	62.9% -> 63.5%	1.10E-73					
Variants: VaAres1_A_FDSW210055060-2r	C	747	747	1	T -> C	67	SNP (transition)	31.30%	2.90E-38					
Variants: VaAres1_A_FDSW210055060-2r	G	750	750	1	A -> G	65	SNP (transition)	32.30%	4.40E-14					
Variants: VaAres1_A_FDSW210055060-2r	C	756	756	1	G -> C	66	SNP (transversion)	51.50%	1.60E-63					
Variants: VaAres1_A_FDSW210055060-2r	TCA	760	762	3	CTC -> TCA	84 -> 89	Substitution	26.2% -> 27.0%	1.50E-13					
Variants: VaAres1_A_FDSW210055060-2r	T	765	765	1	C -> T	99	SNP (transition)	26.30%	2.20E-19					
Variants: VaAres1_A_FDSW210055060-2r	A	768	768	1	T -> A	109	SNP (transversion)	60.60%	2.50E-148					
Variants: VaAres1_A_FDSW210055060-2r	T	790	790	1	G -> T	179	SNP (transversion)	28.50%	5.00E-73	R -> L	hypothetical protein CDS	hypothetica l protein	Substituti on	KLI69 879.1
Variants: VaAres1_A_FDSW210055060-2r	A	795	795	1	G -> A	201	SNP (transition)	53.20%	1.20E-230	A -> T	hypothetical protein CDS	hypothetica l protein	Substituti on	KLI69 879.1
Variants: VaAres1_A_FDSW210055060-2r	AAT	801	803	2	CAC -> AAT	227 -> 239	Substitution	75.3% -> 75.8%	0	H -> N	hypothetical protein CDS	hypothetica l protein	Substituti on	KLI69 879.1
Variants: VaAres1_A_FDSW210055060-2r	C	834	834	1	T -> C	328	SNP (transition)	75.30%	0	Y -> H	hypothetical protein CDS	hypothetica l protein	Substituti on	KLI69 879.1
Variants: VaAres1_A_FDSW210055060-2r	G	888	888	1	A -> G	450	SNP (transition)	32.40%	6.80E-259	I -> V	hypothetical protein CDS	hypothetica l protein	Substituti on	KLI69 879.1
Variants: VaAres1_A_FDSW210055060-2r	C	897	897	1	T -> C	437	SNP (transition)	59.30%	0	C -> R	hypothetical protein CDS	hypothetica l protein	Substituti on	KLI69 879.1

Variants: VaAres1_A_FDSW210055060-2r	C	900	900	1	G -> C	435	SNP (transversion)	59.10%	0	D -> H	hypothetical protein CDS	hypothetical protein	Substitution	KLI69879.1
Variants: VaAres1_A_FDSW210055060-2r	C	942	942	1	G -> C	445	SNP (transversion)	28.80%	6.90E-194	G -> R	hypothetical protein CDS	hypothetical protein	Substitution	KLI69879.1
Variants: VaAres1_A_FDSW210055060-2r	TGT	966	968	2	AGC -> TGT	404 -> 407	Substitution	44.8% -> 45.2%	4.20E-304	S -> C	hypothetical protein CDS	hypothetical protein	Substitution	KLI69879.1
Variants: VaAres1_A_FDSW210055060-2r	G	969	969	1	C -> G	406	SNP (transversion)	26.60%	2.40E-138	P -> A	hypothetical protein CDS	hypothetical protein	Substitution	KLI69879.1
Variants: VaAres1_A_FDSW210055060-2r	A	976	976	1	T -> A	421	SNP (transversion)	27.30%	3.40E-126	V -> E	hypothetical protein CDS	hypothetical protein	Substitution	KLI69879.1
Variants: VaAres1_A_FDSW210055060-2r	A	1008	1008	1	G -> A	416	SNP (transition)	30.80%	2.30E-135	V -> I	hypothetical protein CDS	hypothetical protein	Substitution	KLI69879.1
Variants: VaAres1_A_FDSW210055060-2r	C	1035	1035	1	G -> C	437	SNP (transversion)	25.40%	1.70E-161	G -> R	hypothetical protein CDS	hypothetical protein	Substitution	KLI69879.1
Variants: VaAres1_A_FDSW210055060-2r	A	1041	1041	1	G -> A	442	SNP (transition)	31.00%	4.90E-199	A -> T	hypothetical protein CDS	hypothetical protein	Substitution	KLI69879.1
Variants: VaAres1_A_FDSW210055060-2r	G	1083	1083	1	A -> G	451	SNP (transition)	31.90%	0	K -> E	hypothetical protein CDS	hypothetical protein	Substitution	KLI69879.1
Variants: VaAres1_A_FDSW210055060-2r	A	1100	1100	1	G -> A	435	SNP (transition)	30.80%	3.30E-274		hypothetical protein CDS	hypothetical protein	None	KLI69879.1
Variants: VaAres1_A_FDSW210055060-2r	A	1101	1101	1	G -> A	443	SNP (transition)	26.90%	2.80E-247					
Variants: VaAres1_A_FDSW210055060-2r	C	1104	1104	1	G -> C	446	SNP (transversion)	29.60%	6.10E-307					
Variants: VaAres1_A_FDSW210055060-2r	T	1116	1116	1	C -> T	451	SNP (transition)	29.70%	7.8E-312		hypothetical protein CDS	hypothetical protein	None	KLI69880.1
Variants: VaAres1_A_FDSW210055060-2r	T	1119	1119	1	C -> T	454	SNP (transition)	28.90%	0		hypothetical protein CDS	hypothetical protein	None	KLI69880.1
Variants: VaAres1_A_FDSW210055060-2r	G	1135	1135	1	T -> G	453	SNP (transversion)	51.40%	0	F -> V	hypothetical protein CDS	hypothetical protein	Substitution	KLI69880.1
Variants: VaAres1_A_FDSW210055060-2r	TGTG	1173	1176	3	CATA -> TGTG	421 -> 423	Substitution	34.5% -> 35.0%	0	TI -> TV	hypothetical protein CDS	hypothetical protein	Substitution	KLI69880.1
Variants: VaAres1_A_FDSW210055060-2r	C	1182	1182	1	T -> C	416	SNP (transition)	35.30%	0		hypothetical protein CDS	hypothetical protein	None	KLI69880.1
Variants: VaAres1_A_FDSW210055060-2r	C	1200	1200	1	T -> C	390	SNP (transition)	29.20%	8.80E-299		hypothetical protein CDS	hypothetical protein	None	KLI69880.1
Variants: VaAres1_A_FDSW210055060-2r	G	1203	1203	1	A -> G	389	SNP (transition)	30.60%	1.4E-314		hypothetical protein CDS	hypothetical protein	None	KLI69880.1
Variants: VaAres1_A_FDSW210055060-2r	G	1215	1215	1	A -> G	361	SNP (transition)	53.70%	0		hypothetical protein CDS	hypothetical protein	None	KLI69880.1
Variants: VaAres1_A_FDSW210055060-2r	G	1218	1218	1	A -> G	350	SNP (transition)	40.90%	0		hypothetical protein CDS	hypothetical protein	None	KLI69880.1
Variants: VaAres1_A_FDSW210055060-2r	G	1221	1221	1	C -> G	338	SNP (transversion)	39.30%	0		hypothetical protein CDS	hypothetical protein	None	KLI69880.1

Variants: VaAres1_A_FDSW210055060-2r	GG	1232	1233	2	AC -> GG	292 -> 299	Substitution	34.1% -> 34.9%	3.50E-257	N -> R	hypothetical protein CDS	hypothetical protein	Substitution	KLI69880.1
Variants: VaAres1_A_FDSW210055060-2r	T	1235	1235	1	C -> T	289	SNP (transition)	35.30%	3.50E-257	A -> V	hypothetical protein CDS	hypothetical protein	Substitution	KLI69880.1
Variants: VaAres1_A_FDSW210055060-2r	G	1269	1269	1	A -> G	190	SNP (transition)	54.70%	9.40E-299	I -> M	hypothetical protein CDS	hypothetical protein	Substitution	KLI69880.1
Variants: VaAres1_A_FDSW210055060-2r	G	1275	1275	1	A -> G	169	SNP (transition)	56.80%	3.70E-278		hypothetical protein CDS	hypothetical protein	None	KLI69880.1
Variants: VaAres1_A_FDSW210055060-2r	T	1287	1287	1	A -> T	138	SNP (transversion)	29.00%	8.30E-90		hypothetical protein CDS	hypothetical protein	None	KLI69880.1
Variants: VaAres1_A_FDSW210055060-2r	G	1302	1302	1	A -> G	99	SNP (transition)	43.40%	6.80E-123		hypothetical protein CDS	hypothetical protein	None	KLI69880.1
Variants: VaAres1_A_FDSW210055060-2r	G	1320	1320	1	T -> G	73	SNP (transversion)	26.00%	1.90E-42		hypothetical protein CDS	hypothetical protein	None	KLI69880.1
Variants: VaAres1_A_FDSW210055060-2r	T	1322	1322	1	A -> T	68	SNP (transversion)	25.00%	2.10E-34	E -> V	hypothetical protein CDS	hypothetical protein	Substitution	KLI69880.1
Variants: VaAres1_A_FDSW210055060-2r	AAGTT	1324	1328	4	CACAC -> AAGTT	62 -> 65	Substitution	67.7% -> 71.0%	1.40E-138	HT -> KF	hypothetical protein CDS	hypothetical protein	Substitution	KLI69880.1
Variants: VaAres1_A_FDSW210055060-2r	AGTGCC	1330	1335	5	TTAACA -> AGTGCC	61 -> 64	Substitution	60.9% -> 70.5%	1.30E-119	LT -> SA	hypothetical protein CDS	hypothetical protein	Substitution	KLI69880.1
Variants: VaAres1_A_FDSW210055060-2r	TCGCT	1339	1343	5	CGTTA -> TCGCT	54 -> 63	Substitution	32.8% -> 39.3%	2.40E-53		hypothetical protein CDS	hypothetical protein	Extension	KLI69880.1
Variants: VaAres1_A_FDSW210055060-2r	T	1345	1345	1	G -> T	52	SNP (transversion)	32.70%	1.40E-48					
Variants: VaAres1_A_FDSW210055060-2r	TT	1347	1348	2	AA -> TT	48 -> 51	Substitution	39.6% -> 45.1%	5.80E-58					
Variants: VaAres1_A_FDSW210055060-2r	AA	1351	1352	2	GG -> AA	45 -> 47	Substitution	26.7% -> 31.9%	4.50E-31					
Variants: VaAres1_A_FDSW210055060-2r	T	2097	2097	1	C -> T	15	SNP (transition)	26.70%	2.20E-12		hypothetical protein CDS	hypothetical protein	None	KLI69881.1
Variants: VaAres1_A_FDSW210055060-2r	TTTACA	2101	2106	6	AAGTTT -> TTTACA	19 -> 21	Substitution	47.6% -> 52.6%	9.20E-30	KF -> FT	hypothetical protein CDS	hypothetical protein	Substitution	KLI69881.1
Variants: VaAres1_A_FDSW210055060-2r	GC	2108	2109	2	CA -> GC	20 -> 24	Substitution	55.0% -> 62.5%	5.30E-34	A -> G	hypothetical protein CDS	hypothetical protein	Substitution	KLI69881.1
Variants: VaAres1_A_FDSW210055060-2r	AA	2111	2112	2	TC -> AA	23	Substitution	65.20%	4.90E-46	I -> K	hypothetical protein CDS	hypothetical protein	Substitution	KLI69881.1
Variants: VaAres1_A_FDSW210055060-2r	CCGAAAC GGATGA	2114	2126	12	ATTTTGAAAAAAT -> CCGAAACGGATGA	26 -> 34	Substitution	67.9% -> 81.3%	8.90E-61	DFEKI -> AETDD	hypothetical protein CDS	hypothetical protein	Substitution	KLI69881.1
Variants: VaAres1_A_FDSW210055060-2r	AA	2128	2129	2	GC -> AA	33 -> 34	Substitution	88.2% -> 93.9%	4.70E-93	A -> N	hypothetical protein CDS	hypothetical protein	Substitution	KLI69881.1
Variants: VaAres1_A_FDSW210055060-2r	CAC	2131	2133	2	TAA -> CAC	33	Substitution	97.0% -> 100.0%	5.20E-108		hypothetical protein CDS	hypothetical protein	Extension	KLI69881.1
Variants: VaAres1_A_FDSW210055060-2r	T	2135	2135	1	A -> T	33	SNP (transversion)	100.00%	6.30E-113					

Variants: VaAres1_A_FDSW210055060-2r	CTG	2137	2139	3	AGC -> CTG	33 -> 34	Substitution	100.00%	1.30E-109
Variants: VaAres1_A_FDSW210055060-2r	AG	2141	2142	2	TT -> AG	35 -> 37	Substitution	97.1% -> 97.3%	1.00E-119
Variants: VaAres1_A_FDSW210055060-2r	G	2145	2145	1	A -> G	38	SNP (transition)	97.40%	6.00E-125
Variants: VaAres1_A_FDSW210055060-2r	T	2147	2147	1	A -> T	38	SNP (transversion)	100.00%	1.00E-133
Variants: VaAres1_A_FDSW210055060-2r	G	2149	2148	0	#NAME?	40	Insertion	100.00%	1.00E-140
Variants: VaAres1_A_FDSW210055060-2r	GAG	2902	2904	3	TGA -> GAG	11 -> 14	Substitution	72.7% -> 84.6%	1.60E-14
Variants: VaAres1_A_FDSW210055060-2r	GGA	2906	2908	3	TCG -> GGA	9 -> 10	Substitution	66.7% -> 70.0%	5.00E-09
Variants: VaAres1_A_FDSW210055060-2r	G	2910	2910	1	A -> G	8	SNP (transition)	62.50%	5.30E-07
Variants: VaAres1_A_FDSW210055060-2r		3097	3097	1	#NAME?	145	Deletion	44.10%	3.50E-68
Variants: VaAres1_A_FDSW210055060-2r	AG	3451	3452	2	GT -> AG	15	Substitution	26.7% -> 33.3%	5.30E-08
Variants: VaAres1_A_FDSW210055060-2r	G	3456	3456	1	T -> G	23	SNP (transversion)	39.10%	7.20E-13
Variants: VaAres1_A_FDSW210055060-2r	TTT	3458	3460	3	AAG -> TTT	23 -> 27	Substitution	29.6% -> 34.8%	1.90E-10
Variants: VaAres1_A_FDSW210055060-2r	T	3659	3659	1	G -> T	140	SNP (transversion)	52.90%	2.90E-226
Variants: VaAres1_A_FDSW210055060-2r	C	3758	3758	1	A -> C	159	SNP (transversion)	48.40%	6.60E-216
Variants: VaAres1_A_FDSW210055060-2r	G	3778	3778	1	A -> G	163	SNP (transition)	51.50%	6.60E-247
Variants: VaAres1_A_FDSW210055060-2r	T	3780	3780	1	C -> T	163	SNP (transition)	51.50%	6.60E-247
Variants: VaAres1_A_FDSW210055060-2r	C	3852	3852	1	T -> C	178	SNP (transition)	51.10%	6.70E-267
Variants: VaAres1_A_FDSW210055060-2r	G	3864	3864	1	A -> G	167	SNP (transition)	46.70%	1.20E-232
Variants: VaAres1_A_FDSW210055060-2r	T	3922	3922	1	C -> T	143	SNP (transition)	46.90%	4.30E-180
Variants: VaAres1_A_FDSW210055060-2r	T	4022	4022	1	G -> T	30	SNP (transversion)	96.70%	7.50E-69
Variants: VaAres1_A_FDSW210055060-2r	A	4023	4023	1	T -> A	29	SNP (transversion)	51.70%	7.10E-26
Variants: VaAres1_A_FDSW210055060-2r	G	4023	4023	1	T -> G	29	SNP (transversion)	41.40%	4.90E-23

Variants: VaAres1_A_FDSW210055060-2r	T	4025	4025	1	C -> T	29	SNP (transition)	100.00%	2.50E-70					
Variants: VaAres1_A_FDSW210055060-2r	T	4027	4027	1	A -> T	27	SNP (transversion)	96.30%	4.30E-59					
Variants: VaAres1_A_FDSW210055060-2r	C	4029	4029	1	T -> C	24	SNP (transition)	41.70%	1.90E-20					
Variants: VaAres1_A_FDSW210055060-2r	TTG	4031	4033	3	CCA -> TTG	23 -> 24	Substitution	95.7% -> 95.8%	3.00E-52					
Variants: VaAres1_A_FDSW210055060-2r	G	4035	4035	1	C -> G	22	SNP (transversion)	50.00%	2.20E-22					
Variants: VaAres1_A_FDSW210055060-2r	T	4035	4035	1	C -> T	22	SNP (transition)	50.00%	4.20E-19					
Variants: VaAres1_A_FDSW210055060-2r	T	4036	4036	1	G -> T	19	SNP (transversion)	100.00%	1.60E-42					
Variants: VaAres1_A_FDSW210055060-2r	C	4037	4037	1	G -> C	18	SNP (transversion)	33.30%	2.80E-10					
Variants: VaAres1_A_FDSW210055060-2r	T	4037	4037	1	G -> T	18	SNP (transversion)	61.10%	2.40E-19					
Variants: VaAres1_A_FDSW210055060-2r	TA	4038	4039	2	CT -> TA	16 -> 18	Substitution	93.8% -> 94.4%	1.60E-29					
Variants: VaAres1_A_FDSW210055060-2r	A	4040	4040	1	T -> A	16	SNP (transversion)	43.80%	5.20E-10					
Variants: VaAres1_A_FDSW210055060-2r	G	4040	4040	1	T -> G	16	SNP (transversion)	50.00%	3.10E-14					
Variants: VaAres1_A_FDSW210055060-2r	TGTTTAAG	4041	4048	8	GTACCGTT -> TGTTTAAG	12 -> 15	Substitution	91.7% -> 93.3%	1.30E-23					
Variants: VaAres1_A_FDSW210055060-2r	T	4050	4050	1	C -> T	8	SNP (transition)	100.00%	4.00E-19					
Variants: VaAres1_A_FDSW210055060-2r	G	4052	4052	1	C -> G	6	SNP (transversion)	100.00%	1.60E-14					
Variants: VaAres1_A_FDSW210055060-2r	C	4053	4053	1	T -> C	6	SNP (transition)	66.70%	9.40E-09					
Variants: VaAres1_A_FDSW210055060-2r	AT	4054	4055	2	GC -> AT	4 -> 6	Substitution	100.00%	6.30E-10					
Variants: VaAres1_A_FDSW210055060-2r	AGGTA	4339	4343	4	TTATC -> AGGTA	10 -> 15	Substitution	100.00%	1.00E-14	DK -> YL	hypothetical protein CDS	hypothetica l protein	Substituti on	KLI69 882.1
Variants: VaAres1_A_FDSW210055060-2r	TT	4345	4346	2	AA -> TT	24 -> 26	Substitution	95.8% -> 96.2%	9.40E-41	F -> N	hypothetical protein CDS	hypothetica l protein	Substituti on	KLI69 882.1
Variants: VaAres1_A_FDSW210055060-2r	CCA	4370	4372	3	GGC -> CCA	167 -> 183	Substitution	99.4% -> 99.5%	0	GQ -> VE	hypothetical protein CDS	hypothetica l protein	Substituti on	KLI69 882.1
Variants: VaAres1_A_FDSW210055060-2r	TT	4374	4375	2	GA -> TT	194 -> 198	Substitution	99.50%	0	I -> K	hypothetical protein CDS	hypothetica l protein	Substituti on	KLI69 882.1
Variants: VaAres1_A_FDSW210055060-2r	TGGTTCAT GA	4378	4387	9	GCTTGGTGCT -> TGGTTCATGA	214 -> 258	Substitution	98.6% -> 98.8%	0	EHQA -> VMNH	hypothetical protein CDS	hypothetica l protein	Substituti on	KLI69 882.1

Variants: VaAres1_A_FDSW210055060-2r	T	4389	4389	1	A -> T	268	SNP (transversion)	100.00%	0		hypothetical protein CDS	hypothetical protein	None	KLI69882.1
Variants: VaAres1_A_FDSW210055060-2r	G	4392	4392	1	C -> G	280	SNP (transversion)	100.00%	0		hypothetical protein CDS	hypothetical protein	None	KLI69882.1
Variants: VaAres1_A_FDSW210055060-2r	G	4395	4395	1	A -> G	294	SNP (transition)	100.00%	0		hypothetical protein CDS	hypothetical protein	None	KLI69882.1
Variants: VaAres1_A_FDSW210055060-2r	GTCGTA	4398	4403	6	CGTCAC -> GTCGTA	314 -> 348	Substitution	99.0% -> 99.1%	0	MT -> YD	hypothetical protein CDS	hypothetical protein	Start Codon Loss	KLI69882.1
Variants: VaAres1_A_FDSW210055060-2r	ACA	4404	4406	3	GTT -> ACA	351 -> 365	Substitution	99.4% -> 99.5%	0					
Variants: VaAres1_A_FDSW210055060-2r	GT	4409	4410	2	TC -> GT	379 -> 384	Substitution	100.00%	0					
Variants: VaAres1_A_FDSW210055060-2r	GC	4414	4415	2	CT -> GC	404 -> 413	Substitution	100.00%	0					
Variants: VaAres1_A_FDSW210055060-2r	A	4417	4417	1	G -> A	429	SNP (transition)	99.50%	0					
Variants: VaAres1_A_FDSW210055060-2r	GG	4419	4420	2	CA -> GG	454 -> 464	Substitution	99.80%	0					
Variants: VaAres1_A_FDSW210055060-2r	AC	4423	4424	2	GA -> AC	489 -> 494	Substitution	99.60%	0					
Variants: VaAres1_A_FDSW210055060-2r	G	4427	4426	0	#NAME?	505	Insertion	100.00%	0					
Variants: VaAres1_A_FDSW210055060-2r	TG	4431	4432	2	GA -> TG	534 -> 538	Substitution	100.00%	0					
Variants: VaAres1_A_FDSW210055060-2r	A	4434	4433	0	#NAME?	553	Insertion	99.80%	0					
Variants: VaAres1_A_FDSW210055060-2r	CA	4435	4436	2	GT -> CA	568 -> 574	Substitution	100.00%	0					
Variants: VaAres1_A_FDSW210055060-2r	A	4973	4973	1	G -> A	108	SNP (transition)	73.10%	3.30E-235					
Variants: VaAres1_A_FDSW210055060-2r	C	4975	4974	0	+C	101	Insertion	77.20%	2.00E-243					
Variants: VaAres1_A_FDSW210055060-2r	G	4975	4974	0	#NAME?	97	Insertion	53.60%	9.80E-155					
Variants: VaAres1_A_FDSW210055060-2r	T	4975	4974	0	(T)3 -> (T)4	97	Insertion (tandem repeat)	25.80%	9.40E-53					
Variants: VaAres1_A_FDSW210055060-2r	AA	4975	4976	2	TT -> AA	92 -> 96	Substitution	51.1% -> 52.1%	1.30E-138					
Variants: VaAres1_A_FDSW210055060-2r	G	4978	4978	1	A -> G	88	SNP (transition)	51.10%	8.00E-133					
Variants: VaAres1_A_FDSW210055060-2r	A	4979	4979	1	G -> A	87	SNP (transition)	73.60%	6.40E-204					
Variants: VaAres1_A_FDSW210055060-2r	T	4980	4980	1	A -> T	87	SNP (transversion)	51.70%	1.20E-137					

Variants: VaAres1_A_FDSW210055060-2r	A	4981	4981	1	G -> A	80	SNP (transition)	68.80%	1.10E-161
Variants: VaAres1_A_FDSW210055060-2r	A	4982	4982	1	T -> A	79	SNP (transversion)	78.50%	2.00E-188
Variants: VaAres1_A_FDSW210055060-2r	A	4983	4983	1	G -> A	76	SNP (transition)	90.80%	3.40E-212
Variants: VaAres1_A_FDSW210055060-2r	C	4984	4984	1	A -> C	76	SNP (transversion)	51.30%	2.10E-115
Variants: VaAres1_A_FDSW210055060-2r	A	4985	4985	1	T -> A	75	SNP (transversion)	72.00%	1.30E-160
Variants: VaAres1_A_FDSW210055060-2r	A	4986	4986	1	T -> A	75	SNP (transversion)	26.70%	7.50E-41
Variants: VaAres1_A_FDSW210055060-2r	C	4986	4986	1	T -> C	75	SNP (transition)	66.70%	5.20E-151
Variants: VaAres1_A_FDSW210055060-2r	A	4988	4988	1	G -> A	65	SNP (transition)	56.90%	6.20E-112
Variants: VaAres1_A_FDSW210055060-2r	C	4988	4988	1	G -> C	65	SNP (transversion)	33.80%	1.20E-49
Variants: VaAres1_A_FDSW210055060-2r	A	4989	4989	1	C -> A	62	SNP (transversion)	67.70%	2.30E-123
Variants: VaAres1_A_FDSW210055060-2r	AA	4992	4993	2	CG -> AA	57 -> 58	Substitution	89.5% -> 89.7%	2.30E-156
Variants: VaAres1_A_FDSW210055060-2r	A	4994	4994	1	C -> A	55	SNP (transversion)	38.20%	1.00E-46
Variants: VaAres1_A_FDSW210055060-2r	ACA	4995	4997	3	GTG -> ACA	45 -> 52	Substitution	55.6% -> 61.5%	1.00E-75
Variants: VaAres1_A_FDSW210055060-2r	A	4998	4998	1	G -> A	43	SNP (transition)	25.60%	1.30E-19
Variants: VaAres1_A_FDSW210055060-2r	T	4998	4998	1	G -> T	43	SNP (transversion)	37.20%	1.60E-40
Variants: VaAres1_A_FDSW210055060-2r	T	4999	4999	1	C -> T	43	SNP (transition)	55.80%	7.90E-61
Variants: VaAres1_A_FDSW210055060-2r	C	5000	5000	1	A -> C	43	SNP (transversion)	37.20%	2.60E-45
Variants: VaAres1_A_FDSW210055060-2r	T	5000	5000	1	A -> T	43	SNP (transversion)	34.90%	4.60E-33
Variants: VaAres1_A_FDSW210055060-2r	A	5001	5001	1	T -> A	45	SNP (transversion)	35.60%	2.50E-35
Variants: VaAres1_A_FDSW210055060-2r	A	5002	5002	1	T -> A	43	SNP (transversion)	51.20%	2.60E-50
Variants: VaAres1_A_FDSW210055060-2r	A	5003	5003	1	T -> A	42	SNP (transversion)	35.70%	9.30E-29
Variants: VaAres1_A_FDSW210055060-2r	G	5003	5003	1	T -> G	42	SNP (transversion)	26.20%	2.10E-27

Variants: VaAres1_A_FDSW210055060-2r	G	5005	5005	1	T -> G	38	SNP (transversion)	36.80%	9.60E-40
Variants: VaAres1_A_FDSW210055060-2r	G	5006	5006	1	A -> G	32	SNP (transition)	28.10%	1.30E-17
Variants: VaAres1_A_FDSW210055060-2r	T	5006	5006	1	A -> T	32	SNP (transversion)	31.30%	6.40E-27
Variants: VaAres1_A_FDSW210055060-2r	T	5007	5007	1	C -> T	31	SNP (transition)	38.70%	5.40E-25
Variants: VaAres1_A_FDSW210055060-2r	C	5009	5009	1	A -> C	27	SNP (transversion)	29.60%	3.40E-15
Variants: VaAres1_A_FDSW210055060-2r	TG	5011	5012	2	GC -> TG	20 -> 22	Substitution	25.0% -> 31.8%	1.40E-07
Variants: VaAres1_A_FDSW210055060-2r	C	5579	5579	1	G -> C	19	SNP (transversion)	100.00%	3.20E-67
Variants: VaAres1_A_FDSW210055060-2r	T	5581	5581	1	A -> T	19	SNP (transversion)	100.00%	1.00E-57
Variants: VaAres1_A_FDSW210055060-2r	CG	5583	5584	2	TA -> CG	17 -> 18	Substitution	100.00%	3.20E-60
Variants: VaAres1_A_FDSW210055060-2r	GTC	5605	5607	3	TAA -> GTC	18 -> 19	Substitution	33.3% -> 36.8%	6.50E-08
Variants: VaAres1_A_FDSW210055060-2r	TT	5609	5610	2	AA -> TT	19	Substitution	36.80%	1.10E-08
Variants: VaAres1_A_FDSW210055060-2r	AA	5612	5613	2	GC -> AA	24	Substitution	54.20%	1.90E-29
Variants: VaAres1_A_FDSW210055060-2r	GGG	5615	5617	3	TTA -> GGG	26 -> 29	Substitution	53.8% -> 62.1%	2.30E-27
Variants: VaAres1_A_FDSW210055060-2r	A	5618	5618	1	G -> A	28	SNP (transition)	64.30%	1.30E-47
Variants: VaAres1_A_FDSW210055060-2r	T	5618	5618	1	G -> T	28	SNP (transversion)	32.10%	5.40E-20
Variants: VaAres1_A_FDSW210055060-2r	GT	5619	5620	2	TC -> GT	27 -> 28	Substitution	92.9% -> 96.3%	9.40E-66
Variants: VaAres1_A_FDSW210055060-2r	C	5621	5621	1	G -> C	28	SNP (transversion)	75.00%	1.20E-57
Variants: VaAres1_A_FDSW210055060-2r	T	5622	5622	1	C -> T	31	SNP (transition)	93.50%	4.60E-85
Variants: VaAres1_A_FDSW210055060-2r	G	5623	5623	1	C -> G	33	SNP (transversion)	66.70%	1.90E-58
Variants: VaAres1_A_FDSW210055060-2r	AAA	5624	5626	3	TTG -> AAA	31 -> 32	Substitution	74.2% -> 75.0%	7.80E-63
Variants: VaAres1_A_FDSW210055060-2r	TTATG	5632	5636	5	AATCA -> TTATG	37 -> 43	Substitution	65.0% -> 70.3%	3.30E-71
Variants: VaAres1_A_FDSW210055060-2r	AA	5637	5638	2	TT -> AA	44 -> 45	Substitution	72.7% -> 73.3%	1.10E-82

Variants: VaAres1_A_FDSW210055060-2r	AG	5645	5646	2	TC -> AG	54 -> 57	Substitution	70.2% -> 74.1%	1.40E-102
Variants: VaAres1_A_FDSW210055060-2r		5648	5648	1	(C)3 -> (C)2	65	Deletion (tandem repeat)	61.50%	2.40E-39
Variants: VaAres1_A_FDSW210055060-2r	T	5653	5653	1	G -> T	99	SNP (transversion)	57.60%	1.90E-126
Variants: VaAres1_A_FDSW210055060-2r	GC	5658	5657	0	#NAME?	115	Insertion	43.50%	1.10E-112
Variants: VaAres1_B_FDSW210055061-2r	TGGT	79	82	4	CCTG -> TGGT	63 -> 67	Substitution	85.7% -> 89.1%	1.40E-137
Variants: VaAres1_B_FDSW210055061-2r	G	84	84	1	A -> G	46	SNP (transition)	97.80%	4.60E-107
Variants: VaAres1_B_FDSW210055061-2r	GGGG	86	89	4	CATC -> GGGG	32 -> 39	Substitution	87.5% -> 89.7%	4.90E-58
Variants: VaAres1_B_FDSW210055061-2r	C	91	91	1	T -> C	30	SNP (transition)	80.00%	3.40E-38
Variants: VaAres1_B_FDSW210055061-2r	GGA	93	95	3	CAC -> GGA	26 -> 27	Substitution	73.1% -> 81.5%	2.10E-29
Variants: VaAres1_B_FDSW210055061-2r	T	97	97	1	A -> T	30	SNP (transversion)	50.00%	1.20E-19
Variants: VaAres1_B_FDSW210055061-2r	GAA	99	101	3	CGT -> GAA	30 -> 34	Substitution	28.1% -> 40.0%	2.30E-11
Variants: VaAres1_B_FDSW210055061-2r	C	102	102	1	T -> C	33	SNP (transition)	57.60%	3.10E-24
Variants: VaAres1_B_FDSW210055061-2r	T	126	126	1	C -> T	192	SNP (transition)	43.20%	6.40E-79
Variants: VaAres1_B_FDSW210055061-2r	T	129	129	1	C -> T	207	SNP (transition)	76.80%	2.00E-271
Variants: VaAres1_B_FDSW210055061-2r	G	132	132	1	C -> G	227	SNP (transversion)	34.80%	2.70E-120
Variants: VaAres1_B_FDSW210055061-2r	T	144	144	1	C -> T	291	SNP (transition)	43.60%	1.10E-157
Variants: VaAres1_B_FDSW210055061-2r	G	150	150	1	C -> G	325	SNP (transversion)	33.50%	2.10E-152
Variants: VaAres1_B_FDSW210055061-2r	A	159	159	1	G -> A	353	SNP (transition)	73.90%	0
Variants: VaAres1_B_FDSW210055061-2r	A	165	165	1	T -> A	379	SNP (transversion)	37.70%	2.90E-208
Variants: VaAres1_B_FDSW210055061-2r	A	183	183	1	G -> A	453	SNP (transition)	32.50%	6.70E-217
Variants: VaAres1_B_FDSW210055061-2r	A	192	192	1	G -> A	485	SNP (transition)	37.70%	0
Variants: VaAres1_B_FDSW210055061-2r	A	201	201	1	G -> A	517	SNP (transition)	26.30%	2.80E-240

Variants: VaAres1_B_FDSW210055061-2r	G	216	216	1	A -> G	579	SNP (transition)	28.20%	0
Variants: VaAres1_B_FDSW210055061-2r	C	225	225	1	A -> C	607	SNP (transversion)	25.50%	1.10E-286
Variants: VaAres1_B_FDSW210055061-2r	T	225	225	1	A -> T	607	SNP (transversion)	44.30%	0
Variants: VaAres1_B_FDSW210055061-2r	C	237	237	1	T -> C	650	SNP (transition)	31.50%	2.60E-198
Variants: VaAres1_B_FDSW210055061-2r	C	240	240	1	T -> C	661	SNP (transition)	38.30%	5.6E-319
Variants: VaAres1_B_FDSW210055061-2r	A	294	294	1	G -> A	625	SNP (transition)	44.30%	0
Variants: VaAres1_B_FDSW210055061-2r	A	303	303	1	T -> A	636	SNP (transversion)	25.20%	8.90E-279
Variants: VaAres1_B_FDSW210055061-2r	A	357	357	1	G -> A	884	SNP (transition)	58.40%	0
Variants: VaAres1_B_FDSW210055061-2r	G	363	363	1	A -> G	911	SNP (transition)	59.90%	0
Variants: VaAres1_B_FDSW210055061-2r	C	369	369	1	T -> C	923	SNP (transition)	80.00%	0
Variants: VaAres1_B_FDSW210055061-2r	A	371	371	1	G -> A	934	SNP (transition)	54.80%	0
Variants: VaAres1_B_FDSW210055061-2r	G	387	387	1	C -> G	1031	SNP (transversion)	55.70%	0
Variants: VaAres1_B_FDSW210055061-2r	C	393	393	1	G -> C	1051	SNP (transversion)	47.00%	0
Variants: VaAres1_B_FDSW210055061-2r	C	396	396	1	A -> C	1058	SNP (transversion)	35.90%	0
Variants: VaAres1_B_FDSW210055061-2r	G	420	420	1	A -> G	1134	SNP (transition)	32.00%	0
Variants: VaAres1_B_FDSW210055061-2r	T	420	420	1	A -> T	1134	SNP (transversion)	32.10%	0
Variants: VaAres1_B_FDSW210055061-2r	A	422	422	1	T -> A	1131	SNP (transversion)	32.00%	0
Variants: VaAres1_B_FDSW210055061-2r	A	423	423	1	G -> A	1135	SNP (transition)	62.90%	0
Variants: VaAres1_B_FDSW210055061-2r	A	426	426	1	G -> A	1136	SNP (transition)	62.40%	0
Variants: VaAres1_B_FDSW210055061-2r	G	438	438	1	A -> G	1153	SNP (transition)	48.70%	0
Variants: VaAres1_B_FDSW210055061-2r	A	456	456	1	G -> A	1137	SNP (transition)	50.80%	0
Variants: VaAres1_B_FDSW210055061-2r	C	462	462	1	T -> C	1128	SNP (transition)	25.40%	0

Variants: VaAres1_B_FDSW210055061-2r	C	489	489	1	T -> C	1106	SNP (transition)	43.70%	0
Variants: VaAres1_B_FDSW210055061-2r	C	498	498	1	G -> C	1082	SNP (transversion)	30.20%	0
Variants: VaAres1_B_FDSW210055061-2r	C	513	513	1	T -> C	1032	SNP (transition)	54.70%	0
Variants: VaAres1_B_FDSW210055061-2r	C	516	516	1	T -> C	1039	SNP (transition)	36.40%	0
Variants: VaAres1_B_FDSW210055061-2r	C	537	537	1	G -> C	1028	SNP (transversion)	62.70%	0
Variants: VaAres1_B_FDSW210055061-2r	G	540	540	1	A -> G	1027	SNP (transition)	42.70%	0
Variants: VaAres1_B_FDSW210055061-2r	A	552	552	1	T -> A	976	SNP (transversion)	33.10%	0
Variants: VaAres1_B_FDSW210055061-2r	A	564	564	1	C -> A	941	SNP (transversion)	31.10%	0
Variants: VaAres1_B_FDSW210055061-2r	G	588	588	1	A -> G	892	SNP (transition)	36.00%	0
Variants: VaAres1_B_FDSW210055061-2r	C	663	663	1	T -> C	465	SNP (transition)	72.50%	0
Variants: VaAres1_B_FDSW210055061-2r	G	677	677	1	A -> G	328	SNP (transition)	73.80%	0
Variants: VaAres1_B_FDSW210055061-2r	C	684	684	1	T -> C	285	SNP (transition)	67.00%	0
Variants: VaAres1_B_FDSW210055061-2r	A	687	687	1	G -> A	274	SNP (transition)	66.80%	0
Variants: VaAres1_B_FDSW210055061-2r	T	690	690	1	A -> T	263	SNP (transversion)	70.70%	0
Variants: VaAres1_B_FDSW210055061-2r	A	693	693	1	G -> A	249	SNP (transition)	70.30%	0
Variants: VaAres1_B_FDSW210055061-2r	A	708	708	1	G -> A	182	SNP (transition)	70.30%	0
Variants: VaAres1_B_FDSW210055061-2r	T	723	723	1	C -> T	140	SNP (transition)	72.10%	8.40E-259
Variants: VaAres1_B_FDSW210055061-2r	C	726	726	1	T -> C	123	SNP (transition)	76.40%	2.00E-273
Variants: VaAres1_B_FDSW210055061-2r	C	741	741	1	T -> C	91	SNP (transition)	26.40%	1.40E-29
Variants: VaAres1_B_FDSW210055061-2r	GAC	744	746	3	AGA -> GAC	81 -> 85	Substitution	31.7% -> 38.8%	2.70E-34
Variants: VaAres1_B_FDSW210055061-2r	C	747	747	1	T -> C	88	SNP (transition)	56.80%	1.10E-105
Variants: VaAres1_B_FDSW210055061-2r	C	749	749	1	T -> C	87	SNP (transition)	52.90%	1.00E-90

Variants: VaAres1_B_FDSW210055061-2r	C	750	750	1	A -> C	88	SNP (transversion)	27.30%	1.90E-39					
Variants: VaAres1_B_FDSW210055061-2r	G	750	750	1	A -> G	88	SNP (transition)	55.70%	3.20E-93					
Variants: VaAres1_B_FDSW210055061-2r	TTCGA	751	755	5	CCTTG -> TTCGA	80 -> 93	Substitution	25.0% -> 43.8%	2.20E-24					
Variants: VaAres1_B_FDSW210055061-2r	C	756	756	1	G -> C	82	SNP (transversion)	48.80%	3.70E-81					
Variants: VaAres1_B_FDSW210055061-2r	A	768	768	1	T -> A	120	SNP (transversion)	59.20%	1.20E-179					
Variants: VaAres1_B_FDSW210055061-2r	T	790	790	1	G -> T	229	SNP (transversion)	29.30%	1.50E-129	R -> L	hypothetical protein CDS	hypothetical protein	Substitution	KLI69879.1
Variants: VaAres1_B_FDSW210055061-2r	A	795	795	1	G -> A	248	SNP (transition)	51.20%	9.20E-296	A -> T	hypothetical protein CDS	hypothetical protein	Substitution	KLI69879.1
Variants: VaAres1_B_FDSW210055061-2r	AAT	801	803	2	CAC -> AAT	273 -> 286	Substitution	71.3% -> 74.7%	0	H -> N	hypothetical protein CDS	hypothetical protein	Substitution	KLI69879.1
Variants: VaAres1_B_FDSW210055061-2r	C	834	834	1	T -> C	434	SNP (transition)	75.60%	0	Y -> H	hypothetical protein CDS	hypothetical protein	Substitution	KLI69879.1
Variants: VaAres1_B_FDSW210055061-2r	G	888	888	1	A -> G	590	SNP (transition)	32.40%	0	I -> V	hypothetical protein CDS	hypothetical protein	Substitution	KLI69879.1
Variants: VaAres1_B_FDSW210055061-2r	C	897	897	1	T -> C	598	SNP (transition)	58.00%	0	C -> R	hypothetical protein CDS	hypothetical protein	Substitution	KLI69879.1
Variants: VaAres1_B_FDSW210055061-2r	C	900	900	1	G -> C	606	SNP (transversion)	58.40%	0	D -> H	hypothetical protein CDS	hypothetical protein	Substitution	KLI69879.1
Variants: VaAres1_B_FDSW210055061-2r	C	942	942	1	G -> C	561	SNP (transversion)	26.70%	4.00E-251	G -> R	hypothetical protein CDS	hypothetical protein	Substitution	KLI69879.1
Variants: VaAres1_B_FDSW210055061-2r	TGT	966	968	2	AGC -> TGT	540 -> 547	Substitution	43.0% -> 43.9%	0	S -> C	hypothetical protein CDS	hypothetical protein	Substitution	KLI69879.1
Variants: VaAres1_B_FDSW210055061-2r	G	969	969	1	C -> G	539	SNP (transversion)	30.80%	3.20E-240	P -> A	hypothetical protein CDS	hypothetical protein	Substitution	KLI69879.1
Variants: VaAres1_B_FDSW210055061-2r	A	976	976	1	T -> A	535	SNP (transversion)	32.90%	8.10E-226	V -> E	hypothetical protein CDS	hypothetical protein	Substitution	KLI69879.1
Variants: VaAres1_B_FDSW210055061-2r	A	1008	1008	1	G -> A	512	SNP (transition)	40.60%	2.70E-269	V -> I	hypothetical protein CDS	hypothetical protein	Substitution	KLI69879.1
Variants: VaAres1_B_FDSW210055061-2r	C	1035	1035	1	G -> C	558	SNP (transversion)	33.70%	7.8E-318	G -> R	hypothetical protein CDS	hypothetical protein	Substitution	KLI69879.1
Variants: VaAres1_B_FDSW210055061-2r	A	1041	1041	1	G -> A	557	SNP (transition)	41.50%	0	A -> T	hypothetical protein CDS	hypothetical protein	Substitution	KLI69879.1
Variants: VaAres1_B_FDSW210055061-2r	C	1049	1049	1	T -> C	554	SNP (transition)	31.60%	0		hypothetical protein CDS	hypothetical protein	None	KLI69879.1
Variants: VaAres1_B_FDSW210055061-2r	G	1083	1083	1	A -> G	566	SNP (transition)	42.00%	0	K -> E	hypothetical protein CDS	hypothetical protein	Substitution	KLI69879.1
Variants: VaAres1_B_FDSW210055061-2r	A	1100	1100	1	G -> A	572	SNP (transition)	43.20%	0		hypothetical protein CDS	hypothetical protein	None	KLI69879.1

Variants: VaAres1_B_FDSW210055061-2r	A	1101	1101	1	G -> A	596	SNP (transition)	37.20%	0					
Variants: VaAres1_B_FDSW210055061-2r	C	1104	1104	1	G -> C	596	SNP (transversion)	42.60%	0					
Variants: VaAres1_B_FDSW210055061-2r	T	1116	1116	1	C -> T	584	SNP (transition)	44.30%	0		hypothetical protein CDS	hypothetica l protein	None	KLI69 880.1
Variants: VaAres1_B_FDSW210055061-2r	T	1119	1119	1	C -> T	587	SNP (transition)	40.20%	0		hypothetical protein CDS	hypothetica l protein	None	KLI69 880.1
Variants: VaAres1_B_FDSW210055061-2r	G	1135	1135	1	T -> G	616	SNP (transversion)	64.00%	0	F -> V	hypothetical protein CDS	hypothetica l protein	Substituti on	KLI69 880.1
Variants: VaAres1_B_FDSW210055061-2r	TGTG	1173	1176	3	CATA -> TGTG	595 -> 599	Substitution	49.2% -> 49.7%	0	TI -> TV	hypothetical protein CDS	hypothetica l protein	Substituti on	KLI69 880.1
Variants: VaAres1_B_FDSW210055061-2r	C	1182	1182	1	T -> C	594	SNP (transition)	49.20%	0		hypothetical protein CDS	hypothetica l protein	None	KLI69 880.1
Variants: VaAres1_B_FDSW210055061-2r	G	1194	1194	1	A -> G	598	SNP (transition)	26.60%	0		hypothetical protein CDS	hypothetica l protein	None	KLI69 880.1
Variants: VaAres1_B_FDSW210055061-2r	G	1215	1215	1	A -> G	546	SNP (transition)	56.80%	0		hypothetical protein CDS	hypothetica l protein	None	KLI69 880.1
Variants: VaAres1_B_FDSW210055061-2r	G	1218	1218	1	A -> G	537	SNP (transition)	43.00%	0		hypothetical protein CDS	hypothetica l protein	None	KLI69 880.1
Variants: VaAres1_B_FDSW210055061-2r	G	1221	1221	1	C -> G	534	SNP (transversion)	42.30%	0		hypothetical protein CDS	hypothetica l protein	None	KLI69 880.1
Variants: VaAres1_B_FDSW210055061-2r	GG	1232	1233	2	AC -> GG	500 -> 501	Substitution	39.1% -> 39.2%	0	N -> R	hypothetical protein CDS	hypothetica l protein	Substituti on	KLI69 880.1
Variants: VaAres1_B_FDSW210055061-2r	T	1235	1235	1	C -> T	493	SNP (transition)	39.40%	0	A -> V	hypothetical protein CDS	hypothetica l protein	Substituti on	KLI69 880.1
Variants: VaAres1_B_FDSW210055061-2r	G	1269	1269	1	A -> G	334	SNP (transition)	63.80%	0	I -> M	hypothetical protein CDS	hypothetica l protein	Substituti on	KLI69 880.1
Variants: VaAres1_B_FDSW210055061-2r	G	1275	1275	1	A -> G	305	SNP (transition)	65.60%	0		hypothetical protein CDS	hypothetica l protein	None	KLI69 880.1
Variants: VaAres1_B_FDSW210055061-2r	T	1287	1287	1	A -> T	250	SNP (transversion)	44.00%	1.4E-312		hypothetical protein CDS	hypothetica l protein	None	KLI69 880.1
Variants: VaAres1_B_FDSW210055061-2r	T	1290	1290	1	C -> T	245	SNP (transition)	43.70%	3.00E-282		hypothetical protein CDS	hypothetica l protein	None	KLI69 880.1
Variants: VaAres1_B_FDSW210055061-2r	G	1293	1293	1	C -> G	235	SNP (transversion)	42.60%	2.10E-292	D -> E	hypothetical protein CDS	hypothetica l protein	Substituti on	KLI69 880.1
Variants: VaAres1_B_FDSW210055061-2r	ATTG	1296	1299	4	CAAC -> ATTG	215 -> 223	Substitution	40.4% -> 40.9%	3.10E-231	TN -> TL	hypothetical protein CDS	hypothetica l protein	Substituti on	KLI69 880.1
Variants: VaAres1_B_FDSW210055061-2r	G	1302	1302	1	A -> G	204	SNP (transition)	27.50%	1.80E-151		hypothetical protein CDS	hypothetica l protein	None	KLI69 880.1
Variants: VaAres1_B_FDSW210055061-2r	A	1305	1305	1	G -> A	194	SNP (transition)	40.70%	1.20E-213		hypothetical protein CDS	hypothetica l protein	None	KLI69 880.1
Variants: VaAres1_B_FDSW210055061-2r	A	1311	1311	1	G -> A	173	SNP (transition)	31.80%	5.90E-142		hypothetical protein CDS	hypothetica l protein	None	KLI69 880.1

Variants: VaAres1_B_FDSW210055061-2r	T	1314	1314	1	G -> T	163	SNP (transversion)	31.30%	2.00E-136		hypothetical protein CDS	hypothetica l protein	None	KLI69 880.1
Variants: VaAres1_B_FDSW210055061-2r	G	1317	1317	1	C -> G	149	SNP (transversion)	31.50%	4.70E-126	D -> E	hypothetical protein CDS	hypothetica l protein	Substituti on	KLI69 880.1
Variants: VaAres1_B_FDSW210055061-2r	GCT	1320	1322	3	TGA -> GCT	127 -> 139	Substitution	29.1% -> 34.5%	2.10E-97	TE -> TL	hypothetical protein CDS	hypothetica l protein	Substituti on	KLI69 880.1
Variants: VaAres1_B_FDSW210055061-2r	A	1324	1324	1	C -> A	121	SNP (transversion)	26.40%	8.90E-107	H -> N	hypothetical protein CDS	hypothetica l protein	Substituti on	KLI69 880.1
Variants: VaAres1_B_FDSW210055061-2r	AAGTT	1324	1328	4	CACAC -> AAGTT	108 -> 121	Substitution	67.8% -> 75.2%	2.80E-266	HT -> KF	hypothetical protein CDS	hypothetica l protein	Substituti on	KLI69 880.1
Variants: VaAres1_B_FDSW210055061-2r	AGTGCC	1330	1335	5	TTAACA -> AGTGCC	87 -> 98	Substitution	80.6% -> 82.8%	2.60E-236	LT -> SA	hypothetical protein CDS	hypothetica l protein	Substituti on	KLI69 880.1
Variants: VaAres1_B_FDSW210055061-2r	TCG	1339	1341	3	CGT -> TCG	68 -> 74	Substitution	61.8% -> 64.9%	8.30E-133	R -> S	hypothetical protein CDS	hypothetica l protein	Substituti on	KLI69 880.1
Variants: VaAres1_B_FDSW210055061-2r	CT	1342	1343	2	TA -> CT	58 -> 62	Substitution	58.1% -> 62.1%	3.20E-114		hypothetical protein CDS	hypothetica l protein	Extension	KLI69 880.1
Variants: VaAres1_B_FDSW210055061-2r	G	1343	1343	1	A -> G	58	SNP (transition)	31.00%	7.10E-51		hypothetical protein CDS	hypothetica l protein	Extension	KLI69 880.1
Variants: VaAres1_B_FDSW210055061-2r	C	1345	1345	1	G -> C	51	SNP (transversion)	25.50%	3.80E-37					
Variants: VaAres1_B_FDSW210055061-2r	T	1345	1345	1	G -> T	51	SNP (transversion)	70.60%	3.20E-114					
Variants: VaAres1_B_FDSW210055061-2r	TT	1347	1348	2	AA -> TT	40 -> 43	Substitution	95.0% -> 95.3%	7.80E-131					
Variants: VaAres1_B_FDSW210055061-2r	AAT	1351	1353	3	GGA -> AAT	36 -> 38	Substitution	83.3% -> 86.8%	1.50E-100					
Variants: VaAres1_B_FDSW210055061-2r	CGTC	1355	>135 7	>3	TAA -> CGTC	34 -> 35	Insertion	70.6% -> 76.5%	3.30E-74					
Variants: VaAres1_B_FDSW210055061-2r	TTA	1360	1362	2	ATG -> TTA	26	Substitution	69.20%	6.20E-54	M -> L	hypothetical protein CDS	hypothetica l protein	Start Codon Loss	KLI69 881.1
Variants: VaAres1_B_FDSW210055061-2r	CAAT	1366	1369	4	ATCG -> CAAT	18 -> 21	Substitution	25.0% -> 38.1%	4.90E-15		hypothetical protein CDS	hypothetica l protein	Truncatio n	KLI69 881.1
Variants: VaAres1_B_FDSW210055061-2r	C	2109	2109	1	A -> C	28	SNP (transversion)	25.00%	1.50E-20		hypothetical protein CDS	hypothetica l protein	None	KLI69 881.1
Variants: VaAres1_B_FDSW210055061-2r	AA	2111	2112	2	TC -> AA	28 -> 29	Substitution	37.9% -> 39.3%	8.50E-31	I -> K	hypothetical protein CDS	hypothetica l protein	Substituti on	KLI69 881.1
Variants: VaAres1_B_FDSW210055061-2r	CCGAAAC GGATGA	2114	2126	12	ATTTTGAAAAAAT -> CCGAAACGGATGA	26 -> 38	Substitution	42.3% -> 78.9%	2.40E-32	DFEKI -> AETDD	hypothetical protein CDS	hypothetica l protein	Substituti on	KLI69 881.1
Variants: VaAres1_B_FDSW210055061-2r	AA	2128	2129	2	GC -> AA	39	Substitution	82.10%	6.10E-96	A -> N	hypothetical protein CDS	hypothetica l protein	Substituti on	KLI69 881.1
Variants: VaAres1_B_FDSW210055061-2r	CAC	2131	2133	2	TAA -> CAC	39 -> 41	Substitution	87.2% -> 87.8%	5.70E-114		hypothetical protein CDS	hypothetica l protein	Extension	KLI69 881.1
Variants: VaAres1_B_FDSW210055061-2r	T	2135	2135	1	A -> T	41	SNP (transversion)	87.80%	3.00E-117					

Variants: VaAres1_B_FDSW210055061-2r	CTG	2137	2139	3	AGC -> CTG	41 -> 43	Substitution	87.8% -> 88.4%	1.90E-124
Variants: VaAres1_B_FDSW210055061-2r	AG	2141	2142	2	TT -> AG	44	Substitution	86.40%	1.10E-111
Variants: VaAres1_B_FDSW210055061-2r	G	2145	2145	1	A -> G	46	SNP (transition)	95.70%	1.00E-151
Variants: VaAres1_B_FDSW210055061-2r	T	2147	2147	1	A -> T	47	SNP (transversion)	95.70%	1.10E-150
Variants: VaAres1_B_FDSW210055061-2r	G	2149	2148	0	#NAME?	51	Insertion	96.10%	2.50E-159
Variants: VaAres1_B_FDSW210055061-2r	GAG	2902	2904	3	TGA -> GAG	11 -> 14	Substitution	90.9% -> 92.9%	1.10E-28
Variants: VaAres1_B_FDSW210055061-2r	GGA	2906	2908	3	TCG -> GGA	9 -> 11	Substitution	77.8% -> 81.8%	2.30E-17
Variants: VaAres1_B_FDSW210055061-2r	GGG	2910	2912	3	AAC -> GGG	6 -> 7	Substitution	66.7% -> 71.4%	9.40E-09
Variants: VaAres1_B_FDSW210055061-2r		3097	3097	1	#NAME?	181	Deletion	45.30%	4.30E-80
Variants: VaAres1_B_FDSW210055061-2r	G	3456	3456	1	T -> G	10	SNP (transversion)	60.00%	1.30E-11
Variants: VaAres1_B_FDSW210055061-2r	TT	3458	3459	2	AA -> TT	9	Substitution	44.40%	1.90E-07
Variants: VaAres1_B_FDSW210055061-2r	T	3659	3659	1	G -> T	169	SNP (transversion)	47.30%	3.50E-239
Variants: VaAres1_B_FDSW210055061-2r	C	3758	3758	1	A -> C	162	SNP (transversion)	54.90%	5.20E-265
Variants: VaAres1_B_FDSW210055061-2r	G	3778	3778	1	A -> G	164	SNP (transition)	45.70%	7.80E-208
Variants: VaAres1_B_FDSW210055061-2r	T	3780	3780	1	C -> T	162	SNP (transition)	46.30%	2.30E-208
Variants: VaAres1_B_FDSW210055061-2r	C	3852	3852	1	T -> C	162	SNP (transition)	54.90%	6.50E-274
Variants: VaAres1_B_FDSW210055061-2r	G	3864	3864	1	A -> G	163	SNP (transition)	44.20%	2.40E-205
Variants: VaAres1_B_FDSW210055061-2r	T	3922	3922	1	C -> T	136	SNP (transition)	41.20%	4.30E-141
Variants: VaAres1_B_FDSW210055061-2r	T	4022	4022	1	G -> T	28	SNP (transversion)	100.00%	1.00E-56
Variants: VaAres1_B_FDSW210055061-2r	A	4023	4023	1	T -> A	28	SNP (transversion)	60.70%	1.60E-32
Variants: VaAres1_B_FDSW210055061-2r	G	4023	4023	1	T -> G	28	SNP (transversion)	39.30%	3.10E-12
Variants: VaAres1_B_FDSW210055061-2r	T	4025	4025	1	C -> T	26	SNP (transition)	100.00%	1.00E-52

Variants: VaAres1_B_FDSW210055061-2r	T	4027	4027	1	A -> T	25	SNP (transversion)	100.00%	3.20E-48					
Variants: VaAres1_B_FDSW210055061-2r	TTG	4031	4033	3	CCA -> TTG	24	Substitution	100.00%	6.30E-44					
Variants: VaAres1_B_FDSW210055061-2r	T	4035	4035	1	C -> T	22	SNP (transition)	68.20%	1.60E-28					
Variants: VaAres1_B_FDSW210055061-2r	T	4036	4036	1	G -> T	22	SNP (transversion)	100.00%	2.50E-40					
Variants: VaAres1_B_FDSW210055061-2r	T	4037	4037	1	G -> T	21	SNP (transversion)	66.70%	7.10E-28					
Variants: VaAres1_B_FDSW210055061-2r		4038	4038	1	-C	21	Deletion	100.00%	1.60E-17					
Variants: VaAres1_B_FDSW210055061-2r	A	4040	4040	1	T -> A	20	SNP (transversion)	100.00%	1.00E-34					
Variants: VaAres1_B_FDSW210055061-2r	GTTTAA	4043	4048	6	ACCGTT -> GTTTAA	13 -> 17	Substitution	100.00%	2.00E-25					
Variants: VaAres1_B_FDSW210055061-2r	GTGGTA	4050	4055	6	CGCTGC -> GTGGTA	7 -> 10	Substitution	100.00%	7.90E-17					
Variants: VaAres1_B_FDSW210055061-2r	G	4059	4059	1	T -> G	5	SNP (transversion)	100.00%	3.20E-12					
Variants: VaAres1_B_FDSW210055061-2r	G	4339	4339	1	T -> G	4	SNP (transversion)	100.00%	4.00E-07	K -> T	hypothetical protein CDS	hypothetical protein	Substitution	KLI69882.1
Variants: VaAres1_B_FDSW210055061-2r	G	4342	4342	1	T -> G	24	SNP (transversion)	95.80%	1.20E-47	D -> A	hypothetical protein CDS	hypothetical protein	Substitution	KLI69882.1
Variants: VaAres1_B_FDSW210055061-2r	ACCACTTTG	4369	4375	>6	TGGCCGA -> ACCACTTTG	192 -> 259	Insertion	87.3% -> 97.5%	0		hypothetical protein CDS	hypothetical protein	Truncation	KLI69882.1
Variants: VaAres1_B_FDSW210055061-2r	T	4379	4379	1	C -> T	289	SNP (transition)	99.70%	0	A -> T	hypothetical protein CDS	hypothetical protein	Substitution	KLI69882.1
Variants: VaAres1_B_FDSW210055061-2r	CATGA	4381	4385	5	TGGTG -> CATGA	299 -> 313	Substitution	99.70%	0		hypothetical protein CDS	hypothetical protein	Truncation	KLI69882.1
Variants: VaAres1_B_FDSW210055061-2r	GG	4388	4389	2	CA -> GG	324 -> 335	Substitution	99.40%	0	PE -> PQ	hypothetical protein CDS	hypothetical protein	Substitution	KLI69882.1
Variants: VaAres1_B_FDSW210055061-2r	TGA	4392	4394	2	CGT -> TGA	369 -> 380	Substitution	100.00%	0	T -> S	hypothetical protein CDS	hypothetical protein	Substitution	KLI69882.1
Variants: VaAres1_B_FDSW210055061-2r	GT	4396	4397	2	AA -> GT	390 -> 393	Substitution	99.70%	0	F -> T	hypothetical protein CDS	hypothetical protein	Substitution	KLI69882.1
Variants: VaAres1_B_FDSW210055061-2r	A	4401	4401	1	C -> A	419	SNP (transversion)	99.80%	0	M -> V	hypothetical protein CDS	hypothetical protein	Start Codon Loss	KLI69882.1
Variants: VaAres1_B_FDSW210055061-2r	A	4404	4406	3	GTT -> A	461	Deletion	99.60%	0					
Variants: VaAres1_B_FDSW210055061-2r	GT	4409	4410	2	TC -> GT	508 -> 517	Substitution	99.60%	0					
Variants: VaAres1_B_FDSW210055061-2r	GC	4414	4415	2	CT -> GC	543 -> 550	Substitution	99.80%	0					

Variants: VaAres1_B_FDSW210055061-2r	A	4417	4417	1	G -> A	568	SNP (transition)	99.60%	0
Variants: VaAres1_B_FDSW210055061-2r	GG	4419	4420	2	CA -> GG	663 -> 675	Substitution	99.4% -> 99.5%	0
Variants: VaAres1_B_FDSW210055061-2r	AC	4423	4424	2	GA -> AC	700 -> 705	Substitution	99.40%	0
Variants: VaAres1_B_FDSW210055061-2r	G	4427	4426	0	#NAME?	729	Insertion	99.90%	0
Variants: VaAres1_B_FDSW210055061-2r	TG	4431	4432	2	GA -> TG	757 -> 762	Substitution	99.70%	0
Variants: VaAres1_B_FDSW210055061-2r	A	4434	4433	0	#NAME?	797	Insertion	99.90%	0
Variants: VaAres1_B_FDSW210055061-2r	CA	4435	4436	2	GT -> CA	810 -> 821	Substitution	99.80%	0
Variants: VaAres1_B_FDSW210055061-2r	A	4973	4973	1	G -> A	154	SNP (transition)	66.90%	0.00E+00
Variants: VaAres1_B_FDSW210055061-2r	C	4975	4974	0	+C	149	Insertion	74.50%	0.00E+00
Variants: VaAres1_B_FDSW210055061-2r	G	4975	4974	0	#NAME?	146	Insertion	46.60%	1.60E-182
Variants: VaAres1_B_FDSW210055061-2r	T	4975	4974	0	(T)3 -> (T)4	146	Insertion (tandem repeat)	30.10%	1.00E-90
Variants: VaAres1_B_FDSW210055061-2r	AA	4975	4976	2	TT -> AA	140 -> 143	Substitution	44.1% -> 44.3%	9.10E-165
Variants: VaAres1_B_FDSW210055061-2r	G	4978	4978	1	A -> G	138	SNP (transition)	42.00%	9.80E-147
Variants: VaAres1_B_FDSW210055061-2r	A	4979	4979	1	G -> A	139	SNP (transition)	64.00%	3.70E-256
Variants: VaAres1_B_FDSW210055061-2r	T	4980	4980	1	A -> T	138	SNP (transversion)	42.00%	1.60E-152
Variants: VaAres1_B_FDSW210055061-2r	A	4981	4981	1	G -> A	131	SNP (transition)	61.80%	2.90E-223
Variants: VaAres1_B_FDSW210055061-2r	C	4981	4981	1	G -> C	131	SNP (transversion)	26.70%	7.50E-74
Variants: VaAres1_B_FDSW210055061-2r	A	4982	4982	1	T -> A	130	SNP (transversion)	73.80%	5.40E-267
Variants: VaAres1_B_FDSW210055061-2r	A	4983	4983	1	G -> A	119	SNP (transition)	84.00%	4.80E-289
Variants: VaAres1_B_FDSW210055061-2r	C	4984	4984	1	A -> C	119	SNP (transversion)	43.70%	7.20E-133
Variants: VaAres1_B_FDSW210055061-2r	A	4985	4985	1	T -> A	114	SNP (transversion)	61.40%	7.70E-186
Variants: VaAres1_B_FDSW210055061-2r	A	4986	4986	1	T -> A	114	SNP (transversion)	28.10%	1.90E-68

Variants: VaAres1_B_FDSW210055061-2r	C	4986	4986	1	T -> C	114	SNP (transition)	57.90%	2.30E-179
Variants: VaAres1_B_FDSW210055061-2r	A	4988	4988	1	G -> A	112	SNP (transition)	48.20%	2.20E-146
Variants: VaAres1_B_FDSW210055061-2r	C	4988	4988	1	G -> C	112	SNP (transversion)	37.50%	1.10E-95
Variants: VaAres1_B_FDSW210055061-2r	A	4989	4989	1	C -> A	108	SNP (transversion)	67.60%	5.50E-184
Variants: VaAres1_B_FDSW210055061-2r	AA	4992	4993	2	CG -> AA	102 -> 103	Substitution	80.4% -> 82.5%	5.20E-234
Variants: VaAres1_B_FDSW210055061-2r	A	4994	4994	1	C -> A	102	SNP (transversion)	43.10%	3.60E-99
Variants: VaAres1_B_FDSW210055061-2r	A	4995	4995	1	G -> A	93	SNP (transition)	51.60%	3.00E-132
Variants: VaAres1_B_FDSW210055061-2r	C	4995	4995	1	G -> C	93	SNP (transversion)	26.90%	2.70E-48
Variants: VaAres1_B_FDSW210055061-2r	A	4996	4996	1	T -> A	92	SNP (transversion)	30.40%	7.10E-53
Variants: VaAres1_B_FDSW210055061-2r	C	4996	4996	1	T -> C	92	SNP (transition)	47.80%	5.80E-115
Variants: VaAres1_B_FDSW210055061-2r	A	4997	4997	1	G -> A	88	SNP (transition)	46.60%	1.30E-106
Variants: VaAres1_B_FDSW210055061-2r	C	4997	4997	1	G -> C	88	SNP (transversion)	30.70%	7.70E-54
Variants: VaAres1_B_FDSW210055061-2r	A	4998	4998	1	G -> A	85	SNP (transition)	29.40%	2.00E-49
Variants: VaAres1_B_FDSW210055061-2r	T	4998	4998	1	G -> T	85	SNP (transversion)	36.50%	2.20E-64
Variants: VaAres1_B_FDSW210055061-2r	T	4999	4999	1	C -> T	84	SNP (transition)	58.30%	1.90E-104
Variants: VaAres1_B_FDSW210055061-2r	C	5000	5000	1	A -> C	82	SNP (transversion)	34.10%	6.40E-63
Variants: VaAres1_B_FDSW210055061-2r	T	5000	5000	1	A -> T	82	SNP (transversion)	32.90%	1.90E-49
Variants: VaAres1_B_FDSW210055061-2r	A	5001	5001	1	T -> A	73	SNP (transversion)	34.20%	2.20E-51
Variants: VaAres1_B_FDSW210055061-2r	A	5002	5002	1	T -> A	69	SNP (transversion)	50.70%	4.90E-65
Variants: VaAres1_B_FDSW210055061-2r	A	5003	5003	1	T -> A	61	SNP (transversion)	29.50%	7.00E-29
Variants: VaAres1_B_FDSW210055061-2r	G	5004	5004	1	T -> G	59	SNP (transversion)	25.40%	1.00E-21
Variants: VaAres1_B_FDSW210055061-2r	G	5005	5005	1	T -> G	58	SNP (transversion)	27.60%	4.70E-30

Variants: VaAres1_B_FDSW210055061-2r	G	5006	5006	1	A -> G	58	SNP (transition)	31.00%	4.00E-31
Variants: VaAres1_B_FDSW210055061-2r	T	5006	5006	1	A -> T	58	SNP (transversion)	29.30%	5.50E-29
Variants: VaAres1_B_FDSW210055061-2r	T	5007	5007	1	C -> T	57	SNP (transition)	49.10%	3.20E-46
Variants: VaAres1_B_FDSW210055061-2r	C	5009	5009	1	A -> C	43	SNP (transversion)	34.90%	4.20E-24
Variants: VaAres1_B_FDSW210055061-2r	T	5011	5011	1	G -> T	40	SNP (transversion)	40.00%	8.90E-27
Variants: VaAres1_B_FDSW210055061-2r	G	5012	5012	1	C -> G	39	SNP (transversion)	28.20%	7.40E-17
Variants: VaAres1_B_FDSW210055061-2r	C	5014	5014	1	T -> C	35	SNP (transition)	28.60%	1.70E-16
Variants: VaAres1_B_FDSW210055061-2r	T	5016	5016	1	G -> T	36	SNP (transversion)	25.00%	3.50E-16
Variants: VaAres1_B_FDSW210055061-2r	C	5579	5579	1	G -> C	42	SNP (transversion)	97.60%	2.10E-134
Variants: VaAres1_B_FDSW210055061-2r	T	5581	5581	1	A -> T	42	SNP (transversion)	100.00%	6.30E-131
Variants: VaAres1_B_FDSW210055061-2r	CG	5583	5584	2	TA -> CG	39 -> 40	Substitution	100.00%	1.60E-125
Variants: VaAres1_B_FDSW210055061-2r	TT	5609	5610	2	AA -> TT	32 -> 35	Substitution	25.0% -> 31.4%	1.00E-17
Variants: VaAres1_B_FDSW210055061-2r	AA	5612	5613	2	GC -> AA	38 -> 39	Substitution	39.5% -> 41.0%	4.90E-43
Variants: VaAres1_B_FDSW210055061-2r	GGG	5615	5617	3	TTA -> GGG	43 -> 46	Substitution	53.5% -> 56.5%	6.00E-67
Variants: VaAres1_B_FDSW210055061-2r	A	5618	5618	1	G -> A	46	SNP (transition)	56.50%	2.20E-76
Variants: VaAres1_B_FDSW210055061-2r	T	5618	5618	1	G -> T	46	SNP (transversion)	43.50%	5.40E-46
Variants: VaAres1_B_FDSW210055061-2r	GT	5619	5620	2	TC -> GT	47	Substitution	95.7% -> 100.0%	3.40E-146
Variants: VaAres1_B_FDSW210055061-2r	C	5621	5621	1	G -> C	45	SNP (transversion)	66.70%	3.40E-85
Variants: VaAres1_B_FDSW210055061-2r	T	5622	5622	1	C -> T	44	SNP (transition)	93.20%	1.00E-123
Variants: VaAres1_B_FDSW210055061-2r	G	5623	5623	1	C -> G	43	SNP (transversion)	67.40%	1.60E-85
Variants: VaAres1_B_FDSW210055061-2r	AA	5624	5625	2	TT -> AA	45 -> 46	Substitution	91.1% -> 91.3%	1.20E-122
Variants: VaAres1_B_FDSW210055061-2r	A	5626	5626	1	G -> A	47	SNP (transition)	70.20%	8.50E-95

Variants: VaAres1_B_FDSW210055061-2r	T	5631	5631	1	G -> T	56	SNP (transversion)	25.00%	1.70E-17
Variants: VaAres1_B_FDSW210055061-2r	T	5632	5632	1	A -> T	54	SNP (transversion)	90.70%	1.60E-126
Variants: VaAres1_B_FDSW210055061-2r	TATG	5633	5636	4	ATCA -> TATG	50 -> 53	Substitution	71.7% -> 76.0%	7.50E-100
Variants: VaAres1_B_FDSW210055061-2r	A	5637	5637	1	T -> A	56	SNP (transversion)	83.90%	9.40E-118
Variants: VaAres1_B_FDSW210055061-2r	A	5638	5638	1	T -> A	57	SNP (transversion)	71.90%	7.10E-106
Variants: VaAres1_B_FDSW210055061-2r	AG	5645	5646	2	TC -> AG	63 -> 65	Substitution	72.3% -> 74.6%	1.20E-124
Variants: VaAres1_B_FDSW210055061-2r		5648	5648	1	(C)3 -> (C)2	71	Deletion (tandem repeat)	67.60%	6.70E-50
Variants: VaAres1_B_FDSW210055061-2r	T	5653	5653	1	G -> T	104	SNP (transversion)	57.70%	4.30E-139
Variants: VaAres1_B_FDSW210055061-2r	GC	5658	5657	0	#NAME?	132	Insertion	45.50%	2.00E-130
Variants: VaAres1_C_FDSW210055062-1r	TGGT	79	82	4	CCTG -> TGGT	94 -> 108	Substitution	85.1% -> 88.0%	6.80E-236
Variants: VaAres1_C_FDSW210055062-1r	G	84	84	1	A -> G	90	SNP (transition)	96.70%	1.20E-256
Variants: VaAres1_C_FDSW210055062-1r	GGGG	86	89	4	CATC -> GGGG	82 -> 85	Substitution	70.2% -> 84.7%	1.20E-150
Variants: VaAres1_C_FDSW210055062-1r	C	91	91	1	T -> C	82	SNP (transition)	68.30%	1.60E-147
Variants: VaAres1_C_FDSW210055062-1r	GGA	93	95	3	CAC -> GGA	82 -> 91	Substitution	56.0% -> 65.5%	2.50E-107
Variants: VaAres1_C_FDSW210055062-1r	T	97	97	1	A -> T	87	SNP (transversion)	46.00%	9.30E-88
Variants: VaAres1_C_FDSW210055062-1r	GAA	99	101	3	CGT -> GAA	82 -> 86	Substitution	29.3% -> 33.7%	4.60E-45
Variants: VaAres1_C_FDSW210055062-1r	C	102	102	1	T -> C	86	SNP (transition)	39.50%	2.10E-58
Variants: VaAres1_C_FDSW210055062-1r	C	103	103	1	G -> C	90	SNP (transversion)	25.60%	1.10E-41
Variants: VaAres1_C_FDSW210055062-1r	T	126	126	1	C -> T	203	SNP (transition)	32.50%	9.90E-60
Variants: VaAres1_C_FDSW210055062-1r	T	129	129	1	C -> T	222	SNP (transition)	67.60%	1.60E-241
Variants: VaAres1_C_FDSW210055062-1r	G	132	132	1	C -> G	235	SNP (transversion)	41.70%	2.30E-158
Variants: VaAres1_C_FDSW210055062-1r	T	144	144	1	C -> T	295	SNP (transition)	34.60%	2.70E-134

Variants: VaAres1_C_FDSW210055062-1r	G	150	150	1	C -> G	330	SNP (transversion)	25.50%	4.70E-131
Variants: VaAres1_C_FDSW210055062-1r	G	153	153	1	A -> G	348	SNP (transition)	29.30%	2.10E-196
Variants: VaAres1_C_FDSW210055062-1r	A	159	159	1	G -> A	393	SNP (transition)	72.50%	0
Variants: VaAres1_C_FDSW210055062-1r	A	165	165	1	T -> A	438	SNP (transversion)	36.80%	6.60E-280
Variants: VaAres1_C_FDSW210055062-1r	A	183	183	1	G -> A	524	SNP (transition)	34.00%	8.40E-302
Variants: VaAres1_C_FDSW210055062-1r	A	192	192	1	G -> A	567	SNP (transition)	35.10%	0
Variants: VaAres1_C_FDSW210055062-1r	A	201	201	1	G -> A	623	SNP (transition)	33.40%	0
Variants: VaAres1_C_FDSW210055062-1r	G	216	216	1	A -> G	698	SNP (transition)	35.00%	0
Variants: VaAres1_C_FDSW210055062-1r	C	225	225	1	A -> C	737	SNP (transversion)	30.90%	0
Variants: VaAres1_C_FDSW210055062-1r	T	225	225	1	A -> T	737	SNP (transversion)	32.30%	0
Variants: VaAres1_C_FDSW210055062-1r	T	227	227	1	C -> T	743	SNP (transition)	26.00%	0
Variants: VaAres1_C_FDSW210055062-1r	C	240	240	1	T -> C	771	SNP (transition)	34.60%	0
Variants: VaAres1_C_FDSW210055062-1r	A	294	294	1	G -> A	779	SNP (transition)	48.40%	0
Variants: VaAres1_C_FDSW210055062-1r	A	303	303	1	T -> A	804	SNP (transversion)	31.00%	0
Variants: VaAres1_C_FDSW210055062-1r	G	306	306	1	A -> G	811	SNP (transition)	29.50%	0
Variants: VaAres1_C_FDSW210055062-1r	A	315	315	1	G -> A	820	SNP (transition)	25.20%	0
Variants: VaAres1_C_FDSW210055062-1r	A	357	357	1	G -> A	1035	SNP (transition)	48.20%	0
Variants: VaAres1_C_FDSW210055062-1r	G	363	363	1	A -> G	1048	SNP (transition)	49.80%	0
Variants: VaAres1_C_FDSW210055062-1r	C	369	369	1	T -> C	1055	SNP (transition)	81.80%	0
Variants: VaAres1_C_FDSW210055062-1r	A	371	371	1	G -> A	1060	SNP (transition)	50.70%	0
Variants: VaAres1_C_FDSW210055062-1r	G	387	387	1	C -> G	1168	SNP (transversion)	56.30%	0
Variants: VaAres1_C_FDSW210055062-1r	C	393	393	1	G -> C	1192	SNP (transversion)	50.40%	0

Variants: VaAres1_C_FDSW210055062-1r	C	396	396	1	A -> C	1208	SNP (transversion)	32.30%	0
Variants: VaAres1_C_FDSW210055062-1r	G	420	420	1	A -> G	1328	SNP (transition)	29.90%	0
Variants: VaAres1_C_FDSW210055062-1r	T	420	420	1	A -> T	1328	SNP (transversion)	36.20%	0
Variants: VaAres1_C_FDSW210055062-1r	A	422	422	1	T -> A	1337	SNP (transversion)	36.30%	0
Variants: VaAres1_C_FDSW210055062-1r	A	423	423	1	G -> A	1345	SNP (transition)	65.10%	0
Variants: VaAres1_C_FDSW210055062-1r	A	426	426	1	G -> A	1350	SNP (transition)	64.20%	0
Variants: VaAres1_C_FDSW210055062-1r	G	438	438	1	A -> G	1396	SNP (transition)	48.50%	0
Variants: VaAres1_C_FDSW210055062-1r	A	456	456	1	G -> A	1334	SNP (transition)	46.60%	0
Variants: VaAres1_C_FDSW210055062-1r	G	469	469	1	C -> G	1293	SNP (transversion)	26.00%	0
Variants: VaAres1_C_FDSW210055062-1r	C	489	489	1	T -> C	1226	SNP (transition)	43.60%	0
Variants: VaAres1_C_FDSW210055062-1r	C	498	498	1	G -> C	1209	SNP (transversion)	29.40%	0
Variants: VaAres1_C_FDSW210055062-1r	C	513	513	1	T -> C	1179	SNP (transition)	51.00%	0
Variants: VaAres1_C_FDSW210055062-1r	C	516	516	1	T -> C	1187	SNP (transition)	34.00%	0
Variants: VaAres1_C_FDSW210055062-1r	T	531	531	1	C -> T	1166	SNP (transition)	28.80%	0
Variants: VaAres1_C_FDSW210055062-1r	C	537	537	1	G -> C	1156	SNP (transversion)	56.40%	0
Variants: VaAres1_C_FDSW210055062-1r	G	540	540	1	A -> G	1149	SNP (transition)	35.20%	0
Variants: VaAres1_C_FDSW210055062-1r	A	552	552	1	T -> A	1126	SNP (transversion)	38.00%	0
Variants: VaAres1_C_FDSW210055062-1r	A	564	564	1	C -> A	1091	SNP (transversion)	29.30%	0
Variants: VaAres1_C_FDSW210055062-1r	G	588	588	1	A -> G	981	SNP (transition)	36.30%	0
Variants: VaAres1_C_FDSW210055062-1r	C	663	663	1	T -> C	629	SNP (transition)	73.40%	0
Variants: VaAres1_C_FDSW210055062-1r	G	677	677	1	A -> G	493	SNP (transition)	77.30%	0
Variants: VaAres1_C_FDSW210055062-1r	C	684	684	1	T -> C	447	SNP (transition)	71.60%	0

Variants: VaAres1_C_FDSW210055062-1r	A	687	687	1	G -> A	422	SNP (transition)	72.30%	0					
Variants: VaAres1_C_FDSW210055062-1r	T	690	690	1	A -> T	408	SNP (transversion)	77.00%	0					
Variants: VaAres1_C_FDSW210055062-1r	A	693	693	1	G -> A	389	SNP (transition)	77.60%	0					
Variants: VaAres1_C_FDSW210055062-1r	A	708	708	1	G -> A	306	SNP (transition)	78.80%	0					
Variants: VaAres1_C_FDSW210055062-1r	T	723	723	1	C -> T	234	SNP (transition)	79.90%	0					
Variants: VaAres1_C_FDSW210055062-1r	C	726	726	1	T -> C	223	SNP (transition)	78.90%	0					
Variants: VaAres1_C_FDSW210055062-1r	C	747	747	1	T -> C	143	SNP (transition)	79.00%	0.00E+00					
Variants: VaAres1_C_FDSW210055062-1r	CGTTCGA	749	755	7	TACCTTG -> CGTTCGA	121 -> 137	Substitution	41.3% -> 67.6%	5.30E-126					
Variants: VaAres1_C_FDSW210055062-1r	C	756	756	1	G -> C	125	SNP (transversion)	39.20%	5.40E-93					
Variants: VaAres1_C_FDSW210055062-1r	T	756	756	1	G -> T	125	SNP (transversion)	38.40%	1.60E-114					
Variants: VaAres1_C_FDSW210055062-1r	AGC	757	759	3	TCA -> AGC	118 -> 120	Substitution	27.1% -> 34.2%	1.00E-64					
Variants: VaAres1_C_FDSW210055062-1r	T	765	765	1	C -> T	133	SNP (transition)	32.30%	4.00E-73					
Variants: VaAres1_C_FDSW210055062-1r	A	768	768	1	T -> A	144	SNP (transversion)	60.40%	3.00E-212					
Variants: VaAres1_C_FDSW210055062-1r	T	790	790	1	G -> T	264	SNP (transversion)	26.10%	1.40E-122	R -> L	hypothetical protein CDS	hypothetica l protein	Substituti on	KLI69 879.1
Variants: VaAres1_C_FDSW210055062-1r	A	795	795	1	G -> A	292	SNP (transition)	61.00%	0	A -> T	hypothetical protein CDS	hypothetica l protein	Substituti on	KLI69 879.1
Variants: VaAres1_C_FDSW210055062-1r	AAT	801	803	2	CAC -> AAT	318 -> 336	Substitution	73.5% -> 77.7%	0	H -> N	hypothetical protein CDS	hypothetica l protein	Substituti on	KLI69 879.1
Variants: VaAres1_C_FDSW210055062-1r	C	834	834	1	T -> C	525	SNP (transition)	79.40%	0	Y -> H	hypothetical protein CDS	hypothetica l protein	Substituti on	KLI69 879.1
Variants: VaAres1_C_FDSW210055062-1r	G	888	888	1	A -> G	782	SNP (transition)	29.90%	0	I -> V	hypothetical protein CDS	hypothetica l protein	Substituti on	KLI69 879.1
Variants: VaAres1_C_FDSW210055062-1r	C	897	897	1	T -> C	796	SNP (transition)	59.20%	0	C -> R	hypothetical protein CDS	hypothetica l protein	Substituti on	KLI69 879.1
Variants: VaAres1_C_FDSW210055062-1r	C	900	900	1	G -> C	804	SNP (transversion)	59.10%	0	D -> H	hypothetical protein CDS	hypothetica l protein	Substituti on	KLI69 879.1
Variants: VaAres1_C_FDSW210055062-1r	C	942	942	1	G -> C	778	SNP (transversion)	28.00%	0	G -> R	hypothetical protein CDS	hypothetica l protein	Substituti on	KLI69 879.1
Variants: VaAres1_C_FDSW210055062-1r	TGT	966	968	2	AGC -> TGT	737 -> 738	Substitution	42.4% -> 42.5%	0	S -> C	hypothetical protein CDS	hypothetica l protein	Substituti on	KLI69 879.1

Variants: VaAres1_C_FDSW210055062-1r	G	969	969	1	C -> G	736	SNP (transversion)	28.10%	5.1E-310	P -> A	hypothetical protein CDS	hypothetical protein	Substitution	KLI69879.1
Variants: VaAres1_C_FDSW210055062-1r	A	976	976	1	T -> A	741	SNP (transversion)	28.50%	6.80E-255	V -> E	hypothetical protein CDS	hypothetical protein	Substitution	KLI69879.1
Variants: VaAres1_C_FDSW210055062-1r	A	1008	1008	1	G -> A	688	SNP (transition)	31.40%	2.40E-250	V -> I	hypothetical protein CDS	hypothetical protein	Substitution	KLI69879.1
Variants: VaAres1_C_FDSW210055062-1r	C	1035	1035	1	G -> C	729	SNP (transversion)	26.20%	2.7E-317	G -> R	hypothetical protein CDS	hypothetical protein	Substitution	KLI69879.1
Variants: VaAres1_C_FDSW210055062-1r	A	1041	1041	1	G -> A	734	SNP (transition)	32.00%	0	A -> T	hypothetical protein CDS	hypothetical protein	Substitution	KLI69879.1
Variants: VaAres1_C_FDSW210055062-1r	C	1049	1049	1	T -> C	724	SNP (transition)	25.10%	0		hypothetical protein CDS	hypothetical protein	None	KLI69879.1
Variants: VaAres1_C_FDSW210055062-1r	G	1083	1083	1	A -> G	720	SNP (transition)	33.20%	0	K -> E	hypothetical protein CDS	hypothetical protein	Substitution	KLI69879.1
Variants: VaAres1_C_FDSW210055062-1r	A	1100	1100	1	G -> A	733	SNP (transition)	34.10%	0		hypothetical protein CDS	hypothetical protein	None	KLI69879.1
Variants: VaAres1_C_FDSW210055062-1r	A	1101	1101	1	G -> A	752	SNP (transition)	30.50%	0					
Variants: VaAres1_C_FDSW210055062-1r	C	1104	1104	1	G -> C	757	SNP (transversion)	34.60%	0					
Variants: VaAres1_C_FDSW210055062-1r	T	1116	1116	1	C -> T	786	SNP (transition)	37.90%	0		hypothetical protein CDS	hypothetical protein	None	KLI69880.1
Variants: VaAres1_C_FDSW210055062-1r	T	1119	1119	1	C -> T	791	SNP (transition)	35.70%	0		hypothetical protein CDS	hypothetical protein	None	KLI69880.1
Variants: VaAres1_C_FDSW210055062-1r	G	1135	1135	1	T -> G	798	SNP (transversion)	58.10%	0	F -> V	hypothetical protein CDS	hypothetical protein	Substitution	KLI69880.1
Variants: VaAres1_C_FDSW210055062-1r	TGTG	1173	1176	3	CATA -> TGTG	736 -> 744	Substitution	39.4% -> 40.2%	0	TI -> TV	hypothetical protein CDS	hypothetical protein	Substitution	KLI69880.1
Variants: VaAres1_C_FDSW210055062-1r	C	1182	1182	1	T -> C	735	SNP (transition)	40.80%	0		hypothetical protein CDS	hypothetical protein	None	KLI69880.1
Variants: VaAres1_C_FDSW210055062-1r	G	1215	1215	1	A -> G	649	SNP (transition)	52.10%	0		hypothetical protein CDS	hypothetical protein	None	KLI69880.1
Variants: VaAres1_C_FDSW210055062-1r	G	1218	1218	1	A -> G	635	SNP (transition)	42.00%	0		hypothetical protein CDS	hypothetical protein	None	KLI69880.1
Variants: VaAres1_C_FDSW210055062-1r	G	1221	1221	1	C -> G	619	SNP (transversion)	41.50%	0		hypothetical protein CDS	hypothetical protein	None	KLI69880.1
Variants: VaAres1_C_FDSW210055062-1r	GG	1232	1233	2	AC -> GG	557 -> 562	Substitution	38.6% -> 39.0%	0	N -> R	hypothetical protein CDS	hypothetical protein	Substitution	KLI69880.1
Variants: VaAres1_C_FDSW210055062-1r	T	1235	1235	1	C -> T	548	SNP (transition)	38.90%	0	A -> V	hypothetical protein CDS	hypothetical protein	Substitution	KLI69880.1
Variants: VaAres1_C_FDSW210055062-1r	G	1269	1269	1	A -> G	336	SNP (transition)	53.90%	0	I -> M	hypothetical protein CDS	hypothetical protein	Substitution	KLI69880.1
Variants: VaAres1_C_FDSW210055062-1r	G	1275	1275	1	A -> G	310	SNP (transition)	55.80%	0		hypothetical protein CDS	hypothetical protein	None	KLI69880.1

Variants: VaAres1_C_FDSW210055062-1r	T	1287	1287	1	A -> T	236	SNP (transversion)	29.20%	1.10E-174		hypothetical protein CDS	hypothetical protein	None	KLI69880.1
Variants: VaAres1_C_FDSW210055062-1r	T	1290	1290	1	C -> T	216	SNP (transition)	26.90%	5.20E-133		hypothetical protein CDS	hypothetical protein	None	KLI69880.1
Variants: VaAres1_C_FDSW210055062-1r	G	1302	1302	1	A -> G	177	SNP (transition)	35.60%	4.20E-166		hypothetical protein CDS	hypothetical protein	None	KLI69880.1
Variants: VaAres1_C_FDSW210055062-1r	AAGTT	1324	1328	4	CACAC -> AAGTT	107 -> 117	Substitution	85.0% -> 89.7%	0.00E+00	HT -> KF	hypothetical protein CDS	hypothetical protein	Substitution	KLI69880.1
Variants: VaAres1_C_FDSW210055062-1r	AGTGCC	1330	1335	5	TTAACA -> AGTGCC	87 -> 97	Substitution	80.4% -> 84.5%	4.70E-225	LT -> SA	hypothetical protein CDS	hypothetical protein	Substitution	KLI69880.1
Variants: VaAres1_C_FDSW210055062-1r	TCGCT	1339	1343	5	CGTTA -> TCGCT	67 -> 79	Substitution	54.2% -> 60.8%	1.20E-118		hypothetical protein CDS	hypothetical protein	Extension	KLI69880.1
Variants: VaAres1_C_FDSW210055062-1r	T	1345	1345	1	G -> T	63	SNP (transversion)	55.60%	2.00E-105					
Variants: VaAres1_C_FDSW210055062-1r	TT	1347	1348	2	AA -> TT	58 -> 60	Substitution	60.3% -> 61.7%	2.80E-114					
Variants: VaAres1_C_FDSW210055062-1r	AAT	1351	1353	3	GGA -> AAT	53 -> 54	Substitution	53.7% -> 54.7%	5.30E-87					
Variants: VaAres1_C_FDSW210055062-1r	CGT	1355	1357	3	TAA -> CGT	38 -> 46	Substitution	28.9% -> 43.5%	2.40E-32					
Variants: VaAres1_C_FDSW210055062-1r	A	2106	2106	1	T -> A	32	SNP (transversion)	28.10%	1.40E-26	F -> L	hypothetical protein CDS	hypothetical protein	Substitution	KLI69881.1
Variants: VaAres1_C_FDSW210055062-1r	GC	2108	2109	2	CA -> GC	34 -> 35	Substitution	35.3% -> 37.1%	2.20E-36	A -> G	hypothetical protein CDS	hypothetical protein	Substitution	KLI69881.1
Variants: VaAres1_C_FDSW210055062-1r	AA	2111	2112	2	TC -> AA	38	Substitution	44.70%	2.30E-46	I -> K	hypothetical protein CDS	hypothetical protein	Substitution	KLI69881.1
Variants: VaAres1_C_FDSW210055062-1r	CCGAAACGGATGA	2114	2126	12	ATTTTGAAAAAAT -> CCGAAACGGATGA	37 -> 43	Substitution	48.8% -> 79.1%	2.70E-63	DFEKI -> AETDD	hypothetical protein CDS	hypothetical protein	Substitution	KLI69881.1
Variants: VaAres1_C_FDSW210055062-1r	AA	2128	2129	2	GC -> AA	49 -> 50	Substitution	83.7% -> 84.0%	1.80E-131	A -> N	hypothetical protein CDS	hypothetical protein	Substitution	KLI69881.1
Variants: VaAres1_C_FDSW210055062-1r	CAC	2131	2133	2	TAA -> CAC	53 -> 55	Substitution	84.9% -> 85.5%	2.80E-149		hypothetical protein CDS	hypothetical protein	Extension	KLI69881.1
Variants: VaAres1_C_FDSW210055062-1r	T	2135	2135	1	A -> T	56	SNP (transversion)	87.50%	9.20E-169					
Variants: VaAres1_C_FDSW210055062-1r	CTG	2137	2139	3	AGC -> CTG	60 -> 61	Substitution	90.0% -> 90.2%	5.00E-182					
Variants: VaAres1_C_FDSW210055062-1r	AG	2141	2142	2	TT -> AG	63 -> 66	Substitution	90.5% -> 90.9%	5.40E-181					
Variants: VaAres1_C_FDSW210055062-1r	G	2145	2145	1	A -> G	69	SNP (transition)	95.70%	1.30E-233					
Variants: VaAres1_C_FDSW210055062-1r	T	2147	2147	1	A -> T	72	SNP (transversion)	95.80%	1.90E-237					
Variants: VaAres1_C_FDSW210055062-1r	G	2149	2148	0	#NAME?	72	Insertion	95.80%	1.90E-237					

Variants: VaAres1_C_FDSW210055062-1r	GAG	2902	2904	3	TGA -> GAG	17 -> 19	Substitution	94.1% -> 94.7%	2.70E-44
Variants: VaAres1_C_FDSW210055062-1r	GGA	2906	2908	3	TCG -> GGA	16 -> 18	Substitution	75.0% -> 83.3%	1.80E-27
Variants: VaAres1_C_FDSW210055062-1r	GGG	2910	2912	3	AAC -> GGG	13	Substitution	53.80%	4.00E-10
Variants: VaAres1_C_FDSW210055062-1r		3097	3097	1	#NAME?	200	Deletion	45.00%	2.10E-87
Variants: VaAres1_C_FDSW210055062-1r	G	3454	3454	1	A -> G	18	SNP (transition)	33.30%	6.50E-08
Variants: VaAres1_C_FDSW210055062-1r	T	3455	3455	1	G -> T	17	SNP (transversion)	29.40%	1.90E-08
Variants: VaAres1_C_FDSW210055062-1r	G	3456	3456	1	T -> G	16	SNP (transversion)	37.50%	7.30E-09
Variants: VaAres1_C_FDSW210055062-1r	T	3659	3659	1	G -> T	250	SNP (transversion)	46.00%	0
Variants: VaAres1_C_FDSW210055062-1r	C	3758	3758	1	A -> C	212	SNP (transversion)	58.00%	0
Variants: VaAres1_C_FDSW210055062-1r	G	3778	3778	1	A -> G	222	SNP (transition)	43.70%	1.90E-275
Variants: VaAres1_C_FDSW210055062-1r	T	3780	3780	1	C -> T	218	SNP (transition)	43.60%	1.10E-269
Variants: VaAres1_C_FDSW210055062-1r	C	3852	3852	1	T -> C	209	SNP (transition)	56.90%	0
Variants: VaAres1_C_FDSW210055062-1r	G	3864	3864	1	A -> G	205	SNP (transition)	41.00%	9.80E-236
Variants: VaAres1_C_FDSW210055062-1r	T	3922	3922	1	C -> T	152	SNP (transition)	38.20%	7.60E-138
Variants: VaAres1_C_FDSW210055062-1r	A	4023	4023	1	T -> A	35	SNP (transversion)	57.10%	3.00E-37
Variants: VaAres1_C_FDSW210055062-1r	G	4023	4023	1	T -> G	35	SNP (transversion)	42.90%	8.10E-20
Variants: VaAres1_C_FDSW210055062-1r	C	4029	4029	1	T -> C	32	SNP (transition)	40.60%	1.00E-15
Variants: VaAres1_C_FDSW210055062-1r	G	4035	4035	1	C -> G	28	SNP (transversion)	35.70%	9.40E-11
Variants: VaAres1_C_FDSW210055062-1r	T	4035	4035	1	C -> T	28	SNP (transition)	64.30%	3.10E-33
Variants: VaAres1_C_FDSW210055062-1r	T	4036	4036	1	G -> T	25	SNP (transversion)	100.00%	1.00E-50
Variants: VaAres1_C_FDSW210055062-1r	T	4037	4037	1	G -> T	25	SNP (transversion)	72.00%	1.20E-34
Variants: VaAres1_C_FDSW210055062-1r		4038	4038	1	-C	24	Deletion	100.00%	2.50E-22
Variants: VaAres1_C_FDSW210055062-1r	A	4040	4040	1	T -> A	21	SNP (transversion)	100.00%	1.30E-40

Variants: VaAres1_C_FDSW210055062-1r	GTTTAA	4043	4048	6	ACCGTT -> GTTTAA	9 -> 14	Substitution	88.9% -> 92.9%	1.40E-16					
Variants: VaAres1_C_FDSW210055062-1r	GTGGTA	4050	4055	6	CGCTGC -> GTGGTA	6 -> 8	Substitution	100.00%	2.50E-13					
Variants: VaAres1_C_FDSW210055062-1r	GG	4059	4060	2	TT -> GG	3 -> 5	Substitution	80.0% -> 100.0%	1.30E-07					
Variants: VaAres1_C_FDSW210055062-1r	GGC	4062	4064	3	TCA -> GGC	3	Substitution	100.00%	1.30E-07					
Variants: VaAres1_C_FDSW210055062-1r	GGTAG	4342	4346	4	TCGAA -> GGTAG	15 -> 27	Substitution	86.7% -> 92.6%	3.20E-29	FD -> LP	hypothetical protein CDS	hypothetica l protein	Substituti on	KLI69 882.1
Variants: VaAres1_C_FDSW210055062-1r	TG	4374	4375	2	GA -> TG	319 -> 327	Substitution	99.70%	0	I -> T	hypothetical protein CDS	hypothetica l protein	Substituti on	KLI69 882.1
Variants: VaAres1_C_FDSW210055062-1r	T	4379	4379	1	C -> T	379	SNP (transition)	100.00%	0	A -> T	hypothetical protein CDS	hypothetica l protein	Substituti on	KLI69 882.1
Variants: VaAres1_C_FDSW210055062-1r	CATGA	4381	4385	5	TGGTG -> CATGA	406 -> 434	Substitution	99.50%	0		hypothetical protein CDS	hypothetica l protein	Truncatio n	KLI69 882.1
Variants: VaAres1_C_FDSW210055062-1r	GG	4388	4389	2	CA -> GG	462 -> 472	Substitution	99.80%	0	PE -> PQ	hypothetical protein CDS	hypothetica l protein	Substituti on	KLI69 882.1
Variants: VaAres1_C_FDSW210055062-1r	TGA	4392	4394	2	CGT -> TGA	503 -> 526	Substitution	100.00%	0	T -> S	hypothetical protein CDS	hypothetica l protein	Substituti on	KLI69 882.1
Variants: VaAres1_C_FDSW210055062-1r	GT	4396	4397	2	AA -> GT	545 -> 551	Substitution	99.60%	0	F -> T	hypothetical protein CDS	hypothetica l protein	Substituti on	KLI69 882.1
Variants: VaAres1_C_FDSW210055062-1r	A	4401	4401	1	C -> A	574	SNP (transversion)	100.00%	0	M -> V	hypothetical protein CDS	hypothetica l protein	Start Codon Loss	KLI69 882.1
Variants: VaAres1_C_FDSW210055062-1r	A	4404	4406	3	GTT -> A	600	Deletion	99.70%	0					
Variants: VaAres1_C_FDSW210055062-1r	GT	4409	4410	2	TC -> GT	643 -> 655	Substitution	99.20%	0					
Variants: VaAres1_C_FDSW210055062-1r	GC	4414	4415	2	CT -> GC	685 -> 696	Substitution	99.90%	0					
Variants: VaAres1_C_FDSW210055062-1r	A	4417	4417	1	G -> A	724	SNP (transition)	100.00%	0					
Variants: VaAres1_C_FDSW210055062-1r	GG	4419	4420	2	CA -> GG	755 -> 767	Substitution	99.50%	0					
Variants: VaAres1_C_FDSW210055062-1r	AC	4423	4424	2	GA -> AC	790 -> 800	Substitution	98.90%	0					
Variants: VaAres1_C_FDSW210055062-1r	G	4427	4426	0	#NAME?	826	Insertion	99.90%	0					
Variants: VaAres1_C_FDSW210055062-1r	TG	4431	4432	2	GA -> TG	869 -> 880	Substitution	99.90%	0					
Variants: VaAres1_C_FDSW210055062-1r	A	4434	4433	0	#NAME?	904	Insertion	100.00%	0					
Variants: VaAres1_C_FDSW210055062-1r	CA	4435	4436	2	GT -> CA	923 -> 933	Substitution	99.70%	0					

Variants: VaAres1_C_FDSW210055062-1r	A	4973	4973	1	G -> A	172	SNP (transition)	66.30%	0
Variants: VaAres1_C_FDSW210055062-1r	C	4975	4974	0	+C	164	Insertion	81.70%	0
Variants: VaAres1_C_FDSW210055062-1r	G	4975	4974	0	#NAME?	163	Insertion	48.50%	2.10E-229
Variants: VaAres1_C_FDSW210055062-1r	T	4975	4974	0	(T)3 -> (T)4	163	Insertion (tandem repeat)	35.00%	1.60E-138
Variants: VaAres1_C_FDSW210055062-1r	AA	4975	4976	2	TT -> AA	156 -> 159	Substitution	45.5% -> 46.5%	9.60E-204
Variants: VaAres1_C_FDSW210055062-1r	G	4978	4978	1	A -> G	147	SNP (transition)	44.90%	1.30E-195
Variants: VaAres1_C_FDSW210055062-1r	A	4979	4979	1	G -> A	138	SNP (transition)	63.80%	1.90E-279
Variants: VaAres1_C_FDSW210055062-1r	T	4980	4980	1	A -> T	136	SNP (transversion)	46.30%	3.20E-194
Variants: VaAres1_C_FDSW210055062-1r	A	4981	4981	1	G -> A	131	SNP (transition)	64.90%	5.40E-254
Variants: VaAres1_C_FDSW210055062-1r	C	4981	4981	1	G -> C	131	SNP (transversion)	32.10%	3.60E-113
Variants: VaAres1_C_FDSW210055062-1r	A	4982	4982	1	T -> A	126	SNP (transversion)	79.40%	4.9E-324
Variants: VaAres1_C_FDSW210055062-1r	A	4983	4983	1	G -> A	126	SNP (transition)	96.00%	0
Variants: VaAres1_C_FDSW210055062-1r	C	4984	4984	1	A -> C	123	SNP (transversion)	43.90%	7.50E-149
Variants: VaAres1_C_FDSW210055062-1r	A	4985	4985	1	T -> A	120	SNP (transversion)	62.50%	7.00E-215
Variants: VaAres1_C_FDSW210055062-1r	C	4985	4985	1	T -> C	120	SNP (transition)	33.30%	1.10E-112
Variants: VaAres1_C_FDSW210055062-1r	A	4986	4986	1	T -> A	117	SNP (transversion)	35.90%	1.10E-115
Variants: VaAres1_C_FDSW210055062-1r	C	4986	4986	1	T -> C	117	SNP (transition)	59.80%	1.30E-219
Variants: VaAres1_C_FDSW210055062-1r	A	4988	4988	1	G -> A	115	SNP (transition)	42.60%	2.70E-139
Variants: VaAres1_C_FDSW210055062-1r	C	4988	4988	1	G -> C	115	SNP (transversion)	52.20%	2.70E-177
Variants: VaAres1_C_FDSW210055062-1r	A	4989	4989	1	C -> A	111	SNP (transversion)	73.90%	6.60E-253
Variants: VaAres1_C_FDSW210055062-1r	AA	4992	4993	2	CG -> AA	102	Substitution	92.20%	2.2E-318
Variants: VaAres1_C_FDSW210055062-1r	A	4994	4994	1	C -> A	98	SNP (transversion)	57.10%	9.30E-169

Variants: VaAres1_C_FDSW210055062-1r	A	4995	4995	1	G -> A	95	SNP (transition)	55.80%	1.10E-153
Variants: VaAres1_C_FDSW210055062-1r	C	4995	4995	1	G -> C	95	SNP (transversion)	36.80%	3.70E-97
Variants: VaAres1_C_FDSW210055062-1r	A	4996	4996	1	T -> A	89	SNP (transversion)	39.30%	2.20E-98
Variants: VaAres1_C_FDSW210055062-1r	C	4996	4996	1	T -> C	89	SNP (transition)	52.80%	7.10E-135
Variants: VaAres1_C_FDSW210055062-1r	A	4997	4997	1	G -> A	85	SNP (transition)	51.80%	1.20E-134
Variants: VaAres1_C_FDSW210055062-1r	C	4997	4997	1	G -> C	85	SNP (transversion)	40.00%	2.40E-99
Variants: VaAres1_C_FDSW210055062-1r	A	4998	4998	1	G -> A	82	SNP (transition)	40.20%	1.40E-96
Variants: VaAres1_C_FDSW210055062-1r	T	4998	4998	1	G -> T	82	SNP (transversion)	29.30%	8.00E-62
Variants: VaAres1_C_FDSW210055062-1r	T	4999	4999	1	C -> T	81	SNP (transition)	66.70%	5.70E-163
Variants: VaAres1_C_FDSW210055062-1r	C	5000	5000	1	A -> C	79	SNP (transversion)	25.30%	2.60E-54
Variants: VaAres1_C_FDSW210055062-1r	T	5000	5000	1	A -> T	79	SNP (transversion)	63.30%	3.30E-149
Variants: VaAres1_C_FDSW210055062-1r	C	5001	5001	1	T -> C	75	SNP (transition)	38.70%	2.00E-84
Variants: VaAres1_C_FDSW210055062-1r	A	5002	5002	1	T -> A	73	SNP (transversion)	61.60%	3.80E-129
Variants: VaAres1_C_FDSW210055062-1r	A	5003	5003	1	T -> A	70	SNP (transversion)	62.90%	1.70E-122
Variants: VaAres1_C_FDSW210055062-1r	A	5004	5004	1	T -> A	65	SNP (transversion)	27.70%	1.90E-44
Variants: VaAres1_C_FDSW210055062-1r	G	5004	5004	1	T -> G	65	SNP (transversion)	35.40%	3.60E-66
Variants: VaAres1_C_FDSW210055062-1r	G	5005	5005	1	T -> G	63	SNP (transversion)	44.40%	2.50E-75
Variants: VaAres1_C_FDSW210055062-1r	G	5006	5006	1	A -> G	60	SNP (transition)	41.70%	5.10E-69
Variants: VaAres1_C_FDSW210055062-1r	T	5006	5006	1	A -> T	60	SNP (transversion)	36.70%	1.40E-61
Variants: VaAres1_C_FDSW210055062-1r	G	5007	5007	1	C -> G	57	SNP (transversion)	31.60%	1.90E-47
Variants: VaAres1_C_FDSW210055062-1r	T	5007	5007	1	C -> T	57	SNP (transition)	45.60%	1.20E-75
Variants: VaAres1_C_FDSW210055062-1r	C	5009	5009	1	A -> C	49	SNP (transversion)	36.70%	1.10E-50

Variants: VaAres1_C_FDSW210055062-1r	T	5009	5009	1	A -> T	49	SNP (transversion)	36.70%	7.20E-49
Variants: VaAres1_C_FDSW210055062-1r	C	5010	5010	1	T -> C	51	SNP (transition)	35.30%	2.80E-50
Variants: VaAres1_C_FDSW210055062-1r	T	5011	5011	1	G -> T	47	SNP (transversion)	68.10%	1.20E-97
Variants: VaAres1_C_FDSW210055062-1r	G	5012	5012	1	C -> G	37	SNP (transversion)	40.50%	2.90E-40
Variants: VaAres1_C_FDSW210055062-1r	C	5014	5014	1	T -> C	29	SNP (transition)	48.30%	7.70E-42
Variants: VaAres1_C_FDSW210055062-1r	TT	5016	5017	2	GG -> TT	27 -> 28	Substitution	37.0% -> 39.3%	1.70E-27
Variants: VaAres1_C_FDSW210055062-1r	G	5020	5020	1	T -> G	26	SNP (transversion)	34.60%	1.60E-27
Variants: VaAres1_C_FDSW210055062-1r	T	5022	5022	1	A -> T	23	SNP (transversion)	26.10%	6.40E-18
Variants: VaAres1_C_FDSW210055062-1r	C	5579	5579	1	G -> C	50	SNP (transversion)	98.00%	1.00E-160
Variants: VaAres1_C_FDSW210055062-1r	T	5581	5581	1	A -> T	49	SNP (transversion)	100.00%	1.60E-157
Variants: VaAres1_C_FDSW210055062-1r	CG	5583	5584	2	TA -> CG	48	Substitution	100.00%	4.00E-159
Variants: VaAres1_C_FDSW210055062-1r	GTC	5605	5607	3	TAA -> GTC	46 -> 47	Substitution	27.7% -> 30.4%	4.40E-35
Variants: VaAres1_C_FDSW210055062-1r	TT	5609	5610	2	AA -> TT	44	Substitution	31.8% -> 36.4%	1.10E-31
Variants: VaAres1_C_FDSW210055062-1r	AA	5612	5613	2	GC -> AA	47 -> 49	Substitution	42.6% -> 44.9%	9.60E-52
Variants: VaAres1_C_FDSW210055062-1r	GGG	5615	5617	3	TTA -> GGG	51 -> 53	Substitution	48.1% -> 52.9%	4.90E-77
Variants: VaAres1_C_FDSW210055062-1r	A	5618	5618	1	G -> A	48	SNP (transition)	62.50%	7.30E-90
Variants: VaAres1_C_FDSW210055062-1r	T	5618	5618	1	G -> T	48	SNP (transversion)	37.50%	4.50E-40
Variants: VaAres1_C_FDSW210055062-1r	GT	5619	5620	2	TC -> GT	45 -> 48	Substitution	100.00%	1.00E-144
Variants: VaAres1_C_FDSW210055062-1r	C	5621	5621	1	G -> C	47	SNP (transversion)	66.00%	4.70E-97
Variants: VaAres1_C_FDSW210055062-1r	T	5622	5622	1	C -> T	48	SNP (transition)	97.90%	1.90E-149
Variants: VaAres1_C_FDSW210055062-1r	G	5623	5623	1	C -> G	50	SNP (transversion)	70.00%	2.20E-107
Variants: VaAres1_C_FDSW210055062-1r	AA	5624	5625	2	TT -> AA	50 -> 51	Substitution	98.00%	6.30E-151

Variants: VaAres1_C_FDSW210055062-1r	A	5626	5626	1	G -> A	52	SNP (transition)	71.20%	1.40E-117
Variants: VaAres1_C_FDSW210055062-1r	T	5626	5626	1	G -> T	52	SNP (transversion)	26.90%	6.40E-25
Variants: VaAres1_C_FDSW210055062-1r	T	5632	5632	1	A -> T	63	SNP (transversion)	93.70%	2.30E-148
Variants: VaAres1_C_FDSW210055062-1r	TATG	5633	5636	4	ATCA -> TATG	57 -> 63	Substitution	63.5% -> 70.2%	8.10E-99
Variants: VaAres1_C_FDSW210055062-1r	A	5637	5637	1	T -> A	58	SNP (transversion)	89.70%	6.40E-144
Variants: VaAres1_C_FDSW210055062-1r	A	5638	5638	1	T -> A	59	SNP (transversion)	71.20%	1.70E-116
Variants: VaAres1_C_FDSW210055062-1r	AG	5645	5646	2	TC -> AG	73	Substitution	79.50%	3.60E-171
Variants: VaAres1_C_FDSW210055062-1r		5648	5648	1	(C)3 -> (C)2	83	Deletion (tandem repeat)	68.70%	3.40E-65
Variants: VaAres1_C_FDSW210055062-1r	T	5653	5653	1	G -> T	112	SNP (transversion)	59.80%	1.10E-156
Variants: VaAres1_C_FDSW210055062-1r	GC	5658	5657	0	#NAME?	136	Insertion	48.50%	5.20E-159
Variants: VaphiVaphiSt2_B_A_FDSW210055054-2r	TGGT	79	82	4	CCTG -> TGGT	102 -> 111	Substitution	82.9% -> 84.3%	1.90E-249
Variants: VaphiVaphiSt2_B_A_FDSW210055054-2r	G	84	84	1	A -> G	87	SNP (transition)	95.40%	1.10E-251
Variants: VaphiVaphiSt2_B_A_FDSW210055054-2r	GGGG	86	89	4	CATC -> GGGG	73 -> 79	Substitution	85.3% -> 86.3%	1.70E-193
Variants: VaphiVaphiSt2_B_A_FDSW210055054-2r	C	91	91	1	T -> C	70	SNP (transition)	92.90%	3.80E-182
Variants: VaphiVaphiSt2_B_A_FDSW210055054-2r	GGA	93	95	3	CAC -> GGA	64 -> 69	Substitution	76.6% -> 81.2%	4.70E-127
Variants: VaphiVaphiSt2_B_A_FDSW210055054-2r	T	97	97	1	A -> T	66	SNP (transversion)	83.30%	1.10E-142
Variants: VaphiVaphiSt2_B_A_FDSW210055054-2r	GAA	99	101	3	CGT -> GAA	56 -> 57	Substitution	64.9% -> 69.6%	9.30E-84
Variants: VaphiVaphiSt2_B_A_FDSW210055054-2r	C	102	102	1	T -> C	58	SNP (transition)	75.90%	9.70E-98
Variants: VaphiVaphiSt2_B_A_FDSW210055054-2r	C	103	102	0	+C	57	Insertion	36.80%	6.00E-38
Variants: VaphiVaphiSt2_B_A_FDSW210055054-2r	A	104	104	1	C -> A	71	SNP (transversion)	29.60%	2.20E-31
Variants: VaphiVaphiSt2_B_A_FDSW210055054-2r	C	108	108	1	A -> C	79	SNP (transversion)	38.00%	3.40E-39
Variants: VaphiVaphiSt2_B_A_FDSW210055054-2r	G	120	120	1	A -> G	111	SNP (transition)	33.30%	1.10E-34

Variants: VaphiVaphiSt2_B_A_FDSW21 0055054-2r	T	126	126	1	C -> T	132	SNP (transition)	43.90%	4.70E-45
Variants: VaphiVaphiSt2_B_A_FDSW21 0055054-2r	T	129	129	1	C -> T	139	SNP (transition)	76.30%	3.20E-149
Variants: VaphiVaphiSt2_B_A_FDSW21 0055054-2r	G	132	132	1	C -> G	148	SNP (transversion)	37.20%	1.20E-91
Variants: VaphiVaphiSt2_B_A_FDSW21 0055054-2r	T	144	144	1	C -> T	187	SNP (transition)	46.50%	3.70E-86
Variants: VaphiVaphiSt2_B_A_FDSW21 0055054-2r	G	150	150	1	C -> G	209	SNP (transversion)	35.40%	2.60E-84
Variants: VaphiVaphiSt2_B_A_FDSW21 0055054-2r	A	159	159	1	G -> A	231	SNP (transition)	81.00%	0
Variants: VaphiVaphiSt2_B_A_FDSW21 0055054-2r	A	165	165	1	T -> A	248	SNP (transversion)	37.10%	6.50E-143
Variants: VaphiVaphiSt2_B_A_FDSW21 0055054-2r	A	183	183	1	G -> A	319	SNP (transition)	27.90%	5.20E-134
Variants: VaphiVaphiSt2_B_A_FDSW21 0055054-2r	A	192	192	1	G -> A	356	SNP (transition)	34.60%	1.60E-271
Variants: VaphiVaphiSt2_B_A_FDSW21 0055054-2r	G	216	216	1	A -> G	419	SNP (transition)	27.00%	2.10E-246
Variants: VaphiVaphiSt2_B_A_FDSW21 0055054-2r	T	225	225	1	A -> T	438	SNP (transversion)	47.70%	7.60E-269
Variants: VaphiVaphiSt2_B_A_FDSW21 0055054-2r	C	237	237	1	T -> C	457	SNP (transition)	33.00%	1.50E-135
Variants: VaphiVaphiSt2_B_A_FDSW21 0055054-2r	C	240	240	1	T -> C	463	SNP (transition)	41.70%	8.50E-272
Variants: VaphiVaphiSt2_B_A_FDSW21 0055054-2r	A	294	294	1	G -> A	422	SNP (transition)	42.70%	0
Variants: VaphiVaphiSt2_B_A_FDSW21 0055054-2r	A	303	303	1	T -> A	425	SNP (transversion)	30.10%	2.70E-222
Variants: VaphiVaphiSt2_B_A_FDSW21 0055054-2r	G	306	306	1	A -> G	434	SNP (transition)	29.50%	6.40E-221
Variants: VaphiVaphiSt2_B_A_FDSW21 0055054-2r	A	315	315	1	G -> A	449	SNP (transition)	25.40%	2.30E-188
Variants: VaphiVaphiSt2_B_A_FDSW21 0055054-2r	A	357	357	1	G -> A	442	SNP (transition)	41.20%	0
Variants: VaphiVaphiSt2_B_A_FDSW21 0055054-2r	G	363	363	1	A -> G	450	SNP (transition)	45.30%	0
Variants: VaphiVaphiSt2_B_A_FDSW21 0055054-2r	C	369	369	1	T -> C	461	SNP (transition)	93.10%	0
Variants: VaphiVaphiSt2_B_A_FDSW21 0055054-2r	A	371	371	1	G -> A	459	SNP (transition)	59.00%	0
Variants: VaphiVaphiSt2_B_A_FDSW21 0055054-2r	G	387	387	1	C -> G	517	SNP (transversion)	66.00%	0

Variants: VaphiVaphiSt2_B_A_FDSW210055054-2r	A	393	393	1	G -> A	524	SNP (transition)	30.70%	8.2E-313
Variants: VaphiVaphiSt2_B_A_FDSW210055054-2r	C	393	393	1	G -> C	524	SNP (transversion)	54.20%	0
Variants: VaphiVaphiSt2_B_A_FDSW210055054-2r	C	396	396	1	A -> C	531	SNP (transversion)	38.40%	0
Variants: VaphiVaphiSt2_B_A_FDSW210055054-2r	G	420	420	1	A -> G	586	SNP (transition)	34.60%	0
Variants: VaphiVaphiSt2_B_A_FDSW210055054-2r	T	420	420	1	A -> T	586	SNP (transversion)	41.30%	0
Variants: VaphiVaphiSt2_B_A_FDSW210055054-2r	A	422	422	1	T -> A	584	SNP (transversion)	40.80%	0
Variants: VaphiVaphiSt2_B_A_FDSW210055054-2r	A	423	423	1	G -> A	586	SNP (transition)	75.10%	0
Variants: VaphiVaphiSt2_B_A_FDSW210055054-2r	A	426	426	1	G -> A	592	SNP (transition)	74.30%	0
Variants: VaphiVaphiSt2_B_A_FDSW210055054-2r	G	438	438	1	A -> G	607	SNP (transition)	59.10%	0
Variants: VaphiVaphiSt2_B_A_FDSW210055054-2r	A	456	456	1	G -> A	598	SNP (transition)	55.20%	0
Variants: VaphiVaphiSt2_B_A_FDSW210055054-2r	C	462	462	1	T -> C	595	SNP (transition)	27.10%	1.90E-238
Variants: VaphiVaphiSt2_B_A_FDSW210055054-2r	G	469	469	1	C -> G	602	SNP (transversion)	31.90%	0
Variants: VaphiVaphiSt2_B_A_FDSW210055054-2r	C	489	489	1	T -> C	644	SNP (transition)	48.90%	0
Variants: VaphiVaphiSt2_B_A_FDSW210055054-2r	C	498	498	1	G -> C	635	SNP (transversion)	32.10%	0
Variants: VaphiVaphiSt2_B_A_FDSW210055054-2r	C	513	513	1	T -> C	637	SNP (transition)	53.20%	0
Variants: VaphiVaphiSt2_B_A_FDSW210055054-2r	C	516	516	1	T -> C	631	SNP (transition)	36.10%	0
Variants: VaphiVaphiSt2_B_A_FDSW210055054-2r	T	531	531	1	C -> T	627	SNP (transition)	27.00%	0
Variants: VaphiVaphiSt2_B_A_FDSW210055054-2r	C	537	537	1	G -> C	615	SNP (transversion)	56.30%	0
Variants: VaphiVaphiSt2_B_A_FDSW210055054-2r	G	540	540	1	A -> G	614	SNP (transition)	37.60%	0
Variants: VaphiVaphiSt2_B_A_FDSW210055054-2r	A	552	552	1	T -> A	594	SNP (transversion)	32.50%	0
Variants: VaphiVaphiSt2_B_A_FDSW210055054-2r	A	564	564	1	C -> A	589	SNP (transversion)	27.80%	1.00E-277
Variants: VaphiVaphiSt2_B_A_FDSW210055054-2r	G	588	588	1	A -> G	580	SNP (transition)	32.60%	0

Variants: VaphiVaphiSt2_B_A_FDSW210055054-2r	GAG	658	660	3	CTT -> GAG	329 -> 338	Substitution	37.6% -> 38.3%	1.40E-291					
Variants: VaphiVaphiSt2_B_A_FDSW210055054-2r	C	663	663	1	T -> C	321	SNP (transition)	47.70%	0					
Variants: VaphiVaphiSt2_B_A_FDSW210055054-2r	G	677	677	1	A -> G	265	SNP (transition)	44.20%	4.50E-239					
Variants: VaphiVaphiSt2_B_A_FDSW210055054-2r	C	684	684	1	T -> C	239	SNP (transition)	33.90%	2.30E-154					
Variants: VaphiVaphiSt2_B_A_FDSW210055054-2r	A	687	687	1	G -> A	225	SNP (transition)	32.90%	6.10E-118					
Variants: VaphiVaphiSt2_B_A_FDSW210055054-2r	T	690	690	1	A -> T	214	SNP (transversion)	36.90%	1.90E-138					
Variants: VaphiVaphiSt2_B_A_FDSW210055054-2r	A	693	693	1	G -> A	206	SNP (transition)	35.00%	7.90E-103					
Variants: VaphiVaphiSt2_B_A_FDSW210055054-2r	A	708	708	1	G -> A	165	SNP (transition)	28.50%	1.40E-53					
Variants: VaphiVaphiSt2_B_A_FDSW210055054-2r	T	723	723	1	C -> T	125	SNP (transition)	36.00%	1.30E-65					
Variants: VaphiVaphiSt2_B_A_FDSW210055054-2r	C	726	726	1	T -> C	109	SNP (transition)	33.90%	9.60E-57					
Variants: VaphiVaphiSt2_B_A_FDSW210055054-2r	GAC	744	746	3	AGA -> GAC	34 -> 37	Substitution	29.7% -> 44.4%	2.70E-30					
Variants: VaphiVaphiSt2_B_A_FDSW210055054-2r	C	747	747	1	T -> C	32	SNP (transition)	37.50%	8.70E-25					
Variants: VaphiVaphiSt2_B_A_FDSW210055054-2r	C	749	749	1	T -> C	30	SNP (transition)	30.00%	8.80E-19					
Variants: VaphiVaphiSt2_B_A_FDSW210055054-2r	C	750	750	1	A -> C	30	SNP (transversion)	36.70%	2.20E-30					
Variants: VaphiVaphiSt2_B_A_FDSW210055054-2r	G	750	750	1	A -> G	30	SNP (transition)	30.00%	3.30E-15					
Variants: VaphiVaphiSt2_B_A_FDSW210055054-2r	T	751	751	1	C -> T	32	SNP (transition)	25.00%	9.80E-14					
Variants: VaphiVaphiSt2_B_A_FDSW210055054-2r	C	756	756	1	G -> C	35	SNP (transversion)	54.30%	6.40E-52					
Variants: VaphiVaphiSt2_B_A_FDSW210055054-2r	A	768	768	1	T -> A	52	SNP (transversion)	57.70%	2.70E-82					
Variants: VaphiVaphiSt2_B_A_FDSW210055054-2r	T	790	790	1	G -> T	120	SNP (transversion)	26.70%	8.10E-71	R -> L	hypothetical protein CDS	hypothetical protein	Substitution	KLI69879.1
Variants: VaphiVaphiSt2_B_A_FDSW210055054-2r	A	795	795	1	G -> A	140	SNP (transition)	57.90%	2.00E-195	A -> T	hypothetical protein CDS	hypothetical protein	Substitution	KLI69879.1
Variants: VaphiVaphiSt2_B_A_FDSW210055054-2r	AAT	801	803	2	CAC -> AAT	154 -> 159	Substitution	76.6% -> 76.7%	6.00E-273	H -> N	hypothetical protein CDS	hypothetical protein	Substitution	KLI69879.1
Variants: VaphiVaphiSt2_B_A_FDSW210055054-2r	C	834	834	1	T -> C	233	SNP (transition)	80.30%	0	Y -> H	hypothetical protein CDS	hypothetical protein	Substitution	KLI69879.1

Variants: VaphiVaphiSt2_B_A_FDSW210055054-2r	G	888	888	1	A -> G	361	SNP (transition)	33.20%	1.70E-262	I -> V	hypothetical protein CDS	hypothetical protein	Substitution	KLI69879.1
Variants: VaphiVaphiSt2_B_A_FDSW210055054-2r	C	897	897	1	T -> C	357	SNP (transition)	61.30%	0	C -> R	hypothetical protein CDS	hypothetical protein	Substitution	KLI69879.1
Variants: VaphiVaphiSt2_B_A_FDSW210055054-2r	C	900	900	1	G -> C	358	SNP (transversion)	61.50%	0	D -> H	hypothetical protein CDS	hypothetical protein	Substitution	KLI69879.1
Variants: VaphiVaphiSt2_B_A_FDSW210055054-2r	T	927	927	1	C -> T	366	SNP (transition)	26.20%	1.40E-179		hypothetical protein CDS	hypothetical protein	Truncation	KLI69879.1
Variants: VaphiVaphiSt2_B_A_FDSW210055054-2r	C	942	942	1	G -> C	354	SNP (transversion)	34.70%	4.40E-235	G -> R	hypothetical protein CDS	hypothetical protein	Substitution	KLI69879.1
Variants: VaphiVaphiSt2_B_A_FDSW210055054-2r	TGT	966	968	2	AGC -> TGT	334 -> 338	Substitution	45.0% -> 45.8%	1.50E-269	S -> C	hypothetical protein CDS	hypothetical protein	Substitution	KLI69879.1
Variants: VaphiVaphiSt2_B_A_FDSW210055054-2r	G	969	969	1	C -> G	333	SNP (transversion)	29.10%	5.30E-148	P -> A	hypothetical protein CDS	hypothetical protein	Substitution	KLI69879.1
Variants: VaphiVaphiSt2_B_A_FDSW210055054-2r	A	976	976	1	T -> A	340	SNP (transversion)	27.90%	3.10E-124	V -> E	hypothetical protein CDS	hypothetical protein	Substitution	KLI69879.1
Variants: VaphiVaphiSt2_B_A_FDSW210055054-2r	A	1008	1008	1	G -> A	304	SNP (transition)	31.30%	3.00E-120	V -> I	hypothetical protein CDS	hypothetical protein	Substitution	KLI69879.1
Variants: VaphiVaphiSt2_B_A_FDSW210055054-2r	C	1035	1035	1	G -> C	329	SNP (transversion)	25.80%	4.20E-150	G -> R	hypothetical protein CDS	hypothetical protein	Substitution	KLI69879.1
Variants: VaphiVaphiSt2_B_A_FDSW210055054-2r	A	1041	1041	1	G -> A	338	SNP (transition)	32.20%	2.00E-193	A -> T	hypothetical protein CDS	hypothetical protein	Substitution	KLI69879.1
Variants: VaphiVaphiSt2_B_A_FDSW210055054-2r	C	1049	1049	1	T -> C	355	SNP (transition)	26.20%	5.90E-174		hypothetical protein CDS	hypothetical protein	None	KLI69879.1
Variants: VaphiVaphiSt2_B_A_FDSW210055054-2r	G	1083	1083	1	A -> G	368	SNP (transition)	33.20%	7.70E-279	K -> E	hypothetical protein CDS	hypothetical protein	Substitution	KLI69879.1
Variants: VaphiVaphiSt2_B_A_FDSW210055054-2r	A	1100	1100	1	G -> A	371	SNP (transition)	33.70%	3.40E-299		hypothetical protein CDS	hypothetical protein	None	KLI69879.1
Variants: VaphiVaphiSt2_B_A_FDSW210055054-2r	A	1101	1101	1	G -> A	370	SNP (transition)	32.70%	7.20E-300					
Variants: VaphiVaphiSt2_B_A_FDSW210055054-2r	C	1104	1104	1	G -> C	368	SNP (transversion)	35.30%	0					
Variants: VaphiVaphiSt2_B_A_FDSW210055054-2r	T	1116	1116	1	C -> T	377	SNP (transition)	37.90%	0		hypothetical protein CDS	hypothetical protein	None	KLI69880.1
Variants: VaphiVaphiSt2_B_A_FDSW210055054-2r	T	1119	1119	1	C -> T	378	SNP (transition)	36.50%	0		hypothetical protein CDS	hypothetical protein	None	KLI69880.1
Variants: VaphiVaphiSt2_B_A_FDSW210055054-2r	G	1135	1135	1	T -> G	392	SNP (transversion)	59.20%	0	F -> V	hypothetical protein CDS	hypothetical protein	Substitution	KLI69880.1
Variants: VaphiVaphiSt2_B_A_FDSW210055054-2r	TGTG	1173	1176	3	CATA -> TGTG	361 -> 364	Substitution	37.6% -> 38.6%	0	TI -> TV	hypothetical protein CDS	hypothetical protein	Substitution	KLI69880.1
Variants: VaphiVaphiSt2_B_A_FDSW210055054-2r	C	1182	1182	1	T -> C	359	SNP (transition)	37.90%	0		hypothetical protein CDS	hypothetical protein	None	KLI69880.1
Variants: VaphiVaphiSt2_B_A_FDSW210055054-2r	G	1215	1215	1	A -> G	298	SNP (transition)	50.00%	0		hypothetical protein CDS	hypothetical protein	None	KLI69880.1

Variants: VaphiVaphiSt2_B_A_FDSW210055054-2r	G	1218	1218	1	A -> G	292	SNP (transition)	40.80%	0		hypothetical protein CDS	hypothetical protein	None	KLI69880.1
Variants: VaphiVaphiSt2_B_A_FDSW210055054-2r	G	1221	1221	1	C -> G	287	SNP (transversion)	40.10%	1.2E-320		hypothetical protein CDS	hypothetical protein	None	KLI69880.1
Variants: VaphiVaphiSt2_B_A_FDSW210055054-2r	GG	1232	1233	2	AC -> GG	250 -> 255	Substitution	36.9% -> 37.6%	6.10E-263	N -> R	hypothetical protein CDS	hypothetical protein	Substitution	KLI69880.1
Variants: VaphiVaphiSt2_B_A_FDSW210055054-2r	T	1235	1235	1	C -> T	245	SNP (transition)	37.60%	1.30E-253	A -> V	hypothetical protein CDS	hypothetical protein	Substitution	KLI69880.1
Variants: VaphiVaphiSt2_B_A_FDSW210055054-2r	G	1269	1269	1	A -> G	160	SNP (transition)	46.90%	2.00E-201	I -> M	hypothetical protein CDS	hypothetical protein	Substitution	KLI69880.1
Variants: VaphiVaphiSt2_B_A_FDSW210055054-2r	G	1275	1275	1	A -> G	142	SNP (transition)	47.20%	4.60E-187		hypothetical protein CDS	hypothetical protein	None	KLI69880.1
Variants: VaphiVaphiSt2_B_A_FDSW210055054-2r	G	1302	1302	1	A -> G	84	SNP (transition)	29.80%	4.80E-67		hypothetical protein CDS	hypothetical protein	None	KLI69880.1
Variants: VaphiVaphiSt2_B_A_FDSW210055054-2r	AAGTT	1324	1328	4	CACAC -> AAGTT	54 -> 58	Substitution	83.3% -> 86.2%	1.00E-142	HT -> KF	hypothetical protein CDS	hypothetical protein	Substitution	KLI69880.1
Variants: VaphiVaphiSt2_B_A_FDSW210055054-2r	AGTGCC	1330	1335	5	TTAACA -> AGTGCC	48 -> 51	Substitution	77.1% -> 84.3%	7.10E-120	LT -> SA	hypothetical protein CDS	hypothetical protein	Substitution	KLI69880.1
Variants: VaphiVaphiSt2_B_A_FDSW210055054-2r	TCGCT	1339	1343	5	CGTTA -> TCGCT	36 -> 45	Substitution	38.6% -> 45.5%	5.50E-45		hypothetical protein CDS	hypothetical protein	Extension	KLI69880.1
Variants: VaphiVaphiSt2_B_A_FDSW210055054-2r	T	1345	1345	1	G -> T	34	SNP (transversion)	41.20%	5.50E-42					
Variants: VaphiVaphiSt2_B_A_FDSW210055054-2r	TT	1347	1348	2	AA -> TT	38 -> 39	Substitution	39.5% -> 41.0%	1.50E-41					
Variants: VaphiVaphiSt2_B_A_FDSW210055054-2r	AAT	1351	1353	3	GGA -> AAT	37 -> 40	Substitution	29.7% -> 35.0%	1.70E-32					
Variants: VaphiVaphiSt2_B_A_FDSW210055054-2r	AA	2117	2118	2	TT -> AA	32	Substitution	25.00%	4.10E-20		hypothetical protein CDS	hypothetical protein	Truncation	KLI69881.1
Variants: VaphiVaphiSt2_B_A_FDSW210055054-2r	G	2121	2121	1	A -> G	33	SNP (transition)	30.30%	9.20E-28		hypothetical protein CDS	hypothetical protein	None	KLI69881.1
Variants: VaphiVaphiSt2_B_A_FDSW210055054-2r	GATGA	2122	2126	4	AAAAT -> GATGA	35 -> 38	Substitution	25.7% -> 31.6%	3.50E-26	KI -> DD	hypothetical protein CDS	hypothetical protein	Substitution	KLI69881.1
Variants: VaphiVaphiSt2_B_A_FDSW210055054-2r	AA	2128	2129	2	GC -> AA	36 -> 37	Substitution	36.1% -> 37.8%	4.30E-37	A -> N	hypothetical protein CDS	hypothetical protein	Substitution	KLI69881.1
Variants: VaphiVaphiSt2_B_A_FDSW210055054-2r	CAC	2131	2133	2	TAA -> CAC	33 -> 36	Substitution	38.9% -> 42.4%	2.00E-42		hypothetical protein CDS	hypothetical protein	Extension	KLI69881.1
Variants: VaphiVaphiSt2_B_A_FDSW210055054-2r	T	2135	2135	1	A -> T	35	SNP (transversion)	42.90%	3.20E-42					
Variants: VaphiVaphiSt2_B_A_FDSW210055054-2r	CTG	2137	2139	3	AGC -> CTG	40	Substitution	45.00%	4.50E-49					
Variants: VaphiVaphiSt2_B_A_FDSW210055054-2r	AG	2141	2142	2	TT -> AG	39 -> 40	Substitution	45.0% -> 46.2%	1.10E-50					
Variants: VaphiVaphiSt2_B_A_FDSW210055054-2r	G	2144	2144	1	A -> G	39	SNP (transition)	25.60%	6.30E-25					

Variants: VaphiVaphiSt2_B_A_FDSW210055054-2r	G	2145	2145	1	A -> G	39	SNP (transition)	71.80%	1.10E-86
Variants: VaphiVaphiSt2_B_A_FDSW210055054-2r	T	2146	2146	1	G -> T	36	SNP (transversion)	27.80%	2.50E-26
Variants: VaphiVaphiSt2_B_A_FDSW210055054-2r	G	2147	2147	1	A -> G	37	SNP (transition)	29.70%	3.40E-29
Variants: VaphiVaphiSt2_B_A_FDSW210055054-2r	T	2147	2147	1	A -> T	37	SNP (transversion)	48.60%	1.10E-51
Variants: VaphiVaphiSt2_B_A_FDSW210055054-2r	G	2149	2148	0	#NAME?	37	Insertion	48.60%	7.00E-50
Variants: VaphiVaphiSt2_B_A_FDSW210055054-2r	GAG	2902	2904	3	TGA -> GAG	16 -> 17	Substitution	62.5% -> 64.7%	7.60E-17
Variants: VaphiVaphiSt2_B_A_FDSW210055054-2r	GG	2906	2907	2	TC -> GG	14	Substitution	50.0% -> 57.1%	1.90E-08
Variants: VaphiVaphiSt2_B_A_FDSW210055054-2r		3097	3097	1	#NAME?	181	Deletion	40.30%	6.50E-67
Variants: VaphiVaphiSt2_B_A_FDSW210055054-2r	T	3659	3659	1	G -> T	181	SNP (transversion)	43.60%	1.60E-232
Variants: VaphiVaphiSt2_B_A_FDSW210055054-2r	C	3758	3758	1	A -> C	168	SNP (transversion)	57.10%	1.00E-297
Variants: VaphiVaphiSt2_B_A_FDSW210055054-2r	G	3778	3778	1	A -> G	174	SNP (transition)	38.50%	4.40E-186
Variants: VaphiVaphiSt2_B_A_FDSW210055054-2r	T	3780	3780	1	C -> T	173	SNP (transition)	39.90%	8.10E-200
Variants: VaphiVaphiSt2_B_A_FDSW210055054-2r	C	3852	3852	1	T -> C	164	SNP (transition)	56.10%	4.20E-275
Variants: VaphiVaphiSt2_B_A_FDSW210055054-2r	G	3864	3864	1	A -> G	163	SNP (transition)	41.10%	1.70E-188
Variants: VaphiVaphiSt2_B_A_FDSW210055054-2r	T	3922	3922	1	C -> T	117	SNP (transition)	30.80%	7.10E-93
Variants: VaphiVaphiSt2_B_A_FDSW210055054-2r	T	4022	4022	1	G -> T	24	SNP (transversion)	100.00%	1.00E-72
Variants: VaphiVaphiSt2_B_A_FDSW210055054-2r	A	4023	4023	1	T -> A	24	SNP (transversion)	83.30%	1.10E-62
Variants: VaphiVaphiSt2_B_A_FDSW210055054-2r	T	4025	4025	1	C -> T	24	SNP (transition)	100.00%	1.60E-77
Variants: VaphiVaphiSt2_B_A_FDSW210055054-2r	T	4027	4027	1	A -> T	24	SNP (transversion)	100.00%	4.00E-75
Variants: VaphiVaphiSt2_B_A_FDSW210055054-2r	TTG	4031	4033	3	CCA -> TTG	20 -> 22	Substitution	100.00%	1.00E-64
Variants: VaphiVaphiSt2_B_A_FDSW210055054-2r	TTT	4035	4038	4	CGGC -> TTT	15 -> 19	Deletion	89.5% -> 94.1%	3.20E-23
Variants: VaphiVaphiSt2_B_A_FDSW210055054-2r	A	4040	4040	1	T -> A	14	SNP (transversion)	100.00%	4.00E-44

Variants: VaphiVaphiSt2_B_A_FDSW210055054-2r	GTTTAA	4043	4048	6	ACCGTT -> GTTTAA	4 -> 10	Substitution	100.00%	2.50E-12					
Variants: VaphiVaphiSt2_B_A_FDSW210055054-2r	GTGG	4050	4053	4	CGCT -> GTGG	3	Substitution	100.00%	7.90E-09					
Variants: VaphiVaphiSt2_B_A_FDSW210055054-2r	AG	4336	4337	2	TC -> AG	12 -> 15	Substitution	100.00%	4.00E-09	D -> L	hypothetical protein CDS	hypothetical protein	Substitution	KLI69882.1
Variants: VaphiVaphiSt2_B_A_FDSW210055054-2r	AGGTA	4339	4343	4	TTATC -> AGGTA	17 -> 21	Substitution	94.1% -> 95.2%	4.00E-17	DK -> YL	hypothetical protein CDS	hypothetical protein	Substitution	KLI69882.1
Variants: VaphiVaphiSt2_B_A_FDSW210055054-2r		4345	4346	2	#NAME?	22	Deletion	100.00%	6.30E-14		hypothetical protein CDS	hypothetical protein	Frame Shift	KLI69882.1
Variants: VaphiVaphiSt2_B_A_FDSW210055054-2r	T	4349	4349	1	C -> T	27	SNP (transition)	100.00%	3.20E-41	E -> K	hypothetical protein CDS	hypothetical protein	Substitution	KLI69882.1
Variants: VaphiVaphiSt2_B_A_FDSW210055054-2r	ACCA	4369	4372	4	TGGC -> ACCA	123 -> 146	Substitution	100.00%	0	GQ -> VV	hypothetical protein CDS	hypothetical protein	Substitution	KLI69882.1
Variants: VaphiVaphiSt2_B_A_FDSW210055054-2r	TT	4374	4375	2	GA -> TT	159 -> 168	Substitution	100.00%	0	I -> K	hypothetical protein CDS	hypothetical protein	Substitution	KLI69882.1
Variants: VaphiVaphiSt2_B_A_FDSW210055054-2r	TGGTTCATGA	4378	4387	9	GCTTGGTGCT -> TGGTTCATGA	189 -> 238	Substitution	91.2% -> 99.5%	0	EHQA -> VMNH	hypothetical protein CDS	hypothetical protein	Substitution	KLI69882.1
Variants: VaphiVaphiSt2_B_A_FDSW210055054-2r	T	4389	4389	1	A -> T	243	SNP (transversion)	100.00%	0		hypothetical protein CDS	hypothetical protein	None	KLI69882.1
Variants: VaphiVaphiSt2_B_A_FDSW210055054-2r	G	4392	4392	1	C -> G	258	SNP (transversion)	99.60%	0		hypothetical protein CDS	hypothetical protein	None	KLI69882.1
Variants: VaphiVaphiSt2_B_A_FDSW210055054-2r	G	4395	4395	1	A -> G	272	SNP (transition)	99.60%	0		hypothetical protein CDS	hypothetical protein	None	KLI69882.1
Variants: VaphiVaphiSt2_B_A_FDSW210055054-2r	GTCGTA	4398	4403	6	CGTCAC -> GTCGTA	291 -> 322	Substitution	99.3% -> 99.4%	0	MT -> YD	hypothetical protein CDS	hypothetical protein	Start Codon Loss	KLI69882.1
Variants: VaphiVaphiSt2_B_A_FDSW210055054-2r	ACA	4404	4406	3	GTT -> ACA	329 -> 341	Substitution	99.70%	0					
Variants: VaphiVaphiSt2_B_A_FDSW210055054-2r	GT	4409	4410	2	TC -> GT	356 -> 359	Substitution	100.00%	0					
Variants: VaphiVaphiSt2_B_A_FDSW210055054-2r	GC	4414	4415	2	CT -> GC	384 -> 388	Substitution	100.00%	0					
Variants: VaphiVaphiSt2_B_A_FDSW210055054-2r	A	4417	4417	1	G -> A	400	SNP (transition)	99.50%	0					
Variants: VaphiVaphiSt2_B_A_FDSW210055054-2r	GG	4419	4420	2	CA -> GG	412 -> 416	Substitution	99.80%	0					
Variants: VaphiVaphiSt2_B_A_FDSW210055054-2r	AC	4423	4424	2	GA -> AC	433 -> 442	Substitution	99.30%	0					
Variants: VaphiVaphiSt2_B_A_FDSW210055054-2r	G	4427	4426	0	#NAME?	467	Insertion	99.80%	0					
Variants: VaphiVaphiSt2_B_A_FDSW210055054-2r	TG	4431	4432	2	GA -> TG	498 -> 505	Substitution	99.80%	0					
Variants: VaphiVaphiSt2_B_A_FDSW210055054-2r	A	4434	4433	0	#NAME?	510	Insertion	100.00%	0					

Variants: VaphiVaphiSt2_B_A_FDSW21 0055054-2r	CA	4435	4436	2	GT -> CA	520 -> 524	Substitution	100.00%	0
Variants: VaphiVaphiSt2_B_A_FDSW21 0055054-2r	A	4668	4668	1	G -> A	452	SNP (transition)	25.90%	1.10E-287
Variants: VaphiVaphiSt2_B_A_FDSW21 0055054-2r	A	4973	4973	1	G -> A	93	SNP (transition)	61.30%	2.40E-174
Variants: VaphiVaphiSt2_B_A_FDSW21 0055054-2r	C	4975	4974	0	+C	88	Insertion	94.30%	4.90E-267
Variants: VaphiVaphiSt2_B_A_FDSW21 0055054-2r	G	4975	4974	0	#NAME?	86	Insertion	54.70%	2.90E-145
Variants: VaphiVaphiSt2_B_A_FDSW21 0055054-2r	T	4975	4974	0	(T)3 -> (T)4	86	Insertion (tandem repeat)	39.50%	3.90E-82
Variants: VaphiVaphiSt2_B_A_FDSW21 0055054-2r	AA	4975	4976	2	TT -> AA	84	Substitution	54.80%	4.50E-133
Variants: VaphiVaphiSt2_B_A_FDSW21 0055054-2r	GAT	4978	4980	3	AGA -> GAT	74 -> 79	Substitution	53.3% -> 54.4%	1.40E-123
Variants: VaphiVaphiSt2_B_A_FDSW21 0055054-2r	A	4981	4981	1	G -> A	73	SNP (transition)	58.90%	6.90E-118
Variants: VaphiVaphiSt2_B_A_FDSW21 0055054-2r	C	4981	4981	1	G -> C	73	SNP (transversion)	39.70%	4.70E-79
Variants: VaphiVaphiSt2_B_A_FDSW21 0055054-2r	AA	4982	4983	2	TG -> AA	71 -> 72	Substitution	93.0% -> 93.1%	1.60E-205
Variants: VaphiVaphiSt2_B_A_FDSW21 0055054-2r	C	4984	4984	1	A -> C	72	SNP (transversion)	51.40%	3.40E-102
Variants: VaphiVaphiSt2_B_A_FDSW21 0055054-2r	A	4985	4985	1	T -> A	72	SNP (transversion)	58.30%	4.10E-119
Variants: VaphiVaphiSt2_B_A_FDSW21 0055054-2r	C	4985	4985	1	T -> C	72	SNP (transition)	40.30%	2.20E-76
Variants: VaphiVaphiSt2_B_A_FDSW21 0055054-2r	A	4986	4986	1	T -> A	65	SNP (transversion)	41.50%	2.80E-66
Variants: VaphiVaphiSt2_B_A_FDSW21 0055054-2r	C	4986	4986	1	T -> C	65	SNP (transition)	56.90%	1.50E-104
Variants: VaphiVaphiSt2_B_A_FDSW21 0055054-2r	A	4988	4988	1	G -> A	65	SNP (transition)	49.20%	8.90E-88
Variants: VaphiVaphiSt2_B_A_FDSW21 0055054-2r	C	4988	4988	1	G -> C	65	SNP (transversion)	43.10%	3.00E-69
Variants: VaphiVaphiSt2_B_A_FDSW21 0055054-2r	A	4989	4989	1	C -> A	65	SNP (transversion)	83.10%	1.40E-161
Variants: VaphiVaphiSt2_B_A_FDSW21 0055054-2r	AA	4992	4993	2	CG -> AA	58 -> 61	Substitution	86.2% -> 88.5%	1.90E-151
Variants: VaphiVaphiSt2_B_A_FDSW21 0055054-2r	A	4994	4994	1	C -> A	58	SNP (transversion)	44.80%	8.50E-60
Variants: VaphiVaphiSt2_B_A_FDSW21 0055054-2r	A	4995	4995	1	G -> A	57	SNP (transition)	47.40%	4.40E-79

Variants: VaphiVaphiSt2_B_A_FDSW21 0055054-2r	C	4995	4995	1	G -> C	57	SNP (transversion)	36.80%	1.60E-50
Variants: VaphiVaphiSt2_B_A_FDSW21 0055054-2r	A	4996	4996	1	T -> A	54	SNP (transversion)	38.90%	3.20E-53
Variants: VaphiVaphiSt2_B_A_FDSW21 0055054-2r	C	4996	4996	1	T -> C	54	SNP (transition)	44.40%	3.50E-67
Variants: VaphiVaphiSt2_B_A_FDSW21 0055054-2r	A	4997	4997	1	G -> A	53	SNP (transition)	43.40%	2.00E-66
Variants: VaphiVaphiSt2_B_A_FDSW21 0055054-2r	C	4997	4997	1	G -> C	53	SNP (transversion)	37.70%	2.00E-48
Variants: VaphiVaphiSt2_B_A_FDSW21 0055054-2r	A	4998	4998	1	G -> A	52	SNP (transition)	38.50%	1.20E-50
Variants: VaphiVaphiSt2_B_A_FDSW21 0055054-2r	T	4998	4998	1	G -> T	52	SNP (transversion)	36.50%	1.50E-49
Variants: VaphiVaphiSt2_B_A_FDSW21 0055054-2r	T	4999	4999	1	C -> T	49	SNP (transition)	71.40%	6.70E-101
Variants: VaphiVaphiSt2_B_A_FDSW21 0055054-2r	C	5000	5000	1	A -> C	46	SNP (transversion)	30.40%	2.40E-38
Variants: VaphiVaphiSt2_B_A_FDSW21 0055054-2r	T	5000	5000	1	A -> T	46	SNP (transversion)	45.70%	8.50E-49
Variants: VaphiVaphiSt2_B_A_FDSW21 0055054-2r	A	5001	5001	1	T -> A	43	SNP (transversion)	25.60%	2.30E-28
Variants: VaphiVaphiSt2_B_A_FDSW21 0055054-2r	C	5001	5001	1	T -> C	43	SNP (transition)	39.50%	4.10E-40
Variants: VaphiVaphiSt2_B_A_FDSW21 0055054-2r	A	5002	5002	1	T -> A	38	SNP (transversion)	60.50%	7.70E-62
Variants: VaphiVaphiSt2_B_A_FDSW21 0055054-2r	AG	5003	5004	2	TT -> AG	35 -> 38	Substitution	39.5% -> 40.0%	2.30E-33
Variants: VaphiVaphiSt2_B_A_FDSW21 0055054-2r	GT	5006	5007	2	AC -> GT	32	Substitution	46.90%	5.60E-37
Variants: VaphiVaphiSt2_B_A_FDSW21 0055054-2r	C	5009	5009	1	A -> C	29	SNP (transversion)	44.80%	5.20E-28
Variants: VaphiVaphiSt2_B_A_FDSW21 0055054-2r	TG	5011	5012	2	GC -> TG	26 -> 28	Substitution	38.5% -> 42.9%	5.20E-23
Variants: VaphiVaphiSt2_B_A_FDSW21 0055054-2r	C	5014	5014	1	T -> C	28	SNP (transition)	28.60%	1.20E-16
Variants: VaphiVaphiSt2_B_A_FDSW21 0055054-2r	TT	5016	5017	2	GG -> TT	28 -> 29	Substitution	27.6% -> 32.1%	4.20E-18
Variants: VaphiVaphiSt2_B_A_FDSW21 0055054-2r	C	5579	5579	1	G -> C	36	SNP (transversion)	100.00%	1.60E-119
Variants: VaphiVaphiSt2_B_A_FDSW21 0055054-2r	T	5581	5581	1	A -> T	34	SNP (transversion)	100.00%	1.60E-109
Variants: VaphiVaphiSt2_B_A_FDSW21 0055054-2r	CG	5583	5584	2	TA -> CG	33 -> 34	Substitution	100.00%	1.60E-109

Variants: VaphiVaphiSt2_B_A_FDSW210055054-2r	AGG	5615	5617	3	TTA -> AGG	22 -> 26	Substitution	30.8% -> 36.4%	8.10E-10
Variants: VaphiVaphiSt2_B_A_FDSW210055054-2r	T	5618	5618	1	G -> T	22	SNP (transversion)	63.60%	2.00E-34
Variants: VaphiVaphiSt2_B_A_FDSW210055054-2r	G	5619	5619	1	T -> G	23	SNP (transversion)	100.00%	3.20E-58
Variants: VaphiVaphiSt2_B_A_FDSW210055054-2r	A	5620	5620	1	C -> A	24	SNP (transversion)	41.70%	1.70E-14
Variants: VaphiVaphiSt2_B_A_FDSW210055054-2r	T	5620	5620	1	C -> T	24	SNP (transition)	54.20%	9.80E-31
Variants: VaphiVaphiSt2_B_A_FDSW210055054-2r	T	5622	5622	1	C -> T	23	SNP (transition)	95.70%	3.60E-52
Variants: VaphiVaphiSt2_B_A_FDSW210055054-2r	A	5624	5623	0	#NAME?	24	Insertion	50.00%	6.40E-22
Variants: VaphiVaphiSt2_B_A_FDSW210055054-2r	T	5624	5623	0	(T)2 -> (T)3	24	Insertion (tandem repeat)	50.00%	3.70E-17
Variants: VaphiVaphiSt2_B_A_FDSW210055054-2r	A	5624	5623	0	#NAME?	24	Insertion	45.80%	1.20E-19
Variants: VaphiVaphiSt2_B_A_FDSW210055054-2r	G	5624	5623	0	#NAME?	24	Insertion	50.00%	3.70E-17
Variants: VaphiVaphiSt2_B_A_FDSW210055054-2r	A	5624	5624	1	T -> A	25	SNP (transversion)	52.00%	9.00E-19
Variants: VaphiVaphiSt2_B_A_FDSW210055054-2r	A	5625	5625	1	T -> A	27	SNP (transversion)	51.90%	4.30E-20
Variants: VaphiVaphiSt2_B_A_FDSW210055054-2r	G	5625	5625	1	T -> G	27	SNP (transversion)	40.70%	3.20E-22
Variants: VaphiVaphiSt2_B_A_FDSW210055054-2r	A	5626	5626	1	G -> A	26	SNP (transition)	53.80%	2.10E-20
Variants: VaphiVaphiSt2_B_A_FDSW210055054-2r	G	5627	5627	1	A -> G	26	SNP (transition)	38.50%	5.10E-19
Variants: VaphiVaphiSt2_B_A_FDSW210055054-2r	T	5630	5630	1	A -> T	27	SNP (transversion)	25.90%	3.20E-10
Variants: VaphiVaphiSt2_B_A_FDSW210055054-2r	T	5632	5632	1	A -> T	26	SNP (transversion)	88.50%	2.50E-43
Variants: VaphiVaphiSt2_B_A_FDSW210055054-2r	T	5633	5633	1	A -> T	25	SNP (transversion)	64.00%	1.90E-26
Variants: VaphiVaphiSt2_B_A_FDSW210055054-2r	ATGAA	5634	5638	5	TCATT -> ATGAA	22 -> 25	Substitution	69.6% -> 78.3%	2.30E-27
Variants: VaphiVaphiSt2_B_A_FDSW210055054-2r	AG	5645	5646	2	TC -> AG	33 -> 38	Substitution	55.3% -> 63.6%	2.60E-36
Variants: VaphiVaphiSt2_B_A_FDSW210055054-2r	A	5648	5648	1	C -> A	48	SNP (transversion)	31.30%	5.00E-13
Variants: VaphiVaphiSt2_B_A_FDSW210055054-2r		5648	5648	1	(C)3 -> (C)2	48	Deletion (tandem repeat)	43.80%	1.50E-09

Variants: VaphiVaphiSt2_B_A_FDSW210055054-2r	C	5653	5653	1	G -> C	76	SNP (transversion)	27.60%	2.00E-20
Variants: VaphiVaphiSt2_B_A_FDSW210055054-2r	T	5653	5653	1	G -> T	76	SNP (transversion)	42.10%	5.50E-53
Variants: VaphiVaphiSt2_B_A_FDSW210055054-2r	GC	5658	5657	0	#NAME?	100	Insertion	28.00%	2.40E-43
Variants: VaphiSt2_B_FDSW210055056-1r	TGGT	79	82	4	CCTG -> TGGT	88 -> 93	Substitution	75.0% -> 80.6%	1.60E-140
Variants: VaphiSt2_B_FDSW210055056-1r	G	84	84	1	A -> G	81	SNP (transition)	90.10%	2.00E-165
Variants: VaphiSt2_B_FDSW210055056-1r	G	86	86	1	C -> G	77	SNP (transversion)	67.50%	2.60E-100
Variants: VaphiSt2_B_FDSW210055056-1r	G	87	87	1	A -> G	77	SNP (transition)	74.00%	1.10E-113
Variants: VaphiSt2_B_FDSW210055056-1r	GG	88	89	2	TC -> GG	73 -> 78	Substitution	46.2% -> 50.7%	2.80E-61
Variants: VaphiSt2_B_FDSW210055056-1r	C	91	91	1	T -> C	86	SNP (transition)	37.20%	1.10E-47
Variants: VaphiSt2_B_FDSW210055056-1r	GGA	93	95	3	CAC -> GGA	86 -> 91	Substitution	25.8% -> 29.5%	7.80E-30
Variants: VaphiSt2_B_FDSW210055056-1r	G	96	96	1	T -> G	90	SNP (transversion)	32.20%	8.70E-50
Variants: VaphiSt2_B_FDSW210055056-1r	C	102	102	1	T -> C	102	SNP (transition)	65.70%	1.80E-107
Variants: VaphiSt2_B_FDSW210055056-1r	C	108	108	1	A -> C	137	SNP (transversion)	32.10%	1.50E-61
Variants: VaphiSt2_B_FDSW210055056-1r	G	120	120	1	A -> G	213	SNP (transition)	60.10%	9.60E-171
Variants: VaphiSt2_B_FDSW210055056-1r	T	126	126	1	C -> T	247	SNP (transition)	30.00%	3.10E-85
Variants: VaphiSt2_B_FDSW210055056-1r	T	129	129	1	C -> T	264	SNP (transition)	87.90%	0
Variants: VaphiSt2_B_FDSW210055056-1r	A	132	132	1	C -> A	276	SNP (transversion)	27.50%	2.20E-62
Variants: VaphiSt2_B_FDSW210055056-1r	G	132	132	1	C -> G	276	SNP (transversion)	54.00%	5.20E-292
Variants: VaphiSt2_B_FDSW210055056-1r	T	144	144	1	C -> T	337	SNP (transition)	31.20%	2.90E-153
Variants: VaphiSt2_B_FDSW210055056-1r	T	147	147	1	C -> T	347	SNP (transition)	50.70%	1.20E-215
Variants: VaphiSt2_B_FDSW210055056-1r	A	159	159	1	G -> A	432	SNP (transition)	84.00%	0
Variants: VaphiSt2_B_FDSW210055056-1r	A	165	165	1	T -> A	470	SNP (transversion)	62.60%	0

Variants: VaphiSt2_B_FDSW210055056-1r	A	183	183	1	G -> A	607	SNP (transition)	41.00%	0
Variants: VaphiSt2_B_FDSW210055056-1r	A	192	192	1	G -> A	665	SNP (transition)	36.40%	0
Variants: VaphiSt2_B_FDSW210055056-1r	A	201	201	1	G -> A	713	SNP (transition)	25.70%	0
Variants: VaphiSt2_B_FDSW210055056-1r	G	216	216	1	A -> G	803	SNP (transition)	28.30%	0
Variants: VaphiSt2_B_FDSW210055056-1r	T	225	225	1	A -> T	851	SNP (transversion)	50.90%	0
Variants: VaphiSt2_B_FDSW210055056-1r	C	237	237	1	T -> C	897	SNP (transition)	33.00%	0
Variants: VaphiSt2_B_FDSW210055056-1r	C	240	240	1	T -> C	895	SNP (transition)	39.00%	0
Variants: VaphiSt2_B_FDSW210055056-1r	A	294	294	1	G -> A	886	SNP (transition)	40.70%	0
Variants: VaphiSt2_B_FDSW210055056-1r	A	303	303	1	T -> A	919	SNP (transversion)	27.90%	0
Variants: VaphiSt2_B_FDSW210055056-1r	G	306	306	1	A -> G	930	SNP (transition)	26.80%	0
Variants: VaphiSt2_B_FDSW210055056-1r	A	357	357	1	G -> A	1081	SNP (transition)	55.00%	0
Variants: VaphiSt2_B_FDSW210055056-1r	G	363	363	1	A -> G	1099	SNP (transition)	57.40%	0
Variants: VaphiSt2_B_FDSW210055056-1r	C	369	369	1	T -> C	1136	SNP (transition)	83.20%	0
Variants: VaphiSt2_B_FDSW210055056-1r	A	371	371	1	G -> A	1140	SNP (transition)	56.60%	0
Variants: VaphiSt2_B_FDSW210055056-1r	G	387	387	1	C -> G	1231	SNP (transversion)	58.60%	0
Variants: VaphiSt2_B_FDSW210055056-1r	A	393	393	1	G -> A	1263	SNP (transition)	27.20%	0
Variants: VaphiSt2_B_FDSW210055056-1r	C	393	393	1	G -> C	1263	SNP (transversion)	46.80%	0
Variants: VaphiSt2_B_FDSW210055056-1r	C	396	396	1	A -> C	1293	SNP (transversion)	37.50%	0
Variants: VaphiSt2_B_FDSW210055056-1r	G	420	420	1	A -> G	1419	SNP (transition)	34.70%	0
Variants: VaphiSt2_B_FDSW210055056-1r	T	420	420	1	A -> T	1419	SNP (transversion)	32.40%	0
Variants: VaphiSt2_B_FDSW210055056-1r	A	422	422	1	T -> A	1418	SNP (transversion)	32.50%	0
Variants: VaphiSt2_B_FDSW210055056-1r	A	423	423	1	G -> A	1432	SNP (transition)	66.00%	0

Variants: VaphiSt2_B_FDSW210055056-1r	A	426	426	1	G -> A	1456	SNP (transition)	65.10%	0
Variants: VaphiSt2_B_FDSW210055056-1r	G	438	438	1	A -> G	1513	SNP (transition)	50.40%	0
Variants: VaphiSt2_B_FDSW210055056-1r	A	456	456	1	G -> A	1515	SNP (transition)	51.50%	0
Variants: VaphiSt2_B_FDSW210055056-1r	C	462	462	1	T -> C	1532	SNP (transition)	26.20%	0
Variants: VaphiSt2_B_FDSW210055056-1r	C	489	489	1	T -> C	1513	SNP (transition)	43.40%	0
Variants: VaphiSt2_B_FDSW210055056-1r	C	498	498	1	G -> C	1469	SNP (transversion)	27.90%	0
Variants: VaphiSt2_B_FDSW210055056-1r	C	513	513	1	T -> C	1398	SNP (transition)	51.00%	0
Variants: VaphiSt2_B_FDSW210055056-1r	C	516	516	1	T -> C	1393	SNP (transition)	33.50%	0
Variants: VaphiSt2_B_FDSW210055056-1r	T	531	531	1	C -> T	1331	SNP (transition)	27.30%	0
Variants: VaphiSt2_B_FDSW210055056-1r	C	537	537	1	G -> C	1295	SNP (transversion)	53.70%	0
Variants: VaphiSt2_B_FDSW210055056-1r	G	540	540	1	A -> G	1278	SNP (transition)	34.50%	0
Variants: VaphiSt2_B_FDSW210055056-1r	A	552	552	1	T -> A	1264	SNP (transversion)	33.70%	0
Variants: VaphiSt2_B_FDSW210055056-1r	A	564	564	1	C -> A	1217	SNP (transversion)	27.40%	0
Variants: VaphiSt2_B_FDSW210055056-1r	G	588	588	1	A -> G	1100	SNP (transition)	34.40%	0
Variants: VaphiSt2_B_FDSW210055056-1r	C	663	663	1	T -> C	612	SNP (transition)	69.00%	0
Variants: VaphiSt2_B_FDSW210055056-1r	G	677	677	1	A -> G	509	SNP (transition)	71.90%	0
Variants: VaphiSt2_B_FDSW210055056-1r	C	684	684	1	T -> C	456	SNP (transition)	70.60%	0
Variants: VaphiSt2_B_FDSW210055056-1r	A	687	687	1	G -> A	432	SNP (transition)	71.30%	0
Variants: VaphiSt2_B_FDSW210055056-1r	T	690	690	1	A -> T	418	SNP (transversion)	73.20%	0
Variants: VaphiSt2_B_FDSW210055056-1r	A	693	693	1	G -> A	395	SNP (transition)	74.90%	0
Variants: VaphiSt2_B_FDSW210055056-1r	A	708	708	1	G -> A	294	SNP (transition)	77.60%	0
Variants: VaphiSt2_B_FDSW210055056-1r	T	723	723	1	C -> T	209	SNP (transition)	82.30%	0

Variants: VaphiSt2_B_FDSW210055056 -1r	C	726	726	1	T -> C	191	SNP (transition)	82.70%	0					
Variants: VaphiSt2_B_FDSW210055056 -1r	AC	745	746	2	GA -> AC	104 -> 110	Substitution	29.1% -> 32.7%	5.90E-52					
Variants: VaphiSt2_B_FDSW210055056 -1r	C	747	747	1	T -> C	105	SNP (transition)	61.90%	1.50E-153					
Variants: VaphiSt2_B_FDSW210055056 -1r	C	749	749	1	T -> C	106	SNP (transition)	50.90%	3.60E-121					
Variants: VaphiSt2_B_FDSW210055056 -1r	C	750	750	1	A -> C	107	SNP (transversion)	28.00%	2.90E-61					
Variants: VaphiSt2_B_FDSW210055056 -1r	G	750	750	1	A -> G	107	SNP (transition)	52.30%	2.50E-115					
Variants: VaphiSt2_B_FDSW210055056 -1r	TTCGA	751	755	5	CCTTG -> TTCGA	98 -> 108	Substitution	23.5% -> 40.7%	6.70E-28					
Variants: VaphiSt2_B_FDSW210055056 -1r	C	756	756	1	G -> C	101	SNP (transversion)	47.50%	1.70E-115					
Variants: VaphiSt2_B_FDSW210055056 -1r	A	768	768	1	T -> A	149	SNP (transversion)	54.40%	2.00E-208					
Variants: VaphiSt2_B_FDSW210055056 -1r	T	790	790	1	G -> T	298	SNP (transversion)	28.50%	9.70E-180	R -> L	hypothetical protein CDS	hypothetica l protein	Substituti on	KLI69 879.1
Variants: VaphiSt2_B_FDSW210055056 -1r	A	795	795	1	G -> A	333	SNP (transition)	51.70%	0	A -> T	hypothetical protein CDS	hypothetica l protein	Substituti on	KLI69 879.1
Variants: VaphiSt2_B_FDSW210055056 -1r	AAT	801	803	2	CAC -> AAT	378 -> 390	Substitution	70.3% -> 72.5%	0	H -> N	hypothetical protein CDS	hypothetica l protein	Substituti on	KLI69 879.1
Variants: VaphiSt2_B_FDSW210055056 -1r	C	834	834	1	T -> C	547	SNP (transition)	75.90%	0	Y -> H	hypothetical protein CDS	hypothetica l protein	Substituti on	KLI69 879.1
Variants: VaphiSt2_B_FDSW210055056 -1r	G	888	888	1	A -> G	830	SNP (transition)	29.50%	0	I -> V	hypothetical protein CDS	hypothetica l protein	Substituti on	KLI69 879.1
Variants: VaphiSt2_B_FDSW210055056 -1r	C	897	897	1	T -> C	844	SNP (transition)	56.50%	0	C -> R	hypothetical protein CDS	hypothetica l protein	Substituti on	KLI69 879.1
Variants: VaphiSt2_B_FDSW210055056 -1r	C	900	900	1	G -> C	854	SNP (transversion)	56.80%	0	D -> H	hypothetical protein CDS	hypothetica l protein	Substituti on	KLI69 879.1
Variants: VaphiSt2_B_FDSW210055056 -1r	C	942	942	1	G -> C	817	SNP (transversion)	29.50%	0	G -> R	hypothetical protein CDS	hypothetica l protein	Substituti on	KLI69 879.1
Variants: VaphiSt2_B_FDSW210055056 -1r	TGT	966	968	2	AGC -> TGT	788 -> 795	Substitution	48.5% -> 48.7%	0	S -> C	hypothetical protein CDS	hypothetica l protein	Substituti on	KLI69 879.1
Variants: VaphiSt2_B_FDSW210055056 -1r	G	969	969	1	C -> G	799	SNP (transversion)	33.90%	0	P -> A	hypothetical protein CDS	hypothetica l protein	Substituti on	KLI69 879.1
Variants: VaphiSt2_B_FDSW210055056 -1r	A	976	976	1	T -> A	805	SNP (transversion)	33.70%	0	V -> E	hypothetical protein CDS	hypothetica l protein	Substituti on	KLI69 879.1
Variants: VaphiSt2_B_FDSW210055056 -1r	A	1008	1008	1	G -> A	751	SNP (transition)	37.40%	0	V -> I	hypothetical protein CDS	hypothetica l protein	Substituti on	KLI69 879.1
Variants: VaphiSt2_B_FDSW210055056 -1r	C	1035	1035	1	G -> C	794	SNP (transversion)	32.20%	0	G -> R	hypothetical protein CDS	hypothetica l protein	Substituti on	KLI69 879.1

Variants: VaphiSt2_B_FDSW210055056 -1r	A	1041	1041	1	G -> A	812	SNP (transition)	36.90%	0	A -> T	hypothetical protein CDS	hypothetica l protein	Substituti on	KLI69 879.1
Variants: VaphiSt2_B_FDSW210055056 -1r	C	1049	1049	1	T -> C	819	SNP (transition)	30.90%	0		hypothetical protein CDS	hypothetica l protein	None	KLI69 879.1
Variants: VaphiSt2_B_FDSW210055056 -1r	G	1083	1083	1	A -> G	885	SNP (transition)	37.60%	0	K -> E	hypothetical protein CDS	hypothetica l protein	Substituti on	KLI69 879.1
Variants: VaphiSt2_B_FDSW210055056 -1r	A	1100	1100	1	G -> A	921	SNP (transition)	38.00%	0		hypothetical protein CDS	hypothetica l protein	None	KLI69 879.1
Variants: VaphiSt2_B_FDSW210055056 -1r	A	1101	1101	1	G -> A	926	SNP (transition)	36.50%	0					
Variants: VaphiSt2_B_FDSW210055056 -1r	C	1104	1104	1	G -> C	933	SNP (transversion)	38.30%	0					
Variants: VaphiSt2_B_FDSW210055056 -1r	T	1116	1116	1	C -> T	929	SNP (transition)	38.40%	0		hypothetical protein CDS	hypothetica l protein	None	KLI69 880.1
Variants: VaphiSt2_B_FDSW210055056 -1r	T	1119	1119	1	C -> T	928	SNP (transition)	38.50%	0		hypothetical protein CDS	hypothetica l protein	None	KLI69 880.1
Variants: VaphiSt2_B_FDSW210055056 -1r	G	1135	1135	1	T -> G	943	SNP (transversion)	60.40%	0	F -> V	hypothetical protein CDS	hypothetica l protein	Substituti on	KLI69 880.1
Variants: VaphiSt2_B_FDSW210055056 -1r	TGTG	1173	1176	3	CATA -> TGTG	977 -> 985	Substitution	44.5% -> 45.8%	0	TI -> TV	hypothetical protein CDS	hypothetica l protein	Substituti on	KLI69 880.1
Variants: VaphiSt2_B_FDSW210055056 -1r	C	1182	1182	1	T -> C	963	SNP (transition)	46.30%	0		hypothetical protein CDS	hypothetica l protein	None	KLI69 880.1
Variants: VaphiSt2_B_FDSW210055056 -1r	G	1194	1194	1	A -> G	943	SNP (transition)	25.20%	0		hypothetical protein CDS	hypothetica l protein	None	KLI69 880.1
Variants: VaphiSt2_B_FDSW210055056 -1r	G	1215	1215	1	A -> G	869	SNP (transition)	52.50%	0		hypothetical protein CDS	hypothetica l protein	None	KLI69 880.1
Variants: VaphiSt2_B_FDSW210055056 -1r	G	1218	1218	1	A -> G	846	SNP (transition)	44.90%	0		hypothetical protein CDS	hypothetica l protein	None	KLI69 880.1
Variants: VaphiSt2_B_FDSW210055056 -1r	G	1221	1221	1	C -> G	831	SNP (transversion)	44.60%	0		hypothetical protein CDS	hypothetica l protein	None	KLI69 880.1
Variants: VaphiSt2_B_FDSW210055056 -1r	GG	1232	1233	2	AC -> GG	725 -> 735	Substitution	40.0% -> 40.6%	0	N -> R	hypothetical protein CDS	hypothetica l protein	Substituti on	KLI69 880.1
Variants: VaphiSt2_B_FDSW210055056 -1r	T	1235	1235	1	C -> T	706	SNP (transition)	40.50%	0	A -> V	hypothetical protein CDS	hypothetica l protein	Substituti on	KLI69 880.1
Variants: VaphiSt2_B_FDSW210055056 -1r	G	1269	1269	1	A -> G	501	SNP (transition)	64.70%	0	I -> M	hypothetical protein CDS	hypothetica l protein	Substituti on	KLI69 880.1
Variants: VaphiSt2_B_FDSW210055056 -1r	G	1275	1275	1	A -> G	468	SNP (transition)	66.00%	0		hypothetical protein CDS	hypothetica l protein	None	KLI69 880.1
Variants: VaphiSt2_B_FDSW210055056 -1r	T	1287	1287	1	A -> T	402	SNP (transversion)	52.50%	0		hypothetical protein CDS	hypothetica l protein	None	KLI69 880.1
Variants: VaphiSt2_B_FDSW210055056 -1r	T	1290	1290	1	C -> T	384	SNP (transition)	51.60%	0		hypothetical protein CDS	hypothetica l protein	None	KLI69 880.1
Variants: VaphiSt2_B_FDSW210055056 -1r	G	1293	1293	1	C -> G	358	SNP (transversion)	52.50%	0	D -> E	hypothetical protein CDS	hypothetica l protein	Substituti on	KLI69 880.1

Variants: VaphiSt2_B_FDSW210055056-1r	ATTG	1296	1299	4	CAAC -> ATTG	326 -> 338	Substitution	51.4% -> 52.1%	0	TN -> TL	hypothetical protein CDS	hypothetical protein	Substitution	KLI69880.1
Variants: VaphiSt2_B_FDSW210055056-1r	A	1305	1305	1	G -> A	288	SNP (transition)	50.30%	0		hypothetical protein CDS	hypothetical protein	None	KLI69880.1
Variants: VaphiSt2_B_FDSW210055056-1r	A	1311	1311	1	G -> A	255	SNP (transition)	30.60%	8.00E-207		hypothetical protein CDS	hypothetical protein	None	KLI69880.1
Variants: VaphiSt2_B_FDSW210055056-1r	T	1314	1314	1	G -> T	229	SNP (transversion)	29.70%	4.10E-159		hypothetical protein CDS	hypothetical protein	None	KLI69880.1
Variants: VaphiSt2_B_FDSW210055056-1r	G	1317	1317	1	C -> G	215	SNP (transversion)	26.50%	9.20E-142	D -> E	hypothetical protein CDS	hypothetical protein	Substitution	KLI69880.1
Variants: VaphiSt2_B_FDSW210055056-1r	GCTA	1320	1323	4	TGAG -> GCTA	179 -> 202	Substitution	29.7% -> 33.5%	6.40E-139	TE -> TL	hypothetical protein CDS	hypothetical protein	Substitution	KLI69880.1
Variants: VaphiSt2_B_FDSW210055056-1r	A	1324	1324	1	C -> A	177	SNP (transversion)	34.50%	5.00E-194	H -> N	hypothetical protein CDS	hypothetical protein	Substitution	KLI69880.1
Variants: VaphiSt2_B_FDSW210055056-1r	AAG	1324	1326	2	CAC -> AAG	171 -> 177	Substitution	57.1% -> 59.1%	2.7E-315	H -> K	hypothetical protein CDS	hypothetical protein	Substitution	KLI69880.1
Variants: VaphiSt2_B_FDSW210055056-1r	TT	1327	1328	2	AC -> TT	165 -> 170	Substitution	57.1% -> 58.8%	1.20E-298	T -> F	hypothetical protein CDS	hypothetical protein	Substitution	KLI69880.1
Variants: VaphiSt2_B_FDSW210055056-1r	AGTGCC	1330	1335	5	TTAACA -> AGTGCC	151 -> 161	Substitution	53.6% -> 62.7%	3.10E-248	LT -> SA	hypothetical protein CDS	hypothetical protein	Substitution	KLI69880.1
Variants: VaphiSt2_B_FDSW210055056-1r	TCG	1339	1341	3	CGT -> TCG	123 -> 127	Substitution	36.6% -> 40.2%	2.80E-124	R -> S	hypothetical protein CDS	hypothetical protein	Substitution	KLI69880.1
Variants: VaphiSt2_B_FDSW210055056-1r	CT	1342	1343	2	TA -> CT	115 -> 120	Substitution	33.3% -> 34.8%	1.40E-105		hypothetical protein CDS	hypothetical protein	Extension	KLI69880.1
Variants: VaphiSt2_B_FDSW210055056-1r	T	1345	1345	1	G -> T	105	SNP (transversion)	36.20%	5.60E-105					
Variants: VaphiSt2_B_FDSW210055056-1r	TT	1347	1348	2	AA -> TT	93 -> 97	Substitution	47.3% -> 49.5%	7.00E-128					
Variants: VaphiSt2_B_FDSW210055056-1r	AAT	1351	1353	3	GGA -> AAT	81 -> 84	Substitution	34.6% -> 39.3%	2.70E-74					
Variants: VaphiSt2_B_FDSW210055056-1r	CCGAAACGGATGA	2114	2126	12	ATTTTGAAAAAAT -> CCGAAACGGATGA	43 -> 50	Substitution	20.9% -> 48.9%	2.80E-27	DFEKI -> AETDD	hypothetical protein CDS	hypothetical protein	Substitution	KLI69881.1
Variants: VaphiSt2_B_FDSW210055056-1r	AA	2128	2129	2	GC -> AA	50 -> 52	Substitution	62.0% -> 63.5%	9.50E-96	A -> N	hypothetical protein CDS	hypothetical protein	Substitution	KLI69881.1
Variants: VaphiSt2_B_FDSW210055056-1r	CAC	2131	2133	2	TAA -> CAC	54 -> 55	Substitution	65.5% -> 66.7%	9.30E-115		hypothetical protein CDS	hypothetical protein	Extension	KLI69881.1
Variants: VaphiSt2_B_FDSW210055056-1r	T	2135	2135	1	A -> T	63	SNP (transversion)	68.30%	4.20E-135					
Variants: VaphiSt2_B_FDSW210055056-1r	CTG	2137	2139	3	AGC -> CTG	67 -> 69	Substitution	63.8% -> 65.7%	4.10E-136					
Variants: VaphiSt2_B_FDSW210055056-1r	AG	2141	2142	2	TT -> AG	73 -> 77	Substitution	55.8% -> 58.9%	3.50E-122					
Variants: VaphiSt2_B_FDSW210055056-1r	AG	2143	2144	2	TA -> AG	77 -> 81	Substitution	27.3% -> 30.9%	9.90E-58					

Variants: VaphiSt2_B_FDSW210055056 -1r	G	2145	2145	1	A -> G	81	SNP (transition)	87.70%	5.90E-237
Variants: VaphiSt2_B_FDSW210055056 -1r	T	2146	2146	1	G -> T	82	SNP (transversion)	35.40%	4.90E-83
Variants: VaphiSt2_B_FDSW210055056 -1r	G	2147	2147	1	A -> G	82	SNP (transition)	36.60%	2.20E-74
Variants: VaphiSt2_B_FDSW210055056 -1r	T	2147	2147	1	A -> T	82	SNP (transversion)	53.70%	1.30E-135
Variants: VaphiSt2_B_FDSW210055056 -1r	G	2149	2148	0	#NAME?	82	Insertion	53.70%	3.40E-131
Variants: VaphiSt2_B_FDSW210055056 -1r	GAG	2902	2904	3	TGA -> GAG	20 -> 21	Substitution	65.0% -> 75.0%	9.40E-26
Variants: VaphiSt2_B_FDSW210055056 -1r	GGA	2906	2908	3	TCG -> GGA	18 -> 21	Substitution	50.0% -> 57.1%	5.70E-15
Variants: VaphiSt2_B_FDSW210055056 -1r	GGGA	2910	2913	4	AACG -> GGGA	16 -> 18	Substitution	31.3% -> 44.4%	4.00E-07
Variants: VaphiSt2_B_FDSW210055056-1r		3097	3097	1	#NAME?	366	Deletion	42.10%	1.40E-157
Variants: VaphiSt2_B_FDSW210055056 -1r	TTAG	3449	3452	4	GCGT -> TTAG	34 -> 35	Substitution	26.5% -> 51.4%	1.70E-08
Variants: VaphiSt2_B_FDSW210055056 -1r	G	3456	3456	1	T -> G	40	SNP (transversion)	35.00%	7.60E-20
Variants: VaphiSt2_B_FDSW210055056 -1r	T	3659	3659	1	G -> T	393	SNP (transversion)	48.30%	0
Variants: VaphiSt2_B_FDSW210055056 -1r	C	3758	3758	1	A -> C	339	SNP (transversion)	48.10%	0
Variants: VaphiSt2_B_FDSW210055056 -1r	G	3778	3778	1	A -> G	346	SNP (transition)	52.00%	0
Variants: VaphiSt2_B_FDSW210055056 -1r	T	3780	3780	1	C -> T	344	SNP (transition)	51.50%	0
Variants: VaphiSt2_B_FDSW210055056 -1r	C	3852	3852	1	T -> C	376	SNP (transition)	52.40%	0
Variants: VaphiSt2_B_FDSW210055056 -1r	G	3864	3864	1	A -> G	371	SNP (transition)	48.80%	0
Variants: VaphiSt2_B_FDSW210055056 -1r	T	3922	3922	1	C -> T	284	SNP (transition)	40.80%	7.10E-290
Variants: VaphiSt2_B_FDSW210055056 -1r	T	4037	4037	1	G -> T	40	SNP (transversion)	70.00%	8.10E-50
Variants: VaphiSt2_B_FDSW210055056 -1r	AA	4047	4048	2	TT -> AA	28	Substitution	100.00%	6.30E-54
Variants: VaphiSt2_B_FDSW210055056 -1r	GTGGTA	4050	4055	6	CGCTGC -> GTGGTA	24 -> 25	Substitution	100.00%	1.00E-48
Variants: VaphiSt2_B_FDSW210055056 -1r	GG	4059	4060	2	TT -> GG	23	Substitution	100.00%	1.00E-46

Variants: VaphiSt2_B_FDSW210055056-1r	GGC	4062	4064	3	TCA -> GGC	19 -> 21	Substitution	100.00%	1.00E-38					
Variants: VaphiSt2_B_FDSW210055056-1r	CGGTATTGCGTT	4067	4078	12	ACCGTCGCGCCG -> CGGTATTGCGTT	5 -> 19	Substitution	100.00%	1.00E-07					
Variants: VaphiSt2_B_FDSW210055056-1r	G	4346	4346	1	A -> G	14	SNP (transition)	100.00%	2.50E-20	F -> L	hypothetical protein CDS	hypothetical protein	Substitution	KLI69882.1
Variants: VaphiSt2_B_FDSW210055056-1r	T	4349	4349	1	C -> T	23	SNP (transition)	95.70%	1.40E-34	E -> K	hypothetical protein CDS	hypothetical protein	Substitution	KLI69882.1
Variants: VaphiSt2_B_FDSW210055056-1r	G	4363	4363	1	A -> G	120	SNP (transition)	32.50%	1.70E-105	V -> A	hypothetical protein CDS	hypothetical protein	Substitution	KLI69882.1
Variants: VaphiSt2_B_FDSW210055056-1r	T	4379	4379	1	C -> T	366	SNP (transition)	99.50%	0	A -> T	hypothetical protein CDS	hypothetical protein	Substitution	KLI69882.1
Variants: VaphiSt2_B_FDSW210055056-1r	CATGA	4381	4385	5	TGGTG -> CATGA	396 -> 435	Substitution	99.7% -> 99.8%	0		hypothetical protein CDS	hypothetical protein	Truncation	KLI69882.1
Variants: VaphiSt2_B_FDSW210055056-1r	GG	4388	4389	2	CA -> GG	454 -> 470	Substitution	99.80%	0	PE -> PQ	hypothetical protein CDS	hypothetical protein	Substitution	KLI69882.1
Variants: VaphiSt2_B_FDSW210055056-1r	TGA	4392	4394	2	CGT -> TGA	508 -> 537	Substitution	99.0% -> 99.1%	0	T -> S	hypothetical protein CDS	hypothetical protein	Substitution	KLI69882.1
Variants: VaphiSt2_B_FDSW210055056-1r	GT	4396	4397	2	AA -> GT	559 -> 568	Substitution	99.10%	0	F -> T	hypothetical protein CDS	hypothetical protein	Substitution	KLI69882.1
Variants: VaphiSt2_B_FDSW210055056-1r	A	4401	4401	1	C -> A	613	SNP (transversion)	100.00%	0	M -> V	hypothetical protein CDS	hypothetical protein	Start Codon Loss	KLI69882.1
Variants: VaphiSt2_B_FDSW210055056-1r	A	4404	4406	3	GTT -> A	640	Deletion	99.50%	0					
Variants: VaphiSt2_B_FDSW210055056-1r	GT	4409	4410	2	TC -> GT	667 -> 676	Substitution	99.30%	0					
Variants: VaphiSt2_B_FDSW210055056-1r	GC	4414	4415	2	CT -> GC	715 -> 727	Substitution	100.00%	0					
Variants: VaphiSt2_B_FDSW210055056-1r	A	4417	4417	1	G -> A	752	SNP (transition)	99.90%	0					
Variants: VaphiSt2_B_FDSW210055056-1r	GG	4419	4420	2	CA -> GG	766 -> 779	Substitution	99.6% -> 99.7%	0					
Variants: VaphiSt2_B_FDSW210055056-1r	AC	4423	4424	2	GA -> AC	804 -> 811	Substitution	98.40%	0					
Variants: VaphiSt2_B_FDSW210055056-1r	G	4427	4426	0	#NAME?	846	Insertion	99.90%	0					
Variants: VaphiSt2_B_FDSW210055056-1r	TG	4431	4432	2	GA -> TG	908 -> 910	Substitution	99.80%	0					
Variants: VaphiSt2_B_FDSW210055056-1r	A	4434	4433	0	#NAME?	927	Insertion	99.70%	0					
Variants: VaphiSt2_B_FDSW210055056-1r	CA	4435	4436	2	GT -> CA	951 -> 961	Substitution	99.40%	0					
Variants: VaphiSt2_B_FDSW210055056-1r	A	4668	4668	1	G -> A	991	SNP (transition)	26.50%	0					

Variants: VaphiSt2_B_FDSW210055056-1r	A	4973	4973	1	G -> A	208	SNP (transition)	64.90%	0
Variants: VaphiSt2_B_FDSW210055056-1r	C	4975	4975	1	T -> C	200	SNP (transition)	79.50%	0
Variants: VaphiSt2_B_FDSW210055056-1r	G	4976	4976	1	T -> G	197	SNP (transversion)	64.50%	0
Variants: VaphiSt2_B_FDSW210055056-1r	A	4979	4979	1	G -> A	183	SNP (transition)	77.00%	0
Variants: VaphiSt2_B_FDSW210055056-1r	GA	4980	4981	2	AG -> GA	169 -> 175	Substitution	63.3% -> 64.0%	0
Variants: VaphiSt2_B_FDSW210055056-1r	A	4982	4982	1	T -> A	170	SNP (transversion)	32.40%	1.80E-131
Variants: VaphiSt2_B_FDSW210055056-1r	A	4983	4983	1	G -> A	169	SNP (transition)	79.90%	0
Variants: VaphiSt2_B_FDSW210055056-1r	A	4985	4985	1	T -> A	153	SNP (transversion)	77.10%	0
Variants: VaphiSt2_B_FDSW210055056-1r	A	4986	4986	1	T -> A	149	SNP (transversion)	29.50%	7.90E-108
Variants: VaphiSt2_B_FDSW210055056-1r	C	4986	4986	1	T -> C	149	SNP (transition)	61.70%	4.70E-290
Variants: VaphiSt2_B_FDSW210055056-1r	A	4987	4987	1	C -> A	146	SNP (transversion)	63.00%	6.30E-273
Variants: VaphiSt2_B_FDSW210055056-1r	C	4988	4988	1	G -> C	145	SNP (transversion)	73.80%	0
Variants: VaphiSt2_B_FDSW210055056-1r	AAAA	4992	4995	4	CGCG -> AAAA	121 -> 132	Substitution	74.4% -> 78.0%	6.50E-278
Variants: VaphiSt2_B_FDSW210055056-1r	A	4996	4996	1	T -> A	118	SNP (transversion)	26.30%	1.00E-77
Variants: VaphiSt2_B_FDSW210055056-1r	C	4996	4996	1	T -> C	118	SNP (transition)	54.20%	1.60E-190
Variants: VaphiSt2_B_FDSW210055056-1r	A	4997	4997	1	G -> A	114	SNP (transition)	51.80%	5.70E-180
Variants: VaphiSt2_B_FDSW210055056-1r	C	4997	4997	1	G -> C	114	SNP (transversion)	27.20%	4.70E-72
Variants: VaphiSt2_B_FDSW210055056-1r	A	4998	4998	1	G -> A	110	SNP (transition)	26.40%	7.50E-73
Variants: VaphiSt2_B_FDSW210055056-1r	C	4998	4998	1	G -> C	110	SNP (transversion)	51.80%	5.70E-174
Variants: VaphiSt2_B_FDSW210055056-1r	AT	4999	5000	2	CA -> AT	103 -> 104	Substitution	49.5% -> 50.0%	4.70E-151
Variants: VaphiSt2_B_FDSW210055056-1r	CA	5002	5003	2	TT -> CA	100	Substitution	41.0% -> 43.0%	1.10E-106
Variants: VaphiSt2_B_FDSW210055056-1r	A	5004	5004	1	T -> A	99	SNP (transversion)	63.60%	6.20E-169

Variants: VaphiSt2_B_FDSW210055056 -1r	GTG	5005	5007	3	TAC -> GTG	83 -> 94	Substitution	32.5% -> 36.2%	2.90E-76
Variants: VaphiSt2_B_FDSW210055056 -1r	T	5011	5011	1	G -> T	64	SNP (transversion)	28.10%	1.40E-44
Variants: VaphiSt2_B_FDSW210055056 -1r	C	5579	5579	1	G -> C	93	SNP (transversion)	100.00%	6.3E-317
Variants: VaphiSt2_B_FDSW210055056 -1r	T	5581	5581	1	A -> T	87	SNP (transversion)	100.00%	7.90E-288
Variants: VaphiSt2_B_FDSW210055056 -1r	CG	5583	5584	2	TA -> CG	83 -> 85	Substitution	98.80%	1.30E-277
Variants: VaphiSt2_B_FDSW210055056 -1r	C	5607	5607	1	A -> C	52	SNP (transversion)	28.80%	1.40E-40
Variants: VaphiSt2_B_FDSW210055056 -1r	TT	5609	5610	2	AA -> TT	54	Substitution	31.5% -> 35.2%	3.70E-43
Variants: VaphiSt2_B_FDSW210055056 -1r	AA	5612	5613	2	GC -> AA	55 -> 59	Substitution	41.8% -> 45.8%	1.20E-63
Variants: VaphiSt2_B_FDSW210055056 -1r	GGG	5615	5617	3	TTA -> GGG	58 -> 61	Substitution	50.0% -> 54.1%	7.50E-83
Variants: VaphiSt2_B_FDSW210055056 -1r	A	5618	5618	1	G -> A	65	SNP (transition)	56.90%	3.10E-108
Variants: VaphiSt2_B_FDSW210055056 -1r	T	5618	5618	1	G -> T	65	SNP (transversion)	43.10%	7.40E-61
Variants: VaphiSt2_B_FDSW210055056 -1r	GT	5619	5620	2	TC -> GT	65 -> 67	Substitution	100.00%	1.00E-208
Variants: VaphiSt2_B_FDSW210055056 -1r	C	5621	5621	1	G -> C	65	SNP (transversion)	63.10%	1.60E-122
Variants: VaphiSt2_B_FDSW210055056 -1r	T	5622	5622	1	C -> T	65	SNP (transition)	100.00%	1.00E-208
Variants: VaphiSt2_B_FDSW210055056 -1r	G	5623	5623	1	C -> G	63	SNP (transversion)	65.10%	2.10E-123
Variants: VaphiSt2_B_FDSW210055056 -1r	AA	5624	5625	2	TT -> AA	65	Substitution	96.90%	4.10E-180
Variants: VaphiSt2_B_FDSW210055056 -1r	A	5626	5626	1	G -> A	66	SNP (transition)	68.20%	2.80E-123
Variants: VaphiSt2_B_FDSW210055056 -1r	T	5626	5626	1	G -> T	66	SNP (transversion)	28.80%	3.60E-30
Variants: VaphiSt2_B_FDSW210055056 -1r	GG	5627	5628	2	AT -> GG	66 -> 67	Substitution	25.4% -> 25.8%	3.30E-26
Variants: VaphiSt2_B_FDSW210055056 -1r	T	5632	5632	1	A -> T	78	SNP (transversion)	91.00%	5.20E-183
Variants: VaphiSt2_B_FDSW210055056 -1r	TATG	5633	5636	4	ATCA -> TATG	74 -> 78	Substitution	64.1% -> 68.9%	1.20E-129
Variants: VaphiSt2_B_FDSW210055056 -1r	A	5637	5637	1	T -> A	71	SNP (transversion)	87.30%	7.40E-176

Variants: VaphiSt2_B_FDSW210055056 -1r	A	5638	5638	1	T -> A	73	SNP (transversion)	72.60%	1.10E-152
Variants: VaphiSt2_B_FDSW210055056 -1r	AG	5645	5646	2	TC -> AG	97 -> 104	Substitution	63.5% -> 68.0%	8.80E-177
Variants: VaphiSt2_B_FDSW210055056-1r		5648	5648	1	(C)3 -> (C)2	119	Deletion (tandem repeat)	56.30%	3.80E-61
Variants: VaphiSt2_B_FDSW210055056 -1r	T	5653	5653	1	G -> T	161	SNP (transversion)	51.60%	1.60E-202
Variants: VaphiSt2_B_FDSW210055056 -1r	GC	5658	5657	0	#NAME?	184	Insertion	46.20%	7.70E-202
